# Taxonomic revision of Afrotropical *Laccophilus* Leach, 1815 (Coleoptera, Dytiscidae)

**DOI:** 10.3897/zookeys.542.5975

**Published:** 2015-12-07

**Authors:** Olof Biström, Anders N. Nilsson, Johannes Bergsten

**Affiliations:** 1 Finnish Museum of Natural History, Zoology Unit, PB 17, FI-00014 University of Helsinki, Finland; 2 EMG, Umeå University, SE-90187 Umeå, Sweden; 3 Swedish Museum of Natural History, Department of Zoology, Box 50007, SE-10405 Stockholm, Sweden

**Keywords:** Coleoptera, Dytiscidae, *Laccophilus*, Africa, Madagascar, taxonomy, revision, description, new species

## Abstract

The African species of the genus *Laccophilus* Leach, 1815, are revised, on the basis of study of adult specimens. In all, 105 species are now recognized. A phenetic character-analysis was undertaken, which resulted in a split of the genus into 17 species groups. Diagnoses and a description of each species are given together with keys for identification of species groups and species. We also provide habitus photos, illustration of male genitalia and distribution maps for all species. New species are described as follows: *Laccophilus grossus* **sp. n.** (Angola, Namibia), *Laccophilus rocchii* **sp. n.** (Tanzania, Namibia, Botswana, Mozambique), *Laccophilus ferrugo* **sp. n.** (Mozambique), *Laccophilus furthi* **sp. n.** (Madagascar), *Laccophilus isamberti* **sp. n.** (Madagascar), *Laccophilus inobservatus* **sp. n.** (Gambia, Senegal, Mali, Niger, Sudan, Chad, Ethiopia, Burkina Faso, Ivory Coast, Ghana, Nigeria, Cameroon, Zaire and Asia: Yemen), *Laccophilus cryptos* **sp. n.** (Zaire, Mozambique, Zimbabwe, Namibia, Botswana, South Africa), *Laccophilus enigmaticus* **sp. n.** (Nigeria, Sudan), *Laccophilus bellus* **sp. n.** (Benin, Nigeria), *Laccophilus guentheri* **sp. n.** (Guinea, Ghana), *Laccophilus guineensis* **sp. n.** (Guinea), *Laccophilus decorosus* **sp. n.** (Uganda), *Laccophilus empheres* **sp. n.** (Kenya), *Laccophilus inconstans* **sp. n.** (Guinea, Ivory Coast, Ghana, Nigeria, Cameroon), *Laccophilus brancuccii* **sp. n.** (Central African Republic), *Laccophilus incomptus* **sp. n.** (Cameroon), *Laccophilus australis* **sp. n.** (Tanzania, South Africa), *Laccophilus minimus* **sp. n.** (Namibia), *Laccophilus eboris* **sp. n.** (Ivory Coast), *Laccophilus insularum* **sp. n.** (Madagascar), *Laccophilus occidentalis* **sp. n.** (Gambia, Senegal, Mali, Guinea, Sierra Leone, Ivory Coast, Ghana, Benin, Nigeria, Central African Republic, Zaire) and *Laccophilus transversovittatus* **sp. n.** (Madagascar). *Laccophilus restrictus* Sharp, 1882, is restored as good species; not junior synonym of *Laccophilus pictipennis* Sharp, 1882. New synonyms are established as follows: *Laccophilus continentalis* Gschwendtner, 1935 = *Laccophilus perplexus* Omer-Cooper, 1970, **syn. n.**, *Laccophilus taeniolatus* Régimbart, 1889 = *Laccophilus congener* Omer-Cooper, 1957, **syn. n.**, *Laccophilus adspersus* Boheman, 1848 = *Laccophilus vitshumbii* Guignot, 1959, **syn. n.** = *Laccophilus adspersus nigeriensis* Omer-Cooper, 1970, **syn. n.** = *Laccophilus adspersus sudanensis* Omer-Cooper, 1970, **syn. n.**, *Laccophilus modestus* Régimbart, 1895 = *Laccophilus espanyoli* Hernando, 1990, **syn. n.**, *Laccophilus flaveolus* Régimbart, 1906 = *Laccophilus pampinatus* Guignot, 1941, **syn. n.**, *Laccophilus trilineola* Régimbart, 1889 = *Laccophilus simulator* Omer-Cooper, 1958, **syn. n.**, *Laccophilus mediocris* Guignot, 1952 = *Laccophilus meii* Rocchi, 2000, **syn. n.**, *Laccophilus epinephes* Guignot, 1955 = *Laccophilus castaneus* Guignot, 1956, **syn. n.**, *Laccophilus saegeri* Guignot, 1958 = *Laccophilus comoensis* Pederzani & Reintjes, 2002, **syn. n.**, *Laccophilus restrictus* Sharp, 1882 = *Laccophilus evanescens* Régimbart, 1895, **syn. n.**, *Laccophilus incrassatus* Gschwendtner, 1933 = *Laccophilus virgatus* Guignot, 1953, **syn. n.**, *Laccophilus cyclopis* Sharp, 1882 = *Laccophilus shephardi* Omer-Cooper, 1965, **syn. n.**, *Laccophilus burgeoni* Gschwendtner, 1930 = *Laccophilus wittei* Guignot, 1952, **syn. n.**, *Laccophilus secundus* Régimbart, 1895 = *Laccophilus torquatus* Guignot, 1956, **syn. n.**, *Laccophilus desintegratus* Régimbart, 1895 = *Laccophilus sanguinosus* Régimbart, 1895, **syn. n.** and *Laccophilus flavopictus* Régimbart, 1889 = *Laccophilus bergeri* Guignot, 1953, **syn. n.** = *Laccophilus segmentatus* Omer-Cooper, 1957, **syn. n.** Lectotypes are designated for the following taxa: *Laccophilus productus* Régimbart, 1906, *Laccophilus ruficollis* Zimmermann, 1919, *Laccophilus sordidus* Sharp, 1882, *Laccophilus alluaudi* Régimbart, 1899, *Laccophilus pictipennis* Sharp, 1882, *Laccophilus wehnckei* Sharp, 1882, *Laccophilus continentalis* Gschwendtner, 1935, *Laccophilus simplicistriatus* Gschwendtner, 1932, *Laccophilus complicatus* Sharp, 1882, *Laccophilus rivulosus* Klug, 1833, *Laccophilus ampliatus* Régimbart, 1895, *Laccophilus pilitarsis* Régimbart, 1906, *Laccophilus adspersus* Boheman, 1848, *Laccophilus livens* Régimbart, 1895, *Laccophilus modestus* Régimbart, 1895, *Laccophilus nodieri* Régimbart, 1895, *Laccophilus flaveolus* Régimbart, 1906, *Laccophilus pallescens* Régimbart, 1903, *Laccophilus restrictus* Sharp, 1882, *Laccophilus vermiculosus* Gerstaecker, 1867, *Laccophilus mocquerysi* Régimbart, 1895, *Laccophilus bizonatus* Régimbart, 1895, *Laccophilus tschoffeni* Régimbart, 1895, *Laccophilus persimilis* Régimbart, 1895, *Laccophilus poecilus* Klug, 1834, *Laccophilus lateralis* Sharp, 1882, Laccophilus lateralis var. polygrammus Régimbart, 1903, *Laccophilus cyclopis* Sharp, 1882, *Laccophilus shephardi* Omer-Cooper, 1965, *Laccophilus conjunctus* Guignot, 1950, *Laccophilus grammicus* Sharp, 1882, *Laccophilus flavoscriptus* Régimbart, 1895, *Laccophilus flavosignatus* Régimbart, 1895, *Laccophilus brevicollis* Sharp, 1882, *Laccophilus secundus* Régimbart, *Laccophilus desintegratus* Régimbart, 1895, *Laccophilus gutticollis* Régimbart, 1895, *Laccophilus luctuosus* Sharp, 1882 and *Laccophilus inornatus* Zimmermann, 1926. *Laccophilus remex* Guignot, 1952, comprises a species complex with uncertain taxonomic delimitation; the complex includes *Laccophilus concisus* Guignot, 1953, *Laccophilus turneri* Omer-Cooper, 1957 and *Laccophilus praeteritus* Omer-Cooper, 1957, as tentative synonyms of *Laccophilus remex* Guignot, 1952.

## Introduction

The genus *Laccophilus* Leach, 1815 is by far the most species rich genus among Laccophilinae. The most recent world catalogue (Nilsson 2015) lists 263 valid species out of which a considerable number occur in Africa including Madagascar (94 species prior to this publication, 105 species after). The high species number is not surprising due to the extensive distribution on the Globe of the genus. *Laccophilus* species are found on all continents except for Antarctica. The last taxonomic work, which treated the whole genus on global level, was Sharp (1882). After that a number of works have been published but all of them have focused on a limited geographical area. The East-Palearctic, Oriental and Australian faunas containing 59 species have been revised by Brancucci (1983). Zimmerman (1970) revised the *Laccophilus* in North America, recognizing in all 27 species. The African fauna including Madagascar has been revised by Régimbart (1895) and Guignot (1959a). An additional larger work on African *Laccophilus* was published by Omer-Cooper (1965) who treated the fauna in southern Africa. Besides these, in their scope larger treatments, there are numerous taxonomic papers, giving valuable information on regional and country level. One of the most recent being Hájek and Brancucci (2015), dealing with a species-rich group of *Laccophilus* in South East Asia. A comprehensive, up-to-date revision on the whole African continent is, however, still lacking. The main aim of this work is to fill this gap. We are perfectly well aware of the fact that this work is not complete and that there are still many taxonomic questions to be solved in Africa. Anyway, the present revision, hopefully fulfils its function as a solid base for future studies.

## Material and methods

The study material, numbering almost 11000 adult specimens, comes from a number of institutions, museums and private collections. These are referred in the text by the following abbreviations:

AMGS Albany Museum, Grahamstown, South Africa (Ferdinand de Moor and Helen James)

BMNH The Natural History Museum, London, UK (Christine S. Taylor)

CAS California Academy of Sciences, San Francisco, USA

CCT Collection Clive Turner, Plymouth, UK

CFP Collection Fernando Pederzani, Ravenna, Italy

CGC Collection Gilbert Challet, Celonova, Foothill Ranch, California, USA

CGF Collection Garth Foster, Ayr, Scotland (UK)

CGW Collection Günther Wewalka, Vienna, Austria

CIR Collection Ignacio Ribera, Barcelona, Spain

CSR Collection Saverio Rocchi, Firenze, Italy

HNHM Hungarian Natural History Museum, Budapest, Hungary

IRSNB Institut Royal des Sciences Naturelles de Belgique, Brussels, Belgique (Martina Peeters and Patrick Grootaert)

MHNG Museum d’Histoire Naturelle, Geneva, Switzerland (Giulio Cuccodoro)

MNHN Museum National d’Histoire Naturelle, Paris, France (Antoine Mantilleri)

MRAC Musée Royal de l’Afrique Centrale, Tervuren, Belgique (Marc De Meyer)

MSNM Museo Civico di Storia Naturale, Milan, Italy (Fabricio Rigato)

MZBS Museo de Zoologia, Barcelona, Spain (Gloria Masó, via Ignacio Ribera)

MZH Museum Zoologicum (Finnish Museum of Natural History), Helsinki, Finland

MZLU Zoological Museum, Lund, Sweden (Roy Danielsson)

MZUL Museo di Zoologia dell’Università, La Sapienza, Roma, Italy (Maurizio Mei)

NHMB Naturhistorisches Museum, Basel, Switzerland (the late Michel Brancucci and Matthias Borer)

NHRS Naturhistoriska Riksmuseet, Stockholm, Sweden (the late Bert Wiklund)

NMNW National Museum, Windhoek, Namibia [Comment: Specimens attributed to this museum are temporarily in Berlin, ZMHB]

NMPC National Museum (Natural History), Prague, Czech Republic (Jirí Hájek)

NMW Naturhistorisches Museum, Vienna, Austria (Manfred Jäch)

OLML Oberösterreichisches Landesmuseum, Linz, Austria (Fritz Gusenleitner and Claudia Reitstätter)

RMNH Nationaal Natuurhistorische Museum (Naturalis), Leiden, the Netherlands (A. van Assen)

SAMC Iziko Museum of Cape Town, South Africa (Margie Cochrane and Dawn Larsen)

SMNS Staatliches Museum für Naturkunde, Stuttgart, Germany

TAU Tel Aviv University, Israel (Netta Dorchin and Leonid Friedman)

TMSA Transvaal Museum, Pretoria, South Africa (Ruth Müller)

USNM National Museum of Natural History, Washington D.C., USA (David G. Furth)

ZMHB Museum für Naturkunde der Humboldt-Universität, Berlin, Germany (Manfred Uhlig)

ZMSC Zoologische Staatssammlung, München, Germany (Martin Baehr)

ZMUC Zoological Museum, Copenhagen, Denmark (Alexey Solodovnikov)

Names in brackets in the list above refer to colleagues responsible for arrangements of loans.

The material studied is given for each species in a separate section, where relevant countries are arranged from west to east and north to south.

Species geographical records were provided with decimal degree latitude and longitude coordinates whenever possible (Suppl. material 1).

### Preparation technique, drawings, photographs and mapping

The study material consists both of dry, pinned specimens as well as specimens preserved in ethanol. For study of the genitalia dry specimens were treated as follows. Examined specimen was softened in hot water for some minutes. After that the apical ventrite was detached and the genitalia were released from surrounding, hardened tissue. Often the hardened tissue needed to be treated in a heater-device for about 10 minutes in 10% KOH solution. The genitalia were than washed in water baths and prepared for illustrations. Drawings were made using a Wild M 11 microscope provided with a camera lucida. The cleaned male genitalia were put in a drop of glycerine on a slide for the illustration-process. After this the genitalia and the detached apical ventrite were mounted on a card together with the specimen. Wet specimens were treated in same manner as dry specimens. If the wet specimen studied was still preserved as wet, the genitalia were placed in a microvial together with the specimen. Penis and paramere were illustrated either detached in two pieces or undetached, together in one piece, depending on what was accessible.

Illustrations of external body-parts were made using a Wild M 5 -microscope provided with a camera lucida.

Habitus photos were taken with a Canon EOS 5D Mark II DSLR camera with a supermacro MPE-65 mm f/2.8 1–5× lens and mounted on a stackshot motorized rail from Cognisys. For light the macro twin-head flash MT-24EX was used with homemade light diffusors both directly on the flash heads and as a cylinder around the specimen. Extended focus was achieved with focusstacking technique with between 6 and 20 photos taken for each specimen. The motorized Stackshot rail was controlled via the software Zerene Stacker (Version 1.04 Build T201402072140, Zerene Systems, LLC). Focus range was assessed with live view images delivered by EOS Utility (Version 2.14.10, Canon INC). All species were photographed with the MPE-65 lens set at 3:1 magnification. The PMax algorithm in Zerene Stacker under default settings was used to create an extended focus image from the original stack of photos. Postediting of images was done in Adobe Photoshop CS5 Extended (Version 12.1 x64, Adobe Systems Incorporated) where also a scale was added using a calibration file.

Species geographical records were provided with decimal degree latitude and longitude coordinates whenever possible. Named geographical units were identified on the Microsoft Encarta Premium Suite (version 2003) world map when present. Other sources for the geographical position of named units include published expedition reports, vintage atlases and google search. Records providing only general information like names of regions and countries were not provided with coordinates. Other problematic cases include redundant names without discriminating information. For each species a list of records expressed as decimal degree latitude and longitude coordinates were added to a basemap layer provided by ESRI using ArcGIS 10 and WGS 1984 projection.

The species groups used in this revision are based on the phenetic analysis presented on p. 12. Within the species groups the species are ordered on the basis of morphological similarity.

## Systematics

### Genus Laccophilus

Taxon classificationAnimaliaColeopteraDytiscidae

Leach, 1815

#### Type species

(by monotypy). *Dytiscus minutus* Linnaeus, 1758.

*Laccophilus* Leach, 1815: 84 (673 alternative page number) (original description); Aubé 1838: 415 (description, global distribution); Sharp 1882: 286, 287 (description, faunistics, species list, faunistics, discussion, species group delimitation); Kolbe 1883: 386, 401 (faunistics, discussion); Peschet 1917: 23 (discussion, key); Zimmermann 1919: 119 (description); Zimmermann 1920a: 16 (catalogue, faunistics); Bertrand 1928a: 184 (description, faunistics); Bertrand 1928c: 364 (larva description); Guignot 1937: 137, 138 (discussion, description, key to genera; type species of genus incorrectly given as *Laccophilus hyalinus* De Geer); Guignot 1946a: 116 (type species *Dytiscus minutus* Linnaeus); Guignot 1946c: 260, 261, 315 (description, key to genus and species groups, discussion); Guignot 1948: 15 (description, key to genera); Bertrand 1948: 12 (description larva, faunistics); Bertrand 1951: 114 (discussion, faunistics); Bertrand 1954: 284, 288, 289 (discussion larva, description, faunistics); Guignot 1955a: 37 (biology); Omer-Cooper 1956: 21, 23 (faunistics, biology); Omer-Cooper 1957: 8, 11, 90 (key, description); Omer-Cooper 1958b: 36 (key, subgroups, description); Guignot 1959a: 530 (description, discussion, faunistics, 11 species groups distinguished and keyed); Omer-Cooper 1962: 294, 295 (faunistics); Bertrand 1963: 402, 411, 448 (juvenile discussion); Omer-Cooper 1965: 61, 65 (description, discussion, faunistics, biology); Bertrand 1970: 18, 38 (description, larva); 1971: 252 (larva, faunistics); Bertrand and Legros 1971: 244 (faunistics, biology); Forge 1981: 501 (description, faunistics); Brancucci 1983: 251, 253 (description, key); Brancucci 1983b: 241–426 (description, faunistics, discussion, taxonomic revision Oriental, East-Palearctic and Australian species); Pederzani 1988: 107 (faunistics); Nilsson et al. 1989: 299 (list, type species by monotypy, *Dytiscus minutus* Linnaeus, 1758); Nilsson and Persson 1993: 79 (discussion, faunistics, discussion); Pederzani 1995: 43, 73 (cosmpolitan genus, key, list); Nilsson et al. 1995: 505 (faunistics); Balke et al. 1997: 295–320 (review New Guinea species, melanism, discussion); Nilsson and Roughley 1997: 4 (list); Alarie et al. 2000: 121–164 (Laccophilinae phylogeny discussion, based on larval morphology); Nilsson 2003: 76 (type species: *Dytiscus minutus* Linnaeus); Reintjes 2004: 66 (faunistics list, all continents); Bilardo and Rocchi 2006: 130, 133 (faunistics, discussion); Bilardo and Rocchi 2011: 226 (biology); Bilton 2015: 446 (biology); Nilsson 2015: 208 (catalogue, faunistics). [Comment: literature, associated with Africa are only included. Accordingly, the list is incomplete for non-African species.]

#### Diagnosis.

According to Miller and Bergsten (2014) the tribe Laccophilini, including the genus *Laccophilus*, is characterized by not visible scutellum when elytra closed, a single metatarsal claw, and prominent lobes at the anteroapical apices of the metatarsomeres. All African species of *Laccophilus* have bifid metatibial spines (Fig. 9), which separate them from the other Laccophilini genera in Africa.

#### Description.

Body parameters: Length of body 2.8–6.0 mm, width 1.5–3.4 mm. Shape somewhat variable, elongate to oval, rarely sub-cylindrical (Fig. 382). Often, posteriorly flattened, with various colour pattern (Figs 393, 401, 451, 457, 471, 489, 515, 526).

Microsculpture and reticulation of two different kinds: Simple (meshes equally large, almost uniform, no size categories of meshes distinguished) and double (meshes of two kinds; size categories distinguished). When distinctly double, body covered with large meshes which generally contain a various number (2–8) of fine (less pronounced) meshes. Commonly, lines of large meshes in part reduced and weakly developed; sometimes almost absent and only discerned as fragments/rudiments. Less commonly, lines of finer meshes are reduced and difficult to discern within larger meshes. Sometimes mesh-categories in part mixed and microsculpture appears indistinct or absent while distinct in another location of same specimen. Rarely meshes of microsculpture elongated, being comparatively long in relation to breadth. Dorsal surface of body shiny to mat. Large parts of body in ventral-aspect with very fine, simple and slightly undulate linear microsculpture, which can be reduced, in part absent. Punctures on dorsal surface of body generally sparse and concentrated to various regions. Head at eyes with fine and irregular punctures. Punctures at area of head often enlarged narrowly towards head-centre, forming a sparse, transverse row of punctures connecting ocular punctuate areas. On pronotum fine punctures often discernible, especially at pronotal margins. Elytra with fine, irregular, longitudinal rows of punctures often discernible on disc, dorsoventrally and laterally. Ventral surface largely lacking punctures. Apical ventrite, however, generally with scattered, fine punctures. Lateral, pre-apical furrow of elytra generally distinct and pubescent.

**Figures 1–10.**
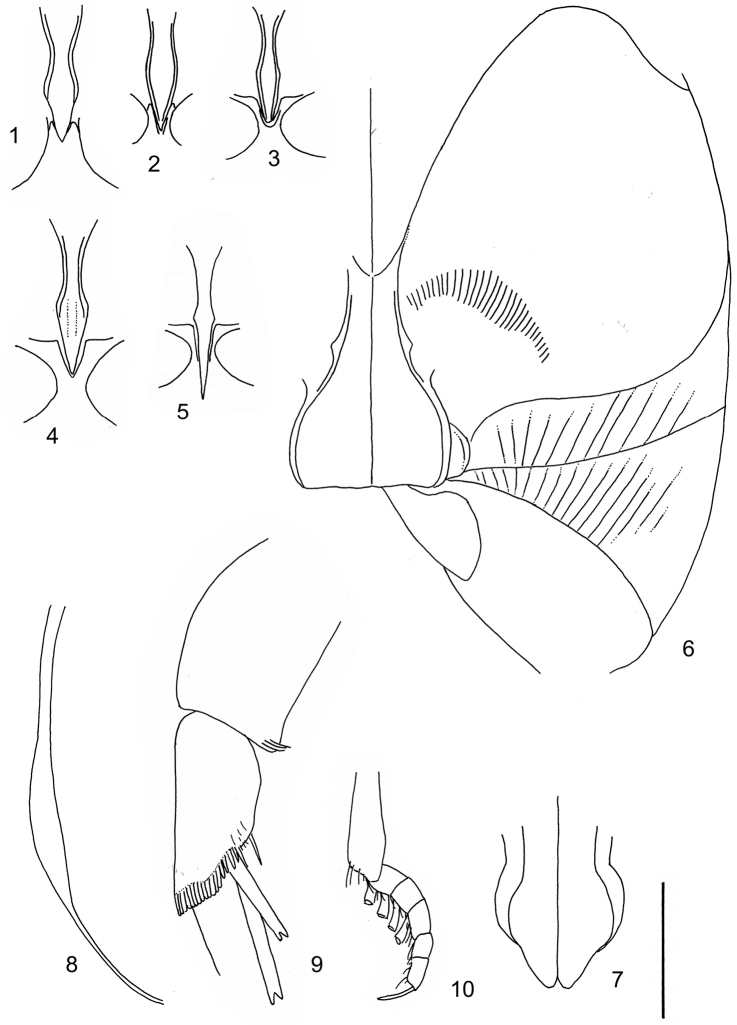
Morphological features in *Laccophilus* spp. **1–5** prosternal process of *Laccophilus* spp **6** metacoxal process, metacoxal plate with stridulation file and striated segments of abdomen in *Laccophilus hyalinus* **7** metacoxal process of *Laccophilus isamberti* **8** enlargement of epipleuron in *Laccophilus pellucidus* **9** bifid metacoxal spurs in *Laccophilus hyalinus* **10** protarsal suckers in male of *Laccophilus hyalinus*. Scale bar 0.5 mm.

Ventral aspect: Prosternal process slender, often strongly extended posteriorly and apically pointed (Figs 1–5). Metacoxal plates often provided with transverse, slightly obscure and shallow furrows, which can be rather indistinct. Stridulatory apparatus, when present, is located posteriorly on metacoxal plates, quite close to midline of body. Apparatus consists of dense ridges forming a semicircular file (Fig. 6). All African *Laccophilus* species have curved, fine striae on basal ventrites of abdomen (Fig. 6). Apical ventrite variable in shape, often modified and asymmetric, provided with a fine knob-like process on one side (Figs 110, 118). Apical ventrite with posterior edge modified with medial part posteriorly to a variable degree extended (Figs 47, 112). Some species groups lack modifications on apical ventrite (Figs 26, 43). Metacoxal process posteriorly rarely expanded (Fig. 7).

Legs: Male pro-and mesotarsus slightly enlarged and provided with suckers, length of which is variable (Fig. 10) – female lacks suckers. Metatibial spurs bifid (Fig. 9).

Sexes: Similar but males provided with pro- and mesotarsal suckers. Male apical ventrites in many species groups more strongly modified than in female; often asymmetric with one-side lateral knob on apical ventrite. Rarely female epipleuron with intraspecific, partial enlargement (Fig. 8).

#### Distribution.

Global distribution covering all continents but Antarctica. According to the world catalogue 263 species recognized (Nilsson 2015).

#### Ecology and collecting circumstances.

In Africa the genus occurs in all kinds of freshwater habitats. Often collected in quite shallow water with sparse vegetation on sandy-clay-bottom, e.g. in drinking pools for domestic animals. No comprehensive work on ecology of *Laccophilus* exists. Scattered information can be obtained by scrolling through faunistic literature, here listed. Additional sparse information on ecology is documented on many collecting labels. Experiences from Madagascar by the last author gives *Laccophilus* as one of the most ubiquitously occurring dytiscid genera. Different species have been found from sea level up to an altitude of over 2000m. *Laccophilus* inhabits many types of both lotic and lentic waters with different species and species groups more specialized. The *Laccophilus alluaudi* species group for example contains typical lotic species. The group is characteristic of small to medium-size canopy-covered rainforest streams with sandy or gravel bottoms lacking vegetation but collecting dead leaves at margins. Species from other groups like the *Laccophilus taeniolatus* group are often very abundant in red-clayish ponds visited by zebu cattle. The *Laccophilus leonensis* group can be found in vegetation-rich forest swamps and marshes or at margins of slow flowing vegetated sections of open landscape meandering rivers. When taken out of the water and put on dry land and when disturbed they can jump distances at least 20x their own body length. The behavior has not been studied in detail and could be both an anti-predatory escape behavior or used when semipermanent streams or side pools gradually dry out and the beetle can without flying move sideways or downstream to new habitats.

### *Laccophilus*: immature stages

As larval morphology is known only for six of the 13 genera of Laccophilinae (Miller et al. 2005), it is hard to give a valid diagnosis for the entire subfamily. Larvae of the genus *Laccophilus* were described in detail by Alarie et al. (2000) based on the study of seven species from Europe and the New World including *Laccophilus poecilus* Klug known also from Africa. In the same work, characters were presented for the separation of especially instar III *Laccophilus* larvae from those of the genera *Africophilus* Guignot, 1948, *Australphilus* Watts, 1978 and *Neptosternus* Sharp, 1882. Mature *Laccophilus* larvae are good swimmers with legs provided with dorsal rows of natatory setae on tibiae and tarsi. The coxae and femora are provided with rows of comb-like spinulae or pectens. The larvae have long urogomphi provided with many secondary setae. A specific feature of the instar I larva is the strong submedian constriction of the frontoclypeus that bears only two marginal spatulate setae. Miller et al. (2005) provided a larval description as well as molecular characters used to link larvae and adults of African *Philodytes umbrinus*. They discuss characters shared between *Philodytes* and known *Laccophilus* larvae as well as diagnostic differences.

Larvae of only a few Afrotropical *Laccophilus* species have been described so far, and most of the descriptions are not very detailed. As already mentioned, Alarie et al. (2000) described the instar III larva of *Laccophilus poecilus*, and all three instars of this species had previously been described in much detail by De Marzo (1976; as *Laccophilus variegatus* Dejean). As no other larvae have been reared from eggs laid in captivity, identifications remain slightly uncertain, and some of the descriptions refer only to *Laccophilus* spp. Bertrand (1928) described the larva of *Laccophilus complicatus* Sharp based on material from Madagascar, and later he provided descriptions of unidentified species collected in the Democratic Republic of the Congo, Guinea and the Ivory Coast (Bertrand 1935, 1948 & 1954). More unidentified *Laccophilus* larvae were reported by Bertrand (1966a & 1968) from various wetlands in the Democratic Republic of the Congo, Kenya, Rwanda, Uganda, and Tanzania. Based on differences in the pigmentation of the head capsule and association with adults, Bertrand (1966b, 1969) gave records of supposed larvae of *Laccophilus adspersus* Boheman, *Laccophilus cyclopis* Sharp and *Laccophilus lineatus* Aubé from various ponds and streams in Lesotho, South Africa and Zimbabwe. Larvae of *Laccophilus adspersus* were also reported from Kenya (Bertrand 1963). These and a few more records of *Laccophilus* larvae from Africa were reviewed by Bertrand (1972).

The pupal stage has been described briefly for selected *Laccophilus* species by Wilson (1923) and Bertrand (1928). A more detailed description of the pupa of the Nearctic *Laccophilus fasciatus rufus* was provided by Sizer et al. (1998). No pupae of African origin have been studied so far.

### Species groups of African *Laccophilus*

The present revision of the genus *Laccophilus* focuses on the species occurring in the African mainland and Madagascar with its neighbour islands. Accordingly, from this standpoint no thorough analysis of the phylogeny of the genus is therefore possible. About 60% of the recognized species-bulk is distributed outside Africa on various other continents. Plans for future, however, include a phylogenetic survey of the whole genus on a global basis in which both morphological characters and molecular data will be considered. The forthcoming study will also focus on groups of species recognized in the genus *Laccophilus*.

Despite problems in understanding *Laccophilus* systematics at a global level a division of the genus in different species groups only for Africa including Madagascar is justified and can be motivated by practical reasons. Management of a total 105 species can be quite demanding without division in practical groups. The survey here undertaken does not count on detection of synapomorphies for delimitation of monophyletic groups, but is based on simple similarity (presence of shared characters). Below, recognized and examined characters are briefly described and discussed. Three additional Laccophilinae genera are included in the survey: *Philodytes* J. Balfour-Browne, 1939, *Neptosternus* Sharp, 1882, and *Philaccolus* Guignot, 1937.

The recognized groups of species in African *Laccophilus*, introduced in this revision do not coincide well with those presented by Guignot (1959a). Within species groups recognized the species are listed in accordance with morphological similarity between the species.

In Table 1 the possession of the discussed characters in the species groups and three reference genera is presented.

**Table 1.** Species groups and outgroups scored for characters 1-10 discussed in the text.

Species group/character numbers	1	2	3	4	5	6	7	8	9	10
sp.gr. 1 (morondavensis)	1	2	1	2	1	2	2	2	2	1
sp.gr. 2 (ruficollis)	1	1	2	2	1	2	2	2	2	1
sp.gr. 3 (hyalinus)	1	1	2	1/2	1	2	2	2	2	1
sp.gr. 4 (alluaudi)	1	1	2	2	1	2	2	2	2	1
sp.gr. 5 (isamberti)	1	1	2	2	1	2	1	2	2	1
sp.gr. 6 (pictipennis)	1	1	2	2	1	2	2	1	2	1
sp.gr. 7 (taeniolatus)	1	1	2	2	1	1	2	1	2	2
sp.gr. 8 (immundus)	1	1	2	2	1	2	2	1	2	2
sp.gr. 9 (pellucidus)	1	1	2	2	1	2	2	1	2	2
sp.gr. 10 (adspersus)	1	1	2	2	1	1	2	1	1	2
sp.gr. 11 (deceptor)	1	1	2	2	1	1	2	1	1	2
sp.gr. 12 (poecilius)	1	1	2	2	1	2	2	1	1	2
sp.gr. 13 (lineatus)	1	1	2	2	1	1	2	1	1	2
sp.gr. 14 (desintegratus)	1	1	1	2	1	1	2	1	1	2
sp.gr. 15 (luctuosus)	1	1	2	2	1	1	2	1	1	2
sp.gr. 16 (leonensis)	1	1	2	1	1	1	2	1	1	2
sp.gr. 17 (laeticulus)	1	1	2	1	1	1	2	1	1	2
Philodytes	2	1	2	2	1	1	2	1	2	2
Neptosternus	2	1	1?	2	2	2	2	2	2	1
Philaccolus	2	1	1?	1	1	2	2	2	2	2

1. Apices of metatibial spurs bifid (Fig. 9) (1) / Not bifid (2).
  The bifid spurs have total coverage in *Laccophilus*, all species exhibiting the feature. In all other Laccophilinae genera the corresponding spines are pointed and most probably bifid spines are a derived character, which indicates the genus *Laccophilus* is monophyletic. However among species outside Africa Balke et al (1997) have reported *Laccophilus* species with pointed metatibial spurs, a likely reversal.
2. Body in posterior half dorso-ventrally somewhat flattened (Fig. 418) (1) / Body subcylindrical (Figs 378–384) (2).
  The dorso-ventrally flattened body is widely distributed in Laccophilinae and also present in all but one species group of *Laccophilus*. Accordingly, the feature could represent a plesiomorphy while the subcylidrical body-shape is a synapomorphy, characteristic of the *Laccophilus morondavensis* species group.
3. Microsculpture of dorsal body surface simple (1) / Double, mixed (2).
  Simple microsculpture indicates reticulation on body, where the meshes are similar in size and shape. No size-categories can be distinguished between meshes, neither are there differences in their qualitative feature – the meshes form a smooth coverage on body surface. Double microsculpture means that there are two kinds of reticulation mixed on same location; large meshes and small meshes. The large meshes are generally more strongly impressed in the body surface than the small ones. A large mesh encloses often a number of small meshes, which can vary in number between 2 and 8. Reduction of meshes occurs often in regard of large meshes but can also be the case for small meshes. When reduced, the meshes are either in part or totally lacking. In such cases fragments of meshes can be detected, mixed with complete meshes of different kind. Sometimes mesh-categories appear variable so that division in size-classes is impossible. Rarely the meshes are deformed and their shape is elongate. Two species groups of African *Laccophilus* have simple microsculpture (i.e. *Laccophilus morondavensis* and *Laccophilus desintegratus* species groups), while 15 species groups seem to have double, by the above definition.
4. Metacoxal plates have a stridulatory file (Fig. 6) (1) / Stridulatory file absent (2).
  The stridulatory file is a semicircular device which is formed by densely located ridges on the metacoxal plate. The function of it has not been thoroughly studied and possible sound has not been heard, nor recorded. In African *Laccophilus* both sexes seem to have the device when present. Presumable use of it can be related to intraspecific communication but it may also be used e.g. in defence against predators. Three species groups of African *Laccophilus* exhibit this feature. In one group (*Laccophilus hyalinus* species group) with a modest number of species, only two species have it. In the two remaining species groups (*Laccophilus leonensis* and *Laccophilus laeticulus* species groups) all representatives are provided with it. In the latter species group the file, however, is very weakly developed and may be rudimentary and out of function. A similar stridulatory device is also present in other Laccophilinae genera, e.g. in genus *Philaccolus* while lacking in e.g. *Philodytes*.
5. Abdominal ventrites provided with sparse, somewhat curved striae (Fig. 6) (1)/ Striae absent (2).
  All African *Laccophilus* species have a number of sparse, curved striae on abdominal ventrites. This character seems to be widely distributed in Laccophilinae as it may be recognized at least in *Philaccolus* and *Philodytes* and in a reduced state in *Neptosternus*.
6. Prosternal process slender, posteriorly distinctly extended, apically pointed (Fig. 5) (1) / Prosternal process shorter; comparatively broad, posteriorly not strongly extended (Figs 1–4) (2).
  At least 10 species groups of *Laccophilus* have slender, extended prosternal process while in 7 species groups the process is shorter and broader, which seem to be the case in Laccophilinae outside *Laccophilus* as well. The slender process may be a synapomorphy of a supposed clade containing the respective species groups.
7. Metacoxal process posteriorly expanded and modified (Fig. 7) (1) / Metacoxal process posteriorly not expanded; ends abruptly (Fig. 6) (2).
  In *Laccophilus* one species placed in its own species group (*Laccophilus isamberti* species group) exhibits this enigmatic and unique feature. It definitely represents the derived state and future studies will reveal if the species deserves a status of a separate genus within Laccophilinae.
8. Posterior edge of apical ventrite modified, forming an undulate structure, with medial part distinctly extended backwards (Fig. 69) (1) / Posterior edge of apical ventrite not modified; outline of ventrite smoothly curved (Fig. 25) (2).
  In African *Laccophilus* 13 species groups out of 17, exhibit the modified apical ventrite. Besides *Laccophilus*, *Philodytes* has a similar modified apical ventrite while at least *Philaccolus* and *Neptosternus* lack it.
9. Male apical ventrite strongly asymmetric, when provided with a distinct, small knob or process on one side of the midline of ventrite (Fig. 69) (1) / No asymmetric knob or process on male apical ventrite (Fig. 47)(2).
  In total 9 recognized species groups of *Laccophilus* in Africa, exhibit this, most probably derived character. It may turn out to be a good synapomorphy for them.
10. Penis apex narrow, often curved and exhibits only slight modifications in anatomical shape (Figs 212, 237) (1) / Penis generally strongly modified, exhibiting various anatomical details (2).
  In all six species groups of *Laccophilus* here distinguished, have a slender to rather slender, often quite evenly curved penis, lacking considerable modifications.

### Key to species groups of African *Laccophilus*

To be considered slightly tentative and mostly only applicable for male specimens.

**Table:** 

1	Male apical ventrite symmetric, lacking lateral process/knob (Figs 12, 27, 51)	**2**
–	Male apical ventrite strongly asymmetric, provided with a minute, lateral process (located to left on ventrite, when viewed from below) (Figs 69, 96, 207)	**10**
2	Large species (body length 5.3–6.0 mm); body dorsoventrally flattened (Fig. 413); penis apex strongly modified (Fig. 259)	**group 9 (*Laccophilus pellucidus*)** (p. 89)
–	Smaller species with flattened or subcylindrical body (body length 3.2–5.8 mm); penis shape different, variable	**3**
3	Body subcylindrical (Fig. 382); generally large species (body length 4.1–5.7 mm), except one species (3.1–3.4 mm); body microsculpture simple, of one kind	**group 1 (*Laccophilus morondavensis*)** (p. 17)
–	Body dorsoventrally flattened; small to large species (body length 3.2–5.8 mm); body microsculpture (dorsal aspect) double (can be reduced, rarely distinctly so)	**4**
4	Metacoxal process posteriorly expanded (Fig. 7)	**group 5 (*Laccophilus isamberti*)** (p. 47)
–	Metacoxal process posteriorly truncate, not expanded (Fig. 6)	**5**
5	Elytra provided with longitudinal, dark markings (Fig. 391) (incl. *Laccophilus rivulosus* (Fig. 416))	**group 4 (*Laccophilus alluaudi*)** (p. 36)
–	Elytral markings variable; never forming distinct, dark, longitudinal markings (excl. *Laccophilus rivulosus* (Fig. 416))	**6**
6	Posterior margin of male apical ventrite modified, undulate with middle part posteriorly extended (Fig. 55)	**7**
–	Posterior margin of male apical ventrite not modified; non-undulate, posterior margin curved and medially not posteriorly extended (Figs 22, 24)	**9**
7	Elytra pale ferrugineous to pale brownish with dense, dark ferrugineous to blackish irrorations/ undulations (Figs 400, 411); one paramere (upper in illustrations) apically enlarged (Fig. 255)	**group 7 (*Laccophilus taeniolatus*)** (p. 52)
–	Elytral colour pattern different; either uniformly, dark ferrugineous (Fig. 417) or with extensive patches (Fig. 398); parameres different, apically not enlarged (Figs 248, 258)	**8**
8	Elytra with distinct colour pattern (with extensive patches) (Fig. 398); penis evenly curved, narrows gradually towards apex (Fig. 247)	**group 6 (*Laccophilus pictipennis*)** (p. 49)
–	Elytra ferrugineous to dark ferrugineous, lacking distinct paler areas (Fig. 417); penis with peculiar curvature and apical expansion (Fig. 258)	**group 8 (*Laccophilus immundus*)** (p. 87)
9	Small species (body length 3.2–3.4 mm); elytra dark ferrugineous with distinct, transverse, pale ferrugineous markings (Fig. 385)	**group 2 (*Laccophilus ruficollis*)** (p. 26)
–	Larger species (body length 4.0–5.3 mm); elytra generally pale brownish to ferrugineous, often with vague, pale ferrugineous to pale brownish areas, or colour pattern absent (Figs 386, 389)	**group 3 (*Laccophilus hyalinus*)** (p. 28)
10	Metacoxal plates provided with a semicircular stridulation apparatus (Fig. 6)	**11**
–	Metacoxal plates lack stridulation apparatus	**12**
11	Penis in lateral view evenly curved; almost evenly broad from middle to apex; inner outline of penis close to apex uneven (provided with minute ridges) (Figs 362, 367)	**group 16 (*Laccophilus leonensis*)** (p. 237)
–	Penis in lateral view slightly angled; narrows distinctly from middle to apex; inner outline of penis close to apex smooth (lacks minute ridges) (Figs 373, 377)	**group 17 (*Laccophilus laeticulus*)** (p. 256)
12	Elytral colour pattern distinct, consists of dark longitudinal markings, which may be undulate and connected with neighbour-markings; sometimes markings merged into extensive dark areas (Figs 472, 476, 480) or reduced (Figs 478, 483)	**13**
–	Elytral colour pattern different (note that there are species with extensive dark elytra), not provided with distinct longitudinal, dark markings; often patchy (Figs 457, 467) or rather pale with dense irrorations/undulations (Figs 419, 437)	**15**
13	Body (dorsal aspect) microsculpture simple, of one kind (no fragments of large meshes discernible); penis apex broad, truncate (Fig. 358)	**group 14 (*Laccophilus desintegratus*)** (p. 231)
–	Body (dorsal aspect) microsculpture double, consists of two kinds of microsculpture, mixed: often larger meshes of microsculpture reduced in part (fragments of large meshes generally discernible); shape of penis different	**14**
14	Penis robust, curved, apically provided with a distinct extension and inner outline provided with distinct ridges (Fig. 359); elytra extensively dark with transverse, basal, pale marking which can be reduced to separate spots (Figs 503–505); small species (body length 2.9–3.6 mm)	**group 15 (*Laccophilus luctuosus*)** (p. 234)
–	Penis slender to robust, apex forming a distinct hook/angled enlargement (Figs 327, 335) or penis curved, sometimes also twisted (Figs 345, 350); elytral colour pattern different (variable); small to large species (body length 2.9–5.1 mm)	**group 13 (*Laccophilus lineatus*)** (p. 178)
15	Penis (lateral aspect) externally close to base with a deep incision (Fig. 322)	**group 12 (*Laccophilus poecilus*)** (p. 176)
–	Penis (lateral aspect) externally close to base without deep incision	**16**
16	Elytra pale ferrugineous to pale brownish, generally with extensive, often delicate, dark irrorations (Figs 419, 425), or almost unicoloured, brownish or blackish (Figs 436, 445); penis rather slender, curved or angled and provided with a distinct apex (Figs 261, 270, 289)	**group 10 (*Laccophilus adspersus*)** (p. 95)
–	Elytral colour pattern different; consists of pale patches arranged in variable, transverse series (Figs 453 461); penis different, variable in shape (e.g. Figs 303, 307, 313, 318, 320)	**group 11 (*Laccophilus deceptor*)** (p. 159)

### Species group 1 (*Laccophilus morondavensis* group)

**Diagnosis.** Large species with length of body 4.1–5.7 mm, width 1.9–3.0 mm, except one small species, *Laccophilus tavetensis*, with length 3.1–3.4 mm and width 1.6–1.7 mm.

Shape of body subcylindrical, dorsoventrally not flattened (Fig. 382). Body dorsally, with distinct colour pattern, which is formed by rather extensive, often longitudinal, dark/pale patches especially on elytra (Fig. 381). One species with body, dorsal aspect, lacking distinct colour pattern; ferrugineous to dark ferrugineous (Fig. 384). Body microsculpture simple, of one kind.

Prosternal process moderately broad, posteriorly not distinctly extended, apically pointed. Apical ventrite modified; posteriorly on both side of midline more or less excavated and post-medially often extended to a narrow enlargement (Fig. 15). Apical ventrite lacks asymmetrical, small knob. No stridulatory apparatus on metacoxal plates. Metacoxal process not extended posteriorly (Fig. 6).

Paramere simple, elongate, apically not distinctly enlarged or modified (Fig. 218). Apical half of penis slender to quite slender, almost straight to distinctly curved, apically not distinctly modified (Fig. 214). One species with tip of penis slightly enlarged (Figs 222–223).

**Species composition and distribution.** Seven African species are included to the group (see the identification key). None of them occurs outside Africa south of Sahara or Madagascar.

**Note.** Male of *Laccophilus mirabilis* is unknown. Large body (length 5.1–5.7 mm). Elytral colour pattern consisting of separate, longitudinal dark markings (Fig. 383). Only known from Madagascar.

#### Key to species (males)

**Table:** 

1	Small species, length of body less than 3.4 mm	***Laccophilus tavetensis*** (p. 18)
–	Larger species, length of body between 4.1–5.7 mm	**2**
2	Body, dorsal aspect, lacks distinct colour pattern (Fig. 384); penis tip with slight enlargement (Figs 222–223)	***Laccophilus ferrugo*** (p. 25)
–	Body, dorsal aspect, with distinct colour pattern (Fig. 381); penis tip not enlarged	**3**
3	Penis, lateral aspect, comparatively broad; apex of penis distinctly curved backwards (Fig. 216)	***Laccophilus morondavensis*** (p. 22)
–	Penis, lateral aspect, narrower; apex of penis not curved backwards	**4**
4	Body broad, oval, large (length 4.9–5.2 mm) (Fig. 379)	***Laccophilus grossus*** (p. 19)
–	Body more elongate, smaller (length 4.1–4.9 mm) (Fig. 380)	**5**
5	Body elongate, slender; pale areas on elytra open with no closed cells or one inner cell formed by dark, narrow, longitudinal marking (Fig. 382)	***Laccophilus productus*** (p. 23)
–	Body slightly broader; pale areas on elytra with two closed cells, formed by dark, narrow, longitudinal markings (Fig. 380)	***Laccophilus rocchii*** (p. 20)

#### Laccophilus tavetensis

Taxon classificationAnimaliaColeopteraDytiscidae

Guignot, 1941

**Figures 11–21.**
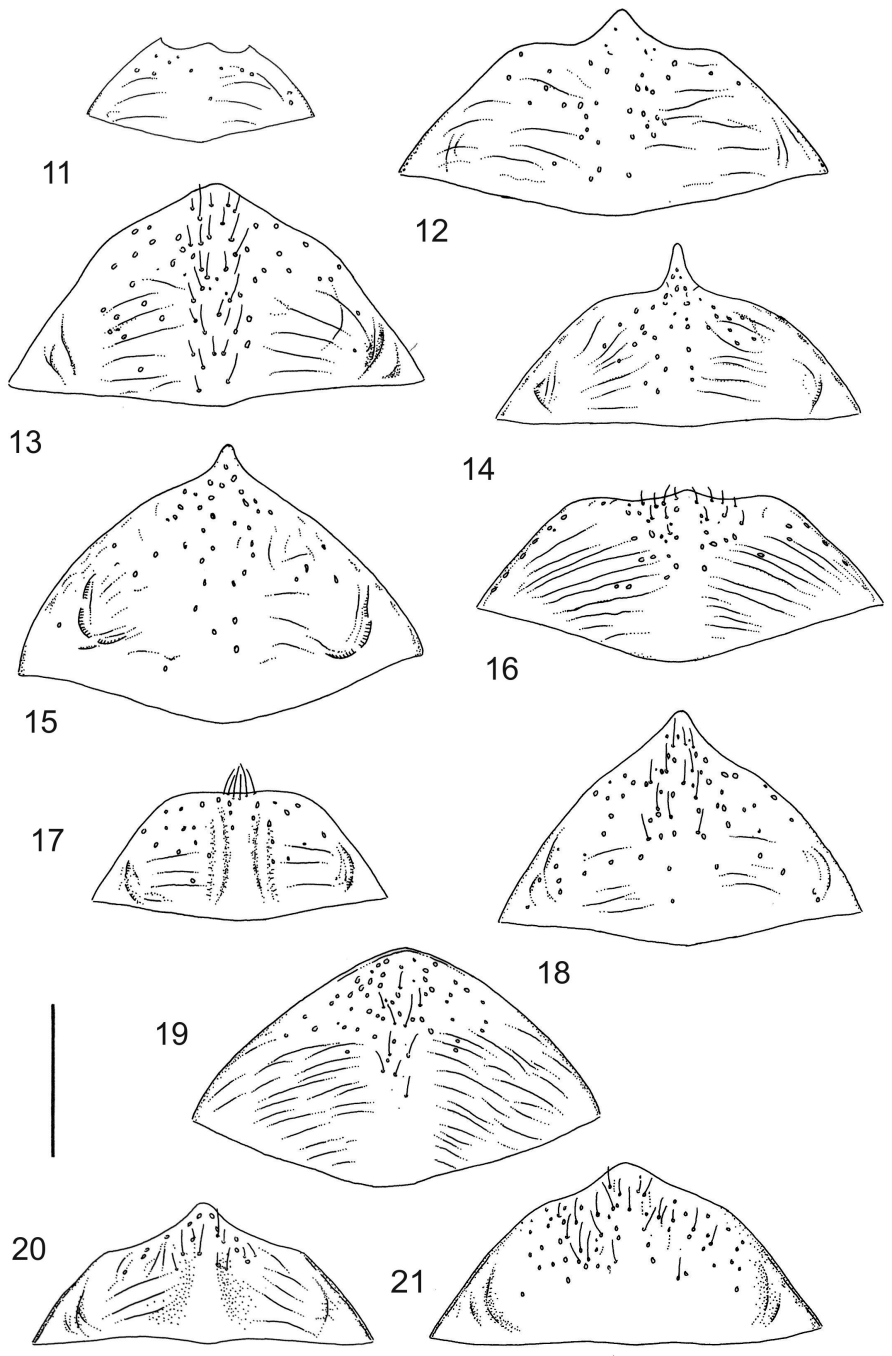
Apical ventrites **11** *Laccophilus tavetensis* male **12** *Laccophilus grossus* male, and **13** female **14** *Laccophilus rocchii* male, and **15** female **16** *Laccophilus morondavensis* male **17** *Laccophilus productus* male, and **18** female **19** *Laccophilus mirabilis* female **20** *Laccophilus ferrugo* male, and **21** female. Scale bar 0.5 mm.

Figs 11 , 209–211 , 378 , 527

Laccophilus tavetensis Guignot 1941: 36 (original description, discussion, faunistics); Guignot 1946c: 283, 285, 313 (redescription, faunistics); Guignot 1959a: 585, 587 (redescription, faunistics); Nilsson 2001: 251 (catalogue, faunistics); Nilsson 2015: 218 (catalogue, faunistics).

##### Type locality.

Kenya: Taveta.

##### Type material studied

(1 ex.). Holotype: male: “Afrique Orient. Anglaise Taveta Alluaud & Jeannel mars 1912 – 750 m St. 65 / male symbol / Type / Det. Dr. Guignot *Laccophilus tavetensis* Guign. Type” (MNHN).

##### Additional material studied

(11 exs.). **Sudan**: “Prov. N Darfur El Geneina / ad lucem Ibrahim M. Abuzinid 20.8. 1979” (1 ex. CGW). – **Kenya**: “S, Voi 11. 1997 leg. Snizek” (4 exs. CFP, 4 exs. CSR); “Kenya eastern Sosoma ca. 200 km E of Thika 27.11. 2011, light trap” (1 ex. NMPC). – **Botswana**: “Chobe NP Savuti-Camp 18°33'55"S-24°03'53"E, 11.3. 1993 lux leg. Uhlig” (1 ex. ZMHB; habitus in Fig. 378).

##### Diagnosis.

A deviate species, separated from the other species in this species group by having small body size in combination with peculiar shape of penis; somewhat sinuate and distinctly enlarged posterior to narrow apex. Note also differently shaped male apical ventrite in comparison with other species in the species group (Fig. 11).

##### Description.

Body length 3.1–3.4 mm, width 1.6–1.7 mm. Pale ferrugineous, dorsal colour pattern ferrugineous and sometimes vague and slightly variable (Fig. 378).

Head: Pale ferrugineous, no colour pattern. Submat, with fine, dense microsculpture. Reticulation simple; only with small, uniform meshes. Impunctate, except at eyes; with fine, irregularly located punctures.

Pronotum: Pale ferrugineous, no distinct colour pattern. Submat, with fine, dense microsculpture. Reticulation simple; only with small, uniform meshes. Impunctate, at margins with fine to very fine, somewhat irregular punctures. Mediobasally punctures absent.

Elytra: Pale ferrugineous, sometimes with vague, ferrugineous, longitudinal markings (Fig. 378). Elytral colour pattern sometimes rather indistinct. Submat, with fine, dense microsculpture. Reticulation simple; only with small, uniform meshes. Fine, somewhat irregular punctures form a discal row. Dorsolateral and lateral rows of punctures indistinct; indicated by scattered, fine punctures. Laterally with a quite long, sparsely pubescent, pre-apical furrow.

Ventral aspect: Abdomen dark ferrugineous to ferrugineous, metathorax and –coxal plates ferrugineous, and prothorax pale ferrugineous. Submat, finely microsculptured. Abdomen with fine, curved striae. Impunctate, except a few fine punctures on apical ventrite; symmetric (Fig. 11). Prosternal process quite narrow, apex somewhat enlarged, short, apically pointed (arrow-shaped). Transverse shallow furrows reduced; 2–3 indistinct, reduced furrows discernible.

Legs: Pro- and mesotarsus slightly enlarged, extended, provided with suckers.

Male genitalia: Penis in dorsal aspect clearly sinuate with narrow tip; in lateral aspect almost evenly curved Figs 209–211.

Female: Unknown.

##### Distribution.

Sudan, Kenya, Botswana (Fig. 527).

##### Collecting circumstances.

In Botswana collected with light.

#### Laccophilus grossus sp. n.

Taxon classificationAnimaliaColeopteraDytiscidae

http://zoobank.org/9D64A9B0-3AA9-4A2D-9276-604D813312B8

Figs 12–13 , 212–213 , 379 , 527

##### Type locality.

Namibia: Damaraland, Oshikango (15.55E, 17.25S).

##### Type material

(5 exs.). Holotype, male: “South Africa Damaraland Oshikango, v. 1948 15.55E, 17.25S, C. Koch / B. Malkin Coll. BMNH (E) 1956–234” (BMNH). – Paratypes: Same data as holotype (1 ex. MZH; habitus in Fig. 379); “Angola Rocadas R. Cunene 19–22.2. 1972/at light“ (1 ex. CFP); “Angola Rocadas 30.3. 1972” (1 ex. CFP); “Namibia 23.2. 1994 17°26'S/14°21'E, Kunene, Ruacana Dorp, lux, leg. M. Uhlig” (1 ex. ZMHB).

##### Diagnosis.

*Laccophilus grossus* belongs to a group of species, characterized by large body-size, by uniform microsculpture, with one kind of meshes (small) and by slender, slightly sinuate penis. The new species is probably closest related to *Laccophilus rocchii*, another so far undescribed species. The two species are distinguished by difference in body size, by deviating dorsal, colour pattern of body and by details in shape of penis apex (curved in different directions).

##### Description.

Body: Length 4.9–5.2 mm, width 2.7–2.8 mm. Dorsal colour pattern exhibits only slight variation (Fig. 379).

Head: Pale ferrugineous to ferrugineous to brownish; posteriorly at pronotum slightly darker than anteriorly; however, change of colour gradual and no colour pattern formed. Submat, entire head finely microsculptured; meshes small and only of one kind. Impunctate, except at eyes, with some fine, irregular punctures. Anteriorly, close to edge of head with a few transverse impressions formed by elongated punctures.

Pronotum: Pale ferrugineous to ferrugineous, medially broadly, distinctly darker; basal area blackish. Submat, finely and densely microsculptured. Meshes of microsculpture small, uniform and of one kind only. Impunctate, except at margins, finely and somewhat irregularly punctate. Broad area basally in middle lacking punctures.

Elytra: Pale ferrugineous, with blackish to dark ferrugineous, slightly variable marking (Fig. 379). Submat, with fine, uniform, evenly distributed microsculpture. Meshes of microsculpture quite small, of one kind. Fine, irregular punctures form a discal row of punctures, which spread out and disappears posteriorly. Scattered, fine punctures indicate presence of a vague, dorsolateral and lateral row of punctures. Pre-apical, lateral row of punctures comparatively long, forms a, in part, distinct furrow with some setae.

Ventral aspect: Blackish to dark ferrugineous; no distinct colour pattern formed. Submat, finely to very finely microsculptured. Abdominal ventrites with dense, curved striae. Metacoxal plates with some transverse furrows, which posteriorly fade away. Apical ventrite lacks asymmetric knob/process (Fig. 12). Prosternal process rather slender, apex moderately, posteriorly extended, apically pointed. Almost impunctate, apical ventrite with some scattered punctures.

Legs: Pale ferrugineous, hindlegs slightly darker, ferrugineous to brownish. Pro- and mesotarsus slightly enlarged, with fine suckers.

Male genitalia: Apical half of penis slightly sinuate and when viewed from above; tip of penis slightly curved right (Figs 212–213).

Female: Pro- and mesotarsus rather slender. Apical ventrite as in Fig. 13.

##### Etymology.

The species name *grossus* is a Latin adjective meaning “big”. It here associates with the body size of the new species.

##### Distribution.

Angola, Namibia (Fig. 527).

##### Collecting circumstances.

Almost unknown. In Angola collected at light.

#### Laccophilus rocchii sp. n.

Taxon classificationAnimaliaColeopteraDytiscidae

http://zoobank.org/54EC33EF-CC49-4674-B2F2-110CF108F698

Figs 14–15 , 214–215 , 380 , 527

##### Type locality.

Mozambique: Manica Province, 60 km W Chitobe.

##### Type material

(15 exs.). Holotype: male: “Mozambique Manica Province 60 km W Chitobe, 16.12. 2005 P. Schüle leg.” (SMNS). – Paratypes: “Tanzania Dodoma Pr. 40 km N Dodoma 14–16.12. 2006, 1100 m A. Kudrna Jr. lgt.” (1 ex. CFP); “Botswana: Chobe Dist., Savute Drift Camp site, 18°34'S, 24°04'E, 29. Dec. 1988 R.D. Ward / Robert D. Ward Collection / *Laccophilus productus* Rég. det. S. Rocchi 92” (1 ex. CSR; habitus photogr. Fig. 380); Similar label data as holotype (6 exs. SMNS, 1 ex. MZH); “Mocambique Prov. Inhambane 15 km SE Save, 18–21.12. 2005, A. Kurdna Jr. lgt.” (3 exs. CFP, 1 ex. MZH); “Namibia Exp. ZMB 1992 East Caprivi: Katima Mulilo, lux, 17°29'S/24°17'E, 3–8.3. 1992 leg. M. Uhlig” (1 ex. ZMHB).

##### Diagnosis.

See diagnosis of *Laccophilus grossus* (p. 19).

##### Description.

Body: Length 4.3–4.9 mm, width 2.3–2.6 mm. Body dorsally pale ferrugineous, with quite distinct and uniform blackish ferrugineous to dark ferrugineous marking (Fig. 380).

Head: Pale ferrugineous. Submat, finely microsculptured; reticulation simple. Meshes small, of same size and shape. Impunctate, except at eyes where head is provided with fine, somewhat irregularly distributed punctures. Anteriorly, close to frontal edge with some punctures, forming slightly irregular transverse impression.

Pronotum: Pale ferrugineous, basally in middle with distinct blackish ferrugineous spot. Submat, finely microsculptured; reticulation simple. Meshes small, of same size and shape. Impunctate, except along margins, with irregular, fine punctures, however, punctures lacking basally in middle.

Elytra: Pale ferrugineous, with quite distinct, quite uniform blackish ferrugineous to dark ferrugineous marking (Fig. 380). Submat, finely microsculptured; reticulation simple, of one size-category. Meshes small, size and appearance uniform. Discal and dorsolateral row of punctures consist of irregular, fine punctures. Rows are diffuse and mixed posterior to middle of elytra. Lateral row indicated by some scattered, fine punctures. Preapical, lateral row of punctures located in a distinct furrow provided with some hairs.

Ventral aspect: Pale ferrugineous to ferrugineous, no distinct colour pattern formed. Submat, very finely microsculptured, except abdomen basally, rather shiny, microsculpture indistinct. Apical ventrite of male (Fig. 14). Ventrites with fine, curved, and quite dense striae. Metacoxal plates with shallow, transverse furrows, which posteriorly, gradually become weaker. Prosternal process rather slender, posteriorly slightly extended, apex pointed.

Legs: Pro- and mesotarsus somewhat enlarged, with suckers.

Male genitalia: Apical half of penis in dorsal aspect only slightly sinuate, almost straight; extreme tip slightly curved to left (Figs 214–215).

Female: Pro- and mesotarsus slender, not enlarged. Apical ventrite as in Fig. 15.

##### Etymology.

The name is a noun in its genitive form based on the name of Mr. Saverio Rocchi, Florence, Italy, who kindly provided us with a part of the type material of the new species besides various other interesting materials.

##### Distribution.

Tanzania, Namibia, Botswana, Mozambique (Fig. 527).

##### Collecting circumstances.

In Namibia collected at light.

#### Laccophilus morondavensis

Taxon classificationAnimaliaColeopteraDytiscidae

Guignot, 1957

Figs 16 , 216–218 , 381 , 527

Laccophilus morondavensis Guignot 1957b: 72 (original description, faunistics); Rocchi 1991: 86 (faunistics, list); Nilsson 2001: 247 (catalogue, faunistics); Nilsson 2015: 214 (catalogue, faunistics).

##### Type locality.

Madagascar: Foret sud de Befasy.

##### Type material studied

(1 ex.). Holotype: male: “Morondava foret sud de Befasy I-56 R.P. / Institut Scientifique Madagascar / F. Guignot det., 1956 *Laccophilus morondavensis* sp. n. Type, male symbol” (MNHN; habitus in Fig. 381).

##### Additional material studied

(1 ex.): **Madagascar**: “W Madag. 60 km NE of Morondava, Foret de Kirindi, 30 m Bednarik leg. 28.1.1996 / *Laccophilus morondavensis* Guignot 1957 Jiri Hájek det. 2006” (1 ex. NMPC).

##### Diagnosis.

*Laccophilus morondavensis* is characterized by its distinct, elytral colour pattern and by peculiarly shaped penis apex. The species resembles externally most of *Laccophilus productus* but body is somewhat larger and broader. Additionally, pronotum is extensively dark while in *Laccophilus productus* almost entirely pale ferrugineous. Tip of penis is slightly upwards curved in *Laccophilus morondavensis* while it is almost straight in *Laccophilus productus*.

##### Description.

Body length 5.3 mm, width 2.9 mm. Dorsal, aspect of body with rather distinct colour pattern (Fig. 381).

Head: Pale ferrugineous. Posteriorly, head becomes gradually slightly darker but lacks distinct colour pattern. Rather shiny, although finely and densely microsculptured; reticulation simple, of one kind. Impunctate, except at eyes, with fine, slightly irregular punctures. Medially, areas with punctures extend slightly towards centre of head.

Pronotum: Blackish to ferrugineous, laterally pale ferrugineous. Colour change gradual; colour pattern vague. Rather shiny, although finely microsculptured. Reticulation of one kind; consists of small meshes. Entire disc with fine, sparse punctures. At margins, except mediobasally, with slightly irregular, coarse punctures.

Elytra: Pale ferrugineous, with blackish to dark ferrugineous markings (Fig. 377). Rather shiny, although finely microsculptured. Reticulation dense, of one kind; meshes moderately sized. Discal row of punctures consists of fine to very fine, scattered, punctures. Dorsolateral and lateral row of punctures as discal row but sparser and more irregular. Laterally, elytra with a rather shallow pre-apical, finely pubescent and quite extensive furrow.

Ventral aspect: Blackish ferrugineous to dark ferrugineous, prothorax paler; pale ferrugineous to ferrugineous. Rather shiny, although finely microsculptured. Abdominal reticulation reduced, in part absent. Abdomen, with fine, curved striae. Metacoxal plates with reduced, transverse furrows, which are only discernible in anterior half. Almost impunctate. Apical ventrite, with punctures, symmetric, lacks lateral knob (Fig. 16). Prosternal process rather slender, apex arrow-shaped, quite short, pointed.

Legs: Pro- and mesotarsus rather slender, somewhat extended, with suckers.

Male genitalia: Penis both in lateral and dorsal aspect broader than related species; apical tip curved upwards (Figs 216–218).

Female: Unknown.

##### Distribution.

Madagascar (Fig. 527).

##### Collecting circumstances.

Unknown.

#### Laccophilus productus

Taxon classificationAnimaliaColeopteraDytiscidae

Régimbart, 1906

Figs 17–18 , 219–221 , 382 , 527

Laccophilus productus Régimbart 1906: 249 (original description, faunistics); Zimmermann 1920a: 25 (catalogue); Peschet 1921: 6 (discussion, description, faunistics); Zimmermann 1926: 23 (faunistics); Guignot 1946c: 284, 313 (description, faunistics); Guignot 1957b: 73 (discussion, faunistics); Guignot 1959a: 585, 586 (redescription, faunistics); Nilsson 2001: 249 (catalogue, faunistics); Nilsson 2015: 216 (catalogue, faunistics).

##### Type locality.

Kenya: Samburu.

##### Type material studied

(5 exs.). Lectotype (by present designation): male: “Afrique Orle Anglaise Samburu (Wa-Nyika) Dr. Alluaud IV. 1904 / Museum Paris coll. Ch. Alluaud / TYPE / *Laccophilus productus* Rég. sp. n. typ” (MNHN; top specimen on pin with two additional paralectotypes). – Paralectotypes: Similar data and on same pin as lectotype (2 exs. MNHN); “Samburu Wa-Nyika / Afr. Orle Angl. Alluaud / Museum Paris coll. Maurice Régimbart 1908 / *productus* Rég.” (2 exs. MNHN; habitus in Fig. 382).

##### Additional material studied

(3 exs.): **Tanzania**: “Kwakiyembe D.O.Afr. April 1916 Methner / *Laccophilus productus* Rég. det. Brancucci 1982” (1 ex. ZMHB); “Narobi b. Tanga 5. 1915 Methner” (1 ex. ZMHB); “Nord-Rabeho D.O. Afr. leg. Methner” (1 ex. ZMHB).

##### Diagnosis.

*Laccophilus productus* is characterized by quite large but slender body and by peculiar dorsal colour pattern and male genitalia (penis apical half slightly twisted; extreme apex bent leftwards). The species resembles most of *Laccophilus morondavensis* which occurs in Madagascar; diagnostic features are given under diagnosis of *Laccophilus morondavensis* on p. 22.

##### Description.

Body length 4.1–4.9, width 1.9–2.5 mm. Elytra with distinct colour pattern (Fig. 382); only minor variation exhibited.

Head: Pale ferrugineous. Submat, finely and densely microsculptured. Reticulation simple; only with small, distinct meshes. Impunctate, except at eyes with scattered, fine, punctures.

Pronotum: Pale ferrugineous, mediobasally with a vague ferrugineous to dark ferrugineous marking. Submat, finely and densely microsculptured. Reticulation simple; only with small, distinct meshes. Impunctate, except at margins; with fine, somewhat sparse and irregular punctures. Mediobasally punctures absent or indistinct.

Elytra: Dark ferrugineous, with subbasal, preapical and apical, pale ferrugineous area (Fig. 382). Colour pattern stable and exhibits only minor variation. Submat, finely and densely microsculptured. Reticulation simple; only with small, distinct meshes. Fine, sparse and somewhat irregular punctures form a discal row. Dorsolateral and lateral rows indicated by scattered, fine punctures. Laterally with a comparatively long, finely pubescent, pre-apical furrow.

Ventral aspect: Dark ferrugineous to ferrugineous; colour pattern vague, indistinct. Rather shiny, finely microsculptured. Abdomen with fine, curved striae. Almost impunctate, except for apical ventrite; with scattered irregular punctures and shape symmetric (Fig. 17). Metacoxal plates with 13–15 almost transverse, fine, shallow furrows which in part are rather indistinct. Prosternal process slightly enlarged; apex moderately extended, pointed.

Legs: Pro- and mesotarsus slightly enlarged, provided with suckers.

Male genitalia: Apical half of penis in dorsal aspect slightly sinuate; extreme apex slightly bent to left (Figs 219–221).

Female: Apical ventrite apically extended (Fig. 18). Pro- and mesotarsus slender, somewhat extended.

##### Distribution.

Kenya, Tanzania (Fig. 527).

##### Collecting circumstances.

Unknown.

#### Laccophilus mirabilis

Taxon classificationAnimaliaColeopteraDytiscidae

Guignot, 1956

Figs 19 , 383 , 527

Laccophilus mirabilis Guignot 1956d: 78 (original description, faunistics); Rocchi 1991: 86 (faunistics, list); Nilsson 2001: 247 (catalogue, faunistics); Nilsson 2015: 214 (catalogue, faunistics).

##### Type locality.

Madagascar: Bas Mangoky.

##### Type material studied

(3 exs.). Holotype: female: “Type / Station Agric Bas Mangoky / Institut Scientifique Madagascar /Guignot det., 1956 *Laccophilus mirabilis* Type” (MNHN). – Paratypes: “Station Agric Bas Mangoky / female symbol / Paratype” (2 exs. MNHN; habitus in Fig. 383).

##### Diagnosis.

*Laccophilus mirabilis* belongs to a distinct group of species characterized by body shape, being longer, thicker and relatively more slender than other African *Laccophilus* species. Other diagnostic features are the body microsculpture, which is simple and fine and shape of penis which is narrow and in dorsal view peculiarly, slightly twisted. *Laccophilus mirabilis* is thus far, however, only known from female but it can be separated from closely related, continental African species by being somewhat larger and by exhibiting different colour pattern of body.

##### Description.

Body length 5.1–5.7 mm, width 2.8–3.0 mm. Only slight variation observed in elytral colour pattern (Fig. 383).

Head: Pale ferrugineous to pale brownish. Submat, finely and distinctly microsculptured. Reticulation simple, of one kind. Impunctate, except at eyes; with a few, fine and irregularly placed punctures. Additionally, in a small depression located a short distance from eyes towards middle with some fine punctures. Frontally along anterior edge with a faint, somewhat irregular impression.

Pronotum: Dark ferrugineous to ferrugineous. Laterally pronotum becomes gradually paler; pale ferrugineous. Submat, finely but distinctly microsculptured. Reticulation simple, of one kind. At margins except basally in middle with fine, sparse and irregularly located punctures. Extremely small, scattered punctures may be discerned on disc.

Elytra: Pale ferrugineous to ferrugineous, with fairly distinct dark ferrugineous markings (Fig. 383). Submat, finely and distinctly microsculptured. Reticulation simple, of one kind. Very fine, somewhat sparse and irregular punctures form a discal, dorsolateral and lateral row of punctures.

Ventral aspect: Blackish to dark ferrugineous. Prothorax ferrugineous to pale ferrugineous. Almost impunctate, except apical ventrite, which especially on apex is distinctly punctate (Fig. 19). Rather shiny, very finely and densely microsculptured. Abdomen (all visible ventrites) with somewhat sparse, curved striae. Metacoxal plates with some 10 shallow, transversely located, furrows. Prosternal process slightly enlarged, apex short, pointed.

Legs: Pro- and mesotarsus rather slender.

Male: Unknown.

##### Distribution.

Madagascar (Fig. 527).

##### Collecting circumstances.

Unknown.

#### Laccophilus ferrugo sp. n.

Taxon classificationAnimaliaColeopteraDytiscidae

http://zoobank.org/DF20C176-9844-458D-A342-94DA2A3DADC2

Figs 20–21 , 222–223 , 384 , 522

##### Type locality.

Mozambique: Prov. Inhambane, 15 km SE Save.

##### Type material

(5 exs.): Holotype, male: “Mocambique Prov. Inhambane 15 km SE Save, 18–21.12. 2005 A. Kurdna Jr. lgt.” (CFP; habitus in Fig. 384). – Paratypes, female: Same data as holotype (2 exs. CFP, 1 ex. MZH, 1 ex. NHRS).

##### Diagnosis.

Absence of dorsal colour pattern (or sometimes presence of indistinct vague darker areas on body) in combination with peculiar, abrupt end of penis-apex, distinguishes *Laccophilus ferrugo* from the other species in this species group.

##### Description.

Body length 4.2–4.7 mm, breadth 2.2–2.4 mm. Dorsal, colour pattern lacking or very indistinct and vaguely delimited (Fig. 384).

Head: Ferrugineous; frontally often narrowly and slightly paler; with a vague, pale ferrugineous border at foremargin. Impunctate, except at eyes; with scattered, irregular punctures. Slightly matt, reticulation, simple, meshes small and of equal size.

Pronotum: Ferrugineous, no distinct colour pattern. Impunctate, except at margins; with fine, irregular punctures, which are lacking medially at base. Slightly matt, with fine, simple reticulation; meshes small and of equal size.

Elytra: Ferrugineous to dark ferrugineous; sometimes with vague, dark ferrugineous to blackish areas (one at scutellar region, one extensive on medial part, and one apically). Dark areas, when present a very diffuse and their delimitation vague (Fig. 384). Submat, finely and equally microsculptured; meshes small and of equal size. Discally with a fine, irregular row of punctures. Dorsolateral row and lateral row of punctures sparse, irregular and especially lateral one is fragmentary. Apically with sparse and irregularly distributed punctures of variable size.

Ventral aspect: Ferrugineous, abdomen in part darker; ferrugineous to dark ferrugineous. Almost impunctate. Scattered punctures on apical ventrite which lacks small knob on one side (Fig. 20). Rather shiny, with very fine, in part, indistinct microsculpture. Apex of prosternal process quite slender, posteriorly moderately extended, apex narrows quite abruptly (approx. as Fig. 1). Metacoxal plates in frontal half provided with, fine, almost transversely located, shallow furrows. Abdomen with fine, curved striae. Metacoxal process ends abruptly; no posterior extensions (Fig. 6).

Legs: Pale ferrugineous to ferrugineous. Pro- and mesotarsus slightly enlarged, provided with suckers. Apex of metatibial spines bifid, although very finely so.

Male genitalia: Penis, lateral aspect, from approximately middle to apex evenly curved; tip somewhat enlarged, ends abruptly (Figs 222–223).

Female: Pro- and mesotarsus slender. Apical sternite (Fig. 21).

##### Etymology.

The species name “ferrugo” is a Latin noun meaning rust (of iron) and relates to the body colour of the new species.

##### Distribution.

Mozambique (Fig. 522).

##### Collecting circumstances.

Unknown.

### Species group 2 (*Laccophilus ruficollis* group)

**Diagnosis.** Quite small sized *Laccophilus* with length of body 3.2–3.4 mm and width 1.8 mm.

Shape of body oval; body dorsoventrally flattened (Fig. 380). Body dorsally, with distinct colour pattern, formed by two distinct, transverse, pale ferrugineous markings, which are broadly broken by dark suture; apex of elytra also pale coloured (Fig. 385). Body microsculpture on dorsal aspect double, of two kinds (in part meshes reduced).

**Figures 22–30.**
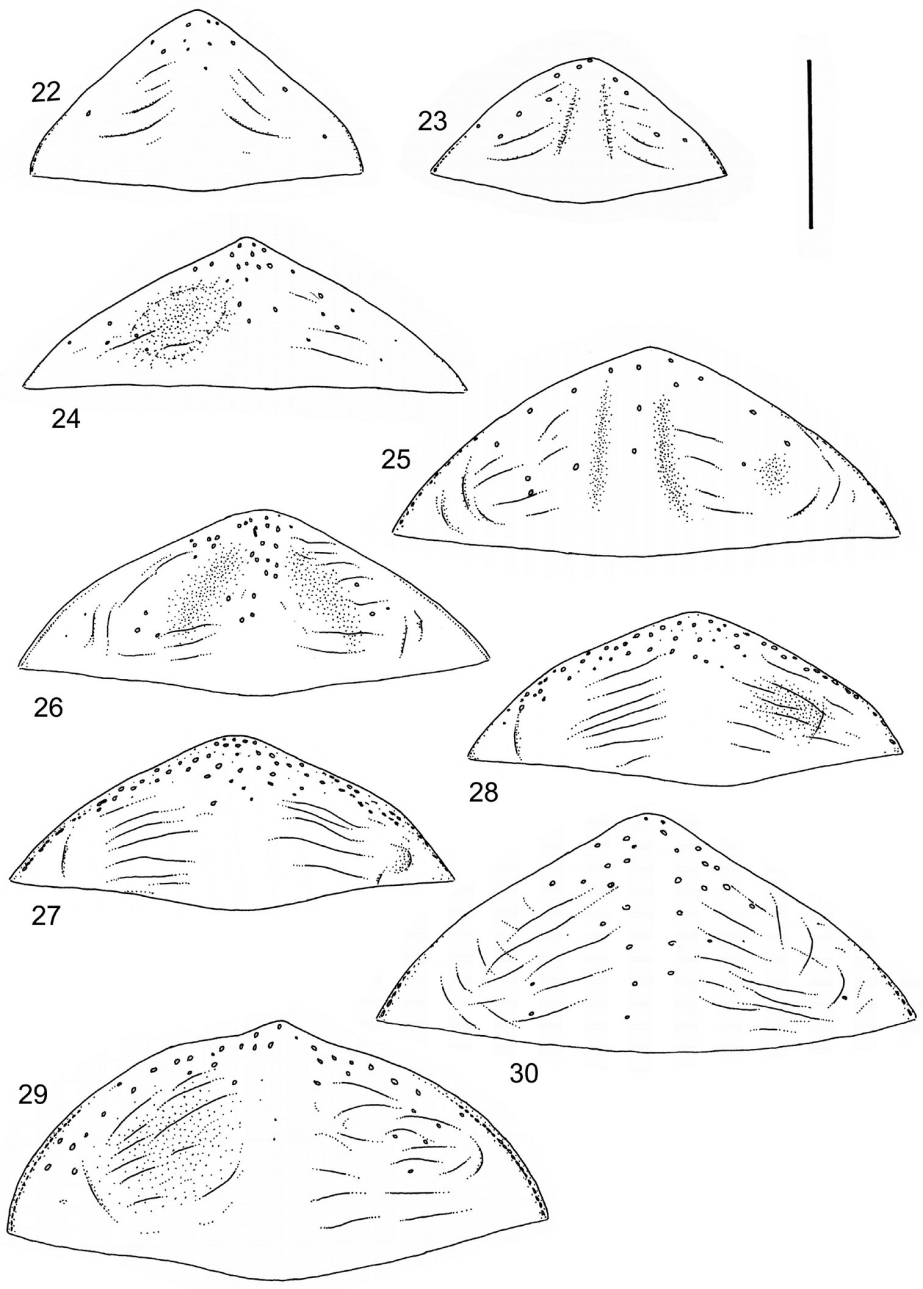
Apical ventrites **22** *Laccophilus ruficollis* male, and **23** female **24** *Laccophilus hyalinus* male, and **25** female **26** *Laccophilus demoflysi* female **27** *Laccophilus minutus* male, and **28** female **29** *Laccophilus mateui* male, and **30** female. Scale bar 0.5 mm.

Prosternal process moderately broad, posteriorly not distinctly extended, apically pointed. Apical sternite not distinctly modified; lacks asymmetrical, small knob (Fig. 22). No stridulatory apparatus on metacoxal plates. Metacoxal process not extended posteriorly (Fig. 6).

Paramere simple, elongate, apically not distinctly enlarged or modified (Fig. 224). Apical half of penis slender, distinctly curved but not distinctly modified (Fig. 224).

**Species composition and distribution.** One African species is recognized. Only known from Madagascar.

#### Laccophilus ruficollis

Taxon classificationAnimaliaColeopteraDytiscidae

Zimmermann, 1919

Figs 22–23 , 224 , 385 , 527

Laccophilus ruficollis Zimmermann 1919: 123 (original description, faunistics); Zimmermann 1920a: 25 (catalogue); Nilsson 2001: 250 (catalogue, faunistics); Nilsson 2015: 217 (catalogue, faunistics).

##### Type locality.

Madagascar.

##### Type material, studied

(3 exs.). Lectotype (by present designation): male: “Madagascar / Type / Samml. A. Zimmermann / Paratypus” (ZSM; habitus in Fig. 385). – Paralectotypes, male and female: Same data as in lectotype (2 exs. ZSM). [Comment: all three specimens studied are provided with a type and a paratype label and no holotype has been chosen. Two of the specimens are females and one, male. We have chosen the male to be the lectotype.]

##### Diagnosis.

*Laccophilus ruficollis* is distinguished from all other African species by unmodified apical ventrite and exhibiting distinct, transverse, pale markings on elytra. Furthermore, penis apex is slender and curved and body-microsculpture is a mix of small and large meshes. In combination with small sized body these characters are useful when *Laccophilus ruficollis* is distinguished.

##### Description.

Body length 3.2–3.4 mm, width 1.8 mm. Dorsal colour pattern rather uniform and distinct (Fig. 385).

Head: Pale ferrugineous. Rather shiny although finely microsculptured. Reticulation almost simple, predominantly of one kind. In part reticulation indistinctly double but small and large meshes difficult to distinguish and place in either category. Between eyes, with fine, sparse punctures. At eyes punctures slightly denser.

Pronotum: Ferrugineous, laterally pale ferrugineous (change of colour gradual). Basally with vague, transverse, dark ferrugineous marking. Rather shiny although finely microsculptured; reticulation mostly uniform: small and large meshes difficult to distinguish. Laterally and at anterior margin, finely punctate.

Elytra: Dark ferrugineous, with distinct pale ferrugineous markings (Fig. 385). Slightly mat, finely and densely microsculptured. Reticulation-meshes not clearly forming two distinct groups. Almost impunctate; laterally and at suture with fine punctures.

Ventral aspect: Pale ferrugineous to ferrugineous; lacks distinct colour pattern, but abdomen in part slightly darker. Rather shiny, very finely microsculptured (in part microsculpture hardly discernible). Scattered, curved striae discernible but sometimes rather indistinct. Almost impunctate. Apex of prosternal process comparatively short although pointed. Apical ventrite simple, not distinctly modified (Fig. 22).

Legs: Protarsus slightly extended and enlarged; mesotarsus long and slender. Provided with suckers.

Male genitalia: Rather delicate in size and exhibits hardly any modifications; penis in lateral aspect slender and evenly curved (Fig. 224).

Female: Externally almost as male. Protarsus slender. Apical ventrite as in Fig. 23.

##### Distribution.

Madagascar (Fig. 527).

##### Collecting circumstances.

Unknown.

### Species group 3 (*Laccophilus hyalinus* group)

**Diagnosis.** Large species with body length 4.0–5.3 mm, and width 2.2–3.0 mm.

Shape of body oblong to oval, dorsoventrally flattened (Figs 386–387). Dorsal side unicoloured pale ferrugineous to ferrugineous, generally lacking colour pattern. Some species exhibit vague pattern; elytra ferrugineous with pale ferrugineous, often vague and variable patches (Fig. 389). Body microsculpture double; small and large meshes mixed, in part meshes often reduced and missing.

Prosternal process moderately slender, posteriorly not strongly extended, apex pointed. Apical ventrite not distinctly modified; lacks asymmetric knob on one side (Fig. 24). Two species have in both sexes stridulatory files on metacoxal plates (Fig. 6). Metacoxal process not extended posteriorly (Fig. 6).

Paramere simple, elongate, apically not distinctly enlarged or modified (Fig. 229). Apical half of penis slender, distinctly curved but not distinctly modified (Fig. 228). In *Laccophilus hyalinus* extreme apex of penis finely hooked (Fig. 225).

**Species composition and distribution.** Five species are recognized in this species group. In Africa they are distributed North of Sahara and most of them exhibit a wider distribution in the Palearctic region.

#### Key to species (males & females)

**Table:** 

1	Metacoxal plates with stridulatory file (Fig. 6)	**2**
–	Metacoxal plates lack stridulatory file	**3**
2	Body shape oval-oblong, narrower (Fig. 386); penis as in Fig. 225	***Laccophilus hyalinus*** (p. 28)
–	Body shape oval, broader (Fig. 387); (female only known)	***Laccophilus demoflysi*** (p. 31)
3.	Smaller species, body length 4.0–4.6 mm; male genitalia slender (Fig. 226)	***Laccophilus minutus*** (p. 31)
–	Large species, body length 4.6–5.3 mm; male genitalia robust (Fig. 227)	**4**
4.	Elytra with distinct colour pattern (Fig. 389); male genitalia (Fig. 227)	***Laccophilus mateui*** (p. 33)
–	Elytra lack colour pattern or pattern is indistinct (Fig. 390); male genitalia (Fig. 228–229)	***Laccophilus sordidus*** (p. 35)

#### Laccophilus hyalinus

Taxon classificationAnimaliaColeopteraDytiscidae

(De Geer, 1774)

Figs 6 , 9–10 , 24–25 , 225 , 386 , 528

Dytiscus hyalinus De Geer 1774: 406 (original description, faunistics).
Laccophilus hyalinus (De Geer), Reiche 1872: 23 (faunistics, list.); Sharp 1882: 301 (description, faunistics); Zimmermann 1920a:19 (catalogue); Zimmermann 1930: 14 (description, faunistics); Normand 1938: 343 (catalogue); Guignot 1946b: 186 (discussion, faunistics); Omer-Cooper 1970: 286 (faunistics, discussion); El Alaoui 1983: 133, 135 (faunistics); Brancucci 1983b: 268 (faunistics); Nilsson 2001: 244 (catalogue, faunistics); Nilsson 2015: 212 (catalogue, faunistics).
Laccophilus testaceus Aubé, Reiche 1872: 23 (faunistics, list); Nilsson 2015: 212 (catalogue, faunistics, list synonymy).
Laccophilus interruptus var. testaceus Aubé, Severin 1892: 472 (type material deposition); Régimbart 1895: 132 (faunistics, *Laccophilus interruptus* = *Laccophilus hyalinus*, synonymy).
Laccophilus hyalinus var. testaceus Aubé, Bertrand 1928b: 46 (larva description); Guignot 1946b: 186 (faunistics, discussion).
Laccophilus hyalinus testaceus Aubé, Zimmermann 1920a:19 (catalogue); Lindberg 1939: 11, 13, 29 (biology, faunistics); Angelini 1982: 82 (faunistics); Nilsson 2003: 76 (faunistics, list); Bennas and Sàinz-Cantero 2006: 58, 62 (faunistics, list).
Laccophilus hyalinus ab. testaceus Aubé, Legros 1972: 467 (faunistics).
Laccophilus hyalinus var. inflatus Wollaston, Machado 1987: 50 (description, faunistics); Nilsson 2015: 212 (catalogue, faunistics, list synonymy).
Laccophilus hyalinus inflatus Wollaston, Balke et al. 1990: 361, 369, 370 (discussion, faunistics, biology).

##### Comment on synonymy.

The present list of references is incomplete; selected references with association to Africa and Canary Islands are included. Synonymy of different taxa is based on earlier studies (see World Catalogue, Nilsson 2001, 2015).

##### Type locality.

Sweden.

##### Type material, studied

(1 ex.). *Laccophilus hyalinus*: Syntype (unique?): (NHRS). [Comments: no original text labels attached with the specimen; specimen provided with orange label; severely damaged by dermestids.]

##### Type material, not studied.

*Laccophilus testaceus*: “France, Italy, Spain” (in Brussels).

##### Additional African material studied

(310 exs.): **Morocco**: “Tanger 25–29.4. 1926 Lindberg” (7 exs. MZH); “Marrakesh 21–23.5. 1926 Lindberg” (5 exs. MZH); “Marrakech 28.5. 1934, 450 m Ball 20M79 / ab. testaceus” (1 ex. MNHN); “Atlas mai, Reraia 29.5–15.6. 1926 Lindberg” (6 exs. MZH); “Atlas mai., Amismiz 24–25.5. 1926 Lindberg” (1 ex. MZH); “M. Atlas Azrou 1200 m 15.3. 1961 Lindberg” (4 exs. MZH); “Foret de la Mamora 23.3.1961 Lindberg” (1 ex. MZH); “Tiflet-Oulmes 18.2.1961 Meinander” (1 ex. MZH); “Oued Tensift pr., Marrakesh 13.3. 1961 Lindberg” (1 ex. MZH); “nr. Figuig, Defilia 5–20.4. 1966/Hutson” (1 ex. BMNH); “Mar. bor. Tetouan 600 m 25.5. 1994 leg. Majzlan” (14 exs. NMW, 3 exs. MZH); “Ouazaza-Te 12.5. 1975 Eckerlein” (7 exs. NMW, 1 ex. MZH); “Oulad Teima pr. Oued Sous 14.2. 1961 Lindberg” (4 exs. MZH); “Mogador (Essaouira) 12.2. 1961 Meinander” (12 esx. MZH); “Tiferhlal N de Tiznit 18.2. 1961 Meinander” (6 exs. MZH); “Oued Massa N de Tiznit 18.2. 1961 Meinander” (3 exs. MZH); “Maroc sud Torkoz 23–24.2. 1961 Meinander” (7 exs. MZH); “Maroc sud Assa 22.2. 1961 Lindberg” (5 exs. MZH; habitus in Fig. 386). – **Algeria**: “55 km N of Tamanrasset 16–17.3. 1971 Gruwell leg.” (90 exs. USNM, 10 exs. MZH); “Bouqie 17.7. 1955 Lauck leg.” (1 ex. USNM). – **Tunisia**: “Totzeur 5.4. 1924 Lindberg” (1 ex. MZH); “Ichkeulsee 1.8. 1991 Schödl” (2 exs. NMW); “Djeb. Ressas / J. Sahlb.” (1 ex. MZH). – **Libya**: “Libya bor. occ. 653 asl, prov. Yafran 9.5.2002 Ain Az-Zarqa (W of Jadu) / 31°57'21.2"N, 12°00'25.9"E, Reiter A. lgt.” (27 exs. NMPC); “Libya Darnah prov. Wadi Darnah, 117 m, 32°42'06.4"N, 22°36'39.9"E, A. Reiter leg. 15.5. 2002” (17 exx. NMPC); “Libya bor. occ. 605 m asl, prov. Yafran-Ghadamis Nana tala, 10 km W Ar Rhaibat 27.5.2002 / 31°47'09"N, 11°47'07.9” Reiter A. lgt.” (3 exs. NMPC); “Libya bor. occ. 336 m asl, prov. Tarhunah 26.5.2002 Ain Sharshara, 3 km N Tarnuah / 32°27'57.7"N, 13°37'04.7” E. Reiter A lgt” (25 exs. NMPC). – **Egypt**: “Wadi Kujib 12.6. 1994 Ullrich / *Laccophilus hyalinus* Deg. Hendrich det. 1994” (24 exs. USNM); “Madiba Wala 20.6. 1994 Ullrich / *Laccophilus hyalinus* Deg. Hendrich det. 1994” (4 exs. USNM). – **Canary Islands (Spain)**: “Tenerife, Valle de Masca 12–13.5. 1947 Lindberg” (1 ex. MZH); “Tenerife, Bco Bufadero 10.8. 1949 Fernandez” (4 exs. MZH); “Gran Canaria, Aldea S. Nicolas 1.3. 1949 Lindberg” (9 exs. MZH); “Gran Canaria, Maspalomas 9-10.3. 1950 Lindberg” (1 ex. MZH).

##### Diagnosis.

*Laccophilus hyalinus* resembles most among African species of *Laccophilus demoflysi*, which also has a similar stridulation apparatus as *Laccophilus hyalinus*. For separation of the two species, see diagnosis of *Laccophilus demoflysi* (p. 31). Stridulation apparatus located on metacoxal plates distinguishes *Laccophilus hyalinus* from *Laccophilus minutus*, *Laccophilus mateui* and *Laccophilus sordidus*, all of which lack similar device. Clear differences between the species are also exhibited in shape of the penis.

##### Description.

Body length 4.7–5.0 mm, width 2.7–2.8 mm. Habitus generally with somewhat paler, longitudinal markings, which often are rather vague, in part indistinct (Fig. 386).

Head: Pale ferrugineous. At eyes, in shallow depression with fine punctures. In short horizontal depressions close to eyes and in connection with shallow depression, with fine punctures. Reticulation double, large meshes contain 2–6 fine meshes. Fine meshes in part weakly developed and indistinct.

Pronotum: Pale ferrugineous. At foremargin and medially with slightly darker areas (dark areas not on surface but “inside” cuticula). Almost impunctate, with a few irregular punctures at frontal margin. Rather finely microsculptured. Reticulation double. Fine meshes in part largely absent, in part very fine to fine. Large meshes, when discernible, contain 4–6 fine meshes.

Elytra: Pale ferrugineous to pale brown, with slightly paler, in part indistinct markings (Fig. 386). Elytral punctation almost absent; discally, dorsolaterally and laterally with a few, very fine, hardly visible punctures placed in vague rows. Slightly mat due to microsculpture. Reticulation double, large meshes contain 2–6 small meshes.

Ventral aspect: Pale ferrugineous. Impunctate. Slightly mat, due to dense, fine microsculpture. Metacoxal plates with about 10 transverse, shallow furrows. Basal segments of abdomen with curved striae. Pronotal process medially slightly enlarged, apex pointed (not strongly extended and sharp). Stridulation apparatus consist of a curved series of about 20 shallow ridges located posteriorly on metacoxa (Fig. 6). Apical ventrite with a shallow depression on one side (Fig. 22).

Legs: Pro- and mesotarsus slightly enlarged, provided with distinct suckers (Fig. 10).

Male genitalia: Extreme apex of penis in lateral aspect strongly curved upwards (Fig. 225).

Female: Provided with similar stridulation apparatus as male. Apical ventrite with uneven surface (Fig. 25). Pro- and mesotarsus rather slender.

##### Distribution.

Morocco, Algeria, Tunisia, Libya, Egypt, Canary Islands (Fig. 528). Additional, African country record is Senegal (Legros 1972).

##### Collecting circumstances.

Information from Africa is rare. In Europe often found in large bodies of running water, in sections with a slow current and some vegetation. Less frequently collected in ponds and lakes (Nilsson and Holmen 1995).

#### Laccophilus demoflysi

Taxon classificationAnimaliaColeopteraDytiscidae

Normand, 1938

Figs 26 , 387 , 529

Laccophilus demoflysi Normand 1938: 343 (original description, faunistics); Nilsson 2001: 242 (catalogue, faunistics); Nilsson 2003a: 76 (faunistics, list); Nilsson 2015: 211 (catalogue, faunistics).

##### Type locality.

Tunisia: El Hamma de Tozeur.

##### Type material

(not studied): Holotype: female: “El Hamma de Tozeur, 4. 1937 Demoflys” (Coll. Normand, kept in Tunisia, Institut National Agronomique de Tunisie, Tunis, specimen not located).

##### Material studied.

(1 ex.): **Tunisia**: “Tunisia, centralis oasis Douz env. 31.5.-1.6. 1994 lgt.S. Becvar / *Laccophilus demoflysi* Norm.det. Rocchi 1998” (1 ex. female CSR; habitus in Fig. 387).

##### Diagnosis.

External characters agree in large with *Laccophilus hyalinus*. Only difference observed was the shape of the body, in *Laccophilus demoflysi* being stouter than in *Laccophilus hyalinus*. Both involved species have a stridulatory apparatus on metacoxal plates, which separates them from *Laccophilus minutus*, *Laccophilus mateui* and *Laccophilus sordidus*. Taxonomic status of *Laccophilus demoflysi* remains open. More specimens (male in particular) are needed to settle this question.

##### Description

(only differences from description of *Laccophilus hyalinus* are recognized). Body length 4.7 mm, width 2.8 mm. Dorsal colour pattern vague, almost absent (Fig. 387).

Ventral aspect: Apical ventrite (Fig. 26).

Male: Unknown.

##### Distribution.

Tunisia (Fig. 529).

##### Collecting circumstances.

Unknown.

#### Laccophilus minutus

Taxon classificationAnimaliaColeopteraDytiscidae

(Linnaeus, 1758)

Figs 27–28 , 226 , 388 , 529

Dytiscus minutus Linnaeus 1758: 412 (original description, faunistics).
Laccophilus minutus (Linnaeus), Leach 1815: 673 (description, biology); Aubé 1838: 417 (description, faunistics); Lucas 1846: 94 (faunistics); Reiche 1872: 23 (faunistics, list); Régimbart 1895: 132 (faunistics; given as *Laccophilus obscurus* Panzer, junior synonym of *Laccophilus minutus*); Zimmermann 1920a: 21 (catalogue); Bertrand 1928b: 274 (juvenile description); Zimmermann 1930: 14 (description, faunistics); Lindberg 1939: 11, 13, 29 (biology, faunistics); Brinck 1943: 154 (faunistics); Balfour-Browne 1951: 193 (discussion); Sanfilippo 1955: 1 (faunistics, biology); Guignot 1956b: 220 (discussion); Guignot 1959a: 579, 583 (redescription, faunistics); Angelini 1982: 82 (faunistics); El Alaoui 1983: 133, 135 (faunistics); Nilsson 2001: 247 (catalogue, faunistics); Nilsson 2003: 76 (faunistics, list); Bennas and Sàinz-Cantero 2006: 59, 62 (faunistics, list); Nilsson 2015: 214 (catalogue, faunistics). [Comments: Only references referring to Africa are listed. The list of synonyms and references is accordingly, not complete. We refer to World catalogue of Nilsson (2015)]

##### Type locality.

Europe.

##### Type material

(not examined): “Europe”. Type specimen deposited in The Linnean Collections, London, UK (item data: LINN 6347 *Dytiscus minutus* (Ins. Linn.), www.linnean.org).

##### African material studied

(49 exs.). **Morocco**: “Tanger 25-29.4. 1926 Lindberg” (6 exs. MZH); “Tiflet-Oulmes 18.3.1961 Lindberg-Meinander” (4 exs. MZH; habitus in Fig. 388); “Sp. Mor., at Tangier border 8.7.1955” (1 ex. USNM); “Rabat 3–4.5. 1926 Lindberg” (2 exs. MZH); “Gharb 7.7. 1926 Lindberg” (11 exs. MZH); “Marrakesch 21–23.5. 1926 Lindberg” (6 exs. MZH); “Dayet Jerans (lake) 11 km E.N.E. Ifrane 5400 ft. 28.5. 1961 / P.N. Lawrence” (1 ex. BMNH); “Middle Atlas, nr Ifrane 28.5. 1961 Dayet Jerane P.N. Lawrence” (1 ex. BMNH, 1 ex. MZH); “Oued Zad 65 km S Ifrane 21.5. 1961 / P. N. Lawrence / Tiny pond, flooded grass” (1 ex. BMNH); “Fr. Mor., Petit Jean 9.7. 1955 D.L. Lauck” (6 exs. USNM, 2 exs. MZH); “Sp. Mor., El Kaar, El Kebir 8.7.1955 D.R. Lauck” (2 exs. USNM). – **Algeria**: “Bouqie 17.7. 1955 D.R. Lauck” (1 ex. USNM). – **Tunisia**: “Tunis J. Sahlb.” (1 ex. MZH); “Tunisien 2.9. 1991 5 km W Utique Schödl” (1 ex. NMW); “Tunisien 3.8. 1991, 22 km N Jendouba Schödl” (1 ex. NMW). – **Libya**: “Libya bor. or. 495 m a.s.l. prov. Al Jabal Al Akhdar, 5 km SW Al Bayda 18.5.2002 / 32°43'41.9"N, 21°41'14"E, Reiter A. lgt.” (1 ex. NMPC). [Comment: Only material collected from Africa is listed.]

##### Diagnosis.

*Laccophilus minutus* resembles most of *Laccophilus mateui* and *Laccophilus sordidus*, both species also lacking stridulation apparatus on metacoxal plates. *Laccophilus minutus* is separated from the two close species by having smaller body and more delicate and slender penis (Figs 226, 227, 228).

##### Description.

Body length 4.0–4.6 mm, width 2.2–2.5 mm. Dorsal, aspect of body without distinct colour pattern. On elytra vague, slightly darker areas may sometimes be discerned (Fig. 388).

Head: Pale ferrugineous. Slightly mat to rather shiny, finely microsculptured. Reticulation double. Large meshes slightly more strongly developed than fine meshes. Large meshes may contain 3–6 small meshes. Impunctate, except at inner eye-margin, with an irregular row of punctures. Additionally close to eyes with a vertically located row of a few punctures.

Pronotum: Pale ferrugineous; lacks distinct colour pattern. Impunctate, except at frontal and lateral margins, where fine and sparse, irregular punctures are discernible. Rather shiny to submat, finely microsculptured. Reticulation double. Large meshes a little more strongly developed than small meshes; may contain 3–6 small meshes. Pronotum base posteriorly in middle produced backwards.

Elytra: Pale ferrugineous, without distinct colour pattern. Sometimes elytra with some vague, irregular, slightly darker areas (Fig. 388). Almost impunctate. Discally with sparse, fine and scattered punctures forming a vague row. Additionally, some scattered, fine punctures may be discerned at location of dorsolateral and lateral rows. Pre-apical, lateral row of punctures form a fine furrow, provided with fine hairs. Rather shiny, although finely microsculptured. Reticulation double. Large meshes a little more strongly developed; may contain 3-6 smaller meshes.

Ventral aspect: Pale ferrugineous to ferrugineous, without distinct colour pattern. Almost impunctate, Abdomen apically with some fine punctures. Rather shiny, very finely and in part indistinctly microsculptured. Ventrites with fine, slightly curved striae. Metacoxal plates with about 10 fine, shallow, transversely located furrows. No stridulatory apparatus. Apical ventrite almost symmetric, lacks lateral knob; finely striated, with distinct lateral impressions (Fig. 27). Prosternal process rather slender; posteriorly, moderately extended, apex pointed.

Legs: Pale ferrugineous to ferrugineous. Pro- and mesotarsus somewhat enlarged, provided with suckers.

Male genitalia: Aedeagus almost as in *Laccophilus mateui* but more delicate in lateral aspect (Fig. 226).

Female: Pro- and mesotarsus slender. Apical ventrite lacks distinct lateral impressions; impressions reduced to fine lines (Fig. 28).

##### Distribution.

Morocco, Algeria, Tunisia, Libya (Fig. 529).

##### Collecting circumstances.

According to Nilsson and Holmen (1995), probably focusing from Nordic perspective, main habitat is permanent water body as lakes and ponds with stagnant water. Vegetation of water body is sparse or absent. Lindberg (1939) briefly described some sampling sites of *Laccophilus minutus* in Morocco. The species was collected both in standing and running waters. Collected also from an almost dried up river-bed with rich vegetation. Adults are capable of flight.

#### Laccophilus mateui

Taxon classificationAnimaliaColeopteraDytiscidae

Omer-Cooper, 1970

Figs 29–30 , 227 , 389 , 529

Laccophilus mateui Omer-Cooper 1970: 285, 287 (original description, faunistics); Nilsson 2001: 246 (catalogue, faunistics); Nilsson 2003: 76 (catalogue, faunistics); Nilsson 2015: 214 (catalogue, faunistics).

##### Type locality.

Algeria: Sahara, Hoggar, Aguelm, Ymeleulauen.

##### Type material, studied

(1 ex.). Holotype: male: “Type male / H. B. Leech Collection / det. J. Omer-Cooper *Laccophilus mateui* sp.n. / Aguelm, Ymeleulauen, Hoggar, Sahara J. Mateu coll. / 18-V- 1951” (AMGS; according to original description, holotype to be deposited in CAS).

##### Additional material, studied

(5 exs.). **Algeria**: “55 km N Tamanrasset 16-17 March 1971 J.A. Gruwell” (4 exs. USNM, 1 ex. MZH; habitus in Fig. 389).

##### Diagnosis.

*Laccophilus mateui* is a close relative to *Laccophilus minutus* and *Laccophilus sordidus*. All three species have similar general appearance and same ground plan regarding penis-shape. Absence of stridulatory apparatus separates it from *Laccophilus hyalinus* and *Laccophilus demoflysi*. Shape of penis distinguishes it from *Laccophilus minutus* (penis apex is broader in *Laccophilus mateui*) and *Laccophilus sordidus* (penis is stouter in *Laccophilus mateui* and longer in *Laccophilus sordidus*). *Laccophilus mateui* (>5 mm) is also larger sized than *Laccophilus minutus*, a species which don’t exceed 5 mm in length.

##### Description.

Body length 5.1–5.3 mm, width 2.8–3.0 mm. Body dorsally pale ferrugineous to ferrugineous, elytral colour pattern vague to fairly distinct (Fig. 389).

Head: Pale ferrugineous, at pronotum darker, ferrugineous to dark ferrugineous (delimitation of colours often vague). Almost impunctate, at eyes with a few, scattered, somewhat indistinct punctures. Submat, finely microsculptured; reticulation double. In central part of head fine reticulation indistinct, in part obliterated; in lateral parts of head fine reticulation clearly discernible; large meshes contain 3–6 fine meshes.

Pronotum: Ferrugineous, laterally pale ferrugineous (gradual change; no distinct delimitation of colours). Frontally, sometimes with a quite distinct dark ferrugineous area. Impunctate, at margin with a few, indistinct, coarser punctures discernible. Submat, rather densely microsculptured; reticulation double, fine meshes in part almost absent or indistinct.

Elytra: Ferrugineous, with vague pale ferrugineous markings (Fig. 389). Elytral colour pattern sometimes quite distinct. Submat, rather finely microsculptured; reticulation double, large meshes contain between 2–6 fine meshes. Each elytron with a discal, dorsolateral and lateral row of punctures, which are sparse and somewhat irregular, in part indistinct. Lateral, pre-apical furrow long, shallow, finely punctate and pubescent.

Ventral aspect: Ferrugineous, metacoxal plate in part dark ferrugineous but no distinct colour pattern formed (delimitation vague). Almost impunctate. Submat, finely microsculptured. No stridulatory apparatus. Metacoxal plates in anterior half with some vague, transversely located, slightly irregular furrows. Abdomen in basal half with rather distinct curved striae. Apex of prosternal process broken in holotype; it is keeled, short and apex pointed. Apical ventrite as in Fig. 29.

Legs: Pro- and mesotarsus somewhat enlarged, provided with suckers.

Male genitalia: Penis comparatively robust; in lateral aspect apical half evenly curved (Fig. 227).

Female: Pro- and mesotarsus slender. Apical ventrite as in Fig. 30.

##### Distribution.

Algeria (Hoggar, Sahara) (Fig. 529).

##### Collecting circumstances.

Unknown.

#### Laccophilus sordidus

Taxon classificationAnimaliaColeopteraDytiscidae

Sharp, 1882

**Figures 31–39.**
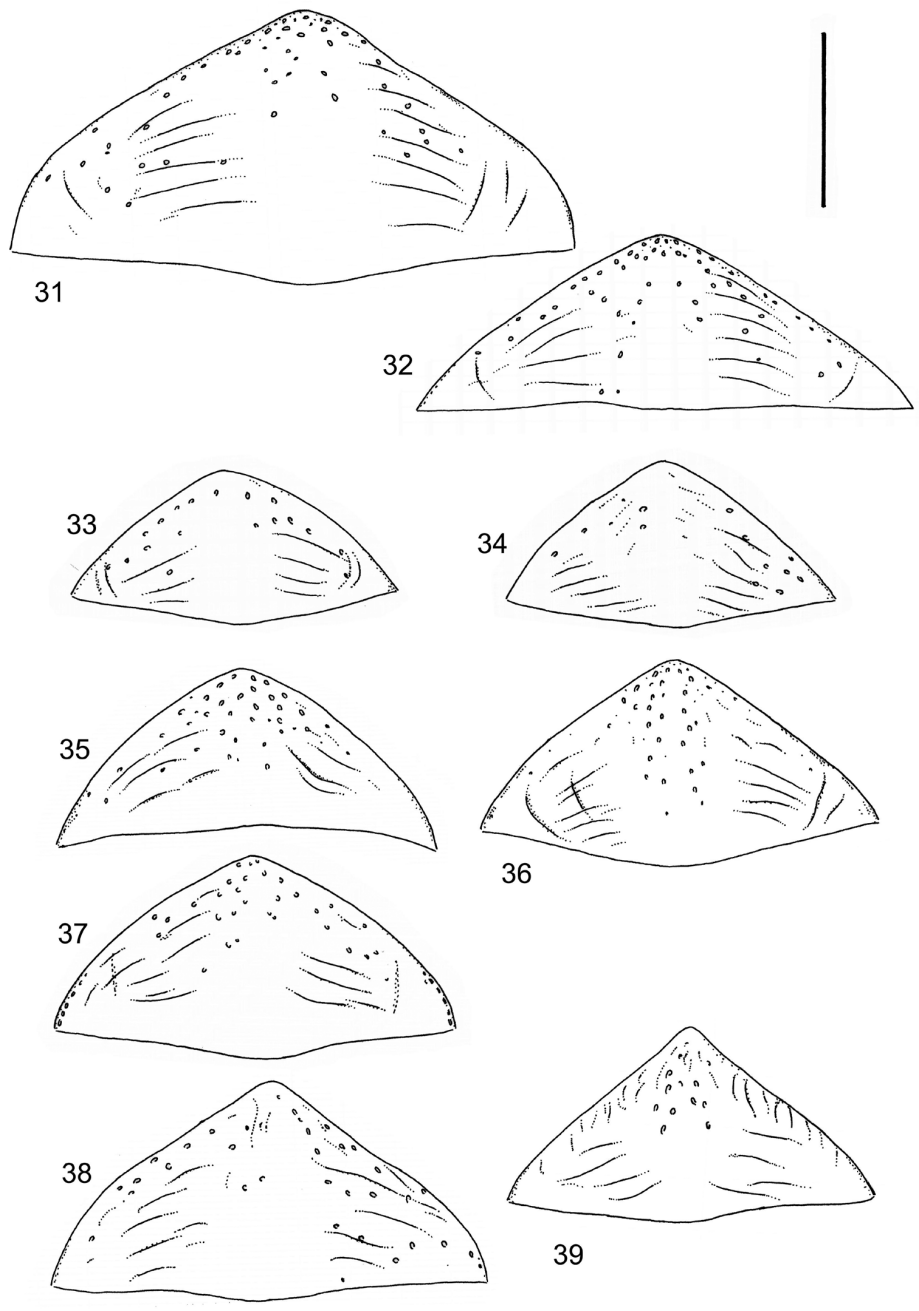
Apical ventrites **31** *Laccophilus sordidus* male, and **32** female **33** *Laccophilus comes* male, and **34** female **35** *Laccophilus alluaudi* male, and **36** female **37** *Laccophilus furthi* male **38** *Laccophilus tigrinus* male, and **39** female. Scale bar 0.5 mm.

Figs 31–32 , 228–229 , 390 , 529

Laccophilus sordidus Sharp 1882: 302 (original description, faunistics); Zimmermann 1920a: 26 (catalogue); Balfour-Browne 1951: 193 (faunistics, discussion, description); Brancucci 1980: 107 (description, faunistics, lectotype designation); Brancucci 1983b: 264, 266 (redescription, faunistics, discussion); Zalat et al. 2000: 39, 40 (description, faunistics, biology); Nilsson 2001: 251 (catalogue, faunistics); Nilsson 2003: 77 (catalogue faunistics, ); Shaverdo et al. 2013: 21, 22 (faunistics); Nilsson 2015: 218 (catalogue, faunistics).

##### Type locality.

Saudi Arabia: El Hedjaz.

##### Type material, studied

(2 exs.). Lectotype: male, designated by Brancucci (1980a): “Syntype / Type / Hedjaz Millingen / Sharp Coll. 1905-313 / *Laccophilus sordidus* / *Laccophilus sordidus* Sharp Type (male symbol)” (BMNH; habitus in Fig. 390). – Paralectotype, female: “Hedjaz Millingen / Sharp Coll. 1905-313 / *Laccophilus sordidus* Sharp Paratype (female symbol) / Syntype” (1 ex. BMNH).

##### Additional material studied

(1 ex.): Yemen: “Aden Prot., Mukeiras, 85 mls NE of Aden, 7000 ft. 29.12. 1939-6.1. 1940 Hebbert / *Laccophilus sordidus* Shp det J. Balfour-Browne” (1 ex. BMNH).

##### Diagnosis.

*Laccophilus sordidus* is closely related to *Laccophilus minutus* and *Laccophilus mateui*. From *Laccophilus minutus*, *Laccophilus sordidus* is separated by its larger body and by having a clearly longer penis. From *Laccophilus mateui*, *Laccophilus sordidus* is separated by its lack of elytral markings – *Laccophilus mateui* is generally provided with a clearly discernible elytral colour pattern. Additionally, the penis of *Laccophilus mateui* is distinctly shorter than in *Laccophilus sordidus*.

##### Description.

Body length 4.6–5.2 mm, width 2.6–2.8 mm. Dorsal, aspect of body dark ferrugineous to brownish, no distinct colour pattern exhibited (Fig. 390).

Head: Dark ferrugineous to brownish; no colour pattern discernible. Submat to mat, distinctly microsculptured; reticulation double. Large meshes may contain 3–6 small meshes. At eyes with an irregular row of punctures, which extends a short distance towards middle.

Pronotum: Dark ferrugineous to brownish, no colour pattern formed. Submat, distinctly microsculptured. Reticulation double. Large meshes contain 3–6 small meshes. Pronotal disc impunctate; at margins with punctures. Laterally at side margin, punctures form a slightly irregular row. Anteriorly punctures very fine and scattered. Latero-basally with a few fine, irregular punctures; mediobasally pronotum impunctate.

Elytra: Dark ferrugineous to brownish. No distinct colour pattern exhibited (Fig. 390). Submat, distinctly microsculptured. Reticulation double; large meshes contain generally 3–6 small meshes. Three somewhat irregular rows of punctures formed. Discal row is rather distinct while dorsolateral and lateral rows are quite vague and punctures appear more scattered. Posteriorly lateral row become more condensed and it is located in a shallow pre-apical furrow.

Ventral aspect: Dark ferrugineous to ferrugineous, no distinct colour pattern. Submat, finely microsculptured. Microsculpture of abdomen weaker and in part reduced. Abdomen with fine, curved striae. Metacoxal plates lack stridulatory apparatus. Very shallow, rudimentary transverse furrows discernible on metacoxa. Impunctate, except abdomen with scattered, sparse punctures especially apically. Prosternal process medially slightly enlarged, moderately extended and apex pointed. Apical ventrite almost symmetric (Fig. 31).

Legs: Ferrugineous to dark ferrugineous. Pro- and mesotarsus slightly enlarged and provided with suckers.

Male genitalia: In lateral aspect penis comparatively long, evenly curved; extreme apex slightly bent and it ends abruptly (not rounded) (Figs 228–229).

Female: Pro- and mesotarsus slender. Apical ventrite as in Fig. 32.

##### Distribution.

The species has been described from Saudi Arabia. African records include Libya and Egypt (Zalat et al. 2000). Thus far we have not seen any specimens from Africa, but a few from Arabian Peninsula (Fig. 529).

##### Collecting circumstances.

The habitat of *Laccophilus sordidus* is briefly described in Zalat et al. (2000) as follows “The species occurs in shallow water pools with gravel bottom and sparse vegetation, the water either being fresh or brackish. Considered rare and occurs in Eastern desert of Egypt in August”.

### Species group 4 (*Laccophilus alluaudi* group)

**Diagnosis.** Medium sized to large species; length of body 3.3–5.8 mm, width 1.8–2.9 mm.

Shape of body, oval-oblong to oblong; body dorso-ventrally flattened (Fig. 393). Body dorsally, with distinct colour pattern. Elytra exhibit somewhat broad, dark, longitudinal markings, which in most species fade away before reaching humeral region. Pale stripes between dark markings in most species shaped as small pearls on a narrow string (Fig. 391). One species exhibits hollow, dark elytral markings, i.e. dark, longitudinal marking encloses a narrow, pale area (Fig. 396). Body microsculpture double; two different size-classes can be recognized. Large meshes often in part reduced. One species with meshes in part longitudinally extended.

Prosternal process rather slender, moderately backwards extended, apically pointed. One species with comparatively short prosternal process. Apical ventrite not distinctly modified, lack asymmetric knob (Fig. 33). No stridulation apparatus on metacoxal plates. Metacoxal process not extended posteriorly (Fig. 6). One paramere simple, apically not distinctly modified or enlarged (Fig. 232). One species with slightly modified paramere. Penis slender, lateral aspect, evenly curved or basally angled and quite straight. Appearance of penis quite simple and delicate, lack considerable modifications (e.g. Figs 230–231).

**Species composition and distribution.** Six species are recognized in this species group, all of which occur on Madagascar or on nearby islands.

#### Key to species (males only)

**Table:** 

1	Large species, body length 5.0–5.8 mm; dark elytral, longitudinal markings hollow (narrow, pale marking enclosed in dark marking) (Fig. 396)	***Laccophilus seyrigi*** (p. 46)
–	Small to medium sized species, body length 3.3–4.2 mm; dark elytral marking entirely dark (no enclosed medial pale marking in dark marking) (Fig. 391)	**2**
2	Elytral dark markings complete or almost complete, reach humeral region (Fig. 391)	***Laccophilus comes*** (p. 37)
–	Elytral dark markings fade away before humeral region (Fig. 393)	**3**
3.	Penis, lateral aspect, evenly curved from base to apex (Fig. 273); penis, dorsal aspect, near base on right-hand side with a distinct enlargement (Fig. 238)	***Laccophilus tigrinus*** (p. 43)
-	Penis, lateral aspect, close to base angled (Fig. 230); penis, dorsal aspect, lacks latero-basal enlargement (Fig. 231)	**4**
4.	Large species, body length 4.1–4.2 mm; penis as in Fig. 242–243	***Laccophilus pseustes*** (p. 44)
-	Small species, body length 3.4-3.9 mm; penis different	**5**
5.	Penis, dorsal aspect, from angle to apex almost evenly broad and almost straight (Fig. 236)	***Laccophilus furthi*** (p. 42)
-	Penis, dorsal aspect, from angle to apex broad to narrow, strongly twisted (Fig. 234)	***Laccophilus alluaudi*** (p. 39)

#### Laccophilus comes

Taxon classificationAnimaliaColeopteraDytiscidae

Guignot, 1955

Figs 33–34 , 230–232 , 391 , 530

Laccophilus comes Guignot 1955f: 141 (original description, faunistics); Rocchi 1991: 86 (faunistics, list); Nilsson 2001: 242 (catalogue, faunistics); Nilsson 2015: 210 (catalogue, faunistics).

##### Type locality.

Madagascar, Tampolo.

##### Type material, studied

(2 exs.). Holotype: male: “Madagascar Tampolo VIII. 1949 / male symbol / Type” (MNHN). – Allotype (= Paratype), female: “Madagascar Tampolo VIII. 1949 / female symbol / Allotype” (1 ex. MNHN; habitus in Fig. 391).

##### Additional material studied

(116 exs.): Madagascar: “Andasibe 11.12. 2004 Lat -18.943 Lon 48.4063, Balke & Monaghan / DNA Voucher BMNH <670655> MSL008:B01 / *Laccophilus comes* Bergsten det.” (1 ex. NHRS); same data but “BMNH<670654>” (1 ex. NHRS); “Toam, Ambatondrazaka, Zahamane, Zahamena NP river, P60BI04: N: E: m, 29.12. 2006 leg. Isambert et al / DNA Voucher BMNH <830741> MSL399:F2 / *Laccophilus comes* Bergsten det.” (1 ex. NHRS); same data but “BMNH <830743> MSL399:F4” (1 ex. NHRS); “Toam, Ambatondrazaka, Zahamane, Zahamena NP stream, P60BI15: N: -17.52 E: 48.721: 1075m / m, 29.12. 2006 leg. Isambert et al / DNA Voucher BMNH <830737> MSL399:E10 / *Laccophilus comes* Bergsten det.” (1 ex. NHRS); same data but “Analamaintsoa 3^rd^ stream before Camp 1, stream of pools almost dry P60BI08, 30.12. 2006 N-17,50500, E48,72450, 1054 m Isambert et al (1 ex. NHRS); same data but “BMNH <830748> MSL399:F)/P60BI12: N: -17.517 E: 48.72: 1075 m, 31.12. 2006” (1 ex. NHRS); same data but “Zahamena NP 1^st^ stream btw. Camp 1 and 2, stream of pools, 31.12. 2006, N-17,51733, 48,72067, 1075 m” (85 exx. NHRS, 2 exs. MZH); same data but “Analamaintsoa Forest 4^th^ stream btw Camp 1 and 2, P6OBI15, 31.12. 2006, N-17,52050, E48,721337, 1075 m” (14 exx. NHRS); “IF Anadiana: Sahamalaotra Ranomafana NP: small stream P27MD31, N -21.2359 E: 47.3963, alt. 1123 m, 6.12.2004 leg. Balke et al / BMNH(E) <794196> DNA Voucher” (1 ex. NHRS); same but” BMNH(E) <794198>” (1 ex. NHRS); same but” BMNH(E) <794197>” (1 ex. NHRS); Mahajanga melaky, Tsingy de Bemaraha NP, S19.03419, E044.77499, 41 m.a.o., 15.12. 2009, Water net, field, Bergsten et al. / 000000464 NHRS-JLKB” (1 ex. NHRS); same but “S19.03572, E044.77507, 66 m.a.o., 15.12. 2009 / 000000467 NHRS/JLKB” (1 ex. NHRS); same but “S18.75643, E044.71398, 119 m.a.o., 17.12. 2009 / 000000463 NHRS/JLKB” (1 ex. NHRS); “Ampasimpotsy Moramanga Antsabe 11.12. 2004 N-18,94300, E48,40630, 979 m, Balke et al” (2 exx. NHRS).

##### Diagnosis.

*Laccophilus comes* forms together with *Laccophilus alluaudi*, *Laccophilus tigrinus*, *Laccophilus pseustes* and *Laccophilus furthi* a distinct group characterized by similar colour pattern of body and male genitalia. *Laccophilus comes*, *Laccophilus furthi* and *Laccophilus alluadi* are smaller than the other species in the group. The three species are separated by small differences in shape of penis; see diagnosis of *Laccophilus alluaudi* on p. 41 and *Laccophilus furthi* on p. 42.

##### Description.

Body length 3.3–3.9 mm, width 1.8–2.2 mm. Dorsal, colour pattern distinct and uniform (Fig. 391).

Head: Pale ferrugineous. At eyes with fine, irregularly distributed punctures. Rather shiny, although microsculptured. Reticulation double; coarse meshes only slightly stronger developed than fine meshes. Coarse meshes, when discernible, contain 2-3 fine meshes. In part, mesh categories cannot be distinguished.

Pronotum: Pale ferrugineous. Impunctate, except anteriorly, with scattered, fine punctures. Rather shiny, although finely microsculptured. Reticulation double. Coarse meshes only slightly more strongly developed than fine meshes; contain when discernible 3-4 fine meshes.

Elytra: Pale ferrugineous, with distinct, blackish ferrugineous to dark ferrugineous, longitudinal markings (Fig. 391). In a few specimens dark, longitudinal markings close to humeral region strongly reduced as in *Laccophilus alluaudi*. Posteriorly with rather fine, scattered punctation. Submat, distinctly and densely microsculptured. In part, reticulation double. Two kinds of reticulation clearly visible at scutellar region; posteriorly and laterally meshes of reticulation approximately one kind.

Ventral aspect: Metathorax and -coxal plates dark ferrugineous to ferrugineous; otherwise pale ferrugineous. Very fine, sparse punctures discernible on metacoxal plates and abdomen. Slightly mat due to very fine and hardly discernible microsculpture. Prosternal process rather slender, apex pointed but not strongly extended backwards. Abdomen with fine, sparse, curved striae. Apical ventrite not distinctly modified (Fig. 33).

Legs: Pro- and mesotarsus slender, somewhat extended; provided with suckers.

Male genitalia: Penis in lateral aspect medially, straight for a long distance, apex slightly bent; in dorsal aspect, penis from middle, strongly bent right (Figs 230–232).

Female: Pro- and mesotarsus slender. Apex of apical ventrite more angular than in male (Fig. 34).

##### Distribution.

Madagascar (Fig. 530).

##### Collecting circumstances.

Label data indicate that *Laccophilus comes* has been sampled in various sized, running waters as streams and rivers.

#### Laccophilus alluaudi

Taxon classificationAnimaliaColeopteraDytiscidae

Régimbart, 1900

Figs 35–36 , 233–234 , 392 , 531

Laccophilus alluaudi Régimbart 1900: 373 (original description, faunistics); Zimmermann 1920a:16 (catalogue); Guignot 1955d: 67 (discussion); Guignot 1955f: 141 (faunistics, discussion); Guignot 1959a: 544, 548 (description, faunistics); Guignot 1959c: 76, 77, 78, 79 (discussion, faunistics); Guignot 1959e: 72 (faunistics); Guignot 1961a: 931 (faunistics); Bertrand and Legros 1971: 244 (faunistics, biology); Wewalka 1980: 724, 726 (faunistics, discussion); Rocchi 1991: 79, 86 (faunistics); Nilsson 2001: 240 (catalogue, faunistics); Pederzani and Rocchi 2009: 95 (faunistics, list); Nilsson 2015: 208 (catalogue, faunistics).

##### Type locality.

Madagascar: Diego Suarez.

##### Type material, studied

(5 exs). Lectotype (by present designation): male: “Madagascar Diego-Suarez Ch. Alluaud 1893 / male symbol / Cotype / Museum Paris col. Guignot” (MNHN). – Paralectotypes: Same data as in lectotype, but two of the specimens with female symbol (4 exx. MNHN; habitus in Fig. 392).

##### Additional material studied

(189 exs.): **Madagascar**: “Forét d’Ambre Lat -12.4754 Lon 49.2173 coll. Balke & Monaghan, BMNH(E)670568_MSL007: B10, 19.11.04 / *Laccophilus alluaudi* Régb. det. Bergsten” (1 ex. NHRS); same data but “BMNH(E)670572_MSL007: CO2” (1 ex. NHRS); “Antsiranana II Mt d’Ambre, Grande cascade stream parallel to GC in deep very steep gorge, mostly isolated pools 17.11. 2004, N: -12,49920 E: 49,17600, 800 m Balke et al” (29 exs. NHRS, 4 exx. MZH); “Antsiranana II Foret d’Ambre, small water hole in dry streambed, gardenland at edge of dry forest 19.11. 2004 N-12,47540, E49,21730, 545m leg. Balke” (2 exs. NHRS); “Antsiranana 1 Mtd French Streampool 12.11. 2004 N-12,33360, E49,35350, 171 m, leg Balke et al” (12 exs. NHRS); “Antsiranana 1 Mt.d’Ambre 16.11. 2004, N-12,52830, E49,17253, 1020 m Balke et al.” (1 ex. NHRS); “Toli, NW Ft Dauphin, forest, watersource, P54E: N: E:: m 19.5. 2006 Bergsten et al. / *Laccophilus alluaudi* det. Bergsten/BMNH(E): <?94192> DNA voucher / *Laccophilus alluaudi* det. Bergsten” (1 ex. NHRS); “Toli, NW Ft. Dauphin, creek with gravel, stones and sand in rainforest along the creek, in small water holes and (*Madaglymbus*) in waterpool on a large rock with wood and leaves 19.5. 2006 N-24°45.583, E46°51.821, 300 m Bergsten et al.” (2 exs. NHRS); “Ambilobe, Anjiabe Ambony Antsabe: Galoko Mts, hygropetric cascade; alt. 50 m, P2506M N -13.6093 E 48.7212, 23.11. 2004 leg. Monaghan, Andriamparany, Balke / BMNH(E) <794160> DNA voucher / *Laccophilus alluaudi* det. Bergsten” (1 ex. NHRS); “Anjiabe Ambony, Ambilobe Antsabe stairways-like cascade with vertical steps, exposed, extremely hot day 23.11. 2004 N-13,60930, E48,72120, 303 m Balke et al.” (17 exx. NHRS); “Ambilobe, Anjiabe Ambony Antsabe: waterhole in streambed on clearing: 50 m, P25MD11 N - 13.648 E 48.721, 21.11. 2004, leg. Balke et al/BMNH(E) <794189> DNA voucher / *Laccophilus alluaudi* det. Bergsten” (1 ex. NHRS); “Toli, Taolanaro; Isaky Ivondro, Foret Manangotry, running water, P67B: N: -24.859: E: 46.862: 310 m, 9.4. 2007 leg. Ranarilalatiana et al / DNA voucher BMNH <830767> MSL399:H4 / *Laccophilus alluaudi* det. Bergsten” (1 ex. NHRS); “Ants, Sambava, Marojejy, Marojejy NP: Forest stream P57BI01: N: -14.437: E: 49.773: 464 m, 6.12. 2006 leg. Isambert et al. / DNA voucher BMNH <830690> MSL399:A11 / *Laccophilus alluaudi* det. Bergsten” (1 ex. NHRS); “Toam, Ambatondrazaka, Zahamena; Zahamena NP: Stream P60BI08 N: -17.505: E: 48.724: 1054 m, 30.12.2006 leg. Isambert et al. / DNA voucher BMNH <830696> MSL399:B5 / *Laccophilus alluaudi* det. Bergsten” (1 ex. NHRS); same data but “P60BI06 N: -17.508: E: 48.724: 1068 m” and “BMNH <830701> MSL399:B10” (1 ex. NHRS); same data but “Zahamena NP, Analamaintsoa 1^st^ stream before Camp 1 within a few m from the GPS point P60BI06, 30.12. 2006 N-17,50617, E48,72400, 1068 m” (4 exs. NHRS); “TOAM Ambatondrazaka Zahamena NP on the way to camp 2 to Fenerive Est “Route des contrebandiers” P60BI29 02.1. 2007, N: -17,54167 E: 48,72183, 1322m leg. Isambert et al” (37 exs. NHRS); same data but “Analamaintsoa, 2nd stream btw Camp 1 and 2, stream of pools 31.12. 2006 N-17,51850, E48,72217, 1075 m” (4 exs. NHRS); same data but “Analamaintsoa, 3^rd^ stream before Camp 1, stream of pools, almost dry P60BI08, 30.12. 2006, N-17,50500, E48,72450, 1054 m Isambert et al” (10 exx. NHRS); “Montagne des Francais, Lat. -12.3336 Lon. 49.3535 leg. Balke & Otke, BMNH(E)_671210 MSL014; 1/1/1904 / DNA voucher BMNH <671210> MSL014: H02/*Laccophilus alluaudi* det. Bergsten” (1 ex. NHRS); “Saratanana, leg. Lees & Ranaivosolo MNH(E)_672835_MSL028; 1/1/1904 /DNA voucher BMNH <672835> MSL028: B05 / *Laccophilus alluaudi* det. Bergsten” (1 ex. NHRS); “Antsabe Lat -13.6093 Lon 49.7212, Balke leg. BMNH(E) _670700_MSL008 23.9. 2004/1904/DNA voucher BMNH <670700> MSL008: E10/*Laccophilus alluaudi* det. Bergsten” (1 ex. NHRS); “Ants, Nosy Be: Lokobe R.N.1, 50 m, 2004/DNA voucher BMNH(E) <794171> / *Laccophilus alluaudi* det. Bergsten” (1 ex. NHRS); “R.N.I. Lokobe Nosy Be, 50 m, 15.12. 2004 Ravo” (1 ex. NHRS); “Mahajanga: Boeny Mahavavy Kinkony RS. S16.05648, E045.76371, 55 m.a.o., 5.12. 2009 water net, field, Bergsten et al. (19 exs. NHRS); same data, add: “000000465 NHRS-JLKB” (1 ex. NHRS); “Mahajanga: Melaky: Tsingy de Bemaraha NP, S19.03572, E044.77507, 66m m.a.o., 15.12. 2009, water net, field, Bergsten et al.” (12 exs. NHRS); same data but “S18.75643, E044.71398, 119 m.a.o., 17.12. 2009” (2 exs. NHRS); same data but “S19.03419, E044.77499, 41 m.a.o., 15.12. 2009” (2 exs. NHRS); same data but “S18.75724, E044.71239, 72 m.a.o., 17.12. 2009” (3 exx. NHRS); same data, add: “000000468 NHRS-JLKB” (1 ex. NHRS); same data but “S19.14114, E044.81245, 45 m.a.o., 14.12. 2009/000000466 NHRS-JLKB” (1 ex.NHRS); “Prov. Antsirarana, P.N. Montagne d’Ambre, elev. 960 m 26–29.1. 2001/N -12*30'52” E 49*10'53” leg. Irwin & al, malaise trap” (1 ex. CAS); “Prov. Antsirarana, P.N. Montagne d’Ambre, elev. 1125 m 30.5.-6.6. 2001/N -12°31'13” E 49°10'45” leg. Irwin & Hala, malaise trap” (1 ex. CAS); “Prov. Antsirarana, P.N. Montagne d’Ambre, elev. 1125 m 21-26.4. 2001/N -12°31'13” E 49°10'45” leg. Irwin & Hala, malaise trap” (1 ex. CAS); “Prov. Antsirarana, Sakalava beach, dwarf littoral forest 10 m, 13–20.8. 2001/N -12°15'46” E 49°23'51” Irwin & Hala leg., malaise trap – across sandy trail” (1 ex. CAS); “Andjamangirana (Majunga) 19.10. 2001/stream in dry forest, upstream. Rice field area (road to Tsaratanana) 200 m a.s.l., 30,8 °C, 0.008 mS/cm/Gerecke & Goldschmidt leg.” (1 ex. BMNH, 1 ex. MZH); “Anjozorobe (Antananarivo) Ravoandrina Riv. Ampanakamonty 21.7.2001 / 1280 m asl, 12,8 °C, 0,078 mS/cm/Gerecke & Goldschmidt leg.” (1 ex. BMNH); “SE-Mad. Rés. Nat. Integr. de Andohahela (NW Ft. Dauphin) Parcelle 1 (versante E) – 300 m foresta pluviale 26.5. 1991/Bartolozzi, Taiti, Raharimina leg. / *Laccophilus alluaudi* Rég. det. Rocchi 1991”(2 exs. CSR); “E-Mad. Ampamoho nr Andilamena 1200-1300 m asl, 18-20.1. 1995 Dunay & Janak” (1 ex. MZH); “Fiananrantsoa, Mania River S Ilaka, 900 m 27 rd km NNW Ambositra 23.10. 2001 Schuh leg.” (1 ex. NMW); “Foret de Fito, ex. coll. Dr. Breuning” (1 ex. MRAC); “Prov. de Tamatave, Foret de Perinet 17.7. 1970 Pederzani / *Laccophilus lateralis* Sharp det. Pederzani” (1 ex. CSR; determination uncertain).

##### Diagnosis.

*Laccophilus alluaudi* forms together with *Laccophilus comes*, *Laccophilus tigrinus*, *Laccophilus pseustes* and *Laccophilus furthi* a distinct group of species characterized by quite similar colour pattern of body and male genitalia exhibiting same ground plan. Penis of *Laccophilus alluaudi*, *Laccophilus comes* and *Laccophilus furthi* is not evenly curved in lateral view as in *Laccophilus tigrinus*, but angled. Longitudinal markings of *Laccophilus comes* reach humeral region while in *Laccophilus alluaudi* and *Laccophilus furthi* corresponding markings fade away before reaching humeral region. Finally, penis (dorsal aspect) in *Laccophilus alluaudi* is strongly twisted, while almost straight in *Laccophilus furthi*. Furthermore *Laccophilus comes* penis is in lateral view medially slightly depressed and immediately after angle towards apex there is a minute, sharp knob which is lacking in *Laccophilus alluaudi*.

##### Description.

(See description of *Laccophilus comes*; only diagnostically important differences noted): Body length 3.4–3.9 mm, width 1.9–2.1 mm. Dorsal, colour pattern (Fig. 392); slightly less pronounced in comparison with *Laccophilus comes* (Fig. 390).

Elytra: Longitudinal markings brownish to ferrugineous; less pronounced in comparison with material of *Laccophilus comes*; especially humeral region with reduced dark markings (Fig. 392).

Ventral aspect: Apical ventrite as in Fig. 35.

Male genitalia: Penis in lateral aspect from base region, almost straight to extreme apex, which is slightly bent upwards; in dorsal aspect, penis bent at right but less so than in *Laccophilus comes* (Figs 233–234).

Female apical ventrite as in Fig. 34.

##### Distribution.

Madagascar (Fig. 531). Also reported from the Comoros (e.g. Guignot 1958b).

##### Collecting circumstances.

Label data indicate that *Laccophilus alluaudi* occurs in both standing and running waters. Collected in a creek with gravel, stones and sand in rainforest along the creek, in small water holes and together with *Madaglymbus* in water pool on a large rock with wood and leaves.

#### Laccophilus furthi sp. n.

Taxon classificationAnimaliaColeopteraDytiscidae

http://zoobank.org/8450329E-15FC-47FD-BBC9-F1FEAC01EA45

Figs 37 , 235–236 , 393 , 532

##### Type locality.

Madagascar: Prov. Fianarantsoa, 7 km West of Ranomafana.

##### Type material

(2 exs.): Holotype: male: “Madagascar: Prov. Fianarantsoa, 7 km W Ranomafana, 1100 m 8-21. October 1988 W.E. Steiner / From stream with mossy rocks and sandy bottom, montane rainforest” (USNM; habitus in Fig. 393). – Paratype: “Madagascar 19–22.1. 2000 Toamasina distr. (Périnet) Analamazaotra S Andasibe 18°56'09"S, 48°24'48"E, O. Hovorka leg., black light” (1 ex. NMPC).

##### Diagnosis.

*Laccophilus furthi* resembles most of *Laccophilus alluaudi*, *Laccophilus comes* and *Laccophilus tigrinus*. Distinguishable by study of the penis, the shape of which is almost straight and comparatively broad in *Laccophilus furthi*, while sinuate and less evenly broad in the three resembling species mentioned above. Vide diagnosis of *Laccophilus alluaudi* (p. 41).

##### Description.

Body length 3.7–3.8 mm, width 2.0 mm. Dorsal, colour pattern of body as in Fig. 393. Dark, longitudinal lines on elytra gradually fade away towards base of elytra.

Head: Pale ferrugineous. Submat to rather shiny, finely microsculptured. Reticulation double, but size classes of meshes difficult to distinguish. In part meshes mixed and sculpture appears irregular, consisting of variable shaped meshes. Impunctate, except at eyes; with fine, irregular punctures, which extend a short distance towards middle of head-disc.

Pronotum: Pale ferrugineous; no distinct colour pattern. Submat to rather shiny, finely microsculptured. Reticulation double but size classes of meshes difficult to distinguish. In part meshes mixed, and sculpture appears irregular, consisting of variable shaped meshes. Impunctate, except frontally and laterally; with very fine, scattered punctures.

Elytra: Pale ferrugineous, with dark ferrugineous to brownish, longitudinal areas, which anteriorly, gradually fade away in the holotype while quite distinct in paratype (Fig. 393). Rather shiny, although finely and densely microsculptured. Reticulation double, but large meshes almost absent because strongly reduced (only rudiments discernible). Very fine, irregular punctures form a somewhat vague, discal row. Dorsolateral and lateral rows indistinct; indicated by some scattered fine punctures. Pre-apical row consists of fine, slightly impressed punctures provided with fine hairs. In apical quarter of elytra fine punctures mixed and no separate rows discernible.

Ventral aspect: Pale ferrugineous, except metathorax and -coxal plates; blackish to dark ferrugineous. Shiny to rather shiny, microsculpture almost absent. Only very fine rudimentary microsculpture can sporadically be discerned. Abdomen with very fine, curved striae. Impunctate, except apical ventrite; with some fine, scattered punctures; shape of ventrite almost symmetric (Fig. 35). Metacoxal plates with 3-4, very fine, in part reduced, transverse furrows. Lateral impression on metacoxal plate moderate but clearly discernible. Prosternal process rather slender, posteriorly moderately extended, apically pointed.

Legs: Pale ferrugineous. Pro- and mesotarsus slightly enlarged and extended, with suckers.

Male genitalia: Penis exhibits few modifications, being almost straight both in lateral and dorsal aspects (Figs 235–236).

Female: Unknown.

##### Etymology.

The name is a noun in its genitive form based on the name of Dr. David Furth, Washington D.C., USA, who kindly assured the loan of large African *Laccophilus* collections for this study, deposited in USNM.

##### Distribution.

Madagascar (Fig. 532).

##### Collecting circumstances.

Collecting label informs that *Laccophilus furthi* has been “collected from stream with mossy rocks and sandy bottom in montane rainforest”. The single paratype was collected by black light.

#### Laccophilus tigrinus

Taxon classificationAnimaliaColeopteraDytiscidae

Guignot, 1959

Figs 38–39 , 237–239 , 394 , 532

Laccophilus tigrinus Guignot 1959c: 76, 78, 79 (original description, faunistics); Guignot 1961a: 931 (faunistics); Wewalka 1980: 724, 726 (faunistics); Nilsson 2001: 251 (catalogue, faunistics); Nilsson 2015: 218 (catalogue, faunistics).

##### Type locality.

Comoro Islands: Anjouan, Foret de M’Remani.

##### Type material, studied

(5 exs.). Holotype: male: “Type / F. Guignot det. 1955 *Laccophilus tigrinus* sp. n. Type, male symbol” (MNHN). – Paratypes: Same data as holotype but labelled “Paratype” (1 ex. MNHN); “Anjouan Fet de M’Remani X-1953 (Millot) / male symbol / Paratype” (1 ex. MNHN; habitus in Fig. 394); “Moheli Foret de Fomboni 600 m 2eme torrent 6.54 (JM) / male symbol / Paratype” (1 ex. MNHN); Same data but provided with label “Coll. Guignot” and a peculiar label with the following text: “R. Mouchamps det. 63 *Laccophilus mohelicus* sp. n. paratype” (1 ex. MNHN).

##### Additional material studied

(5 exs.): **Comoro Islands**: “Grande Comore Nioumbadjou 9.8. 1981 R. Joqué”(2 exs. MRAC, 1 ex. MZH); “Moheli Foret de Fomboni 600 m 2eme torrent 6.54 (J.M.)” (2 exs. IRSNB).

##### Diagnosis.

*Laccophilus tigrinus* resembles most of all species of *Laccophilus comes*, *Laccophilus alluaudi* and *Laccophilus furthi*, but it is often slightly larger and penis in lateral view almost evenly curved and not angled. Additionally, penis of *Laccophilus tigrinus* on one side, provided with a latero-basal expansion which is absent in *Laccophilus comes*, *Laccophilus alluaudi* and *Laccophilus furthi*. Resembles also of *Laccophilus pseustes* but size of body smaller.

##### Description

(only diagnostically important differences to description *Laccophilus alluaudi* are recognized):

Body length 3.6–4.0 mm, width 2.1–2.3 mm. Dorsal, colour pattern of body distinct (Fig. 394).

Head: Posteriorly head often becomes gradually a little darker.

Pronotum: Frontally in middle with a vague, somewhat darker area.

Elytra: Very fine, sparse punctures form a discal, a dorsolateral and a lateral row of punctures discernible on each elytron. Pre-apical, lateral furrow rather shallow; punctate with fine hairs.

Ventral aspect: Metacoxal plates in part blackish. Apical ventrite as in Fig. 38.

Legs: Pro- and mesotarsus slightly enlarged and extended, provided with distinct suckers.

Male genitalia: Penis, in lateral aspect evenly curved towards apex, in dorsal aspect, slightly sinuate; basally provided with a distinct enlargement (Figs 237–239).

Female: Pro- and mesotarsus slender. Apical ventrite as in Fig. 39.

##### Distribution.

Comoro Islands (Fig. 532).

##### Collecting circumstances.

Unknown.

#### Laccophilus pseustes

Taxon classificationAnimaliaColeopteraDytiscidae

Guignot, 1955

**Figures 40–46.**
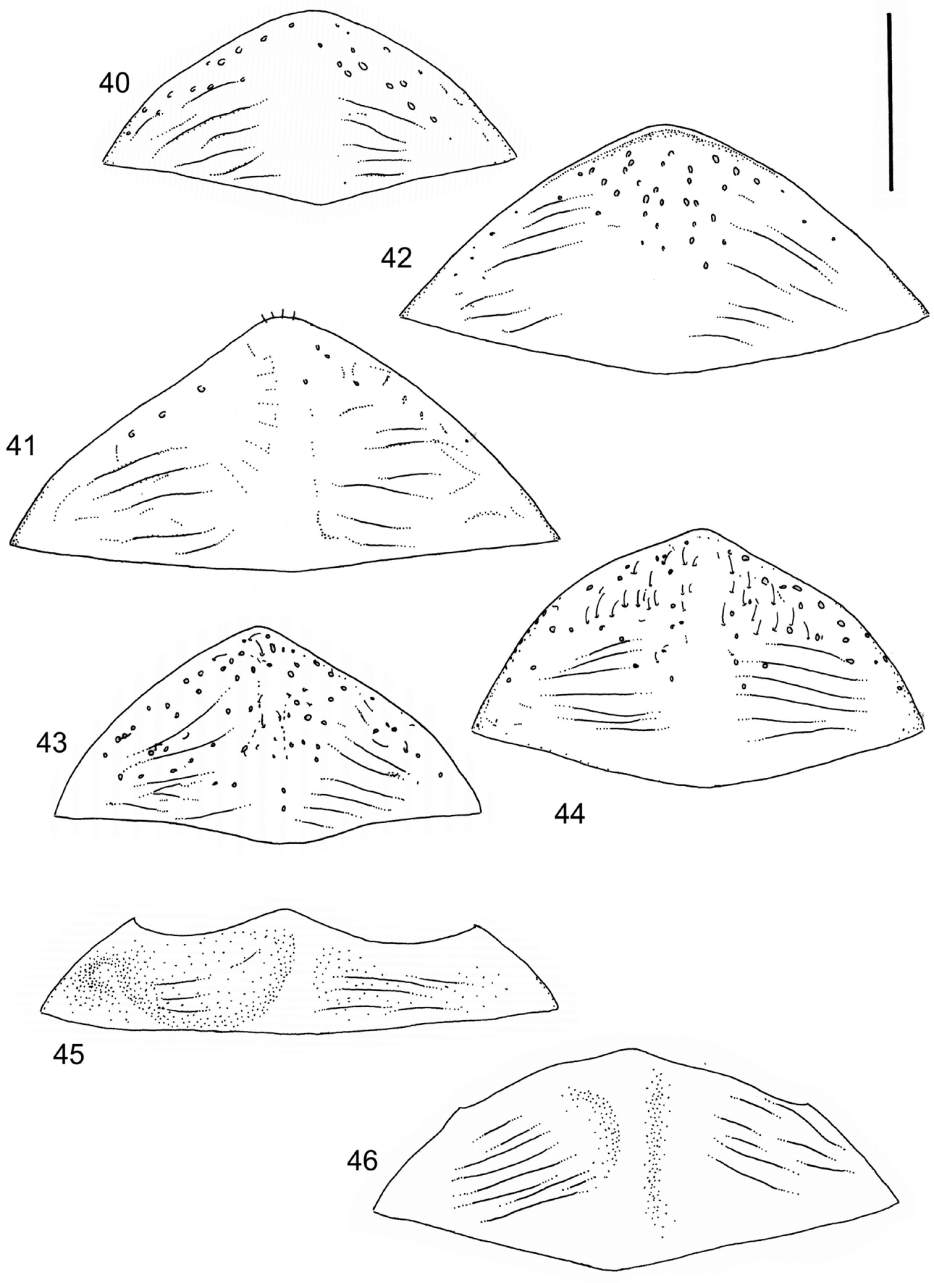
Apical ventrites. **40** *Laccophilus pseustes* male **41** *Laccophilus seyrigi* male, and **42** female **43** *Laccophilus isamberti* male, and **44** female **45** *Laccophilus pictipennis* male, and **46** female. Scale bar 0.5 mm.

Figs 40 , 240–241 , 395 , 532

Laccophilus pseustes Guignot 1955d: 67 (original description, faunistics); Rocchi 1991: 86 (faunistics, list); Nilsson 2001: 249 (catalogue, faunistics); Pederzani and Rocchi 2009: 95 (faunistics, list); Nilsson 2015: 216 (catalogue, faunistics).

##### Type locality.

Madagascar: Isalo sur Pandamus.

##### Type material, studied

(1 ex.). Holotype: male: “Isalo sur Pandamus, Inst. Sci. Madagascar VIII.48 RP / Type / Guignot det. 1955 *Laccophilus pseustes* Type, male symbol” (MNHN; habitus in Fig. 395).

##### Additional material studied

(23 exs.): **Madagascar**: “Fian: Isalo, source of piscine naturelle, waterhole, P41K: N -22.553: E: 45.368: 859 m 12.5. 2006 leg. Bergsten et al / BMNH(E) <794199> DNA voucher / *Laccophilus pseustes* det. Bergsten” (1 ex. NHRS); same data but “BMNH(E)<745062> DNA voucher ” (1 ex. NHRS); “Fian: Isalo, source of piscine naturelle, small water holes at beginning of stream P41K, 12.5. 2006 N-22°33.206, E45°22.089, 859 m, Bergsten et al.” (5 exs. NHRS); “Fian: Isalo, Canyon de Makis: River: P41E: N: -22.548: E: 45.408: 780 m, 11.5. 2006 leg. Bergsten et al / BMNH(E) <745068> DNA voucher / *Laccophilus pseustes* det. Bergsten” (1 ex. NHRS); “Fian: Isalo, Canyon de Makis, sandy bottom of river, with side pools and hygropetric sections at sides, wood in water, P41E 11.5. 2006 N-22°32.922, E45°24.064, 780 m Bergsten et al.” (6 exs. NHRS, 2 exs. MZH); “Fian, Isalo, Namaza R.: stagnant waterpool P41I: N: -22.539: E: 45.377: 794 m, 12.5. 2006 leg. Bergsten et al / BMNH(E) <745060> DNA voucher / *Laccophilus pseustes* Bergsten det.” (1 ex. NHRS); “Fian, Isalo: P41O: Trib. to Namaza R.: Waterhole, N: -22.543: E: 45.377, 842. 1624 m, 13.5.2006 leg. Isambert et al / DNA voucher BMNH(H) <831017> MSL 402:E2 / *Laccophilus pseustes* det. Bergsten” (1 ex. NHRS); same as but “DNA voucher BMNH(E) <831016> MSL402:E1” (1 ex. NHRS); same as but “DNA voucher BMNH(E) <831019> MSL402:E4” (1 ex. NHRS); same as but “DNA voucher BMNH(E) <831020> MSL402:E5” (1 ex. NHRS); “Fian: Isalo Namaza R. stagnant water pool with lots of woody debris and leaves 12.5. 2006, N-22°32.348, E45°22.626, 794 m, Bergsten et al. (2 exs. NHRS).

##### Diagnosis.

*Laccophilus pseustes* resembles most *Laccophilus alluaudi* and *Laccophilus comes* and also of some other species in this group but its body is generally clearly larger. Additional diagnostic features are found in the shape of the penis: In lateral aspect, penis long and narrow and extreme apex slightly curved upwards; in dorsal aspect, penis quite broad and somewhat sinuate with narrow slightly curved tip.

##### Description.

Body length 4.1–4.2 mm, width 2.3–2.4 mm. Elytral colour pattern slightly vague (Fig. 395).

Head: Pale ferrugineous. Rather shiny, finely microsculptured. Reticulation indistinctly double. Large meshes only slightly more strongly developed in comparison with small meshes. In part, small meshes reduced and hardly visible. Impunctate, except at eyes; with fine, scattered punctures; closely towards centre of head, there is an additional small group of fine punctures located in a small depression.

Pronotum: Pale ferrugineous. Submat, finely microsculptured. Reticulation double; large meshes contain 3–5 small meshes. Laterally and frontally, with fine, scattered punctures.

Elytra: Pale ferrugineous, with somewhat vague, dark ferrugineous to ferrugineous colour pattern (Fig. 395). Slightly mat, finely microsculptured; reticulation double, but small meshes distinct while large meshes strongly reduced and only in part discernible. Fine, scattered punctures form a somewhat irregular, discal row. Dorsolateral and lateral rows indistinct; simply indicated by few scattered punctures. Postero-laterally, with a pre-apical, pubescent furrow.

Ventral aspect: Pale ferrugineous to ferrugineous, no distinct colour pattern. Submat, very finely and in part indistinctly microsculptured. Abdomen with fine, curved striae. Metacoxal plates with some 8–9 transversely located, shallow furrows. Almost impunctate, except on apical ventrite; with a few scattered punctures. Apical ventrite symmetric and lacks lateral knob (Fig. 40). Prosternal process quite narrow, apex short, only slightly extended, apically pointed.

Legs: Pro- and mesotarsus somewhat enlarged, extended and provided with suckers. Hindlegs quite robust.

Male genitalia: Penis in dorsal aspect comparatively broad with narrow, slightly curved apex; in lateral aspect, penis quite slender and long with tip curved slightly upwards (Figs 240–241).

Female: Pro- and mesotarsus rather slender.

##### Distribution.

Madagascar (Fig. 532).

##### Collecting circumstances.

Mainly unknown. Label data simply indicate that the species has been collected in a river with sandy bottom, with side pools and hygropetric sections at sides, wood in water. Additionally, recorded in stagnant water pool with lots of woody debris and leaves.

#### Laccophilus seyrigi

Taxon classificationAnimaliaColeopteraDytiscidae

Guignot, 1937

Figs 41–42 , 242–244 , 396 , 532

Laccophilus seyrigi Guignot 1937: 140 (original description, faunistics); Guignot 1959a: 544, 545, 550 (description, faunistics); Rocchi 1991: 86 (faunistics, list); Nilsson 2001: 250: (catalogue, faunistics); Nilsson 2015: 217: (catalogue, faunistics).

##### Type locality.

Madagascar: Békily.

##### Type material, studied

(6 exs.). Holotype: male: “Madagascar Békily III 1936 – S / male symbol / Type” (MNHN). – Paratypes: Same data as holotype but “Paratype” (1 ex. MNHN; habitus in Fig. 396); Same data as holotype but “female symbol / Paratype” (3 exs. MNHN, 1 ex. IRSNB).

##### Diagnosis.

*Laccophilus seyrigi* forms together with *Laccophilus comes* and some other morphologically similar species an own group of species. *Laccophilus seyrigi* is, however, a deviating species in the group, and it is separated from the other species by clearly larger body size, by peculiar elytral colour pattern, by longitudinally extended meshes of microsculpture and by species-characteristic shape of penis; in dorsal aspect being long, slender and straight; in lateral aspect basally, with a distinct enlargement.

##### Description.

Body length 5.0–5.8 mm, width 2.8–2.9 mm. Dorsal, colour pattern of body rather distinct and stable; only minor variation exhibited (Fig. 396).

Head: Pale ferrugineous. At eyes with dense and fine punctures. Additionally with fine punctures in a short transverse impression located close to each eye. Submat, finely microsculptured. Reticulation double. Coarse meshes distinct; fine reticulation reduced, only in part discernible. Fine meshes extensively obliterated.

Pronotum: Pale ferrugineous, frontally in middle with distinct dark ferrugineous area; posteriorly in middle with a vague, bilobed ferrugineous to dark ferrugineous spot. Almost impunctate, except frontally and laterally with fine scattered punctures. Rather shiny, distinctly microsculptured. Reticulation double. Large meshes distinct; especially in middle meshes longitudinally extended. Fine meshes clearly discernible laterally; medially fine reticulation absent or almost totally obliterated.

Elytra: Pale ferrugineous, with distinct, dark ferrugineous markings (Fig. 396). Almost impunctate; discally and laterally with a few, fine punctures. Rather shiny, although distinctly microsculptured. Reticulation double. Coarse meshes distinct, in frontal half meshes longitudinally extended. Fine meshes frontally almost totally obliterated; in posterior half fine meshes clearly discernible. When discernible, coarse meshes contain 3–6 fine meshes.

Ventral aspect: Pale ferrugineous, laterally gradually darker, or with quite distinct, dark, lateral spots; dark ferrugineous to blackish. Abdomen pale ferrugineous. Ventrites latero-posteriorly with darker areas (dark ferrugineous to blackish). Apical ventrite pale except for latero-basally, with dark ferrugineous areas. Apical ventrite not modified (Fig. 41). Almost impunctate. Apical ventrite with fine punctures. Rather shiny, although very finely microsculptured. Microsculpture in part reduced, obliterated. Abdomen with fine striae. Metacoxal plates with 5–6 very fine, shallow furrows, which are almost transversely located. Prosternal process rather slender and comparatively short, apically pointed.

Legs: Pro- and mesotarsus rather slender and extended, with protruding suckers.

Male genitalia: Penis in dorsal aspect long, slender and straight; in lateral aspect basally, with a distinct enlargement (Figs 242–244).

Female: Pro- and mesotarsus slender, somewhat extended. Apical ventrite uniform (Fig. 42).

##### Distribution.

Madagascar (Fig. 532).

##### Collecting circumstances.

Unknown.

### Species group 5 (*Laccophilus isamberti* group)

**Diagnosis.** Medium sized species with body length 3.7–4.0 mm, width 2.2–2.4 mm.

Shape of body oval-oblong, body dorsoventrally flattened (Fig. 397). Dorsal colour pattern distinct and peculiar. Elytra with blackish to dark ferrugineous base from which comparatively broad blackish to dark ferrugineous, longitudinal lines start, leaving pale ferrugineous, somewhat vague lines between them. Apically dark lines become somewhat irregular (Fig. 397). Body microsculpture double, although division in two size-classes difficult. Large meshes obscure, extensively strongly reduced (only fragments of large meshes discernible), while small meshes rather distinct.

Prosternal process quite narrow, apex only moderately extended, apex pointed. Apical ventrite simple, not distinctly modified; no asymmetrical knob located on one side of ventrite (Fig. 44). No stridulatory apparatus discernible on metacoxal plates. Metacoxal process posteriorly expanded (not truncate as in other African *Laccophilus*) (Fig. 7).

Paramere quite simple but clearly enlarged in apical half (Fig. 246). Penis, lateral aspect, in apical half evenly curved, exhibits no distinct modifications (Fig. 245).

**Species composition and distribution.** One species recognized in this species group. Only recorded from Madagascar.

#### Laccophilus isamberti sp. n.

Taxon classificationAnimaliaColeopteraDytiscidae

http://zoobank.org/DA915883-FB68-4C10-AE9B-A579CFA7CA21

Figs 7 , 43–44 , 245–246 , 397 , 533

##### Type locality.

Madagascar: Zahamena N.P., Ambatondrazaka. (N: -17,50800 E: 48,72283).

##### Type material studied

(23 exs.): Holotype: male: “MAD TOAM: Ambatondrazaka Zahamena: Zahamena N.P. close to Camp site 1 Manambota River, on the Rocks. PB60BI01: N: -17,50800 E: 48,72283: 943 m 28.XII. 2006 Leg. Isambert et. al. / *Laccophilus* sp.n. *lateralis* gr. Det. J. Bergsten. 2008” (NHRS, habitus in Fig. 397). – Paratypes: Same data as holotype (4 exs. NHRS, 2 exs. MZH); same data as holotype and “DNA VOUCHER BMNH(E) <834433> MSL:430:G02” (1 ex. BMNH); same data as holotype, but ”<834432> MSL:430:G01” (1 ex. BMNH); “MAD TOAM: Ambatondrazaka Zahamena: Zahamena N.P. Analamaintsoa Forest between Camp Site 1 and Camp Site 2 Manambota River. PB60BI02: N: -17,50750 E: 48,72250: 1071 m 29.XII. 2006 Leg. Isambert et al. / *Laccophilus* sp.n. *lateralis* gr. Det. J. Bergsten 2008/DNA VOUCHER BMNH(E) <834434-7> MSL:430:G03-6” (3 exs. BMNH, 1 ex. MZH); same data but not vouchers (1 ex. NHRS, 1 ex. NMW); same data but”P60BI04” (2 exs. NHRS, 1 ex. NMW); “MAD TOAM: Ambatondrazaka Zahamena: Zahamena N.P. Analamaintsoa Forest Manambota Rv, 500 m between Camp 1 & Camp 2 PB60BI11 30.XII. 2006 N: -17,50717 E: 48,72400 leg. Isambert et al. / *Laccophilus* sp.n. *lateralis* gr. Det. J. Bergsten, 2008/DNA VOUCHER BMNH(E) <834438> MSL:430:G07” (1 ex. BMNH); same data but not vouchers (2 exs. NHRS); “MAD TOAM: Ambatondrazaka Zahamena: Zahamena N.P. Analamaintsoa Forest 5th stream between Camp 1 and Camp 2 PB60BI16 31.XII. 2006 N: -17,52183 E: 48,72067 1092 m leg. Isambert et al. / *Laccophilus* sp.n. *lateralis* gr. Det. J. Bergsten, 2008/DNA VOUCHER BMNH(E) <834439> MSL:430:G08” (1 ex. BMNH).

##### Diagnosis.

A deviating species, which on the basis of external appearance and shape of penis may be closely related to species group 4 (*Laccophilus alluaudi*). *Laccophilus isamberti*, however, exhibits peculiar modification on metacoxal process, being posteriorly expanded (Fig. 7). This feature is lacking in all other African *Laccophilus* species and accordingly the location within the genus *Laccophilus* can also be discussed. Further study is definitely needed to establish the status of the species *Laccophilus isamberti*.

##### Description.

Body length 3.7-4.0 mm, width 2.2-2.4 mm. Dorsal colour pattern of body (Fig. 397); minor variation observed in width of elytral, longitudinal markings.

Head: Posteriorly dark brown; anteriorly head becomes gradually paler. Rather shiny, although finely microsculptured. Reticulation indistinctly double; large meshes generally quite distinct while small meshes in part indistinct. When discernible, large meshes contain 2-4 small meshes. At eyes with fine, irregular punctures. Area with punctures extended from eyes towards middle of head but they don’t meet medially.

Pronotum: Blackish ferrugineous to dark ferrugineous, laterally broadly paler; ferrugineous to pale ferrugineous. Slightly mat, finely microsculptured. Reticulation indistinctly double. Small meshes distinct; large meshes strongly reduced and almost absent, only slightly stronger developed than small meshes. Large meshes, when discernible, contain 2-4 small meshes. Impunctate, except at margins with fine, irregular punctures (mediobasally, punctures also absent).

Elytra: Blackish to dark ferrugineous, with pale ferrugineous, longitudinal markings. Posteriorly markings undulate (Fig. 397). Width of longitudinal markings slightly variable. Slightly mat, finely microsculptured. Reticulation double, but large meshes strongly reduced and almost absent. Fine, scattered punctures form a discal row. Dorsolateral and lateral rows indicated by a few scattered punctures. Lateral, pre-apical furrow shallow, rather finely pubescent.

Ventral aspect: Blackish to dark ferrugineous; without distinct colour pattern. Rather shiny, very finely microsculptured. Reticulation in part absent. Abdomen basally with fine, curved striae. Almost impunctate; ventrites with fine punctures. Apical ventrite as in Fig. 43. Prosternal process quite narrow, apex only moderately extended, apex pointed, Metacoxal plates in anterior half with four outwards curved, distinct furrows; in posterior half furrows strongly reduced. Metacoxal process posteriorly expanded (Fig. 7).

Legs: Pro- and mesotarsus slightly enlarged, with suckers.

Male genitalia: Shape of penis (Figs 245–246) resembles some of the species located in species group 4 and especially the species *Laccophilus pictipennis*, placed in an own species group 6.

Female: Pro- and mesotarsus slender. Apical ventrite as in Fig. 44.

##### Etymology.

The name is a noun in its genitive form based on the name of Dr. Benjamin Isambert, Toulouse, France, who collected the type material during his PhD studies.

##### Distribution.

Madagascar, so far only known from Zahamena National Park (Fig. 533).

##### Collecting circumstances.

This is a lotic species occurring in rivers and streams. The known localities are at an altitude of 1000-1100 m.

### Species group 6 (*Laccophilus pictipennis* group)

**Diagnosis.** Large species with body length 4.4–4.7 mm and width 2.5–2.8 mm.

Shape of body oval-oblong; body dorsoventrally flattened (Fig. 398). Dorsal side with distinct colour pattern; pale ferrugineous to ferrugineous with dark ferrugineous, often somewhat vague patches (Fig. 399). Body microsculpture double; both small and large meshes exhibited. Small meshes extensively reduced and weakly developed; sometimes totally missing.

Prosternal process moderately slender, posteriorly not strongly extended, apex pointed. Apical ventrite with posterior end excavated on both sides and medially ventrite moderately produced backwards; lacks asymmetric knob on one side (Fig. 45). No stridulatory files on metacoxal plates. Metacoxal process not extended posteriorly (Fig. 6).

Paramere simple, elongate, apically not enlarged or modified (Fig. 248). Penis rather slender, clearly curved and apex not distinctly modified (Fig. 247).

**Species composition and distribution**: One species recognized in this species group. In Africa it occurs in north-eastern part; also recorded from Arabian Peninsula.

#### Laccophilus pictipennis

Taxon classificationAnimaliaColeopteraDytiscidae

Sharp, 1882

Figs 45–46 , 247–248 , 398–399 , 534

Laccophilus pictipennis Sharp 1882: 305 (original description, faunistics); v. d. Branden 1885: 23 (catalogue, faunistics); Régimbart 1895: 131, 132 (description, faunistics, discussion); Zimmermann 1920a: 24 (catalogue); Zimmermann 1930: 21, 23 (description, faunistics); Balfour-Browne 1951: 193 (discussion, faunistics); Guignot 1952d: 4, 5 (discussion); Guignot 1959a: 533, 536 description, faunistics); Brancucci 1979: 159 (faunistics, description, discussion); Brancucci 1983b:274, 394, 416 (description, faunistics; lectotype designation); Rocchi 1984: 447 (faunistics.); Nilsson and Persson 1993: 79, 94 (faunistics, discussion); Nilsson 2001: 248 (catalogue, faunistics); Nilsson 2003: 77 (faunistics, list); Angus 2003: 16 (synonymy, discussion); Hajek and Reiter 2014: (faunistics, biology); Nilsson 2015: 168 (catalogue, faunistics).
Laccophilus wehnckei Sharp 1882: 306 (original description, faunistics); v. d. Branden 1885: 24 (catalogue, faunistics); Régimbart 1895: 131 (description, faunistics, discussion); Zimmermann1920a: 28 (catalogue); Guignot 1943: 99 (description, faunistics); Guignot 1946c: 270, 273, 277, 312, 315 (*Laccophilus wehnchei* Sharp, misspelling: description, faunistics, discussion); Guignot 1952d: 4 (discussion: misidentification by Guignot 1946c); Legros 1954: 268 (faunistics); Legros 1958: 211 (faunistics); Guignot 1959a: 536, 567 (listed as synonym of *Laccophilus pictipennis* Sharp, discussion); Nilsson and Persson 1993: 79 (list, synonymy); Nilsson 2001: 249 (catalogue, faunistics, list, synonymy); Nilsson 2015: 168 (catalogue, faunistics, list, synonymy). **Confirmed synonym.**

##### Type localities.

*Laccophilus pictipennis*: Saudi Arabia: Hedjaz.

*Laccophilus wehnckei*: Tanzania: Zanzibar.

##### Type material studied

(6 exs.): *Laccophilus pictipennis*: Lectotype (designated by Brancucci (1983b)): Male: “Lectotype / Lectotypus / male-label / 566 / Hedjaz Millingen / Sharp Coll. 1905-313 / *Laccophilus pictipennis* Shp M.E. Bacchus det. 1977 Syntype / Lectotype *Laccophilus pictipennis* Sharp des. Brancucci 81” (BMNH; habitus in Fig. 398). – Paralectotypes: same data as lectotype but labeled as “Paralectotype” (1 ex. BMNH); “Paralectotype / Paralectotypus / Abyssinia / Sharp Coll. 1905-313 / Type 566 *Laccophilus pictipennis* / *Laccophilus pictipennis* Shp M.E. Bacchus det. 1977 Syntype” (1 ex. BMNH); same data but add: “Raffray” (1 ex. BMNH).

*Laccophilus wehnckei*: Lectotype (by present designation): female: “Type / E. Africa / Sharp Coll. 1905-313 / Type 620 *Laccophilus wehnckei* sp. n. Zanzibar” (BMNH; habitus in Fig. 399). – Paralectotype: female: principally same data as lectotype but labelled as “cotype” (1 ex. BMNH).

##### Additional material studied

(15 exs.). **Ethiopia**: “Saati Levander” (1 ex. MZH); “Abyssinia” (1 ex. ZMHB). – **Somalia**: “Daragodleh 25.6. 1963 Linnavuori” (6 exs. MZH); “Lasgori / *Laccophilus pictipennis* Sharp det. Brancucci 1982” (5 exx. ZMHB, 1 ex. NHMB). Non-African record: – **Yemen**: “W. Aden Prot. nr Lahej 9-15.7. 1963 Linnavuori” (1 ex. MZH).

##### Comments on synonymy.

Earlier established synonymy of *Laccophilus pictipennis* and *Laccophilus wehnckei* is confirmed by study of external characters; no diagnostically important differences detected. As no males are available of *Laccophilus wehnckei* we could not in this case undertake comparison of male genitalia. *Laccophilus discretus* Sharp, 1882, described from Saudi Arabia, has earlier been synonymized with *Laccophilus pictipennis*. It has never been recorded from Africa by its own name and accordingly, it is outside the scope of this study.

##### Diagnosis.

*Laccophilus pictipennis* is characterized by peculiar elytral colour pattern in combination with penis, which is slightly and evenly curved, tapering gradually towards its apex. Note also that male apical ventrite lacks asymmetrically located knob, although excavated on each side of midline and slight medial extension (Fig. 45).

##### Description.

Body length 4.4–4.7 mm, width 2.5–2.8 mm. Dorsal, aspect of body with fairly distinct colour pattern. African specimens seem to have vaguer dorsal colour pattern (Figs 398–399).

Head: Pale ferrugineous. Punctation indistinct, almost absent; close to eyes with two minute deptressions with irregular, fine punctures. Shiny, although irregularly and rather finely reticulated. In part double reticulation weakly discernible (delimitation in two distinct size classes of meshes vague).

Pronotum: Pale ferrugineous. At frontal margin with a vague, ferrugineous, almost bilobed marking. Punctation fine, sparse to rather sparse and irregularly distributed. Punctures frontally densest. Rather shiny although microsculptured. Reticulation indistinctly divided into two kinds; smaller meshes sometimes discernible within large meshes. In part, only large meshes well-developed.

Elytra: Pale ferrugineous, with extensive, distinct, dark ferrugineous to brownish markings (Fig. 398). Sometimes elytral colour pattern rather vague (Fig. 399). Reticulation double; large meshes contain generally 3–6 small meshes. Small meshes fine, sometimes weakly developed and indistinct. Irregular, discal, dorsolateral and lateral rows of punctures are discernible. All rows of punctures rather sparse, not forming straight rows.

Ventral aspect: Pale ferrugineous to ferrugineous. Rather shiny, although extensively, finely microsculptured. Besides microsculpture metacoxal plates with shallow, transverse furrows and abdomen especially basally with distinct striae. Apical ventrite lacks knob (Fig. 45). Apex of prosternal process rather narrow and pointed.

Legs: Pro- and mesotarsus slightly enlarged, with fine suckers.

Male genitalia: Penis in lateral aspect quite broad, from base slightly and evenly curved to apex (Figs 247–248).

Female: Pro- and mesotarsus slender. Apical ventrite (Fig. 46).

##### Distribution.

Somalia, Ethiopia, Tanzania (Zanzibar) (Fig. 534). Material examined also from Arabian Peninsula (Saudi Arabia and Yemen). Only personally examined specimens accepted.

##### Collecting circumstances.

Almost unknown in Africa. Hajek and Reiter (2014) report the species from Oman being mostly associated with running water, especially in relatively permanent side pools of streams and river at lower and middle altitudes.

### Species group 7 (*Laccophilus taeniolatus* group)

**Diagnosis.** Medium to large sized species; body length 3.5–5.3 mm, width 1.9–2.9 mm.

Shape of body oval-oblong, dorsoventrally flattened (Fig. 403). Body dorsally with distinct colour pattern. Elytron provided with dense irrorations, which often cover whole disc. In a few species dark irrorations “hollow”; i.e. single irroration encloses a pale and narrow irroration (Fig. 401). One species has a sub-basal, pale area with reduced and sparse irrorations (Fig. 415). Some species have a moderate, mediobasal area with no or reduced irrorations (Fig. 412). Finally, sometimes, irrorations in part merged into larger, dark areas. Dorsal microsculpture almost simple/indistinctly double, of one kind; large meshes strongly reduced and hardly discernible; sometimes discernible but only slightly more strongly developed than small meshes. In two species, small meshes are reduced or weakly developed, while large meshes discernible.

**Figures 47–58.**
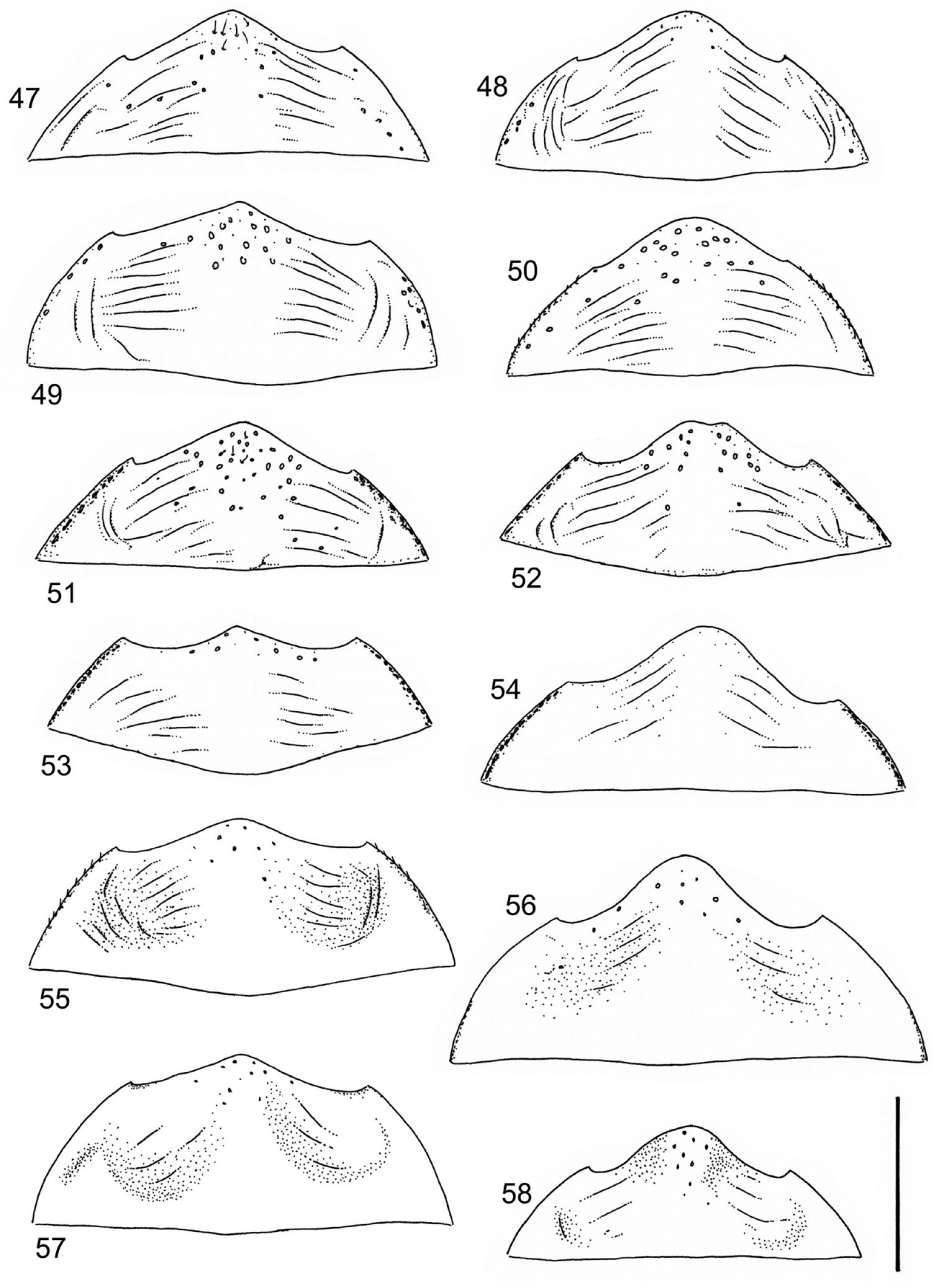
Apical ventrites **47** *Laccophilus continentalis* male, and **48** female **49** *Laccophilus posticus* male, and **50** female **51** *Laccophilus inobservatus* male, and **52** female **53** *Laccophilus simplicistriatus* male, and **54** female **55** *Laccophilus taeniolatus* male, and **56** female **57** *Laccophilus propinquus* male, and **58** female. Scale bar 0.5 mm.

Prosternal process rather slender, extended, apically pointed. Apical ventrite posteriorly on each side excavated, medially posteriorly extended, but asymmetrical knob always absent (posterior outline of ventrite “undulate” with medial extension) (Fig. 47). No stridulation apparatus on metacoxal plates. Metacoxal process posteriorly truncate, not posteriorly extended (Fig. 6).

Paramere simple, somewhat enlarged but not distinctly modified (Fig. 250). Penis more or less evenly curved, apically often enlarged and provided with minor processes. One species with penis apex lacking modifications (Fig. 256).

**Species composition and distribution.** Nine species are recognized; two of them occur in Madagascar and seven in mainland Africa, South of Sahara. To observe, that from point 3 in the key below, external characters are variable and male genitalia must be studied.

#### Key to species (males)

**Table:** 

1	Large species, length of body 4.8-5.3 mm; elytra with vague but clear, dark, longitudinal lines of which medial lines enclose an undulate pale marking (Fig. 416)	***Laccophilus rivulosus*** (p. 85)
–	Smaller species, length of body 3.5-4.6 mm; elytral colour pattern different	**2**
2	Dark irrorations sparse at elytra-base forming a sub-basal, transverse pale area (Fig. 415); apical half of penis, strongly curved, simple, exhibits no distinct modifications (Fig. 256)	***Laccophilus irroratus*** (p. 83)
–	Dark irrorations dense at base; if sparse no transverse, pale area formed (Figs 403, 412); apex of penis exhibits modifications	**3**
3	Dark irrorations at least partly “hollow” with pale irroration-area enclosed (Fig. 401)	**4**
–	Dark irroration almost completely dark (Fig. 412)	**6**
4	Penis apex broad; ends abruptly and exhibits no extension (Fig. 251)	***Laccophilus inobservatus*** (p. 63)
–	Penis apex less broad; apex externally somewhat extended (Fig. 249)	**5**
5	Penis apex delicate, less pronounced (Fig. 249) (African mainland)	***Laccophilus continentalis*** (p. 53)
–	Penis apex robust, pronounced (Fig. 250) (Madagascar)	***Laccophilus posticus*** (p. 58)
6	Penis apex enlarged on both sides close to truncate apex (Fig. 252)	***Laccophilus simplicistriatus*** (p. 66)
–	Penis apex enlarged on one (marginal) side or not enlarged close to truncate apex (Fig. 253)	**7**
7	Apical process of penis apex curved upwards (Fig. 255) (Madagascar)	***Laccophilus complicatus*** (p. 80)
–	Apical process of penis apex not curved upwards (Fig. 253) (African mainland)	**8**
8	Penis long with apical process distinct (Fig. 253)	***Laccophilus taeniolatus*** (p. 72)
–	Penis shorter with vague apical process (Fig. 254)	***Laccophilus propinquus*** (p. 79)

#### Laccophilus continentalis

Taxon classificationAnimaliaColeopteraDytiscidae

Gschwendtner, 1935

Figs 47–48 , 249 , 400–401 , 535

Laccophilus posticus continentalis Gschwendtner 1935a: 16, 18 (original description, faunistics).
Laccophilus continentalis Gschwendter, Guignot 1946c: 279, 281, 283, 312, 316 (discussion, description, faunistics); Capra 1952: 6: (faunistics); Omer-Cooper 1956: 21 (faunistics, biology); Omer-Cooper 1957: 20, 21 (discussion, description); Omer-Cooper 1958a: 57, 59 (faunistics, biology); Omer-Cooper 1958b: 37, 43, 44, 45 (description, discussion, faunistics, biology); Guignot 1959a: 570, 573, 575 (redescription, faunistics); Omer-Cooper 1965: 77, 81 (description, faunuistics); Omer-Cooper 1970: 288, 289, 290 (discussion, description, faunistics); Rocchi 1975: 48 (faunistics); Bilardo 1976: 190 (faunistics, biology); Bilardo and Rocchi 1987: 104 (faunistics, biology); Rocchi 1990:442 (faunistics); Nilsson 2001: 242 (catalougue, faunistics); Nilsson 2015: 210 (catalougue, faunistics).
Laccophilus perplexus Omer-Cooper 1970: 287, 288, 289, 290 (original description, faunistics, discussion.); Nilsson 2001: 248 (catalogue, faunistics); Nilsson 2015: 215 (catalougue, faunistics). **New synonym**.

##### Type localities.

*Laccophilus continentalis*: Botswana: Kalahari, Tsotsorogo Pan.

*Laccophilus perplexus*: Mozambique: Umbeluzi River near Goba.

##### Type material, studied

(21 exs.). *Laccophilus continentalis*: Lectotype (by present designation): male: “V.-L. Kal. Exp. Tsotsorogo Pan 17/6-9/7/30 / Type male (symbol) Gschw. / *Laccophilus posticus continentalis* det. Gschwendtner” (TMSA; habitus in Fig. 400). – Paralectotypes: Same data as lectotype, but “Type female (symbol)” (1 ex. TMSA); “V.-L. Kal. Exp. N’Kate Makarikari 6-23/8/1930 / Paratypus *Laccophilus posticus continentalis* ssp. L. Gschwendtner” (10 exs. TMSA, 1 ex. OLML); same as lectotype but labelled as “Paratype Gschw.” (5 exs. OLML, 2 exs. AMGS).

*Laccophilus perplexus*: Holotype: male: “Type / *Laccophilus perplexus* sp. n. / Mozambique Umbeluzi River near Goba 4.12. 1948 J.O.C.” (AMGS; according to original description holotype preserved in BMNH).

##### Additional material, studied

(518 exs.). **Senegal**: “Sumpf von Peykone, Senegal 9. 08 Riggenbach S.V.“ (1 ex. ZMHB). – **Gambia-S. Senegal**: “Stream of Selety 13°10'N-16°36'W 19.2. 1976 Holmen leg.” (2 exs. ZMUC). – **Sudan**: “Wad Medani a. Bl. Nil 29.10. 1979 Hieke“ (1 ex. ZMHB); same but “12.10. 1979 lux“ (2 exs. ZMHB); same but “18.10. 1979“(1 ex. ZMHB); same but “8.10. 1979“ (2 exs. ZMHB); same but “9.10. 1979“ (1 ex. ZMHB); same but “30-31.10. 1979“ (1 ex. ZMHB); same but “20.10.1979“ (1 ex. ZMHB); same but “15.10. 1979“ (1 ex. ZMHB); same but “22.10.1979“ (2 exs. ZMHB); same but “leg. Königsmann“ (1 ex. ZMHB); “Senaar a.Bl. Nil, lux 21.10.1979 Hieke“ (5 exs. ZMHB); “Umm Banein, light trap 14.11. 1962 Linnavuori” (1 ex. MZH). – **Ghana**: “N Region Nyankpala 183 m N9°25’-W1°00’ Dr. S. Endrödy-Younga / shore washing 10.2. 1970“ (1 ex. CGW). – **Nigeria**: “Samaru 17.5. 1959, Sands / light trap” (1 ex. BMNH); “Nt Kano 1.5. 1928 Lindwer Madsen” (1 ex. ZMUC). – **Somalia**: “Somali Rep. 1961 Roffey”(3 exs. BMNH, 1 ex. MZH). – **Kenya**: “Kibwezi Scheffler leg.“ (1 ex. ZMHB); same as but “1906“ (1 ex. ZMHB); “Eastern Mwingi, Nguni env. 28.11. 1999 Snizek” (1 ex. NMW); “SE Kenya ShimbaHills 20 km S Mombasa 5.6. 1985 Lödl” (1 ex. NMW, 1 ex. MZH); Mafisini, pond, Kwale District 19.9. 1976 Holmen leg.” (1 ex. ZMUC); “Maji ya Chumvi River, Kwale District 16.9. 1976 Holmen leg.” (1 ex. ZMUC); “Maji-Chumwi (Wa Nyika) Alluaud 7. 1903” (2 exs. NHMB); “Athi River, Machakos District 14.9. 1976 Holmen det“ (1 ex. ZMUC); “Kombeni River, Mazeras, Kilifi distr 15.9. 1976 Holmen” (2 exs. ZMUC, 2 exs. MZH); “Mariakani dam, Kilifi District 16.9. 1976 Holmen” (4 exs. MZH); “Mandera R. Dana 23.10. 1970 Brown” (1 ex. BMNH); “Wajir 27.10. 1970 Brown” (1 ex. BMNH); “Malindi, alle luci, 15.11.-5.12. 1989 / *Laccophilus continentalis* Gschwendtner det. Rocchi 1990” (3 exs. CSR); “Voi 11. 1997 Snizek M. / *Laccophilus continentalis* Gschwendtner det. Rocchi 1990” (3 exs. CSR); “Voi Mtito Andei, light trap roof Tsavo Inn 24-25.11. 1990 Päts & Viklund” (1 ex. MZH); “Fort Hall / Coll. E. Häuser / *Laccophilus continentalis* Gschwendtner det. Brancucci” (1 ex. ZMHB); “Afr. or Jkurha” (1 ex. ZMHB). – **Tanzania**: “Daressalaam, Pangani und Hinterland Regner” (1 ex. ZMHB); “Daressalaam leg. Methner” (1 ex. NHMB, 1 ex. ZMHB); “Reg. Morogoro Mikumi 17-20.12. 1993 Bednatik” (1 ex. NMW); “Mts Uluguru, Morogoro Campus Fac. Agric., UV, 600 m 5-6. 1971 / *Laccophilus continentalis* Gschw. det. Bilardo” (1 ex. NHMB); “Ponds S of Korogwe, Korogwe District 24.9. 1976 Holmen” (2 exs. ZMUC, 1 ex. MZH); “Rice field S of Tanga, Tanga District 26.9. 1976 Holmen” (1 ex. ZMUC); “Tanga / Sjöstedt” (1 ex. NHRS); “Tanga Reimer S.” (1 ex. ZMHB); “Tanganyika Pond in stream 103 (?) miles from Dodoma 15.2. 1954 JOC” (1 ex. AMGS); “Tanga Prov. 4-5. 1950 Sweeney / At light” (2 exs. BMNH); “Usa River 3900 feet Dr. J. Szunyoghy / Light trap 15.11.-31.12. 1965” (1 ex. CGW); ”2 mi to L. Manyara, seashore 3150 feet Dr. J. Szunyoghy / singled material 1–26. 1965” (1 ex. CGW); “Usagara” (1 ex. ZMHB); “Umgb Urumba 15.9. D.O.A. leg. Methner / *Laccophilus continentalis* Gschw. det. M. Brancucci” (3 exs. ZMHB); “J. Kurha” (1 ex. ZMHB); “Zanzibar Pemba 23. Sept. 1955 Fowler” (5 exs. AMGS); “Zanzibar 13^th^ Sept. 1955 JOC.” (2 exs. AMGS); “Zanzibar 5. 1954 Brown” (2 exs. BMNH, 1 ex. MZH); “Zanzibar / Reimer S. & Schultz. – **Zambia**: “S Luangwa NP, Mfuwe Crocodile Farm, 13.06.03S, 31.47.32E, 450 m, lux 23.3. 1993 Uhlig” (45 exs. ZMHB, 2 exs. MZH); same but “21.3. 1993” (4 exs. ZMHB); same but “24.3. 1993” (6 exs. ZMHB); same but “13.06S, 31.47S, 21-24.3. 1993 Göllner” (2 exs. ZMHB); same but “Deckert” (1 ex. ZMHB). – **Namibia**: “E Caprivi: Katima Mulilo, lux, 17.29S, 24.17E, 3-8.3. 1992 Uhlig” (65 exs. ZMHB, 5 exs. MZH; habitus in Fig. 401); same but “Deckert leg.” (2 exs. ZMHb); same but “Göllner leg.” (2 exs. ZMHB); “E Caprivi: 3 km E Katima Mulilo, 17.29S, 24.18E, Hippo Camp, in Swimming Pool 6.3.1992 Uhlig” (1 ex. ZMHB); “E Caprivi: 30 km SE Katima Mulilo 17.31S, 24.25E, Zambesi – Altwasserarm, lux 6.3. 1992 Uhlig” (7 exs. ZMHB, 1 ex. NMNW); “E Caprivi: Mudumu NP, Nakatwa, 18.10S, 23.26E, 8-13.3. 1992 Uhlig” (3 exs. ZMHB); same but “Buffalo Trails Camp lux, ca. 18.10S, 23.26E, 12.3. 1992” (3 exs. ZMHB); same but “Kwando-Ufer, *Phragmites*, schlammig” (1 ex. ZMHB); “Kavango Popa Falls 18.07S, 21.35E, lux 26.2.-3.3. 1992 lux Deckert” (1 ex. ZMHB); same but “Uhlig” (1 ex. ZMHB); “Kavango Mahango Game Reserve 18.17S, 21.43E, lux 2.3. 1992 Göllner” (1 ex. ZMHB); Okavango Distr., Mutompo, 60 km S Rundu, 18.18.38,7S, 19.15.29,4E, 1180 m NN, 13.3. 2003, hand light trap, Frisch & Voland” (1 ex. ZMHB). – **Botswana**: “Serowe, sevage ponds, Farmer’s Brigade 1.6. 1987, SE22 26 BD, Forchhammer leg.” (3 exs. MZH); same but “7.6. 1987” (3 exs. MZH); same data but ”4. 1988 / *Laccophilus continentalis* Gschwendtner det. Rocchi 1993” (2 exs. CSR); “Kasane, Chobe Safari lodge, 17.48.32S, 25.08.39E, 26.11.1993 lux, Uhlig” (5 exs. ZMHB, 3 exs. MZH); “5 km NW San-ta-wani Safari Lodge, 19.27.01S, 23.38.46E, lux 8-9.3.1993 Uhlig” (27 exs. ZMHB); “5 km NW San-ta-wani Safari Lodge, 19.27S, 23.38E, lux 8-9.3. 1993 Göllner” (1 ex. ZMHB); “6 km E Kalkfontein, 22.04S, 20.56E, 7.3. 1993 lux Göllner” (1 ex. ZMHB); “Okavango Delta, Moremi Wildlife Res. Third Bridge Campsite, lux 10.3. 1993, 19.14.22S, 23.21.24E, 10.3. 1993 Uhlig” (3 exs. ZMHB); “Chobe NP Savuti Camp 18.33.55S, 24.03.53E, lux 11.3. 1993 Uhlig” (5 exs. ZMHB); “R. Thamalakane 7 mls NE Maun 20.4. 1972/at light” (1 ex. BMNH). – **Zimbabwe**: “Victoria Falls Zambezi NP –Camp 11-12.12. 1993, 17.53S, 25.49E, lux, Uhlig” (1 ex. ZMHB); “S. Rhodesia Pool Lundi 22. N. 1948 JOC.” (6 exs. AMGS); “S. Rhodesia Wankie Reserve water holes 3.9. 1948 / *Laccophilus continentalis* Gschw. Det. JOC.” (1 ex. AMGS); “Wankie Game Res. 5 Sept. 1948 JOC. Pools at Robins rest camp / *Laccophilus continentalis* Gschw. Det. JOC.” (1 ex. AMGS); “Wankie Game Res. 4 Sept. 1948 JOC.” (4 exs. AMGS, 1 ex. BMNH); “Wankie Reserve water hole Sept. 1948” (13 exs. AMGS); “Wankie Game Reserve Shapi pond 5.IX. 1948 JOC. / *Laccophilus continentalis* Gschw. Det. JOC.” (9 exs. AMGS); “Wankie Game Reserve Shapi pan 5.IX. 1948” (3 exs. AMGS); “Wankie Reserve Musumu dam 14.IX. 1948 JOC. / *Laccophilus continentalis* Gschw. Det. JOC.” (1 ex. AMGS); “Wankie Game Reserve Sept. waterhole 1948 JOC. / *Laccophilus continentalis* Gschw. Det. JOC.” (7 exs. AMGS);”5 mi SE Wankie 7.4.1968 Spangler” (34 exs. USNM, 6 exs. MZH); “Marandellas 2. N. 1948 JOC. / *Laccophilus continentalis* Gschw. Det. JOC.” (1 ex. AMGS); “Gwai River 3.4. 1968 Spangler” (8 exs. USNM); “Shamgani 60 km SW of Gweru 2.12. 1998 F. Kantner leg.” (1 ex. NMPC);”Tongwe 30 km N Beitbridge 7.12. 1998 Kantner leg.” (1 ex. NMPC). – **Mozambique**: “Mozambique Beira 7. Sept. 1955 JOC.” (12 exs. AMGS); “Moz., Dambo Pan 30.6. 1960” (2 exs. AMGS); “Port. E Afr. Lorenco Marques 3.12. 1948 JOC.” (2 exs. AMGS); “Lourenco Marques Dec. 1948 JOC.” (1 ex. AMGS); “Umbuluzi R. nr. Goba 4.12. 1948 J.O-C.” (2 exs. AMGS). – **Swaziland**: “Muddy pond nr Stegi 4.12. 1948 / *Laccophilus continentalis* Gschw. Det. JOC.” (1 ex. AMGS). – **South Africa**: “Kruger Nat. Pk. Mathlakusapan 22.37S-31.22E / shorewashing 9.2. 1994 Endrödy-Younga leg.” (9 exs. TMSA, 2 exs. MZH); “Kruger Nat. Pk. Skukuza 12 km S, 25.04S-31.37E / UV light, 6.3. 1996 Endrödy-Younga leg.” (10 exs. TMSA, 2 exs. MZH); “Kruger Nat. Pk, Skukuza Res. camp 24.59S-31.36E / UV light & trap 25.2. 1995 Endrödy-Younga” (1 ex. TMSA); “Kruger Nat. Pk, Skukuza Res. camp 24.59S-31.35E / UV light 3.3. 1996 Endrödy-Younga” (1 ex. TMSA); “Kruger Nat. Pk, Skukuza Res. camp 25.00S-31.35E / UV light & trap 19.2. 1995 Endrödy-Younga” (1 ex. TMSA); “Kruger Nat. Pk, Skukuza, 40 km S dam 20.04S-31.36E/Shorewashing 23.2. 1995 Endrödy-Younga” (1 ex. TMSA); “Kruger Nat. Pk, Skukuza Res. camp 24.59S-31.35E / UV light 7.3. 1996 Endrödy-Younga” (1 ex. TMSA); “Kruger Nat. Pk, Pafuri research camp 4 km W, 22.25S-31.09E / 1.2.1994 UV light & trap Endrödy-Younga leg.” (4 exs. TMSA); “Kruger Nat. Pk, Pafuri res. camp, 22.25S-31.12E/30.1. 1994 UV light & trap Endrödy-Younga leg.” (3 exs. TMSA, 1 ex. MZH); “Kruger Nat. Pk, Pafuri research camp, 22.25S-31.10E / 2.2. 1994 UV light & trap Endrödy-Younga leg.” (1 ex. TMSA); “Kruger Nat. Pk Levuvu River 22.27S-31.10E / 12.2. 1994 shorewashing Endrödy-Younga leg.” (4 exs. TMSA); “Kruger Nat. Pk, Malonga springs 22.36S-31.20E / 8.2. 1994 shorewashing Endrödy-Younga leg.” (1 ex. TMSA); “Kruger Nat. Pk Punda Maria Ngots Dam 21.26S-31.14E / 7.2. 1994 shorewashing Endrödy-Younga leg.” (1 ex. TMSA); “Kruger Nat. Pk Letaba Riv. bel. dam 23.46S-31.30E/1.3. 1995 shorewashing Endrödy-Younga leg.” (1 ex. TMSA); “Transvaal Kruger National Park, Leeu Pan NE Skukuza 1.5. 1951 / *Laccophilus continentalis* Gschw. det. J. Omer-Cooper” (1 ex. MZLU); “Trsvl, Waterhole nr Police Picket, KNP. 30.6. 1960” (25 exs. AMGS); “Trsvl, Kumana Pan 24 km S Satara Camp, KNP, N-24.610, E31.800, 18.6. 1960” (1 ex. AMGS); “Gauteng Tswaing 25.24S-28.06E / 16.2. 2003 light trap TMSA staff leg.” (2 exs. TMSA); “Trsvl Mmabolela Estate 22.40S-28.15E / 21.11. 1991 shorew. Limpopo Riv. Klimazewski leg.” (1 ex. TMSA); “Trsvl 5 mi W Warmbad 24-25.2. 1968 Spangler” (1 ex. USNM); “Trsvl, Hartebeespoortdam, N-25.730, E27.820, 30.5. 1971 Reavell” (1 ex. AMGS); “N. Prov. Messina Nat. Res. 22.21S-30.03E / 13.12. 2000 light trap Müller, Burger leg.” (1 ex. TMSA); “Kw. Natal, Sivai Lagoon 10.3. 1981 Reavell” (1 ex. AMGS); “Zululd. Ndumu Banzi fresh. wat. pan 26.53S-32.16E / 16.2. 1989 shorewashing Endrödy & Klimazew” (1 ex. TMSA); “Natal Zululand Mtuba-tuba 23.9. 1947 JOC”. (1 ex. AMGS); “Natal Durban Umgeni Trägårdh” (1 ex. MZLU); “Zululand Hhu-Hluve 18.IX. 1947” (1 ex. AMGS); “Natal, roadside puddles, ca. 2 km S Mbazwana to Hluhluwe nr Sodwana 9.1. 1997 Turner” (9 exs. NHMB); same data but ”5.3. 1997” (6 exs. NHMB); “Natal, Mkuze NP 17.36S, 32.13E, 2-3.2. 1994 lux Uhlig (1 ex. ZMHB);”Natal, Waterton Timber Co. N-28.20.5, E32.14, at light Atkinson” (5 exs. NHMB).

##### Specimens with uncertain location.

“Lowrie 17.5. 1955” (1 ex. AMGS); “Kotsch Lu.(?) Kohu” (1 ex. NMW).

##### Comments on the synonymy.

The lectotype of *Laccophilus continentalis* and the holotype of *Laccophilus perplexus* have been examined and compared. Minor difference is present in appearance of the elytral colour pattern and shape of the penis. Differences observed are, however, superficial and clearly falls within the variation exhibited by one species. *Laccophilus continentalis* being the older name is the valid name of the species.

##### Diagnosis.

Externally *Laccophilus continentalis* resembles much of *Laccophilus posticus*. Useful diagnostic character is the shape of the penis. In *Laccophilus continentalis* apical part of penis is less prominent in comparison with rest of the penis when *Laccophilus posticus* is characterized by distinctly more prominent apex of penis. The shape of penis separates the two species from all other African *Laccophilus* species.

##### Description.

Body length 3.6–4.1 mm, width 1.9–2.2 mm. Pale ferrugineous, with dark ferrugineous, extensive but variable elytral irrorations (Figs 400–401). Sometimes single irroration is formed only by distinct outlines leaving the middle pale coloured. Other extreme is that single irroration is solid and totally dark.

Head: Frontally pale ferrugineous; posteriorly head becomes gradually slightly darker; at pronotum ferrugineous to pale brown. Head often uniformly pale coloured. Submat, finely microsculptured; reticulation almost uniform. Indistinct fragments of double reticulation discernible (large meshes incomplete). Almost impunctate; at eyes with fine, hardly discernible punctures.

Pronotum: Pale ferrugineous, frontally with a vague dark ferruginous to dark brownish marking. Darker marking on disc sometimes reduced and visible in frontal part of pronotum. Submat, finely microsculptured. Reticulation almost simple; indistinct fragments of coarse reticulation discernible: large meshes incomplete. Almost impunctate; frontally and laterally with scattered, very fine punctures discernible.

Elytra: Pale ferrugineous, with variable, dark ferrugineous irrorations. Irrorations sometimes in part reduced; outlines only distinct (Figs 400–401). Submat, finely microsculpture; reticulation of one kind. Discally with sparse, irregular row of very fine punctures. Laterally with sparsely scattered, very fine punctures. Fine lateral furrow formed of fine punctures located somewhat posterior to middle towards elytral apex.

Ventral aspect: Pale ferrugineous. Abdomen in part ferrugineous to dark ferrugineous, with lateral and apical areas paler. Almost impunctate. Extensively very finely microsculptured. Prosternal process slender; apex extended and pointed. Metacoxal plates in anterior half with some transversely located, shallow furrows. Abdomen with sparse but distinct striae. Apical ventrite (Fig. 47).

Legs: Pro- and mesotarsus rather long and slender. Pro- and mesotarsus provided with distinct suckers.

Male genitalia: Extreme apex of penis forms a short, sharp and small extension (Fig. 249).

Female: Apical ventrite (Fig. 48).

##### Distribution.

Gambia, Senegal, Sudan, Ghana, Nigeria, Somalia, Kenya, Tanzania, Namibia, Botswana, Zimbabwe, Mozambique, South Africa (Fig. 535). Additionally, *Laccophilus continentalis* is recorded from Swaziland (Omer-Cooper 1958b). Record outside Africa but in close neigborhood is Yemen (Socotra) by Hájek and Reiter (2014).

##### Collecting circumstances.

The biology and habitats of *Laccophilus continentalis* are not well documented. Scattered observations can be gathered from literature and from collection data written on the labels. Label data give that the species is capable of flying and attracted by light. *Laccophilus continentalis* has also been sampled from various water-bodies as ponds and streams. Omer-Cooper (1956) reports the species in Mozambique from pools, a ditch and a slow flowing stream with vegetation as *Marsilia* sp., *Lagarosiphon* sp., *Limnophyton* sp., water lilies and duckweed. Also reported from Zimbabwe in water holes, springs and dams used by the game. Additionally, taken from streams e.g. with a pool in the river bed, in ponds with rock and gravel bottom and some mud deposition; blue water lilies and weed growing in the pools. Guignot (1946c) assumed that the species is a character-species of steppes and savannes.

#### Laccophilus posticus

Taxon classificationAnimaliaColeopteraDytiscidae

Aubé, 1838

Figs 49–50 , 250 , 402–405 , 536

Laccophilus posticus Aubé 1838: 428 (original description, faunistics); Erichson 1843: 205 (faunistics); Boheman 1848: 245 (faunistics, description); Sharp 1882: 309 (redescription, faunistics); van den Branden 1885: 23 (catalogue, faunistics); Severin 1892: 472 (type deposition); Régimbart 1895: 136, 137, 138, 141 (description, faunistics, discussion); Alluaud 1897: 212 (faunistics); Peschet 1917: 23, 24, 26, 55 (description, faunistics, discussion); Zimmermann 1920a: 24 (catalogue); Gschwendtner 1935a: 17, 18 (faunistics, description); Guignot 1946c: 279, 281, 283 (discussion, description, faunistics); 1952e: 170 (discussion); Guignot 1955d: 67 (discussion); Vinson 1956: 28 (faunistics, list, biology); Omer-Cooper 1958b: 43, 45 (discussion, description); Guignot 1959a: 570, 572, 573, 575 (description, discussion, faunistics); Guignot 1961a: 931 (faunistics); Vinson 1967: 314 (faunistics, list); Omer-Cooper 1970: 290 (description); Bertrand and Legros 1971: 245 (faunistics, biology); Wewalka 1980: 730 (faunistics list); Bameul 1984: 94 (faunistics,); Rocchi 1991: 80, 86 (faunistics); Nilsson 2001: 249 (catalogue, faunistics); Pederzani and Rocchi 2009: 95 (faunistics, list); Nilsson 2015: 216 (catalogue, faunistics).

##### Type locality.

Mauritius (Ile de France).

##### Type material studied

(1 ex.). Holotype: female: “Data in NHRS JLKB 000030279/Ex – Museo Dejean / D. Sharp Monogr. / *irroratus* après Dej. / *Laccophilus posticus* var. / Dr. Régimbart vidit 1893 / Coll. Oberthur” (MNHN). [Comment: in the original description Aubé (1838) mentions existence of an additional specimen from Philippines. We have not located it and accordingly the exact taxonomic status of this specimen remains unknown.]

##### Additional material studied

(486 exs.). **Mauritius**: “Ile de Maurice Avril 1908 d’Emmerez / Museum Paris 1945 Coll. R. Peschet / *Laccophilus posticus* Aubé” (4 exs. MNHN); “Ile de Maurice Bambous Carié Déc. 1912 / Museum Paris 1945 Coll. R. Peschet / *Laccophilus posticus* Aubé R. Peschet det. 1917” (1 ex. MNHN); “Balaclava 4.5. 2007 Madl” (2 exs. NMW, 1 ex. MZH); “Nr. Triolet, nr. Fond du Sac, temp. new pool at roadside, terr. plants flooded by water, ephemeral aquatic habitat” (1 ex. CCT); “Ins. Mauritius Westw.” (1 ex. ZMHB); “Mauritius” (2 exs. ZMHB). – **Madagascar**: “Mahajanga: Boeny: Ankarafantsika NP, S16.30341, E046.81073, 74 m.a.o. 29.11. 2009, 22W black light, field Bergsten et al. leg” (13 exs. NHRS); same data, add “NHRS-JLKB 000000511” (1 ex. NHRS); same data, add “NHRS-JLKB 000000514” (1 ex. NHRS); same data but “S16.30270, E046.80996, 75 m.a.o. 30.11. 2009” (12 exs. NHRS); same data, add “NHRS-JLKB 000000515” (1 ex. NHRS); same data but “S16.30653, E046.81227, 108 m.a.o. 28.11. 2009” (4 exs. NHRS); same data, add “NHRS-JLKB 000000494” (1 ex. NHRS); same data but “S16.31215, E046.81523, 76 m.a.o. 29.11. 2009” (17 exs. NHRS); same data, add “NHRS-JLKB 000000513” (1 ex. NHRS); “Mahajanga: Boeny: Mahavavy Kinkony RS. S16.14653, E045.94926, 9 m.a.o. 4.12. 2009 water net, field leg. Bergsten et al” (15 exs. NHRS); Same data; add “NHRS-JLKB 000000501” (1 ex. NHRS); same data but “S16.06651, E045.77627, 24 m.a.o. 5.12. 2009” (10 exs. NHRS); same data, Andasibe Adasibe add “NHRS-JLKB 000000510” (1 ex. NHRS); same data but “S16.15502, E045.91878, 10 m.a.o. 3.12. 2009” (10 exs. NHRS); same data but “S16.13337, E045.95778, 19 m.a.o., 4.12. 2009 / NHRS-JLBK 000000509” (1 ex. NHRS); same data but “NHRS-JLKB 000000496” (1 ex. NHRS); same data but “S16.15890, E045.93967, 3.12. 2009 / NHRS-JLBK 000000508” (1 ex. NHRS); same data but “S16.14147, E045.93661, 12 m.a.o., 3.12. 2009 / NHRS-JLKB 000000502” (1 ex. NHRS); “Toliara: Menabe: Menabe RS, S19.92773, E045.52253, 102 m.a.o. 10.12. 2009 water net, field” (13 exs. BMNH); same data, add “NHRS-JLKB 000000516” (1 ex. NHRS); same data but “S20.09034, E044.56400, 45 m.a.o. 11.12. 2009 / NHRS-JLKB (1 ex. NHRS); “Toliara: Menabe: Kirindy RS. S20.07641, E044.67478, 65 m.a.o., 11.12. 2009 water net, field” (1 ex. NHRS); same data, add “NHRS-JLKB 000000497” (1 ex. NHRS); same data but “000000504” (1 ex. NHRS); same data but “S20.07655, E044.67532, 57 m.a.o., 12.12. 2009/NHRS-JLKB 000000505” (1 ex. NHRS); “Mahajanga: Melaky btw. Morafenobe-Ambohijanahary, S18.19091, E45.19986, 290 m.a.o. 19.12. 2009 water net, field Bergsten et al.” (9 exs. NHRS); same data, add “NHRS-JLKB 000000512” (1 ex. NHRS); “Mahajanga: Melaky: Tsingy de Bemaraha NP. S18.75724, E044.71239, 72 m.a.o., 17.12. 2009 water net, field Bergsten et al.” (1 ex. NHRS); same data, add “NHRS-JLKB 000000498” (1 ex. NHRS); same data but “S18.775797, E044.71289, 81 m.a.o. 17.12. 2009, 22W black light, Field” (2 exs. NHRS); same data, add: “NHRS-JLKB 000000493” (1 ex. NHRS); same as, except “S19.14210, E044.81309, 59 m.a.o., 14.12. 2009, 22 w black light, field / NHRS-JLBK 000000499” (1 ex. NHRS); “Mahajanga: Melaky: betw. Bekopaka-Antsalova, 18.91556, E044.55546, 47 m.a.o., 16.12. 2009 water net, field Bergsten et al.” (66 exs. NHRS); Same data, add “NHRS-JLKB 000000506” (1 ex. NHRS); “Mahajanga: Melaky: btw. Antsalova-Maintirano S18.30233, E044.18071, 37 m.a.o., 18.12. 2009 Bergsten et al. / NHRS-JLKB 000000503” (1 ex. NHRS); “Ankarana Lat -12.947 Lon 49.0119 27.11. 2004 / DNA voucher BMNH 675044, MSL045:E07 / *Laccophilus posticus* Aubé det. Bergsten” (1 ex, NHRS); “Antsabe, Lat -13.648, Lon 48.721, 21.11. 2004, Balke, Lees & Monaghan / DNA voucher BMNH 672769, MSLO27:D 11 / *Laccophilus posticus* det. Bergsten” (1 ex. NHRS); same but “DNA voucher BMNH 672774, MSL027: E04” (1 ex. NHRS); “Anjiabe Ambony, Ambilobe, Antsabe near camp, ¾ moon, dry, very many water beetles: P25MD12: 21.11. 2004, N: -13.6518, E: 48.7267, 49 m Balke et al” (1 ex. NHRS); “Ambilobe 4. 1951 R.P. / Paratype” (ab. pseudotaenilatus Guignot – not available name) (1 ex. MNHN); “Androka 5. 51 / Paratype” (ab. pseudotaenilatus Guignot – not available name) (1 ex. MNHN); “Isaky Ivondro Ampasy, rice paddies P66, 9.4. 2007 N-24,93056, E46,86317,64 m Ranarilalatiana et al” (41 exs. NHRS); “Isaky Ivondro, Foret Manangotry (rte towards Ranomafana) running water P67C, 9.4. 2007, N-24.7994, E46,86244, 406 m Ranarilalatiana et al.” (4 exs. NHRS); “TOLI, Taolanaro: Isaky Ivondro, Foret Managotry, running water P67C: N -24.799 E 46.862, 406 m 9.4. 2007, leg. Ranarilalalatiana et al./*Laccophilus posticus* Aubé det. Bergsten” (1 ex. NHRS); “TOLI, muddy waterhole, N -23.242, E 44.229, 415 m, 17.5. 2006 Bergsten et al. / BMNH(E) 794210 DNA voucher / *Laccophilus* Aubé Bergsten det. (1 ex. MZH); same as but “BMNH(E) 794228 DNA voucher” (1 ex. NHRS); same but “BMNH(E) 794252 DNA voucher” (1 ex. NHRS); “TOLI NW Ft. Dauphin, rice paddies, P54F, N -24.824, E 46.866, 34.44 m, 19.5. 2006, leg. Bergsten et al / *Laccophilus posticus* det. Bergsten/BMNH(E): <74511> DNA voucher” (1 ex. NHRS); Same but “<794236> DNA voucher” (1 ex. NHRS); “Toli NW Ft Dauphin, rice paddies with water somewhat running under road, 19.5. 2006 N-24°49.472, E46°51.974 34 m Bergsten et al.” (12 exs. NHRS); “TOLI Zombitse Ankilemiletsy, muddy zebu waterhole, some emergent vegetation P42B, 14.5. 2006, N-22°52.112, E44°34.616, 545 m Bergsten et al.” (1 ex. NHRS); “Toli Zombitse Ambiamena, edge PN Zombitse, stagnant zebu-visited marshland, muddy and lots of vegetation, 14.5. 2006 N-22°51.605, E44°37.035, 533 m Bergsten et al.” (4 exs. NHRS); “Toli Zombitse Andranomena R. (Anomena R. ?) near Ranomena, PN Zombitse section Isoky. Pools of muddy & vegetation, stagnant waters in the river basin, among ricefields and *Phragmites* ? 15.5. 2006 -22°38.407, E44°51.866, 578 m Bergsten et al.” (16 exs. NHRS); “Toli MK Manakaralahy, Manakaralahy R. Dried out river with waterhole on sandy bottom with algal mats, 18.5. 2006, N-24°28.162, E44°35.683, 210 m Bergsten et al.” (3 exs. NHRS); “Toli Sakondry Sakondry R, near RN 10 bridge at Satria river with sandy bottom, wide (50m +) and shallow, algal mats along the edges 17.5. 2006, N-23°20.807, E44°20.353, 214 m Bergsten et al.” (5 exs. NHRS); “Toli Menarandra Menarandra R, 49 km from Ampanihy pools beside a river close to village, algae in pools and sandy bottom with some wood 18.5. 2006, N-24°43.104, E45°2.859, 227 m Bergsten et al.” (9 exs. NHRS); “Fian Isalo, Menamaty R.: degraded river, P41AMO1 N: -22.55, E: 45.401, 757 m, 11.5. 2006 leg. Bergsten et al. / *Laccophilus posticus* det. Bergsten/BMNH(E) <745103> DNA voucher” (1 ex. NHRS); “Fian Isalo, Menamaty R., sandy/stony bottom with some vegetation at edges, zebu crossing, degraded P41C, 11.5. 2006 N-22°29.359, E45°23.505, 715 m Bergsten et al.” (3 exs. NHRS); “Fian Isalo Menamaty Riv. degraded with lots of vegetation, used by women to wash clothes in P41AM01, 11.5. 2006 N-22°33.001, E45°24.074, 757 m Bergsten et al.” (40 exs. NHRS); “Tanandava, lum. Schmitz” (8 exs. MRAC, 1 ex. MZH; habitus as in Figs 404–405); “Marovoay, lampe UV 8. 1962 Dubois” (1 ex. MRAC, 1 ex. MZH); “TAM Morarano-Chrome foret 25 km W. bac j. 4. 1992 Pauly” (1 ex. MRAC); “Diego Suarez Alluaud 10. 1893 / Museum Paris coll. Maurice Régimbart 1908 / *posticus* Aubé” (1 ex. MNHN); “Maromandia (Antalaha, Antsiranana) 30.10. 2001 / R. Ankavia nr village, 40 m asl / Gerecke & Goldsmith leg.” (1 ex. BMNH, 1 ex. MZH); “Maroambihy (Sambava, Antsiranana) left affl. R. Lokoho upstr. from village 12.11.2001/90 m a.s.l. / Gerecke & Goldsmith leg.” (1 ex. BMNH; habitus in Fig. 402); “E Mad. Fenerive, foret Tampolo 28.12. 1998 Moravec” (1 ex. NMW); “Mad. east Tampolo 17.17S – 49.25E / 12.11. 1998, E-Y: 3372 light trap, Müller leg.” (1 ex. TMSA); same but “E-Y: 3364, 10.11. 1998” (1 ex. TMSA); “S Mad. Umg. Beloha Franz 1969 / *Laccophilus posticus* Aubé det. Wewalka 1969” (1 ex. NMW); “S Mad. Ambilalilalika, Rd Betioky-Beneloka 50 m asl, 27.1. 1995 Dunay & Janak” (1 ex. NMW); “Betsiboka Bas. 53 km Maevatanana, 47°04'33"E, 16°42'13"S, Alt. 49 m, 2.4. 1993 leg. ORSTOM” (4 exs. NMW); “Betsiboka Bas. Manjakavaradrano, Mamokomita Riv., 46°54'20"E, 17°38‘00"S, Alt. 625 m, 16.4. 1991 leg. ORSTOM” (1 ex. NMW); “Betsiboka Bas., Ambalanbongo, Affl. de Betsiboka Riv., 47°00'30"E, 16°48'00"S, 30.3. 1993 leg. ORSTOM” (2 exs. NMW); “Betsiboka bas., Ambohimanalrika, Kamoro Riv., 47°10'06"E, 16°28'55"S, alt. 40 m, 1.4. 1993 leg. ORSTOM” (10 exs. NMW); “Betsiboka Bas., Ambohimanatrika, Kamoro Riv., 47°10'06"E, 16°28'55"S, 4.11. 1995 Elouard & Oliarinony leg.” (1 ex. NMW; habitus in Fig. 403); “Betsiboka Bas., Andriantoany Riv., 46°56'23"E, 17°19'40"S, 5.11.1995 Elouard & Oliarinony leg.” (1 ex. NMW); “Mandrare bas., Betanimena, Manananara Riv., 46°39'20"E, 24 48 17"S, alt. 118 m, 23.5. 1994 leg. ORSTOM” (3 exs. NMW); “Sahankazo Bas., 5 km au Nord de Antsandrangotika Riv. 49°23'46"E – 12°28'40"S, alt. 50 m, 4.4. 1994, leg. Edouard et Sartori” (1 ex. NMW); “Anove à Ivondro Bas., Tampolo, Affl. non nommé Riv. 49°25'40"E, 17°17'07"S, Alt. 8 m, 12.4. 1997, Gibon F. -M, Randriamasimanana D.” (3 exs. NMW); “Onilahy Bas., Ambatofotsy (Horombe), Aff. de Ihazofotsy Riv., 45°40'43"E, 22°30'49"S, 1.6. 1995, Elouard J-M.” (1 ex. NMW); “Onilahy Bas., Onilahy Riv., 44°33'53"E, 23°32'18"S, leg. ORSTOM” (1 ex. NMW); “Tsiribihina Bas., Antazoa, Manampanda Riv., 45°35'04"E, 20°21'40"S, Alt. 145 m, 29.5. 1996 Elouard & Sambatra leg.” (1 ex. NMW); “Antseranana distr., Sambirana Riv., Marovata vill. 5-12.12. 2001 Horak leg.” (2 exs. NMW); “Fianarantsoa Pr. Foret d’Analalava 29.6 km 280˚ W Ranohira, elev. 700 m, 1-5.2. 2003/22°35'30"S, 045°07'42"E, at light in tropical dry forest, Fischer, Griswold et al leg.” (1 ex. CAS); “Tollara Prov., Rés. privé Berenty, Foret de Bealoka, Mandraré Riv. 14.6 km 329˚ NNW Amboasary, elev. 35 m3-8.2. 2002/24°57'25"S, 46°16'17"E, at light in gallery forest, Fischer, Griswold et al leg.” (2 exs. CAS); “Maroansetra, Restaurant La Baquette d’or, because of light trap 20.12. 2006, N-15,42467, E49,73800, 12 m Isambert et al” (3 exs. NHRS); “Madagask., Kaudern / 19.1. / *Laccophilus posticus* Aubé det. Zimmermann” (1 ex. NHRS); same but “febr.” (1 ex. NHRS); same but “Tamatave / febr.” (1 ex. NHRS); “Tamatava distr., Andasibe J. Rolschik leg. 17-30.12. 2001” (1 ex. NMPC); “Katsepy (Majunga) 24-31.12. 1997 Pacholátko” (35 exs. NHMB, 8 exs. MZH); “Majunga, Cirque Rouge 22-23.12. 1997 Pacholátko” (1 ex. NHMB); “Moramanga env. 10-18.12. 1997 Pacholátko” (1 ex. NHMB); “Mangily N of Tulear 12.1. 2005 Bergsten” (5 exs. NHRS); “Kap Diego 1916 Friederichs S.G.” (1 ex. ZMHB); “Andranohinaly Voeltzkow S. / *Laccophilus posticus* Aubé det. Brancucci” (1 ex. ZMHB); “Res. Spec. Beza Mahafaly 44°32'E, 23°40'S, ca. 150 m, 18.5. 199/leg. Bartolozzi et al / *Laccophilus posticus* Aubé det. Rocchi 1991” (2 exs. CSR); “Madagascar, Fairmaire / *Laccophilus posticus* Aubé” (1 ex. NMW); “Madagascar, Fruhstorfer” (1 ex. NMW). – **Aldabra**: “Aldabra-Ins. N v. Madagascar 4.5. 1895 A. Voeltskow S.” (9 exs. ZMHB); “Ald. Atoll, 9°24'S, 46°20'E, Takamaka Camp 14.2. 1968 Shaffer J.C. / Black light / *Laccophilus posticus* Aubé det. Bameul 1986” (1 ex. USNM).

##### Diagnosis.

*Laccophilus posticus* and *Laccophilus continentalis* form a pair of species with considerable resemblance. The two species are separated by differences in shape of the penis, being distinctly more prominent in *Laccophilus posticus* than in *Laccophilus continentalis*; especially, anterior part is more extended in *Laccophilus posticus*. The penis of *Laccophilus posticus* and *Laccophilus continentalis* resembles also the penis of *Laccophilus rivulosus* but this species exhibits clear differences in the elytral colour pattern (irrorations are formed as longitudunal lines).

##### Description.

Body length 3.5–4.4 mm, width 2.0–2.5 mm. Dorsal, colouration of body as in Figs 402–405. Quite stable, although colour pattern exhibits some variation.

Head: Pale ferrugineous, posteriorly sometimes slightly darker. Submat, finely, evenly and distinctly microsculptured; reticulation indistinctly double. Large meshes rudimentary, weakly indicated and only in part discernible. Impunctate, except at eyes; with fine, scattered punctures.

Pronotum: Pale ferrrugineous. Frontally and basally in middle with rather vague dark ferrugineous to ferrugineous areas. Submat, finely, evenly and distinctly microsculptured; reticulation indistinctly double. Large meshes rudimentary, weakly indicated and only in part discernible. Impunctate, except anteriorly and laterally; here with fine, scattered punctures.

Elytra: Pale ferrugineous, with dark ferrugineous irrorations. Elytral colour pattern quite stable, exhibits some variation. Single dark marking sometimes only defined by its dark outlines while centre is of pale colour. Rarely the dark, longitudinal markings are in part mixed with each other forming a larger dark area (Figs 402–405). Submat, finely, evenly and distinctly microsculptured; reticulation indistinctly double. Large meshes rudimentary, weakly indicated and only in part discernible. Rows of punctures indistinct, indicated by fine, irregular punctures. Elytron with pre-apical lateral furrow, which is rather discrete and moderately pubescent.

Ventral aspect: Blackish ferrugineous to ferrugineous, prothorax pale ferrugineous to ferrugineous; no distinct colour pattern. Rather shiny, although very finely microsculptured. Abdomen with fine, curved striae. Almost impunctate. Metathorax with about 10 very fine, shallow and in part transversely located furrows. Prothorax moderatly broad, apex distinctly extended, apically pointed. Metacoxal process not distinctly modified. Apical ventrite (Fig. 49); symmetric and lacks lateral knob.

Legs: Pro- and mesotarsus slightly enlarged, provided with distinct suckers.

Male genitalia: Penis in lateral aspect prominent; apex extended to a sharp process (Fig. 250).

Female: Apical ventrite as in Fig. 50. Pro- and mesotarsus slender.

##### Distribution.

Mauritius, Madagascar, Aldabra (Fig. 536).

##### Collecting circumstances.

Insufficiently documented. Vinson (1956) reports the species to occur in stagnant water. On the other hand label data indicates that the species also occur in running water. Sometimes the species has been collected at light in forests (gallery forest and tropical dry forest). Obviously a lowland species the highest elevation for collection being 700 m a.s.l. *Laccophilus posticus* has also been recorded from rice paddies.

#### Laccophilus inobservatus sp. n.

Taxon classificationAnimaliaColeopteraDytiscidae

http://zoobank.org/A01A25B7-A2C0-4B3D-B810-315E70BB68CA

Figs 51–52 , 251 , 406–408 , 537

##### Type locality.

Chad: Near Bongor.

##### Type material

(234 exs.). Holotype: male: “Chad nr Bongor 27.5. 1973 R. Linnavuori” (MZH; habitus in Fig. 406). – Paratypes: Gambia: “Abuko Nature Reserve, at light at the bamboo pool 18.30-20.30, 18.11. 1977 UTM 28 PCK2181 Loc. 24 / Cederholm-Danielsson-Hammarstedt-Hedquist-Samuelsson” (1 ex. MZLU, 1 ex. NHMB); “Outside Abuko Nature Reserve at waterworks. At light 19.00-22.00, 26.2. 1977 Loc. No. 6 UTM 28 PCK 214812 / Cederholm-Danielsson-Hammarstedt-Hedquist-Samuelsson” (1 ex. MZLU); “Tendeba Camp at light in semiarid veg. near River Gambia 18.30-20.30, 14.11. 1977, UTM 28 POK1285 Loc. 12a / Cederholm-Danielsson-Hammarstedt-Hedquist-Samuelsson” (1 ex. MZLU); “3.5 km S Georgetown, Hilltop at Sankuli Kunda, alt. about 30 m, at light 18.30-20.15, 15.11. 1977 UTM 28PEK2593 Loc. 37 /-Danielsson-Hammarstedt-Hedquist-Samuelsson” (1 ex. MZLU); “2 km S Kitty, 7 km SSW Brikama Road junction. In and at Fresh Water Stream 13.11. 1977 UTM 28PCK 1761 Loc 7/-Danielsson-Hammarstedt-Hedquist-Samuelsson” (1 ex. MZLU, 1 ex. MZH, 1 ex. NHMB); “Gam Bathurst Jan. 68 Palm / *Laccophilus taeniolatus* Reg. det. Sven Persson” (4 exs. MZLU); “Bathurst Jan. 1968 T-E Leiler” (5 exs. NHRS, 1 ex. MZH); “Kuntaur NW Georgetown 21.11. 2003 B. Vondel / *Laccophilus taeniolatus* Régb. det. Rocchi 2004” (1 ex. CSR); Gambia: Bakau 6-26.11. 1984 leg. Palm / *Laccophilus taeniolatus* Rég. det. Sven Persson” (1 ex. MZLU); “Gambia Oil Palm and mangrove veg. close to the beach, about 5 km SSW Gunjur, at light 18.45-20.30 13.11. 1977 UTM 28PCK0554 Loc. 8 / Cederholm & al.” (1 ex. NHMB); “Gambia-southern Senegal 13°10'N 16°36'W stream N of Selety 19,2, 1976 M. Holmen” (1 ex. ZMUC). – Senegal: “70 km W Tambacounda 13°57.4'N, 14°15.9'E, 29.6. 2004 Halada leg.” (2 exs. NMPC); “Senegal, Parc National de Niokolo Koba 16.2. 1989” (1 ex. NHMB); “Senegal Cayare II. 46 A. Villiers” (1 ex. NHMB). – Mali: “NW Afr., K. Macina 10.11. 1973 D.R. Reynolds (C. O. R.R.) BM 1974-222” (1 ex. BMNH, 1 ex. MZH); “Kogoni X. 1966 G. Schmitz” (8 exs. MRAC, 2 exs. MZH); “Korioume, Niger Riv. 18.2. 2000 18°40'N, 3°00'W, leg. Komarek & Mayer / *Laccophilus taeniolatus* Régb. det. Wewalka 2001” (1 ex. NMW); “Markala Niger River, 13°40'N, 6°05'W, leg. Komarek & Mayer 9-4 / *Laccophilus taeniolatus* Régb. det. Wewalka 2001” (1 ex. NMW); “S Tombouctou 16°40'N, 3°00'W, 18.2. 2000 leg. Komarek & Mayer 18-1 / *Laccophilus taeniolatus* Régb. det. Wewalka 2001” (1 ex. NMW, 1 ex. MZH); “Mopti Niger Riv. 14°30'N, 4°12'W, 21.2. 2000 leg. Komarek & Mayer 21-2 / *Laccophilus taeniolatus* Régb. det. Wewalka 2001” (1 ex. NMW, 1 ex. MZH); “Goundaka, Bandiagara Riv. 14°29'N, 3°56'W, 12.2. 2000 leg. Komarek & Mayer 12-1 / *Laccophilus taeniolatus* Régb. det. Wewalka 2001” (2 exs. NMW) “SE Douna, Bani Riv. 13°13'N, 5°54'W, 10.2. 2000 leg. Komarek & Mayer 10-1 / *Laccophilus taeniolatus* Régb. det. Wewalka 2001” (1 ex. NMW). – Niger: “Nr. Boureimi 9.11. 1973 R. Linnavuori” (1 ex. MZH); “Rég. de Zinder, Sultanat de Damagherim Dungass, Mission Tilho Dr. R. Gaillard 1910” (6 exs. NHMB). – Sudan: “Blue Nile Ingessana Mts. 17-22.11. 1962 Linnavuori” (6 exs. MZH); “Nile Blue Nile Singa-Roseiras 15-17.11. 1962 Linnavuori” (1 ex. MZH); “Upper Nile Malakal 5-20.1. 1963 Linnavuori” (6 exs. MZH); “Kordofan Lake Keilak 8-11.2.1963 Linnavuori” (4 exs. MZH); “Bahr el Gazal, Wau 19.2. 1963 Linnavuori” (1 ex. MZH); “Bahr el Abiad Trägårdh / *Laccophilus taeniolatus* Régt var.” (1 ex. NHRS); “Torit 2.7. 1980 Armstrong” (1 ex. USNM); “Nairege River 27.2. 1980 Armstrong” (1 ex. USNM); “Gilo water tank (pumped up from stream) 20.3. 1980 Armstrong (8 exs. USNM, 3 exs. MZH); “Kinyetti River at Imeila 19.3. 1980 Armstrong” (5 exs. USNM, 1 ex. MZH); “Sudan Wad Medani am Bl. Nil, 18.10, 1979 lux, leg. Hieke” (1 ex. NHMB); same but “21.10. 1979” (1 ex. NHMB); same but “29.10. 1979” (1 ex. MZH); same but “12.10. 1979” (1 ex. NHMB); same but “30+31.10. 1979” (1 ex. NHMB); “Tombe 17.1. 1954 Omer-Cooper” (1 ex. AMGS). – Chad: Same data as holotype (5 exs. MZH); “Distr. Kanem N’Gouri X-XI. 1958 P. Renaud ex. coll. Breuning” (21 exs. MRAC, 2 exs. MZH). – Ethiopia: “Shoa, Awash NP, Filwoha Hot Springs 25.12. 1988, 1500 m leg. S. Persson / *Laccophilus taeniolatus* Régb. det. A. Nilsson” (2 exs. MZLU); “Shoa, Metahara 2.10. 1988 950 m, water hole in lava field, leg. S. Persson / *Laccophilus taeniolatus* Régb. det. A. Nilsson” (6 exs. MZLU, 2 exs. MZH; habitus in Fig. 407); “Hora Harsadi, Addas 7000 ft 2.12. 1926 J. Omer-Cooper” (1 ex. AMGS). – Burkina Faso:”Pundu Mte Volta1927-1928 Dez.-Juni Olsufiew” (2 exs. NHRS); “Pundu Olsufiew” (38 exs. NHRS, 3 exs. MZH); “Yatenga Gourcy Barrage 300 m 14.1. 1995 Trockengefallen lehmiges Ufer leg. B. Maier / *Laccophilus taeniolatus* Rég. det. Rocchi 2002” (1 ex. CSR). – Ivory Coast: “Comoé NP, N8,5°, W3,5° leg. N. Reintjes, det. F. Pederzani / 1.3. 1999 rock pool in Comoé river bed” (1 ex. NMW). “Comoé NP, N8,5°, W3,5° leg. N. Reintjes, det. F. Pederzani / 3.4. 1999 temporary Pond” (1 ex. NMW). – Ghana: “Upper E Prov. Navrongo env. 11-13.6. 2006 Pokorny leg.” (5 exs. NMPC, 1 ex. MZH). – Nigeria: “Kano St. Wudil-Kari 17.5. 1973 R. Linnavuori” (1 ex. MZH); “NW St. Gummi-Anka 24.7. 1973 R. Linnavuori” (1 ex. MZH); “NE St. Gombe-Bauchi 27.8. 1973 R. Linnavuori” (4 exs. MZH; habitus in Fig. 408); “Detritus pond, Jos-Bauchi rd 9.4. 1963 JOC”(4 exs. AMGS); “Samaru 17.5. 1959 W. Sands B.M. 1961-525 / Light trap” (1 ex. BMNH); “Marsk, road Katsina-Daura 6.4. 1963 JOC” (14 exs. AMGS). – Cameroon: “Maroua (lumière) X-XI. 1965 G. Schmitz” (1 ex. MRAC); “Maroua, Miss. Cath. 26.8. 73” (1 ex. NHMB); “Maroua 5 Aout 71” (1 ex. NHMB); “Tokombere, dint. Maroua, 12.7. 1979 Onore / *Laccophilus congener* O-C. det. Rocchi 1980” (1 ex. CSR). – Zaire: “P.N.G. Ndelele. K.117/14S, 19.3. 1952 H, De Saeger, 3199” (2 exs. MRAC). P.N.G. II/fd/13, 5.5. 1952 H, De Saeger, 3421” (1 ex. MRAC). – Non-African records: Yemen: “Wadi am Rija W Lahj Al Hulah by road, 13.01.57N, 44.33.30E (GPS) 25-26.10. 2007, 297 m a.s.l., Reitter leg.” (7 exs. NMPC, 2 exs. MZH).

##### Specimens with unclear labelling.

Two specimens in NHRS labelled “Egypten” also belong to this species. The material is fairly old and the exact location of these records is somewhat unclear. The specimens are therefore not included in type material.

##### Diagnosis.

*Laccophilus inobservatus* is closely related especially to *Laccophilus continentalis*, *Laccophilus simplicistriatus* and *Laccophilus taeniolatus*. The species can be distinguished by study of penis apex-shape, which is peculiar and stable in all four species. Penis apex of *Laccophilus inobservatus* is cut off straight and lacks any signs of anterior processes – resembling species have all at least minor kinds of modifications/processes on penis apex.

##### Description.

Body length 3.6–4.0 mm, width 1.9–2.2 mm. Colour pattern dorsally, reasonably uniform; rarely reduced so that elytral irrorations are in part fragmentary (Figs 406–408).

Head: Pale ferrugineous, lacks darker areas. Slightly mat, finely microsculptured. Reticulation double. Large meshes only slightly more strongly developed in comparison with small meshes. Large meshes may contain 2–6 small meshes. Impunctate, except at eyes, with fine, irregular punctures.

Pronotum: Pale ferrugineous. At foremargin between eyes with a dark ferrugineous to blackish, slightly vague marking. At base in middle with two narrow, blackish spots. Sometimes dark areas on pronotum may be reduced. Slightly mat, finely microsculptured. Reticulation double. Large meshes only slightly more strongly developed than small meshes. Large meshes may contain 2–6 small meshes. Impunctate, but at margins except basally in middle with very fine scattered punctures.

Elytra: Pale ferrugineous, with dense, blackish to dark ferrugineous irrorations. Irrorations generally quite evenly distributed; sometimes irrorations reduced and at least in part separate irrorations rudimentary. Posterior to middle irrorations can be strongly reduced forming an irregular pale spot on each elytron (Figs 406–408). Slightly mat, finely microsculptured; reticulation double. Large meshes only slightly more strongly developed than small meshes. Large meshes contain 2–6 small meshes. Punctures very fine, sparse and irregularly distributed; on disc, irregular punctures form a vague row of punctures. Pre-apical, lateral row of punctures form a shallow furrow provided with hairs.

Ventral aspect: Pale ferrugineous, abdomen distinctly darker; dark to blackish ferrugineous. Almost impunctate, except apical ventrite; with some, fine, irregular punctures. Apical ventrite lacks lateral knob (Fig. 51). Rather shiny although finely microsculptured; microsculpture in part indistinct and reduced. Ventrites with fine, slightly curved striae. Metacoxal plates with some 10 almost transversely located, shallow and in part reduced, furrows. Prosternal process slender, posteriorly distinctly extended, apex pointed.

Legs: Pro- and mesotarsus somewhat extended and enlarged, provided with distinct suckers.

Male genitalia: Extreme penis apex, blunt, abruptly broken and lacks any kinds of processes (Fig. 251).

Female: Pro- and mesotarsi slender. Apical ventrite as in Fig. 52.

##### Etymology.

The species name *inobservatus* is a Latin adjective meaning “unobserved”. It here refers to the peculiar situation that the species remained overlooked for a long time due to misinterpretation, although it is widespread and common.

##### Distribution.

Africa: Gambia, Senegal, Mali, Niger, Sudan, Chad, Ethiopia, Burkina Faso, Ivory Coast, Ghana, Nigeria, Cameroon, Zaire and Asia: Yemen (Fig. 537). Only personally verified records are included in the map. Egypt is also among examined material but exact location of this record is unknown and therefore record is not mapped.

##### Collecting circumstances.

Label data provide some information on the living habits of *Laccophilus inobservatus*. Accordingly, in Gambia collected at light at a bamboo pool and in semiarid vegetation near a river. Moreover the species has been collected in and at a fresh water stream and in a rock pool in river bed.

#### Laccophilus simplicistriatus

Taxon classificationAnimaliaColeopteraDytiscidae

Gschwendtner, 1932

Figs 53–54 , 252 , 409 , 538

Laccophilus simplicistriatus Gschwendtner 1932b: 260 (original description, faunistics); Gschwendtner 1935a: 16, 17, 18 (description, faunistics); Gschwendtner 1938a: 5 (faunistics); Guignot 1943: 99 (faunistics); Guignot 1946c: 271, 273, 279, 280, 281, 312 (faunistics, biology); Guignot 1948: 15 (faunistics); Balfour-Browne 1950: 360 (faunistics); Guignot 1951: 215 (faunistics); Guignot 1952a: 533 (faunistics); Guignot 1952c: 522 (faunistics, biology); Guignot 1953b: 234 (faunistics); Legros 1954: 268 (faunistics); Guignot 1954: 26, 27 (faunistics, discussion); Guignot 1956b:219 (faunistics); Omer-Cooper 1956: 21 (faunistics, biology); Omer-Cooper 1957: 20, 21 (description, discussion); Omer-Cooper 1958b: 37, 43, 45 (description, discussion, faunistics, biology); Legros 1958: 211 (faunistics); Guignot 1959a: 570, 571, 573 (description, faunistics); Omer-Cooper 1965: 81, 82 (description, discussion, faunistics); Omer-Cooper 1970: 289, 290, 291 (description, discussion); Legros 1972: 466 (faunistics); Bilardo and Rocchi 1987: 104 (faunistics, biology); Curtis 1991: 186 (faunistics); Nilsson and Persson 1993: 81, 94 (discussion, biology); Nilsson 2001: 251 (catalogue, faunistics); Nilsson 2015: 218 (catalogue, faunistics).
Laccophilus monas Guignot 1953a: 238 (original description, faunistics); Guignot 1954: 27 (description, faunistics); Guignot 1959a: 571 (list as synonym of *Laccophilus simplicistratus*); Guignot 1959d: 162 (discussion, faunistics); Omer-Cooper 1965: 82 (list, synonymy); Nilsson 2001:251 (catalogue, list, synonymy, faunistics); Nilsson 2015: 218 (catalogue, list, synonymy, faunistics). **Confirmed synonym**.

##### Type localities.

*Laccophilus simplicistriatus*: Zaire: Lusindol.

*Laccophilus monas*: Zaire: Route Shangugu-Usumbura, riv. Lua.

##### Type material studied

(12 exs.). *Laccophilus simplicistriatus*: Lectotype (by present designation): male: “Paratypus / Musée du Congo Lusindol 15-VIII-1911 L. Burgeon / R. Det. 2093 C” (MRAC; habitus in Fig. 409). – Paralectotypes: Same data as lectotype but “7-VIII-1911” (1 ex. MRAC); “Paratypus / Musée du Congo Albertville 20-X-1925 Dr. H. Schouteden / R. Det. 2093 C” (1 ex. MRAC); “Paratypus / Musée du Congo Karemi V-1912 Dr. Bayer / R. Det. 2093 C / *Laccophilus simplicistriatus* Gschw. det. Gschwendt.” (1 ex. MRAC); same data as preceding but no determination label (1 ex. MRAC); “Musée du Congo Haut Uelé: Moto 1920 L. Burgeon / R. Det. 2093 C” (2 exs. MRAC); “Musée du Congo Riv. Lobozi 5.11. 1912 Dr. Stappers 1548/ R. Det. 2093 C” (1 ex. MRAC); “Musée du Congo Bavrengura Haut Uelé L. Burgeon / R. Det. 2093 C” (1 ex. MRAC); “Musée du Congo Katanga: Katompe 1/15-VI-1930 Dr. P. Gerard / R. Det. 2093 C” (1 ex. MRAC); “Musée du Congo Kil. 345 de Kindu, nuit Dr. Russo / R. Det. 2093 C” (1 ex. MRAC). [Comments: no clarification exists why five of the specimens above have earlier been provided with a paratype label? Regarded as a case of mislabeling.]

*Laccophilus monas*: Holotype: male: “Holotypus / I.R.S.A.C. –Mus. Congo/Route Shangugu-Usumbura riv. Lua 5-VIII 1949 G. Marlier / Type / Eaux thermals rivier Lua 5-8-49/R Dét. I. 6182 / Guignot det., 1953 *Laccophilus monas* Guign. Type, male/= *simplicistriatus* Gschw. det. J. Omer-Cooper May 25^th^ 1954” (MRAC).

##### Additional material studied

(396 exs.). **Sudan**: “Meya Saku 43 mi. from Amadi, Juba rd. 29.I. 1954 JOC” (2 exs. AMGS); “L. Yirol 6,33N, 30,3E 24.I. 1954 JJOC” (2 exs. AMGS); “Nimule, Fula rapids 4.XI. 1954 JOC.” (1 ex. AMGS); “Nimule Ferry 4.XI. 1954 JJOC.” (3 exs. AMGS); “L. Nyibor 23.I. 1954 JJOC.” (3 exs. AMGS); “Sandy river 50 mi. NW of Juba JJOC.” (5 exs. AMGS); “Aluakluak 30,5E, 6,30N 15.IV. 1954” (2 exs. AMGS); “Stream from hot springs Nyangwara 30,5E, 4,39N 29.I. 1954 JJOC.” (3 exs. AMGS); “Equatoria Tali Post 8.IV. 1954” (1 ex. AMGS); “L. Shambe 21.I. 1954 JJOC.” (3 exs. AMGS); “Upper Nile, Malakal 5-20.1. 1963 Linnavuori / ad lucem” (4 exs. MZH); “Upper Nile, Malakal 5-20.1. 1963 Linnavuori” (10 exs. MZH); “Upper Nile Malakal Linnavuori” (1 ex. MZH); “Upper Nile Pr. Malakal, nr junction Nile – Sobat 21.9. 1957 Forsberg/*Laccophilus simplicistriatus* Gschw. det. Nilsson 1996” (1 ex. MZLU); “Blue Nile Ingessana Mts. 17-22.11. 1962 Linnavuori” (2 exs. MZH); “Weisser Nil bei Tonga 10-13.4. 1914 Ebner”(1 ex. NMW); “Mongalla 50 Werner” (2 exs. NMW); “Kadugli at light, 11. 1954 Sweeney” (1 ex. BMNH); “Torit 2.7. 1980 Armstrong” (11 exs. USNM, 2 exs. MZH); “Gilo water tank (pumped of from stream) 20.3. 1980 Armstrong” (13 exs. USNM, 3 exs. MZH); “Kinyetti Riv. at Imeila 19.3. 1980 Armstrong” (4 exs. USNM); “SW Sudan nr Yambio Abbott” (1 ex. USNM); “Nairege Riv. 27.2. 1980 Armstrong” (1 ex. USNM). – **Ethiopia**: “Arsi, Dehra 40 km N Assella 25.9. 1988 1800 m, temp pool, Persson / *Laccophilus simplicistriatus* Gschw. det. Nilsson” (2 exs. MZLU); “Shoa, Soddere 16.10, 1988, 1500 m, temp. pool without vegetation Persson / *Laccophilus simplicistriatus* Gschw. det. Nilsson” (1 ex. MZLU); “Shoa, Soddere 25.9. 1988, 1500 m Persson / *Laccophilus simplicistriatus* Gschw. det. Nilsson” (2 exs. MZLU); “Shoa Dobre Zeit Hora lake 15.3. 1989, 2200 m, polluted water Persson /*Laccophilus simplicistriatus* Gschw. det. Nilsson” (1 ex. MZLU); “7000 ft. Hora Harsadi Addas 2.XII. 1926 J. Omer-Cooper” (1 ex. AMGS); “West marsh L. Zwai 5500 ft. 2-3.XI. 1926 JOC.” (2 exs. AMGS); 5000 ft. small pond Hora Shala 21.XI. 1926 JOC.” (1 ex. AMGS); “7000 ft. Mt. Chilalu 8.XI. 1926 JOC.” (2 exs. AMGS); “Baher Dar 8.10.1968 Horde leg. /Lichtfang” (7 exs. NHMB, 2 exs. USNM); “Bahar Dar, at light 4.4. 1967 P. Stys leg.” (1 ex. NMPC). – **Zaire**: “Kigoma V. 1930 / Paratype / *Laccophilus simplicistriatus* Gschw. det. Gschwendtner” (1 ex. OLML; not type material); same as preceding but no determination label (1 ex. MRAC; not type material); “Karemi V- 1912 Dr. Bayer / *Laccophilus simplicistriatus* G. J. Balfour-Browne det. 1963” (3 exs. MRAC); “Riv. Lobozi 5.11.1912 / *Laccophilus taeniolatus* Rég. var. R. Peschet det. 1914“ (1 ex. MAC); “Kivu: Luvungi XII-1932” (2 exs. MRAC); “Elisabethville (a la lumière) X/XI-1950 / *Laccophilus simplicistriatus* Gschw. det. Guignot 1953” (1 ex. MRAC); “PNG I/c/4, 15.3. 1950 Demoulin 234 / Paratype / *Laccophilus monas* Guign. det. Guignot” (1 ex. IRSNB; not type material); “PNG Napokomweli 18.X. 1950 G. Demoulin 893 / *Laccophilus monas* Guign. det. Guignot 1957” (1 ex. AMGS); “PNG, I/a/2, 21.4. 1950 Demoulin 452” (1 ex. NHMB); “PNG, Ndelele 19.3. 1952, 3199” (1 ex. MRAC); “PNG II/fd/12, 10.3. 1952, 3180” (2 exs. MRAC, 1 ex. MZH); “PNG PpK/14/g/14s, 4.4. 1952, 3290” (3 exs. MRAC, 1 ex. MZH); “PNG II/fd/14s, 3.4. 1952, 3278” (1 ex. MRAC); “PNG II7fd/Gar 29.2. 1952, 3152” (1 ex. MAC). – **Uganda**: “Mabira Forest Tinga 19.7. 1970 Brown” (1 ex. BMNH). – **Kenya**: “Lambwe Valley on light 11.6.1974 van Etten” (1 ex. RMNH); “Aberdares NP 5.12. 1989 Jäch” (1 ex. NMW); “Thika 7.12. 1989 Jäch” (2 exs. NMW, 1 ex. MZH); “Meru Distr., Gatunga 5.4. 1987 Mourglia” (1 ex. NHMB). – **Rwanda**: “Rumonge, Regenwald Jan. 1986 Heiss” (1 ex. NHMB). – **Tanzania**: “Ukerewe Tang. Terr. VIII. / *Laccophilus simplicistriatus* Gschw. det. Gschwendtner” (4 exs. OLML); “TPC S of Moshi canals 28.9. 1976 Holmen” (1 ex. ZMUC); “Mwanza nr. Lake Victoria 31.7. 1957 / sweet potato channels” (2 exs. BMNH); “Mwanza nr. L. Victoria 1957 / Marginal pools and ditches” (7 exs. BMNH); “Tanganyika 1959 Eccles” (1 ex. BMNH); “SW Tanganyika Mpanda (dans ruisseau) 6. 1960 Leleup” (1 ex. MAC); “T.T. Rukwa Milepa 25.4. 1951 Water in road- tracks Backlund / *Laccophilus simplicistriatus* Gschw. det. Nilsson 1996” (1 ex. MZLU); “T.T. Rukwa Tumba 29.1. 1951 Backlund / *Laccophilus simplicistriatus* Gschw. det. Nilsson 1996” (1 ex. MZLU); “T.T. Rukwa Tumba 12.1. 1951 T. river Backlund / *Laccophilus simplicistriatus* Gschw. det. Nilsson 1996” (1 ex. MZLU); “T.T. Rukwa Kipangati 28.11. 1950 sulphurous pools, shallow in rich woodland Backlund / *Laccophilus simplicistriatus* Gschw. det. Nilsson 1996” (1 ex. MZLU); “Tang. terr. Nzega, Naro 19.8. 1951 Backlund / *Laccophilus simplicistriatus* Gschw. det. Nilsson 1996” (1 ex. MZLU); “Rukwa, Rungwa Riv. 18.5. 1950 Backlund / *Laccophilus simplicistriatus* Gschw. det. Nilsson 1996” (1 ex. MZLU); “T.T. Shinyanga 21.2. 1935 Burtt” (1 ex. BMNH); “T.T. Rukwa Mkumbwa 12.5. 1950 Backlund / *Laccophilus simplicistriatus* Gschw. det. Nilsson 1996” (1 ex. MZLU); “Iringa Prov., 100 km NE Iringa 07°37'S, 36°17'E, 9.1. 2007, 660 m, J. Halada leg.” (2 exs. NMPC, 1 ex. MZH); “Mbeya prov., 120 km E Mbeya 08°51'S, 34°00'E, 1220 m, 6.1. 2007 m, J. Halada leg.” (1 ex. NMPC). – **Angola**: “Namakunda 6. 1948, 16.15E, 18.50 S Koch” (13 exs. BMNH, 1 ex. MZH); “Namakunda 6. 1948, 16.15 E. 1850S, Koch” (11 exs. BMNH); “Mongua 4.6. 1954, shallow reedy vlei” (1 ex. BMNH); “Mossamedes Distr., Rio Coroca 23.6. 1954/small clear pool with *Chara”* (1 ex. BMNH); “Rio Coroca 8 m. N of Porto Alexandre 22-23.6. 1954/Pond with Algae & *Lemna*, fringing *Juncus”* (1 ex. BMNH); “Pediva, ca, 30 mi. E of Porto Alexandre 400 ft. 26-27.6. 1954 / Ponds in warm, saline river; thick weed” (1 ex. BMNH); “Angola Schönlein” (1 ex. ZMHB). – **Zambia**: “Central Pr. Lusaka 8.1. 1982 Selander / rain pond” (1 ex. MZH); ”29.3.1993. Kafue NP., Chunga Camp, 15°02'35"S/26°00'09"E, lux Uhlig” (1 ex. ZMHB). – **Malawi**: “Stream 20 mi. from Dedza on Lower Lilongwae rd 30.IX. 1948” (8 exs. AMGS); “R. Diedma Lilongwe rd 30.IX. 1948” (1 ex. AMGS); “Bua R. 2.X. 1948 JOC.” (1 ex. AMGS); “Dallys Hotel nr. Ft. Johnstone 23.VIII. 1948” (3 exs. AMGS); “Zomba plateau res.7.XI. 1948” (3 exs. AMGS); “Stream, Zomba plateau 6000 ft. 7.XI. 1948” (1 ex. AMGS); “Gomba plateau (?) 7.XI. 1948” (3 exs. AMS); “Stream 6 mi. N of R. Mtiti 2.X. 1948” (1 ex. AMGS); “Swampy pool nr. L. Nyasa 9.6. 1946” (1 ex. BMNH). – **Zimbabwe**: “Stream at Salisbury17.IX. 1948” (2 exs. AMGS); “Marandellas 2. XI. 1948 JOC.” (2 exs. AMGS); “Small stream nr. Halfway Hotel Salisbury-Gatooma 14.IX. 1948” (2 exs. AMGS); “Wankie Reserve water holes 3.IX. 1948” (5 exs. AMGS); “Wankie Game Res. IX. 1948 water holes” (10 exs. AMGS); “Wankie Game Res. IX. 1948 water hole / *Laccophilus simplicistriatus* Gschw. det. Omer-Cooper” (3 exs. AMGS); “Wankie Game Res. Mazume Dam 4.IX. 48 / *Laccophilus simplicistriatus* Gschw. det. Omer-Cooper” (5 exs. AMGS); “Wankie game reserve, Shapi pan 5-6.IX. 1948 (7 exs. AMGS); “Wankie Game Reserve 4.9. 1948 / J. OmerCooper” (1 ex. BMNH); “Wanki Game Reserve 4-5.9. 1948 J. Omer-Cooper / *Laccophilus simplicistriatus* Gschw. det. J. Omer-Cooper”(4 exs. NHMB, 9 exs. USNM); ”5 mi SE Wankie 7.4. 1968 Spangler” (9 exs. USNM, 3 exs. MZH); “Victoria Falls rainforest 6.X. 1948” (2 exs. AMGS); “Gwai River 3.4. 1968 Spangler” (7 exs. USNM, 1 ex. MZH). – **Mozambique**: “Niassa Prov., S12°17'28.8”, E34°46'31.4” Mandambuzi Marsh, Watson 6.4. 2009” (1 ex. CGF); “Niassa Prov. Cmimulimuli River, S12°11.520’, E34°42.288’, Watson 10.2. 2008” (1 ex. CGF). – **Namibia**: “Windhoek Town Dam 7.VII. 39” (1 ex. AMGS); “Okahandja Distr. Toggekry 250, Omatako Ranch, 55 km NNW, NNW Okahandja, thornbush savannah / 7.2. 2001, 21°30'43"S/16°43'00” lux 22°-14°, 25.4. 2001, 17,45-20,00 Uhlig & Ebert” (1 ex. ZMHB, 1 ex. NMNW, 1 ex. MZH); “Damaral. Okahandja 21.59S-16.52E / 12.9. 1974 shore washing, Endrödy-Younga” (2 exs. TMSA, 1 ex. MZH); “Damaraland Oshikango 5. 1948 15.55 E 17.25 S Koch” (1 ex. BMNH); “Kavango:Popa Falls 18°07'S-21°35'E, 26.2.-3.11.1992 lux Uhlig leg.” (1 ex. ZMHB); “Ovamboland Namutoni 31.5. 1954 / weedy waterhole and stream” (16 exs. BMNH, 2 exs. MZH); “Oshikango SE of frontier post 2.6. 1954/shallow water, svampy marsh” (27 exs. BMNH, 2 exs. MZH); “Ca. 7 mls N.E. of Grootfontein / waterhole in dolomite” (4 exs. BMNH); “Okarupa, ca. 17 mi. E of Okahandja, 4900 ft, 22.5. 1954 / pools in overflow stream from dam, much weed & algae” (2 exs. BMNH); “Kro, ca. 15 mi. SE of Namutoni 30.5. 1954 / shallow & muddy with algae” (1 ex. BMNH); “Etosha Pan Okaukujo camp 19.11S-15.55E/28.12. 1974 shore washing Endrödy-Younga” (1 ex. TMSA); “Etosha Pan, 60 m. NW Namutoni 5. 1937”(1 ex. TMSA); “Etosha Game Res., Namutoni 27.5. 1937” (3 exs. TMSA, 1 ex. MZH); “Kaokoweld Kowares, 90 mi SE Ohopoho 3.6. 1951” (1 ex. MZLU); “Kaokoweld Sanitatas, abt 85 mi. WSW Ohopoho 14-16.6. 1951” (1 ex. MZLU); “Kaokoweld Anabib (Orupembe) 100 mi. W Ohopoho 12-13.6. 1951” (1 ex. MZLU); “Kaokowelld Omutati, 70 mi. WSW Ohopoho 5.6. 1951” (2 exs. MZLU); “Kaokoweld 17.10. 1963 Gaerdes” (1 ex. MZLU); “Kaokoweld Sesfontain, 17 km WSW, 19.12S-13.32E/1.2. 1975 singled in riv. bed, Endrödy-Younga” (1 ex. TMSA); “Distr. Grootfontein leg. Irish / Farm klein Nosib 19.28S-14.50E Anfang April 1989” (1 ex. ZMHB); “Okomite R, temp. pool, N-17.4305, E14.1666, 12.11. 1997 De Moor” (5 exs. AMGS); “Omapapurawe Guard Post, 200 m from campsite, Kunene R., N-17.218, E13.645, 15.11. 1997 Bethune & al.” (3 exs. AMGS); “Kunene R., stream from cave into pool, N-17.00.07, E12.59.54, 20.6. 1997 De Moor & al.” (1 ex. AMGS). – **Botswana**: “Tsotsorogo Pan 17.VI-9. VIII. 1930 / Type male / female / paratype / *Laccophilus simplicistriatus* Gschw. det. Gschwendtner” (2 exs. AMGS, 2 exs. TMSA, 1 ex. OLML; not type material); “Chobe Park Savuti Camp11.3. 1993, 18°33'S/24°03'E, lux Göllner” (1 ex. ZMHB); ”5 km NW San-ta-Wani Safari Lodge 19°27'01”/23°38'46”lux, Uhlig” (1 ex.ZMHB). – **South Africa**: “Trsvl 5 mi W Warmbad 24-25.2. 1968 Spangler” (1 ex. USNM); “Trsvl Randburg, N-26.070, E27.950, 6.6.1971 Reavell” (1 ex. AMGS); “Caffraria / J. Wahlb.” (1 ex. RMS); “Gauteng Cullinan Premier Mine Res. 25,40S–28,29E / 17.1.2002 Endrödy-Younga, light trap” (1 ex. TMSA); “Gauteng Tswaing 25.24S-28.06E / 16.2. 2003 light trap, TMSA staff leg.” (1 ex. TMSA); “Roodeplaat Pretoria distr. 10. 1960 Neubecker” (1 ex. TMSA); “Xolo R, small stream, riverbed, trib. Kunene R, 15.11. 1997 De Moor” (1 ex. AMGS). – **Swaziland**: “Eranchi 5-10.1. 1955 Capener / *Laccophilus simplicistriatus* J. Omer-Cooper det.” (3 exs. MZH). – **Lesotho**: “Nazareth M. S., 20 mi.ESE Maseru 24.3. 1951” (1 ex. MZLU).

##### Specimen with uncertain determination.

**Cameroon**: female “Yaounde, Bor to Kosti by boat 13-14.3. 1978 Perkins” (1 ex. USNM).

##### Specimen with uncertain labelling.

**Mauritius**: male “Insel Mauritius Westw. Nr. 9984 / *Laccophilus simplicistriatus* Gschw. det. Brancucci 82” (1 ex. ZMHB). Until additional specimens from Mauritius are available, this record is considered a case of mislabelling.

##### Comments on synonymy.

Confusion regarding the original description and the type material of *Laccophilus simplicistriatus* followed when Gschwendtner (1932b) in a faunistic paper listed and mentioned *Laccophilus simplicistriatus* and at the same time he provided a short description of the species. Originally this act was not ment to be the original description, which it is in fact. Accordingly, the report (Gschwendtner 1932b) is the original description and not the later publication of Gschwendtner (1935a). Designation of type in the later article is accordingly invalid. A lectotype has been chosen from the valid type material of *Laccophilus simplicistriatus* and it has been compared with the holotype of *Laccophilus monas*. This examination confirms earlier proposed synonymy of the two taxa.

##### Diagnosis.

*Laccophilus simplicistriatus* externally resembles most of *Laccophilus taeniolatus* and *Laccophilus complicatus*. From *Laccophilus taeniolatus* it is distinguished by elytral irroration, which is complete (uniform coverage) and not reduced frontally at suture. In *Laccophilus taeniolatus* elytral irroration frontally at suture is always sparser, often formed as a pale somewhat irregular spot. Shape of penis apex exhibits differences separating *Laccophilus simplicistriatus* from *Laccophilus complicatus* (apex of penis sharp and strongly curved) as well as from all other *Laccophilus* species.

##### Description.

Body length 3.6–4.3 mm, width 1.9–2.4 mm. Habitus, dorsal aspect as in Fig. 409. Dorsal colour pattern quite uniform exhibiting only minor variation.

Head: Pale ferrugineous. Finely microsculptured; reticulation indistinctly double. In part, coarser meshes not discernible. When visible one large mesh contains 2–6 fine meshes. At eyes with fine, irregular punctures; a few scattered punctures medially between eyes.

Pronotum: Pale ferrugineous, anteriorly and posteriorly in middle with narrow ferrugineous to dark ferrugineous area. At margins, except basally in middle, with fine, irregular punctures. Microsculpture fine; reticulation indistinctly double (only in part, discernible). Slightly coarser, large meshes contain 2–6 fine meshes.

Elytra: Pale ferrugineous, with quite coarse and distinct, dark ferrugineous irrorations (Fig. 409). Irrorations laterally sometimes slightly reduced and less distinct than at suture. Slightly mat, finely microsculptured; double reticulation indistinct, only in part discernible; coarser meshes extensively rudimentary and hardly visible. Very fine, sparse punctures laterally and discally (form an irregular, discal row of punctures) discernible. Lateral, pre-apical furrow fine, finely pubescent.

Ventral aspect: Ferrugineous, apical half of abdomen dark ferrugineous. Rather shiny, although extensively, very finely microsculptured. Almost impunctate. Metacoxal plates with some transversely located, shallow furrows. Abdomen with fine striae. Prosternal process narrow, apically pointed. Apical ventrite not distinctly asymmetric (no process or knob on one side) (Fig. 53).

Legs: Pale ferrugineous, hindlegs a little darker. Pro- and mesotarsus rather slender, claws not especially long, slightly curved. Pro- and mesotarsus with suckers.

Male genitalia: Penis in lateral aspect, with apical half, somewhat enlarged on both sides of apex; extreme apex with two small processes (Fig. 252).

Female: Externally as male but apical ventrite less impressed on both side of midline and apex more extended backwards and rounded (Fig. 54).

##### Distribution.

Sudan, Ethiopia, Zaire, Uganda, Kenya, Rwanda, Tanzania, Angola, Zambia, Malawi, Zimbabwe, Mozambique, Namibia, Botswana, South Africa, Swaziland, Lesotho (Fig. 538). Uncertain female record from Cameroon. One male record from Mauritius is considered a probable case of mislabelling.

##### Collecting circumstances.

Very little, detailed information is available on the biology of *Laccophilus simplicistriatus*. In Malawi *Laccophilus simplicistriatus* was e.g. collected in a reservoir surrounded by a marshy area with small areas of open water and red mud and from a swift river with a wide coarse gravel bed where the species was collected in a pool. Additionally from Malawi, the species was sampled in a clear river with white water lilies, reed beds and patches of swamp. In Zimbabwe the species was collected in a number of water holes, springs and dams used by the game, and also in a dam with reeds and water plants, the bottom largely covered with dark mud, but sand in places. Finally, also in Zimbabwe collected in a series of pools with a small stream connecting them. The bottom of the water body was rock, sand and gravel with some deposited mud. Some vegetation growing in the pools. Also recorded from streams (Omer-Cooper 1958b). Data of collection labels is also rather scarce and simply describe method of sampling or kind of water body; light collection or temporary pool, stream from hot spring, pond in warm, saline river etc.

#### Laccophilus taeniolatus

Taxon classificationAnimaliaColeopteraDytiscidae

Régimbart, 1889

Figs 56–57 , 253 , 410–412 , 539

Laccophilus taeniolatus Régimbart 1889: 52 (original description, faunistics); Severin 1892: 472 (incorrect type deposition); Régimbart 1894: 237 (description, faunistics); Régimbart 1895: 136, 137 (description, discussion, faunistics); Sharp 1904: 3 (faunistics); Régimbart 1904: 66 (faunistics); Régimbart 1906: 248 (description variation, faunistics); Régimbart 1908: 5 (faunistics); Zimmermann 1919: 122 (faunistics); Zimmermann 1920a: 26 (catalogue, faunistics); Peschet 1920: 250 (discussion, faunistics); Régimbart 1922: 532 (faunistics); Peschet 1922: 374 (faunistics); Zimmermann 1926: 23 (faunistics, description); Gschwendtner 1930: 88 (faunistics); Gschwendtner 1931: 180 (faunistics); Omer-Cooper 1931: 757 (biology, description, faunistics); Gschwendtner 1932b: 260 (discussion); Gschwendtner 1935a: 16, 17, 18 (description, discussion, faunistics); Guignot 1943: 99 (faunistics); Guignot 1946c: 271, 273, 278, 280, 312 (description, discussion, faunistics); Guignot 1951: 215 (faunistics); Guignot 1952a: 533, 535 (discussion, faunistics); Capra 1952: 6–7 (faunistics); Guignot 1954: 29 (faunistics); Guignot 1955d: 67 (discussion); Guignot 1955g: 865 (faunistics); Guignot 1956b: 219 (faunistics); Omer-Cooper 1957a: 20, 21 (description, discussion, incorretc association); Omer-Cooper 1958b: 43, 45 (description, discussion, incorretct assocation); Guignot 1959a: 570, 573, 574, 575 (description, faunistics, incorrect association); Bruneau de Miré and Legros 1963: 874, 883 (faunistics); Omer-Cooper 1965: 81, 82 (list); Omer-Cooper 1970: 288, 289, 290 (description, discussion); Legros 1972: 466 (faunistics, list.); Medler 1980: 155 (faunistics, list.); Forge 1981: 500, 501 (description, faunistics); Nilsson and Persson 1993: 58, 81, 88, 94 (biology, discussion, faunistics); Nilsson et al. 1995: 506 (discussion, faunistics); Nilsson 2001: 251 (catalogue, faunistics); Bilardo and Rocchi 2002: 174 (faunistics, list); Reintjes 2004: 68 (faunistics); Aistleitner and Jäch 2014: 47, 49 (faunistics, biology); Nilsson 2015: 218 (catalogue, faunistics). [Comment: This species was quite early misinterpreted and accordingly, any information in articles listed above on *Laccophilus taeniolatus* should be considered carefully before acceptance.]
Laccophilus congener Omer-Cooper 1957a: 19, 21, 90 (original description, discussion, faunistics); Omer-Cooper 1958a: 59 (faunistics); Omer-Cooper 1958b: 37, 43, 44, 45, 46 (biology, description, discussion, faunistics); Guignot 1959a: 570, 571, 573 (biology, description, discussion, faunistics); Omer-Cooper 1962: 295 (faunistics); Omer-Cooper 1965: 76, 81, 82 (description, discussion, faunistics); Omer-Cooper 1970: 288, 289, 290 (description, discussion); Bilardo and Pederzani 1978: 119 (description, faunistics); Medler 1980: 155 (faunistics, list); Pederzani and Rocchi 1982: 72 (faunistics); Bilardo 1982a: 447 (description, faunistics. Spelled *Laccophylus*); Pederzani 1988: 107 (biology, faunistics); Bilardo and Rocchi 1990: 162, 177 (biology, faunistics); Franciscolo and Sanfilippo 1990: 145 (biology, description, faunistics); Curtis 1991: 186 (faunistics); Nilsson et al. 1995: 506 (faunistics); Rocchi 2000: 24 (faunistics); Nilsson 2001: 242 (catalogue, faunistics); Bilardo and Rocchi 2002: 156, 161, 162, 174 (faunistics, list); Reintjes 2004: 67 (faunistics, list); van Vondel 2005: 130 (biology, faunistics); Bilardo and Rocchi 2006: 130 (faunistics); Bilardo and Rocchi 2013: 141 (faunistics, biology); Nilsson 2015: 210 (catalogue, faunistics). **New synonym.**

##### Type localities.

*Laccophilus taeniolatus*: Angola: Humpata.

*Laccophilus congener*: South Africa: Transvaal, Belfast.

##### Type material studied

(12 exs.). *Laccophilus taeniolatus*: Holotype: male: “P.J. vd Kellen Humpata Afr. trop. / *Laccophilus taeniolatus* sp. n. type Régb. / *taeniolatus* sp. n. Régimb.” (RMNH; habitus in Fig. 410). [Comment: type material contains only one specimen.]

*Laccophilus congener*: Holotype: male: “Type / Transvaal Belfast pond 23. N. 1948 Omer-Cooper / Brit. Mus. 1957-660 / *Laccophilus congener* O-C.” (BMNH). – Paratype: female: Same information as holotype but labelled as “Allotype” (1 ex. BMNH); additional paratypes: same information as holotype but labelled as “Paratype” (4 exs. AMGS); “Paratype / Transvaal Belfast 30 Dec. 1948 J. Omer-Cooper” (1 ex. AMGS); “Paratype / Transvaal Standerton 8.12. 1948 J.O-C. /*Laccophilus congener* O-C. det. J. Omer-Cooper” (1 ex. AMGS); “Paratype / Transvaal R. Nyl at Num Num 23 Aug. 1948 J.O-C. (1 ex. AMGS); Paratype / Transvaal Misselburg 29 N. 1948” (2 exs. AMGS).

##### Specimens with type status uncertain

(not given in original description) (2 exs.): “Paratype / Transvaal Deel Kraal 20.8. 1948 J. O-C.” (1 ex, AMGS); “Paratype / Transvaal Poerzya R., Waterberg Distr. 19.8. 1948” (1 ex. AMGS).

##### Additional material studied

(457 exs.): **Gambia**: “Stream N of Selety 19.2.1976 Holmen leg.” (2 exs. ZMUC, 1 ex. MZH); “Bathurst Jan. 1968 Leiler T.” (1 ex. NHRS); “Outside Abuko Nature Reserve at water works, at light 18.30–21.00, 4.11. 1977 / Cederholm-Danielsson-Hammarstedt-Hedquist-Samuelsson” (1 ex. MZLU); same data but ”19.00–22.00, 26.11. 1977” (1 ex. NHMB); same data but ”in and at Lamin stream 25–26.11. 1977” (1 ex. NHMB); “Abuko Nature Reserve, at light at the bamboo-pool 18.30-20.30, 18.11. 1977 / Cederholm-Danielsson-Hammarstedt-Hedquist-Samuelsson” (1 ex. MZLU, 1 ex. NHMB); “2 km S Kitty, 7 km SSW Brikama rd., junction in and at fresh water stream 13.2. 1977 / Cederholm-Danielsson-Hammerstedt-Hedquist-Samuelsson” (1 ex. MZLU). – **Senegal**: “Swamps ca. 3 km SW of Ziguinchor 8.3. 1977 / Cederholm-Danielsson-Larsson-Mireström-Norling-Samuelsson” (1 ex. MZLU); “In forest 1,5 km NE Djibélor, ca 6.5 km SW Ziguinchor, 8.3. 1977, at light 19.00-21.30 / Cederholm-Danielsson-Larsson-Norling-Samuelsson” (1 ex. MZLU); “Village Saré Sara 21 km ESE Kolda, in and at the junction of Rivers Koring and Tiángol, Dianguina 6.3. 1977 6.3. 1977 / Cederholm-Danielsson-Larsson-Norling-Samuelsson” (1 ex. MZLU). – **Guinea Bissau**: “Cacheu, 12 km E Varela, earth pit pond 9.4. 1993 S. Persson leg.” (8 exs. MZLU); “Cacheu, Bula, temporary pools 16.7. 1992 S. Persson leg.” (2 exs. MZLU); “Cacheu, 5 km W Bula, ponds 25.7. 1992 S. Persson leg.” (3 exs. MZLU). – **Guinea**: “Seredoux, lux 7-8.4. 1975 Zott” (14 exs. ZMHB, 1 ex. MZH); same data but ”4.5. 1975” (5 exs. ZMHB); same data but ”5.4. 1975” (2 exs. ZMHB); same data but ”4.4. 1975” (4 exs. ZMHB, 3 exs. MZH); same data but ”16.4. 1975” (1 ex. ZMHB); same data but ”18.4. 1975” (1 ex. ZMHB). – **Burkina Faso**: “Karfiguéla 29.10. 1973 Linnavuori leg.” (1 ex. MZH); “Niangoloko 26.10. 1973 Linnavuori leg.” (1 ex. MZH); “Ouagadougou 7.10-11. 1926” (1 ex. NHMB); “Nadiagow MV August 2005 Moretto” (1 ex. NHMB). – **Chad**: “Bebedja 28-31.5. 1973 Linnavuori leg.” (7 exs. MZH); “Nr Bongor 27.5. 1973 Linnavuori leg.” (1 ex. MZH). – **Central African Republic**: “Bambari UV 2. 1964 Pierrard” (1 ex. MRAC). – **Sudan**: “Equatoria, Yambio 18-25.4. 1963 Linnavuori leg.” (1 ex. MZH); “Dahr el Ghazal, Wau 19.2. 1963 Linnavuori leg.” (1 ex. MZH); “Dahr el Ghazal R. Malmul 21.2. 1963 Linnavuori leg.” (1 ex. MZH); “Aluoklua Riv. 30.5E-6.30N, 15.4. 1954 Reid T.” (1 ex. AMGS); “L. Shambe 21.1. 1954 Omer-Cooper” (1 ex. AMGS); “Alel R. Lau 14 mi.NE of Yirol 17.1. 1954 Omer-Cooper” (1 ex. AMGS); “L. Nyibor 23.1. 1954” (1 ex. AMGS); “Rain ponds S of Rumbek nr Wulu 19.1. 1954”(3 exs. ZMHB); “R. Maila 30.57E, 4.39N, 29.1. 1954” (5 exs. AMGS); “Minkammon 31.31E-6.2N, 16–17.1.1954 O-C.” (1 ex. AMGS). – **Sierra Leone**: “Makeni 28.11. 1993 light trap/Cederhalm-Danielsson-Hall” (4 exs. MZLU); same data but ”27.11. 1993” (7 exs. MZLU; habitus in Fig. 412); “Kalangba 8.11. 1980 Jump leg.” (1 ex. USNM, 1 ex. MZH); same data but ”9.11. 1980” (1 ex. USNM); same data but ”25.10. 1980” (4 exs. USNM). – **Liberia**: “Suakoko 26.4. 1952 light trap Blickenstaff” (1 ex. USNM); “Suakoko 8.4. 1953 Blickenstaff” (1 ex. USNM). – **Ivory Coast**: “Nord CI, Ferkessedougou 10-20.5. 1964 Decelle leg.” (1 ex. MRAC). – **Ghana**: “Upper East Pr. Navrongo Env. 11-13.6. 2006 Pokorny S. leg.” (3 exs. NMPC, 1 ex. MZH). – **Togo**: “Reg. Plateaux, Pref. Kloto, nr. Kpimé, 1 km NW Seva village 10.2.2006 Komarek & Hounguè leg. / 300 asl, agricultural irrigation ditch” (1 ex. NMW); “Sokodé- Kmpamgalam, FL HQ (niedere vegetat.) N-22.132 S, 4. 1980/Krell leg.” (1 ex. NHMB). – **Benin**: “Dép. Zou, commune de Zogbodomè 29.1. 2006 Goergen leg. / Lokoli forest 17 m asl, light trap” (1 ex. NMW); “Penessoulou, pond, forest area Oct. 2003 Goergen leg.” (1 ex. NMW, 1 ex. MZH); “SE Benin, 15 km SE Save 8–11.4.2000 Halada leg.” (1 ex. NMW). – **Nigeria**: “NW St. Badeggi rice fields 8-9.8.1973 Linnavuori leg.” (70 exs. MZH); “Ilorin Prov., Ilorin15–18.2 1949 Malkin / small clear pond” (3 exs. BMNH, 1 ex. MZH); “NW St. Yelwa 23.7. 1973 Linnavuori leg.” (5 exs. MZH); “W St. Igboho-Kishi 19.7.1973 Linnavuori leg.” (1 ex. MZH); “R St. nr Mbiama 4-5.7. 1973 Linnavuori leg.” (1 ex. MZH); “MW St. Sapoba forest 1.9.1973 Linnavuori leg.” (1 ex. MZH); “NE St. Gombe-Bauchi 27.8.1973 Linnavuori leg.” (1 ex. MZH); “Ibadan ca. Jan.-Juni 1954 Stenholt-Clausen leg. / *Laccophilus congener* O-C. J. Balfour-Browne det. 1961” (2 exs. ZMUC); “R. Kaduna at Kadoura ? 1964” (1 ex. AMGS); “Little stream Oyo-Ibadan 28.3. 1963 JOC.” (1 ex. AMGS); “Pools in dry stream bed Kontagora 5.4. 1963” (1 ex. AMGS); “Pools in river bed Kontagora 3.4. 1963 JOC.” (1 ex. AMGS); “Stream at Assob 36 miles from Jos 13.4. 1963” (2 exs. AMGS); “Stream reservoir Jos 10.4. 1963” (1 ex. AMGS); “Pond in stream bed Kontagora-Kaduna rd 5.4. 1963 JOC.” (1 ex. AMGS); “Stream 86 miles from Makurdi at Jos road 24.4. 1963 JOC.” (1 ex. AMGS); “Detritus pond, Jos-Bauchi rd 9.4. 1968” (3 exs. AMGS); “Stream, escarpment, rd Jos-Wambe 13.10. 1963 JOC.” (1 ex. AMGS); “Stream 64 miles from Bida on Jebba rd 15.IV. 1963 JOC.” (9 exs. AMGS); “Stream Zaria rd about 3 miles from Kaduna 4.4. 1963 JOC.” (1 ex. AMGS); “Stream nr Zaria 4.4. 1963 JOC.” (3 exs. AMGS); “Stream 3,5 miles from Oyo 28.3. 1963 JOC.” (10 exs. AMGS); “Trib. of R. Gagere (?) Zaria-Katsina 5.4. 1963 JOC.” (1 ex. AMGS); “Zaria 1969” (1 ex. NHMB); “Rivercrossing rd about 49 miles from Makurdi in Enyogo direction 24.4. 1963 JOC.” (4 exs. AMGS); “New Calabar River nr Port Harcourt 13.1. 1989 Umeozor leg.” (1 ex. USNM). – **Cameroon**: “SW Kumba-Mamfe 23.6. 1973 Linnavuori leg.” (2 exs. MZH); “Maroua 10/11.1965 (lumière) Schmitz leg.” (1 ex. MZH); “Boura, partially parched small stream with riverine forest in savannah, at light 13.1. 1978/Gärdenfors-Hall-Samuelsson” (1 ex. MZLU); “Obala Juin 1969” (2 exs. NHMB); “Tsang Monatiele 5.1. 1970” (1 ex. NHMB). – **Gabon**: “Lagune Iguelá, Fortét à Est. Tassi Gen. 97 Bilardo / *Laccophilus congener* O-C. det. Rocchi S. 1998” (1 ex. OLML). – **Congo**: “Brazzaville P.K. Rouge G. Onore 4. 1979 G. Onore / *Laccophilus congener* O-C. det. Rocchi S. 1998” (1 ex. OLML); “Djili P.K. Rouge 3. 1979 Onore” (2 exs. NHMB); “Loudima, sur la route en savane 20.3. 1980 Onore” (3 exs. NHMB). – **Zaire**: “Parc National Garamba 1.9.1952 De Saeger leg.” (1 ex. MRAC); PNG II/fc/13, 7.3. 1952 De Saeger 3257” (1 ex. IRSNB); “PNG 30.1. 1950 G. Demoulin 240” (1 ex. NHMB); same data but ”8.5. 1950, 494” (1 ex. NHMB); “PNG II/fd/12, 10.3. 1952 De Saeger 3180” (1 ex. NHMB); “Tshuapa-Mbandaka 1964, A.B. Stam leg.” (3 exs. RMNH); “Dilolo 8-9. 1931 de Witte” (2 exs. NHMB); “Haut Uele Moto 1920 L. Burgeon / *Laccophilus congener* O-C. det. Wewalka 1981”(1 ex. OLML); “Lusindoi 15.7. 1911 L. Burgeon/*Laccophilus congener* O-C. det. Wewalka 1981” (1 ex. OLML); “Elisabethville 1.1956-1.1957 à la lumière Seydel leg.” (1 ex. MRAC, 1 ex. MZH, 1 ex. NHMB); same but ”1957-1958”(1 ex. NHMB); “Katanga Kansenia 6. 1925 G.F. de Witte / *Laccophilus congener* O-C. det. Wewalka 1981” (1 ex. OLML). – **Kenya**: “Mariakani Dam, Kilifi distr. 16.9.1976 Holmen leg.” (1 ex. ZMUC); “Machakos district, Athi River 14.9.1976 Holmen leg.” (1 ex. ZMUC); “Djili P.K. Rouge 3. 1979 G. Onore” (2 exs., NHMB). – **Tanzania**: “Petukiza, ponds, Tanga Distr. 23.9. 1976 Holmen leg.” (1 ex. ZMUC); “Deforested place nr Mangula, 297 m, N-07°52'20”, E36°55'06”, 18.7. 2004, 297 m at light, Sprecher” (4 exs. AMGS, 1 ex. MZH). – **Angola**: “Pediva ca. 30 mi. E of Porto Alexandre 400 ft. 26-27.6. 1954 / Pools in warm, saline river, thick weed” (1 ex. BMNH); Mossamedes Distr. Rio Coroca 23.6. 1954 / Small clear pool in sand with *Chara”*(1 ex. BMNH); “Rio Coroca 8 m N of Porto Alexandre 22-23.6. 1954 / Pond with Algae & *Lemna*, fringing *Juncus”* (6 exs. BMNH, 1 ex. MZH); “Ca. 10 mls. W of Cainde, ca 3500 ft 15.6. 1954 / Stagnant water hole, nitellid algae and muddy silt” (2 exs. BMNH). – **Zambia**: “Central Prov., Lusaka 8.1. 1982 Selander leg. / rain pond” (2 exs. MZH); “Lusaka 8.5. 1974 Lange” (3 exs. CGC); same data but ”23.4. 1974” (1 ex. CGC); “Kafue NP Chunga Camp, 15.02.35S-26.00.09E, 29.3. 1993 lux Uhlig leg.” (2 exs. ZMHB, 1 ex. MZH); “Kackhalola 820 m, 14.45.43S-30.35.46E, 19.3. 1993 lux Uhlig leg.” (1 ex. ZMHB); “Copperbelt Pr. Mwekera 23.1. 1982 Selander leg. / rain pond” (2 exs. MZH); “Abercorn 13.4. 1951 H.O. Backlund leg. / *Laccophilus propinquus* O.-C. det. A.N. Nilsson 1996” (1 ex. MZLU); “Kasanka Nat. Res. N-12.30, E30.15, rain-filles dams Reavell” (4 exs. NHMB). – **Malawi**: “Fort Hill 8.10. 1945 JOC.” (1 ex. AMGS); “Mulanje Mts.,env. 22.-26.12. 2001 Kantner” (1 ex. NHMB); “Dambo below Livingstonia, lakeshuve 21.9. 1945” (1 ex. IRSNB). – **Zimbabwe**: “Wankie Res. waterhole 3.9. 1948” (5 exs. AMGS); “Wankie Game Res. 9. 1948 waterhole JOC: / *Laccophilus congener* det. J. Omer-Cooper” (10 exs. AMGS); “Wankie Game Res. Shapi Pan 5.9. 1948 / *Laccophilus congener* det. J. Omer-Cooper” (2 exs. AMGS); “Wankie Game Res. Shapi Pond 5.9. 1948” (1 ex. AMS); “Wankie Game Res. 4.9. 1948” (3 exs. AMGS); “Wankie Res., Masumu Dam 4.9. 1948” (3 exs. AMGS); “Wankie Res., Masumu Dam 9. 1948” (2 exs. AMGS); “Wankie Res., Masumu Dam 9. 1948, waterhole” (2 exs. AMGS); ”5 mi. SE Wankie 7.4. 1968 Spangler” (2 exs. USNM, 1 ex. MZH); “Marandellas 2.9. 1948 JOC. / *Laccophilus congener* det. J. Omer-Cooper” (1 ex. AMGS); “Sinkukwe 30.12. 1948 JOC.” (1 ex. AMGS); “Small stream, Halfway Hotel Salisbury-Gatooma 14.9. 1948” (3 exs. AMGS); “Gwaai River 1.9. 1948 JOC.” (1 ex. AMGS); “Gwai R. 3.4. 1968 Spangler” (4 exs. USNM). – **Mozambique**: “Niassa Prov. S12°17'28.8”, E34°46'31.4”, Mandambuzi Marsh Watson 6.4. 2009” (1 ex. CGF); “Jangamo Block N-24.3212, E35.280, 7.6. 2008 De Moor” (1 ex. AMGS). – **Namibia**: “Windhoek, New Dam 7.7. 34 JOC:” (3 exs. AMGS); “Windhoek Town Dam 7.7. 1939” (1 ex. AMGS); “SW Prot. Windhuk Parch 1919” (1 ex. SAMC); “E Capriwi, Katima Mulilo lux, 17.29S-24.17E, 3-8.3. 1992 Uhlig leg.” (3 exs. ZMHB, 1 ex. NMNW); “Kavango: Kaudom-Camp, Wasserloch, Schilf + Gras-Gesiebe, 18.31S-20.43E, 22-25.2. 1992 Uhlig leg.” (1 ex. ZMHB); “E Capriwi Mudumu NP: Nakatwa 18.10S-23.26E, 8-13.3. 1992 lux Uhlig leg.” (1 ex. ZMHB); “E Capriwi Mudumu NP: Buffalo Trails Camp, lux, ca. 18.10S-23.26E, 12.3. 1992 Uhlig leg.” (1 ex. ZMHB, 1 ex. MZH); “Kavango Mahango Game Reserve, piknik site, lux 24.11. 1993, 18.13S-21.45E Uhlig leg.” (1 ex. ZMHB); “Kavango: Popa Falls 18.07S-21.35E, 26.2.-3.3. 1992 Uhlig leg.” (2 exs. ZMHB, 1 ex. MZH; habitus in Fig. 411); “Okarupa ca. 17 mi E of Okahandja 4900 ft, 22.5. 1954/pools in overflow stream from dam, much weed and algae” (2 exs. BMNH); “Namib Mt. Naukluft river 24.16 S-16.15 E 10.8. 1989 shorewashing, river Endrödy & Klimaszew” (1 ex. TMSA); “Damaral. Okahandja 21.59S-16.52E / 12.9. 1974 shore washing Endrödy-Younga leg.” (1 ex. TMSA); “Kro, ca.15 mi. SE of Namutoni 30.5. 1954 / Shallow and muddy, with algae” (2 exs. BMNH, 1 ex. MZH); “Ca. 7 mi NE of Grootfontein 29.5. 1954 / Waterhole in dolomits” (2 exs. BMNH, 1 ex. MZH); “Ovamboland Namutoni 31.5. 1954 / Weedy water-hole and stream” (1 ex. TMSA). – **Botswana**: “Tsotsorogo Pan 17.6.-9.7. 1930 / *Laccophilus taeniolatus* Régimbart det. Gschwendtner” (14 exs. TMSA); same data but *“Laccophilus congener* O-C. det. Wewalka 1981” (3 exs. OLML); “N´Kate Makarikari 6-23.8. 1930 / *Laccophilus taeniolatus* Régimbart det. Gschwendtner” (2 exs. TMSA); “Chobe NP, Savuti-Camp 18.33.55S-24.03.53E, 11.3. 1993 lux Uhlig leg.” (1 ex. ZMHB). – **South Africa**: “Trsvl., 5 mi W Warmbad 24-25.2. 1968 Spangler” (33 exs. USNM, 5 exs. MZH); “Tvl Nylstroom Donkerpoort dam 24.8. 1948” (1 ex. AMGS, possibly belongs to the type serie of *Laccophilus congener* but lacks paratype label); “Tvl Dam wall 28.11. JOC.” (1 ex. AMGS); “Tvl Potgietersrust 23.4. 1933 Taylor” (1 ex. AMGS); “OLF 101D 21.3. 55” (TVL Olifantsvlei) (1 ex. AMGS); “Transvaal Bronkhorstspruit Rinosterpoort b. Franz leg.” (1 ex. NMW); “C. Transvaal Roodeplaat Dam 28.37S-28.23E / 14.8.1974 shore washing leg. A. Strydom” (1 ex. TMSA, 1 ex. MZH); “Transvl Pretoria distr., Roodeplaat / UV light trap 30.10.-10.11. 1960 Dr. Neubecker” (1 ex. TMSA); “Roodeplaat Pretoria Distr. 10. 1960 Neubecker” (2 exs. TMSA); “Tv Nelshoogte Forest Station 25.50-30.50E / 2.12.1986 UV light collection Endrödy-Younga leg.” (1 ex.TMSA); “Tv Nelshoogte galery for. below St. 25.51S-30.53E / 4.12. 1987 UV light collection leg. Endrödy-Younga” (1 ex. TMSA); “Tvl Naboomspr. Torino Ranche24.37S-28.38E / 15.1. 1989 UV light, vlei edge Endrödy-Younga leg.” (1 ex. TMSA); “Tvl Rhenosterpoort N.R. 25,45S-28.55E / 15.11. 1975 at light Endrödy-Younga leg.” (1 ex. TMSA, 1 ex. MZH); “Nelspruit Pond 27.4. 2010, S25°32'13,83”, E30°59'50,35” Hidalgo-Galiano & Kleynhans leg.” (1 ex. CIR); “Transvaal April 1946” (1 ex. AMS); “Fountains 26.8. 05” (1 ex. TMSA); “Fountains 26.8. 1895(?)” (1 ex. TMSA); “Plat River 6-18.4. 05 / Waterberg Distr. Swierstra / *Laccophilus taeniolatus* Régimbart Gschwendtner det.” (7 exs. TMSA); “W Cape Arniston 7. 1946” (1 ex. BMNH); “ECPr. Matatiele 4-5.5. 1956 JOC.” (3 exs. AMGS); “ECPr. Matatiele 5.5. 1956 JOC.” (1 ex. AMGS);” “ECPr. Matatiele 5.5. 1956 JOC. / *Laccophilus congener* O-C. det. J. Omer-Cooper” (5 exs. AMGS); “ECPr. Mt Currie 6.5. 1956 JOC.” (2 exs. AMGS); “EC., Hwy 352, 3 km S Tsomo, in river 22.5. 2005 Challet” (1 ex. CGC); “ECPr., Qumbu 2.5. 1956 J.O-C.” (1 ex. AMGS); “Transkei Port St Jones Silaka 31.33S-29.30E/2.12. 1987 UV light collection leg. Endrödy-Younga” (1 ex. TMSA); Nat. -Drakensbg. Cathedral Peak 28.57S-29.12E / 15.3.1976 at light leg. Endrödy-Younga” (3 exs. TMSA); “Natal, TUG 77 Q38 (Tugela River system, Colenso 25.7. 1954” (1 ex. AMGS); “Natal Zululand Mtuba-tuba 23.9. 1947 JOC.” (1 ex, AMGS); “Natal 1942” (1 ex. AMGS); “Caffraria / Wahlb.” (1 ex. NHRS). – **Swaziland**: ”9 mi. from Mbabane 6.12. 1948 JOC” (1 ex. AMGS). – **Lesotho**: “Hensley’s Dam 8 mi. SW Leribe 30.3. 1951 / Brinck-Rudebeck” (6 exs. MZLU).

##### Comments on synonymy.

The holotypes of *Laccophilus taeniolatus* and *Laccophilus congener* have been examined and compared with each other. No diagnostic differences can be discerned in shape of male genitalia. Externally both species are similar and accordingly they both belong to the same species, and *Laccophilus congener* is hence a junior subjective synonym of *Laccophilus taeniolatus*.

##### Diagnosis.

*Laccophilus taeniolatus* is a widely distributed, but still reasonably uniform species. It resembles externally most of *Laccophilus propinquus*, *Laccophilus simplicistriatus* and *Laccophilus complicatus*. From the two latter species *Laccophilus taeniolatus* can in most cases be separated by study of the elytral colour pattern: dark irrorations are clearly reduced frontally on each side of the suture and an irregular, pale area is formed on base of each elytron. *Laccophilus propinquus* and *Laccophilus taeniolatus* requires dissection of male genitalia for correct identification; apex of penis exhibits a small difference in location of the small, frontal process. Additionally penis is distinctly shorter in *Laccophilus propinquus*.

##### Description.

Body length 3.8–4.2 mm, width 1.9–2.3 mm. Colour pattern of dorsal aspect of body distinct; exhibits limited variation (Figs 410–412).

Head: Pale ferrugineous, posteriorly darker, ferrugineous to dark ferrugineous. Sometimes posteriorly with darker area; however vague and hardly discernible. Slightly mat; rather finely microsculptured; double reticulation indistinct; weakly developed medially on head. At eyes finely and irregularly punctate; between eyes with a few scattered, fine punctures.

Pronotum: Pale ferrugineous, frontally and posteriorly in middle with a dark brownish to dark ferrugineous marking. Posterior margin narrowly dark. Frontal and basal dark areas sometimes connected by a vague, ferrugineous area. Finely microsculptured, submat. Reticulation indistinctly double; large meshes (when distinguishable) contain 2–4 small meshes. At margins with very fine, sparse and irregularly distributed punctures.

Elytra: Pale ferrugineous, with dense, dark ferrugineous to blackish irrorations, which are somewhat unevenly distributed on elytra. Frontally, along dark suture, dark irrorations sparser, in part absent. Longitudinal, dark irrorations can in part (especially laterally) simply be “hollow” and consist only of dark outlines (Figs 410–412). Submat, finely microsculptured; reticulation indistinctly double. Kind of meshes, in general, difficult to classify according to size. Weakly developed rudiments of large meshes can be discerned. Each elytron with three (discal, dorsolateral and lateral), longitudinal areas with fine and sparse punctures.

Ventral aspect: Pale ferrugineous to ferrugineous. No distinct colour pattern. Rather shiny, very finely microsculptured; in part microsculpture indistinct. Abdomen basally very finely and sparsely striated. Metacoxal plates with fine, shallow, transversely located furrows. Apex of prosternal process slender, pointed. Apical sternite lacks asymmetric knob; provided with curved, sublateral impressions (of variable kind; one more pronounced) (Fig. 56).

Legs: Pro- and mesotarsus somewhat enlarged, rather long. Provided with suckers.

Male genitalia: Penis in lateral aspect quite long and evenly curved; minor frontal process protuding (Fig. 253).

Female: Apical sternite (Fig. 57).

##### Distribution.

Gambia, Guinea-Bissau, Guinea, Burkina Faso, Chad, Central African Republic, Sudan, Sierra Leone, Liberia, the Ivory Coast, Togo, Benin, Nigeria, Cameroon, Gabon, Congo, Zaire, Kenya, Tanzania, Angola, Zambia, Malawi, Zimbabwe, Mozambique, Namibia, Botswana, South Africa, Swaziland, Lesotho (Fig. 539). Because of extensive taxonomic confusion we have only accepted personally verified records.

##### Collecting circumstances.

A tolerant species, which occurs in a great variety of water bodies, both in stagnant and running waters. It is also collected from temporary pools. *Laccophilus taeniolatus* is a good flier and has often been captured at light collection. It also occurs in water bodies created or highly influenced by man, as in agricultural irrigation ditches. It dwells in forests as well as in open habitats as steppes and savannas (e.g. Guignot 1959a). Regarding collecting localities, see also Bilardo and Rocchi (2013).

#### Laccophilus propinquus

Taxon classificationAnimaliaColeopteraDytiscidae

Omer-Cooper, 1958

Figs 57–58 , 254 , 413 , 540

Laccophilus propinquus Omer-Cooper 1958b: 37, 43, 45, 46 (original description, faunistics); Omer-Cooper 1965: 77, 82 (description, faunistics); Omer-Cooper 1970: 288, 289, 290 (discussion, description, faunistics); Pederzani 1988: 107 (faunistics, biology); Nilsson 2001: 249 (catalogue, faunistics); Nilsson 2015: 216 (catalogue, faunistics).

##### Type locality.

Malawi: Mwanza.

##### Type material studied

(14 exs.). Holotype: male: “Type / male, female (symbols) Types / River near portuguese border, near Mwanza 9.II. (11 ?) 1948 / Brit. Mus. 1957-660 / *Laccophilus propinquus* O-C.”(BMNH; habitus in Fig. 413). – Paratype: female: Pinned together with holotype but on separate label (1 ex. BMNH); “Paratype / Nyasaland Zomba Plateau Reservoir 7.11. 1948” (1 ex. AMGS); “Paratype / Nyasaland Reservoir Mwanza 9.11. 1948” (2exs. AMGS); “Paratype / Nyasaland stream longer Lilongwe rd. 20 miles from Dedza 30.9. 1948” (1 ex. AMGS); “Paratype / Nyasaland dam on lower Lilongwe rd. 29.9. 1948” (2exs. AMGS); “Paratype / Nyasaland Cisaiti R. nr. Dedza 28.9. 1948” (1 ex. AMGS); “S. Rhodesia Inganyi River 17.IX. 1948 / *Laccophilus propinquus* sp. n. det. J. Omer-Cooper” (4 exs. AMGS); Nyasaland Dambo below Livingstonea Lake shore 21.9. 1945 / Paratype / *Laccophilus propinquus* O.-C., O. Cooper det.” (1 ex. IRSNB).

##### Additional material studied

(20 exx.). **Tanzania**: “Ruvu North Forest Reserve, waterholes, 3 km SE of Base 6°37'20” S, 38°55'00” E alt. 250 ft 1.11. 1992 / Hynd Collection” (4 exs. BMNH, 1 ex. MZH); “Ruvu North Forest Reserve, Base Camp6°37'40” S, 38°51'14” E alt. 200 ft 30.10. 1992 / Hynd Collection” (1 ex. MZH); “Zanzibar Pemba Sept. 1955 Fowler” (3 exs. AMGS); “Zanzibar Mangapwani Rd. Sept. 1955 JOC.” (5 exs. AMGS). – **Malawi**: “Dam, Dedza on lower Lilongwe rd. 30.9. 1948” (2exs. AMGS); “Mtiti R. 1.10. 1948” (3exs. AMGS).

##### Diagnosis.

*Laccophilus propinquus* is very closely related to *Laccophilus taeniolatus* and externally very similar to this species. Small but distinct differences in shape of penis allow confident separation of the two species; penis short and apical process vague in *Laccophilus propinquus* (see also diagnosis of *Laccophilus taeniolatus*).

##### Description.

Body length 3.8–3.9 mm, width 1.9–2.0 mm. Pale ferrugineous; dorsal, dark ferrugineous colour pattern of body quite distinct (Fig. 413).

Head: Pale ferrugineous, posteriorly with vague dark ferrugineous area. Submat, finely microsculptured. Reticulation almost of one kind, simple; double reticulation indistinct, weakly developed and difficult to distinguish. At eyes with irregularly distributed, sparse punctures.

Pronotum: Pale ferrugineous, anteriorly and at base with rather narrow, vague blackish ferrugineous to dark ferrugineous markings which are medially connected by a vague ferrugineous area. Submat, finely microsculptured; reticulation indistinctly double but size classes difficult to separate. Laterally and anteriorly with indistinct and sparse punctures.

Elytra: Pale ferrugineous, with rather distinct dark ferrugineous markings formed as irrorations (Fig. 413). Rather shiny, although finely microsculptured. Reticulation indistinctly double; clear size-classes difficult to discern. Discal, dorsolateral and lateral rows of fine and irregular punctures discernible but weakly developed.

Ventral aspect: Pale ferrugineous. Rather shiny and very finely (partly indistinctly) microsculptured. Abdomen with fine to very fine striae. Metacoxal plates with fine and shallow transverse furrows. Almost impunctate. Prosternal process slender, pointed. Apical ventrite almost symmetric, lacks lateral knob; apex more angle-shaped than in female (Fig. 57).

Legs: Pro- and mesotarsus long, slender, and with suckers.

Male genitalia: Penis In lateral aspect comparatively short, evenly curved and apical process not prominent (Fig. 254).

Female: Externally as male but apex of apical ventrite broader and more rounded (Fig. 58).

##### Distribution.

Malawi, Zimbabwe, Tanzania and Zanzibar (Fig. 540). Omer-Cooper (1965) adds Mozambique.

##### Collecting circumstances.

The species has been collected in streams e.g. with rocks, sand and some vegetation. It is also recorded from standing water in a swamp and a reservoir.

#### Laccophilus complicatus

Taxon classificationAnimaliaColeopteraDytiscidae

Sharp, 1882

**Figures 59–66.**
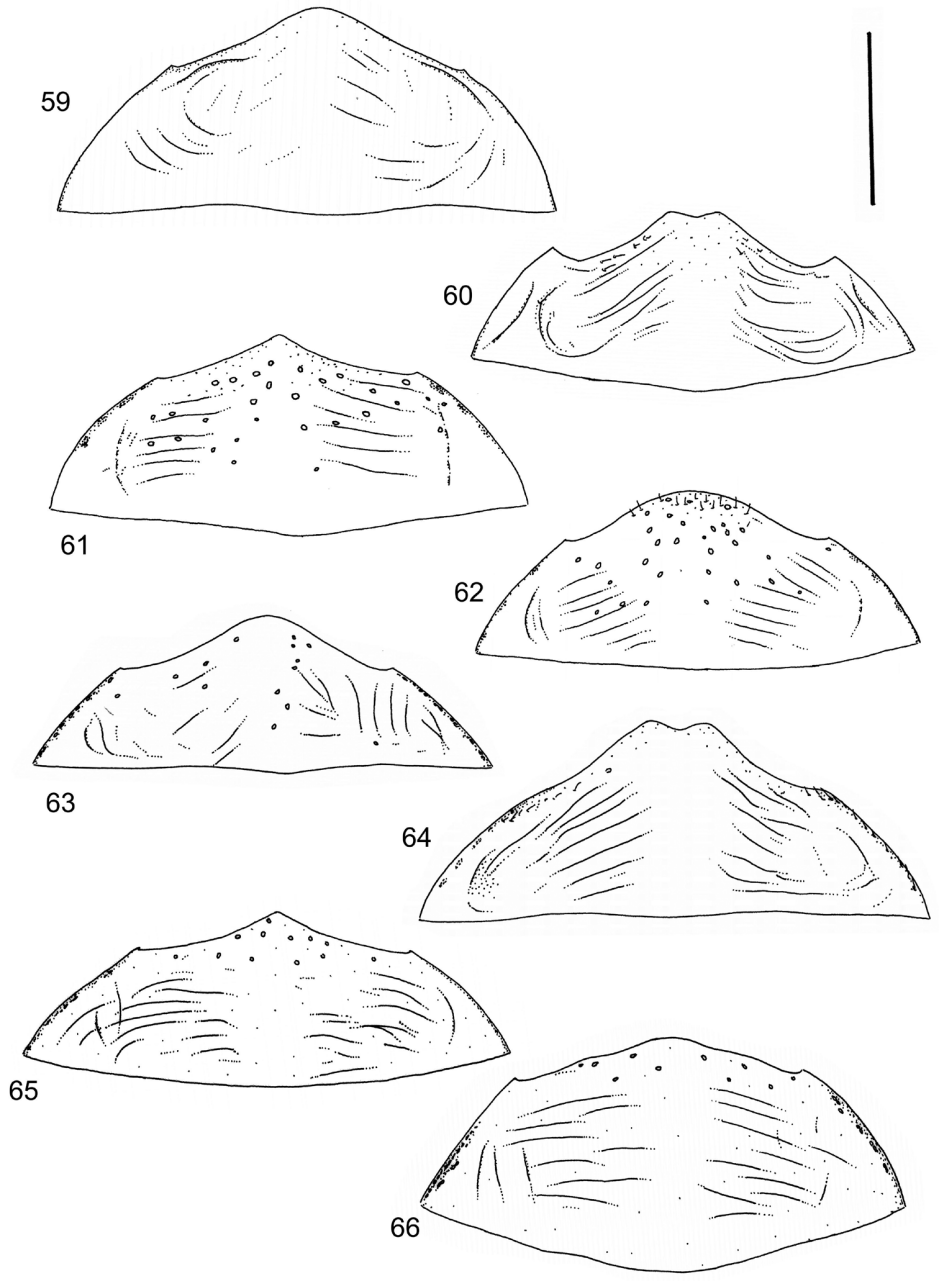
Apical ventrites **59** *Laccophilus complicatus* male, and **60** female **61** *Laccophilus irroratus* male, and **62** female **63** *Laccophilus rivulosus* male, and **64** female **65** *Laccophilus immundus* male, and **66** female. Scale bar 0.5 mm.

Figs 59–60 , 255 , 414 , 541

Laccophilus complicatus Sharp 1882: 308, 309 (original description, faunistics, discussion); Kolbe1883: 402 (description, faunistics); v. d. Branden 1885: 20 (catalogue, faunistics); Régimbart 1895: 138 (description, faunistics); Zimmermann 1919: 122 (faunistics); Zimmermann 1920a:17 (catalogue, faunistics); Bertrand 1928a: 184, 185 (juvenile faunistics); Bertrand 1928c: 365 (juvenile description); Bertrand 1948: 12: (juvenile faunistics); Bertrand 1954: 284 (juvenile discussion, faunistics); Guignot 1946c: 279, 281, 283 (discussion, description, faunistics); Guignot 1959a: 570, 573, 574 (description, faunistics); Bertrand 1963: 411 (discussion, faunistics); Omer-Cooper 1970: 290, 291 (description); Bertrand and Legros 1971: 245 (faunistics, biology); Bameul 1984: 94 (faunistics); Rocchi 1991: 80, 86 (faunistics); Nilsson 2001: 242 (catalogue, faunistics); Pederzani and Rocchi 2009: 95 (faunistics, list); Nilsson 2015: 210 (catalogue, faunistics).

##### Type locality.

Madagascar.

##### Type material studied

(2 exs.). *Laccophilus complicatus*: Lectotype (by present designation) male: “Type / Madagascar / Sharp Coll. 1905-313 / Madag. / *Laccophilus rivulosus* Klug /Type 574 *Laccophilus complicatus* sp. n. Madagascar” (BMNH). – Paralectotype: female: ”574 / Cotype / Madagascar / Sharp Coll. 1905-313 / Madagascar / *Laccophilus complicatus* Shp Co-type” (1 ex. BMNH).

##### Additional material studied

(390 exs.). **Madagascar**: “E-Mad., Morarano, N. Morananga 900 m asl, 13.1. 1995 Janák” (9 exs. NMW; habitus in Fig. 414); “Moramanga env. 10-18.12. 1997 Pacholátko” (1 ex. NHMB); “E-Mad. Ampamoho nr Andilamena 1200-1300 m asl 18-20.1. 1995 Dunay & Janák” (20 exs. NMW); “Ambatombe nr Andilamena 900 m asl, 17.1. 1995 Dunay & Janák” (10 exs. NMW, 10 exs. MZH, 1 ex. NHRS); “E-Mad. Andranokobaka, N Moramanga 800 m asl 13.1. 1995 Dunay & Janák” (21 exs. NMW); “W-Mad. Manindaray W Sakahara 700-800 m asl 30.1. 1995 Dunay & Janák” (1 ex. NMW); ”5 km S Ampamoho 950-1000 m asl 18-20.1. 1995 Dunay & Janák” (3 exs. NMW); “E Sakahara 30.1. 1995 Manindray 700-800 m Dunay & Janák” (1 ex. NMW); “Mad. Centr. Antananarive 18-19.1. 1993 Janák” (1 ex. NMW); “Envir. de Tananarive 7. 1970 Pederzani” (1 ex. AMGS, 2 exs. NHMB); “Prov. Tananarive, env. de Arivonimamo 22.7. 1970 Pederzani” (1 ex. AMGS); “Tan. Manjakatompo 3.1. 1958 Keiser” (1 ex. NHMB); “Mandrare Bas., Loc. prés Andaza, affl. non nommé, Riv., 46°34'05"E, 24°03'16"S, Alt. 315 m 26.4. 1995 Elouard & Pilaka” (1 ex. NMW); “Prov. Tamatava, 3.3 km N Ambabasoratra 31.8. 1962 Cashatt” (1 ex. USNM); “Mad-est 1100-1200 m, NP Ranomafana / Vohiparara 21-24.1. 1993 Janák” (2 exs. NMW); “Prov. Fianarantsoa 7 km W Ranomafana, 1100 m 8-21-10. 1988 Steiner W.E. / At black light in montane rainforest” (3 exs. USNM, 1 ex. MZH); “Prov. Fianarantsoa 7 km W Ranomafana, 1000 m 23-28.2. 1990 Steiner W.E.” (1 ex. USNM); “Ankaratra (Antananarivo) Res. Manjakatompo 6.10. 2001 / Gerecke et Goldsmith leg. / Helocrene at left border of affluent to Lac Froid, 1700 m asl” (2 exs. BMNH); “Manjakatompo 5.10. 1989 Bartolozzi & Taiti leg. / m 1700 Stazione pescicoltura / *Laccophilus complicatus* Sharp det. Rocchi 1989”(3 exs. CSR); “Antranovy (Antananarive) 13 km W from Arivonimana, 13.7. 2001 helocrene in rice field exp. N / 1480 m asl / Gerecke et Goldsmith leg.” (9 exs. BMNH, 2 exs. MZH); “Tsimelahy (Tuelar) 5.9. 2001, Riv. Antarantsa downstr. piceine naturelle / 200 m asl / Gerecke et Goldsmith leg.” (1 ex. BMNH); “Foret de l’Est Perinet-Anosibe 11-12. 1959” (1 ex. BMNH); “Tan. Madag, Ampefy, Lac Kavitaha wi 25.III. 58 Keiser” (1 ex. NHMB); “Tam. Perinet 3.12. 1957 Keiser” (1 ex. NHMB); “La Mandraka, ex. coll. Breuning” (1 ex. MRAC); “Suberbieville, ex. coll. Breuning” (5 exs. MRAC); “Tananarive 22-29.i. 1972 Hecq” (1 ex. MRAC); “Tananarive, at light 12.12. 1955 E. Mac (?) Callan” (1 ex. AMGS); “Tananarivo Friedrichs / *Laccophilus complicatus* Sharp det. Brancucci” (1 ex. ZMHB); “Fianarantsoa Prov., Foret d’Antsirakanbiaty, 7.6 km 285˚ WNW Itremo, elev. 1550 m 22-26.1.2003 / 20°35'36"S 046°33'48"E, collected at light in montane rainforest, Fischer, Griswold & al. leg.” (2 exs. CAS); “Fianarantsoa Ranomafana National Park, Talatakely res. lab. area, black light mercury vapour / light, elev. 940 m 21°14'53,5"S, 47°25'35,9"E, 31.10-20.11.1998 Lee & Ribardo leg.” (1 ex. CAS); “Fian Isalo Menamaty R., degraded river with lots of vegetation used by women to wash clothes in, 11.5. 2006, N-22°33.001, E45°24.074, 757 m Bergsten et al.” (12 exs. NHRS, 5 exs. MZH); “Fian Andringitra Zomandao R. bridge on road to the camp belamba, vegetation rich edges along the river N-22°6.225, E46°55.244, 8-9.5.2006, 1421 m Bergsten et al.” (13 exs. NHRS); “Fian. Col. des Tapias Rte Tana-Fianarantsoa, vegetation- rich pond with fish 6.5. 2006 N-20°46.376, E47°10.749, 1718 m Bergsten et al.” (9 exs. NHRS); “Mangoky Bas, Loc. Andringitra, Camp B, Zomandao River 46°53'46"E/22°07'12"S 1600 m asl 30.11. 1993 leg. ORSTOM” (1 ex. NHRS); “Andringitra Rambavy R. (Cascade) N-22.153, E 46.9, 1979 m, 8.5. 2006 Bergsten et al. / BMNH(E) <794244> DNA Voucher” (1 ex. NHRS); “Ft Dauphin, rice paddies N-24.824, E 46.866, 34.44 m 19.5. 2006 Bergsten at al/BMNH(E)(E) <794247> DNA voucher” (1 ex. NHRS); “TOLI Zombitse, N-22 64 Andramomena R. pool, N-22.64, E44.864: 577 m, 15.5.2006 leg. Bergsten” (1 ex. NHRS); “Mangoro Bas Loc. Ankirihitra Tsarantanana River 47°17'37"E/19°23'00"S, 11.1. 1997 Elouard leg.” (1 ex. NHRS); “Mad-est. 1100-1200 m P.N. Ranomafana / Vohipara 21-24.1. 1993 Dunay & Janák leg.” (1 ex. NHRS); “Mah/Tol. Melaky/Menabe Ambojihanahary NP, S18.26849. E045.46346, 906 m.a.o., 19.12. 2009 water net, field Bergsten et al. / NHRS-JLKB 000000723” (1 ex. NHRS); “Madagascar” (1 ex. USNM, 2 exs. ZMHB).

##### Specimen with uncertain locality

(2 exs.). “Dirjo 24” (1 ex. NMW); “Mexique” (1 ex. MHNG).

##### Diagnosis.

*Laccophilus complicatus*, distributed solely on Madagascar, resembles most of three species on mainland of Africa, viz. *Laccophilus simplicistriatus*, *Laccophilus taeniolatus* and *Laccophilus propinquus*. In general *Laccophilus complicatus* is somewhat larger than the three other species. Elytral colour pattern in *Laccophilus complicatus* is uniform and more evenly distributed than in *Laccophilus taeniolatus* and in *Laccophilus propinquus*, in which a vague and irregular, dark, longitudinal marking can be discerned discally on each elytron. Elytral colour pattern in *Laccophilus simplicistriatus* is fairly uniform and exhibits only slight variation. In *Laccophilus complicatus* penis in lateral view is not expanded posterior to apex while in *Laccophilus simplicistriatus* corresponding feature is clearly expanded on each side. From *Laccophilus taeniolatus* the species is distinguished by the tip of penis, which is clearly curved ”upwards” while tip of *Laccophilus complicatus* penis is almost straight. Regarding *Laccophilus propinquus*, *Laccophilus complicatus* is separated from it as *Laccophilus taeniolatus* is but tip of penis vague.

##### Description.

Body length 3.9–4.6 mm, width 2.2–2.6 mm. Pale ferrugineous, with blackish ferrugineous to dark ferrugineous, dense irrorations on elytra (Fig. 414). Dorsal colour pattern of body quite stable; rarely elytral irrorations laterally and at base slightly reduced.

Head: Pale ferrugineous, posteriorly, narrowly dark ferrugineous. Submat, reticulation in part double; large meshes often weakly developed and hardly visible. At eyes, with fine and irregular punctures, extending medially towards midhead.

Pronotum: Pale ferrugineous, anteriorly and posteriorly in middle with vague, darkened areas. Reticulation quite dense, double. Large meshes weakly developed; one mesh contains between three and six fine meshes. At margins with fine, irregular punctures, which are rather indistinct at posterior margin.

Elytra: Pale ferrugineous, with dense blackish ferrugineous to dark ferrugineous irrorations (Fig. 414). Submat, with quite dense reticulation. Reticulation indistinctly double; indistinct fragments of larger meshes extensively discernible. Each elytron provided with three, longitudinal, rather vague areas of fine and sparse punctures.

Ventral aspect: Pale ferrugineous to ferrugineous, abdomen in posterior part blackish to dark ferrugineus. Rather shiny to submat, very finely reticulated. Almost impunctate; fine punctures discernible on apical ventrite. Apical ventrite as in Fig. 59. Basal ventrites provided with fine, slightly curved, striae. Metacoxal plate with some very shallow, almost transversely located furrows. Prosternal process, slender, extended and pointed.

Legs: Pro- and mesotarsus somewhat enlarged; provided with suckers.

Male genitalia: Penis in lateral aspect evenly curved, broad; extreme apex curved upwards and sharp (Fig. 255).

Female: Pro- and mesotarsus slender. Apical ventrite as in Fig. 60. Rarely microsculpture of body is strongly developed and such a specimen is clearly matter than ordinary female specimens.

##### Distribution.

Madagascar (Fig. 541).

##### Collecting circumstances.

In literature, detailed documentation is not available. From collecting labels appear that *Laccophilus complicatus* has been sampled in areas between 700-1700 m a.s.l. At least once collected in a degraded river with lots of vegetation, used to wash clothes in. Obviously it is attracted by light and sampled in a montane rain forest.

#### Laccophilus irroratus

Taxon classificationAnimaliaColeopteraDytiscidae

Aubé, 1838

Figs 61–62 , 256 , 415 , 535

Laccophilus irroratus Aubé 1838: 427 (original description, faunistics); Sharp 1882: 309 (description, faunistics); Kolbe 1883: 426 (description, faunistics); Régimbart 1895: 138, 141 (description, faunistics, discussion); Alluaud 1897: 212 (faunistics); Peschet 1917: 23, 24, 55 (description, faunistics); Zimmermann 1920a:20 (catalogue, faunistics); Guignot 1946c: 268 (description, faunistics); Vinson 1956: 29 (faunistics, list, biology); Guignot 1957a: 98 (faunistics); Guignot 1959a: 557, 560, 562 (description, faunistics); Guignot 1961a: 930 (faunistics); Vinson 1967: 314 (faunistics, list); Wewalka 1980: 729, 730 (faunistics); Bameul 1984: 93, 102 (faunistics); Nilsson 2001: 245 (catalogue, faunistics); Pederzani and Reintjes 2002: 40 (faunistics); Nilsson 2015: 213 (catalogue, faunistics).

##### Type locality.

Réunion: Ile de France et Bourbon.

##### Type material studied

(1 ex.): Holotype, male: “Data in NHRS JLKB 000030278 / *Laccophilus irroratus* mihi h. in ile de France D. Latereille / Ex Musaeo Dejean / D. Sharp Monogr. / *irroratus* / Dr. Régimbart 1893 / Coll. Oberthur” (MNHN).

##### Additional material studied

(21 exs.). **Mauritius**: “I. Maurice Montrésor Ch. Alluaud 1893 / male symbol / Museum Paris coll. Maurice Régimbart” (1 ex. MNHN); “I. Maurice Mon Désert P. Carie Fév. 1903 / *Laccophilus irroratus* Aubé / Museum Paris coll. 1945 R. Peschet” (1 ex. MNHN); “Ile Maurice / de Borre” (3 exs. MHNG); “Ins. Mauritius Westw.” (1 ex. ZMHB). – **Reunion**: “La Reunion Palmistes Ch. Alluaud 1893 / Museum Paris coll. Maurice Régimbart 1908 / *irroratus* Aubé” (2 exs. MNHN; habitus in Fig. 415); “S St. Benoit NW Cambourg, ca. 250 m 2-3.1. 1999 Wewalka / *Laccophilus irroratus* Aubé Wewalka det. 99” (2 exs. CGW, 1 ex. MZH); “E St. Joseph Riv. Langevin 250 m 28.12. 1998 leg. Wewalka / *Laccophilus irroratus* Aubé Wewalka det. 99” (4 exs. CGW, 4 exs. MZH); “Plaine des Cafres 3.3. 1935 Vinson” (1 ex. MNHN); “Ins. I. Fr. Dufr.” (1 ex. ZMHB).

##### Diagnosis.

*Laccophilus irroratus* is characterized by large body size, peculiar elytral colour pattern and penis, which is different from all other African *Laccophilus* species; penis in lateral aspect quite delicate, distinctly curved and simple, exhibiting minor modifications.

##### Description.

Body length 4.0–4.3 mm, breadth 2.4–2.6 mm. Dorsal, colour pattern of body stable, exhibits only minor variation (Fig. 415).

Head: Pale ferruginous, posteriorly close to pronotum with narrow but distinct, dark ferrugineous area. Rather shiny, although distinctly microsculptured. Reticulation double, large meshes contain 3–5 small meshes. In middle of head small meshes in part reduced. At eyes with scattered, fine punctures; area with punctures extends towards middle of head.

Pronotum: Pale ferrugineous to ferrugineous. Anteriorly, at level of eyes with a broad, black to dark ferrugineous area. At base with a quite narrow, black to dark ferrugineous area, which is medially somewhat enlarged. Delimitation of darker areas is somewhat vague. Rather shiny, although distinctly microsculptured; reticulation double. Large meshes distinct while small meshes, especially medially, are fine, in part hardly visible or totally absent. Punctures absent, except in frontal part and laterally where puncture very fine and scattered.

Elytra: Pale ferrugineous, with distinct, only slightly variable, dark ferrugineous irrorations (Fig. 415). One specimen with quite broad, transverse, pale area frontally. Rather shiny, although distinctly microsculptured; reticulation double. Large meshes contain, when discernible, 3–5 fine meshes. Fine meshes sometimes obliterated. Fine scattered punctures form a discal row of punctures. Scattered, irregular punctures indicate presence of dorsolateral and lateral rows of punctures. Laterally, with a pre-apical furrow.

Ventral aspect: Blackish ferrugineous to dark ferrugineous, no distinct colour pattern but three basal ventrites are somewhat paler than apical ones. Rather shiny, in part with very fine microsculpture. Almost impunctate, except apical ventrite, with scattered fine punctures, and frontally on metathorax, with fine, fairly dense punctures. Abdominal ventrites with fine, curved striae. Metacoxal plates with about 10 shallow and in part slightly indistinct furrows. Metacoxal process not modified. Apical ventrite, symmetric (no knob discernible) (Fig. 61). Prosternal process rather narrow, apex only slightly extended, pointed.

Legs: Pro- and mesotarsus slender, somewhat extended. Segments provided with suckers.

Male genitalia: Penis delicate, in lateral aspect distinctly curved; apex simple and exhibits hardly any modifications (Fig. 256).

Female: Pro- and mesotarsus slender. Apical ventrite (Fig. 62).

##### Distribution.

Mascarene Islands; Réunion, Mauritius (Fig. 535). Guignot (1957a) gives also Rodriguez. *Laccophilus irroratus* is an endemic species of Mascarene Islands (Guignot 1961a).

##### Collecting circumstances.

Almost unknown. Reported in rock pools of slow stream (Vinson 1956).

#### Laccophilus rivulosus

Taxon classificationAnimaliaColeopteraDytiscidae

Klug, 1833

Figs 63–64 , 257 , 416 , 542

Laccophilus rivulosus Klug 1833: 48 (original description, faunistics); Aubé 1838: 4 (description, faunistics); Sharp 1882: 287, 821 (description, faunistics); Kolbe 1883: 401 (description, faunistics); v. d. Branden 1885: 24 (catalogue, faunistics); Régimbart 1895: 136, 140 (discussion, description, faunistics); Régimbart 1903: 14 (faunistics); Zimmermann 1919: 122 (faunistics); Zimmermann 1920a: 25 (catalogue, faunistics); Guignot 1937: 141 (discussion); Guignot 1959a: 544 (description, faunistics); Bertrand and Legros 1971: 245 (faunistics, biology); Rocchi 1991: 86: (faunistics, list); Nilsson 2001: 250 (catalogue, faunistics); Nilsson 2015: 217 (catalogue, faunistics).

##### Type locality.

Madagascar.

##### Type material studied

(4 exs.): Lectotype (by present designation): male: “9982 / Typus / *Laccophilus rivulosus* Kl. Madag, Goudot / Hist-Coll. (Coleoptera) Nr. 9982 *Laccophilus rivulosus* Kl. Madagascar Goudot Zool. Mus. Berlin” (ZMHB). – Paralectotypes: Madagascar Goud. Nr. 9982 / Typus / Hist-Coll. (Coleoptera) Nr. 9982 *Laccophilus rivulosus* Kl. Madagascar Goudot Zool. Mus. Berlin” (3 exs. ZMHB).

##### Additional material, studied

(38 exs.). **Madagascar**: “Antakotako II 1936” (1 ex. MNHN; habitus in Fig. 416); “Mad. Sud, Ft Dauphin Alluaud 1900” (3 exs. MNHN); “Mad. Sud, Ft Dauphin Alluaud” (2 exs. MNHN); “Tamatave Perrot” (1 ex. Z MHB, 2 exs. MNHN); “Antsianaka Perrot Freres, 1er Semestre 1892” (1 ex. MNHN, 1 ex. NHMB, 6 exs. SAMC); “Antsianaka / *Laccophilus rivulosus* Kl. det. M. Brancucci” (1 ex. MNB); “Suberbieville” (1 ex. MNHN); “Mt. d’Ambre / Mai” (1 ex. ZMHB); “St. Marie Moaroay / Mad. Kaudern / *Laccophilus rivulosus* Kl. det. Zimmermann” (4 exs. NHRS); “Mahajanga Melaky, btw Bekopaka-Antsalova, S18.91556, E044.55546, 47 m.a.o. 16.12. 2009 water net, field Bergsten et al.” (2 exs. NHRS); same data but add “NHRS-JLKB 000000726” (1 ex. NHRS); “Mahajanga Melaky, btw Antsalova-Maintirano S18.30233, E044.18071, 37 m.a.o., 18.12. 2009, water net, field, Bergsten et al. /NHRS-JLBK 000000728” (1 ex. NHRS); “Mahajanga Boeny, Ankarafantsika NP, S16.30341, E046.81073, 74 m.a.o., 29.11. 2009, 22 W black light, field, Bergsten et al.” (1 ex. NHRS); same data but “S16.31215, E046.81523, 76 m.a.o., water net, field” (2 exs. NHRS); same data but “S16.31215, E046.81523, 76 m.a.o., water net, field/NHRS-JLKB 000000724” (1 ex. NHRS); “Mahajanga Boeny, Mahavavy Kinkony RS, S16.06651, E045.77672, 24 m.a.o., 5.12. 2009, water net, field, Bergsten et al./NHRS-JLKB 000000727” (1 ex. NHRS); “Toliara Menabe, Kirindy R. S., S20.07641, E044.674708, 65 m.a.o., 11.12. 2009 water net, field, leg. Bergsten et al” (2 exs. NHRS); “FIAN, Isalo, Piscine Noir, hygropetric 12.5. 2006 Bergsten et al. /BMNH(E) <794159> DNA voucher / *Laccophilus rivulosus* Kl. det. Bergsten” (1 ex. NHRS); “Madagascar Fairmaire / *Laccophilus rivulosus* Kl. Madg.” (1 ex. NMW); “Madag. Perrier” (2 exs. SAMC).

##### Diagnosis.

*Laccophilus rivulosus* is characterized by large body, peculiar elytral colour pattern and shape of penis. Dark longitudinal markings of elytra are quite broad and distinct. Pale irrorations can generally be discerned within dark, longitudinal marking. Penis resembles much of penis of *Laccophilus posticus* but it is somewhat larger and extreme apex more extended. Additionally large body size and clear differences in elytral colour pattern easily separates *Laccophilus rivulosus* from *Laccophilus posticus*.

##### Description.

Body length 4.8-5.3 mm, width 2.7-3.0 mm. Dorsal colour pattern of body generally distinct and uniform (Fig. 416); rarely slightly variable.

Head: Pale ferrugineous, posteriorly between eyes with a distinctly delimited blackish ferrugineous to dark ferrugineous area. Rarely dark area reduced and only in part visible because hidden beneath frontal part of pronotum. Almost impunctate. At eyes in shallow depression with fine, irregularly distributed punctures. Submat, rather distinctly microsculptured. Reticulation double. Coarser meshes only slightly stronger in comparison with fine meshes. In part, kinds of meshes difficult to separate. When discernible coarse meshes contain 3-5 fine meshes.

Pronotum: Pale ferrugineous, frontally with a distinct, dark ferrugineous area. Posteriorly in middle with an, often, bilobed, dark ferruginoeus, narrow spot. Almost impunctate. No distinct punctation discernible. Submat, finely microsculptured. Reticulation double. Coarse meshes well developed, contain 2-5 fine meshes.

Elytra: Pale ferrugineous, with distinct, longitudinal, dark ferrugineous markings. Dark markings somewhat undulate and merged into pairs, forming pale, inner, irrorations (Fig. 416). Rarely undulation in anterior half of elytra indistinct because of expanded dark areas. Almost impunctate; an indistinct, discal row of punctures discernible. Rather shiny, although distinctly microsculptured. Reticulation double. Coarse meshes contain 2-5 fine meshes. Narrowly at suture elytra slightly elevated.

Ventral aspect: Pale ferrugineous. Almost impuntate; abdomen with a few scattered punctures. Rather shiny, although extensively, finely microsculptured. In part microsculpture almost obliterated or totally obliterated. Abdomen laterally with fine, curved striae. Apical ventrite as in Fig. 63. Prosternal process rather slender, apex pointed but not strongly extended posteriorly. Metacoxal plates with about 10 furrows, of which 2–3 closest to metasternal wing are distinct while others rather indistinct to indistinct.

Legs: Pro- and mesotarsus slightly enlarged, somewhat extended and provided with some protruding suckers.

Male genitalia: Penis quite robust, in lateral aspect distinctly curved and apex extended and sharp (Fig. 257).

Female: Apical ventrite as in Fig. 64. Pro- and mesotarsus rather slender, extended.

##### Distribution.

Endemic for Madagascar (Fig. 542).

##### Collecting circumstances.

Information on biology is almost totally lacking. Bertrand and Legros (1971) reports the species from a small swamp and from pools of a temporary river.

### Species group 8 (*Laccophilus immundus* group)

**Diagnosis.** Quite large species with body length 4.3–4.5 mm, width 2.3–2.5 mm.

Body shape oval-oblong, dorsoventrally flattened (Fig. 417). Body colour ferrugineous to dark ferrugineous. Lacks distinct colour pattern (Fig. 417). Body microsculpture double, distinctly of two kinds.

Prosternal process slender, slightly extended, apically pointed. Apical ventrite modified; posteriorly on each side somewhat excavated; medially, posteriorly extended, lacks asymmetrical knob on one side of ventrite (Fig. 65). No stridulatory apparatus on metacoxal plates. Metacoxal process, posteriorly not extended (Fig. 6).

Paramere apically narrow, basally enlarged; moderately modified (Fig. 258). Apical part of penis straight to almost straight; apex resembles a harpoon (Fig. 258).

**Species composition and distribution.** One species is recognized and distributed in South Africa.

#### Laccophilus immundus

Taxon classificationAnimaliaColeopteraDytiscidae

Sharp, 1882

Figs 65–66 , 258 , 417 , 543

Laccophilus immundus Sharp 1882: 304 (original description, faunistics, discussion); v. d. Branden 1885: 21 (catalogue, faunistics); Régimbart 1895: 131 (description, faunistics); Zimmermann 1920a: 20 (catalogue, faunistics); Guignot 1959a: 579 (description, faunistics); Omer-Cooper 1962: 294, 296 (faunistics, discussion); Omer-Cooper 1965: 76, 81 (description, faunistics); Nilsson 2001: 244 (catalogue, faunistics); Nilsson 2015: 212 (catalogue, faunistics).
Laccophilus spadix Omer-Cooper 1953: 23 (original description, faunistics, biology); Omer-Cooper 1965: 81 (list, synonymy); Nilsson 2001: 244 (catalogue, list, synonymy); Nilsson 2015: 212 (catalogue, faunistics, list, synonymy). **Confirmed synonym.**

##### Type localities.

*Laccophilus immundus*: South Africa: Cape Town.

*Laccophilus spadix*: South Africa: Cape Province, Caledon district.

##### Type material studied

(4 exs.). *Laccophilus immundus*: Holotype: female: “Type / S. Africa / Sharp Coll. 1905-313 / Type 595 *Laccophilus immundus* sp. n. Capetown” (BMNH). – *Laccophilus spadix*: Holotype: male: “Type / W Cape P., stream with pools in pine wood, Caleda dist. 18.XI. 1947 J. Omer-Cooper / Brit. Mus. 1957-660 / *Laccophilus spadix* O-C. det. J. Omer-Cooper” (BMNH). – Paratypes: female: “Type / Cape Peninsula, small vlei nr Cape Town 18.XI. 1947 J. Omer-Cooper / Brit. Mus. 1957-660 / *Laccophilus spadix* O-C. det. J. Omer-Cooper female allotype” (1 ex. BMNH); “W. Cape Pr., stream with pools in pine wood Caledon distr. 18.XI. 1947 JOC. / *Laccophilus spadix* O-C.” (1 ex. AMGS).

##### Additional material studied

(945 exs.). **South Africa**: “Western C. Pr. stream with pools in pine wood Caledeon Dist. 18.XI. 1947 Omer-Cooper” (1 ex. AMGS); “Pools in Caledon 11. 1947” (14 exs. AMGS); “WC. Prov. Princess vlei Cape Town 13.IV. 1947”(1 ex. AMGS); “Cap. b. sp. De Vylder / *Laccophilus immundus* Shp det. A. Zimmermann” (5 exs. NHRS); “Cape Prov., Cape Flats, Varden Vlei 2 mi E Ottery, 2.2. 1951 / *Laccophilus spadix* = *Laccophilus immundus* det. J. Omer-Cooper” (3 exs. MZLU; habitus in Fig. 417); “Cape prov., Silversand, 8 mi W Kleinmond 19.12. 1950” (1 ex. MZLU); “Cape Good Hope Nature Reserve 7-10.3. 1968 Spangler” (422 exs. USNM, 20 exs. MZH); “CPr., Cape of Good Hope 8.1. 1994 Wewalka” (2 exs. CGC); “WC, Cape point Res., Cape peninsula 28.8. 2007, dam, Pryke leg., 34.30947S, 18.44977E” (2 exs. CCT); “WC, Cape of Good Hope 20.3. 2001, pond 2 km N field museum, Ribera et Cleslak” (1 ex. CIR); “WC, Cape of Good Hope Reserve, pond, 26.2. 1997 Turner” (60 exs. CCT, 6 exs. MZH); same data but “16.2. 1997” (100 exs. CCT); “WC, Cape Point Reserve, S341436, E182306, 30.8. 2003, seasonal pool with fibrous vegetation, Turner, Mann & Reavell” (46 exs. CCT, 6 exs. MZH); “WC, Cape Point Res., seasonal pool, 3418S, 182631E, alt. 87 m, Turner, Mann & Reavell” (25 exs. CCT); “WC, Cape Town, Cape of Good Hope Res., pond 26.2. 1997 Turner” (166 exs. CCT); same data but “Reservoir on roadside to Bordjlesri 15.2. 1997 Turner” (55 exs. CCT); “WC, Kromrivier at roadsite, Cape G.H. Res. 15.2. 1997 Turner” (1 ex. CCT); “WC, Schusters Kraal, stream, netted between footbridge and dam, 341223S, 182237E, 13.9. 2003, Turner, Mann & Reavell” (8 exs. CCT).

##### Comments on synonymy.

Holotypes of both involved taxa have been examined and compared. No diagnostically important differences detected and accordingly, earlier introduced synonymy is confirmed. *Laccophilus immundus*, being older than *Laccophilus spadix*, is the valid name of the species.

##### Diagnosis.

A peculiar species, which is especially characterized by shape of penis apex (harpoon-like) and by body, being almost one-coloured, piceous to dark brownish or dark ferrugineous.

##### Description.

Body length 4.3–4.5 mm, width 2.3–2.5 mm. Dark ferrugineous to ferrugineous, dorsal colour pattern of body is vague, reduced and rather indistinct (Fig. 417).

Head: Pale ferrugineous to ferrugineous, posteriorly and at eyes dark ferrugineous; delimitation of colour pattern vague. Rather finely to finely and densely punctate. Close to eyes with a few coarser punctures. Submat, distinctly microsculptured. Meshes double; larger meshes include 2-5 fine meshes.

Pronotum: Ferrugineous, basally in middle with a dark ferrugineous marking. Punctures fine to very fine, rather dense and slightly irregularly distributed. At margins with a partly irregular row of punctures. (In part, row frontally replaced by rather narrow area of fine punctures.) Microsculpture distinct, dense and double: coarser meshes include 2-6 finer meshes.

Elytra: Dark ferrugineous, vague darker markings sometimes discernible but form no distinct colour pattern (Fig. 417). Finely and densely punctate. Very sparse (indistinct) longitudinal rows of slightly coarser punctures discernible. Laterally in posterior half along the edge of elytron punctures form a narrow furrow. Submat, microsculpture double; coarse meshes include 2-6 fine meshes.

Ventral aspect: Pale ferrugineous to ferrugineous. Almost impunctate. In part, very finely microsculptured. Metacoxal plates, especially in frontal part with rather distinct furrows. Apical ventrite lacks “one-side” asymmetric knob (Fig. 65). Ventrites with curved striae. Prosternal process slender and pointed.

Legs: Pro-and mesotarsus slightly enlarged, provided with suckers.

Male genitalia: Penis quite long and slender, slightly sinuate and apex “harpoon-like” (Fig. 258).

Female: As male but apical ventrite slightly different in shape (Fig. 66). Pro- and mesotarsus slender.

##### Distribution.

South Africa (Fig. 543).

##### Collecting circumstances.

Poorly documented. Label data gives various water bodies as pond, stream with pools in pinewood as a collecting sites, but available information is generally quite superficial.

### Species group 9 (*Laccophilus pellucidus* group)

**Diagnosis.** Large species; body length 5.3–6.0 mm, width 3.0–3.4 mm.

Shape of body oval, dorsoventrally distinctly flattened (Fig. 418). Dorsal colour pattern diffuse, sometimes almost absent. Rarely epipleura of female distinctly expanded posterior to middle (Fig. 8). Often elytra exhibit rather dense, dark ferrugineous to ferrugineous irrorations, which laterally in part become indistinct. A few pale spots generally present at base and apicolaterally on elytra (Fig. 418). Dorsal microsculpture double, of two different kinds; in part reticulation is obscured and indistinct.

**Figures 67–74.**
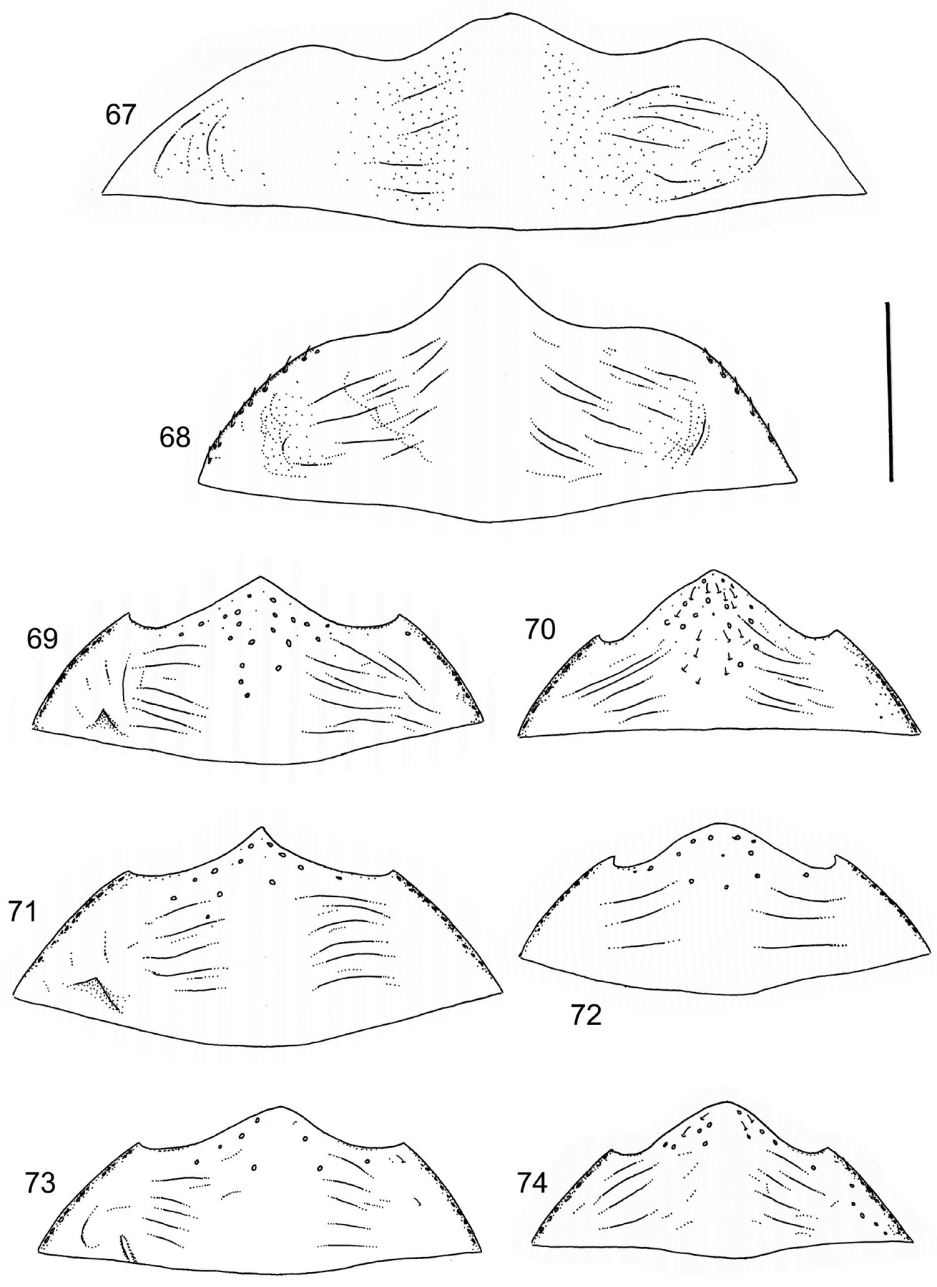
Apical ventrites **67** *Laccophilus pellucidus* male, and **68** female **69** *Laccophilus adspersus* male, and **70** female **71** *Laccophilus olsoufieffi* male, and **72** female **73** *Laccophilus modestus* male, and **74** female. Scale bar 0.5 mm.

Prosternal process quite broad, rather short, apex pointed. Apical ventrite somewhat modified; posterior end of ventrite excavated on both sides and medially ventrite somewhat extended. Asymmetric knob on one side of ventrite lacking (Fig. 67). Stridulatory apparatus absent. Metacoxal process not extended posteriorly (Fig. 6).

Parameres moderately modified. Penis is exceptionally large with apex strongly modified; clearly different from all other African species (Fig. 259).

**Species composition and distribution.** One species recognized in this species group, distributed in Africa South of Sahara excluding Madagascar.

#### Laccophilus pellucidus

Taxon classificationAnimaliaColeopteraDytiscidae

Sharp, 1882

(Figs 8 , 67–68 , 259–260 , 418 , 560)

Laccophilus pellucidus Sharp 1882: 304 (original description, faunistics); v. d. Branden 1885: 23 (catalogue, faunistics); Régimbart 1895: 131 (description, faunistics); Zimmermann 1920a: 24 (catalogue, faunistics); Guignot 1959a: 579, 584 (description, faunistics); Omer-Cooper 1962: 295 (faunistics); Omer-Cooper 1965: 76, 87 (description, discussion, faunistics, synonymy *Laccophilus ampliatus* Régimbart and *Laccophilus pellucidus*); Omer-Cooper 1967: 60 (discussion); Biström 1979: 22 (faunistics); Pederzani 1988: 107 (faunistics, biology); Nilsson and Persson 1993: 82, 94 (faunistics, biology); Rocchi 2000: 25 (discussion, description); Nilsson 2001: 248 (catalogue, faunistics); Nilsson 2015: 215 (catalogue, faunistics).
Laccophilus ampliatus Régimbart 1895: 130 (original description, faunistics); Régimbart 1906: 248 (faunistics); Zimmermann 1920a:16 (catalogue, faunistics); Omer-Cooper 1958b: 37, 48, 49, 51 (discussion, description, faunistics, biology, synonymy *Laccophilus ampliatus* and *Laccophilus pilitarsis* Régimbart); Omer-Cooper 1965: 87 (description, faunistics, synonymy *Laccophilus ampliatus* and *Laccophilus pellucidus* Sharp); Omer-Cooper 1967: 60 (discussion, list, synonymy); Nilsson and Persson 1993: 82 (list, synonymy); Nilsson 2001: 24 (catalogue, faunistics, list, synonymy); Nilsson 2015: 215 (catalogue, faunistics, list, synonymy). **Confirmed synonym.**
Laccophilus pilitarsis Régimbart 1906: 247 (original description, faunistics); Régimbart 1908: 5 (faunistics); Zimmermann 1920a: 24 (catalogue, faunistics); Peschet 1921: 5, pl. 1 fig. 5 (discussion, description, faunistics); Omer-Cooper 1931: 756 (description, biology, faunistics); Guignot 1946c: 282, 283, 284, 312 (description, faunistics); Guignot 1954: 29 (faunistics); Omer-Cooper 1957: 21, 90 (faunistics); Omer-Cooper 1958a: 59 (faunistics); Omer-Cooper 1958b: 37 (synonymy *Laccophilus ampliatus* Régimbart); Guignot 1959a: 576, 581 (description, faunistics); Guignot 1959b: 355 (faunistics); Guignot 1959d: 162 (faunistics); Bertrand and Legros 1975: 672 (discussion); Nilsson and Persson 1993: 82 (list, synonymy *Laccophilus pellucidus* Sharp); Nilsson 2001: 248 (catalogue, faunistics, list, synonymy); Nilsson 2015: 215 (catalogue, faunistics, list, synonymy). **Confirmed synonym.**

##### Type localities.

*Laccophilus pellucidus*: South Africa: Natal, Bedford District.

*Laccophilus ampliatus*: South Africa: Natal.

*Laccophilus pilitarsis*: Kenya: Taveta.

##### Type material studied

(6 exs.). *Laccophilus pellucidus*: Holotype (type unique): male: *“Laccophilus pellucidus* Type D.S. Bedford District, Caffraria 175 *Laccophilus* / Type H.T. / Sharp Coll. 1905-313 (BMNH).

*Laccophilus ampliatus*: Lectotype (by present designation): female: “Natal / Museum Paris Coll. Maurice Régimbart 1908 / Type / *ampliatus* Rég. type” (MNHN). – Paralectotype: female: “98 / *Laccophilus ampliatus* Rég. type unique / SAM Type acc. no. 838” (1 ex. SAMC). [Comment: note that Régimbart, when describing *Laccophilus ampliatus*, indicated presence of both sexes in type material – both are, however, females.]

*Laccophilus pilitarsis*: Lectotype (by present designation): male: “Afrique Orle Anglaise Taveta Ch. Alluaud I-IV. 1904 / Museum Paris coll. Alluaud / Type” (MNHN). – Paralectotypes: “Afrique Orle Anglaise Nairobi (Wa Kikuyu et Masai) Ch. Alluaud 2. sem. 1903 / Museum Paris coll. Alluaud / Type” (2 exs. MNHN).

##### Additional material studied

(413 exs.). **Ethiopia**: “Abyssinia Hora Bishoftu 7000 ft. 23.XII. 1926 JOC.” (3 exs. AMGS); “Hora Horeso 7000 ft. 1.12. 1926 JOC.” (12 exs. AMGS); “Small pond Hora Abjata 5000 ft. 18.XI. 1927 JOC.” (2 exs. AMGS). – **Sudan**: “S. Sudan R. Yei at Amadi 28.1. 1954 JJOC.” (1 ex. AMGS); “S. Sudan stream from hot springs Nyangwara 30,5E 4,39N 29.1. 1954 JJOC.” (6 exs. AMGS); “S. Sudan R. Yel at Amadi 28.1. 1954 JJOC.” (1 ex. AMGS); “S Sudan, sandy river 50 mi NW Juba 29.1. 1954 J. & J. Omer-Cooper” (5 exs. AMGS); “Equatoria Mundri-Lalyo 25-26.2. 1963 Linnavuori” (1 ex. MZH); “Blue Nile Ingessana Mts. 17-22.11. 1962 Linnavuori” (2 exs. MZH); “Equatoria Lalyo-Juba 26-27.2. 1963 Linnavuori” (7 exs. MZH). – **Uganda**: “Madi V. 1927 Carpenter” (1 ex. AMGS). – **Kenya**: “Selengai Riv. 21.6. 1970 E.S. Brown” (2 exs. BMNH); “River Athi Bushwackers´ Camp 29.3. 1964 E.S. Brown” (1 ex. BMNH); “Athi River Machakos District 14.9. 1976 M. Holmen / *Laccophilus pilitarsis* Rég. det. M. Holmen” (1 ex. ZMUC); “Nairobi Natnl Park 22.3. 1953 Hippo pools E.S. Brown” (1 ex. BMNH); “Kibwezi River Machakos District 13.9. 1976 Holmen M. / *Laccophilus pilitarsis* Rég. det. Holmen 1976” (8 exs. ZMUC); “Cha Shimba R., Kwale Kwale District 18.9. 1976 M. Holmen” (3 exs. ZMUC); “Mwatsuma R. Mariakani Kilifi District 16.9. 1976 M. Holmen” (2 exs. ZMUC); “Manjewa Riv Mariakani Kilifi / Kwale District 16.9. 1976 M. Holmen” (5 exs. ZMUC); “Maji ya Chumwi River Kwale District 16.9.1976 M. Holmen / *Laccophilus pilitarsis* Rég. det. M. Holmen 1976” (3 exs. ZMUC) “Afrique Orle Anglaise Voi Ch. Alluaud 1909 / Septembre” (2 exs. MNHN); “Afrique Orle Anglaise Voi Ch. Alluaud 1909 / Septembre / Det. Dr. Guignot *Laccophilus pilitarsis* Rég.” (1 ex. MNHN); “Fort Hall Br. O. A.” (1 ex. NHMB, 1 ex. ZMHB); “Mulango Br. O. A.” (1 ex. ZMHB);” Br. O. A. Kibwezi Scheffler” (1 ex. ZMHB). – **Tanzania**: “Mkulumuzi Riv. Paramba, Tanga District 26.9. 1976 M. Holmen / *Laccophilus pilitarsis* Rég. det. Holmen det.” (1 ex. ZMUC); “Sigi Riv. Ralley Estate Tanga District 26.9. 1976 M. Holmen / *Laccophilus pilitarsis* Rég. det. M. Holmen” (1 ex. ZMUC); “Kombe Stream Doda Tanga District 23.9. 1976 M. Holmen / *Laccophilus pilitarsis* Rég. det. Holmen” (2 exs. ZMUC); “TPC S Moshi canals 28.9. 1976 / *Laccophilus pilitarsis* Rég. det. Holmen” (3 exs. ZMUC); “Stream of Hegongo Tanga District 22.9. 1976 M. Holmen / *Laccophilus pilitarsis* Régb. det. Holmen” (1 ex. ZMUC); “Uluguru Mts. Kimboza Forest 250 m 18.7. 1981 Stoltze & Scharff leg. / *Laccophilus pilitarsis* Rég. det. Holmen 1981” (1 ex. ZMUC); “Kilimandj. Sjöstedt / Kibonoto 1-1200 m” (4 exs. NHRS); same data but “1000-1300 m” (1 ex. ZMHB); “? einem Tümpel nahe am Myanwaya Fluss 24.5. 1899” (1 ex. NHMB). – **Zaire**: “C.B. PNU, Kamusanga affl. g. Lufira / f. mt. Sombwe (750 m) 12.VII. 1949 Mis. De Witte 2776a” (1 ex. MNHN). – **Zambia**: “Muchinga Escarpment ca. 47 km ENE Rufunsa 14°57'S, 30°04'E lux Göllner leg. 25.3. 1993” (1 ex. ZMHB); “Mountain stream crossing road Kafue-Chirundu 9.8. 1986 Pederzani / *Laccophilus pellucidus* Sharp det. Rocchi 1990” (1 ex. CSR). – **Malawi**: “7 km W Golomoti SE14,34Bc 11.12. 1983” (2 exs. TMSA); “Nyasaland Ft. Hill Yambe Stream 17.10. 1948 JOC.” (1 ex. AMGS); “Nyasaland 40 mi. from Njakwa on Ft Hill rd 18.10. 1948” (1 ex. AMGS); “Nyasaland stream 20 miles from Dedza on Lower Lilongwe rd 30.9. 1948” (1 ex. AMGS); “Nyasaland Mtiti River 1.X. 1948 JOC.” (2 exs. AMGS); “Selima env. 80 km E Lilongwe 5-6.1. 2002 Kantner” (3 exs. NHMB); “Mulanje mnts env. 22-26.12. 2001 Kantner” (2 exs. NHMB). – **Mozambique**: “Mkura Stream on Chikukwa Camp, N-19.5516, E33.06916, 24.9. 2002 Bills” (1 ex. AMGS). – **Zimbabwe**: “S. Rhod., stream with lilies betw. Salisbury & Bromley 12.IX. 1948” (2 exs. AMGS); “Rhodesia Sebakwe” (2 exs. SAMC); “Shangani R. / 13.9. 1948 JOC. / *Laccophilus pilitarsis* Rég. det. J. Omer-Cooper” (2 exs. AMGS, 1 ex. TMSA); “Wankie Nat. Pk. Main Camp nr Pan S. / MV light trap 10.11. 1961 J.S. Weir” (1 ex. BMNH, 1 ex. MZH; habitus in Fig. 418); “S. Rhod. Wankie reserve Masumu dam 4.IX. 1948” (2 exs. AMGS). – **Swaziland**: “Little Usutu River nr. Mbabane 5.12. 1948 JOC.” (1 ex. AMGS). – **South Africa**: “Tv. Nelshoogte gallery forest below St. 25.51S-30.53E / 4.12. 1986 UV light collection Endrödy-Younga 2354” (2 exs. TMSA, 1 ex. MZH); “Kruger Nat. Pk Letaba Riv. bel. dam 23.46S-31.30E / shorewashing 1.3. 1995 Endrödy-Younga 3122” (4 exs. TMSA, 1 ex. MZH); “Kruger Nat. Pk Levuvu Riv.22.27S-31.10E / 12.2. 1994 shorewashing Endrödy-Younga 2998” (1 ex. TMSA); “Trsvl KNP, Gudzani, N-24.260, E31.840, 30.6. 1960” (1 ex. AMGS); “Trsvl, Wit R at Plaston, N-25.350, E31.070, 25.6. 1960” (3 exs. AMGS); “Trsvl, Polluted Pan, S of Olifants Gorge, KNP, N-24.010, E31.740 28.6. 1960” (1 ex. AMGS); “Trsvl, Satara KNP, Windmill dam, N-24.400, E31.770, 30.6. 1960” (2 exs. AMGS); “KNP survey Shingwedzi 19-20.11. 1961 Vári & Rork” (1 ex. TMSA); “Trsvl Warmbad 24-25.2. 1968 Spangler” (21 exs. USNM, 4 exs. MZH); “Trsvl Bundu Inn 25.28S-28.55E / 24.3. 1974 shore washing Endrödy-Younga 304” (1 ex. TMSA); “Fountains Pta 5.11. 1932 G. van Son” (7 exs. TMSA); “Transvaal Koop R. Barberton 5. Dec. 1948” (1 ex. AMGS); “OFS Parys” (1 ex. SAM); “Zululand Hluhluwe Game Res. 28.05S-32.04E / 27.11. 1992 shorewashing, shade, E-Y 2861” (2 exs. TMSA); “Natal Hluhluwe Game Reserve 18.4. 1951/Brinck-Rudebeck” (2 exs. MZLU); “Natal R. Natal National Park, the Hostel 5.4. 1951/Brinck-Rudebeck” (8 exs. MZLU); “Zululd Mtubatuba 28.22S-32.19E / 4.4. 1974 muddy shore washing, Endrödy-Younga 319” (3 exs. TMSA, 1 ex. MZH); “Natal Richmond 4.7. 1947 J.O.C.” (3 exs. AMGS, 8 exs. USNM, 1 ex. MZH); “Natal Little Bushmans River 15.4. 54 Oliffe” (2 exs. AMGS); “Umzikulu 6.4. 1947 J.O.C.” (3 exs. AMGS); “Natal Celenso (?) 12.11. 1953 / Tug 36Q3 12.11.53” (1 ex. AMGS); “Kw. Natal, Mhlatuze R above weir, Felixton N-28.840, E31.910, 7.3. 1962” (7 exs. AMGS); “Kw. Natal, below Masonite Effluent. Estcourt, Little Bushmans R., N-29.000, E29.880, 15.4. 1954 Oliff” (1 ex. AMGS); “Kw. Natal, Lufafa R confluence, N-30.001, E30.1825, 5.5. 1996 Dickens & de Moor” (2 exs. AMGS); “Kw. Natal, Mooi R, at Hornet Corner, N-28.950, E30.380, 15.3. 1995 Dickens” (3 exs. AMGS); “Kw. Natal, Mooi R, at Glenfem Bridge, N-29.390, E29.810, 15.3. 1995 Dickens” (1 ex. AMGS); “Kw. Natal, Klein Mooi R, at Durleigh Farm, N-29.230, E29.900, 15.3. 1995 Dickens” (1 ex. AMGS); “Kw. Natal, Betw. Aberfoyle, Impofana Farms, Mpofana R. N-29.400, E30.070, 3.1. 1995 Dickens” (1 ex. AMGS); “Kw. Natal, Mooi R., above Rosetta N-29.320, E29.970, 15.3. 1995 Dickens” (26 exs. AMGS); “Kw. Natal, Lions R, at Weltwreden Farm, N-29.440, Dickens” (1 ex. AMGS); “NW Pr., twin streams in Siyai, 10.3.1981 Reawell” (5 exs. AMGS); “Kw. Natal, Richards Bay, Umhlatuze floodplain 7.6. 1985 Reavell” (4 exs. AMGS); “Kw. Natal, Volkrust Road Bridge, Ncandu R., N-27.750, E29.930, 4.12. 1973 Metz” (6 exs. AMGS); same data but “24.9. 1974” (1 ex. AMGS); same data but “20.5. 1974” (1 ex. AMGS); same data but “8.1. 1974” (2 exs. AMGS); same data but “19.3. 1974” (2 exs. AMGS); Kw. Natal, Ncandu R. St. 7, 27.8. 1974 Metz” (2 exs. AMGS); “Kw. Natal, Ngagane R., below Newcastle Sewage works, N-27.720, E30.020, 5.12. 1973 Metz” (1 ex. AMGS); same data but “20.3. 1974” (1 ex. AMGS); “Kw. Natal, Ngagane R., Mandini-Iscor Rd. Bridge, N-27.720, E30.060, 24.9. 1974 Metz” (1 ex. AMGS); “Kw. Natal, Ngagane R., Steildrift rd., N-27.770, E30.020, 19.6. 1974 Metz” (1 ex. AMGS); “Kw. Natal, Injambili R, inland S coast rd., N-30.620, E30.520, 7.6. 1972 Chutter” (20 exs. AMGS); “Kw. Natal, Amahlongwa R, S Coast Rd. N-30.250, E30.720,9.6. 1972 Chutter” (12 exs. AMGS); “Kw. Natal, Izotsha R., Inland Coast Rd. 5.6. 1972 Chutter” (10 exs. AMGS); “Kw. Natal, Buffalo R, at Retreat, N-27.720, E30.180, 7.11. 1973 Metz” (1 ex. AMGS); “Kw. Natal, Little Amazimtoti R. N-30.060, E30.820, 15.6. 1984 Pretorius” (5 exs. AMGS); “Kw. Natal, Stn 10 at Colenso, Tugela R., N-28.730, E29.820, 12.11. 1953 Oliff” (2 exs. AMGS); “Kw. Natal, Ingane R., old S Coast Rd., N-30.170, E30.780, 15.6. 1964 Pretorius” (17 exs. AMGS); “Coward’s Bus Dam 26.6. 1993 Reavell” (9 exs. AMGS); “Nyebo Stream Transkei 5.4. 1947 J.O.C.” (2 exs. AMGS); “Butterworth Riv. 16.4. 1947 J.O.C.” (4 exs. AMGS); “Freddy van Zyl Bridge, Oorslas Spruit 25.2. 1947 J.O.C.” (1 ex. AMGS); “Modder River” (1 ex. SAMC); “Z.A. Barbeton Distr. Suid Kaap R. / Humus Oct. 1961 / Leleup” (1 ex. TMSA); “East London Fort Jackson pond by railway 14.III. 1955” (3 ex. AMGS); “E.C.Pr. Plutos Vale 20 July 1946 J.O-C.” (1 ex. AMGS); “ECPr., close to entrance of Dwesa Nature Reserve, stone bottomed river edge, S32°15.602, E28.46.368, Alt. 27 m, 23.1.1905 Bergsten leg.” (2 exs. NHRS); “Albany Blouwkrantz 1939” (1 ex. AMGS); “WPr., Mouth Swart R entering Hartebeesport Dam, in *Eichhornia* beed 30.5. 1971 Reavell (1 ex. AMGS); “CPr., Swart Kei R, nr Tylden, N-32.110, E27.020, 4.2. 1973 Stuart & Greig” (3 exs. AMGS); “ECPr., Bloukrans R. GHT Pt Alfred Road, N-33.375, E26.705, 9.9. 1972 Stobbs” (1 ex. AMGS); “ECPr., Mncotsho R., N-32.54.48, E27.36.52, 11.8. 2003, De Moor & Barber-James” (1 ex. AMGS); “ECPr., Mncotsho R, Trib. Buffalo R., N-32.54.48, E27.36.52, 11.2. 2003, De Moor & Barber” (1 ex. AMGS); same data but “N-32.54.43, E27.36.48, 18.5. 2004” (1 ex. AMGS); same data but “N-32.54.43, E27.36.48, 30.8. 2001 De Moor & De Moor” (2 exs. AMGS); “ECPr., Xolo R, Trib. carrying sewage works discharge, N-32.50.11, E27.37.49, 11.12. 2003 De Moor & Barber-James” (1 ex. AMGS); same data but “19.5. 2004” (3 exs. AMGS); same data but “2.10. 2002” (1 ex. AMGS); same data but “20.2. 2002” (1 ex. AMGS); “ECPr., Xolo R., upstream of confluence with trib., N-32.50.15, E27.37.48, 8.6. 2000 De Moor & Barber-James” (2 exs. AMGS); same data but “30.8. 2000” (1 ex. AMGS); “ECPr., Xolo R. Dam at Lily Stone Farm, N-32.52.10, E27.38.34, 16.5. 2001, De Moor & Barber-James” (1 ex. AMGS); “ECPr., Xolo R. Tributary, N-32.50.11, E27.37.49,1.12. 2004 De Moor & Barber-James” (1 ex. AMGS); “ECPr., Xolo R. Dam on Sidney Hill Farm, N-32.52.00, E27.37.09, 1.4. 2000, De Moor & Barber-James” (2 exs. AMGS); “ECpr., Rwantsa R., Dam, N-32.53.20, E27.37.55, 30.8. 2000, De Moor & Barber James” (1 ex. AMGS); “ECpr., Rwantsa R. at Farm Wolsley, N-32.54.03, E27.41.51, 2.10. 2002 De Moor & Barber-James” (1 ex. AMGS); same data but “10.12. 2003” (4 exs. AMGS); “ECpr., Rwantsa R. dam at Farm Mistrey, N-32.53.20, E27.37.55, 12.10. 2002 De Moor & Barber-James” (1 ex. AMGS); same data but “1.10. 2002” (2 exs. AMGS); same data but “15.5. 2001” (1 ex. AMGS); same data but “11.12. 2003” (5 exs. AMGS); “ECPr., Nahoon River at Witchkranz, N-32.51.10, E27.39.08, 19.5. 2004 De Moore & Barber-James” (2 exs. AMGS); same data but “4.5. 2000” (1 ex. AMGS); same data but “Nahoon R system 13.8. 2003” (2 exs. AMGS); “ECPr. Nahoon R. ca. 100 m above Dabadaba R confluence, N-32.50.28, E27.39.21, 10.12. 2003, De Moor & Barber-James” (1 ex. AMGS); same data but “19.2. 2002” (3 exs. AMGS); “ECPr., Maclear Munic. Dam, Mooi R. trib., N-31.0561, E28.31472, 27.3. 1993 Scott & al.” (3 exs. AMGS); “C.Pr., Upper Gatberg River, at Madun N-31.270, E28.170, 24.3. 1991 Barber-James & De Moor” (1 ex. AMGS)

##### Comments on synonymy.

Earlier introduced synonymy of *Laccophilus pellucidus*, *Laccophilus ampliatus* and *Laccophilus pilitarsis* is confirmed. *Laccophilus pellucidus*, being the oldest, is the valid name of the species.

##### Diagnosis.

*Laccophilus pellucidus* is especially characterized by large body combined with very peculiarly shaped penis, different from all other African *Laccophilus* species; penis voluminous with twisted, apical extension.

##### Description.

Body length 5.3–6.0 mm, width 3.0–3.4 mm. Dorsal, aspect of body pale ferrugineous, without distinct colour pattern (Fig. 418). Elytra sometimes provided with pale, vague spots. Additionally, vague irrorations may be discerned on elytra.

Head: Pale ferrugineous. Slightly mat and distinctly microsculptured; reticulation double; large meshes contain 2–6 finer meshes. At eyes with an irregular row of fine punctures.

Pronotum: Pale ferrugineous, laterally with vague paler areas. Slightly mat, with distinct microsculpture. Reticulation generally of two different kinds; larger meshes contain 2–7 small meshes. At margins, with scattered, fine and irregular punctures.

Elytra: Pale ferrugineous to ferrugineous, without distinct colour pattern; sometimes with vague paler areas/spots (Fig. 418). Epipleuron posterolaterally not expanded. Weakly developed, fine irrorations often discernible. Rather shiny although finely reticulated. Reticulation double; large meshes contain 2-6 finer meshes. Posterolaterally double reticulation obscure and in part indistinct. Discal, dorsolateral and lateral, rather fine and somewhat irregular row of punctures discerned. Elytron posterolaterally with a row of fine and dense, quite long hairs.

Ventral aspect: Almost impunctate, except base of metathorax and two sclerites covered by apical ventrite; with distinct punctures. Rather shiny, extensive and very fine microsculpture discernible. Metacoxal plates and base of abdomen with striae. Prosternal process pointed, comparatively short and broad. Apical ventrite lacks distinct knob (Fig. 67).

Legs: Pro- and mesotarsus enlarged, somewhat extended and provided with distinct suckers.

Male genitalia: Penis almost straight, voluminous with twisted, apical extension (Figs 259–260).

Female: Rarely elytron laterally, between middle and apex, with a distinct lateral expansion (Fig. 8). Apical ventrite (Fig. 68). Pro- and mesotarsus slender.

##### Distribution.

Sudan, Ethiopia, Uganda, Kenya, Tanzania, Zaire, Zambia, Malawi, Mozambique, Zimbabwe, Swaziland, South Africa (Fig. 560). Additional country record is Lesotho (Omer-Cooper 1965)

##### Collecting circumstances.

Some biological information is available in Omer-Cooper (1958b); in most cases sampled in various kinds of streams but also in water holes. In Ethiopia sampled at high altitudes (between 5000-7500 feet) under the name *Laccophilus pilitarsis* (Omer-Cooper 1931) and as *Laccophilus pellucidus* at altitudes of 1500-2300 m (Nilsson and Persson 1993). See also Pederzani (1988).

### Species group 10 (*Laccophilus adspersus* group)

**Diagnosis.** Small to large species; body length 2.9–5.1 mm, width 1.6–2.9 mm.

Shape of body oval to oblong, dorsoventrally flattened (Figs 419–420). Dorsal colour pattern variable; sometimes unicoloured without any pattern, sometimes with vague, dense irroration which in part can be reduced and finally species with distinct, often patchy colour pattern (Figs 426, 438, 443, 445). Dorsal microsculpture double; divided into two size-classes i.e. large and small meshes. Sometimes either kind can be reduced and at least in part indistinct or absent.

Prosternal process slender, posteriorly extended, apically pointed. (Prosternal process broken in unique specimen of *Laccophilus amicus* and state accordingly unknown). Apical ventrites with posterior end modified; excavated on each side of midline; at midline posteriorly extended (Figs 69–70), in males apical ventrite provided with an asymmetric knob on one side (Figs 71, 73). Stridulatory apparatus lacking. Metacoxal process not extended posteriorly (Fig. 6).

Paramere generally slightly enlarged, quite simple and exhibits generally no or moderate modifications (Fig. 268). Penis rather slender, always curved or angled; almost all species with a distinct apex which is variously modified (hooked, bifid, curved etc.) (Figs 263, 270, 291, 299).

**Species composition and distribution.** 24 species are recognized in this species group, which most probably is artificial and can be further split. No synapomorphous character for the group detected.

**Comments.** *Laccophilus amicus*, of which only female is known is characterized by small body (length 3.3–3.4 mm, width 1.8) and by peculiar elytral colour pattern (Fig. 442). *Laccophilus amicus* is most probably closely related to *Laccophilus bellus*, on the basis of external similarity.

To observe that present key is tentative and in determination both external features and male genitalia should be checked.

#### Key to species (males)

**Table:** 

1	Elytra unicoloured, dark to pale ferrugineous; lack distinct colour pattern (Figs 444–446); small species (length 2.9–3.3 mm)	**2**
–	Elytra with variable colour pattern; generally larger species	**3**
2	Elytra pale ferrugineous to ferrugineous (Fig. 444)	***Laccophilus septicola*** (p. 145)
–	Elytra blackish ferrugineous (Fig. 445)	***Laccophilus pullatus*** (p. 147)
3	Elytra blackish ferrugineous with small, pale ferrugineous spots (Fig. 446); penis as in Figs 293–294; small species (length 2.9 mm)	***Laccophilus luteosignatus*** (p. 148)
–	Elytral colour pattern different; penis shape different; larger species (length min. 3.1 mm)	**4**
4	Penis strongly angled in lateral aspect (about 75–90°) (Figs 295, 301); large species, length 4.3–4.9 mm	**5**
–	Penis generally straighter (angle more than 100°); many species less than 4 mm in length	**6**
5	Elytral colour pattern distinct; with two transverse, pale, areas where irrorations reduced (Fig. 452); penis as in Fig. 301	***Laccophilus guignoti*** (p. 157)
–	Elytral colour pattern vague, indistinct, no transverse pale areas (Fig. 447); penis as in Fig. 295	***Laccophilus benoiti*** (p. 150)
6	Penis, lateral aspect, with small, sharp knob located close to inner curvature (Fig. 266)	**7**
–	Penis lacks corresponding knob (Fig. 276)	**9**
	[Comment: note that some specimens of *Laccophilus adspersus* have a resembling knob on penis, which is less sharp and pronounced]
7	Colour pattern evenly distinct in basal half of elytra (Fig. 424); penis as in Fig. 268	***Laccophilus nodieri*** (p. 113)
–	Scutellar region with reduced, indistinct elytral colour pattern (dark ferrugineous irrorations vague) (Fig. 422); penis different	**8**
8	Penis, lateral aspect, with apex outline rounded (Fig. 266)	***Laccophilus modestus*** (p. 107)
–	Penis, lateral aspect, with apex outline angled (Fig. 267)	***Laccophilus cryptos*** (p. 111)
9	Penis apex distinct, almost bifid (Fig. 299); large species (4.6–5.1 mm)	***Laccophilus vermiculosus*** (p. 154)
–	Penis apex different; generally smaller species	**10**
10	Penis apex simple, exhibits no modifications (Figs 297–298) (Madagascar)	***Laccophilus addendus*** (p. 151)
–	Penis apex variously modified (Mainland Africa, Madagascar)	**11**
11	Penis robust, apex large and outline evenly curved (Fig. 270); elytral colour pattern variable Figs 426–429	(sp. complex?) ***Laccophilus remex*** (p. 118)
–	Penis less robust to quite delicate, apex smaller, often differently shaped	**12**
12	Penis, lateral aspect, external outline angled (curvature smooth) (Figs 275–276)	**13**
-	Penis, lateral aspect, external outline almost evenly curved (Figs 277, 283)	**15**
13	Penis apex large, distinct (Fig. 275)	***Laccophilus turbatus*** (p. 123)
–	Penis apex, small, hardly discernible (Fig. 276)	**14**
14	Elytral irrorations distinct (Fig. 433); penis apex broad (Fig. 276)	***Laccophilus pallescens*** (p. 125)
–	Elytral irrorations diffuse (Fig. 435); penis apex narrow (Fig. 278)	***Laccophilus mediocris*** (p. 132)
15	Extreme penis apex projects forwards (Fig. 277); elytra anteriorly at suture with distinct, quite long, narrow pale area without irroration (Fig. 434)	***Laccophilus trilineola*** (p. 130)
–	Extreme penis apex curved (Fig. 286); elytral colour pattern different	**16**
16	Penis, lateral aspect, inner outline with medial expansion (Fig. 285)	***Laccophilus enigmaticus*** (p. 138)
–	Penis, lateral aspect, inner outline lacks medial expansion (Fig. 283)	**17**
17	Penis apex broad, truncate, and turned upwards, appears in lateral aspect, narrow (Fig. 280)	**18**
–	Penis apex broad, truncate but not turned upwards, appears in lateral aspect broad (Fig. 269), or shape of penis apex different (Fig. 265)	**19**
18	Elytral colour pattern distinct (Figs 438–439; penis as in Fig. 283	***Laccophilus saegeri*** (p. 136)
–	Elytral colour pattern diffuse (Fig. 436); penis as in Fig. 280	***Laccophilus epinephes*** (p. 134)
19	Penis apex broad, hooked (Fig. 269); elytral colour pattern generally distinct (Fig. 425)	**20**
–	Penis apex different (e.g. Fig. 265); elytral colour pattern rather diffuse (irrorations less pronounced) (e.g. Fig. 419)	**21**
20	Large species (body length 4.2–4.6 mm); male genitalia (Fig. 269)	***Laccophilus flaveolus*** (p. 115)
–	Small species (body length 3.1–3.3 mm); male genitalia (Fig. 288)	***Laccophilus bellus*** (p. 144)
21	Penis, inner outline almost evenly curved from base to apex (Fig. 286)	***Laccophilus restrictus*** (p. 140)
–	Penis, inner outline angled, not evenly curved from base to apex (Fig. 265)	**22**
22	Penis apex (Fig. 265); elytral colour pattern (Fig. 420) (Madagascar)	***Laccophilus olsoufieffi*** (p. 105)
–	Penis apex variable (Figs 261–264); elytral colour pattern (Fig. 419) (Mainland Africa)	***Laccophilus adspersus*** (p. 97)

#### Laccophilus adspersus

Taxon classificationAnimaliaColeopteraDytiscidae

Boheman, 1848

Figs 69–70 , 261–264 , 419 , 544

Laccophilus adspersus Boheman 1848: 246 (original description, faunistics); Sharp 1882: 287, 819 (description, faunistics); v. d. Branden 1885: 20 (catalogue, faunistics); Régimbart 1894: 237 (description, faunistics); Régimbart 1895: 135 (discussion, description, faunistics); Régimbart 1905: 208 (faunistics); Régimbart 1906: 248 (faunistics); Régimbart 1908: 5 (faunistics); Zimmermann 1920a:16 (catalogue, faunistics); Peschet 1925: 31 (faunistics); Omer-Cooper 1931: 756 (description, biology, faunistics); Gschwendtner 1932a: 12 (faunistics); Gschwendtner 1935a: 15 (faunistics); Guignot 1946c: 270, 273, 274, 276, 278, 312 (description, faunistics, discussion); Guignot 1953c: 145 (faunistics); Omer-Cooper 1956: 21 (faunistics, biology); Omer-Cooper 1957: 16, 18, 19, 90 (description, discussion, faunistics); Omer-Cooper 1958a: 59 (faunistics); Omer-Cooper 1958b: 37, 47 (discussion, description, faunistics); Guignot 1959a: 562, 566 (description, discussion, faunistics); Omer-Cooper 1962: 295 (faunistics); Bertrand 1963: 411 (discussion, faunistics); Omer-Cooper 1965: 77, 85 (description, faunistics); Bertrand and Legros 1967: 862 (faunistics); Omer-Cooper 1970: 290, 291, 292, 293, 294 (description, discussion, faunistics); Medler 1980: 155 (faunistics, list.); Bilardo 1982b: 251 (faunistics); Bilardo and Rocchi 1987: 104: (faunistics, biology); Pederzani 1988: 107 (faunistics, biology); Bilardo and Rocchi 1990: 162, 177 (faunistics, biology); Curtis 1991: 186 (faunistics); Nilsson and Persson 1993: 80 (discussion); Rocchi 2000: 24 (faunistics); Nilsson 2001: 240 (catalogue, faunistics); Bilardo and Rocchi 2002: 173 (list, faunistics); Pederzani and Reintjes 2002: 38 (faunistics); Reintjes 2004: 66 (faunistics); van Vondel 2005: 130 (faunistics, biology); Nilsson 2015: 208 (catalogue, faunistics).
Laccophilus livens Severin 1892: 472 (nomen nudum, discussion); Régimbart 1895: 135 (original description, faunistics); Zimmermann 1920a:21 (catalogue, faunistics); Zimmermann 1926: 24 (faunistics, discussion); Gschwendtner 1930: 88 (faunistics); Gschwendtner 1931: 180 (faunistics); Gschwendtner 1935a: 15: (faunistics); Gschwendtner 1938a: 5 (faunistics); Gschwendtner 1938b: 337 (faunistics); Guignot 1943: 99 (faunistics); Guignot 1946c: 269, 273, 276, 277, 312 (description, faunistics, discussion); Guignot 1953b: 234 (faunistics); Legros 1954: 268 (faunistics); Omer-Cooper 1957: 16 (synonym *Laccophilus adspersus* Boh.); Legros 1958: 211 (faunistics); Guignot 1959a: 558, 562, 565, 566 (description, discussion, faunistics); Guignot 1959d: 161, 162 (discussion, faunistics); Omer-Cooper 1965: 85 (list, synonymy); Omer-Cooper 1970a: 290, 291, 292, 29 (list, synonymy); Hernando 1990: 177, 178: (discussion, description); Nilsson 2001: 240 (catalogue faunistics, list, synonymy); Pederzani and Reintjes 2002: 40 (faunistics); Nilsson 2015: 208 (catalogue, faunistics). **Confirmed synonym.**
Laccophilus vitshumbii Guignot 1959d: 161 (original description, faunistics); Guignot 1961b: 238 (faunistics, discussion); Omer-Cooper 1970: 290, 293, 294 (discussion, description); Nilsson and Persson 1993: 58, 80, 94 (faunistics, biology); Nilsson 2001: 253 (catalogue, faunistics); Pederzani and Reintjes 2002: 40 (faunistics); Nilsson 2015: 219 (catalogue, faunistics). **New synonym.**
Laccophilus adspersus nigeriensis Omer-Cooper 1970: 291, 292, 293 (original description, faunistics); Medler 1980: 155 (catalogue, faunistics); Nilsson 2001: 240 (catalogue, faunistics); Pederzani and Reintjes 2002: 38 (faunistics); Nilsson 2015: 208 (catalogue, faunistics). **New synonym.**
Laccophilus adspersus sudanensis Omer-Cooper 1970: 292, 293 (original description, faunistics); Nilsson 2001: 240 (catalogue, faunistics); Pederzani and Reintjes 2002: 38 (faunistics); Nilsson 2015: 208 (catalogue, faunistics). **New synonym.**

##### Type localities.

*Laccophilus adspersus*: South Africa: Caffraria interior.

*Laccophilus livens*: Zaire: Boma.

*Laccophilus vitshumbii*: Zaire: Lake Edouard, Vitshumbi.

*Laccophilus adsperus nigeriensis*: Nigeria: Jos.

*Laccophilus adspersus sudanensis*: Sudan: S of Rumbek near Wulu.

##### Type material studied

(56 exs.). Lectotype (by present designation): male: “Caffraria. / J. Wahlb. / Paratype / 3465 E91 / Naturhistoriska Riksmuseet Stockholm Loan no 1261/05” (NHRS). [Commments: no holotype was chosen in original description and neither has any lectotype thus far been designated. One male specimen is provided with Typus label but unfortunately its dissected genitalia are missing. We have dissected another male belonging to the type series (labelled paratype). Male genitalia is preserved in this specimen and we have chosen it therefore to be lectotype of *Laccophilus adspersus* Boheman.] – Paralectotypes: Same data as lectotype, but labelled as “Typus / Allotypus / Paratypus” (4 exs. NHRS).

*Laccophilus livens*: Lectotype (by present designation) male: Label with “male” symbol / Matadi Congo / Type / *Laccophilus livens* Rég.” (MNHN). – Paralectotypes: “Banana-Boma M. Tschoffen 91. Det. Régimb. 91 / 11174 / Régimbart det. 1891: *Laccophilus livens* Rég. / Ex. Typis” (7 exs. IRSNB). [Comment: one additional paralectotype in IRSNB with same data belongs to another species (= *Laccophilus modestus* Régb.)]; “Paratype / Banana-Boma M. Tschoffen 91. Det. Régimb. 91 / 11174 / Type *Laccophilus livens* Régt.“Type” (1 ex. BMNH); “Cotype / Congo / *Laccophilus livens* Régt.“Co-type”” (1 ex. BMNH); “Severin Banana Africa / Banana Boma M. Tshoffen 91 Dét. Régimb. / *Laccophilus livens* Rég. Type / Type” (1 ex. RMNH); same, but “Afr. occ.” (2 exs. RMNH); “Matadi M. Tshoffen / *Laccophilus livens* Rég. Types / SAM Type Acc. no. 839” (2 exs. SAMC); same data and “Cotype” (1 ex. IRSNB).

*Laccophilus vitshumbii*: Holotype: male: “Lac Édouard, Vitshumbi, 3043, mare I à Juissiaea M.T. 13-14.VI. 1953” (not studied; in IRSNB according to original description). – Paratypes: studied: “Congo Belge Lac. Edouard Vitshumbi, mare I + Juisseau MT 13-14.VI. 1953, 3043 / male symbol / Paratype” (2 exs. MNHN); “Congo Belge, Lac Edouaurd, Ishango d’Semliki (sur Graminées) 5.II. 1954, 3118a / Paratype” (1 ex. MNHN); “Male symbol / Congo Belge, Lac Edouaurd, Vitshumbi, mare II + *Lemna* 14.VI. 1953, 3042 / Paratype / *Laccophilus vitshumbii”* (1 ex. MNHN).

*Laccophilus adspersus nigeriensis*: Holotype: male: “Type, male symbol / *Laccophilus adspersus nigeriensis* O-C. / Nigeria, Reservoir, stream, Jos 10.IV. 1963 J.O-C.” (AMGS). – Paratypes: “*Laccophilus* spp. / *adspersus* ? / Nigeria, stream & reservoir Jos 10.IV. 1963 J. O-C.” (4 exs. AMGS); “Nigeria, stream near Zaria 4.IV. 1963 J. O-C.” (4 exs. AMGS); “Nigeria (15A), stream Kaduna-Kontagora rd. 3.IV. 1963 J. O-C.” (1 ex. AMGS); “Nigeria, river, Jos-Bauchi rd. 9.IV. 1963 J. O-C.” (3 exs. AMGS); “Nigeria, river between Jos & Bauchi 9.IV. 1963 J. O-C.” (1 ex. AMGS); “Nigeria 27, detritus pond 45 miles from Jos on Bauchi rd 9.IV. 1963 J. O-C.” (14 exs. AMGS); *“adspersus* / Nigeria, stream nr Bukuru 11.IV. 1963 J. O-C:” (1 ex. AMGS).

*Laccophilus adspersus sudanensis*: Holotype: male: *“Laccophilus adspersus* subsp. *sudanensis* O-C. / Type / S. Sudan, rain ponds S. of Rumbek nr. Wulu 19.VII. 1954” (AMGS).

##### Additional material studied

(428 exs.). **Sudan**: “Rain ponds S of Rumbek nr Wulu 19.VII. 1954” (1 ex. AMGS); “Upper Nile Malakal 5-20.1. 1963 Linnavuori” (1 ex. MZH); “Equatoria, Mundri-Lalyo 25-26.2. 1963 Linnavuori” (1 ex. MZH); “Equatoria, Mwolo-Mundri 24.2. 1963 Linnavuori” (2 exs. MZH); “Equatoria, Lalyo-Juba 26-27.2. 1963 Linnavuori” (6 exs. MZH); “Equatoria, Loka Forest 8-10.4. 1963 Linnavuori” (1 ex. MZH); “Equatoria, Nzara 22.4. 1986 Wewalka / *Laccophilus adspersus sudanensis* O-C. det. Wewalka” (6 exs. CGW). – **Ethiopia**: “Water hole N Makki River, 6000 ft., 28.9. 1926 J. Omer-Cooper” (5 exs. BMNH); “Stream W of Zaquála 6000 ft., 27.10. 1926 J. Omer-Cooper” (1 ex. BMNH, 1 ex. MZH); “Hora Harsadi, Addas 1.12. 1926, 7000 ft, JOC” (1 ex. AMGS); “Hora Horeso 7000 ft., 1.12. 1926 JOC” (1 ex. AMGS). – **Ivory Coast**: “Comoé NP, N8,5° W3,5° Reintjes / 20.2. 1999 temporary creek” (1 ex. NMW). – **Nigeria**: “Kontagora pools in dry stream bed 3.IV. 1963 JOC.” (2 exs. AMGS); “Stream nr Zaria 4.IV. 1963 JOC.” (5 exs. AMGS); “Stream escarpment Jos-Wambe rd 13.IV. 1963 JOC” (1 ex. AMGS); “River between Jos-Bauchi 9.IV. 1963 JOC.” (4 exs. AMGS); “A stream nr Bakura 11.IV. 1963 JOC.” (1 ex. AMGS); “Stream Kaduna-Zaria rd 4.IV. 1963 JOC (10 exs. AMGS); “Kaduna-Zaria rd 4.IV. 1963 JOC” (2 exs. AMGS); “Stream & reservoir Jos 10.IV. 1963 JOC” (5 exs. AMGS); “Stream 86 miles from Makureli on Jos road 25.IV. 1963” (5 exs. AMGS); “Pools in dry stream bed Kontagora 5.IV. 1963 JOC” (1 ex. AMGS); “Pools, bridge over trib. of R. Niger, rd Kaduna-Kontagora” (1 ex AMGS); “Stream Kaduna-Kontagora rd 3.IV. 1963 JOC” (1 ex. AMGS); “Stream crossing Kaduna rd Zaria 8.IV. 1963 JOC” (1 ex. AMGS); “R. Kaduna 4.5 miles from Jos 13.IV. 1963 JOC.” (3 exs. AMGS). – **Cameroon**: “20 km NW Bangante Forest, savannah at river, at light 15.1. 1978/Gärdenfors, Hall & Samuelsson leg.” (1 ex. MZLU); “Maroua 26.8. 1973” (1 ex. NHMB). – **Central African Republic**: “Bozo 21.5. 1981/Degallier” (1 ex. MZH); same but “12. 1981” (1 ex. NHMB); same but “8. 1981” (1 ex. NHMB). – **Zaire**: “Longitshimo River, N-7.163, E20.880, 17.8. 2007 Graham” (1 ex. AMGS); “Lulimbi (Rutshuru) 1976 Lejeune” (2 exs. MRAC); “Parc National Garamba 29.9. 1951 De Saeger H. 2494” (2 exs. NHMB) “Parc National Garamba 28.8. 1952 De Saeger H. 3987” (1 ex. MRAC); “Parc National Garamba 27.6. 1952 De Saeger H. 3717” (1 ex. MRAC); “Parc National Garamba 5.5. 1952 De Saeger H. 3421” (1 ex. MRAC); “Parc National Garamba 19.3. 1952 De Saeger H. 3199” (1 ex. MRAC); “Parc National Garamba 6.2. 1952 De Saeger H. 3095” (1 ex. MRAC); “Parc National Garamba 3.4. 1952 De Saeger H. 3278” (6 exs. MRAC, 3 exx. MZH); “Parc National Garamba 2.4. 1952 De Saeger H. 3272” (2 exs. MRAC); “Parc National Garamba 31.8. 1952 De Saeger H. 3870” (2 exs. MRAC); “Parc National Garamba 1.8. 1952 De Saeger H. 3871” (1 ex. MRAC); “Parc National Garamba 4.4. 1952 De Saeger H. 3290” (1 ex. MRAC); “Parc National Garamba 1.9. 1952 De Saeger H. 4035” (5 exs. MRAC); “Katanga, Mwadingusha 21.5. 1965 Verheyen leg.” (1 ex, MRAC). – **Uganda**: “Kampala Hoima Rd 16.4. 1929 G.L.R. Hancock” (1 ex. AMGS); “Kampala 30.1. 1927 H. Hargreaves” (2 exs. AMGS). – **Kenya**: “Mombasa 25 km Nord palude de Kikalibala presso strada 14.7. 1968 Pederzani” (1 ex. AMGS); “Mombasa 30 km Nord Kikalibala swamp 14.7. 1968 Pederzani” (1 ex. AMGS); “Lambwe Valley, on light 11.6. 1974 van Etten” (2 exs. RMNH); “Manjewa R. Mariakani Kilifi / Kwale district 16.4. 1976 Holmen 4281” (1 ex. MZH); “Pond NE of Mariakani, Kilifi Distr. 16.9. 1976 Holmen 6076” (1 ex. ZMUC); Momb. Kilifi district 17.9. 1976 Holmen EF 8057” (1 ex. ZMUC); “Dam N of Gotani, Kilifi District 15.9. 1976 Holmen 5987” (1 ex. ZMUC); “Arabuko Sokoke Forest (30 km S Malindi) 8-24.6. 1998 Bartolozzi & Sforzi leg. alla luce” (1 ex. CSR); “Arabuko Sokoke Forest Res., Kilifi Distr., 20 km S Malindi/21.5.-7.6. 1994 Bartolozzi et al” (2 exs. CSR); “Thika 7.12. 1989 Jäch leg.” (1 ex. NMW); “Nairobi 3.11. 1967 / Reichart leg.” (1 ex. USNM); “Rabur 20.11. 1967 / Reichart leg.” (1 ex. USNM); “Kiserian 26.10. 1967/Reichart leg.” (1 ex. USNM, 1 ex. MZH); same data but “30.10. 1967” (1 ex. USNM); “Kibwezi Scheffler” (1 ex. ZMHB); “Meru Distr., Mourglia/Matiri (Mituguu) 8.11. 1983 800 m” (3 exs. NHMB); “Wa Kikuyu Bassin de l’Athi, Alluaud N. 1908” (1 ex. NHMB); “Br. O. A. Fort Hall” (1 ex. NHMB). – **Tanzania**: “Petukiza, ponds Tanga district 23.9. 1976 Holmen 1772” (1 ex. ZMUC); “Lukoka Pond, Tanga District 22.9.1976 Holmen 7230” (1 ex. ZMUC); “Tanganyika Ukerewe VII. 1933” (3 exs. OLML); “Kilimandjaro Sjöstedt 1905-1906/Kibonoto 1000-1300 m/21 Sept.” (1 ex. NHMB, 6 exs. NHRS, 3 exs. ZMHB); “Wembäre Steppe 6. 1911” (1 ex. ZMHB); “Pr. Shinyanga 60 km E Kahama 22.12. 2006 1150 m Kudrna Jr. lgt.” (1 ex. CFP); “Pond in stream bed, 107 mi from Dodoma 15.2. 1954” (1 ex. AMGS). – **Angola**: “Ca 10 mls W of Cainde, c. 3500 ft 15.4. 1954 / stagnant water hole, nitellid algae and muddy silt” (10 exs. BMNH, 2 exx. MZH); “Namakunda 6. 1948 16.15E. 18.50S C. Koch leg.”(1 ex. BMNH). – **Zambia**: “Kasempa env. 16-18.11. 2006, Z. Jindra leg.” (1 ex. NMPC); “Chinganganka 17.3. 1993 lux 15°53’ / 28°11'E, lux, hills, Uhlig leg.” (1 ex. ZMHB); “Kafue NP, Chunga Camp 26-29.3. 1993, 15°02'S / 26°00'E, lux, Göllner leg.” (1 ex. ZMHB); “Kafue NP, Chunga Camp 27.3. 1993, 15°02'35"S/26°00'09"E, lux, Uhlig leg.” (2 exs. ZMHB); “Africa Copperbelt Pr. Muekera 23.1. 1982 Selander / rain pond” (1 ex. MZH). – **Malawi**: “R Mtiti N of Lilongwe 1.X. 1948 JOC.” (1 ex. AMGS); “R Diedma Lilongwe rd. 30.IX. 1948 JOC.” (2 exs. AMGS); “River nr Dedza 28.IX. 1948 JOC.” (1 ex. AMGS); “Stream 20 miles from Dedza on lower Lilongwe rd 30.IX. 1948” (4 exs. AMGS); “Dedza dam on lower Lilongwe rd 29.9. 1948” (2 exs. AMGS); “Dedza env. 6-13.1. 2002 Bezdek leg.” (1 ex. NMPC); “Balaka env. 19-20.7. 2001 J. Bezdek leg.” (3 exs. NMPC, 1 ex. MZH); same but “5-6.1. 2002” (4 exs. NMPC, 1 ex. MZH); “Balaka env., 19.12. 2002 180 km SE Lilongwe Kantner” (1 ex. NHMB); “Stream (?) N of R. Mtiti X. 1948 / Paratype / *Laccophilus simulator* sp. n. det. J. O. Cooper” (1 ex. IRSNB; paratype *Laccophilus simulator* O-C.). – **Zimbabwe**: “Wankie Game Res. JOC. Waterholes / *Laccophilus adspersus* Boh. Det. JOC. (2 exs. AMGS); “Wankie Game Res. 5 Sept. 1948 JOC. Ponds at Robins Restcamp / *Laccophilus adspersus* Boh. Det. JOC.” (2 exs. AMGS); “Wankie Game Res. September 1948 JOC., waterhole / *Laccophilus adspersus* Boh. Det. JOC.” (4 exs. AMGS); “Wankie Game Res. 2.IX. 1948 JOC. / *Laccophilus adspersus* Boh. Det. JOC.” (2 exs. AMGS); ”5 mi SE Wankie 7.4. 1968 Spangler” (2 exs. USNM); “Gokwe Sengwa W.LaccophilusR.I. 28.12. 1982 -4.1. 1983 Bell / blacklight” (1 ex. NHMB); “Shangani R. 13.IX. 1948 J. O-C. / *Laccophilus adspersus* Boh. Det. JOC.” (2 exs. AMGS); “Stream Halfway hotel Gatooma Salisbury 14.IX. 1948 JOC.” (3 exs. AMGS); “Stream Halfway hotel Gatooma-Salisbury 14.IX. 1948 / *Laccophilus adspersus* Boh. Det. JOC.” (1 ex. AMGS); “Pool Lundi 22.N. 1948 J.O-C.” (5 exs. AMGS); “Sinkukwe 30 Dec. 1948 JOC.” (21 exs. AMGS, 3 exs. USNM, 1 ex. MZH); “Salisbury Mashonaland 1893 Marshall” (2 exs. SAM); “Nuanetsi River, Majinji Pan 4-5. 1961” (8 exs. BMNH, 1 ex. MZH); “Matopos NP 28.11-1.12. 1993, 20°33'S/28°30'E lux Uhlig leg.” (1 exs. ZMHB, 1 ex. MZH); “Gwai River 3.4. 1968 Spangler” (1 ex. USNM); “Ngezi N.P. env., 1.12. 1998 Kantner” (1 ex. NHMB); “Birkennough Bridge 24.1. 1998 Kantner” (1 ex. NHMB); “Mushandike Sanct. 10.12.1998 Kantner” (2 exs. NHMB, 1 ex. MZH); “Kariba env. 20.12. 1998 Kantner” (1 ex. NHMB); “Pond 26 mi. from Fort Victoria, Beit Bridge Rd.13.11.1948 J.O-C.” (2 exs. AMGS). – **Namibia**: “Omapapurawe Guard Post, 200 m from campsite, Cunene R., N-17.218, E13.645, pool, 15.11. 1997 Bethune et al. (1 ex. AMGS); “Kaokoveld, Sanitatas abt 85 mi WSW Ohopoho 14-16.6. 1951 / *Laccophilus adspersus* Boh. det. J. Omer-Cooper” (4 exs. MZLU); “Kaokoveld, Kowares 90 mi SE Ohopoho 3.6. 1951” (1 ex. MZLU). – **Botswana**: “Metsimaklaba 7-12.3. 1930 / *Laccophilus livens* Régb. det. Gschwendtner” (1 ex. OLML, 6 exs. TMSA); “N’Kate Makarikari 6-23.8. 1930 / *Laccophilus adspersus* Boh. det. Gschwendtner” (1 ex. TMSA); “Tsotsorogo Pan 17.6.-9.7. 30 / *Laccophilus livens* Régimbart det. Gschwendtner” (1 ex. OLML, 1 ex. TMSA); “Kasane 25-28.7. 1930 / *Laccophilus addendus* Shp det. Gschwendtner” (2 exs. TMSA). – **South Africa**: “Transvaal Sand R. 16.XII. 1953” (1 ex. AMGS); “Tshakoma Zpbg N. 1931 van Son / *Laccophilus adspersus* Boh. det. Omer-Cooper” (1 ex. TMSA); same but ”det. Gschwendtner” (1 ex. TMSA); “Valdesia Zpbg N. 1931 van Son / *Laccophilus adspersus* Boh. det. Gschwendtner” (1 ex. TMSA); “Trsvl Koring Spruit / Waterberg Dist. 20.8. 1948 J.O.C. / *Laccophilus livens* Rég. det. J. Balfour-Browne” (1 ex. TMSA); “Transvaal Kruger Park 1.VII. 1960” (2 exs. AMGS); “Kruger N.P. Skukuza, 12 km S, 25.04S, 31.37E / 6.3. 1996 UV light, Endrödy-Younga” (5 exs. TMSA, 1 ex. MZH); “Kruger N.P. Skukuza Res. camp, 24.59S, 31.36E / 25.2. 1995 UV-light & trap Endrödy-Younga” (4 exs. TMSA; habitus in Fig. 419); “Kruger N.P. Levuvu River, 22.27E, 31.10E / 12.2. 1994 shorewashing Endrödy-Younga” (2 exs. TMSA); “Kruger N.P. Letaba Riv. bel. dam 23.46S-31.30E / 1.3. 1995 shorewashing Endrödy-Younga” (2 exs. TMSA); “Trsvl, K.N.P., Pan 24 km S Satara Camp, N-24.610, E31.800, 18.6. 1960” (1 ex. AMGS); “Transvaal R. Nyl at Num Num 23.VIII 1948” (2 exs. AMGS); “Transvaal R. Nyl at Num Num 23.VIII 1948 / *Laccophilus livens* Reg. J. Balfour-Browne det.” (1 ex. AMGS); “RSA N. Prov. near Nylstroom 20.11. 2004 Werner & Smrz” (1 ex. NHRS); “Transvaal *Laccophilus adspersus* Boh. Det. J. Omer-Cooper” (2 exs. AMGS); “Trsvl Naboomspruit Torino Ranche 24.37S-28.38E / 15.1. 1989 UV light, vlei edge Endrödy-Younga” (3 exs. TMSA); “Trsvl Pretoria distr. Roodeplaat / 25-26.10. 1960 UV-light Neubecker” (2 exs. TMSA); “Pretoria 6.11. 1959 Janse / *Laccophilus adspersus* det. Gschwendter” (1 ex, TMSA); “Trsvl 5 mi W Warmbad 24-25.2. 1968 Spangler” (16 exs. USNM, 3 exs. MZH); “Trsvl Bundu Inn 25.28S-28.55E/24.3. 1974 at merc. vap. light Endrödy-Younga” (1 ex. TMSA); “Trsvl Swartspruit Mouth, Hartebeespoortdm. N-25.750, E27.900, 11.2. 1972 Reavell” (1 ex. AMGS); “Trsvl, stream in Magaliesberg Mts., mountain stream 11.9. 1972 Reavell” (1 ex. AMGS); “Plat R. 6-18.4. 05/Waterberg Distr. Swierstra” (2 exs. TMSA); “Frere Natal 1893 Marshall” (3 exs. SAMC); “Nat. -Drakensbg, Cathedral Peak, 28.57S, 29.12E/14.3. 1976 UV light station Endrödy-Younga” (2 exs. TMSA); “Kw. Natal Port Shepstone 20 km W 2.2. 2000 Halada” (2 exs. NMW); “Kw. Natal, McLeod’s Farm nr Dargle, Umgeni R., 4.2. 1989 Reavell” (1 ex. AMGS); “Kw. Natal Lundy’s Hill N-29.741, E29.872, marginal vegetation, stones, 30.4. 1996 de Moor et al.” (1 ex. AMGS); “Kw. Natal Gravesend estate N-30.170, E30.736, 12.10. 1996 Dickens et al.” (2 exs. AMGS); “Kw. Natal, Umlazi R., Tali Area, N-29.800, E30.520, 11.2. 1954 Oliff” (1 ex. AMGS); “Kw. Natal, Felixton, main drain from mill, N-28.840, E31.880, 3.7. 1962” (1 ex. AMGS); “Kw. Natal, Pond btw. Unizul and Mtunzini N-28.930, E31.750, marginal vegetation 20.9. 1995 Reavell” (1 ex. AMGS); “Kw. Natal Volkrust Road Bridge, Ncandu R., N-27.750, E29.930, 4.12. 1973 Metz” (1 ex. AMGS); “Kw. Natal, Below Newcastle Sewage Works, Ngagane R. N-27.720, E30.020, 19.6. 1994 Metz” (1 ex. AMGS); “Kw. Natal, Klip R. Stn. 3, 11.9. 1975 Sibbald & Brown” (1 ex. AMGS); “Kw. Natal, Ngogo R,. N-28.21.23, E29.43.25., 3.4. 1975 Metz” (2 exs. AMGS); “Kw. Natal, Izotsha R. Inland S Coast Rd N-30.780, E30.400, 5.6. 1972 Chutter” (1 ex. AMGS); “Kw. Natal, Little Amanzimtoti R, N-30.060, E30.820, 15.6. 1964 Pretorius” (1 ex. AMGS); “Natal Ladysmith 1000 m 29.12. 1993 Wewalka / *Laccophilus adspersus* Boh. det. Wewalka 1994” (3 exs. CGW); “Natal roadside puddles ca 2 km S Mbazwana to Hluhluwe nr Sodwana 5.3. 1997 Turner” (1 ex. NHMB); “Gauteng Tswaing 25.24S, 28.06E / 16.2. 2003 light trap” (1 ex. TMSA, 1 ex. MZH); “ECPr., close to Dwesa Nature Reserve, vegetation rich pond S32°17.027, E28°47.506, alt. 188 m 23.1. 2005 Bergsten” (1 ex. NHRS); “ECPr., close to Dwesa Nature Reserve, muddy pond with vegetation edges S32°18.582, E28°49.002, alt. 76 m 24-25.1. 2005 Bergsten” (1 ex. NHRS); “ECPr. Lusikisiki 19.III. 1956 JOC.” (1 ex. AMGS); “ECPr. Komgha quarry pond 20.III. 1955” (1 ex. AMGS); “ECPr. Quanbu 2.V. 1956” (1 ex. AMGS); “ECPr. St Johns 10.II. 1956” (3 exs. AMGS); “ECPr. Umzikulu 14.III. 1956 JOC.” (1 ex. AMGS); “Pirie Forest II. 1944 JOC.” (2 exs. AMGS); “ECPr. Mncotsho R., Trib. Buffalo Riv. N-32.54.43, E27.36.48, 18.2. 2002 de Moor” (5 exs. AMGS); ECpr. Nahoon R. at Witch Kranz, site NO, N-32.502, E27.392, 22.5. 2002, de Moor & Barber-James” (1 ex. AMGS); “ECpr., Dam on Rwantsa R, N-32.53.20, E27.37,55, 30.8. 2000 de Moor & Barber-James” (3 exs. AMGS); “ECPr. Rwantsa R. dam on Farm Mistley, N-32.53.20, E27.37.55, 10.11.2000 de Moor & Barber-James” (1 ex. AMSG); “ECPr. Rwantsa R. at Witchkranz, N-32.52.25, E27.38.34, 1.9. 2000 De Moor & Barber-James” (1 ex. AMGS); “ECPr. Rwantsa R. at Witchkranz, N-32.52.25, E27.38.34, 7.6. 2000 de Moor & Barber-James” (1 ex. AMGS); “ECPr., Rwantsa R. at Farm Wolsley, N-32.54.02, E27.51.95, 18.5. 2004 de Moor & Barber-James” (1 ex. AMGS); “ECPr., Rwantsa R. at Farm Wolsley, N-32.54.02, E27.41.51, 10.12. 2003 de Moor & Barber-James” (1 ex. AMGS); “ECPr., Rwantsa R. at Farm Wolsley, N-32.54.03, E27.41.51, 18.5. 2004 de Moor & Barber-James” (1 ex. AMGS); “ECPr. Rwantsa R. at Farm Sebastepol, N-32.53.00, E27.40.45, 7.5. 2000 De Moor & Barber-James” (3 exs. AMGS); “ECPr., Xolo R. trib. carrying sewage discharge, N-32.50.11, E27.37.49, 19.2. 2004 de Moor &Barber-James” (1 ex. AMGS); “ECPr., Mncotsho R., N-32.54.48, E27.36.52, 11.8.2003 de Moor & Barber-James” (1 ex. AMGS); “ECPr., Mncotsho R., N-32.54.48, E27.36.52, de Moor 15.5. 2001” (1 ex. AMGS); “ECPr., Mncotsho R., trib. Buffalo R., N-32.54.43, E27.36.48, 8.11. 2000 de Moor& Barber-James” (1 ex. AMGS); “ECPr., Mncotsho R., trib. Buffalo R., N-32.54.43, E27.36.48, 30.8. 2000 de Moor & Barber-James” (17 exs. AMGS); “ECPr., Mncotsho R., trib. Buffalo R., N-32.54.43, E27.36.48, 18.5. 2000 De Moor & Barber-James” (2 exs. AMGS); “ECPr., Nahoon R. at Witchkranz N-32,51,10, E27.39.08, 19.5. 2004, de Moor & Barber-James” (1 ex. AMGS); “ECPr., Nahoon R. at Witch Kranz N-32,50,28, E27.39.21, 22.5. 2002, De Moor & Barber-James” (1 ex. AMGS); “ECPr. Port, St Johns 15.2. 1956 / *Laccophilus adspersus* Boh. Det. JOC” (2 exs. AMGS); “ECPr. Mt Frere 8.V. 1956 JOC. / *Laccophilus adspersus* Boh. Det. JOC.” (1 ex. AMGS); “ECpr. 9.8. 1990 N-33.348, E26.678, Old Quarry Site, Manley Flats, dam, Baber-James & de Moor (2 exs. AMGS); “E Cape 9.3. 1997 Amatola Mts. 20 km NNE Aice 32°47'S 26°50'E Hess & Heckes” (1 ex. NMW); “EC., Hwy 352, 3 km S Tsomo, in river 22.5. 2005 Challet leg.” (1 ex. CGC); “North West 50 km S Kimberley Ritchie 12.1. 2000 Halada leg.” (1 ex. NMW). – **Swaziland**: “Little Usutu R nr. Bremersdorp 5.12. 1948” (1 ex. AMGS); “Bremersdorp 4.12. 1948 stream with muddy ponds JOC.” (2 exs. AMGS).

##### Comments on synonymy.

The type material of all five taxa involved have been studied and compared (except holotype of *Laccophilus vitshumbii*; not found). Minor variation in shape of penis apex and dorsal colour pattern can be recognized. There are, however, a series of transitional morphs between the extremes both regarding genitalia and external appearance. Distribution covers extensive areas of Africa south of Sahara which justifies occurrence of minor morphological variation within a species. No clear morphological evidence and distributional pattern are thus present which would merit separation of species or subspecies. Accordingly earlier synonymy of *Laccophilus adspersus* and *Laccophilus livens* is confirmed. Furthermore *Laccophilus vitshumbii*, *Laccophilus adspersus nigeriensis* and *Laccophilus adspersus sudanensis* are all considered new synonyms of *Laccophilus adsperus*. *Laccophilus adsperus* being the oldest available name is the valid name of the species.

##### Diagnosis.

Despite slight variation in shape of penis apex and elytra colour pattern in *Laccophilus adspersus* these features are still the best way of separation the species from other *Laccophilus* species. *Laccophilus adspersus* resembles most of *Laccophilus olsoufieffi*. Further study may show that they are also conspecific (see diagnosis of *Laccophilus olsoufieffi* on p. 106).

##### Description.

Body length 3.6–4.2 mm, width 1.9–2.2 mm. Body almost unicoloured pale ferrugineous to ferrugineous; elytra with slightly indistinct irrorations. At base irrorations slightly sparser and often slightly reduced. Some specimens exhibit a variable pale spot with reduced irrorations posteriorly on each elytron (Fig. 419).

Head: Pale ferrugineous. Slightly mat to rather shiny, finely microsculptured; reticulation indistinctly double. Large meshes contain 2–6 fine meshes. Almost impunctate. At eyes with some scattered, fine punctures.

Pronotum: Pale ferrugineous. Rather shiny, although finely microsculptured; reticulation double. Finer meshes sometimes indistinct and hardly discernible. When discernible large meshes contain 2–8 finer meshes. Almost impunctate; punctures indistinct and hardly visible. Scattered punctures may be discerned laterally and at anterior margin.

Elytra: Pale ferrugineous. With ferrugineous to dark ferrugineous, slightly obsolete irrorations. Sometimes each elytron posterior to middle with a pale spot where irrorations reduced (Fig. 419). Rather shiny, although densely microsculptured. Reticulation double; laterally and posteriorly double reticulation becomes indistinct. Large meshes contain generally 3–8 fine meshes. Fine meshes in part weakly developed and difficult to discern. Discal, dorsolateral and lateral rows of punctures, irregular, very fine and in part hardly visible. Pre-apical furrow fine, sparsely pubescent.

Ventral aspect: Pale ferrugineous to ferrugineous. Almost impunctate. Rather shiny although very finely microsculptured. Metacoxal plates with in part reduced transverse furrows. Abdomen basally with sparse, somewhat curved striae. Apex of prosternal process slender and pointed. Apical ventrite with a small knob on one side (Fig. 69).

Legs: Pro- and mesotarsus slender, somewhat extended, provided with suckers.

Male genitalia: Note variation in shape of penis apex; extreme apex exhibits a gradual change from pointing straight forwards to, being somewhat curved and blunt (Figs 261–264).

Female: Externally as male but apical ventrite lacks asymmetric knob (Fig. 70). Additionally pro- and mesotarsus slender.

##### Distribution.

Sudan, Ethiopia, Ivory Coast, Nigeria, Cameroon, Central African Republic, Zaire, Uganda, Kenya, Tanzania, Angola, Zambia, Malawi, Zimbabwe, Namibia, Botswana, South Africa, Swaziland (Fig. 544). Confusion in species delimitation has been common during the years and accordingly only personally verified records are accepted in the map.

##### Collecting circumstances.

Insufficiently documented. Van Vondel (2005) reports the species to be sampled in a pond surrounded by tree, bottom covered with plant remains and water lily growths. Furthermore collected from pools with water lily and *Pistia stratiotes* and in stagnant remain of brooklet. Nilsson and Persson (1993) report *Laccophilus vitshumbii* collected at light, in temporary ponds in almost dry stream (1450–2350 m a.s.l.). Omer-Cooper (1931) recorded the species in high altitudes (5500–7500 ft). Additional information may be gathered from the literature, e.g. Bilardo and Rocchi (1987) and Pederzani (1988). Often collected at light and with light traps.

#### Laccophilus olsoufieffi

Taxon classificationAnimaliaColeopteraDytiscidae

Guignot, 1937

Figs 71–72 , 265 , 420–421 , 545

Laccophilus olsoufieffi Guignot 1937: 141 (original description, faunistics); Gschwendtner 1938a: 5 (faunistics); Guignot 1941: 36 (description, discussion); Guignot 1946c: 269, 273, 274, 275, 276, 278, 312 (description, faunistics); Guignot 1959a: 558, 562, 565, 566 (description, discussion, faunistics); Bameul 1984: 94 (faunistics); Hernando 1990: 177, 178 (discussion, description); Rocchi 1991: 86 (faunistics, list); Nilsson 2001: 248 (catalogue, faunistics); Pederzani and Reintjes 2002: 40 (faunistics); Nilsson 2015: 215 (catalogue, faunistics).

##### Type locality.

Madagascar: Maroansétra.

##### Type material studied

(8 exs.). Holotype: male: “Maroansétra, Madagascar X. 1936 / male symbol / Type” (MNHN). – Paratypes: males and females: “Madagascar Maroansetra X 1936 / Paratype” (1 ex. IRSNB); “Antakotako Madagascar II 1936 / female-symbol / Paratype” (1 ex. IRSNB); same data but “male symbol” (1 ex. MNHN; habitus in Fig. 416); same data, but with “n. spec. det. Gschwendt”, (1 ex. MNHN); “Male / Madagascar Vatomandry VIII. 1934 Vadon / Paratype” (1 ex. AMGS); “Madagascar Antakotako 11. 1936 / female-mark / Paratype” (2 exs. AMGS).

##### Additional material studied

(12 exs.). **Madagascar**: “Tananarive 7. 1934 Vadon / Lac Tzimbamzaza / male symbol / Type / ab. fuscinus” (1 ex. MNHN). [Comment: the specimen has no status as type material being associated with the name ab. fuscinus, which is infrasubspecific.] – “E-Mad. Ampamoho nr Andilamena 1200-1300 m asl.18-20.1. 1995 Dunay & Janak” (7 exs. NMW, 2 exs. MZH; habitus in Fig. 420); “Ese 5 km S Ampamoho pr. Andimalena 1. 1995 G. Dunay & J. Janak leg.” (1 ex. NMPC); “Toliara Menabe, Kirindy RS, S20.07655, E044.67532, 57 m.a.o., 12.12. 2009, water net, field, Bergsten et al. / 000000470 NHRS-JLKB” (1 ex. NHRS).

##### Diagnosis.

Resembles most of and probably closely related to *Laccophilus adspersus* from which *Laccophilus olsoufieffi* can generally be distinguished by study of the penis. Minor difference can be recognized in bending of the penis. Moreover, body of *Laccophilus olsoufieffi* seems to be slightly more robust than *Laccophilus adspersus* in general. In *Laccophilus olsoufieffi* irroration covers often almost entire elytron but sometimes there is posterior to middle a patch with sparse irroration or irroration is totally absent. Further study may reveal that the two species are synonymous.

##### Description.

Body length 3.8–4.3 mm, width 2.1–2.4 mm. Specimens regarded as aberration “fuscinus” are slightly larger; length 3.9–4.4 mm, width 2.2–2.5 mm. Additionally “fuscinus” lacks pale area (irroration absent or strongly reduced) posterior to middle of elytron. Habitus and dorsal colour pattern (Figs 420–421).

Head: Pale ferrugineous. Slightly dull, rather finely reticulated. Reticulation double; large meshes contain 3–4, often indistinct small meshes. Almost impunctate, except at eyes; fine and scattered punctures may be discerned.

Pronotum: Pale ferrugineous to ferrugineous; no distinct colour pattern. Submat, reticulated; reticulation quite distinct and double. Large meshes may contain 4–7 small meshes. Anteriorly and laterally with fine, in part indistinct, scattered punctures.

Elytra: Pale ferrugineous, extensively provided with ferrugineous irrorations (Figs 420–421). Somewhat posterior to middle with a vague transverse area where irrorations extensively absent (forming a vague transverse pale marking interrupted by suture). Rarely pale area lacking; “ab. fuscinus”. Submat, finely and quite distinctly reticulated. Reticulation distinctly double; large meshes contain generally 3–6 smaller meshes. Laterally, sublaterally and discally with sparse and irregular punctures (forming longitudinal areas with scattered puncture). Lateral, pre-apical furrow fine, finely pubescent.

Ventral aspect: Pale ferrugineous to ferrugineous, distinct colour pattern lacking. Rather shiny to submat; extensively with fine reticulation, which in part is rather indistinct. Basal ventrites with rather distinct, curved striae. Almost impunctate. Apex of prosternal process slender, slightly extended and pointed. Metacoxal plates in anterior half with fine, transversely located, shallow furrows; in posterior half furrows absent. Apical ventrite asymmetric, with knob on one side (Fig. 71).

Legs: Pro- and mesotarsi slightly enlarged, extended, with suckers.

Male genitalia: Penis long, bended and extreme apex points forwards (Fig. 265).

Female: Apical ventrite symmetric, lacks knob (Fig. 72). Protarsus slender; claws slightly extended and moderately curved.

##### Distribution.

Madagascar (Fig. 545). Records outside Madagascar are to be considered uncertain.

##### Collecting circumstances.

Not documented.

#### Laccophilus modestus

Taxon classificationAnimaliaColeopteraDytiscidae

Régimbart, 1895

Figs 73–74 , 266 , 422 , 546

Laccophilus modestus Régimbart 1895: 133 (original description, faunistics); Régimbart 1906: 248 (faunistics, disussion.); Zimmermann 1920a: 23 (catalogue, faunistics); Guignot 1946c: 270, 273, 276, 278, 312 (discussion, description, faunistics); Guignot 1952c: 521 (faunistics); Capra 1952: 6 (faunistics); Guignot 1953b: 234 (faunistics); Guignot 1955b: 1096 (faunistics); Guignot 1956a: 88 (faunistics); Omer-Cooper 1958b: 37, 47, 48, 49 (discussion, description, faunistics); Guignot 1958: 8 (discussion); Guignot 1959a: 562, 566, 568 (description, discussion, faunistics); Guignot 1959d: 162 (discussion, faunistics); Guignot 1961b: 238 (faunistics); Omer-Cooper 1965: 77, 87 (description, faunistics); Omer-Cooper 1970: 293 (description); Legros 1972: 466 (faunistics); Bilardo and Pederzani 1978: 119 (faunistics, description); Medler 1980: 155 (faunistics, list); Bilardo and Rocchi 1990: 177 (faunistics); Nilsson and Persson 1993: 81, 94 (faunistics, biology); Nilsson et al.1995: 505 (faunistics); Rocchi 2000: 24 (faunistics); Nilsson 2001: 247 (catalogue, faunistics); Bilardo and Rocchi 2002: 174 (list, faunistics); Pederzani and Reintjes 2002: 40 (faunistics); Reintjes 2004: 68 (faunistics); Nilsson 2015: 214 (catalogue, faunistics).
Laccophilus modestus v. tostus Régimbart 1895: 133, 134 (original description, faunistics); Zimmermann 1920a: 23 (catalogue, faunistics); Guignot 1946c: 270 (description); Guignot 1959a: 568 (female description, faunistics); Guignot 1959d: 162 (discussion, faunistics); Bilardo and Pederzani 1978: 119 (faunistics, description); Nilsson 2001: 247 (catalogue, list, synonymy *Laccophilus modestus* Régimbart); Nilsson 2015: 214 (catalogue, faunistics, list, synonymy). **Confirmed synonym**.
Laccophilus espanyoli Hernando 1990: 177 (original description, faunistics); Nilsson 2001: 243 (catalogue); Pederzani and Reintjes 2002: 40 (faunistics); Nilsson 2015: 211 (catalogue, faunistics). **New synonym.**

##### Type localities.

*Laccophilus modestus*: Mali: Badoumbé (Ht. Senegal).

Laccophilus modestus var. tostus: Gabon: Cap Lopez.

*Laccophilus espanyoli*: Senegal: Oussaduye.

##### Type material studied

(9 exs.). *Laccophilus modestus*: Lectotype (by present desgination): male: “Ht. Sénégal Badoumbé Dr. Nodier I à V – 1882 / male symbol / co-type / *Laccophilus modestus* Rég.” (MNHN). [Comment: Guignot (1959a) indicates existence of a lectotype but survey of collections in Paris museum reveals that no such specimen can be distinguished; see also Nilsson (2001).] – Paralectotypes: Same data as lectotype but, labelled with female symbol (1 ex. MNHN); “Badoumbé / Museum Paris coll. Maurice Régimbart 1908 / *modestus* Rég. Koppi Wehncke” (1 ex. MNHN).

Laccophilus modestus var. tostus: Cotype: female: “Gabon Mocquerys / female symbol / Cotype” (MNHN). Additionally, three specimens mounted together and labelled “Gabon Mocquerys/Museum Paris Coll. Maurice Régimbart 1908 / modestus Rég. v. tostus Rég.” probably also belong to the type material but have no type indication (3 exs. MNHN).

*Laccophilus espanyoli*: Holotype: male: “Holotip / Senegal Oussaduye 13-XI-65 Sala leg. / *Laccophilus espanyoli* sp. n. C. Hernande det. / 78-0572 MZB” (MZBS). – Paratype: female: Same data as holotypus but “Paratypus / 78-0752 MZB” (1 ex. MZBS).

##### Additional material studied

(371 exs.). **Gambia**: “Abuko Nat. Res., at light at the Bamboo Pool 18.30-20.30, 18.11. 1977 UTM 28PCK2181 / Cederholm et al. N. 1977” (3 exs. MZLU); “Tendema Camp, at light in semiarid veg near river Gambia 18.30-20.30, 14.11. 1977, UTM 28POK1285, loc. 12A / Cederholm et al. N. 1977” (1 ex. MZH); “Riv. Tanji 3 km SW Brufut. At light 19.00-21.00, 28.2 1977, UTM 28PCK087773 / Cederholm et al. Febr.-March 1977” (2 exs. MZLU, 1 ex. NHMB); “Outside Abuko Nat. Res., at waterworks. At light 19.00-22.00 UTM 28PCK214812 / Cederholm et al. Febr-March 1977” (2 exs. MZLU); “Bathurst Jan. 68 Palm / *Laccophilus modestus* Régb.det. Persson” (5 exs. MZLU); “Bathurst Januari 1968 Leiler” (3 exs. NHRS); “Kuntaur NW Georgetown 21.11. 2003 Vondel” (1 ex. CSR). – **Gambia/S. Senegal**: “Stream N of Selety 13°10'N, 16°36'W, 19.2. 1976 Holmen” (2 exs. ZMUC). – **Senegal**: “Riv. Cazamance Carabane Dr. Collin / Museum Paris coll. Maurice Régimbart 1908” (2 exs. MNHN); “3 km SSW Toubakouta, 10 km S Ziguinchor, 4.3. 1977, at light 19.00-22.00, Loc. No. 16, UTM 28PCJ585782 / Cederholm et al. Febr-March 1977 / *Laccophilus modestus* Rég. det. M. Brancucci” (3 exs. MZLU, 6 exs. NHMB); “Swamps ca 3 km SW Ziguinchor 8.3. 1977, UTM 28 PJC59-89- / Cederholm et al. Febr- March 1977” (5 exs. MZLU); “2.5 km ESE Ziguinchor in cultivated area, at light 20-21.30, 11.11. 1997 / Cederholm & al. (1 ex. NHMB); “In forest, 1 km NE Djibelor, ca 7,5 km SW Ziguinchor, at light 19-21. 9.11. 1977 / Cederholm & al.” (1 ex. NHMB); “Ht Senegal Badoumbé 1-5. 1882 Nodier” (1 ex. SAMC); “60 km S Velingara Pakour 27.6. 2004 leg. Marek Halada” (2 exs. NMPC); “1 km NW Bignona 26 km N Ziguinchor, at light 19.15-20.30, 3.3. 1977, UTM 28PCK654170 / Cederholm & al.” (1 ex. NHMB). – **Guinea Bissau**: “Cachheu 12 km E Varela, earth pit pond, 9.4. 1993 Persson” (12 exs. MZLU). – **Guinea**: “Seredou 4.4. 1975, lux Zott leg.” (2 exs. ZMHB). – **Mali**: “Haut Sénégal Khayes Dr. Nodier 11-12 1881 / female symbol / Co-type / *Laccophilus modestus* Rég.” (2 exs. MNHN; not type material due to deviating label data); “Kogoni 10. 1966 Schmitz” (1 ex. MRAC, 1 ex. MZH); “K. Macina 10.11. 1973 Reynolds” (18 exs. BMNH, 4 exs. MZH); “San, Bani river 13°18'N, 4°54'W, 22.2. 2000 Komarek & Meyer / *Laccophilus modestus* Régb. det. Wewalka 2001” (1 ex. NMW); “Kéniéroba 70 km SW Bamako 12°06’ N 8°20'W, 1. 2011 Kravchenko” (1 ex. TAU). – **Niger**: “Niamey 9. 1988, at light, Jongema / *Laccophilus modestus* Régb. det. Wewalka 2005” (7 exs. CGW). – **Burkina Faso** (= Ht Volta): “Ouagadougou X. 1926” (1 ex. NHMB); “Haute Volta Bobo Dioulasso / Museum Paris 12 – 1930 – IV 1931 Ch. Alluaud & P. Chappuis / male symbol / *Laccophilus modestus* Rég.” (1 ex. MNHN; habitus in Fig. 422); “Nadiagow MV August 2005 Moretto” (3 exs. NHMB). – **Chad**: “nr Bongor 27.5. 1973 Linnavuori” (2 exs. MZH). – **Sudan**: “Upper Nile, Malakal 5-20.1. 1963 Linnavuori” (12 exs. MZH); “Sudan Malakal 1963 Linnavuori” (1 ex. MZH); “Dahr el Ghazal, Wau 19.2. 1963 Linnavuori” (2 exs. MZH); same but “R. Malmul 21.2. 1963” (1 ex. MZH); Equatoria Lalyo-Juba 26-27. 2. 1963 Linnavuori” (1 ex. MZH); “Gilo water tank (pumped up from stream) 20.3. 1980 Armstrong” (1 ex. USNM); “Kinyetti River at Imeila 19.3. 1980 Armstrong” (2 exs. USNM); “Senaar a. bl. Nil, lux 21.10. 1979 Hieke” (1 ex. NHMB); “Nyangwara, stream from hot springs, N4.39, E30.5, 29.1. 1954 J. Omer-Cooper” (1 ex. AMGS); “Sandy River 50 mi NW Juba 29.1. 1954J. Omer-Cooper” (1 ex. AMGS); “Moya Sawu 45 mi from Amadi-Juba rd 29.1. 1954” (1 ex. AMGS). – **Liberia**: “Suakoko 8.4. 1953” (3 exs. USNM); “Suakoko 18-19.3. 1952 / Blickenstaff Light trap” (6 exs. USNM, 2 exs. MZH); “Suakoko 27.2. 1952 Blickenstaff” (1 ex. USNM); “Suakoko 22.25.2. 1952 Blickenstaff” (2 exs. USNM); “Suakoko 14.3. 1952 / 6-9 pm light trap” (2 exs. USNM). – **Sierra Leone**: “Makeni 12°03'W, 8°53'N, 27.11. 1993, light trap / Cederholm-Danielsson-Hall / *Laccophilus modestus* Régb. det. Nilsson 94” (10 exs. MZLU; 2 exs. MZH); same as but “det. Persson” (4 exs. MZLU); “Kalangba 8.11. 1980 D. Jump leg.” (1 ex. USNM). – **Ivory Coast**: “Bingerville 1-12.3. 1962 Decelle” (1 ex. MRAC); same but “7. 1962” (1 ex. MRAC); “Comoé NP, N8,5° - W3,5°, Reintjes / 9.1. 1999” (2 exs. NMW); same but “28.2. 1999” (1 ex. NMW); same but “21.3. 1999” (1 ex. NMW); “Touba, à la lumière 4. 2002 Moretto / *Laccophilus modestus* Régb. det. Rocchi 2002” (1 ex. CSR). – **Ghana**: “Upper East Pr., Navrongo env. 11-13.6. 2006 Pokorny S.” (2 exs. NMPC); “N Reg. Nyankpala 15 km W von Tamale leg. Endrödi / Lichtfalle 1-30.4. 1970” (1 ex. CGW); “N Reg. Damongo Mole game res. 220 m, N9°04’ – W1°48’ Endrödy-Younga / on light 12.8. 1971” (1 ex. CGW); “Ashanti Reg. Kumasi Nhiasu 330 m, N 6.43-W 1.36 Endrödy-Younga / at light 12.6. 1967” (1 ex. CGW); “Volta Reg., Volta Riv. at Kpong 28.11. 1993 light trap T. Andersen leg.” (1 ex. MZH). – **Ethiopia**: “Bahar Dar 8.10. 1968 Harde leg.” (2 exs. CGW). – **Benin**: “Zagnanado Dahomey” (1 ex. MNHN); “Dep. Atlantique, Allada, Glotomè (village) 31.1.2006 leg. Goergen et al. / 06°41'06,8"N, 02°02'36,8"E, 17 m asl, slowly running stream” (3 exs. NMW, 1 ex. MZH); same but “Awoute / 06°39'54,9"N, 02°09'34,1"E, 25 m asl, small ponds” (1 ex. NMW); “Dep. Zou, Zogbodomé, Lokoli (forest), Hlanzoun riv. 3, 6.2. 2006 leg. Goergen et al. / 0.7°03’ N, 02°15’ E, muddy stream” (6 exs. NMW, 2 exs. MZH);“Dep. du Zou, commune de Zogbodomé 29.1. 2006 Goergen / Lokoli forest 07°03'N, 02°15'E, 17 m asl, light trap” (4 exs. NMW, 1 ex. MZH); “Calav i IITA, light trap: fallow 20.6. 2004 Goergen” (1 ex. NMW). – **Nigeria**: “Ibadan ca. Jan-Juni 1954 Stenholt Clausen / *Laccophilus mediocris* G. det. J. Balfour-Browne” (1 ex. ZMUC); “Ibadan, at light 27.11. 1955” (1 ex. BMNh); same but “26.9. 1956” (1 ex. BMNH); “NW St. Badeggi rice fields 8-9.8. 1973 Linnavuori” (133 exs. MZH); “W. St. Ife 7-8.7. 1973 Linnavuori” (1 ex. MZH); “EC St. Norcap near Abakaliki 29.6. 1973 Linnavuori“ (2 exs. MZH); “Zaria 1969 à la lumière Roberts” (1 ex. MRAC); “Zaria pr. Zaria 5-6.3. 1949 Malkin” (1 ex. BMNH); “Zaria 1969” (1 ex. NHMB); “Samaru Endrödy Younga / Light trap 20.10. 1969” (1 ex. CGW); “River, Bauchi rd 21 mi from Jos 9.4. 1963 J. Omer-Cooper” (1 ex. AMGS); “Katsina-Dawyra rd, Marsh, 6.4. 1963 JOC” (4 exs. AMGS). – **Cameroon**: “Akonolinga, moist secondary forest and plantation, at light, 7.1. 1978 / *Laccophilus modestus* Régb. det. Nilsson” (2 exs. MZLU); “Yaounde Bor to Kosti by boat 3-14.3. 1978 Perkins leg.” (8 exs. USNM, 1 ex. MZH); “Maroua 26.8. 1973” (1 ex. NHMB); “Maroua, Nayo Tsanaga 26.10. 1977” (1 ex. NHMB); “Emana Obala 16.5. 1970” (1 ex. NHMB). – **Central African Republic**: “Bozo, lum. 11. 1981 / Degallier” (1 ex. NHMB). – **Congo**: “Parc Nat. d’Odzala, Mboko-Lango 21.8. 2002 Bilardo” (4 exs. CSR). – **Zaire**: “Tshuapa, Mbandaka ca. 0°03'N - 18°28'E, 8-9.3. 1963 a.l., Stam” (1 ex. RMNH); same but “15-16.3. 1963” (1 ex. RMNH); “Banana Boma M. Tschoffen 91 Det. Régimb. / *Laccophilus livens* Régb. det. Régimbart 1891 / Ex Types” (1 ex. IRSNB; type material of *Laccophilus livens*); “Boma M. Tschoffen” (1 ex. SAMC); “PNG I/c/2”’, 25.2. 1950 Demoulin 259” (1 ex. IRSNB); “PNG I/a/2”’, 30.1. 1950 Demoulin 240” (1 ex. IRSNB); “PNG I/b/3’, 15.2. 1950, Demoulin 253” (1 ex. NHMB); “PNG I/a/4, 6.3. 1950, Demoulin 297” (1 ex. NHMB).

##### Specimens with uncertain determination.

**Tanzania**: “Deforested place nr Mangula, 297 m, at light, 18.7. 2004 Sprecher” (1 ex. NHMB; single female specimen); “Mizimu Mwanihana Mnts N.P. S07.48.21,8, E36.51.09,5, 850 m, 3-6.8. 2010 light trap Smith & Takano” (1 ex. BMNH; single female specimen). – **Mozambique**: “Mandambuzi, Manda Wilderness Res. S12°17.697’, E34°46.260’ Watson 16.2. 2008” (1 ex. CGF; single female specimen).

##### Comments on synonymy.

The lectotype of *Laccophilus modestus* and holotype of *Laccophilus espanyoli* have been examined and compared. Morphological features in shape of penis and external appearance of body show that the two taxa are conspecific. *Laccophilus modestus*, being the older name is the valid name of the species. Earlier established synonymy of Laccophilus modestus var. tostus and *Laccophilus modestus* is also confirmed (name given for female being dimorphous, vide below under female description).

##### Diagnosis.

*Laccophilus modestus* is characterized by appearance of elytra; irrorations reduced basally, and by features exhibited by the penis; inner outline of penis provided with a minute but distinct knob. Extreme apex of penis frontally rounded (vide diagnosis of *Laccophilus cryptos* on p. 112).

##### Description.

Body length 3.5–3.8 mm; width 1.9–2.1 mm. Elytral irrorations are dark ferrugineous to ferrugineous against pale ferrugineous background (Fig. 422). Most specimens have vague irrorations on elytra but basal part of elytra to a variable degree lacking darker markings.

Head: Pale ferrugineous. Submat, finely microsculptured. Reticulation double, but due to minor size difference division in two mesh-size classes difficult. Almost impunctate; at eyes small area with fine, dense and irregular punctures.

Pronotum: Pale ferrugineous, no distinct colour pattern. Submat, finely microsculptured. Reticulation double; large meshes contain 3–6 small meshes. At margins with very fine, irregular punctures. Basally punctures hardly visible; only laterally clearly discernible.

Elytra: Pale ferrugineous, with slightly vague ferrugineous to dark ferrugineous, quite dense irrorations. Sometimes, irrorations anteriorly reduced and almost absent (Fig. 422). Submat, finely microsculptured. Reticulation double, but large meshes only discernible in mediobasal area. Large meshes, when discernible, contain 3–6 small meshes. Very fine, somewhat irregular punctures form a discal, dorsolateral and lateral row, out of which the two latter rows are rather indistinct.

Ventral aspect: Pale ferrugineous to ferrugineous, no colour pattern exhibited. Almost impunctate. Scattered, single punctures may be discerned. Rather shiny, although very finely microsculptured. In part reticulation reduced or absent. Abdomen with fine, slightly curved striae. Prosternal process slender, apex extended and pointed. Metacoxal plates frontally with indistinct, shallow furrows. Apical ventrite asymmetric, with single, minute, lateral knob (Fig. 73).

Legs: Pro- and mesotarsus slightly enlarged, somewhat extended and provided with distinct suckers.

Male genitalia: Penis (Fig. 266) in lateral aspect with extreme apex rounded and not sharply angled as in *Laccophilus cryptos* (Fig. 267).

Female: Externally resembles male but pro- and mesotarsus slender. Apical ventrite lacks knob, almost symmetric (Fig. 74). Some female specimens have strongly developed dorsal reticulation making body dull. Female is accordingly dimorphous. This extreme morph was named *var. tostus* and here listed as synonym of *Laccophilus modestus*.

##### Distribution.

Gambia, Senegal, Guinea Bissau, Guinea, Mali, Niger, Burkina Faso, Sudan, Ethiopia, Liberia, Sierra Leone, Ivory Coast, Ghana, Benin, Nigeria, Cameroon, Central African Republic, Gabon, Congo and Zaire (Fig. 546). A single female specimen from Mozambique and two females from Tanzania are considered uncertain. Due to widespread confusion of species-status and delimitation of it, only verified records are included in the map. Additional country records are Tanzania (Zanzibar) (Régimbart 1895), Kenya (Régimbart 1906), Somalia (Capra 1952), Malawi (Omer-Cooper 1958b) and South Africa (Omer-Cooper 1965).

##### Collecting circumstances.

Information in literature is uncertain but can be found by checking the references above. Label data indicate that the species occur both in stagnant and running waters: collected in swamps and small ponds as well as in a slowly running stream and in a muddy stream. Also collected at light, e.g. in semiarid vegetation near a river and in moist secondary forest and in a plantation.

#### Laccophilus cryptos sp. n.

Taxon classificationAnimaliaColeopteraDytiscidae

http://zoobank.org/CE49BCA0-9D3D-4045-8C70-2BB6B21520EF

**Figures 75–84.**
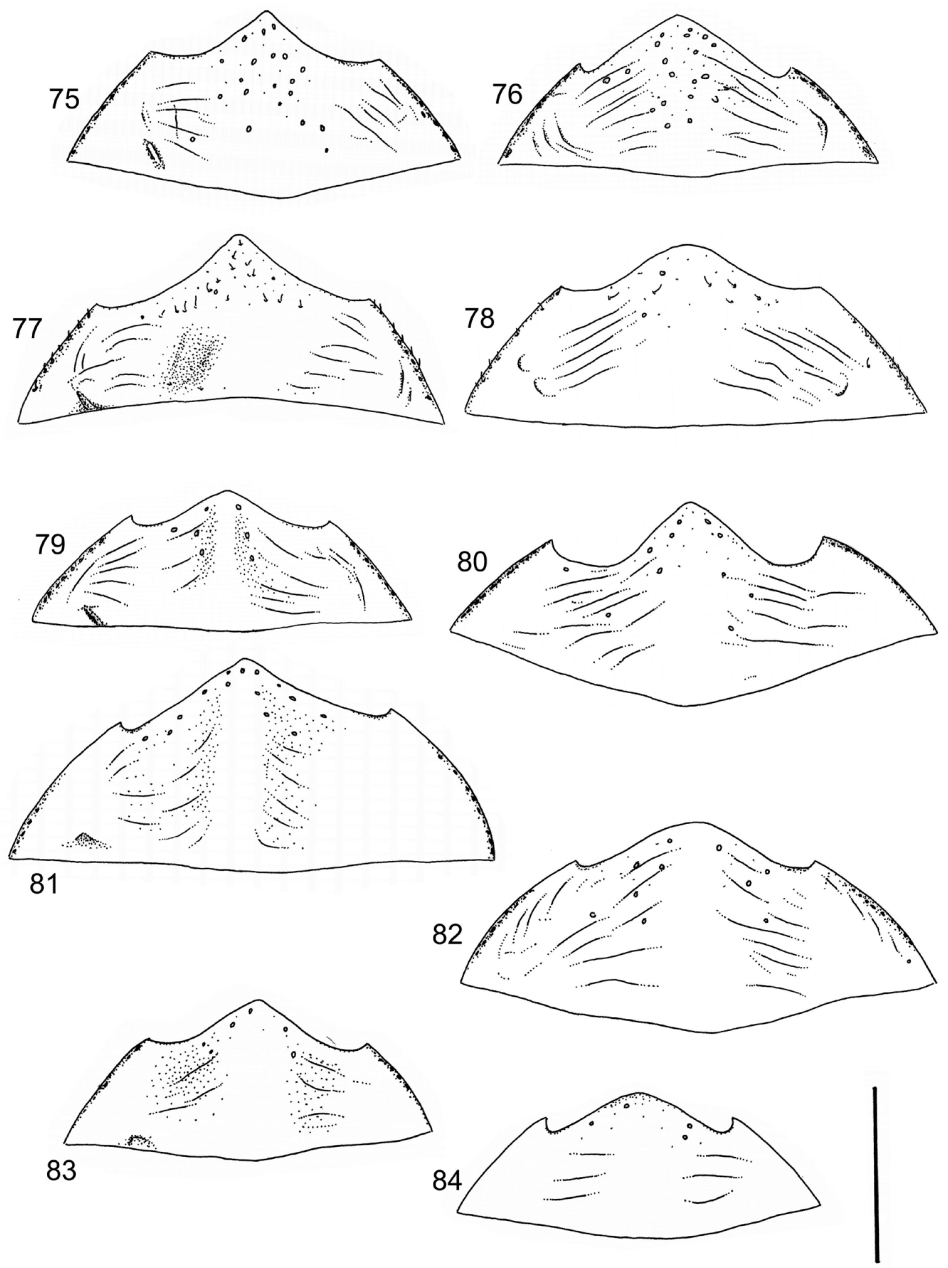
Apical ventrites **75** *Laccophilus cryptos* male, and **76** female **77** *Laccophilus nodieri* male, and **78** female **79** *Laccophilus flaveolus* male, and **80** female **81** *Laccophilus remex* (sp. gr.), **82** female **83** *Laccophilus turbatus* male, and **84** female. Scale bar 0.5 mm.

Figs 75–76 , 267 , 423 , 547

##### Type locality.

South Africa: Zululand, Mission Rock, St. Lucia (28.22S-32.35E).

##### Type material studied

(24 exs.). Holotype: male: “S. Afr.; Zululand St. Lucia, Mission Rock 28.22S-32.35E / 18.12. 1975; E-Y: 983, at black light leg. Endrödy-Younga” (TMSA). – Paratypes: Same data as holotype (3 exs. TMSA, 1 ex. MZH; habitus in Fig. 423); “S. Afr.; Zululd Ndumu Banzi, fresh wat. pan 26.53S-32.16E / 16.2. 1989; E-Y: 2612 shorewashing, Endrödy & Klimaszew” (2 exs. TMSA). – Zaire: “Coll. Mus. Congo, Elisabethville (à la lumière) I-1956/I-1957 Ch. Seydel” (1 ex. MRAC). – Mozambique: “Prov. Sofala 10 km NW Save 6-7.12. 2003 A. Kudrna jr. lgt.” (1 ex. CFP). – Zimbabwe: “Zimbabwe centr. 30 km S Harare 30.11. 1998 leg. F. Kantner” (1 ex. NHMB). – Namibia: “SW Africa Tondoro Okawango 20-23.1. 1975 leg. H. Roer” (1 ex. CGW); “SWA / Namibia Nyangara / Okawango 1-9.4. 1988 leg. H. Roer” (3 exs. CGW). – Botswana: “V.-L. Kal. Exp. Tsotsorogo Pan 17.6.-9.7. 1930 / *Laccophilus livens* Régb. det. L. Gschwendtner” (9 exs. TMSA, 1 ex. MZH).

##### Diagnosis.

Very closely related to *Laccophilus modestus*. Correct determination requires examination of the penis, the apex of which exhibits distinct, species-specific features. In *Laccophilus cryptos* extreme tip of penis apex clearly angled while rounded in *Laccophilus modestus*.

##### Description.

Body length 3.3–3.8 mm, breadth 1.7–2.0 mm. Dorsal, colour pattern slightly variable. Elytra with somewhat obscure, dark ferrugineous irrorations, which at base generally, are almost lacking (Fig. 423).

Head: Pale ferrugineous to ferrugineous; lacks distinct colour pattern. Rather shiny, although finely microsculptured; reticulation double. Large meshes only slightly more strongly developed than small meshes. Large meshes contain 2–5 small meshes. Impunctate, except at eyes; with fine, irregular punctures. Area of punctures extends towards disc-middle, still leaving a considerable impunctate area in middle of head.

Pronotum: Pale ferrugineous to ferrugineous; lacks distinct colour pattern. Rather shiny, although finely microsculptured; reticulation double. Large meshes only slightly more strongly developed than small meshes. Large meshes contain 2–5 small meshes. Smaller meshes, in part, rather indistinct. Impunctate, except frontally and laterally; with fine, irregular punctures.

Elytra: Pale ferrugineous, with somewhat vague, dark ferrugineous irrorations, which often disappear at base of elytra (Fig. 423). Rather shiny, although finely microsculptured. Large meshes only slightly more strongly developed than small meshes. Large meshes contain 2–5 small meshes. Laterally and posteriorly size-categories of microsculpture disappear. Fine, somewhat irregular punctures form a clearly discernible discal row of punctures. Dorsolateral and lateral rows of punctures indistinct; simply indicated by a few, fine and scattered punctures. Pre-apical, lateral row of punctures form a shallow by discernible, pubescent furrow.

Ventral aspect: Pale ferrugineous to ferrugineous; without distinct colour pattern. Rather shiny, although very finely microsculptured; microsculpture in part indistinct. Ventrites with very fine, slightly indistinct, curved striae. Metacoxal plates with a number of rudimentary, transverse furrows. Lateral impression of metacoxal plates moderately deep. Furrows weakly developed, in part indistinct. Impunctate, except apical ventrite, with some fine, scattered punctures and an asymmetric minute knob locate on one side of the ventrite (Fig. 75). Prosternal process slender, posteriorly somewhat extended; apex pointed.

Legs: Pale ferrugineous to ferrugineous. Pro- and mesotarsus slightly enlarged, with distinct suckers.

Male genitalia: Extreme apex of penis tip angled (Fig. 267).

Female: Pro- and mesotarsus slender. Apical ventrite lack small, asymmetric knob (Fig. 76). Body microsculpture variable; sometimes more strongly developed and denser than in male; sometimes as male.

##### Etymology.

The species name *cryptos* is a Greece noun in apposition and refers to something hidden or secret. The name refers to the identity of the new species, which remained hidden until male genitalia were dissected, being externally similar to *Laccophilus modestus*.

##### Distribution.

Zaire, Mozambique, Zimbabwe, Namibia, Botswana, South Africa (Fig. 547).

##### Collecting circumstances.

Almost unknown. The species has been collected by shore washing and at light collection. Recorded also, from a fresh water pan.

#### Laccophilus nodieri

Taxon classificationAnimaliaColeopteraDytiscidae

Régimbart, 1895

Figs 77–78 , 268 , 424 , 547

Laccophilus nodieri Régimbart 1895: 134 (original description, faunistics); Guignot 1946c: 270, 273, 277 (description, faunistics, discussion); Guignot 1959a: 562, 567 (description, faunistics); Bruneau de Miré and Legros 1963: 873, 888 (faunistics); Bilardo and Pederzani 1978: 119: (faunistics, description); Biström 1979: 22 (faunistics); Medler 1980: 155 (faunistics, list.); Nilsson 2001: 247 (catalogue, faunistics); Pederzani and Reintjes 2002: 38, 40 (discussion, faunistics); Reintjes 2004: 68 (faunistics); van Vondel 2005: 131 (faunistics); Nilsson 2015: 215 (catalogue, faunistics).

##### Type locality.

Mali: Badoumbé.

##### Type material studied

(2 exs.). Lectotype (by present designation) male: “Ht. Sénégal Badoumbé Dr. Nodier I à V 1882 / male symbol /Co-Type / *Laccophilus nodieri* Rég. cotype” (MNHN; habitus in Fig. 424). – Paralectotype: female: “Badoumbé Ht. Senegal / Museum Paris coll. Maurice Régimbart 1908 / *nodieri* Reg.” (1 ex. MNHN). [Comment: most probably the female paralectotype belongs to *Laccophilus modestus*.]

##### Additional material studied

(70 exs.). **Gambia**: “W Div. Abuko Nat. Res. 27.11. 2003 Vondel / *Laccophilus nodieri* det. Rocchi” (1 ex. CSR); “Abuko Nat. Res., at light at the Bamboo pool 18.30-20.30 18.11. 1977, UTM 28PCK2181 / Cederholm et. al leg. / *Laccophilus nodieri* Rég. det. Brancucci 85” (1 ex. MZLU). – **Senegal**: “Casamance Tabor” (1 ex. MNHN); “Dakar 5. 1939” (2 exs. MNHN). – **Mali**: “Goundaka, Bandiagara river 14°29'N, 3°56'W, 12.2. 2000 Komarek & Mayer / *Laccophilus nodieri* Rég. det. Wewalka 2001” (1 ex. NMW); “W Bandiagara, pools 14°22'N, 3°41'W, 12.2. 2000 Komarek & Mayer / *Laccophilus nodieri* Rég. det. Wewalka 2001” (1 ex. NMW); “W50 km E Djenne 13°50'N, 4°25'W, 12.2. 2000 Komarek & Mayer / *Laccophilus nodieri* Rég. det. Wewalka 2001” (1 ex. NMW). – **Sudan**: “Aluakulak 30,5E 6,30N 14.5. 1954 / *Laccophilus nodieri* Reg. det. J. Omer-Cooper” (1 ex. AMGS); “Alel rock pool 30,56 E 6,11N 18.I. 1954 JJOC.” (7 exs. AMGS); “Sandy river 50 mi. NW of Juba 29.1. 1954 JJOC.“ (4 exs. AMGS); “Stream from hot springs Nyangwara 30,51E 4,39N, 29.I. 1954 JJOC.” (2 exs. AMGS); “Sandy river 50 mi. NW of Juba 29.I. 1954 JJOC.” (2 exs. AMGS). – **Ivory Coast**: “Comoé N8,5° - W3,5°, 22.3. 1999 Reintjes” (6 exs. NMW); same and *“Laccophilus nodieri* det. Rocchi” (3 exs. CSR). – **Burkina Faso**: “Pundu, Mte Volta 1927-28 Dez-Juni Olsufiejev” (3 exs. NHRS). – **Ghana**: “17 mi S Palbe 1.9. 1971, filtered black light, Gruwell” (1 ex. USNM). – **Nigeria**: “Stream crossing Kaduna rd. nr Zaria 4.4. 1963 JOC.” (1 ex. AMGS); “Trib. of R. Gagere en route Zaria-Katsina 5.IV. 1963 JOC.” (2 exs. AMGS); “Stream 64 mi. from Bida on Jebba rd.15.IV. 1963 JOC.” (1 ex. AMGS); “Detritus pond 45 mi. from Jos on Bauchi rd.9.IV. 1963 JOC.” (1 ex. AMGS); “R. Niger, bridge Kontagora-Kaduna rd. 3.IV. 1963 JOC.” (1 ex. AMGS); “Kontagora stream 3.IV. 1963 JOC.” (1 ex. AMGS); “Stream nr. Zaria 4.IV. 1963 JOC.” (8 exs. AMGS); “Zaria Pr., Zaria 5-6.3. 1949 Malkin” (1 ex. BMNH, 1 ex. MZH); “NC St. Zaria 2-3.8. 1973 Linnavuori” (1 ex. MZH); “NW St. Yelwa 23.7. 1973 Linnavuori” (1 ex. MZH); “Kano St., Kano-Wudil 17.5. 1973 Linnavuori” (1 ex. MZH); “R. Ogun, Olokomeji nr. Ibadan 24.III. 1963 JOC.” (2 exs. AMGS); “Pond, road Dawria-Kano 6.4. 1963 JOC.” (4 exs. AMGS). – **Zaire**: “PNG, Utukuru 14/s, 22.7. 1952 De Saeger 3812” (2 exs. NHMB).

##### Diagnosis.

Externally *Laccophilus nodieri* resembles very much of *Laccophilus flaveolus*. Both species have elytral irrorations in part reduced, which makes coverage of it uneven and patchy. Shape of penis, however, is peculiar with a distinct, sharp knob in inner curvature. Corresponding sharp knob lacks in *Laccophilus flaveolus*.

##### Description.

Body length 4.0–4.3 mm, width 2.2–2.4 mm. Elytral colour pattern consists of rather fine, in part unevenly distributed irrorations (Fig. 424).

Head: Pale ferrugineous; no distinct colour pattern. Impunctate, except at eyes; with fine, dense and somewhat irregular punctures. Areas of punctures extend a little towards middle of head. Slightly mat, finely microsculptured. Reticulation almost simple; only indistinct fragments of large meshes discernible. Large meshes, when discerned, contain 3–6 fine meshes.

Pronotum: Pale ferrugineous; lacks colour pattern. Rather shiny, although very finely microsculptured. Reticulation double, but difference between size classes small. Large meshes contain 3–6 fine meshes. At margins with fine, scattered and irregular punctures, which basally in middle are indistinct.

Elytra: Pale ferrugineous, with distinctly reduced, somewhat vague dark ferrugineous irrorations. Irrorations are sparsest basally and in a transverse area posterior to middle (Fig. 424). Slightly mat; finely microsculptured. Reticulation indistinctly double; difference between size classes small. Large meshes, when discernible, contain 3–6 fine meshes. Mesh-organization in part slightly vague. Almost impunctate except for discal row of punctures; consist of fine, irregularly placed fine punctures. Dorsolateral and lateral row of punctures reduced to a few irregular fine punctures.

Ventral aspect: Pale ferrugineous to ferrugineous; no distinct colour pattern exhibited. Almost impunctate. Submat to rather shiny, very finely, in part indistinctly microsculptured. Metacoxal plates in frontal half with some transversely located very shallow furrows. Abdomen basally with fine curved striae. Prosternal process rather slender, apex slightly extended and pointed. Apical ventrite asymmetric; provided with a minute but sharp lateral knob (Fig. 77).

Legs: Pro- and mesotarsus slightly enlarged, somewhat extended and provided with distinct suckers.

Male genitalia: Penis in lateral aspect slightly curved, broad, provided with a sharp, minor process, approximately in middle of the inner side of penis. Extreme apex of penis hooked (Fig. 268).

Female: Externally as male but pro- and mesotarsus slender. Apical ventrite not distinctly asymmetric; lacks lateral knob (Fig. 78).

##### Distribution.

Gambia, Senegal, Mali, Sudan, Ivory Coast, Burkina Faso, Ghana, Nigeria, Zaire (Fig. 547). Personally verified records only accepted in map. Additional country records are Chad (Bruneau de Miré and Legros 1963), Guinea (Reintjes 2004) and Benin (van Vondel 2005a).

##### Collecting circumstances.

Sampled e.g. in pools with loamy bottom and in stagnant remain of brooklet (van Vondel 2005a). Label data give the species to be collected from various pools, at light and in a sandy river.

#### Laccophilus flaveolus

Taxon classificationAnimaliaColeopteraDytiscidae

Régimbart, 1906

Figs 79–80 , 269 , 425 , 548

Laccophilus flaveolus Régimbart 1906: 249 (original description, faunistics); Zimmermann 1920a:18 (catalogue, faunistics); Gschwendtner 1930: 88 (faunistics, description, discussion); Guignot 1943: 98 (faunistics, discussion); Guignot 1946c: 265, 268, 272, 273, 312, 315 (discussion, description, faunistics); Guignot 1948: 15 (faunistics); Guignot 1950a: 262, 263 (faunistics); Guignot 1952e:167 (discussion); Legros 1954: 268 (discussion); Guignot 1955e: 2 (discussion); Guignot 1956b: 220 (discussion); Omer-Cooper 1958b: 37, 48, 49 (discussion, description, faunistics, biology); Guignot 1959a: 557, 561, 562 (description, faunistics); Guignot 1959d: 161 (discussion, faunistics); Omer-Cooper 1965: 77, 87 (description, faunistics); Legros 1972: 466 (faunistics); Bameul 1984: 94 (faunistics); Pederzani 1988: 107 (faunistics, biology); Rocchi 1991: 86 (faunistics, list); Nilsson 2001: 243 (catalogue, faunistics); Pederzani and Reintjes 2002: 38, 40 (discussion, faunistics); Nilsson 2015: 211 (catalogue, faunistics).
Laccophilus pampinatus Guignot 1941: 35 (original description, faunistics); Guignot 1946c: 269, 273, 274, 276, 313 (description, faunistics, discussion); Guignot 1959a: 558, 562, 564, 565, 566, 568 (redescription, discussion, faunistics); Nilsson 2001: 248 (catalogue, faunistics); Pederzani and Reintjes 2002: 38, 40 (discussion, faunistics); Nilsson 2015: 215 (catalogue, faunistics). **New synonym.**

##### Comment on identity.

Confusion in determination of species has been a common problem. Accordingly, records and data from old literature must be considered carefully.

##### Type localities.

*Laccophilus flaveolus*: Kenya: Baie de Kavirondo.

*Laccophilus pampinatus*: Uganda: Central Uganda.

##### Type material studied

(37 exs.). *Laccophilus flaveolus*: Lectotype (by present designation) male: Kenya “Lac Victoria Nyanza Baie de Kavirondo IX-X. 1903 / male symbol / Cotype” (MNHN). – Paralectotypes: Same data as lectotype (2 exs. MNHN; habitus in Fig. 425); “Cotype / Victoria Nyanza Kavirondo-Bay / Brit. Mus.1905-199 / *Laccophilus flaveolus* Rég. sp. n. type” (1 ex. BMNH); “Cotype / Lac Victoria Nyanza Baie de Kavirondo IX-X-1903” (1 ex. IRSNB); *“Laccophilus flaveolus* Baie de Kavirondo Lac Victoria Nyanza Alluaud IX-X. 1903” (4 exs. IRSNB); same data and on same pin (1 ex. IRSNB; belongs to *Laccophilus pallescens* Régimbart); Afr. Orle Anglaise Baie de Kavirondo (Victoria Nyanza N-E.) Ch. Alluaud IX-X-1903 / Museum Paris coll. Maurice Régimbart 1908 / *Laccophilus flaveolus* Rég. sp. n. type / Type” (20 exs. MNHN); “Baie de Kavirondo (Alluaud) / Museum Paris coll. Maurice Régimbart 1908 / *flaveolus* Rég.” (3 exs. MNHN).

*Laccophilus pampinatus*: Holotype: male: Uganda, “Ouganda Central Alluaud I-II. 1909 / male symbol / Type / Det. Dr. Guignot *Laccophilus pampinatus* Guign. Type” (MNHN). – Paratypes: Uganda, Same as holotype, but labelled as “Paratype” (1 ex. MNHN). – Kenya, “Lac Victoria Baie de Kavirondo Alluaud IX. 1903 / male symbol / *Laccophilus pampinatus* Guignot Paratype / Paratype” (1 ex. MNHN); “Afr. Or. Angl. (Lac Victoria) Baie de kavirondo Alluaud & Jeannel Déc. 1911 – 1112 - St. 22 et 23 / female symbol / *Laccophilus pampinatus* Guignot Allotype female / Allotype” (1 ex. MNHN).

##### Additional material studied

(155 exs.). **Sudan**: “Kawrajena 20.3. 1947” (2 exs. ZMUC). – **Zaire**: “PNA 23.8. 1957 Vanschuytbroeck / Secteur Nord, rive dr. Semliki, rte Muramba, 905 m” (6 exs. MRAC, 1 ex. MZH); “PNA 26.8. 1957 Vanschuytbroeck / Secteur Nord, marais Buyansha sur r. dr. Semliki, 905 m” (6 exs. MRAC); “PNA 27.8. 1957 Vanschuytbroeck / Secteur Nord, rive Ihunga, af dr. Semliki 1300 m” (2 exs. MRAC); “PNG Ndelele K. 117/14s 19.3. 1952 H. De Saeger” (2 exs. MRAC); “PNG Ndelele/14s 1.8. 1952 H. De Saeger” (2 exs. MRAC); “PNG PpK.14/g/14s, 4.4. 1952 H. De Saeger 3290” (4 exs. MRAC, 1 ex. MZH); PNG II/gd/14, 30.7. 1952 H. De Saeger” (1 ex. MRAC); “PNG II/gd/4, 29.5.1952 De Saeger” (1 ex. IRSNB). – **Uganda**: “Uganda Central Alluaud I-II. 1909 / Cotype” (1 ex. MNHN; not type material); “Butiaba Flats 2.9. 1967 Brown” (1 ex. BMNH). – **Kenya**: “Dam at Kaloleni Mission, Kilifi Distr. 15.9. 1976 Holmen” (1 ex. ZMUC). – **Tanzania**: “Ukerewe I. Father Conrad” (5 exs. BMNH, 1 ex. MZH); “Tanganyika Ukerewe / *Laccophilus pampinatus* Guignot det. Wewalka 1979” (7 exs. OLML); “Mwanza nr Lake Victoria 1957 / Marginal pools and ditches” (2 exs. BMNH, 1 ex. MZH); “Mwanza nr Lake Victoria / Sweet potato channels” (1 ex. BMNH); “Stream, Mbeya-Tunduma rd., 18.10. 1948 JOC” (1 ex. AMGS); “Foothills of Kilimandjaro 14.2. 1954” (2 exs. AMGS); “Zanzibar Pemba, Sept, 1955 Fowler” (2 exs. AMGS). – **Zambia**: “S Luangwa NP, Mfuwe Crocodile Farm 23.3. 1993 13°06'03"S-31°47'32"E, 450 m, lux Uhlig leg.” (1 ex. ZMHB); “Lusaka 5.11. 1973 Lange” (1 ex. CGC). – **Malawi**: “River nr Portuguese border nr Mwanza 9.2. 1948” (1 ex. AMGS); “Zomba plateau, stream 6000 ft 7.3. 1948” (2 exs. AMGS). – **Mozambique**: “Magude16.8. 1915 C.J. Sw. / *Laccophilus adspersus* Boh. det. Gschwendtner” (8 exs. TMSA). – **Zimbabwe**: “Small stream Halfway Hotel Salisbury-Gatooma 14.9. 1948” (1 ex. AMGS); “Salisbury 14.9. 1948 J. Omer-Cooper” (2 exs. NHMB); “Wankie Game Res. Masumu Dam Sept. 1948 JOC.” (4 exs. AMGS); “Wankie Game Res. Waterhole Sept. 1948 JOC.” (1 ex. AMGS); “5 mi SE Wankie 7.4. 1968 Spangler” (25 exs. USNM, 5 exs. MZH); “Pool Lundi 22. N. 1948 JOC.” (20 exs. AMGS); “Sinkukwe 30.12. 1948 J.O.C.” (6 exs. AMGS). – **Botswana**: “Tsotsorogo Pan 17.6.-9.7. 1930 V.-L. Kal. Exp. / *Laccophilus adspersus* Boh. Gschwendtner det.” (9 exs. TMSA, 1 ex. MZH); same data but *“Laccophilus pampinatus* Guignot det. Wewalka 1979” (1 ex. OLML); “Kabulabula Chobe River 11-24.7. 1930 V.-L. Kal. Exp. / *Laccophilus adspersus* Boh. Gschwendtner det.” (1 ex. TMSA); “N’Kate Makarikari 6-23.8. 1930 V.-L. Kal. Exp. / *Laccophilus adspersus* Boh. Gschwendtner det.” (2 exs. TMSA); “Metsimaklaba 7-12.3. 1930 V.-L. Kal. Exp. / *Laccophilus adspersus* Boh. Gschwendtner det.” (3 exs. TMSA). – **South Africa**: “Kruger Nat. Pk, Skukuza res. camp, 25.00S-31.35E/19.2.1995 UV-light & trap E-Y: 3102, Endrödy-Younga leg.” (1 ex. TMSA); “Kruger Nat. Pk, Skukuza 12 km, 25.04S-31.37E / 6.3. 1996 UV-light E-Y: 3217, Endrödy-Younga leg.” (1 ex. TMSA, 1 ex. MZH); “Trsvl Kruger National Pk, Leeu Pan NE Skukuza 1.5. 1951 / Brinck-Rudebeck / *Laccophilus flaveolus* Régb. det. J. Omer-Cooper” (1 ex. MZLU); “Mpumalanga, 7 km upstream from Skukuza, Sabie R. N-24.970, E31.540, 25.10. 1990 de Moor” (1 ex. AMGS); “Kruger Nat. Pk, Levuvu River 22.27S-31.10E / 12.2. 1994 E-Y: 2998 shorewashing” (1 ex. TMSA); “Trsvl., Naboomspruit Torino Ranche 24.37S-28.38E / 15.1. 1989 E-Y: 2774, UV light, vlei edge Endrödy-Younga leg.” (1 ex. TMSA). – **Madagascar**: “Zombitse Ankilemiletsy, muddy waterhole N- 22.868, E 44.576, 544 m 14.5. 2006 Bergsten/BMNH(E) <794187> DNA voucher / *Laccophilus flaveolus* Régb. det. Bergsten” (1 ex. NHRS); same data, but “<794193> DNA voucher” (1 ex. NHRS).

##### Comments on synonymy.

The lectotype of *Laccophilus flaveolus* and the holotype of *Laccophilus pampinatus* have been examined and compared. No morphological features, which would justify separation of two species were detected. Accordingly, they are synonyms and *Laccophilus flaveolus* being the older name is the valid name for the species.

##### Diagnosis.

*Laccophilus flaveolus* is separated from resembling species by study of elytral colour pattern in combination with peculiarly shaped penis. Externally it resembles most of *Laccophilus nodieri* but there is clear difference in shape of penis: Penis of *Laccophilus flaveolus* lacks sharp knob on inner outline. Additionally, penis long, medially somewhat bent and extreme apex hooked with extreme tip sharp (Fig. 269).

##### Description.

Body length 4.2–4.6 mm, width 2.3–2.4 mm. Pale coloured except elytra. Elytral colour pattern is formed by quite extensive irrorations; irrorations somewhat sparse and in part reduced (Fig. 425).

Head: Pale ferrugineous. Rather shiny to submat, reticulation double but difference between size-categories small. Large meshes contain 2–10 finer meshes when they are discernible. Almost impunctate; at eyes with fine and irregularly distributed punctures. Area of punctures at each eye extends towards middle but areas are not connected.

Pronotum: Pale ferrugineous, base in middle often with narrow darkened area. Rather shiny although finely microsculptured; reticulation double. Large meshes contain 2–10 meshes; sometimes fine meshes indistinct and hardly visible. At margins with very fine, scattered punctures; pronotum discally impunctate.

Elytra: Pale ferrugineous, extensively with dark brown to dark ferrugineous, rarely slightly variable irrorations (Fig. 425). Same groundplan of irrorations discernible, although slight reduction sometimes present. Rather shiny to submat, finely microsculptured; reticulation double. Large meshes only slightly coarser than fine meshes. Large meshes contain between 2–7 fine meshes. Posteriorly fine and large meshes are mixed so that separate size categories are not discernible. Impunctate, except for three irregular longitudinal rows of very fine and scattered punctures, located discally, dorso-laterally and laterally. Lateral, pre-apical furrow rather shallow, distinctly pubescent.

Ventral aspect: Pale ferrugineous. Slightly mat, very finely microsculptured. Metacoxal plates with some very indistinct transverse furrows. Ventrites basally sparsely striated; striae curved. Almost impunctate. Apex of prosternal process slender and pointed. Apical ventrite with a minute but distinct asymmetric knob. A minor knob can also be detected on the other side (Fig. 79).

Legs: Pale ferrugineous. Pro- and mesotarsus slightly enlarged and extended, with suckers.

Male genitalia: Penis long, medially somewhat bent and extreme apex hooked with extreme tip sharp (Fig. 269).

Female: Apical ventrite as in Fig. 80. Pro- and mesotarsus slender. Epipleuron slightly enlarged posterior to middle and external edge can sometimes be detected when specimen is studied from above.

##### Distribution.

Sudan, Zaire, Uganda, Kenya, Tanzania, Zambia, Malawi, Mozambique, Zimbabwe, Botswana, South Africa and Madagascar (Fig. 548). Confusion in the determination of species frequent. Accordingly only verified records are mapped. Additional country records, not verified, are Ivory Coast and Mali (Omer-Cooper 1965) and Senegal (Legros 1972).

##### Collecting circumstances.

Insufficiently known. Omer-Cooper (1958b) reports the species from various minor water bodies, as streams and ponds. Label data give as collection technique UV light collection, light trap and shorewashing. Detailed information is not available.

#### Laccophilus remex

Taxon classificationAnimaliaColeopteraDytiscidae

Guignot, 1952

Possibly a species complex Figs 81–82 , 270–274 , 426–429 , 549

Laccophilus remex Guignot 1952e: 167 (description, faunistics); Guignot 1953e: 4 (discussion); Guignot 1954: 25 (discussion); Guignot 1956b: 219 (faunistics); Omer-Cooper 1958b: 37, 47 (discussion, description, faunistics); Guignot 1958: 7 (discussion); Guignot 1959a: 535, 557, 560, 562 (description, discussion, faunistics); Omer-Cooper 1965: 77, 85 (description, faunistics); Medler 1980: 155 (faunistics, list); Pederzani and Rocchi 1982: 72 (faunistics); Nilsson and Persson 1993: 80, 94 (faunistics); Nilsson, Persson and Cuppen 1995: 505 (faunistics); Bilardo and Rocchi 1999: 232, 234 (faunistics); Nilsson 2001: 250 (catalogue, faunistics); Bilardo and Rocchi 2002: 174 (list, faunistics); Pederzani and Reintjes 2002: 38, 40 (discussion, faunistics); Bilardo and Rocchi 200a: 291 (faunistics); Bilardo and Rocchi 2006a: 130 (faunistics); Nilsson 2015: 217 (catalogue, faunistics).
Laccophilus concisus Guignot 1953e: 4 (original description, faunistics); Guignot 1954: 24 (description, faunistics); Guignot 1958: 7 (discussion); Guignot 1959a: 558, 562, 563 (description, faunistics); Guignot 1961b: 238 (faunistics); Omer-Cooper 1962: 295 (faunistics); Omer-Cooper 1965: 77, 86 (description, faunistics); Omer-Cooper 1967: 60 (discussion, synonym *Laccophilus praeteritus* O-C. = *Laccophilus concisus* Guig.); Bilardo 1982: 447 (description, faunistics; given as *Laccophylus concisus*); Bilardo and Rocchi 1987: 104 (faunistics, biology); Nilsson et al. 1995: 505 (faunistics); Rocchi 2000: 23 (faunistics); Nilsson 2001: 242 (catalogue, faunistics); Pederzani and Reintjes 2002: 38 (faunistics); Nilsson 2015: 210 (catalogue, faunistics). **New synonym.**
Laccophilus turneri Omer-Cooper 1956: 21 (no description, faunistics, biology); Omer-Cooper 1957: 17, 90 (original description, faunistics); Omer-Cooper 1958b: 47 (discussion); Omer-Cooper 1965: 86 (synonym *Laccophilus remex* Guignot ); Nilsson and Persson 1993: 80 (list, synonymy); Nilsson et al. 1995: 505 (list, synonymy); Nilsson 2001: 250 (catalogue, faunistics, list, synonymy); Pederzani and Reintjes 2002: 40 (faunistics); Nilsson 2015: 217 (catalogue, list, synonymy). **Confirmed synonym.**
Laccophilus praeteritus Omer-Cooper 1957: 18, 90 (original description, faunistics); Omer-Cooper 1958b: 37, 47, 48, 49 (discussion, description, faunistics, biology); Omer-Cooper 1965: 86 (list synonymy, *Laccophilus concisus* Guignot); 1967: 60 (discussion, synonymy); Nilsson 2001: 242 (catalogue, faunistics, list, synonymy); Pederzani and Reintjes 2002: 40 (faunistics, list, synonymy); Nilsson 2015: 210 (catalogue, list, synonymy). **New synonym** of *Laccophilus remex*.

##### Type localities:

*Laccophilus remex*: Ivory Coast: Duékoué.

*Laccophilus concisus*: Zaire: PNU, Lusinga.

*Laccophilus turneri*: South Africa: Boekenhout, Nylstroom (River Nyl at Num Num).

*Laccophilus praeteritus*: South Africa: Transvaal, Ermelo.

##### Type material studied

(26 exs.) *Laccophilus remex*. Holotype: male: “Cote d’Ivoire Duékoué / Museum Paris 12-1930-IV-1931 Ch. Alluaud et P.A. Chappuis / Det. Dr. Guignot *Laccophilus tschoffeni* Rég. / Type / F. Guignot det. 1952 *Laccophilus remex* Guign. Type, male symbol” (MNHN). – Paratypes: females: “Cote d’Ivoire Duékoué / female symbol / Museum Paris 12-1930-IV-1931 Ch. Alluaud et P.A. Chappuis / Paratype” (1 ex. MNHN); same data but labelled “Allotype” (1 ex. MNHN).

*Laccophilus concisus*: Holotype: male: “Holotypus / Congo Belge: PNU, Lusinga (Galerié) 22-25-V-1945 G. F de Witte: 29 / Coll. Mus. Congo (ex. coll. I.P.N.C.B.) / *Laccophilus concisus* Guign. sp. n. Type male / F. Guignot det., 1952 *Laccophilus concisus* sp. n. Type male” (MRAC; habitus in Fig. 427). – Paratypes: “Congo Belge: PNU, Lusinga (Galerié) 22-25-V-1945 G. F de Witte: 29 / Paratype / F. Guignot det., 1952 *Laccophilus concisus* sp. n. / R.I.Sc.N.B. I.G. 24.054” (1 ex. IRSNB); same but “7-20-VI-1945” and “191” (2 exs. IRSNB); same, but “Kabwekanono (1.815 m) 18-III-1947” and “64a” (1 ex. IRSNB).

*Laccophilus praeteritus*: Holotype: male: “Male / Type / Transvaal Ermelo / Dec. 1948 J. Omer-Cooper / *Laccophilus praeteritus* Omer-Cooper/Brit. Mus. 1957-660 / *Laccophilus concisus* Guign. J. Balfour-Browne det. 1960” (BMNH). – Paratypes: “Female / Type / Transvaal Belfast 30.XI. 1948 J.O.C. / Female allotype / Brit. Mus. 1957-660 / *Laccophilus praeteritus”* (1 ex. BMNH); “Paratype / Transvaal pond W. Belfast 23.11. 1948 JOC. / *Laccophilus praeteritus* O-C. det. J. Omer-Cooper” (1 ex. AMGS); “Paratype / Duivels Kloof Merenskydam 24.11. 1948 Omer-Cooper” (1 ex. AMGS); “Transvaal sluggish stream / nr Ermelo 1.12. 1948 J.O.C. / *Laccophilus praeteritus* O-C. / Paratype” (1 ex. TMSA).

*Laccophilus turneri*: Holotype: male: “Type / Transvaal Nylstroom R. Nyl at Num Num 23. Aug. 1948 J. Omer-Cooper / Type, male / *Laccophilus turneri* O-C.” (BMNH). – Paratypes: female: Principally with same data as holotype, but labelled as “female allotype” (1 ex. BMNH); Almost labelled as holotype but “paratype” (6 exs. AMGS, 1 ex. TMSA); “Paratype / Transvaal Waterberg distr. Deel Kraal 20.8. 1948 JOC.” (1 ex. AMGS); “Paratype / Transvaal Nylstroom 19 Aug. 1948 JOC. / *Laccophilus adspersus* Boh. J. Balfour-Browne det.” (1 ex. AMGS); “Paratype / Transvaal Duivels Kloof 24. N. 1948 JOC.” (1 ex. AMGS); “Paratype” (1 ex. AMGS; status uncertain, because locality information absent).

**Additional material, studied** (278 exs.): **Sudan**: “Dahr el Ghazal M’Boloko 23.2. 1963 Linnavuori” (1ex. MZH); “Equatoria, Nzara 22.4. 1986 Wewalka” (1 ex. CGW); “O. Sudan Adjuba I.U. Neumann” (1 ex. ZMHB). – **Sierra Leone**: “Musaia 16.1. 1946 / Hippo mud pan” (1 ex, BMNH). – **Liberia**: “Suakoko 19.12, 1951/6-9 pm light trap Blickenstaff” (9 exs. USNM, 2 exs. MZH); “Suakoko 11.12. 1951 Blickenstaff” (1 ex. USNM, 1 ex. MZH); “Suakoko 1.1. 1952 Blickenstaff” (6 exs. USNM). – **Ivory Coast**: “Divo 28.11. 1963 Decelle” (2 exs. MRAC). – **Ghana**: “Ashanti Kumasi 330 m, N6.43-W 1.36 / 15.9. 1967 at light Endrödy-Younga” (1 ex, TMSA); “Kumasi 3.6. 67 Endrödy-Younga / *Laccophilus remex* Guignot det. Wewalka 76” (1 ex. MHNG); same data but “6.7. 67” (1 ex. MHNG); same data but “16.7. 67” (3 exs. MHNG); same data but “24.6. 67” (2 exs. MHNG). – **Nigeria**: “Stream 64 mi. from Bida on Jebba rd. 15.IV. 1963 JOC.” (3 exs. AMGS); “EC St. Norcap nr Abakaliki 29.6. 1973” (1 ex. MZH); same and “ad lucem / Linnavuori” (1 ex. MZH). – **Gabon**: “Makoukou Riv. Oua (Ivindo) 16-19.1. 2001 Bilardo / *Laccophilus remex* det. Rocchi 02” (2 exs. CSR); “Belinga 5.2.-4.4. 1963 Coiffat” (62 exs. NHMB). – **Congo**: “Parc Nat. d’Odzala Mboko-Lango 21.8. 2002 Bilardo” (1 ex. CSR); “Odzala NP, 400 m, 10.2. 1997 Murzin leg.” (4 exs. NMPC). – **Zaire**: “PNG Miss. H. De Saeger II/fd/14, 18.6. 1951, 1946” (1 ex. NHMB); “PNG Miss. H. De Saeger II/hd/17, 13.10. 1951, 2595” (1 ex. NHMB); “PNG Miss. H. De Saeger II/fd/14s, 3.4. 1952, 3278” (9 exs. MRAC, 2 exs. MZH); “PNG Miss. H. De Saeger II/fd/12, 10.3. 1952, 3180” (13 exs. MRAC, 1 ex. MZH); “PNG Miss. H. De Saeger Pali/8, 22.3. 1952, 3217” (1 ex. MRAC); “PNG Miss. H. De Saeger II/fd/13, 5.5. 1952, 3421” (1 ex. MRAC); “PNG Miss. H. De Saeger II/fc/14, 17.7. 1952, 3806” (1 ex. MRAC); “PNG Miss. H. De Saeger I/a/M 7.6. 1950 Rec. G. Demoulin 584 / *Laccophilus remex* Guignot det. Guignot 1957” (1 ex. AMGS); “PNG Miss. H. De Saeger II/fc/14, 4.7. 1952, 3736 / Paratype of *Laccophilus saegeri* Guignot” (1 ex. IRSNB); “PNU Mukana, 1810 m, 24.3. 1947 / Dr. F. Guignot det., 1953 *Laccophilus concisus* Guign.”(2 exs. IRSNB, 2 exs. MRAC; labelled as paratypes but not mentioned in original description); “PNU Kabwekanono p.t.s. Lufwa affl. dr. Lufira (1.815 m) 12.1. 1948, 1199a” (1 ex. MRAC; labelled as paratype but not mentioned in original description); “PNU Kaswabilenga (700 m) 17.10. 1947, 845a” (1 ex. MAC; labelled as paratype but not mentioned in original description); “PNU Mubale – 1480 m, 10-13.5. 1947, 352a” (2 exs. MRAC; labelled as paratypes but not mentioned in original description); “de Luebo à Luluabourg N. 1921 Ghesquière / *Laccophilus remex* Guignot det. Wewalka 1979” (1 ex. OLML). – **Tanzania**: “Kondoa 300 m 10. 1938” (1 ex. MNHN; habitus in Fig. 426); “Mlowa R. Tunduma-Mbeya rd. 16.10. 1948” (1 ex. AMGS); “Rukwa 26.12. 1961 C. Carnegie” (3 exs. AMGS); “T.T. Rukwa Tumba 12.1. 1991 T. river Backlund / *Laccophilus remex* Guignot det. Nilsson -96” (1 ex. MZLU); “Ukerewe VIII., 2 / 1294 B / *Laccophilus remex* Guign. det. Wewalka 79” (4 exs. OLML); “Zansibar Küste Hildebr.” (1 ex. ZMHB). – **Zambia**: “Katambora 40 ml W Victoria Falls, April, 1962” (1 ex. BMNH); “Kapiri Mpushi env. 13.12. 2002 Kantner” (2 exs. NHMB). – **Malawi**: “Nyasal. Bua R. 2.10. 1948 JOC.” (2 exs. AMGS). – **Mozambique**: “Mandambuzi, Manda Wilderness Res. S12°17.679’, E34°46.260’, Watson 16.2. 2008” (2 exs. CGF); “Umbeluzi R. Dec. 4. 1948 JOC.” (2 exs. AMGS). – **Namibia**: “E Caprivi 10 km SE Katima Mulilo 17°31'S/24°25'E, Zambesi Altwasserarm, lux 6.3. 1992 Uhlig” (5 exs. ZMHB); “Kavango Popa Falls, lux 18°07'S/21°35'E, 26.2.-3.3. 1992 Uhlig leg.” (1 ex. ZMHB). – **Botswana**: “Kabulabula Chobe River 11-24.7. 1930” (1 ex. OLML); “Maun Thamalakane R. 10.10. 1982 Bilardo / *Laccophilus concisus* G. det. Bilardo” (2 exs. MSNM). – **Zimbabwe**: “Kyle Recr. Pk at Lake Mutirikwi 1-5.12. 1993, lux Uhlig” (1 ex. ZMHB); “Stream Rusapi 13.11. 1948” (3 exs. AMGS); “Pool Lundi 22. N. 1948 JOC.” (5 exs. AMGS); “Stream between Salisbury-Bromley 12.11. 1948” (6 exs. AMGS); “Small stream Halfway Hotel-Gatooma, Salisbury 14.9. 1948 / *Laccophilus turneri* O-C. det. J. Omer-Cooper” (1 ex. AMGS); “Pool Lundi 22. N. 1948 JOC.” (11 exs. AMGS); “Kariba env. 20.12. 1998 Kantner” (1 ex. NHMB). – **South Africa**: “Transvaal Ermelo stream 7. Dec. 1948 JOC.” (1 ex. AMGS); “Transvaal Ermelo Dec. 1946” (1 ex. AMGS); “Kruger Nat. Pk. Skukuza Res. camp 24.59S-31.35E/3.3. 1996 UV-light Endrödy-Younga” (1 ex. TMSA); “Kruger Nat. Pk. Skukuza Res. camp 25.00S-31.35E/19.2. 1995 UV-light & trap Endrödy-Younga” (1 ex. TMSA); “Trsvl Bundu Inn 25.28S-28.55E/24.3. 1974 at merc. vap. light, Endrödy-Younga” (2 exs. TMSA); “Natal Lions R. 30.3. 1960” (2 exs. AMS); “Natal Zululand Hlui-Hlui 15.9. 1947” (1 ex. AMGS); “Zululd Ndumu Nyamithi, saline pan 26.54S-32.16E/12.6. 1989 shorewashing Endrödy-Klimaszew” (1 ex. TMSA); “Kwazulu-Natal Ndumu 25.55S-32.18E / 21.11. 2002 light trap Harrison & Müller” (1 ex. TMSA; habitus in Fig. 428); “Zululd Ndumu Banzi, fresh wat. pan 26.53S-32.16E / 16.2. 1989 shorewashing Endrödy & Klimaszew” (7 exs. TMSA, 1 ex. MZH); “Blinkwater Reserve, Greytown Natal, first stream from entrance, 1100 m mist belt grassland 4.2. 1997 Turner” (2 exs. AMGS, 1 ex. CCT); “Kwazulu Natal” (1 ex. AMGS); “Gen. 766G 3.7. 1962 (= Insese River near Empangeni station 6, Tugela River)” (1 ex. AMGS); “W. C. Prov., pond on Plettenberg rd, Knysna Distr. 14.2. 1947 JOC.” (2 exs. AMGS); “WCP, Knysna 34°04.28'S, 23°04.11'E 9 m Hotový & Mateju leg.” (2 exs. NMPC); “E. C. Prov. Uitenhage 5.12. 1954 JOC.” (1 ex. AMGS); “E. C. Prov. Mount Currie 6.5. 1956 JOC.” (6 exs. AMGS); “E. C. Prov. Mount Currie 13.11. 1957 JOC.” (6 exs. AMGS); “E. C. Prov. Hogsback 2. 1942 O.C.” (3 exs. AMGS); “E. C. Prov. E. London pond nr. Mooiplaats 8.3. 1955” (1 ex. AMGS); “E. C. Prov. Maclear 9.5. 1956 JOC.” (10 exs. AMGS); “E. C. Prov. nr. Zwatrberg 1960 Chutter” (3 exs. AMGS); “E. C. Prov. Krom R. 1960” (2 exs. AMGS); “E. C. Prov. Albany stream Storm May 1939” (3 exs. AMGS); “E. C. Prov. Albany Distr. Grahamstown 10.3. 1946 JOC.” (1 ex. AMGS); “E. C. Pr. Albany distr. Grahamstown Teafontein stream (33 26 AA) 3.V8. 1939 JOC.” (1 ex. AMGS); “CPr. Knysna Main Forest Buffelsnek 18.I. 1951 / Brinck-Rudebeck / *Laccophilus concisus* Guig. = *Laccophilus praeteritus* O-C. det. JOC” (3 exs. MZLU; habitus in Fig. 429); “CP., Pond NO Knysna on Hwy R340 8.3. 1997 Challet” (4 exs. CGC, 1 ex. MZH); “Cape, Gatberg R, vlei, N-31.250, E28.120, 26.3. 1993 De Moor & al.” (1 ex. AMGS).

##### Specimen with uncertain determination.

South Africa: “Blinkwater Reserve, Greytown Natal, bog on summit, 1100 m nist belt grassland 4.2. 1997 Turner” (1 ex. CCT).

##### Comments on synonymy and species status.

Male holotypes of *Laccophilus remex*, *Laccophilus concisus*, *Laccophilus turneri* and *Laccophilus praeteritus* have been examined and compared. Shape of penis is almost identical in the four taxa. Considering all examined male genitalia there is variation in shape between extremes, which makes a separation of different species difficult or almost impossible. Colour pattern of elytra is quite variable but exhibit similar ground-plan with transitional morphs between extremes. Accordingly, the four taxa are for the time being regarded, conspecific and synonymies are introduced as follows: Earlier synonymy of *Laccophilus remex* and *Laccophilus turneri* is confirmed as well as earlier synonymy of *Laccophilus concisus* and *Laccophilus praeteritus*. *Laccophilus concisus* is a new synonymy of *Laccophilus remex*. *Laccophilus remex*, being the oldest available name is the valid name of the species. An alternative, plausible, interpretation is that *Laccophilus remex* is in fact a complex of very closely related species. With present knowledge, the delimitation of the different species remains, however, an open question. Further study is definitely needed.

##### Diagnosis.

Although *Laccophilus remex*, as delimited here, exhibits considerable variation in appearance of elytral colour pattern this feature can often be used for recognition of the species. The species is also characterized by the robust penis, which often exhibits some variation. *Laccophilus remex* resembles very much of *Laccophilus turbatus* but *Laccophilus remex* is almost always larger (body length exceeds in most cases 4 mm). Additionally external outline of penis is rounded when it is angled in *Laccophilus turbatus*.

##### Description.

Body length 4.0–4.7 mm; width 2.2–2.6 mm. Elytral colour pattern variable; elytra covered with dense irrorations, which basally often are to a variable degree reduced. Basally often with quite extensive pale areas where irrorations are absent. Additionally, elytra sometimes with irrorations being sparser posterior to middle (Figs 426–429).

Head: Pale ferrugineous. Posteriorly sometimes with dark area. Submat, finely microsculptured. Reticulation double; large meshes contain 2–7 smaller meshes. At eyes with fine and irregular punctures, which extend towards middle of head.

Pronotum: Pale ferrugineous. At frontal margin and medially at posterior margin with a distinct dark to blackish ferrugineous area. Basal dark area sometimes reduced. Frontal, dark marking sometimes totally absent. Submat, finely microsculptured; reticulation double: larger meshes contain 2–7 smaller meshes. Frontally and laterally with scattered, fine punctures.

Elytra: Pale ferrugineous, with dense, dark ferrugineous to blackish irrorations. At base, irrorations often sparser, in part reduced, sometimes forming variable pale areas lacking irroration (Figs 426–429). Submat, finely microsculptured; reticulation double: large meshes contain 2–6 smaller meshes. Posteriorly two kinds of microsculpture become mixed and difficult to separate. Laterally and posteriorly double reticulation becomes obscure and indistinct. Elytron with discal, dorsolateral and lateral rows of fine and irregular punctures. Pre-apical, lateral furrow with fine hairs.

Ventral aspect: Pale ferrugineous to ferrugineous. Almost impunctate. Rather shiny, with very fine microsculpture. Metacoxal plates with a few, rather vague, transverse furrows. Ventrites with fine, somewhat curved striae. Apical ventrite of male with fine knob on one side (Fig. 81). Apex of prosternal process narrow, pointed.

Legs: Pro- and mesotarsus slightly enlarged and extended, with suckers.

Male genitalia: Penis in lateral aspect quite broad, medially distinctly bended and extreme apex hooked, outline of it, however, rounded (Figs 270–274).

Female: Pro- and mesotarsus rather slender. Apical ventrite symmetric (Fig. 82).

##### Distribution.

Sudan, Sierra Leone, Liberia, Ivory Coast, Ghana, Nigeria, Gabon, Congo, Zaire, Tanzania, Malawi, Zambia, Namibia, Botswana, Zimbabwe, South Africa and Swaziland (Fig. 549). Unverified country records are Senegal and Guinea (Guignot 1956b), Mozambique (Omer-Cooper 1965), Ethiopia (Nilsson and Persson 1993) and Guinea Bissau (Nilsson et al. 1995). Additionally, under the name *Laccophilus concisus*, Bilardo (1982) gives Cameroon.

##### Collecting circumstances.

Information in literature is superficial and sparse. Omer-Cooper (1958b) reports the species from a river with clear water and with water lilies, reed beds and patches of swamp. Also collected from pools e.g. in a tributary. Label data indicate that the species is often caught with light traps or at light. Moreover the species has been collected in various kinds of pools and ponds but also from running water as streams.

#### Laccophilus turbatus

Taxon classificationAnimaliaColeopteraDytiscidae

Guignot, 1958

Figs 83–84 , 275 , 430–431 , 550

Laccophilus turbatus Guignot 1958: 8 (original description, faunistics); Omer-Cooper 1965: 86 (list, synonymy *Laccophilus concisus* Guignot?); Nilsson 2001: 242 (catalogue, faunistics, list, synonymy, *Laccophilus concisus* Guignot); Pederzani and Reintjes 2002: 40 (faunistics, list, synonymy, *Laccophilus concisus* Guign.); Nilsson 2015: 210 (catalogue, faunistics, list, synonymy, *Laccophilus concisus* Guignot. **Restored species.**

##### Type locality.

Zaire: Parc National Garamba.

##### Type material studied

(8 exs.): Holotype, male: “Holotypus / Congo belge P.N.G. Miss. H. De Saeger II/hd/17, 13-X-1951 Réc. H. De Saeger. 2595 / Guignot det., 1957*Laccophilus turbatus* sp. n. Holotype” (MRAC). – Paratypes: “Congo Belge P.N.G. Miss. H. De Saeger/13-X-1951 Réc. H. De Saeger. 2595 / female / Paratype” (1 ex. IRSNB, 4 exs. MNHN); same data but “II/fc/14, 17.7. 1952, 3608” (1 ex. NHMB); same data but “II/gd/14s, 25.8. 1952, 3984” (1 ex. NHMB).

##### Additional material studied

(50 exs.): **Sudan**: “S. Sudan stream from hot springs, Nyangwara 30,5E, 4,39N, 29.1. 1954 JJOC.” (2 exs. AMGS). – **Liberia**: “Suakoko 18.3. 1952 / Blickenstaff Light trap” (1 ex. USNM, 1 ex. MZH); “Suakoko 20.2. 1952 Blickenstaff” (1 ex. USNM). – **Nigeria**: “R. Kaduna 4.5 mi. from Jos 13.4.1963 JOC.” (4 exs. AMGS);“R. Kaduna rd. 13.5 mi. from Jos 13.IV. 1963 JOC.” (1 ex. AMGS); “Zaria 1969 Brancucci” (1 ex, CSR); “stream Kaduna-Zaria rd. 4.IV. 1963 JOC.” (1 ex. AMGS); “river, rd. to Enugo about 79 mi. from Makurdi 24.4. 1963” (1 ex. AMGS); “stream 64 mi. from Bida on Jebba rd.12.4. 1963 JOC.” (2 exs. AMGS); “Kontagora pools in dry stream 3.IV. 1963 JOC.” (2 exs. AMGS). – **Zaire**: Same data as paratype, but not labelled as belonging to type material (1 ex. IRSNB); “PNG Miss. H. De Saeger II/fd/12, 10.3. 1952, 3180” (2 exs. MRAC, 1 ex. MZH); same but “II/fd/13, 5.5. 1952, 3421” (3 exs. MRAC); same but “II/fd/14s, 3.4. 1952, 3278” (6 exs. MRAC, 2 exs. MZH; habitus in Fig. 430); same but “II/fc/14, 27.6. 1952, 3717” (1 ex. MRAC); “Ht Zaire Umg. Doruma 18.4-10.5. 1986 Wewalka” (2 exs. CGW, 1 ex. MZH). – **Namibia**: “Kavango, Popa Falls 18°07'S-21°35'E, lux 26.2.-3.3. 1992 Uhlig leg.” (10 exs. ZMHB, 2 exx. MZH, 1 ex. NMNW; habitus in Fig. 431); “Kavango, Mahango Game Res., Piknik Site 24.11. 1993 lux 18°13'S-21°45'E Uhlig leg.” (2 exs. ZMHB). – **Botswana**: “Moremi Res. 6.10. 1982 Bilardo / *Laccophilus concisus* Guignot Rocchi det. 1985” (1 ex. CSR).

**Comments on synonymy**: Omer-Cooper (1965) listed *Laccophilus turbatus* as a possible synonym to *Laccophilus concisus*, a species, here associated with *Laccophilus remex* (possible species complex). The latest citation (Nilsson 2015) maintains the opinion of this synonymy. After comparison of holotypes of the two species we prefer to keep the two species separate and accordingly, *Laccophilus turbatus* is here raised back to the level of a valid species because of diagnostic differences in shape of penis.

##### Diagnosis.

*Laccophilus turbatus* is close to *Laccophilus remex*, but smaller (length of body less than 4 mm). The shape of penis also resembles that of *Laccophilus remex* but in *Laccophilus turbatus* penis is more slender and in lateral view external outline somewhat angled while robust and more evenly curved in *Laccophilus remex*.

##### Description.

Body length 3.5–3.9 mm, width 1.8–2.2 mm. Pale ferrugineous, elytra with dense, dark ferrugineous irrorations, which are slightly sparser at base of elytra. Elytral colour pattern exhibits some variation (Figs 430–431).

Head: Pale ferrugineous. Rather shiny. Reticulation fine, double. Larger meshes contain often 4–6 smaller meshes. Almost impunctate, except at eyes; here with a few scattered, fine punctures. Area with punctures extended towards middle of head.

Pronotum: Pale ferrugineous; almost unicoloured pale. Mediobasally with narrow ferrugineous area, delimitation of which is vague. Rather shiny, although finely microsculptured. Reticulation double but rather indistinctly so; in part fine meshes indistinct and hardly visible. At margins with fine punctures; discally almost impunctate.

Elytra: Pale ferrugineous with dense ferrugineous to dark ferrugineous irrorations. Basally irrorations in part sparse, forming irregular slightly larger areas without irroration (Figs 430–431). Slightly mat, with fine microsculpture. Reticulation in part double (feature weakly developed). Larger meshes sometimes indistinct and hardly discernible. Extensively impunctate; discally with a very irregular, longitudinal area with fine and sparse punctures discernible. Lateral, pre-apical furrow rather distinct, finely pubescent.

Ventral aspect: Pale ferrugineous to ferrugineous. Almost impunctate. Slightly mat, very finely and in part indistinctly microsculptured. Basal ventrites of abdomen with fine, in part reduced, curved striae. Metacoxal plates with very fine, in part slightly indistinct transversely located, shallow furrows. Prosternal process slender, apex extended, pointed. Apical ventrite (Fig. 83).

Legs: Protarsus rather slender. Pro- and mesotarsus with suckers.

Male genitalia: Penis in lateral aspect quite long, slender; external outline distinctly curved, exhibiting two bends. Extreme apex hooked but outline rounded (Fig. 275).

Female: Apical ventrite (Fig. 84). Pro- and mesotarsus almost as male but lack suckers.

##### Distribution.

Sudan, Liberia, Nigeria, Zaire, Namibia, Botswana (Fig. 550).

##### Collecting circumstances.

Hardly any information is available. On the basis of label data the species has in Sudan been collected in a stream from hot springs. In Namibia and Liberia collected with light.

#### Laccophilus pallescens

Taxon classificationAnimaliaColeopteraDytiscidae

Régimbart, 1903

**Figures 85–95.**
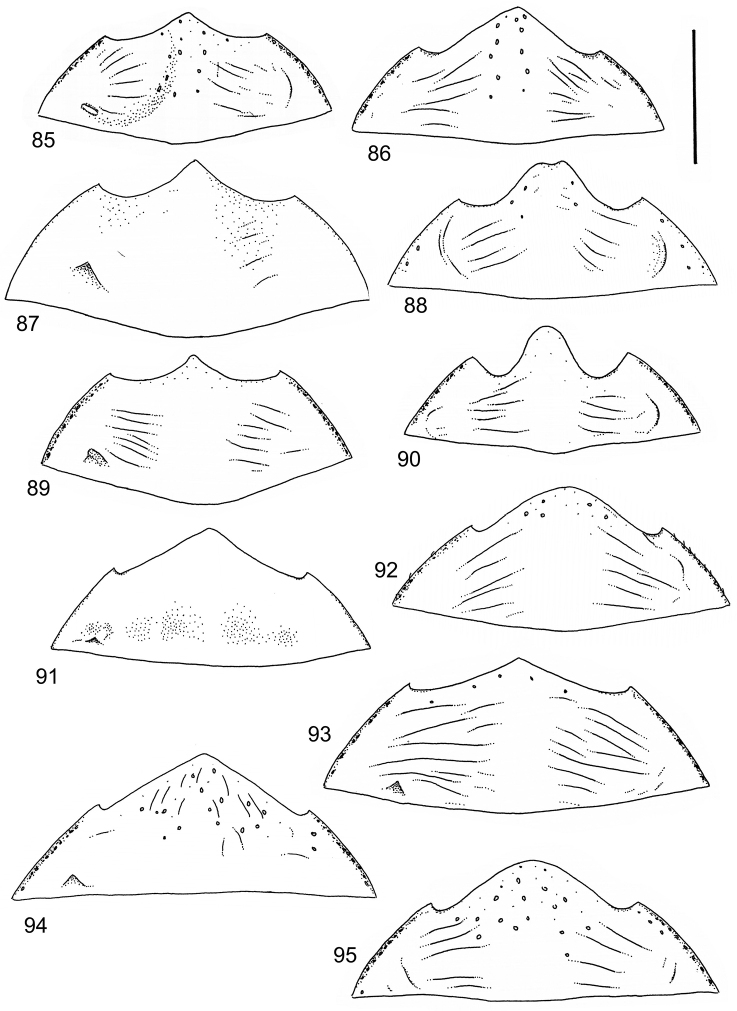
Apical ventrites **85** *Laccophilus pallescens* male, and **86** female **87** *Laccophilus trilineola* male, and **88** female **89** *Laccophilus mediocris* male, and **90** female **91** *Laccophilus epinephes* male, and **92** female **93** *Laccophilus saegeri* male **94** *Laccophilus enigmaticus*, male, and **95** female. Scale bar 0.5 mm.

Figs 85–86 , 276 , 432–433 , 551

Laccophilus pallescens Régimbart 1903: 14 (original description, faunistics); Régimbart 1906: 248 (faunistics); Zimmermann 1920a:23 (catalogue, faunistics); Peschet 1921: 4 (discussion, faunistics); Guignot 1941: 36 (description, discussion); Guignot 1943: 99 (faunistics, discussion); Guignot 1946c: 269, 272, 273, 274, 275, 278, 312, 316 (description, faunistics, discussion); Guignot 1952c: 521 (faunistics); Omer-Cooper 1958b: 37, 48, 49, 50 (discussion, description, faunistics, biology); Legros 1958: 211 (faunistics); Guignot 1959a: 557, 561, 562, 564, 568 (description, discussion, faunistics); Omer-Cooper 1965: 77, 86 (description, faunistics); Bertrand and Legros 1971: 244, 247 (faunistics, biology); Bilardo and Pederzani 1978: 119 (faunistics, description); Medler 1980: 155 (faunistics, list); Pederzani 1988: 107 (faunistics, biology); Rocchi 1990: 442 (faunistics); Rocchi 1991: 86 (faunistics, list); Nilsson 2001: 248 (catalogue, faunistics); Pederzani and Reintjes 2002: 40 (faunistics); Wewalka 2004: 471 (description, faunistics); van Vondel 2005: 131 (faunistics, biology); Pederzani and Rocchi 2009: 95 (faunistics list); Hájek and Reiter 2014: 94 (faunistics, biology); Nilsson 2015: 215 (catalogue, faunistics).

##### Type locality.

Madagascar: Pays Androy.

##### Type material studied

(11 exs.). *Laccophilus pallescens*: Lectotype (by present designation): male: “Madag. Sud Pays Androy C. Alluaud / *pallescens* Rég. types”(MNHN; mounted on same pin on separate label above label with two paralectotypes). – Paralectotypes: Same data as lectotype (2 exs. MNHN). – “Madag. B. Antongil / Museum Paris coll. Maurice Régimbart” (1 ex. MNHN); “S. Baie Antongil / Museum Paris coll. Maurice Régimbart” (1 ex. MNHN); “Madagascar Diego Suarez 10 Ch. Alluaud 1893 / *pallescens* Rég. sp. n. / Museum Paris coll. Maurice Régimbart” (1 ex. MNHN); “Madagascar Sud Pays Androy Nord Alluaud 1900, 34 / Museum Paris 1945 Coll. R. Peschet / *Laccophilus pallescens* Rég.” (1 ex. MNHN);“Madagascar Sud Pays Androy Nord Alluaud 1900, 34” (1 ex. MNHN). Additional three specimens are labelled “Baie de Kavirondo”, but Kavirondo is separately (later and incorrectly) written on the label. When the specimens are mounted on exactly similar labels as those regarded as type material our conclusion is that they also can have this status (3 exs. MNHN). The pin is provided with a label bearing the handwritten text “should be Antongil”.

##### Additional material studied

(234 exs.). **Mali**: “Mopti, Niger Riv. 21.2. 2000, 14°30'N, 4°12'W, Komarek & Mayer leg. / *Laccophilus pallescens* Reg. det. Wewalka 2001 (1 ex. NMW). – **Sudan**: “L. Shambe 21.1. 1954 JJOC.” (1 ex. AMGS); “S. Sudan, stream from hot springs, Nyangwara 30,5E 4,39N 29.1. 1954 JJOC.” (1 ex. AMGS). – **Ethiopia**: “NO Afr. Galla Erlanger / 13.4. 1901” (1 ex. NHMB). – **Ivory Coast**: “Divo 28.11.1963” (1 ex. MRAC); “Comoé N.Pk., N8,5° W 3,5° Reintjes leg. / 4.2. 1999 Kongo Riv. / *Laccophilus pallescens* Régb. det. Pederzani” (2 exs. NMW); “Parc Nat. Comoé, gen. 2000 Moretto” (1 ex. CSR); “Foro-Foro, ad lucem, Duviard leg.” (1 ex. MZH). – **Ghana**: “Ashanti Reg. Kwadaso, agric. st. 6.42N-1.39W / light trap 26.2. 1969 Endrödy-Younga leg.”(2 exs. TMSA, 1 ex. MZH); “V.F. Eastop/Tafo, light 8.5. 1957” (1 ex. BMNH); “N. Reg., Nyankpala 183 m, N9°25’, W1°00’ Endrödy-Younga / shore washing 10.2. 1970” (1 ex. CGW); “Kumasi 18.5. 67 Endrödy-Younga / *Laccophilus pallescens* Rég. det. Wewalka 76” (1 ex. MHNG); same data but ”12.6. 67” (1 ex. MHNG). – **Benin**: Dep. Atlantique, Allada nr Niaouli (village) 6.2. 2006 leg. Goergen et al/06°44'31,7"N; 02°07'55,6"E, ca. 70 m asl, slowly running stream” (1 ex. NMW); “Dep. Atlantique, Allada Avoutè (village) 31.1. 2006 leg. Goergen et al/06°39'54,9"N; 02°09'34,1"E, ca. 25 m asl, small ponds” (1 ex. NMW); “Dep. Zou, Zogbodomè Lokoli (forest), Hlanzoun Riv. 6.2. 2006 Goergen et al. leg. / 07°03'N, 02°15'E muddy stream” (1 ex. NMW). – **Nigeria**: “Detritus pond 45 mi. from Jos on Bauchi rd. 9.4. 1963 JOC.” (1 ex. AMGS); “Stream 3,5 mi. from Oyo 25.3. 1968” (3 exs. AMGS); “Stream 86 mi. from Makurdi on Jos rd. 25.4. 1963” (2 exs. AMGS); “Stream, Kaduna-Zaria, rd. 4.4. 1963 JOC.” (1 ex. AMGS); “Zaria 1969 Brancucci” (1 ex. NHMB); “Trib. of R. Gagere en rte Zaria-Katsina 5.4. 1963 JOC.” (1 ex. AMGS); “Little stream Oyo-Ibadan 25.3. 1963 JOC.” (1 ex. AMGS); “Lagos Colony Iseri 26-27.3. 1949 Malkin / stream, deep slimy mud with sand over” (1 ex, BMNH); “Ondo Prov. h’way 15 mi W of Owo 29.1. 1949 Malkin / Muddy pool in forest, dead leaves” (1 ex. BMNH); “Ile-Ife 10.3. 1969 Medler” (1 ex. USNM); “New Calabar River nr Port Harcourt 13.1. 1989 Umeozor leg.” (1 ex. USNM). – **Cameroon**: “Minkama 15.4. 1970” (1 ex. NHMB). – **Central African Republic**: “Bozo 21.5. 1981 / Degallier” (1 ex. NHMB); “Bozo lum. 11. 1981 / Degallier” (1 ex. NHMB). – **Zaire**: “Holotypus / Musée du Congo / K 300 de Kindu 9-V-1911 L. Burgeon / R DET´1621 T / *Laccophilus burgeoni* Gschw. det. Gschwendt.” (1 ex. MRAC; not type material, see. *Laccophilus burgeoni*; habitus in Fig. 433); “PNA 13.7. 1957 Vanschuytbroeck/Secteur Nord Riv. Semliki 690 m” (1 ex. MRAC); “PNA 26.7. 1957 Vanschuytbroeck/Secteur Nord, marais près riv. Semliki 690 m” (1 ex. MRAC, 1 ex. MZH); “PNA 14.7. 1957 Vanschuytbroeck/Secteur Nord, marais bordure Riv. Semliki 690 m” (1 ex. MRAC); “Katanga Kakyelo 1-9.11. 1931 G.F. de Witte / *Laccophilus pallescens* Régb. det. Wewalka 1979” (1 ex. OLML). – **Kenya**: “Lambwe Valley, on light 11.6. 1974 van Etten” (1 ex. RMNH); “S’Afri Kenya, Dian Beach 5. 1957 Krauss” (1 ex. BMNH); “Maji Ya Chumwi, Mombasa 28.12. 1969 Brown” (1 ex. BMNH); “Malindi 20 km Sud strada per Kilifi 16.7. 68 Pederzani” (1 ex. AMGS); “Kombeni Riv., Mazeras, Kilifi distr. 15.9. 1976 Holmen” (1 ex. ZMUC); “Manjewa R., Mariakani, Kilifi/Kwale distr. 16.9. 1976 Holmen” (1 ex. ZMUC);“Pond NE of Mariakani, Kilifi distr. 16.9. 1976 Holmen” (1 ex. ZMUC); “Dam at Kaloleni Mission, Kilifi distr. 15.9. 1976 Holmen” (3 exs. ZMUC, 1 ex. MZH); “Pond W of Kinango, Kwale distr. 19.9.1976 Holmen” (3 exs. ZMUC, 2 exs. MZH); “Maji ya Chumwi River, Kwale distr. 16.9.1976 Holmen” (2 exs. ZMUC); “Shambini, dam, Kwale distr. 18.9. 1976 Holmen leg.”(1 ex. ZMUC); “Stream W of Kwale district 19.9. 1976 Holmen” (1 ex.ZMUC); “Shimba Hills 10.12. 1989 Jäch leg.” (1 ex. NMW, 1 ex. MZH); “Voi 11. 1997 Snizek / *Laccophilus pallescens* Régb. det. Rocchi 2001” (2 exs. CSR); *“Laccophilus flaveolus* Baie de Kavirondo Lac Victoria Nyanza Alluaud IX-X. 1903” (1 ex. IRSNB; paralectotype of *Laccophilus flaveolus* Régimbart). – **Tanzania**: “Tang. Terr. Ukerewe I. Father Conrad” (1 ex. BMNH, 1 ex. MZH); “SW Tanganyika: Mpanda (dans ruisseau) 6. 1960 Leleup” (1 ex. MRAC); “Zanzibar, stream Mangapwani rd. Sept. 1955 JOC.” (2 exs. AMGS); “Stream Mangapwani rd. 13 Sept. 1955 JOC.” (2 exs. AMGS); “Zanzibar Sept. 1955” (1 ex. AMGS); “Mtawalani, springs, Tanga district 23.9. 1976 Holmen“ (1 ex. MZUC); “Streams S of Hehongo, Tanga distr. 22.9. 1976 Holmen leg.” (1 ex. MNHN); “Goo, small stream, Korogwe distr. 24.9. 1976 M.H.” (1 ex. MZH); “Morogoro Dec. 1909 Schoenheit” (1 ex. ZMHB); “Mahenge Scarp Forest S08.37.10,6,E36.42.46,3, 562 m, 24.4.2011 light trap Smith & Takano” (1 ex. BMNH). – **Zambia**: “Mountain stream crossing the road Kafue-Chirundu 9.8. 1986 Pederzani / *Laccophilus pallescens* Régb det. Rocchi 1988” (2 exs. CSR); “Livingstone env. 6.11. 2006 Z. Jindra leg.” (1 ex. NMPC). – **Malawi**: “Balaka env. 19-20.7. 2001 J. Bezdek leg.” (1 ex. NMPC, 1 ex. MZH); “Balaka env. 19.12. 2002, 180 km SE Lilongwe, Kantner” (1 ex. NHMB); “Mulanje Mts env. 22-28.12. 2001 Kantner” (1 ex. NHMB); “Dedza env. 16.7. 2001 J. Bezdek leg.” (1 ex. NMPC); “Nyasaland Njakwa Distr. 18. Oct. 1948 JOC.” (6 exs. AMGS); “River nr Portuguese border nr Mwanza 9.11. 1948“ (1 ex. AMGS). – **Zimbabwe**: “Wankie Game Res. 4. Sept. 1948 JOC.” (2 exs. AMGS); same data but “5. Sept. 1948” (3 exs. AMGS); “5 mi SE Wankie 7.4. 1968 Spangler” (3 exs. USNM, 2 exs. MZH); “Sinkukwe 30. Dec. 1948 JOC.” (1 ex. AMGS); “Kariba env. 20.12. 1998 Kantner” (1 ex. NHMB, 1 ex. MZH). – **South Africa**: “Transvaal, Nerina Nat. Res. 23.19S-29.47E / 21.12. 1974, from rock pool, leg. Breytenbach” (1 ex. TMSA); “Transvaal Nelspruit 17.5. 1955 C. Frank” (2 exs. AMGS); “Transvaal Kruger Park 1.7. 1960” (1 ex. AMGS); “Kruger Nat. Pk, Letaba Riv. bel. dam 23.46S-31.30E / 1.3. 1995, shorewashing, Endrödy-Younga leg.” (2 exs. TMSA); “Kruger Nat. Pk, Kruger Gate-Skukuza c. 24.59S-31.32E/20.2. 1995 air plancton, Endrödy-Younga leg.” (1 ex. TMSA); “Kruger Nat. Pk, Skukuza Res. Camp 24.59S-31.36E / 25.2.1995 UV light & trap Endrödy-Younga leg.” (1 ex. TMSA); “Kruger Nat. Pk, Skukuza 12 km S, 25.04S-31.37E / 6.3. 1996 UV light Endrödy-Younga leg.” (3 exs. TMSA, 2 exs. MZH); “Kruger Nat. Pk, Pafuri Res. Camp 22.25S-31.12E / 30.1. 1994 UV light & trap leg. Endrödy-Younga” (1 ex. TMSA); “Trsvl, KNP, small waterhole nr Police Picket 30.6. 1960” (1 ex. AMGS); “Zululd, Ndumu, Banzi, fresh wat. pan 26.53S-32.16E / 16.2. 1989, shorewashing, Endrödy & Klimaszew” (1 ex. TMSA); “Natal, roadside puddles, ca 2 km S Mbazawana to Hluhluwe nr Sodwana 5.3. 1997 Turner” (5 exs. CCT, 1 ex. MZH); “Magudu 16.8. 1915 C.J.S.”(1 ex. TMSA); “Tug 77.Q39 (= Station 19 at Mandini, Tugela River 25.7. 51)” (1 ex. AMGS). – **Madagascar**: “Pays Androy, Nord, Alluaud 1900” (1 ex. MNHN); “Ankarana 25.11. 2004, Lat 12.9261, Lon 49.0952 coll. Balke / DNA voucher BMNH <670683> MSL008:D05 / *Laccophilus pallescens* Régb. det. Bergsten” (1 ex, NHRS); “Toli, Morondava: Kirindy, Kirindy Forest, pond, N:-20,072; E: 44,671, 38 m Isambert et al. / DNA Voucher BMNH <830769> MSL 399: H6 / *Laccophilus pallescens* det. J. Bergsten” (1 ex. NHRS); Toli, Menarandra, Menarandra R. pool, N: -24,718, E: 45,047, 227 m, 18.5. 2006 Bergsten et al. / BMNH(E) <794201> <794202>, <794203>, DNA voucher / *Laccophilus pallescens* Régb. det. Bergsten” (3 exs., NHRS); Toli, Taolanaro, Ft Dauphin, Ft Dauphin: Pond N:-25,021, E 46.973: 11 m, 6.5. 2007 Ranarilalalitiana et al. / DNA Voucher BMNH <830771> MSL 399: H8 / *Laccophilus pallescens* det. J. Bergsten” (1 ex. NHRS); Toli, Fort Dauphin, pond in Ft Dauphin P62, 6.4. 2007 N-25,02119, E46,97319, 11 m, Ranarilalatiana leg.” (1 ex. NHRS); “Ankarana; Lat -12.9215, Lon 49.0866, coll. Balke / DNA Voucher BMNH <675013> MSL045:B12 / *Laccophilus pallescens* det. J. Bergsten” (1 ex. NHRS); “Toli, Menarandra Menarandra R 40 km from Ampanihy pools beside a river close to village, algae in pools and sandy bottom with some wood 18.5. 2006, N-24°43.104, E45°2.859, 227 m Bergsten et al.” (2 exs. NHRS); “Toliara Menabe, Kirindy RS, S20.07655, E044.67532, 57 m.a.o., 12.12. 2009 water net, field, Bergsten et al.” (5 exs. NHRS); same data, add “000000476 NHRS-JLKB” (1 ex. NHRS); same data but “S20.07476, E044.67075 / 000000469 NHRS-JLKB” (1 ex. NHRS); same data but “S20.07641, E044.67478, 65 m.a.o., 11.12. 2009” (7 exs. NHRS); same data, add “000000472 NHRS-JLKB” (1 ex. NHRS); “Toliara Menabe, Menabe RS, S19.92773, E045.52253, 102 m.a.o., 10.12. 2009, water net, field, Bergsten et al.” (1 ex. NHRS); “Mahajanga Melaky, betw. Antsalova-Maintirano, S18.30233, E044.18071, 37 m.a.o. 18.12. 2009, water net, field Bergsten et al.” (11 exs. NHRS); same data, add: “000000477 NHRS-JLKB” (1 ex. NHRS); “Mahajanga Melaky, Tsingy de Bemaraha NP, S18.75595, E044.71245, 80 m.a.o., 17.12. 2009 water net, field Bergsten et al.” (36 exs. NHRS); same data, add “000000473 NHRS-JLKB” (1 ex. NHRS); same but “18.75724 E044.71239, 72 m.a.o. 17.12. 2009” (13 exs. NHRS); same data, add “000000475 NHRS-JLKB” (1 ex. NHRS); “Mahajanga Melaky, btw. Morafenobe-Ambohijanahary S18.19091, E045.19986, 290 m.a.o. 19.12. 2009 water net, field Bergsten et al.” (1 ex. NHRS); same data, add “000000474 NHRS-JLKB” (1 ex. NHRS); “Manakambahiny / Coll. R. Peschet” (1 ex. MNHN); “Antakotako II, 1936” (2 exs. MNHN); “Maroansetra 12. 1946 Vadon” (1 ex. MNHN; habitus in Fig. 432); “Andjamangirana (Majunga) 19.10. 2001 / Stream in dry forest, rice field area (road to Tsarantanana) 220 m a.s.l., 30.8°C, 0.008 mS/cm / Gerecke & Goldsmith leg.” (1 ex. BMNH); “Matitanana Bas. Loc. Nato, Matitanana Riv. 47°49'32” E, 22°18'36"S, alt. 43 m, 19.6.1995 Andriamihaja” (1 ex. NHRS); “Manajary Bas., 1 km avant Kianjavato (Amont) Fotobohitra Riv. 47°51'38"E, 21°22'36"S, 3.12.1995 Pilaka” (1 ex. NHRS); “Tamatave (Toamasina) Park Ivoloina Pfütze an Strasse im Wald 21.11. 2000 Dolin” (4 exs. NHRS).

##### Specimen with unclear labelling.

**Swaziland**: “Eranchi 5-10.1. 1955 A.L. Capener” (1 ex. AMGS). The specimen bears a second label “Swedish South Africa Expedition 1950-1951 Brinck-Rudebeck” which makes the origin obscure.

##### Diagnosis.

*Laccophilus pallescens*, being externally a somewhat variable species, is characterized by shape of penis; in lateral view, external outline of penis with two somewhat vague flexures/bends. Penis shape resembles *Laccophilus turbatus* but extreme apex much smaller.

##### Description.

Body length 3.4–3.8 mm, width 1.7–2.0 mm. Dorsal, colour pattern of body somewhat variable but similar ground plan of it, is exhibited in all studied specimens (Figs 432–433).

Head: Pale ferrugineous; posteriorly often, with two minute, somewhat vague, dark spots. Almost impunctate except at eyes; with irregular, fine punctures. Areas with punctures expand towards middle but puncture-areas are not connected. Shiny to rather shiny although finely microsculptured. Reticulation double but small meshes in part reduced, and indistinct. When discernible, large meshes contain 4–8 fine meshes.

Pronotum: Pale ferrugineous; no distinct colour pattern exhibited. Rather shiny, finely microsculptured. Reticulation double but fine meshes in large extent reduced or absent. Laterally, fine meshes discernible. Almost impunctate except laterally and frontally.

Elytra: Pale ferrugineous, with dense, dark ferrugineous irrorations. At base and slightly posterior to middle irrorations sparser (paler, transverse areas formed). Base sometimes almost lacking dark colour pattern. Irrorations of elytra comparatively coarse (Figs 432–433). Rather shiny, although microsculptured; reticulation extensively double. Large meshes contain 3–6 fine meshes. Fine meshes in part reduced, indistinct. Discal, dorsolateral and lateral rows are formed by very fine, scattered punctures.

Ventral aspect: Ferrugineous to pale ferrugineous, abdomen slightly paler; pale ferrugineous. Almost impunctate. Rather shiny and slightly mat, very finely microsculptured; reticulation in part reduced or absent. Abdomen with very fine curved striae. Metacoxal plates in anterior half with very fine, shallow furrows (furrows in part rather indistinct). Prosternal process rather slender, apex somewhat extended and pointed. Apical ventrite asymmetric, with single minute knob located laterally (Fig. 85).

Legs: Pro- and mesotarsus somewhat enlarged, slightly extended; provided with distinct suckers.

Male genitalia: Penis in lateral aspect, with external outline bended twice; extreme apex finely hooked with outline rounded (Fig. 276).

Female: Apical ventrite not distinctly asymmetric; lacks lateral knob (Fig. 86). Pro- and mesotarsus slender.

##### Distribution.

Mali, Sudan, Ethiopia, Ivory Coast, Ghana, Benin, Nigeria, Cameroon, Central African Republic, Zaire, Kenya, Tanzania, Zambia, Malawi, Zimbabwe, South Africa, Madagascar (Fig. 551). Guignot (1943) gives Burkina Faso and (1946c) Uganda, Reintjes (2004) Guinea and Botswana and Wewalka (2004) Republic of Congo. Single record from Swaziland is considered unclear.

##### Collecting circumstances.

Quite scarcely documented. Sampled in brooklet and in stagnant remain of brooklet and in a rain pool (van Vondel 2005). Label data give that the species has been collected from a stream from hot springs in Sudan. Additionally labels tell that the species was collected from slowly running stream, from a stream in dry forest and from a rock pool. Also collected at light or with light traps. Some information on biology can also be gained from Omer-Cooper (1958b). In Socotra Island, Yemen, the species was mostly collected in larger pools of drying up streams in wadis (Hájek and Reiter 2014).

#### Laccophilus trilineola

Taxon classificationAnimaliaColeopteraDytiscidae

Régimbart, 1889

Figs 87–88 , 277 , 434 , 552

Laccophilus trilineola Régimbart 1889: 52 (original description, faunistics); Régimbart 1895: 132 (description, faunistics); Zimmermann 1920a: 27 (catalogue, faunistics); Guignot 1959a: 577 (description, faunistics); Nilsson 2001: 252 (catalogue, faunistics); Nilsson 2015: 218 (catalogue, faunistics).
Laccophilus simulator Omer-Cooper 1958b: 37, 50, 53 (original description, faunistics, biology); Nilsson 2001: 251 (catalogue, faunistics); Pederzani and Reintjes 2002: 40 (faunistics); Nilsson 2015: 218 (catalogue, faunistics). **New synonym.**

##### Type localities.

*Laccophilus trilineola*: Angola: Humpata.

*Laccophilus simulator*: Malawi: Dowa.

##### Type material studied

(5 exs.) *Laccophilus trilineola*. Holotype: female: “P.J. vd Kellen, Humpata, Afr. trop. / *Laccophilus trilineola* sp. n. type Régb. / *trilineola* sp. n. Régimb. / type” (RMNH).

*Laccophilus simulator*: Holotype, male: “Type / Nyasaland, stream 6 miles N of R. Mtiti 1.10. 1948 / *Laccophilus simulator* sp. n. Det. J. Omer-Cooper” (BMNH; two specimens on same pin but as holotype is clearly stated as male it is evident that specimen upper on pin must be the holotype). – Paratypes: Same data as holotype and pinned with it (1 ex. female BMNH); “Nyasaland stream, 6 miles N R. Mtiti 2.10. 1948 / Paratype / Paratype / *Laccophilus simulator* O-C. J. Omer-Cooper” (1 ex. IRSNB). One additional paratype labelled “Nyasaland stream? N of R. Mtiti 10. 1948 / *Laccophilus simulator* sp. n. J. Omer-Cooper det” belongs to *Laccophilus adspersus* Boheman.

##### Additional material studied

(1 ex.). **Zaire**: “Dilolo VIII-IX-1931 G.F. de Witte” (1 ex. OLML; habitus in Fig. 434).

##### Comments on synonymy.

The holotypes of *Laccophilus trilineola* and *Laccophilus simulator* have been examined and compared. The holotype of *Laccophilus trilineola* is a female and it is a unique specimen which makes study of male genitalia impossible. Both taxa, however, exhibit peculiar colour pattern on elytra (three longitudinal pale areas at base), which lack in other African species. Moreover, shape of female apical ventrite is also characteristic and similar in both taxa. Accordingly, the two taxa are considered conspecific. *Laccophilus trilineola*, being the older name is the valid name of this species.

##### Diagnosis.

*Laccophilus trilineola* is characterized by elytral colour pattern, peculiar female apical ventrite and uniquely shaped penis; penis in lateral aspect quite long, medium robust and medially bent; extreme apex peculiar with external end, expanded to form a sharp extension.

##### Description.

Body length 4.0–4.5 mm, width 2.1–2.5 mm. Dorsal, colour pattern as in Fig. 434.

Head: Pale ferrugineous, posteriorly only slightly darker. Submat, finely microsculptured. Reticulation double. Large meshes in part indistinct. When discernible they may contain 2–6 small meshes. Impunctate, except at eyes where irregular, fine punctures discernible. Areas of punctures extend towards middle of head.

Pronotum: Pale ferrugineous. Medially slightly darker than laterally. Rather shiny to submat although finely microsculptured. Reticulation double: Large meshes contain 2–6 small meshes. Finer reticulation laterally, in part indistinct. At margins with scattered very fine punctures; at posterior margin punctures almost absent; a few very fine punctures may, however, be discerned.

Elytra: Pale ferrugineous, with dense ferrugineous to dark ferrugineous irrorations. At base with three, slightly irregular, longitudinally extended, pale areas (Fig. 434). Slightly mat, finely microsculptured. Reticulation at least in frontal part double; posteriorly double reticulation is indistinct or absent. Scattered, very fine punctures may be discerned. In part punctures absent.

Ventral aspect: Pale ferrugineous to ferrugineous; no distinct colour pattern formed. Rather shiny to submat, finely and partly indistinctly microsculptured. Finely and sparsely striated; distinct striae only discerned on two basal ventrites. Almost impunctate. Prosternal process slender; apex extended and pointed. Apical ventrite asymmetric with sharp knob on one side (Fig. 87).

Legs: Protarsus slightly enlarged; provided with suckers.

Male genitalia: Penis in lateral aspect quite long, medium robust and external outline medially bent; extreme apex peculiar with external end, expanded to a sharp extension (Fig. 277).

Female: Protarsus slender. Apical ventrite (Fig. 88).

##### Distribution.

Zaire, Angola, Malawi (Fig. 552).

##### Collecting circumstances.

According to Omer-Cooper (1958b) the species has been collected in a stream with some pools full of decaying matter. Furthermore it was collected in a sluggish stream with patches of reeds and water weeds, and the bottom with fine algal growth. Also found in a clear river with water lilies, reed beds and swamp patches.

#### Laccophilus mediocris

Taxon classificationAnimaliaColeopteraDytiscidae

Guignot, 1952

Figs 89–90 , 278–279 , 435 , 552

Laccophilus mediocris Guignot 1952d: 4 (original description, faunistics); Guignot 1956a: 88 (faunistics); Omer-Cooper 1958b: 51 (discussion); Guignot 1959a: 562, 567, 568 (description, faunistics); Bilardo and Pederzani 1978: 119 (faunistics, description); Medler 1980: 155 (faunistics, catalogue); Pederzani and Rocchi 1982: 72 (faunistics); Nilsson et al. 1995: 505 (faunistics); Nilsson 2001: 246 (catalogue, faunistics); Pederzani and Reintjes 2002: 40 (faunistics); Reintjes 2000: 67 (faunistics); Bilardo and Rocchi 2008: 211, 236 (faunistics, biology); Bilardo and Rocchi 2013: 141 (faunistics); Nilsson 2015: 214 (catalogue, faunistics).
Laccophilus meii Rocchi 2000: 24 (original description, faunistics, discussion); Nilsson 2001: 246 (catalogue, faunistics); Nilsson 2015: 214 (catalogue, faunistics). **New synonym.**

##### Type localities.

*Laccophilus mediocris*: Ivory Coast: Toumodi.

*Laccophilus meii*: Republic of Guinea: Faranah, Sidakoro.

##### Type material studied

(8 exs.) *Laccophilus mediocris*. Holotype: male: “Cote d’Ivoire Toumodi /male symbol / Wehnckei Shp det. Gschwendt. / Type / *Laccophilus mediocris* Guign. Type, male” (MNHN). – Paratypes: “Cote d’Ivoire Toumodi / Museum Paris 12-1930-IV-1931 Ch. Alluaud & P.A. Chappuis / female symbol / Paratype” (3 exs. MNHN; habitus in Fig. 435); Mali: “Soudan Francais Bamako / Museum Paris 12-1930-IV-1931 Ch. Alluaud & P.A. Chappuis / male symbol / Paratype” (1 ex. MNHN).

*Laccophilus meii*: Holotype: male: “République de Guinée, PNHN 10 10o15'08"N, 10o28'20"W Faranah, Sidakoro, mare, 12.1. 1996 leg. M. Mei” (in acqua con fondo fangoso e molto detrito) / Holotypus / *Laccophilus meii* sp. n. det. S. Rocchi 1997” (MZUL). – Paratypes, female with same data as holotype but labelled “Paratypus” (1 ex. MZUL); male: “République de Guinée, PNHN9, 10o27'50"N, 10o26'26"W, Faranah, Somorya F. Koffin 18.1. 1996 leg. M. Mei / Paratypus / *Laccophilus meii* sp. n. det. S. Rocchi 1997” (1 ex. CSR).

##### Additional material studied

(22 exs.). **Ivory-Coast**: “Comoé NP, N8,5°-W3,5° Reintjes / 20.2. 1999” (1 ex. NMW); same data but “5.1. 1999” (3 exs. NMW); “Touba, a la lumière 4. 2002 Moretto / *Laccophilus mediocris* det. Rocchi 2002” (1 ex. CSR). – **Ghana**: “Ashanti Reg. Kwadaso, agric. st., 6.42N-1.39W / 26.2. 1969 light trap Endrödy-Younga” (1 ex. TMSA, 1 ex. MZH); “N Reg., Damongo game res. 9.04N-1.48W / at light 12.8. 1971 Endrödy-Younga” (1 ex. TMSA). – **Nigeria**: “Kontagora 3.IV. 1963 JOC.” (3 exs. AMGS); “Stream, road Kaduna-Kontagora 3.IV. 1963 JOC.” (1 ex. AMGS); “River, Enugo rd about 47 mi. from Makurdi 24.4. 1963 JOC.” (3 exs. AMGS); “Stream Enugo-Makurdi 24.IV. 1963 JOC.” (1 ex. AMGS); “Stream 86 mi. from Makurdi on Jos road 25.IV. 1963” (5 exs. MNHN). – **Congo**: “Ewo 5. 1979 Onore / *Laccophilus mediocris* Guignot det. Pederzani” (1 ex. CSR).

##### Comments on synonymy.

Male holotypes of *Laccophilus mediocris* and *Laccophilus meii* have been studied and compared. Both external and male genital features seem to be identical. Accordingly the two species are regarded conspecific, and *Laccophilus mediocris*, which is the older name, is the valid name of the species. *Laccophilus mediocris* seems to have been overlooked when *Laccophilus meii* was introduced as a new species because no reference to it is given to it in the original description (Rocchi 2000).

##### Diagnosis.

*Laccophilus mediocris* is especially characterized by its peculiar, twisted penis, which is different from all other African *Laccophilus* species. Additionally, apical portion of penis comparatively delicate, narrow and moderately twisted; extreme apex finely hooked.

##### Description.

Body length 3.6–4.1 mm, width 2.0–2.2 mm. Elytra with vague colour pattern; irrorations of elytra somewhat weakly developed and in part vague. In frontal half some vague, pale areas may be discerned (Fig. 435).

Head: Pale ferrugineous. Submat, finely microsculptured; reticulation in part indistinctly double. Large meshes indistinct; when discernible they are only slightly coarser than fine meshes; may include 3–6 fine meshes. At eyes with very fine, scattered punctures. Area of punctures extends a short distance towards centre of head.

Pronotum: Pale ferrugineous; distinct colour pattern lacking. Finely microsculptured and reticulation indistinctly double. Anteriorly and laterally with very fine punctures.

Elytra: Pale ferrugineous, with extensive but rather indistinct, ferrugineous irrorations (Fig. 435). Slightly mat, densely microsculptured. Difference between fine and coarse meshes very small. Rows of punctures hardly visible, almost absent. Discal row of punctures consists of fine, somewhat irregularly located punctures. Dorsolateral and lateral row of punctures only with a few, scattered punctures discernible.

Ventral aspect: Pale ferrugineous; no distinct colour pattern discernible. Almost impunctate. Rather shiny, although very finely, in part indistinctly microsculptured. Semitransverse furrows of metacoxal plates shallow and quite indistinct. Abdomen basally with curved striae. Prosternal process slender and pointed. Apical ventrite with asymmetric (located on one side) knob (Fig. 89).

Legs: Pro- and mesotarsus slightly enlarged, provided with protruding suckers.

Male genitalia: Penis twisted, in lateral aspect with apical portion of penis comparatively delicate, narrow and moderately undulate; extreme apex finely hooked (Figs 274–275).

Female: Apical ventrite almost symmetric lacking lateral knob (Fig. 90). Pro- and mesotarsus narrower than in male.

##### Distribution.

Mali, Guinea, Ivory Coast, Ghana, Nigeria, Congo (Fig. 552). Non-verified country-records are Zaire (Guignot 1956a), Guinea-Bissau (Nilsson et al. 1995) and Gabon (Bilardo and Rocchi 2008).

##### Collecting circumstances.

Rather insufficiently documented. When describing *Laccophilus meii*, Rocchi (2000), however, added some data on the sampling localities: the species was collected in water with a muddy bottom and much debris and moreover, in a pool along river bed of Koffin River.

#### Laccophilus epinephes

Taxon classificationAnimaliaColeopteraDytiscidae

Guignot, 1955

Figs 91–92 , 280–282 , 436 , 553

Laccophilus epinephes Guignot 1955e: 2 (original description, faunistics); Bertrand and Legros 1975: 681 (faunistics, list); Medler 1980: 155 (faunistics, list.); Pederzani and Rocchi 1982: 72 (faunistics); Bilardo and Rocchi 1990:162, 177 (faunistics, biology); Nilsson 2001: 243 (catalogue, faunistics); Bilardo and Rocchi 2002: 174 (list, faunistics); Pederzani and Reintjes 2002: 38 (faunistics); Reintjes 2004: 67 (faunistics); Bilardo and Rocchi 2008: 211, 235 (faunistics, biology); Bilardo and Rocchi 2013: 141 (faunistics, biology); Nilsson 2015: 211 (catalogue, faunistics).
Laccophilus castaneus Guignot 1956b: 220, 221 (original description, faunistics); Nilsson 2001: 241 (catalogue, faunistics); Nilsson 2015: 210 (catalogue, faunistics). **New synonym.**

##### Type localities.

*Laccophilus epinephes*: Zaire: Parc National de la Garamba.

*Laccophilus castaneus*: Senegal: Niokolo Koba, Badi.

##### Type material studied

(15 exs.). *Laccophilus epinephes*: Holotype: male: “Holotypus / Congo belge P.N.G. Miss. H. De Saeger I/a/2, 21-IV-1950 Réc. G. Demoulin / Coll. Mus. Congo (ex. coll. I.P.N.C.B.) / F. Guignot det., 1955 *Laccophilus epinephes* sp. n. Type male symbol” (MRAC). – Paratypes: Same as holotype but “I/b/2, 27.9. 1950, 847 / Paratype” (1 ex. MNHN); “Congo Belge P.N.G. Miss. H. De Saeger II/gd/14s, 25-VIII-52 H. De Saeger. 3984 / Paratype / F. Guignot det., 1958 *Laccophilus epinephes* sp. n.” (5 exs. IRSNB, 1 ex. MNHN); same data but “I/c/2, 17.3. 1950/Paratype” (1 ex. MNHN); same data but “I/a/2-3, 10.7. 1950 Paratype” (1 ex. MNHN); same data but “II/hd/14s, 17-X-1951, 2644” (1 ex. NHMB); same data, but “Utukuru/14, 22-VII-52, 3812” (1 ex. IRSNB, 1 ex. NHMB).

*Laccophilus castaneus*: Holotype: male: “Mission IFAN au Parc National du Niokolo Koba Badi (Sénégal) 15.VIII-25.IX -1955 / Type / F. Guignot det. 1956 *Laccophilus castaneus* sp. n. Type” (MNHN). – Paratypes: females: “Mission IFAN au Parc National du Niokolo Koba/Badi (Sénégal) 15.VIII-25.IX -1955 / female symbol / Paratype” (1 ex. MNHN); “Ouassadou 12.VIII. 55 / Mission IFAN au Parc National du Niokolo Koba / female symbol / Paratype” (1 ex. MNHN).

##### Additional material studied

(47 exs.). **Ivory Coast**: “Nord C. d’I. Ferkessédougou 10-20.5. 1964 Decelle” (1 ex. MRAC; habitus in Fig. 436); “Comoé NP, N8,5°-W3,5° Reintjes 1.5. 1999 / *Laccophilus epinephes* Guignot det. Pederzani” (1 ex. CSR). – **Nigeria**: “River Crossing Erugo rd 80 mi. From Makurdi 24.4. 1963” (1 ex. AMGS); “Stream Kaduna-Zaria rd. 4.IV. 1963 JO-C.” (1 ex. AMGS); “River about 79 mi from Makurdi on Erugo road 24.4. 1963” (1 ex. AMGS); “Pools in dry stream bed, Kontagora 5.IV. 1963 J.O-C.” (6 exs. AMGS); “Niger Prov., Zunguru 18.3. 1949 Malkin / pond, much detritus, reeds” (11 exs. BMNH, 3 exs. MZH); “Ilorin Prov., Ilorin 15-18.2. 1949 Malkin / small clean pond” (1 ex. BMNH). – **Cameroon**: “Tokombere (dint. Maroua) 12.7. 1979 leg. Onore / *Laccophilus epinephus Guignot* det. Rocchi 1981” (5 exs. CSR); “Kamerun int. Satsche 10-14.5. 1909 Riggenbach” (1 ex. ZMHB); “Mokolo pr. Maroua 8-10.7. 1979 Onore / zona pre-Saheliana, pozzangera residuo di ruscello ambiente soleggiato” (1 ex. NHMB). – **Gabon**: “Res. Lopé-Okanda, milieu de savane 1.2.1986 Bilardo / *Laccophilus epinephes* Guig. det. Bilardo” (1 ex. CSR, 2 exs. MSNM). – **Congo**: “Ewo 12.5. 1979 Onore” (1 ex. NHMB); “Parco Nazion. Mbomo 16.5. 1979 Onore” (1 ex. NHMB); “P. K. Rouge 4-1979 Onore / *Laccophilus epinephes* Guign. det. Rocchi 1981” (1 ex. NHMB). – **Zaire**: “Lualaba, Kolwezi riv. Dilolo 1953 Allard” (1 ex. MRAC, 1 ex. MZH); “PNG Mabanga 14, 31.8. 1952 H. De Saeger, 3870” (1 ex. MRAC, 1 ex. MZH); “PNG Ndelele/14s, 1.8.1952 H. De Saeger, 3871” (1 ex. MRAC); “PNG PpK.14/g/14s, 4.4. 1952 H. De Saeger, 3290” (1 ex. MRAC); “PNG II/fd/12, 10.3. 1952 H. De Saeger, 3180” (1 ex. MRAC); “PNG II/gd/14, 30.7. 1952 H. De Saeger, 3857” (1 ex. MRAC).

##### Comments on synonymy.

The male holotypes of *Laccophilus epinephes* and *Laccophilus castaneus* have been studied and compared. No diagnostically decisive features in external appearance of the body or in the male genitalia were detected. Accordingly the two species are considered conspecific. *Laccophilus epinephes*, being the older available name is the valid name of the species.

##### Diagnosis.

*Laccophilus epinephes* is characterized by big sized body and peculiar shape of penis. Penis in lateral aspect quite broad, robust, medially distinctly curved and extreme apex protruding forwards, not distinctly hooked.

##### Description.

Body length 4.2–4.8 mm, width 2.3–2.5 mm. Elytral colour pattern somewhat vague; ferrugineous with dense but somewhat diffuse, dark ferrugineous irrorations, which sometimes can be rather indistinct. Habitus and dorsal colour pattern as in Fig. 436.

Head: Pale ferrugineous; no distinct colour pattern. Rather shiny to slightly mat, although finely reticulated. Reticulation double; large meshes contain 2–5 small meshes. Large meshes only slightly more strongly developed that fine meshes. At eyes and on disc, with fine, irregular punctures.

Pronotum: Pale ferrugineous to ferrugineous; no distinct colour pattern. Rather shiny to slightly mat although finely microsculptured; reticulation double. Large meshes contain 2–6 small meshes (sometimes meshes rather indistinct and weakly developed). At margins, very fine, irregular punctures may be discerned.

Elytra: Pale ferrugineous, with dense and somewhat vague, ferrugineous to dark ferrugineous irrorations (Fig. 436). Rather shiny to slightly mat, finely reticulated. Reticulation double but feature rather weakly developed. Large meshes contain 2–6 smaller meshes. Laterally and posteriorly double reticulation indistinct and mesh-size-classes cannot be distinguished. Discal row of punctures formed by very fine, slightly irregular punctures. Other rows simply indicated by a few, irregular punctures. Lateral, pre-apical furrow shallow, pubescent.

Ventral aspect: Pale ferrugineous to ferrugineous, abdomen generally slightly darker. Rather shiny to submat, finely microsculptured. Almost impunctate. Basal ventrites with fine, in part reduced, curved striae. Apical ventrite lacks distinct knob but surface on one side uneven (knob-rudiment?) (Fig. 91). Apex of prosternal process slender, somewhat extended and pointed. About 10 transversely located, shallow furrows of metacoxal plates clearly discernible in anterior half; in posterior half indistinct, almost absent.

Legs: Pro- and mesotarsus slightly enlarged; provided with suckers.

Male genitalia: Penis in lateral aspect quite broad, robust, medially distinctly curved and tip of penis protruding forwards, not distinctly hooked (Figs 280–282).

Female: Apical ventrite as in Fig. 92. Pro- and mesotarsus quite slender.

##### Distribution.

Senegal, Ivory Coast, Nigeria, Cameroon, Gabon, Congo, Zaire (Fig. 553).

##### Collecting circumstances.

According to label-data the species has been collected from pools in dry stream bed, in a pond, with much detritus and reeds. Some additional information is available in Bilardo and Rocchi (1990 and 2013) where general descriptions are given for collecting localities. The species is listed as a preferring savannah-habitats in Bilardo and Rocchi (2008).

#### Laccophilus saegeri

Taxon classificationAnimaliaColeopteraDytiscidae

Guignot, 1958

Figs 93 , 283–284 , 437 , 438 , 553

Laccophilus saegeri Guignot 1958: 7 (original description, faunistics); Medler 1980: 155 (faunistics, list); Nilsson 2001: 250 (catalogue, faunistics); Pederzani and Reintjes 2002: 40 (faunistics); Nilsson 2015: 217 (catalogue, faunistics).
Laccophilus comoensis Pederzani and Reintjes 2002: 35, 38, 39 (original description, faunistics, biology, list); Reintjes 2004: 66 (faunistics, list); Nilsson 2015: 210 (catalogue, faunistics). **New synonym.**

##### Type localities.

*Laccophilus saegeri*: Zaire: Garamba National Park.

*Laccophilus comoensis*: The Ivory Coast: Comoé Nat. Park.

##### Type material studied

(16 exs.). *Laccophilus saegeri*: Holotype: male: “Holotypus / Congo Belge, P.N.G. Miss. H. De Saeger II/hd/14s, 17-X-1951 Réc. De Saeger, 2644 / Coll. Mus. Congo (ex. coll. I.P.N.C.B.) / Guignot det., 1957 *Laccophilus saegeri* sp. n. Holotype” (MRAC; habitus in Fig. 437). – Paratypes: “Congo Belge, P.N.G. Miss. H. De Saeger II/hd/14s, 17-X-1951 Réc. De Saeger, 2644 / Paratype” (3 exs. IRSNB, 3 exs. MNHN, 2 exs. NHMB); “Congo Belge, P.N.G. Miss. H. De Saeger II/fc/14, 4-VII-1952 H. De Saeger, 3736 / Paratype” (2 exs. IRSNB; one paratype belongs to *Laccophilus remex* species complex); “PNG Miss. H. De Saeger II/gc/13s, 3.9. 1951 H. De Saeger, 2359” (1 ex. MNHN, 1 ex. NHMB).

*Laccophilus comoensis*: Holotype, male: “Cote d’Ivoire Comoé N.P. N8,5, W3,5, leg et det. N. Reintjes / 4.2. 1999 CB6A Comoé River / Holotypus in Quad. Studi Nat. Romagna 16 suppl.: 35-41 / *Laccophilus comoensis* Pederzani & Reintjes, 2002 /*Laccophilus comoensis* Pederzani & Reintjes Holotype” (NMW; habitus in Fig. 438). – Paratypes: “Cote d’Ivoire Comoé N.P. N8,5, W3,5, leg et det. N. Reintjes / 3.1. 1999 ABIA Temporary pond / Paratypus in Quad. Studi Nat. Romagna 16 suppl.: 35-41 / *Laccophilus comoensis* Pederzani & Reintjes, 2002 / *Laccophilus comoensis* Pederzani & Reintjes Paratype” (1 ex. NMW); “Cote d’Ivoire Comoé N.P. N8,5, W3,5, leg et det. N. Reintjes / 26.12. 1998 TBIA Temporary pond/Paratypus in Quad. Studi Nat. Romagna 16 suppl.: 35-41 / *Laccophilus comoensis* Pederzani & Reintjes, 2002 / *Laccophilus comoensis* Pederzani & Reintjes Paratype” (1 ex. NMW).

##### Additional material studied

(2 exs.): **Zaire**: “Katanga, Kansenia -6. 1925 de Witte” (1 ex. CGW); “Jadotville 9. 52” (1 ex. NHMB).

##### Specimen with uncertain determination

(1 ex.). **Tanzania**: “16 km W Iringa, Isimilia-Schlucht 14.8. 1998 M. Wewalka leg.” (1 female ex. CGW).

##### Records which need re-confirmation

(specimens now, not available) (4 exs.). **Nigeria**: “Kontagora 3.IV. 1963 JOC.” (2 exs. AMGS). – **Zambia**: “N. Rhodesia Lusaka” (2 exs. AMGS).

##### Comments on synonymy.

Holotypes, males, of *Laccophilus saegeri* and *Laccophilus comoensis* have been studied and compared. The shape of penis is almost identical. Some minor variation in appearance of elytral colour pattern exists. The difference is, however, superficial and equally great differences are found between the specimens constituting the type series of *Laccophilus saegeri*. Accordingly, it seems clear that the two species are conspecific. Valid name of the species is *Laccophilus saegeri*, being the older available name.

##### Diagnosis.

*Laccophilus saegeri* is characterized by quite large body; quite uniform elytral colour pattern in combination with shape of penis, which exhibits only minor variation. Penis in lateral aspect distinctly curved, extreme apex pointing forwards and not distinctly hooked. Resembles much *Laccophilus epinephes* but penis is less broad and quite slender. *Laccophilus saegeri* resembles in part also of *Laccophilus pulcher*, here located in specgies group 11 (*deceptor*). Similarity between *Laccophilus saegeri* and *Laccophilus pulcher* is especially confined to shape of penis. Colour pattern of elytra is however completely different (*Laccophilus pulcher* has extensive, dark colour on elytra). Similarity in penis shape indicates possibility of closer relationship between these two species – this question needs further study.

##### Description.

Body: Length 4.1–4.7 mm, width 2.2–2.6 mm. Dorsal, colour pattern of body exhibits some variation (Figs 437–438).

Head: Pale ferrugineous. Post-medially, sometimes with two minor, ferrugineous spots. Slightly mat. Finely microsculptured. Double reticulation discernible but rather indistinct; only in part visible. Large meshes, when discernible may contain 3–7 small meshes. Impunctate, except at eyes; with fine, irregular punctures. Areas of fine punctures extend towards middle of head.

Pronotum: Pale ferrugineous. Anteriorly between eyes often narrowly darkened; with a ferrugineous to dark ferrugneous marking. Mediobasally sometimes with two minute, narrow darkened markings. Pronotum sometimes almost unicoloured pale. Rather shiny, although finely microsculptured. Reticulation fine, double and extensively discernible. Large meshes contain 3–7 small meshes. Impunctate, except anteriorly and laterally; with very fine to fine, irregular punctures.

Elytra: Pale ferrugineous. Colour pattern variable but specimens exhibit same ground-plan; consists of irregular, dark ferrugineous irrorations, which are sparser subbasally and slightly posterior to mid of elytra where two transverse, pale, areas are discernible (Figs 437–438). Rather shiny, finely reticulated. Double reticulation discernible, but large meshes of reticulation often rather indistinct. When discernible, large meshes may contain 3-7 small meshes. Impunctate, except for discal row of punctures, formed by fine, irregular punctures. Dorsolateral and lateral rows indicated by a few scattered punctures. Lateral, pre-apical furrow fine, pubescent.

Ventral aspect: Pale ferrugineous to ferrugineous, apically on abdomen and metacoxal processes ferrugineous to dark ferrugineous. Submat to rather shiny, finely reticulated; in part reticulation obliterated. Basal ventrites with fine, curved striae. Almost impunctate. Prosternal process slender, apex slightly extended, pointed. Apical ventrite as in Fig. 92. Metacoxal plates, especially in anterior half with fine, almost transversely located, shallow furrows.

Legs: Protarsus slender; claws slightly curved, equally long. Pro- and mesotarsus with suckers.

Male genitalia: Penis in lateral aspect quite long, distinctly bended and extreme apex expanding forwards, not distinctly hooked (Figs 283–284).

Female: Not studied. No females have been available for study.

##### Distribution.

Ivory Coast, Zaire (Fig. 553). Specimen from Tanzania is a female, the determination of which is uncertain. Specimens from Nigeria and Zambia need to be re-examined but are not now available.

##### Collecting circumstances.

Collected from temporary ponds. See also Reintjes (2002).

#### Laccophilus enigmaticus sp. n.

Taxon classificationAnimaliaColeopteraDytiscidae

http://zoobank.org/8A25143F-4849-495B-AF13-8820977C2934

Figs 94–95 , 285 , 439 , 555

##### Type locality.

Nigeria: Zaria province, Zaria.

##### Type material

(9 exs.): Holotype, male: “Nigeria Zaria Prov., Zaria 5–6.III. 1949 B. Malkin / B. Malkin Coll. BMNH(E) 1956-234” (BMNH). – Paratypes: Same data as holotype (3 exs. BMNH, 1 ex. MZH; habitus in Fig. 439); “Sudan Bahr el Ghazal Wau 19.2. 1963 Linnavuori” (1 ex. MZH); “Sudan (Equatoria) Nzara 22.4. 1986 leg. Wewalka (Z11-13)” (2 exs. CGW, 1 ex. MZH).

##### Diagnosis.

*Laccophilus enigmaticus* is especially characterized by comparatively big sized body, peculiar, uniform elytral colour pattern in combination with the penis, which is different in comparison to all other recognized species in Africa; inner outline curvature medially somewhat enlarged, forming a distinct but smooth expansion of penis. Apex of penis broadly but rather indistinctly hooked.

##### Description.

Body length 4.3–4.6 mm, width 2.3–2.5 mm. Dorsal, colour pattern of body almost uniform, exhibits only minor variation (Fig. 439).

Head: Pale ferrugineous. Slightly mat, microsculptured. Reticulation double; large meshes distinctly more strongly developed than small meshes. Large meshes may contain 2–5 small meshes. Impunctate, except at eyes; with fine, irregular punctures, the area of which extends a short distance towards middle of head-disc.

Pronotum: Pale ferrugineous, frontally and basally in middle with narrow, often indistinct, ferrugineous to dark ferrugineous marking. Slightly mat, microsculptured. Reticulation double. Large meshes clearly, more strongly developed than small meshes. Large meshes may contain 2–5 small meshes. Almost impunctate, except frontally and discally; with fine, sparse and irregular punctures.

Elytra: Pale ferrugineous, with dense, almost uniform, dark brown to dark ferrugineous irrorations (Fig. 439). Rather shiny, although microsculptured. Reticulation double. Large meshes in part quite indistinct. When discernible large meshes may contain 2–5 small meshes. Discally with fine, irregular row of punctures. Scattered, sparse punctures may also be discerned outside discal row-area. Pre-apical, lateral row of punctures fine, provided with some hairs.

Ventral aspect: Pale ferrugineous. Almost impunctate; apical ventrite with some scattered, rather fine punctures. Apical ventrite asymmetric having a small, sharp knob on one side (Fig. 92). Rather shiny, very finely and in part indistinctly microsculptured. Ventrites with very fine, slightly curved striae. Metacoxal plates with shallow, especially in posterior half, reduced and indistinct, furrows, which are almost transversely located. Prosternal process quite slender, posteriorly slightly extended, apically pointed.

Legs: Pale ferrugineous. Pro- and mesotarsus slightly enlarged, with distinct suckers.

Male genitalia: Penis in lateral aspect peculiar, being quite slender and curved, provided with a broad extension on inner outline of penis. Extreme apex broadly but moderately hooked (Fig. 285).

Female: Pro- and mesotarsus slender. Apical ventrite as in Fig. 93.

##### Etymology.

The species name *enigmaticus* is a Latin adjective meaning “enigmatic” and refers to the situation that, externally, *Laccophilus enigmaticus* resembles of some other African species but regarding male genitalia, it deviates strongly from the corresponding features in those species.

##### Distribution.

Nigeria, Sudan (Fig. 555).

##### Collecting circumstances.

Unknown, no information available.

#### Laccophilus restrictus

Taxon classificationAnimaliaColeopteraDytiscidae

Sharp, 1882

**Figures 96–109.**
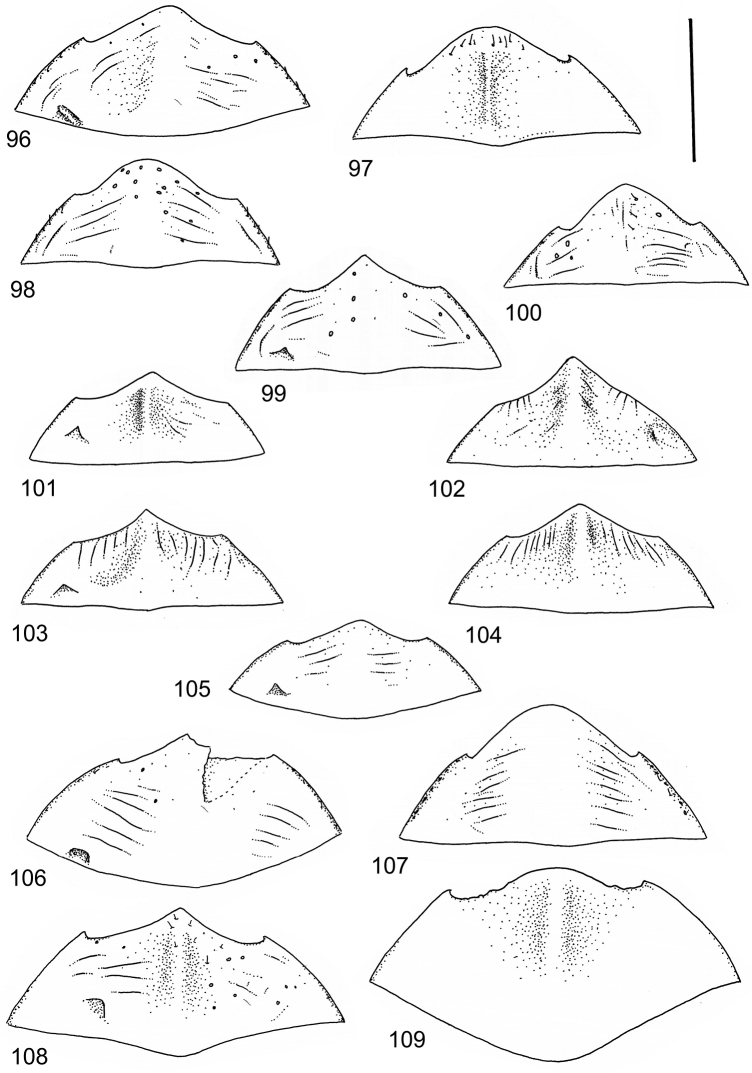
Apical ventrites **96** *Laccophilus restrictus* male, and **97** female **98** *Laccophilus amicus* female **99** *Laccophilus bellus* male, and **100** female **101** *Laccophilus septicola* male, and **102** female **103** *Laccophilus pullatus* male and **104** female **105** *Laccophilus luteosignatus*, male **106** *Laccophilus benoiti* male, and **107** female **108** *Laccophilus addendus* male, and **109** female. Scale bar 0.5 mm.

Figs 96–97 , 286–287 , 440–441 , 556

Laccophilus restrictus Sharp 1882: 315 (original description, faunistics); v. d. Branden 1885: 23 (catalogue, faunistics); Régimbart 1895: 135 (description, faunistics); Sharp 1904: 3 (faunistics, discussion); Zimmermann 1920a: 25 (catalogue, faunistics); Zimmermann 1930: 21, 23 (description, faunistics); Guignot 1946c: 282 (description, faunistics); Guignot 1959a: 536 (list, synonymy, *Laccophilus pictipennis* Sharp); Alfieri 1976: 31 (faunistics); Nilsson and Persson 1993: 79 (list, synonymy, *Laccophilus pictipennis* Sharp); Nilsson 2001: 249 (catalogue, faunistics, list, synonymy, *Laccophilus pictipennis* Sharp); Nilsson 2015: 216 (catalogue, faunistics, list synonym *Laccophilus pictipennis* Sharp). **Restored species.**
Laccophilus evanescens Severin 1892: 472 (nomen nudum); Régimbart 1895: 134 (original description, faunistics); Zimmermann 1920a:18 (catalogue, faunistics); Guignot 1946c: 270, 273, 278, 312 (description, faunistics, discussion); Legros 1953: 1562 (faunistics); Guignot 1955g: 865 (faunistics); Guignot 1956a: 88 (faunistics); Guignot 1956b: 219 (faunistics); Omer-Cooper 1956: 21, 23 (faunistics, biology); Guignot 1959a: 562, 568 (description, faunistics); Omer-Cooper 1965: 77, 87 (description, faunistics); Legros 1972: 466 (faunistics); Bilardo 1976: 190 (faunistics, biology); Bilardo and Pederzani 1978: 119 (faunistics, description); Bilardo and Rocchi 1987: 104 (faunistics, biology); Pederzani 1988: 107 (faunistics, biology); Bilardo and Rocchi 1990: 161, 177 (faunistics, biology); Nilsson and Persson 1993: 81, 94 (faunistics, biology); Nilsson et al.1995: 505 (faunistics); Rocchi 2000: 24 (faunistics); Nilsson 2001: 243 (catalogue, faunistics); Bilardo and Rocchi 2002: 156, 161, 174 (faunistics, list); Pederzani and Reintjes 2002: 40 (faunistics); Reintjes 2004: 67 (faunistics); van Vondel 2005: 130 (faun, biology); Bilardo and Rocchi 2013: 141 (faunistics); Nilsson 2015: 211 (catalogue, faunistics). **New synonym.**

##### Type localities.

*Laccophilus restrictus*: Egypt.

*Laccophilus evanescens*: Zaire: Matadi.

##### Type material studied

(2 exs.). *Laccophilus restrictus*: Lectotype (by present designation): male: “Type / Egypt / Sharp Coll. 1905-313 / Type 588 *Laccophilus restrictus* sp. n. Egypt” (BMNH).

*Laccophilus evanescens*: Cotype: female: “Matadi Congo / Museum Paris coll. Maurice Régimbart 1908 / *evanescens* Régb.” (MNHN). [Comments: the label data fits perfectly with what is given in the original description and accordingly we consider it as belonging to the type material despite it lacks type label. Previous authors have stated that the type material is deposited in IRSNB. We have examined two specimens from IRSNB (see below) with sampling locality Banana-Boma, located very closely to Matadi, which is given as type locality in the original description. One of the specimens from Boma is additionally provided with a type label. Status of these two specimens as type material of *Laccophilus evanescens* is, however, considered improbable. No lectotype designation is done here because an additional specimen from Matadi may still exist. It could be a male and in that case it is more suitable to be the lectotype.]

##### Additional material studied

(73 exs,). **Ethiopia**: “Eth. Merid., Delta de l’Omo Lac Rodolphe 570 m” (1 ex. MNHN). – **Sudan**: “R. Yei at Amadi 28.1. 1954 JJOC.” (9 exs. AMGS); “Nimule Ferry 4.XI. 1954 J. & J. Omer-Cooper” (1 ex. AMGS); “Chartum coll. Baum” (1 ex. CGW); “Bahr el Abiad / *Laccophilus evanescens* Régb. det. Brancucci 1982” (1 ex. ZMHB); “Equatoria Nzara 22.4. 1986 Wewalka” (2 exs. CGW, 1 ex. MZH); “Wad Medani am Bl. Nil 20.10. 1979 lux Hieke” (1 ex. ZMHB); same but “26.10. 1979” (1 ex. ZMHB). – **Mali**: “Bamako” (1 ex. MNHN); “Kogoni X. 1966 Schmitz” (1 ex. MRAC; habitus in Fig. 440). – **Chad**: “Lake Chad nr Abide 23.5. 1973 Linnavuori” (7 exs. MZH); “Bebedja 28-31.5. 1973 Linnavuori” (1 ex. MZH). – **Guinea**: “Faranah, Sidakoro, base-vie N10°14'27”, W10°27'41”, 6-27.5. 1994 Mei / *Laccophilus evanescens* Régb. det. Rocchi 1996” (1 ex. CSR). – **Burkina Faso**: “Ob. Volta Pundu Olsifiev” (1 ex. NHRS). – **Ivory Coast**: “Riv. Leraraba” (1 ex. MNHN); Ferkessédougou 10-20.5. 1964 Decelle” (1 ex. MRAC; habitus in Fig. 441). – **Benin**: “Dep. Littoral, Toho nr Pahou (village) 2.2. 2006 Goergen, Homarek & Hounguè leg./06°23'11.3"N 02°12'30,2” E, lake with rich riparian vegetation” (1 ex. NMW). – **Nigeria**: “R Jos-Bauchi rd. 9.IV. 1963 JOC.” (1 ex. AMGS); “R. St. nr Mbiama 4-5.7. 1973 Linnavuori” (2 exs. MZH); “NW St. Badeggi, rice fields 8-9.8. 1973 Linnavuori” (1 ex. MZH); “BPl. St. Wamba-Gudi 7.8. 1973 Linnavuori” (1 ex. MZH); “K. State N. Bussa 12.1. 1970 Medler” (1 ex. USNM). – **Gabon**: “Ogové Riv. Good / *Laccophilus evanescens* Régb. det. Rocchi 1991” (1 ex. CSR); “Lambarènè 7.8. 1973 Bilardo & Pederzani” (3 exs. CFP). – **Zaire**: “Banana–Boma M. Tschoffen 91 Det. Régimb. / 11170/Régimbart det. 1895 / Type (type label only pinned to female specimen) / Régimbart det. 1895 *Laccophilus evanescens* Rég”. (2 exs. IRSNB); PNG 17.7.1952/Miss. H. De Saeger II/fc/14 3806” (1 ex. MNHN); “Tshuapa-Mbandaka ca. 0°03'N-18°28'E, a.l. 1964 Stam leg.” (1 ex. RMNH, 1 ex. MZH); “Coquilhatville 3-4.4. 1963 Stam leg. / at light”(1 ex. RMNH). – **Kenya**: “Br.O.A., Fort Hall” (1 ex. ZMHB). – **Zambia**: “27.3.1993, 15°02'35"S/26°00'09E, Kafue NP Chunga Camp lux, Uhlig leg.” (1 ex. MZH); “29.3. 1993, 15°02'35"S/26°00'09E, Kafue NP Chunga Camp lux, Uhlig leg.” (1 ex. ZMHB). – **Mozambique**: “Beira 7.IX. 1955 JOC.“ (5 exs. AMGS); “Umbuluzi R. Nr Goba 4.12. 1948 JOC.” (2 exs. AMGS). – **Namibia**: “Kavango: Gelukkie Kavango Ufer 18°03'S/21°08'E, 1.3. 1992 Uhlig leg.” (5 exs. ZMHB, 2 exs. MZH, 1 ex. NMNW); “Kavango, Popa Falls 18°07'S/21°35’ E Kavango Ufer, Schilf-Papyrus-Ufer-Vegetation, gesiebt 2.3. 1992 leg. M. Uhlig” (2 exs. ZMHB); “E Capriwi, Mudumu NP Nakatwa 18°10’/23°26’ E, 8-13.3. 1992 lux. Uhlig leg.” (1 ex. ZMHB); “30.3.1999, 18°07'16S/21°34'51 Popa Falls NP. Banks of Okawango, floating Uhlig” (1 ex. ZMHB). – **Botswana**: “6.4. 1998 Shakawe Fishing Camp 18°27'S, 21°56'E Deckert leg.”(1 ex. ZHMB). – **South Africa**: “Natal Sodwana Bay Nat.P. 20.12. 1992 Koch leg.” (1 ex. ZHMB).

##### Comments on synonymy.

Type material of the two involved species, *Laccophilus restrictus* and *Laccophilus evanescens*, have been compared together with study of some additional specimens. No male type material of *Laccophilus evanescens* have, however, been available but external similarity between type material of the two species together with additional studies strongly indicate that they are synonymous. *Laccophilus restrictus*, being the older name is the valid name of the species. *Laccophilus restrictus* was earlier considered synonymous with *Laccophilus pictipennis* Sharp. Examination of the type material of *Laccophilus pictipennis*, however, clearly shows that the two species are not synonyms and accordingly *Laccophilus restrictus* deserves rank as a good species.

##### Diagnosis.

*Laccophilus restrictus* is characterized by vague and weak dorsal colour pattern in combination with penis, which in lateral view has a somewhat angled outline close to base of penis; from angle forwards penis evenly curved to extreme apex. Penis apex is extended to a small tip.

##### Description.

Body length 3.3–3.8 mm, width 1.8–2.0 mm. Oval to oblong; dorsal aspect pale ferrugineous to ferrugineous. With somewhat irregular and weakly developed, dense irroration on elytra. Irroration rarely quite distinct (Figs 440–441).

Head: Pale ferrugineous; posteriorly slightly darker but dark area lacks clear delimitation. Slightly mat, finely microsculptured. Reticulation double, of two kinds; difference between fine and larger reticulation in part indistinct. Large meshes include 2–5 small meshes. At eyes, with fine, irregular punctures.

Pronotum: Pale ferrugineous to ferrugineous; without distinct darker markings. Submat, finely microsculptured; microsculpture in part indistinct. Reticulation double but in part distinction of different kinds of reticulation difficult. Larger meshes (when distinguishable) include 2–5 small meshes. Laterally and anteriorly, with sparse, irregular, fine punctures.

Elytra: Pale ferrugineous, with dense, vague, ferrugineous irrorations; rarely elytral colour pattern comparatively distinct (Figs 440–441). Rather shiny, very finely microsculptured. Reticulation in part double; posteriorly this feature indistinct or weakly developed; anteriorly slightly more distinct. Discal row of punctures fine, slightly irregular but still clearly discernible. Additional punctation very fine, irregular and hardly visible.

Ventral aspect: Pale ferrugineous, metacoxal process-area slightly darker; ferrugineous to dark ferrugineous. Slightly mat, finely microsculptured. Striae and furrows on metacoxal plates and abdomen fine and vague. Almost impunctate. Prosternal process apex slender, extended and pointed. Apical ventrite, with fine knob on one side (Fig. 96).

Legs: Pale ferrugineous to ferrugineous. Protarsal claws slightly extended; and moderately curved. Protarsus and mesotarsus slightly enlarged, somewhat extended and provided with distinct suckers.

Male genitalia: Penis in lateral aspect anterior to base distinctly angled and from there forwards, curved to apex, which is extended to a small tip (Figs 286–287).

Female: Externally almost as male. Apical ventrite as in Fig. 97. Pro- and mesotarsus slender.

##### Distribution.

Egypt, Ethiopia, Sudan, Mali, Guinea, Chad, Burkina Faso, the Ivory Coast, Benin, Nigeria, Gabon, Zaire, Kenya, Zambia, Mozambique, Namibia, Botswana, South Africa (Fig. 556). Additional, unverified records are Mauretania (Guignot 1955g), Senegal (Guignot 1956b), Niger (Omer-Cooper 1965), Tanzania (Bilardo 1976) and Guinea Bissau (Nilsson et al.1995).

##### Collecting circumstances.

In Mozambique *Laccophilus restrictus* has been collected in a small, grassy stream and in a marsh with lily pools and weeds (Omer-Cooper 1956). Van Vondel (2005) reports that the species has been sampled e.g. in stagnant remain of brooklet, in pool with loamy bottom, and at light. In Tanzania collected with UV-light (Bilardo 1976). Additional information on sampling localities where *Laccophilus restrictus* has been collected is available in Bilardo and Rocchi (1987, 1990), Pederzani (1988) and Nilsson and Persson (1993).

#### Laccophilus amicus

Taxon classificationAnimaliaColeopteraDytiscidae

Guignot, 1955

Figs 98 , 442 , 550

Laccophilus amicus Guignot 1955b: 1096, 1098 (original description, faunistics); Nilsson 2001: 240 (catalogue, faunistics); Nilsson 2015: 208 (catalogue, faunistics).

##### Type locality.

Guinea: Kindia.

##### Type material studied

(1 ex.). Holotype, female: “IFAN 1964 Kindia Guinee Fse A. Villiers / Type / F. Guignot det. 1955 *Laccophilus amicus* sp. n. Type female” (MNHN).

##### Additional material studied

(1 ex.). **Liberia**: “Suakoko 19.12. 1951 / 6-9 pm light trap Blickenstaff” (1 ex. USNM; habitus in Fig. 442).

##### Diagnosis.

Only female known, which makes diagnosing difficult. According to Guignot (1955b) *Laccophilus amicus* resembles externally of *Laccophilus tschoffeni* Régimbart (here located in species group 11 (*deceptor*)) but with distinctly smaller body. At present, the small body, in combination with peculiar elytral colour pattern is the most useful characters for identification of the species. *Laccophilus amicus* seems to be closely related to *Laccophilus restrictus* and *Laccophilus bellus*, on the basis of external similarity. Further study will reveal, whether *Laccophilus amicus* proves to be synonymous with *Laccophilus restrictus*.

##### Description

(only female). Body length 3.3–3.4 mm, width 1.8 mm. Habitus dorsal aspect as in Fig. 442.

Head: Pale ferrugineous. Finely microsculptured, reticulation double. Large meshes only slightly stronger developed than small meshes. Large meshes, when discernible, contain 2–5 small meshes. Mesh-size-classes cannot always be distinguished. Almost impunctate; a few very fine, scattered punctures may be discerned at eyes.

Pronotum: Pale ferrugineous, anteriorly and basally in middle with vague dark ferrugineous to ferrugineous areas. Basal dark area bilobed. Finely microsculptured, reticulation double. Large meshes only slightly stronger developed than small meshes. Large meshes, when discernible, contain 2–5 small meshes. Mesh-size-classes cannot always be distinguished. Almost impunctate, frontally and laterally with very fine, sparse punctures.

Elytra: Ferrugineous to brownish, with, pale ferrugineous markings. At base, posterior to middle and apically with pale area expanded; dark ferrugineous to brown irrorations are there sparser (Fig. 442). Rather shiny, although finely microsculptured. Reticulation double but especially large meshes weakly developed and only indicated by mesh-rudiments. Fine, irregular punctures form a discal row. Dorsolateral and lateral rows of punctures indistinct; indicated by a few scattered punctures. Posterolaterally with a fine, slightly pubescent pre-apical furrow.

Ventral aspect: Pale ferrugineous; no colour pattern. Rather shiny, very finely microsculptured. Reticulation in part hardly discernible, almost absent. Almost impunctate. Curved striae on abdomen very fine, in part reduced and indistinct. Prosternal process slender, pointed. Metacoxal furrows very indistinct and reduced; some weak fragments only discernible. Apical ventrite symmetric (Fig. 98).

Legs: Pale ferrugineous to ferrugineous. Pro- and mesotarsus slender.

Male: Thus far unknown.

##### Distribution.

Guinea, Liberia (Fig. 555).

##### Collecting circumstances.

Almost unknown. In Liberia collected with light trap.

#### Laccophilus bellus sp. n.

Taxon classificationAnimaliaColeopteraDytiscidae

http://zoobank.org/B9A796D4-D74D-4CD1-8167-EEFBD988DB4A

Figs 99–100 , 288 , 443 , 555

##### Type locality.

Benin: Dep. Zou, Hlanzoun Riv, Zogbodomè Lokoli (forest) (07°03'N 02°15'E).

##### Type material

(20 exs.). Holotype, male: “Bénin: Dep. Zou, Zogbodomè Lokoli (forest), Hlanzoun Riv. 6.II. 2006 leg. Goergen, Komarek & Hounguè (18)/07°03'N 02°15'E muddy stream” (NMW). – Paratypes: Same data as holotype: (11 exs. NMW, 3 exs. MZH; habitus in Fig. 443). – Nigeria: “In vegetation in the river/Nigeria: Abraka (Kwale), ? Warri Prov. ii. 1949 B. Malkin/Brit-Mus. 1956-234” (1 ex. BMNH); “Nigeria Delta St. Osobi Wetland Area N5.533; E5.816, 14.5. 2008 Mesumbe” (1 ex. AMGS); “Nigeria Delta St., R. Orogoda N6.333, E6.250 nr water, 20.7. 2005 Mesumbe” (2 exs. AMGS, 1 ex. MZH).

##### Diagnosis.

*Laccophilus bellus* is characterized by peculiar elytral, colour pattern, by comparatively small body and by the shape of the penis, differing it from all other recognized *Laccophilus* species in Africa. Penis in lateral aspect, delicate, close to base at external outline distinctly angled and forwards from there almost straight to slightly extended tip. Extreme apex only weakly hooked. Possibly, close to *Laccophilus amicus* on the basis of external resemblance. Male genitalia of *Laccophilus amicus* are thus far unknown since only female is known of it.

##### Description.

Body length: Length 3.1–3.3 mm, width 1.6–1.7. Elytral colour pattern quite uniform; rarely reduced or extended (Fig. 443). Specimen from Nigeria with elytral colour pattern slightly vague.

Head: Uniformly pale ferrugineous. At eyes with fine, irregular punctures, which extend towards middle of head. Finely microsculptured; reticulation double. Size classes of microsculpture in part difficult to distinguish because almost equally, strongly developed. Large meshes, when discernible may contain 2–5 fine meshes.

Pronotum: Pale ferrugineous. Basally in middle with a rather narrow, dark brownish area. Frontally in middle with a narrow, weakly delimited darker area. Discally, broadly impunctate. At margins with fine, irregular punctures except medially at base where pronotum is also impunctate. Rather shiny although finely microsculptured. Reticulation indistinctly double. Size-classes of reticulation in part, difficult to distinguish. Large meshes extensively hardly discernible.

Elytra: Dark ferrugineous to dark brown, generally with well delimited, pale ferrugineous markings. On dark areas broad, dark undulations may be discerned (Fig. 443). Discal row of punctures somewhat irregular but clearly discernible. Dorsolateral and lateral rows of punctures rather indistinct, consist of scattered, irregular punctures. Pre-apical, lateral row of punctures finely pubescent. Rather shiny, although finely microsculptured. Reticulation double; coarse meshes reduced and in part difficult to discern.

Ventral aspect: Pale ferrugineous to ferrugineous, no distinct colour pattern. Almost impunctate. Rather shiny although finely microsculptured. Sternites with sparse, slightly curved striae. Metacoxal plates with about 10 fine, partly reduced, almost transverse furrows. Apical sternite asymmetric, with small knob on one side (Fig. 99). Prosternal process slender, extended and apically pointed.

Legs: Pro- and mesotarsus slightly enlarged, with suckers.

Male genitalia: Penis in lateral aspect, delicate, close to base at external outline distinctly angled and forwards from there almost straight/slightly sinuate to slightly extended tip. Extreme apex only weakly hooked (Fig. 284).

Female: Apical sternite lacks knob (Fig. 100). Pro- and mesotarsus narrower.

##### Etymology.

The name *bellus* is a Latin adjective meaning “beautiful”. The name refers to the external appearance of the new species, being especially handsome.

##### Distribution.

Benin, Nigeria (Fig. 555).

##### Collecting circumstances.

Almost unknown. From label data it appears that the species has been collected in a muddy stream and in vegetation of a river.

#### Laccophilus septicola

Taxon classificationAnimaliaColeopteraDytiscidae

Guignot, 1956

Figs 101–102 , 289–290 , 444 , 557

Laccophilus septicola Guignot 1956b: 220, 221 (original description, faunistics); Omer-Cooper 1967: 60 (discussion, *Laccophilus pullatus* Omer-Cooper *Laccophilus septicola* Guignot, *Laccophilus alberticus* Guignot and *Laccophilus luteosignatus* Gschwendtner are synonyms); Medler 1980: 155 (faunistics, list); Nilsson 2001: 250 (catalogue, faunistics, list, synonymy, *Laccophilus luteosignatus* Gschwendtner); Nilsson 2015: 213 (catalogue, faunistics, list, synonymy, *Laccophilus luteosignatus* Gschwendtner). **Restored species.**
Laccophilus alberticus Guignot 1959d: 163 (original description, faunistics, discussion); Omer-Cooper 1967: 60 (discussion, *Laccophilus pullatus* O-mer-Cooper, *Laccophilus septicola* Guignot., *Laccophilus alberticus* Guignot and *Laccophilus luteosignatus* Gschwendtner are synonyms); Nilsson 2001: 246 (catalogue, faunistics, list, synonymy, *Laccophilus luteosignatus* Gschwendtner; Nilsson 2015: 213 (catalogus, list, synonymy, *Laccophilus luteosignatus* Gschwendtner). **Confirmed synonymy.**

##### Type localities.

*Laccophilus septicola*: Senegal: Niokolo-Koba, Badi.

*Laccophilus alberticus*: Zaire: Lake Albert, Mwita.

##### Type material studied

(8 exs.). *Laccophilus septicola*: Holotype: male; “Mission IFAN au Parc National du Niokolo Koba Badi (Sénégal) 15.VIII.-25.IX-1955 / Type / F. Guignot det. 1954 / F. Guignot det.1954 *Laccophilus septicola* sp. n. Type (MNHN). – Paratypes: “Mission IFAN au Parc National du Niokolo Koba Badi (Sénégal) 15.VIII.-25.IX-1955 / Paratype / Dr. F. Guignot det. *Laccophilus septicola* Guign.” (4 exs. IRSNB); same but “Ouassadou 12.VIII. 55” (1 ex. IRSNB).

*Laccophilus alberticus*: Holotype: male: “Congo Belge. Lac Albert: Mwita (prét foret galerie) U.V. 22.XII. 1953 J. Verbeke – KEA 4083 / Type / F. Guignot det., 1956 *Laccophilus alberticus* sp. n. Type male” (IRSNB). – Paratype: “Congo Belge Lac Albert: Bezaha U.V. 19.12. 1953, 4070 / female symbol / Paratype” (1 ex. MNHN).

##### Additional material studied

(29 exs.): **Sudan**: “Upper Nile Malakal 5-20.1. 1963 Linnavuori” (22 exs. MZH; habitus in Fig. 444); same data and “ad lucem” (2 exs., MZH); “Sudan / Linnavuori” (1 ex. MZH); “L. Shambe 21.1. 1954 Omer-Cooper” (2 exs. AMGS). – **Nigeria**: “Marsk, Katsina-Dawura rd. 6.10.1963 JOC.” (1 ex. AMGS). – **Zaire**: “Lac Albert Kasenyi UV 21.VI. 1953 4012” (1 ex. IRSNB).

##### Comments on synonymy.

Earlier synonymy of *Laccophilus septicola* and *Laccophilus alberticus* is verified by examination of their holotypes. *Laccophilus septicola*, being the older name is the valid name of the species. Furthermore, examination revealed that *Laccophilus septicola* is not synonymous with *Laccophilus luteosignatus* and *Laccophilus pullatus*.

##### Diagnosis.

*Laccophilus septicola* is characterized by pale ferrugineous to ferrugineous elytra in combination with peculiarly shaped penis (extreme apex slightly extended). Morphologically, in case of penis and body shape, *Laccophilus septicola* resembles much of *Laccophilus pullatus* and *Laccophilus luteosignatus*. These two species have, however, blackish to dark ferrugineous elytra, which separate them clearly from *Laccophilus septicola*, with its much paler main colour of elytra.

##### Description.

Body length 2.9–3.3 mm, width 1.6–1.8 mm. Dorsal, aspect of body almost without colour pattern (Fig. 444). Rarely humeral regions may be slightly paler forming minor pale spots.

Head: Pale ferrugineous. Submat, finely reticulated. Reticulation indistinctly double. Large meshes indistinct; only in part discernible and slightly more strongly developed than small meshes. Almost impunctate; fine punctures discernible at eyes.

Pronotum: Pale ferrugineous, mediobasally sometimes slightly darker, but lacks well delimited darkened area. Submat, finely microsculptured. Reticulation double; large meshes contain two to five small meshes. Small meshes sometimes weakly developed and hardly discernible. Punctation sparse, very fine, in part absent; discernible at frontal margin and laterally.

Elytra: Pale ferrugineous to ferrugineous. In comparsion with head and pronotum, elytra extensively slightly darker (Fig. 444). Rarely with somewhat vague, paler humeral spots. Submat, finely microsculptured. Double reticulation only in part discernible (at basal region). Punctation very fine and irregularly distributed; large areas lack punctures. Irregular, somewhat sparse punctures form a rather indistinct, discal row. Lateral, pre-apical furrow fine, finely pubescent.

Ventral aspect: Pale ferrugineous. Submat, very finely and partly indistinctly microsculptured. Basal ventrirtes with fine, curved striae. Almost impunctate. Metacoxal lines-process-area not distinctly modified. Metacoxal plates in anterior half with fine, in part rather indistinct, transversely located furrows. In posterior half furrows almost absent. Prosternal process slightly extended, rather slender, pointed. Apical ventrirte asymmetric; with a sublateral, sharp knob (Fig. 101).

Legs: Protarsus slender, claws moderately extensive and slightly curved. Pro- and mesotarsus with suckers.

Male genitalia: Penis in lateral aspect medially bent and from there penis narrows towards extreme apex, which is extended to a small tip (Figs 289–290).

Female: Apical ventrirte simple (Fig. 102). Pro- and mesotarsus almost as in male but lack suckers.

##### Distribution.

Senegal, Sudan, Nigeria, Zaire (Fig. 557). Only verified records accepted in map because of different earlier taxonomic opinion regarding species delimitations.

##### Collecting circumstances.

Not documented. In Sudan sampled at light collection.

#### Laccophilus pullatus

Taxon classificationAnimaliaColeopteraDytiscidae

Omer-Cooper, 1958

Figs 103–104 , 291–292 , 445 , 557

Laccophilus pullatus Omer-Cooper 1958b: 42, 45 (original description, faunistics, biology); Omer-Cooper 1967: 60 (discussion, *Laccophilus pullatus* synonym with *Laccophilus septicola* Guignot, *Laccophilus alberticus* Guignot and *Laccophilus luteosignatus* Gschwendtner); Nilsson 2001: 246 (catalogue, faunistics, list, synonymy, *Laccophilus luteosignatus* Gschwendtner); Nilsson 2015: 213 (catalogue, faunistics, list, synonymy, *Laccophilus luteosignatus* Gschwendtner). **Restored species.**

##### Type locality.

Malawi: Florence Bay below Livingstonia.

##### Type material studied

(2 exs.). Holotype: male: “Holotype / *Laccophilus pullatus* mihi Det. J. Omer-Cooper / Nyasaland, lake shore below Livingstonia 21.10. 1948 / Brit. Mus. 1978-308 / *Laccophilus pullatus* J. O.C. M. E. Bacchus det 1978 HOLOTYPE” (BMNH). – Paratype: female: “Allotype / *Laccophilus pullatus* sp. n. J. Omer-Cooper / Nyasaland, Dambo below Livingstonia 21.10. 1948 / Brit. Mus. 1978-308” (1 ex. BMNH).

##### Additional material studied

(3 exs.). **Malawi**: “Nkhotakota env. 2-3.1. 2002 J. Bezdek leg.” (1 ex. NMPC); “Selima env. 4.1. 2002 Kantner” (1 ex. NHMB, 1 ex. MZH; habitus in Fig. 445).

##### Specimen with uncertain determination.

South Africa: “Natal Zululand Mtuba-Tuba 23.9. 1947 J. O-C” (1 ex. female AMGS).

##### Comments on synonymy.

Earlier synonymy rejected – study of type material shows that *Laccophilus pullatus* is a valid species.

##### Diagnosis.

*Laccophilus pullatus* externally resembles most of *Laccophilus luteosignatus* but can be distinguished by absence of pale spots on elytra. *Laccophilus pullatus* is also close to *Laccophilus septicola*; elytra of *Laccophilus septicola* is, however, much paler than in *Laccophilus pullatus*. Penis of *Laccophilus pullatus* in lateral aspect is evenly curved from base to apex while in *Laccophilus septicola* penis curvature is somewhat angled.

##### Description.

Body length 3.0–3.3 mm, width 1.6–1.8 mm. Head and pronotum predominantly pale, elytra dark, lack pale spots (Fig. 445).

Head: Pale ferrugineous. Submat, finely microsculptured. Reticulation almost simple; vague indications of double reticulation discernible. Almost impunctate; at eyes with some, irregular and very fine punctures.

Pronotum: Pale ferrugineous. Frontally, a little posterior to foremargin with a vague, transverse, ferrugineous marking. Posteriorly, in the middle with a rather vague, transverse, ferrugineous to dark ferrugineous marking. Finely microsculptured. Reticulation double. Large meshes vary in size. Finer meshes sometimes obliterated and not distinguishable within large meshes. At margins with very fine, irregular punctation, which in part is indistinct.

Elytra: Blackish to blackish ferrugineous. Laterally, narrowly paler, ferrugineous to dark ferrugineous (Fig. 445). Rather shiny, although finely microsculptured. Reticulation double, but differences between large and small meshes superficial; sometimes difficult to distinguish (posteriorly meshes hardly discernible). Large meshes variable in size and may contain 2–10 small meshes. Almost impunctate; discally with two vague areas where scattered, fine punctures discernible. Elytron with a pre-apical, lateral furrow (provided with haired punctures).

Ventral aspect: Black to blackish ferrugineous, except for head and prothorax, which are pale ferrugineious. Slightly mat, very finely and in part, indistinctly microsculptured. Metacoxal plates with variable, transverse, shallow (vague) furrows. Basal ventrites with longitudinal, fine striae. Impunctate. Prosternal process rather slender, apex pointed. Apical ventrite has a sharp knob on one side (Fig. 103).

Legs: Pale ferrugineous to ferrugineous. Pro- and mesotarsi somewhat enlarged; provided with suckers.

Male genitalia: Penis in lateral aspect evenly curved from base to apex; extreme apex extended to a small tip (Figs 291–292).

Female: Apical ventrite lacks knob (Fig. 104). Pro- and mesotarsus slender.

##### Distribution.

Malawi (Fig. 557). One female with uncertain determination has been examined from Natal, South Africa.

##### Collecting circumstances.

Collected in a “dambo” swamp on a lake shore (Omer-Cooper 1958b).

#### Laccophilus luteosignatus

Taxon classificationAnimaliaColeopteraDytiscidae

Gschwendtner, 1943

Figs 105 , 293–294 , 446 , 557

Laccophilus luteosignatus Gschwendtner 1943: 417 (original description, faunistics); Guignot 1954: 26 (description, faunistics); Guignot 1959a: 534, 542 (description, faunistics); Omer-Cooper 1967: 60 (discussion, *Laccophilus pullatus* Omer-Cooper, *Laccophilus septicola* Guignot, *Laccophilus alberticus* Guignot and *Laccophilus luteosignatus* Gschwendtner are synonyms); Nilsson 2001: 246 (catalogue, faunistics); Nilsson 2015: 213 (catalogue, faunistics). **Restored species.**

##### Type locality.

Zaire: Bukama.

##### Type material studied.

Holotype: male: “Type Gschw. / Coll. Mus. Congo Bukama –VII-1937 Lt Mardée / coll. Gschwentner / *Laccophilus luteosignatus* Gschw. det. Gschwendt. / Type”. (1 ex. OLML; habitus in Fig. 446).

##### Comments on synonymy.

*Laccophilus luteosignatus* was earlier considered synonymous with three other *Laccophilus* species (see above). After examination of types of all involved species we consider *Laccophilus luteosignatus* not synonymous with *Laccophilus pullatus*, *Laccophilus septicola* or *Laccophilus alberticus*, but being a separate species.

##### Diagnosis.

*Laccophilus luteosignatus* resembles most of *Laccophilus septicola* and *Laccophilus pullatus*. From the former species *Laccophilus luteosignatus* is separated by mostly blackish to blackish ferrugineous elytra while from *Laccophilus pullatus* by exhibiting minor but well delimited pale markings on elytra; elytra of *Laccophilus pullatus* lack pale markings. Male genitalia seem to be quite similar shaped in all three species, however, penis of *Laccophilus pullatus* and *Laccophilus luteosignatus* is evenly curved while distinctly bent in *Laccophilus septicola*.

##### Description.

Body length 2.9 mm, width 1.6 mm. Body distinctly bicoloured; head and pronotum predominantly pale ferrugineous, and elytra blackish to dark ferrugineous with minute pale humeral markings (Fig. 446).

Head: Pale ferrugineous. Submat, very finely microsculptured; reticulation indistinctly double. Coarse reticulation with meshes almost absent and very indistinct; fine reticulation with distinct meshes. At eyes with fine, irregular punctures.

Pronotum: Pale ferrugineous, medially at base with a transversely located dark ferrugineous marking. Finely microsculptured, reticulation double; coarser meshes only a little stronger developed in comparison with fine meshes. Coarse meshes contain 2–5 fine meshes. Almost impunctate; very fine, somewhat irregular punctures discernible.

Elytra: Blackish to dark ferrugineous, with minute, pale ferrugineous markings (Fig. 446). Submat, finely microsculptured. Reticulation double, but coarse meshes only indistinctly stronger developed than fine meshes. Posteriorly, coarse meshes become in part obliterated and indistinct. Very fine, somewhat irregular punctures discernible. Rows of punctures absent or very indistinct and hardly visible. Laterally, posterior to middle, with a discernible row of punctures.

Ventral aspect: Blackish to dark ferrugineous, thorax pale ferrugineous. Apex of prosternal process long, slender and pointed. Submat, very finely and in part indistinctly microsculptured. Metacoxal plates with some, shallow, transversely located furrows. Ventrites with distinct, somewhat curved striae. Almost impunctate. Male apical ventrite as in Fig. 105.

Legs: Pro- and mesotarsus quite extensive, slender and tarsal segments moderately enlarged with fine suckers.

Male genitalia: Penis in lateral aspect almost evenly curved from base to apex; extreme apex extended to a small but distinct tip (Figs 293–294).

Female: Unknown.

##### Distribution.

Zaire (Fig. 557).

##### Collecting circumstances.

Unknown.

#### Laccophilus benoiti

Taxon classificationAnimaliaColeopteraDytiscidae

Guignot, 1953

Figs 106–107 , 295–296 , 447 , 555

Laccophilus benoiti Guignot 1953b: 234, 236 (original description, discussion, faunistics); Guignot 1955c: 182, 188 (faunistics, female description); Guignot 1959a: 579, 581, 584 (description, faunistics); Rocchi 2000: 25 (discussion); Nilsson 2001: 240 (catalogue, faunistics); Nilsson 2015: 209 (catalogue, faunistics).

##### Type locality.

Zaire: Elisabethville.

##### Type material studied

(1 ex.). Holotype: male: “Holotypus / Coll. Mus. Congo Elisabethville, A la lumière XI-50/VI-51 Ch. Seydel / Type / R. DET H. 6182 / Guignot det., 1953 *Laccophilus benoiti* Guign. Type male” (MRAC; habitus in Fig. 447).

##### Additional material studied

(1 ex.). **Zaire**: “Allotypus female / Coll. Mus. Congo Elisabethville (à la lumière) 1-III-52/30-IX-1953 Ch. Seydel / Allotype / R. DET H 6649 ee. / Guignot det., 1954 *Laccophilus benoiti* Guign. Allotype female” (1 ex. MRAC; not type material). [Comment: attribution of the female specimen to *Laccophilus benoiti* is based on Guignot’s determination.]

##### Diagnosis.

*Laccophilus benoiti* is characterized by exhibiting inconspicuous external characters but with very peculiar, strongly angled penis, which separates it from all other African *Laccophilus* species. Thus far only one male, however, is known and available for study. Comparison with males of *Laccophilus epinephes* shows that their bodies are externally identical. The unique appearance of penis, being strongly bent, raises suspicion that it is a case of deformation and that *Laccophilus benoiti* in fact is conspecific with *Laccophilus epinephes*. Further study is needed to settle this problem.

##### Description.

Body length 4.3–4.4 mm, width 2.1–2.2 mm. Habitus (Fig. 447), distinct colour pattern absent (in female specimen, studied, there is a semi-transverse row of small, pale spots (about five spots/elytron) posterior to foremargin of elytra).

Head: Pale ferrugineous. Submat, finely microsculptured; reticulation in part indistinctly double. Large meshes hardly discernible; when discernible large meshes only slightly coarser than fine meshes. At eyes, areas with fine, irregular punctures, the areas which are extended a short distance towards middle of head-disc.

Pronotum: Pale ferrugineous, no distinct colour pattern. Very finely microsculptured; reticulation indistinctly double. Anteriorly and laterally with very fine, sparse and irregular punctures.

Elytra: Pale ferrugineous, with extensive and dense but somewhat indistinct, ferrugineous irrorations (Fig. 447). Rather shiny. Very finely microsculptured; reticulation very indistinctly double. Difference between coarser and fine meshes almost non-existent. Discal, dorsolateral and lateral row of punctures very fine and somewhat irregular.

Ventral aspect: Pale ferrugineous to ferrugineous, no distinct colour pattern discernible. Almost impunctate. Rather shiny, although very finely, in part indistinctly microsculptured. Semitransverse furrows on metacoxal plates shallow, in part indistinct. Abdomen basally with fine, curved striae. Prosternal process slender, apically pointed. Apical ventrite with a small, asymmetric knob (Fig. 106); ventrite broken.

Legs: Pale ferrugineous, pro- and mesotarsus slightly enlarged, with suckers.

Male genitalia: Penis strongly modified and different from all other African *Laccophilus* species; in lateral aspect penis forms an angle of almost 90° (Figs 295–296).

Female: Externally as male, but elytra basally among irrorations with a semitransverse, irregular row of small pale spots. Apical ventrites simple, without knob (Fig. 107). Pro- and mesotarsus slender.

##### Distribution.

Zaire (Fig. 555).

##### Collecting circumstances.

Almost unknown, sampled at light.

#### Laccophilus addendus

Taxon classificationAnimaliaColeopteraDytiscidae

Sharp, 1882

Figs 108–109 , 297–298 , 448 , 558

Laccophilus addendus Sharp 1882: 316 (original description, faunistics); v. d. Branden 1885: 20 (catalogue, faunistics); Régimbart 1895: 136, 137 (discussion, description, faunistics); Régimbart 1903: 14 (discussion); Peschet 1917: 25 (discussion, faunistics); Zimmermann 1920a:16 (catalogue, faunistics); Zimmermann 1926: 23 (faunistics, description); Bertrand 1928a: 185 (juvenile record, faunistics); Gschwendtner 1931: 180 (faunistics); Gschwendtner 1935a: 18 (faunistics); Gschwendtner 1938a: 5 (faunistics); Guignot 1946c: 271, 273, 280, 316 (description, faunistics); Vinson 1956: 28 (faunistics, list.); Guignot 1959a: 570, 576 (description, faunistics); Omer-Cooper 1965: 77, 85 (description, faunistics); Vinson 1967: 314 (faunistics, list); Bameul 1984: 94 (faunistics); Curtis 1991: 186 (faunistics); Rocchi 1991: 80, 86 (faunistics); Nilsson 2001: 240 (catalogue, faunistics); Pederzani and Rocchi 2009: 95 (faunistics, list); Nilsson 2015: 208 (catalogue, faunistics).
Laccophilus addendus v. geminatus Severin 1892: 472 (nomen nudum); Régimbart 1895: 137, 138 (original description, discussion, faunistics); Régimbart 1903: 14 (discussion); Peschet 1917: 24, 25, 55 (description, faunistics); Zimmermann 1920a:16 (catalogue, list, synonymy, *Laccophilus addendus* Sharp); Vinson 1956: 28 (faunistics); Omer-Cooper 1965: 85 (list, synonymy); Nilsson 2001: 240 (catalogue, faunistics, list, synonymy); Nilsson 2015: 208 (catalogue, faunistics, list, synonymy, *Laccophilus addendus* Sharp).
Laccophilus addendus ab. geminatus Régimbart, Guignot 1946c: 271 (description); 1959a: 576 (description, faunistics); Guignot 1961a: 931 (faunistics).
Laccophilus addendus geminatus Régimbart, Guignot 1961a: 931 (faunistics). **Confirmed synonym.**

##### Type localities.

*Laccophilus addendus*: Madagascar.

Laccophilus addendus var. geminatus: Madagascar.

##### Type material studied

(1 ex.). *Laccophilus addendus*: Holotype: female: “Madagascar 591 / Sharp Coll. 1905-313 / Type 591 *Laccophilus addendus* Madagascar” (BMNH; from original description it appears that type material contains only this specimen).

Laccophilus addendus v. geminatus (5 exs. with type status unclear): “Annanarivo Sikora Res(?) Régimb. -91 / *Laccophilus geminatus* Rég. sp. n. type” (3 exs. IRSNB); “Tananarive / Museum Paris Coll. Maurice Régimbart 1908 / v. geminatus Rég.” (2 exs. MNHN). [Comments: labelled, in part, as types but label data do not exactly fit with information given in the original description.]

##### Additional material studied

(54 exs.). **Madagascar**: “Suberbieville Breuning coll.” (2 exs. MRAC, 1 ex. MZH); “Tsarafidy Breuning coll.” (1 ex. MRAC); “Maroansetra E. le Moult” (1 ex. RMNH); “E Mad., Ampamoho nr Andilamena 1200-1300 m asl, 18-20.1. 1995 Dunay & Janak leg” (1 ex. NMW); “Fenerive, foret Tampolo 28.12. 1998 Moravec leg.” (1 ex. NMW, 1 ex. MZH); “Ft Dauphin (Tulear) Mandena (QMM area) / Pond at right border of Riv. Maendano, 13.9.2001, 10 m asl., 21.6˚C, 0.211 mS/cm / Gerecke & Goldsmith leg.” (3 exs. BMNH, 1 ex. MZH; habitus in Fig. 448); “Betsiboka Bas, Loc. Manjkavararadrano Marmokomita Riv., 46, 54°20'E/17° 38'00"S, alt 625, 16.4.1991 leg. ORSTOM” (1 ex. NMW); “Fianarantsoa Prov., Foret d’Ananalava 29.6 km. 280°, W Ranhohira, elev. 700 m 1-5.2. 2003/22°35'30"S, 045°07'42"E, Griswold et al., at light, in tropical dry forest” (1 ex. CAS); “TOLI, Zombitse Ankilemiletsy: muddy waterhole, N: -22.868, E: 44.576, 544 m 14.5. 2006 Bergsten leg. / BMNH(E) <794166, 794206, 794209> DNA voucher / *Laccophilus addendus* det. Bergsten” (3 exs. NHRS); “TOLI, Manakaravavy, Manakaravavy Riv., Dried out river P49: N: -24.52, E: 44.623, 218 m, 18.5. 2006 Bergsten et al. leg. / BMNH(E) <794207> DNA voucher / *Laccophilus addendus* det. Bergsten” (1 ex. NHRS); TOA, Moramanga, Andasibe, Andasibe NP Lenti, N: -18.935, E: 48.417, 933 m, 5.1. 2007, Isambert leg. / BMNH(E) <830996> MSL402:C5, DNA voucher / *Laccophilus addendus* det. Bergsten” (1 ex. NHRS); “Andasibe, NP Perinet 1250 m, Pfütze im Urvald 5.12. 2000, W. Dolin” (8 exs. NHRS); “Isaky Ivondro, Foret Managotry, Lotic, N: -24.799, E: 46.862, 406 m, 9.4. 2007, Ranarilalatiana leg. / DNA voucher BMNH(E)<831007> MSL 402:C9 / *Laccophilus addendus* det. Bergsten” (1 ex. NHRS); “Isalo, Source of Piscine Naturelle, waterholes, N: -22.553, E: 45.368, 2312 m, 12.5. 2006, Isambert et al. leg. / DNA Voucher BMNH(E) <831005, 831007>MSL402:C1 / *Laccophilus addendus* det. Bergsten” (2 exs. NHRS); “Sambava, Marojejy, Marojejy NP, Lentic, N: -14.457, E: 49.79, 162 m, 10.12. 2006, Isambert et al. leg. / DNA Voucher BMNH(E) <830992>MSL402:C1 / *Laccophilus addendus* det. Bergsten” (1 ex. NHRS); “Mahajanga Melaky btw Morafenobe-Ambohijanahary N-18.19091, E045.19986, 290 m.a.o. 19.12.2009 water net, field, Bergsten et al” (1 ex. NHRS); same data, add “NHRS-JLKB 000000500”(1 ex. NHRS); “Antsianaka Perrot Freres 1er semester 1892” (5 exs. ZMHB); “SW Madagascar Andranohinaly, Voeltskov S.” (1 ex. ZMHB); “Mahajanga Melaky, Tsingy de bemaraha NP, S19.14114, E044.81245, 45 m.a.o., 14.12. 2009, water net, field, Bergsten et al./NHRS-JKLB 000000495” (1 ex. NHRS); “Toliara Menabe Menabe RS N-19.92773, E045.52253, 102 m.a.o. 10.12. 2009 water net, field Bergsten et al” (1 ex. NHRS); same data, add “000000471 NHRS-JLKB” (1 ex. NHRS); “Tamatava Prov., 3.3 km N Ambabasoratra 31.8. 1962 Cashatt” (1 ex. USNM); “Andohahela NP, Tsimelahy, pools in creek 15-16.11. 2007, 24°57'13.69"S, 46°37'23,30"E Stastny” (1 ex. NHRS); “Reserve de Perinet 29.5. 1991, forestra pluviale, L. Bartolozzi leg.” (2 exs. CSR); “Tamatava Pr. 3 km E Perinet 2.4.1963 Cashatt” (1 ex. NHMB); “Pr. Tananarive, env. Arivonimamo 22.7.1970 Pederzani” (1 ex. NHMB); “Env. Tananarive 7. 1934 Olsoufieff / Lac Tsimbazaza” (1 ex. MNHN); “Pr. Tamatave, Foret de Perinet 17.7. 1970 Pederzani” (1 ex. NHMB); “Tamatave (Toamasina) Park Ivoloina, Pfütze auf Strasse im Wald 21.11. 2000 Dolin” (1 ex. NHRS); “SW Mad. Andranohilany Voeltzkow / *Laccophilus addendus* Sharp det. Brancucci 1982“ (1 ex. NHMB); “Pays Androy nord Alluaud 1900” (2 exs. MNHN); “Vatomandry 8. 1934 Vadon” (1 ex. MNHN).

##### Comments on synonymy.

No justification to distinguish a varation (var. geminatus) to *Laccophilus addendus* has been detected. Accordingly earlier synonymization by Zimmermann (1920) is considered valid.

##### Diagnosis.

*Laccophilus addendus* is characterized by peculiar elytral colour pattern and shape of penis. Elytra with evenly distributed irrorations but at base with irregular longitudinally shaped pale areas. Penis strongly curved and apex evenly broad to abrupt end. Shape of penis is different from all African *Laccophilus* species.

##### Description.

Body length 3.8–4.1 mm, width 2.2–2.3 mm. Habitus and dorsal colour pattern (Fig. 448).

Head: Pale ferrugineous to pale brown. Finely reticulated, slightly mat. Reticulation double; large meshes contain two to six smaller meshes. Sometimes double reticulation in part somewhat indistinct; large meshes strongly reduced, rather indistinct. Punctation extensively absent or indistinct; at eyes fine punctures may be discerned.

Pronotum: Pale ferrugineous. At foremargin (between eyes) dark ferrugineous. Basally at midline with two small dark ferrugineous spots. Submat, finely reticulated and reticulation double. Large meshes, when discernible, contain 2–6 small meshes. Extensively impunctate; frontally fine, rather sparse punctures may be discerned.

Elytra: Pale ferrugineous. Colour pattern consists of extensive dark, irrorations; subbasally dark irrorations sparse forming a pale, transverse, irregular area (Fig. 448). Submat, finely reticulate. Double reticulation rather indistinct; only in part of elytral disc clearly discernible. Almost impunctate; scattered, very fine punctures discernible. Lateral, pre-apical furrow fine, finely pubescent.

Ventral aspect: Pale ferrugineous to ferrugineous. Metacoxal plates somewhat darker; sometimes plates vaguely darker only close to margins. Shiny, submat, very finely reticulated. Basal ventrites with fine, curved striae. Almost impunctate. Prosternal process slender, apex only slightly extended, pointed. Metacoxal plates with shallow, transversely located furrows, which are anteriorly quite distinct, posteriorly indistinct (fade away). Apical ventrite medially keeled; with a small knob on one side (Fig. 108).

Legs: Pro- and mesotarsus slightly enlarged; with suckers. Claws slender, slightly curved and almost equally long.

Male genitalia: Penis in lateral aspect quite slender, medially distinctly bent; extreme apex straight and simple, unmodified. In dorsal aspect penis apex ends abruptly (Figs 297–298).

Female: Apical ventrite medio-apically keeled (Fig. 109). Pro- and mesotarsus slender.

##### Distribution.

Madagascar (Fig. 558). Records from mainland of Africa are considered uncertain.

##### Collecting circumstances.

Insufficiently documented. Sampled at light in tropical dry forest. Also reported from a pond and a muddy waterhole.

#### Laccophilus vermiculosus

Taxon classificationAnimaliaColeopteraDytiscidae

Gerstaecker, 1867

Figs 110–111 , 299–300 , 449–450 , 451 , 559

Laccophilus vermiculosus Gerstaecker 1867: 25 (original description, faunistics); Sharp 1882: 287, 822 (description, faunistics); v. d. Branden 1885: 24 (catalogue, faunistics); Régimbart 1895: 136 (description, faunistics); Zimmermann 1920a: 28 (catalogue, faunistics); Guignot 1946c: 268, 271, 273, 312 (description, faunistics); Guignot 1952c: 521 (faunistics); Omer-Cooper 1956: 21 (faunistics, biology); Omer-Cooper 1957: 14, 90 (discussion, faunistics); Omer-Cooper 1958b: 37, 45, 46 (discussion, description, faunistics); Legros 1958: 211 (faunistics); Guignot 1959a: 557, 559, 560, 562 (discussion, description, faunistics); Guignot 1959d: 161 (faunistics); Omer-Cooper 1965: 77, 83 (description, faunistics); Bertrand and Legros 1967: 862, 867 (faunistics); Bilardo and Pederzani 1978: 119 (faunistics, description); Forge 1981: 500, 501 (description, faunistics); Bilardo and Rocchi 1987: 104 (faunistics, biology); Pederzani and Rocchi 1982: 72 (faunistics); Bilardo and Rocchi 1990: 177 (faunistics); Curtis 1991: 186 (faunistics); Nilsson 2001: 252 (catalogue, faunistics); Bilardo and Rocchi 2002: 174 (list, faunistics); Pederzani and Reintjes 2002: 40 (faunistics); Reintjes 2004: 68 (faunistics); Bilardo and Rocchi 2008: 211, 235 (faunistics); Rocchi 2013: 141 (faunistics); Nilsson 2015: 219 (catalogue, faunistics).
Laccophilus mocquerysi Severin 1892: 472 (nomen nudum); Régimbart 1895: 139 (original description, faunistics); Zimmermann 1919: 122 (faunistics); Zimmermann 1920a: 23 (catalogue, faunistics); Zimmermann 1926: 23 (description, discussion, synonymy, faunistics); Gschwendtner 1935a: 15 (faunistics); Gschwendtner 1938a: 5 (faunistics); Omer-Cooper 1965: 83 (list, synonymy); Nilsson 2001: 252 (catalogue, faunistics, list, synonymy); Nilsson 2015: 219 (catalogue, faunistics, list, synonymy, *Laccophilus vermiculosus* Gerstaecker).
Laccophilus vermiculosus ab. mocquerysi Régimbart, Zimmermann 1926a: 23 (synonym, faunistics); Guignot 1946c: 268, 271 (description, discussion, faunistics); Guignot 1959a: 559, 560 (description, faunistics); Guignot 1959d: 161 (faunistics).
Laccophilus vermiculosus var. mocquerysi Régimbart, Omer-Cooper 1957: 14 (discussion, faunistics). **Confirmed synonym.**

##### Type localities.

*Laccophilus vermiculosus*: Kenya: Mombasa.

*Laccophilus mocquerysi*: Senegal: Dakar.

##### Type material studied

(9 exs.). *Laccophilus vermiculosus*: Lectotype (by present designation): female: “43615 / Typus / Hist.-Coll. (Coleoptera) Nr. 43165 *Laccophilus vermiculosus* Gerst. Mombas v.d. Decken Zool. Mus. Berlin / *vermiculosus* Gerst. Mombas v.d. Decken” (ZMHB). – Paralectotype: female: “Mombasa v.d. Decken Nr. 43615 / Typus / Hist.-Coll. (Coleoptera) Nr. 43165 *Laccophilus vermiculosus* Gerst. Mombas v.d. Decken Zool. Mus. Berlin” (1 ex. ZMHB).

*Laccophilus mocquerysi*: Lectotype (by present designation): male: “Sénégal Dakar A. Moquerys Février 1889 / Museum Paris coll. Maurice Régimbart 1908 / *mocquerysi* Régb.” (MNHN). – Paralectotypes: Senegal: Sama data as lectotype (1 ex. MNHN); “Db / Senegal Dr. Roussel / Museum Paris coll. Maurice Régimbart 1908” (1 ex. MNHN). – Gabon: “Gabon Mocquerys / Museum Paris coll. Maurice Régimbart 1908” (1 ex. MNHN). – Zaire: “Matadi Congo / Museum Paris coll. Maurice Régimbart 1908” (3 exs. MNHN).

##### Additional material studied

(208 exs.). **Senegal**: “Dakar V. 1939 Bouvet” (4 exs. MNHN). – **Sudan**: “Agadi Dar el Fungi Alluaud 1906 / Mares temporaries d’eau de pluie” (3 exs. MNHN); “S. Sudan Alel rock pool 30,56E, 6,11N, 18.1. 1954 JJOC.” (5 exs. AMGS). – **Ivory Coast**: “Comoé Nat. Pk, N 8.5°, W3.5°/11.4. 1999 Temp. Creek leg. Reintjes” (1 ex. NHMW). – **Benin**: “Dep Littoral Cotonou City, pond 8.2.2006 leg. Goergen, Komarek & Houngué” (1 ex. NHMW, 1 ex. MZH). – **Zaire**: “PNA 23.8. 1957 Vanschuytbroeck VS 127a/b/Secteur Nord riv. de Semliki rte Muramba, 905 m” (4 exs. MRAC, 1 ex. MZH). – **Kenya**: “Gulanze Dam, Kwale Distr., 19.9. 1976 Holmen leg.” (7 exs. ZMUC, 1 ex. MZH); “Mafisini Pond, Kwale Distr., 19.9. 1976 Holmen leg.” (4 exs. ZMUC, 1 ex. MZH); “Makalanga Dam, Kwale Distr., 19.9. 1976 Holmen leg.” (2 exs. ZMUC); “Makalanga Dam, Kwale Distr., 19.9. 1976 Holmen leg.” (2 exs. ZMUC); “Rice field, Mwande Dam, Kwale Distr., 19.9. 1976 Holmen leg.” (1 ex. ZMUC); “Maji ya Chumvi Riv., Kwale Distr., 16.9. 1976 Holmen leg.” (1 ex. ZMUC); “Dam at Kaloleni Mission, Kilifi Distr., 15.9.1976 Holmen leg.” (2 exs. ZMUC); “Mombasa / Ch. Alluaud / Museum Paris coll. Maurice Régimbart 1908” (1 ex. MNHN); “Mombasa Ch. Alluaud” (2 exs. MNHN); “Cote d’Afr. Or. Angl. Tiwi Alluaud & Jeannel 1911 St. 5” (1 ex. MNHN). – **Tanzania**: “Lukoka, pond, Tanga Distr., 22.9. 1976 Holmen leg.” (2 exs. ZMUC, 1 ex. MZH); “Narobi b. Tanga 2. 1951 Methner leg.” (1 ex. ZMHB); “DOA Uruba Methner” (1 ex. ZMHB); same but “15.9.” (1 ex. NHMB); “Tandaguru Linndi Dec. 1909- Jan. 1910 Janensch S.G.” (1 ex. ZMHB); “Petukiza, ponds, Tanga Distr., 23.9. 1976 Holmen leg.” (1 ex. ZMUC, 1 ex. MZH); “Ngezani ponds, Tanga Distr., 23.9. 1976 Holmen leg.” (3 exs. ZMUC); “Pongwe, rice field, Tanga Distr., 24.9. 1976 Holmen leg.” (1 ex. ZMUC); “Rice fields of Tanga, Tanga Distr., 26.9. 1976 Holmen leg.” (4 exs. ZMUC); “Tang. Terr. Ukerewe L. Conrad leg.” (1 ex. BMNH); “Zanzibar Pemba 23. September 1955 Fowler” (2 exs. AMGS); “Zanzibar 17.5. 1888” (2 exs. ZMHB); “Zanzib.” (1 ex. ZMHB). – **Angola**: “20 km E Luanda, Luanda-Katete Hwy 9.10. 1949 Malkin / waterhole, gravel and clay bottom” (1 ex. BMNH). – **Malawi**: “River near Portuguese border nr Malanza 4.XI. 1948” (2 exs. AMGS). – **Namibia**: “E. Kapriwi: Katima Mulilo 17°29'S, 24°17'E, 3-8.3. 1992 Uhlig leg.” (1 ex. ZMHB). – **Botswana**: “Tsotsorogo Pan 17.6.-9.7. 1930 / *Laccophilus mocquerysi* Rég. det. Gschwendt.” (1 ex. MNHN, 25 exs. TMSA, 4 exs. MZH); “Serowe sevage ponds, Farmer’s Brigade 1.6. 1987, SE22 26BD Forschhammer leg.” (1 ex. MZH); same data but ”9. 87 / *Laccophilus vermiculosus* Ger. det. Rocchi 1993” (1 ex. CSR). – **Zimbabwe**: “Matopos NP 20°33'S, 28°30E, lux 28.9-1.12. 1993 Uhlig leg.” (28 exs. ZMHB, 4 exs. MZH; habitus in Fig. 449–451); “S Rhod. Pool Lundi 22. N. 1948 JOC.” (1 ex. AMGS); “Wankie Game Reserve, Shapi Pan 5.9. 1948 JOC.” (2 exs. AMGS, 1 ex. TMSA); “Wankie Game Reserve, Sept. 1948 water holes JOC.” (3 exs. AMGS); “Wankie Game Reserve Masumu Dam 4.9. 1948” (10 exs. AMGS, 1 ex. BMNH); “Wankie Game Reserve 5. September 1948 JOC. Pools at Robins restcamp” (2 exs. AMGS); “Wankie Game Res., waterholes nr head-quarter 2.9. 1948” (2 exs. AMGS); “Wankie Nat. Pk, Pan 0-8 M. V. light trap 9. 1961 Weir leg.” (1 ex. BMNH); “5 mi SE Wankie 7.4. 1968 Spangler” (3 exs. USNM); “Gwai River 3.4. 1968 Spangler” (13 exs. USNM, 4 exs. MZH). – **Mozambique**: “Beira 7 September 1955 JOC.” (1 ex. AMGS); “Moz. Dambo Pan 30.6. 1960” (1 ex. AMGS). – **South Africa**: “Kruger Nat. Pk, Skukuza 12 km S, 25.04S, 31.37E / 6.3. 1996 UV light leg. Endrödy-Younga” (17 exs. TMSA, 3 exs. MZH); “Kruger Nat. Pk, Malonga Springs 22.36S, 31.20E / 8.2. 1994 shorewashing leg. Endrödy-Younga” (1 ex. TMSA); “Kruger Nat. Pk, Punda Maria Ngots Dam 21.26S, 31.14E / 7.2. 1994 shorewashing leg. Endrödy-Younga” (1 ex. TMSA); “Transvaal small cattle dam Louis Trichart 23.III. 1948 JOC.” (1 ex. AMGS); “Transvaal Kruger Park 29.VI. 1960” (1 ex. AMGS); “Kw. Natal, Lake Nhlange, Kosi lake compl. N-27.00, E32.50, 27.1. 1967 Allanson” (1 ex. AMGS); “Natal Crocodile R. 5.V. 1956” (1 ex. AMGS).

##### Specimen with collecting site unknown.

“Magude 16.8. 1915 C.J. Sw. / *Laccophilus adspersus* Boh. det. Gschwendtner” (1 ex. TMSA).

##### Comments on synonymy.

Synonymy of *Laccophilus vermiculosus* and *Laccophilus mocquerysi* was suggested the first time by Zimmermann (1926), when he placed *Laccophilus mocquerysi* as an aberration of *Laccophilus vermiculosus*. Examination of type material of both taxa confirms earlier synonymization.

##### Diagnosis.

*Laccophilus vermiculosus* is especially characterized by comparatively large body size, peculiar, slightly variable, elytral colour pattern and shape of penis; curved and apex with a peculiar, bifid projection. Not to be confused with any recognized African *Laccophilus* species.

##### Description.

Body length 4.6–5.1 mm, width 2.4–2.8 mm. Dorsal colour pattern generally quite stable, exhibiting only minor variation Elytral irroration sometimes somewhat reduced but exhibits still original, ground-plan pattern (Figs 449–451). Dark area on head and pronotum may rarely be reduced.

Head: Pale ferrugineous, posteriorly close to pronotum with blackish to dark ferrugineous, well-delimited area. Dark area rarely somewhat vague or in part hidden under foremargin of pronotum. Submat, finely microsculptured. Reticulation double, but difference between size-categories slight; in part large meshes indistinct or non-discernible. Large meshes, when discernible, contain 2–5 small meshes. Impunctate, except close to eyes; with some, scattered, fine punctures. Punctate areas extend towards middle of head-discussion

Pronotum: Pale ferrugineous; at foremargin and basally in middle with narrow blackish ferrugineous to dark ferrugineous marking. Discally, often with a more or less well-delimited, dark spot, which is often bilobed. Submat, finely microsculptured. Reticulation double but size-categories often difficult to distinguish. Large meshes, when discernible, contain 3–7 small meshes. Frontally and laterally with fine, slightly indistinct, scattered punctures.

Elytra: Pale ferrugineous, with distinct, slightly variable, blackish ferrugineous to dark ferrugineous irrorations (Figs 449–451). Irrorations may sometimes be somewhat reduced but colour pattern still exhibits same, basic organization. Submat, finely but rather distinctly microsculptured. Reticulation extensively double; laterally and posteriorly separation of size-categories difficult. Large meshes, when discernible, contain 3–5 small meshes. Fine, slightly irregular punctures form a discal row. Dorsolateral and lateral rows only indicated by a few, scattered punctures. Pre-apically, fine, haired punctures form a short, shallow, lateral furrow.

**Figures 110–119.**
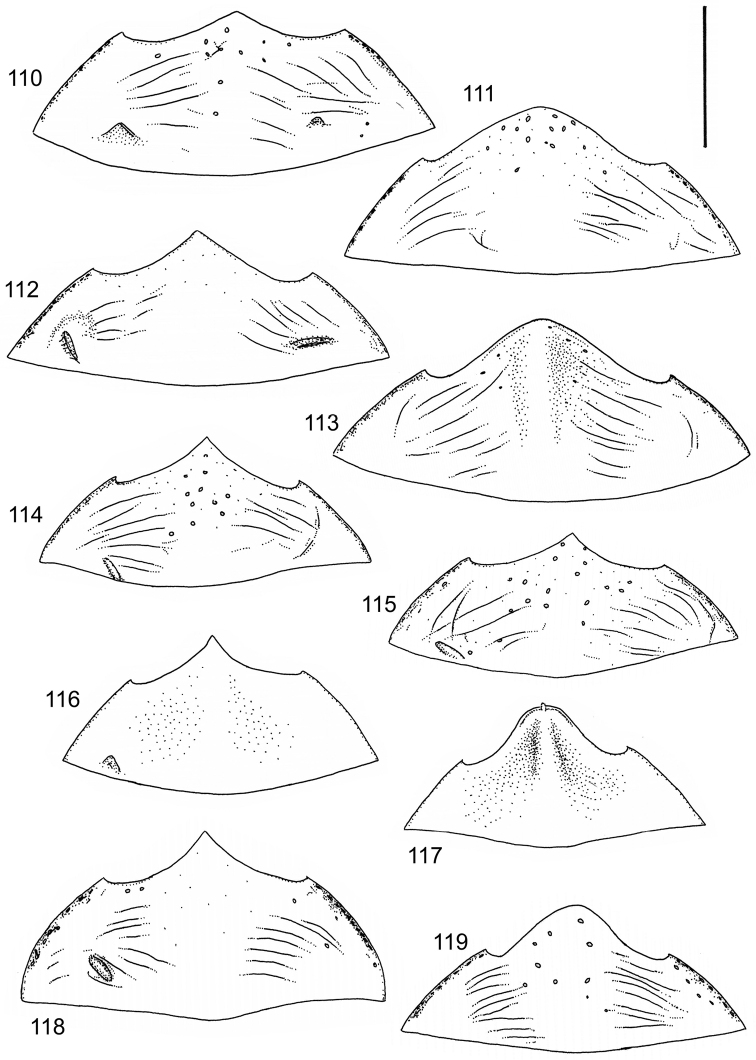
Apical ventrites **110** *Laccophilus vermiculosus* male, and **111** female **112** *Laccophilus guignoti* male, and **113** female **114** *Laccophilus guentheri* male **115** *Laccophilus guineensis* male **116** *Laccophilus bizonatus* male, and **117** female **118** *Laccophilus pulcher*, male, and **119** female. Scale bar 0.5 mm.

Ventral aspect: Pale ferrugineous to ferrugineous; no distinct colour pattern formed. Rather shiny, with very fine, in part indistinct microsculpture. Abdomen with curved fine striae. Almost impunctate. Metacoxal plates with about ten transversely located shallow furrows. Prosternal process rather slender, apex somewhat extended, pointed. Metacoxal process area does not exhibit any modifications. Apical ventrite asymmetric, with distinct lateral knob (Fig. 110).

Legs: Pro- and mesotarsus slightly enlarged and extended, with distinct suckers.

Male genitalia: Shape of penis characteristic; somewhat curved and apex with a peculiar, bifid projection (Figs 299–300).

Female: Apical ventrite almost symmetric; lacks lateral knob (Fig. 111). Pro- and mesotarsus slender, slightly extended.

##### Distribution.

Senegal, Sudan, Ivory Coast, Benin, Gabon, Zaire, Kenya, Tanzania, Angola, Malawi, Namibia, Botswana, Zimbabwe, Mozambique, South Africa (Fig. 559). Omer-Cooper (1958b) gives Uganda and Ghana and Legros (1958) Guinea. Finally Omer-Cooper (1965) adds Somalia, and Pederzani and Rocchi (1982) former French Congo.

##### Collecting circumstances.

Rather insufficiently documented. According to label data the species is collected in rice fields and various kinds of ponds, dams and pools. In Angola collected from a waterhole with clay and gravel bottom. Sometimes sampled at light collection (e.g. UV). Literature records are scarce; Omer-Cooper (1958b) superficially describes some sampling sites at streams, ponds, pools in river banks etc.

#### Laccophilus guignoti

Taxon classificationAnimaliaColeopteraDytiscidae

Legros, 1954

Figs 112–113 , 301–302 , 452 , 554

Laccophilus guignoti Legros 1954: 268 (original description, faunistics); Guignot 1955b: 1096 (faunistics); Nilsson 2001: 244 (catalogue, faunistics); Nilsson 2015: 212 (catalogue, faunistics).

##### Type locality.

Guinea: Mont Tò, Nimba.

##### Type material studied

(3 exs.). Holotype: male: “Mont Tò 1600 m, Camp I / Muséum Paris Nimba (Guinée) M. Lamotte II. VI. 42 / Type / *Laccophilus guignoti* sp. n. C. Legros det.” (MNHN). – Paratypes: “Mt Tò (1600 m) Camp 1 / Muséum Paris Nimba (Guinée) M. Lamotte II. VI. 42 / Allotype / *Laccophilus guignoti* Legros” (1 ex. MNHN); same data as preceeding, but labelled as “cotype” (1 ex. MNHN).

##### Additional material studied

(3 exs.). **Guinea**: “Exped. Mus. G. Frey Franz. Guinea 1951 W. Afr. leg. Bechyne / Région Kindia Mt. Gangan 600 m 18.5. 51 /male symbol” (1 ex. MNHN; habitus in Fig. 452); “5 km N de Madina-Salambande 11–12.7. 2004 Kudrna” (2 exs. CFP).

##### Diagnosis.

*Laccophilus guignoti* is particularly characterized by large sized body, peculiar elytral colour pattern and female, having expanded epipleura posterior to middle (expansions visible from above). Male genitalia is also characteristic and differs from other *Laccophilus*; penis strongly curved in lateral view and apex broad, of almost equal width except for basal part which is a little broader. Closest relative may be *Laccophilus irroratus*. Regarding external colour pattern (e.g. robust irroration) there is some resemblance with species, here placed in Species group 11 (*deceptor*). Final location of *Laccophilus guignoti* remains an open question, which need further study to be solved.

##### Description.

Body length 4.3–4.9 mm, width 2.5–2.9 mm. Dorsal colour pattern of body rather distinct; elytra with irrorations; at base and preapically with transverse, pale areas where irrorations in part absent or sparser (Fig. 452).

Head: Pale ferrugineous. At eyes with a few, scattered, fine punctures. Submat, with distinct and dense microsculpture. Reticulation double. Coarse meshes distinct, contain 2–4 fine meshes. In part, fine meshes indistinct or obliterated.

Pronotum: Pale ferrugineous to ferrugineous. Frontally in middle with a dark ferrugineous area; basally in middle, narrowly, dark ferrugineous (basal, dark area sometimes, somewhat enlarged). Anteriorly, very fine, scattered punctures may be discerned. Submat, distinctly microsculptured. Reticulation double; coarse meshes distinct but rather small. Coarse meshes contain, in general, 2–4 fine meshes. Fine meshes in part not discernible at all. Large meshes become “crowded” laterally.

Elytra: Pale ferrugineous to ferrugineous, with undulate, dark ferrugineous markings; colour pattern slightly variable (Fig. 452). Discally, dorsolaterally and laterally with scattered, sparse and fine punctures, which form longitudinally located, indistinct rows. Submat, with dense and distinct microsculpture. Reticulation double. Coarse meshes contain 2–5 fine meshes. Laterally coarse and fine meshes appear almost equal in strength and therefore difficult to distinguish. Epipleuron posterior to middle not enlarged.

Ventral aspect: Pale ferrugineous to ferrugineous, distinct colour pattern absent. Impunctate. Slightly mat, due to very fine microsculpture. Ventrites with curved striae. Prosternal process rather slender, apex extended and pointed. Metacoxa with approximately 10 very shallow, in most cases, transversely located furrows. Metacoxal process-area of usual appearance. Apical ventrite with a sharp keel on each side (Fig. 112).

Legs: Pro- and mesotarsus rather slender, somewhat extended, first to third segment provided with a few, protruding suckers.

Male genitalia: Penis in lateral aspect strongly curved and extreme apex broad, truncate, and almost equally broad in comparison with basal part of penis (Figs 301–302).

Female: Epipleuron posterior to middle for a short distance enlarged. Apical ventrite lacks lateral keels; as in Fig. 113.

##### Distribution.

Guinea (Fig. 554).

##### Collecting circumstances.

Unknown.

### Species group 11 (*Laccophilus deceptor* group)

**Diagnosis.** Medium to large-sized species; length of body 3.5–4.5 mm, width 1.9–2.5 mm.

Body shape oblong to oval, dorsoventrally flattened. All species with distinct colour pattern. Elytra with dark ferrugineous to blackish irroration, which is quite robust and often vague. In some species irroration almost entirely merged to extensive areas of dark colour. All species also exhibit pale patches, which in many species are arranged in more or less distinct transverse series (Figs 453, 456, 462). Body microsculpture double, but large meshes are often in part reduced and indistinct. Fragments of microsculpture-meshes generally discernible.

Prosternal process slender, apically extended and pointed. Apical ventrites modified; posterior part on both side excavated and medial part forms a backwards extending process. Apical ventrite provided with asymmetrical knob on one side of ventrite (Figs 118, 124). Metacoxal plates lack stridulatory apparatus. Metacoxal process ends abruptly; lacks posterior extension (Fig. 6).

Paramere rather simple, apically enlarged but not strongly modified (Fig. 315). Penis often strongly modified with external outline almost straight to undulate. Penis sometimes robust and voluminous (Figs 305, 313, 318).

**Species composition and distribution.** 12 species are recognized in this species group. All of them are distributed in mainland Africa, south of Sahara.

#### Key to species (with one exception only applicable for males)

**Table:** 

1	Small species, body length 3.5 mm (male unknown)	***Laccophilus caiaricus*** (p. 175)
-	Larger species, body length 3.6–4.5 mm	**2**
2.	Penis, lateral aspect, external outline evenly to slightly unevenly curved (Figs 303, 306)	**3**
-	Penis, lateral aspect, external outline strongly undulate or otherwise modified (Figs 317, 318)	**6**
3	Base of elytra pale (at base with transverse area lacking dark spots or dark, transverse area) (Fig. 453)	**4**
–	Base of elytra with distinct, dark, transverse area (Fig. 455)	**5**
4	Apex of penis narrow; extreme apex with a minor hook (Fig. 303)	***Laccophilus guentheri*** (p. 160)
–	Apex of penis broad; extreme apex not hooked (Fig. 305)	***Laccophilus guineensis*** (p. 161)
5	Pronotum blackish to dark ferrugineous; posterior to eyes with small, pale spots (Fig. 457); penis, lateral aspect, external outline in apical half almost straight (Fig. 307)	***Laccophilus pulcher*** (p. 164)
–	Pronotum almost entirely pale ferrugineous; mediobasally with broad but narrow, vague, dark ferrugineous marking (Fig. 455); penis, lateral aspect, external outline in apical half slightly curved (Fig. 306)	***Laccophilus bizonatus*** (p. 163)
6	Penis, lateral aspect, apically strongly enlarged, forming a triangular part (Fig. 320)	***Laccophilus persimilis*** (p. 174)
–	Penis, lateral aspect, shape different, never with triangular part (Figs 309, 318)	**7**
7	Large species, length 4.2–4.5 mm	**8**
–	Smaller species, length 3.6–4.0 mm	**10**
8	Pronotum blackish to dark ferrugineous, posterior to eyes with yellow, somewhat vague spot (Fig. 462)	***Laccophilus decorosus* sp. n.** (p. 171)
-	Pronotum pale, medio-basally often with a slightly vague, dark marking (Fig. 460)	**9**
9	Penis, lateral aspect, robust and strongly modified (Fig. 318)	***Laccophilus tschoffeni*** (p. 172)
–	Penis, lateral aspect, delicate and modified (Figs 313–314)	***Laccophilus deceptor*** (p. 168)
10	Pronotum blackish to blackish ferrugineous, posterior to eyes with yellow, somewhat vague spot (Fig. 461)	***Laccophilus bilardoi*** (p. 170)
–	Pronotum pale ferrugineous, medio-basally sometimes with a somewhat vague marking (Fig. 458)	**11**
11	Penis apex truncate (Fig. 311)	***Laccophilus biai*** (p. 167)
–	Penis apex pointed, apex slightly curved (Fig. 309)	***Laccophilus concettae*** (p. 165)

#### Laccophilus guentheri sp. n.

Taxon classificationAnimaliaColeopteraDytiscidae

http://zoobank.org/E04144B3-F345-4911-86B9-826AE71334FD

Figs 114 , 303–304 , 453 , 561

##### Type locality.

Ghana: Ashanti Reg, Kumasi, Nhiasu (N6°43' – W1°36').

##### Type material studied

(2 exs.). Holotype: male: “Ghana: Ashanti Region, Kumasi, Nhiasu 330 m, N6°43' – W1°36' Dr. S. Endrödy-Younga / Nr. 225, at light 12.VI. 1967” (CGW; habitus in Fig. 453). – Paratype, male: “Rep. Guinea Seredoux, lux 7-8.4.1975 leg. Zott” (1 ex. ZMHB).

##### Diagnosis.

*Laccophilus guentheri* has a broad, pale ferrugineous, basal area on elytra lacking dark spots or areas, and resembles in this respect of *Laccophilus guineensis*. The shape of male genitalia deviates, however, very strongly. Apical half of *Laccophilus guentheri* penis is, very characteristic and separates it from the other species recognized in this species group; apex of penis narrow, extreme tip provided with a minute hook,

##### Description.

Body length 3.6–3.8 mm, width 2.0–2.1 mm. Dorsal colour pattern distinct; elytra basally with broad, pale area followed by a uniform, dark ferrugineous to brownish area, which towards apex dissolves into fairly robust, pale ferrugineous irrorations (Fig. 453).

Head: Pale ferrugineous. Slightly mat, finely microsculptured. Reticulation double; larger meshes contain generally 3–6 small meshes. Impunctate, except at eyes; there with fine, irregularly distributed punctures. Area of punctures continues a short distance towards middle of head-disc as a slightly irregular row of punctures.

Pronotum: Pale ferrugineous. Submat, finely microsculptured. Reticulation double; large meshes moderately stronger developed than fine meshes, contain generally 3–6 fine meshes. Impunctate, except laterally and anteriorly; here with fine and somewhat irregularly distributed punctures.

Elytra: Pale ferrugineous with dark ferrugineous to brownish colour pattern (Fig. 453). Submat, finely microsculptured. Reticulation double but large meshes in part, strongly reduced and indistinct. A distinct and discernible discal row of punctures formed by very fine, somewhat irregular punctures. Dorsolateral and lateral rows of punctures are somewhat indistinct, because weakly developed and mixed with scattered, fine punctures. Pre-apical lateral row of punctures sparse, finely pubescent.

Ventral aspect: Dark ferrugineous to ferrugineous, no distinctly delimited colour pattern. Prosternal process rather slender, apically extended, pointed. Ventral surface almost impunctate; apically on abdomen with some scattered punctures. Metacoxal plates in anterior half with some 5–6 shallow and in part reduced furrows. Posterior half of metacoxal plates smooth; furrows absent. Abdomen with fine, somewhat curved striae. Apical ventrite asymmetric; with one lateral knob (Fig. 114)

Legs: Pale ferrugineous to ferrugineous. Pro- and mesotarsus somewhat enlarged, provided with suckers.

Male genitalia: Penis in lateral aspect somewhat curved, apical half narrow, peculiarly folded and extreme tip provided with a minute hook (Figs 303–304).

Female: Unknown.

##### Etymology.

The name is a noun in its genitive form based on the name of Prof. Dr. Günther Wewalka, Vienna, who sent the holotype to us for examination.

##### Distribution.

Guinea, Ghana (Fig. 561).

##### Collecting circumstances.

Almost unknown. Collected at light.

#### Laccophilus guineensis sp. n.

Taxon classificationAnimaliaColeopteraDytiscidae

http://zoobank.org/29EC75C3-5F13-4530-B8E0-85F0BBB14B70

Figs 115 , 305 , 454 , 562

##### Type locality.

Guinea: Seredou.

##### Type material

(3 exs.). Holotype: male: “Guinea Seredou, lux, 5.4. 1975 leg. Zott” (ZMHB). – Paratypes: Same data as holotype but “4.4. 1975” (1 ex. MZH); “Rep. Guinea Sérédou, lux, 4. Apr. 1975 leg. A. Zott” (1 ex. ZMHB; habitus in Fig. 454).

##### Diagnosis.

Closely related to *Laccophilus guentheri* and *Laccophilus bizonatus*; characterized by quite similar, elytral colour pattern. *Laccophilus guineensis* is separated from them by having a robust penis, the apical half of which is broad, simple and moderately modified; extreme apex formed as a broad, rounded enlargement; not hooked.

##### Description.

Body length 3.9 mm, width 2.1 mm. Dorsal, aspect of body with stable colour pattern (Fig. 454).

Head: Pale ferrugineous to ferrugineous. Rather shiny, although finely microsculptured. Reticulation double; large meshes in part reduced and only slightly more strongly developed in comparison with fine meshes. Impunctate, except at eyes with a group of irregularly distributed, fine punctures. Area of punctures extended a short distance towards middle of head-disc.

Pronotum: Pale ferrugineous, almost unicoloured. Narrowly darker at foremargin. Basally at moderate distance from midline with two small and vague, dark ferrugineous spots. Submat, finely microsculptured. Reticulation double; large meshes only slightly more strongly developed than small meshes. Large meshes contain, when discernible, 2-6 small meshes (sometimes number difficult to estimate). Impunctate, except laterally and at foremargin; here fine, irregular punctures discernible.

Elytra: Pale ferrugineous, with irregularly distributed, dark ferrugineous irrorations. Frontally at pronotum dark irrorations absent, forming an irregular, pale, transverse area. Posterior to pale area dark irrorations thickened, forming a dark transverse area. Posterior to dark area, irrorations somewhat loosened and appear somewhat irregular (Fig. 454). Rather shiny, although finely microsculptured; reticulation double. In frontal part of elytra division into two mesh-size-categories is clearly discernible. Posteriorly division becomes vague and indistinct. Impunctate, except for three, vague, longitudinal rows of punctures. Pre-apical, lateral row of fine punctures located in shallow furrow, which is finely pubescent.

Ventral aspect: Dark ferrugineous to ferrugineous, no distinct colour pattern. Prothorax somewhat paler, ferrugineous to pale ferrugineous. Almost impunctate; apical ventrite with some punctures. Rather shiny, although finely microsculptured. Prosternal process rather slender; posteriorly somewhat elongated, apically pointed. Metacoxal plates in posterior half smooth; anteriorly with fine, shallow, in part reduced, transversely located furrows. Rather shiny, although very finely microsculptured. Abdomen with fine, curved striae. Apical ventrite as in (Fig. 115); asymmetric knob hardly discernible; rudimentary.

Legs: Pale ferrugineous to ferrugineous. Pro- and mesotarsus slightly enlarged, provided with distinct suckers.

Male genitalia: Penis in lateral aspect curved; extreme apex formed as a broad, rounded enlargement; not hooked (Fig. 305).

Female: Unknown.

##### Etymology.

The species name *guineensis* is an adjective meaning “from Guinea”, the country from where, the new species is described.

##### Distribution.

Guinea (Fig. 562).

##### Collecting circumstances.

Almost unknown. Collected at light.

#### Laccophilus bizonatus

Taxon classificationAnimaliaColeopteraDytiscidae

Régimbart, 1895

Figs 116–117 , 306 , 455–456 , 561

Laccophilus bizonatus Régimbart 1895: 143 (original description, faunistics); Zimmermann 1920a:17 (catalogue, faunistics); Zimmermann 1926: 24 (discussion); Omer-Cooper 1956: 21, 23 (faunistics, biology); Guignot 1959a: 533, 536, 537 (description, faunistics); Bilardo and Pederzani 1978: 118 (faunistics, description); Bilardo and Rocchi 1990: 161, 176, 177, 187 (faunistics, description, biology); Bilardo and Rocchi 1999: 232 (faunistics); Nilsson 2001: 241 (catalogue, faunistics); Bilardo and Rocchi 2002: 155, 160, 174 (faunistics, list); Bilardo and Rocchi 2004: 286 (discussion); Bilardo and Rocchi 2008: 210, 234 (faunistics, biology); Bilardo and Rocchi 2011: 194 (faunistics, biology); Bilardo and Rocchi 2013: 141 (faunistics, biology); Nilsson 2015: 2409 (catalogue, faunistics).

##### Type locality.

Gabon: Riv. N’Gounié, Chutes de Samba.

##### Type material studied

(3 exs.). Lectotype (by present designation): male: “Chutes de Samba Riv. N’Gounié Mocuerys / Type / Régimbart det., 1895 *Laccophilus bizonatus* Rég.” (IRSNB; habitus in Fig. 455). – Paralectotypes: Same data but also *“Laccophilus bizonatus* Rég. type” (1 ex. IRSNB); “Gabon Mocquerys / Museum Paris coll. Maurice Régimbart 1908 / *bizonatus* Rég.” (1 ex. MNHN).

##### Additional material studied

(23 exs.). **Cameroon**: “Ekiliwindi 19.3. 1970” (17 exs. NHMB, 4 exs. MZH; habitus in Fig. 456). – **Gabon**: “Lambarènè 7.8. 1973 Bilardo & Pederzani / *Laccophilus bizonatus* Régb. det. Bilardo” (1 ex. CSR); “Belinga 12.5. 1963 Coiffait” (1 ex. NHMB).

##### Diagnosis.

*Laccophilus bizonatus* is especially characterized by peculiar shaped penis; penis medially twisted and somewhat curved; forwards from middle almost straight and extreme apex distinctly hooked.

##### Description.

Body length 3.6–4.2 mm, width 2.0–2.3 mm. Dorsal, colour pattern of body fairly distinct; some variation often observed (Figs 455–456).

Head: Pale ferrugineous. Submat, finely microsculptured; reticulation double. Large meshes contain generally 4–6 smaller meshes. Almost impunctate; at eyes with fine punctures.

Pronotum: Pale ferrugineous; mediobasally and frontally (area between eyes) with a vague ferrugineous to dark ferrugineous marking. Rather shiny to submat. Reticulation fine, double. In particular medially, smaller meshes rather indistinct. Almost impunctate.

Elytra: Dark ferrugineous. Subbasal, transverse area provided with well-delimited pale ferrugineous, longitudinal spots; separate spots often in part confluent. Elytra posteriorly with rather sparse irrorations, which in part are rather indistinct (Figs 455–456). Submat, reticulation double. Large meshes extensively weakly developed and indistinct. Small meshes in general distinct. Punctation rather indistinct; laterally and discally with vague longitudinal area where punctures are discernible. Lateral, pre-apical furrow fine, finely pubescent.

Ventral aspect: Ferrugineous to dark ferrugineous, prothorax extensively pale ferrugineous. Apex of prosternal process narrow, pointed. Rather shiny, very finely and indistinctly microsculptured. Basal ventrites with fine, curved striae. Metacoxal plates in anterior half with transversely located, shallow furrows; in posterior half furrows absent. Almost impunctate. Apical ventrite asymmetric; with a basal, right-hand, located process/knob (Fig. 116).

Legs: Pro- and mesotarsus slightly enlarged, with suckers.

Male genitalia: Penis in lateral aspect, medially twisted and somewhat curved; forwards from there almost straight and extreme apex distinctly hooked (Fig. 306).

Female: Apical ventrite simple (Fig. 117). Pro- and mesotarsus slender.

##### Distribution.

Cameroon, Gabon (Fig. 561).

##### Collecting circumstances.

Detailed information unknown. Some sampling localities in Gabon are described in Bilardo and Rocchi (1990, 2008, 2011 and 2013) and they give a general picture of the sites but not details of how *Laccophilus bizonatus* occurred in them.

#### Laccophilus pulcher

Taxon classificationAnimaliaColeopteraDytiscidae

Bilardo & Rocchi, 2004

Figs 118–119 , 307–308 , 457 , 562

Laccophilus pulcher Bilardo and Rocchi 2004: 286, 290, 291 (original description, faunistics); Bilardo and Rocchi 2008: 211, 234 (faunistics, biology); Bilardo and Rocchi 2013: 141 (faunistics); Nilsson 2015: 216 (catalogue, faunistics).

##### Type locality.

Gabon: Riv. Louayé, Mékambo, Malouma (0.36N, 13.50E).

##### Type material studied

(2 exs.): Holotype: male: “Gabon Mékambo (Malouma) Riv. Louayé (Borne) 24/07/01 A. Bilardo / 4.12 x 2.24 / Holotypus / *Laccophilus pulcher* Bil. & Roc. Det. Bilardo 04” (MSNM). – Paratype: female: “Gabon, VIII. Bissok (Oyem) F. Lara 8/8/91 A. Bilardo / 4,00 x 2,24 / Paratypus / *Laccophilus pulcher* Bil. & Roc. Det. Bilardo 04” (1 ex. MSNM; habitus in Fig. 457).

##### Notes on taxonomy.

Reflecting solely at the shape of penis *Laccophilus pulcher* resembles much of *Laccophilus saegeri*, in this revision placed in another species group (see Figs 283–284). The penis of *Laccophilus saegeri* is, however, more evenly curved than in *Laccophilus pulcher*. Moreover, elytra are extensively dark coloured in *Laccophilus pulcher*, while they are pale ferrugineous and provided with irrorations in *Laccophilus saegeri*. Similarity in male genitalia between these two species still indicates need of further study in the delimitation of species groups in *Laccophilus*.

##### Diagnosis.

*Laccophilus pulcher* is easy to distinguish by study of male genitalia. No other species in this species group has a penis of same type; penis in lateral aspect long, external outline curved, somewhat undulate. Extreme apex with a fine hook.

##### Description.

Body length 4.0–4.1 mm, width 2.2 mm. Dorsal, colour pattern of body exhibits only minor variation (Fig. 457).

Head: Pale ferrugineous. Rather shiny, although finely microsculptured; reticulation double but difference between size classes minimal. Large meshes extensively hardly discernible. At eyes with very fine, irregular punctures.

Pronotum: Ferrugineous to dark ferrugineous, with pale ferrugineous anterior corners. Rather shiny, although distinctly microsculptured. Reticulation double but difference between size classes small. Large meshes, when discernible, contain 2–4 small meshes. At margin, except basally in the middle, with fine, scattered punctures.

Elytra: Blackish ferrugineous to dark brown, with a subbasal, transversely located, pale ferrugineous markings. Furthermore, with a transverse, pale area discernible posterior to middle. Additionally, extreme apex of elytra pale (Fig. 457). Rather shiny, although finely microsculptured. Reticulation double; large meshes strongly reduced and only in part discernible. Laterally and apically almost solely with fine meshes. Discal row of punctures formed by fine, somewhat irregular punctures. Dorsolateral and lateral rows of punctures indicated by sparse, scattered punctures. Laterally, with a pre-apical, finely pubescent furrow.

Ventral aspect: Ferrugineous to dark ferrugineous; no distinct colour pattern discernible. Rather shiny, although finely microsculptured. Abdomen with fine, curved striae. Prosternal process slender, apex extended, pointed. Metacoxal plates in anterior half with 8–9, almost transversely located, shallow furrows; in posterior half furrows strongly reduced and only minor fragments discernible. Apical ventrite asymmetric, with lateral keel (Fig. 118).

Legs: Pro- and mesotarsus rather slender, somewhat extended, provided with suckers.

Male genitalia: Penis in lateral aspect long, external outline curved, somewhat undulate. Extreme apex finely hooked (Figs 307–308).

Female: Pro- and mesotarsus slender. Apical ventrite symmetric, no lateral keel (Fig. 119).

##### Distribution.

Gabon (Fig. 562).

##### Collecting circumstances.

Almost unknown. Some information may be found in available literature (e.g. Bilardo and Rocchi 2004, 2008): *Laccophilus pulcher* was collected in puddles of a residual stream in forest. It is also listed as a general forest species by the present authors.

#### Laccophilus concettae

Taxon classificationAnimaliaColeopteraDytiscidae

Pederzani, 1983

**Figures 120–129.**
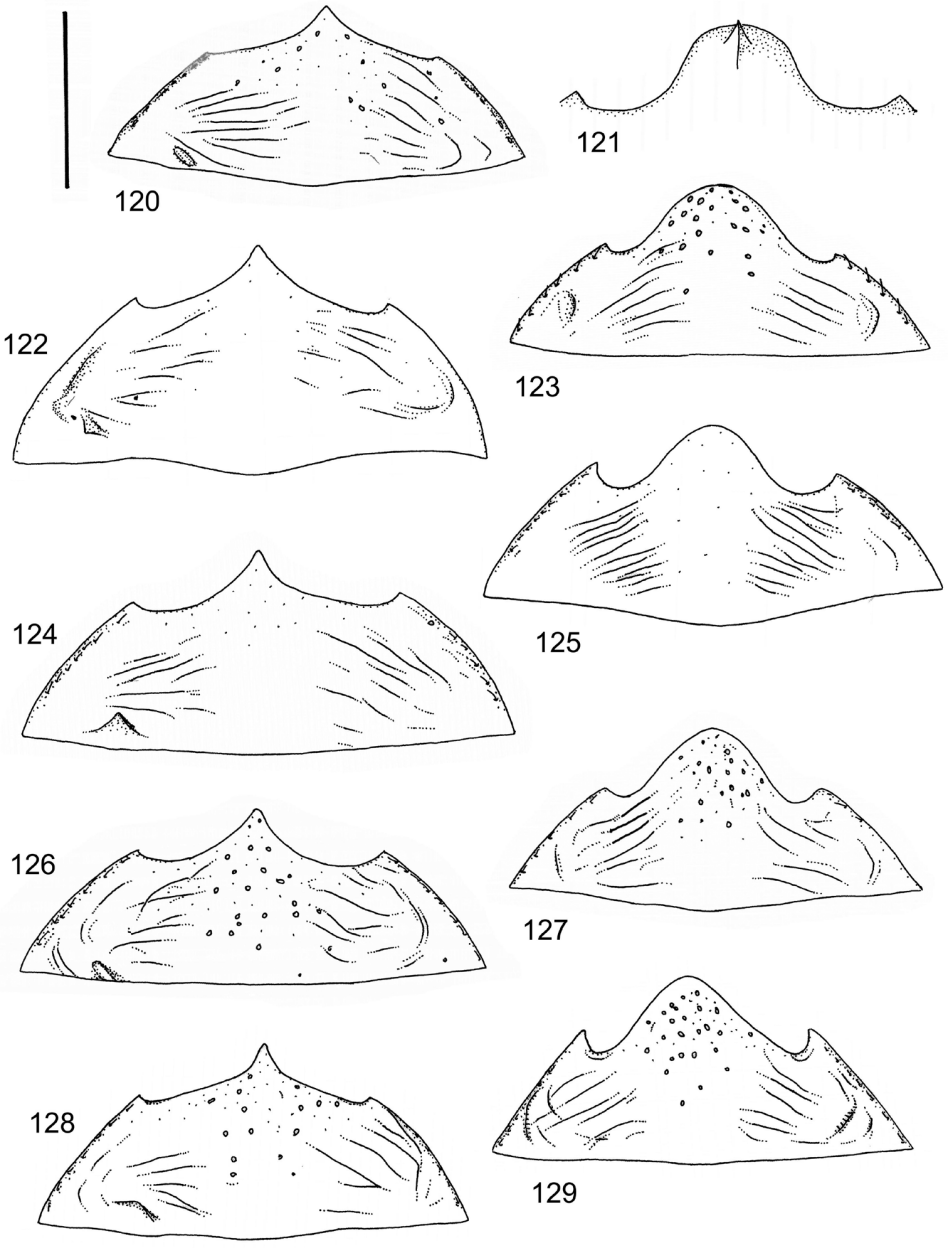
Apical ventrites **120** *Laccophilus concettae* male, and **121** female (according to Pederzani 1983) **122** *Laccophilus biai* male, and **123** female **124** *Laccophilus deceptor* male, and **125** female **126** *Laccophilus bilardoi* male, and **127** female **128** *Laccophilus decorosus* male, and **129** female. Scale bar 0.5 mm.

Figs 120–121 , 309–310 , 458 , 563

Laccophilus concettae Pederzani 1983: 139 (original description, faunistics); Bilardo and Rocchi 1990: 161, 176, 177 (faunistics, description, biology); Leonardi et al. 1995: 192 (list, types in MSMN); Bilardo and Rocchi 1999: 232 (faunistics); Nilsson 2001: 242 (catalogue, faunistics); Bilardo and Rocchi 2002: 174 (list, faunistics); Bilardo and Rocchi 2004: 286, 291 (discussion, faunistics); Bilardo and Rocchi 2006: 129 (faunistics); Nilsson 2015: 210 (catalogue, faunistics).

##### Type locality.

Central African Republic: Zomea at de Mbaiki.

##### Type material studied

(8 exs.). Holotype: male: “R. Centraficaine Zomea près de Mbaiki 29-31.XII. 1980 leg. G. Onore / *Laccophilus concettae* sp. n. det. Pederzani /Holotypus” (MSNM ). – Paratypes: Same data as in holotype but labelled “Paratypus” (7 exs. CFP).

##### Additional material studied

(14 exs.). **Central African Republic**: Same data as holotype (2 exs. NHMB; habitus in Fig. 458). – **Cameroon**: “Foumbot, March 67/B. de Miré” (1 ex. NHMB); “Ebolowa 3.6. 1966 / B. de Miré” (1 ex. NHMB); “Abong-Mbang N. 1966 / B. de Miré” (1 ex. NHMB); “Ngoundéré 14.9. 1969” (1 ex. NHMB). – **Equatorial Guinea**: “Nkolentangan 11. 07-5. 08 G. Tessmann S.G.” (4 exs. ZMHB, 1 ex. MZH). – **Gabon**: “Mékambo, vill. Malouma, rivière Louaié 24.7. 2001 A. Bilardo / *Laccophilus concettae* Ped. det. S. Rocchi 2002” (3 exs. CSR).

##### Diagnosis.

The species *Laccophilus biai*, *Laccophilus deceptor*, *Laccophilus bilardoi*, *Laccophilus decorosus* and *Laccophilus concettae* have a similar groundplan on their male genitalia. Their penises, in lateral aspect have strongly curved, undulate external outline; penis being also quite slender posterior to middle. *Laccophilus concettae* is characterized by dark elytra with pale transverse area posterior to base and apical part of elytra provided with pale, irregular and sparse irrorations. Apex of penis is obtuse in *Laccophilus concettae*. See also diagnosis of *Laccophilus biai* (p. 167).

##### Description.

Body length 3.7–3.9 mm, width 2.0–2.2 mm. Dorsal, colour pattern of body distinct, rather uniform, exhibits only minor variation (Fig. 458).

Head: Pale ferrugineous. Rather shiny although finely microsculptured; reticulation double. Difference between mesh-size categories rather indistinct; large meshes only slightly more strongly developed than small meshes. In part meshes rather indistinct and reduced. When discernible, large meshes may contain 2–6 small meshes. Almost impunctate, except at eyes, with fine, irregular punctures. Area of punctures extends towards middle of head-discussion

Pronotum: Pale ferrugineous. Anteriorly, with vague, slightly darker area. Basally, with a vague dark ferrugineous area. Rather shiny, although finely microsculptured; reticulation double. Large meshes slightly more strongly developed in comparison with small meshes. Large meshes contain 2–6 small meshes. Discally meshes in part slightly reduced and indistinct. Almost impunctate; at margins except basally with fine, irregular punctures.

Elytra: Blackish ferrugineous to dark ferrugineous, with distinct, quite uniform, pale ferrugineous markings (Fig. 458). Rather shiny, although finely microsculptured; reticulation double. Large meshes slightly more strongly developed in comparison with small meshes. Large meshes, when discernible, contain 2–6 small meshes. Laterally and posteriorly mesh-size categories become reduced and difficult to distinguish; still distinctly reticulated. Fine, somewhat irregular punctures form a discal row. Dorsolateral and lateral rows indistinct; indicated by a few fine, scattered punctures. Laterally, with a shallow, finely pubescent, pre-apical furrow.

Ventral aspect: Blackish ferrugineous to ferrugineous, except prothorax, pale ferrugineous. Almost impunctate. Rather shiny, finely microsculptured but reticulation extensively indistinct or totally absent. Abdomen with curved striae, which are reduced at midline. Prosternal process slender; apex extended, pointed. Metacoxal plates in anterior half with about ten, almost transverse and shallow furrows. Apical ventrite with laterobasal, flattened knob (Fig. 120).

Legs: Pro- and mesotarsus rather slender, somewhat extended, with suckers.

Male genitalia: Penis in lateral aspect undulate, posterior to middle quite slender and apex in dorsal aspect obtuse; in lateral aspect penis slightly extended to narrow tip. Furthermore, penis at base provided with a soft flap (Figs 309–310).

Female: Not studied; all four examined specimens are males. According to original description (Pederzani 1983) apical ventrite apically pointed (Fig. 121); most probably female lacks lateral knob on apical ventrite, although absence not mentioned in description.

##### Distribution.

Central African Republic, Cameroon, Equatorial Guinea, Gabon (Fig. 563). Bilardo and Rocchi (2006) report the species from Congo Brazzaville

##### Collecting circumstances.

Some information is available in Bilardo and Rocchi (1990): The site of *Laccophilus concettae* is briefly described as streams in forest with the banks in some places covered by less shaded, semi-submerged vegetation. The bottom is sandy and water is running even in the dry season.

#### Laccophilus biai

Taxon classificationAnimaliaColeopteraDytiscidae

Bilardo & Rocchi, 1990

Figs 122–123 , 311–312 , 459 , 563

Laccophilus biai Bilardo and Rocchi 1990: 161, 176, 177, 187 (original description, faunistics, biology); Leonardi et al. 1995: 192 (list types MSNM); Bilardo and Rocchi 1995: 140 (faunistics, list); Bilardo and Rocchi 1999: 232, 234 (faunistics); Nilsson 2001: 241 (catalogue, faunistics); Bilardo and Rocchi 2002: 173 (list, faunistics); Bilardo and Rocchi 2004: 286 (discussion); Nilsson 2015: 209 (catalogue, faunistics).

##### Type locality.

Gabon: Makokou.

##### Type material studied

(4 exs.). Holotype: male: “Gabon Makoukou 23.8.1987 A. Bilardo / 4.05. x 2.20 / Holotypus / *Laccophilus biai* Bil. & Rocchi det. A. Bilardo” (MSMN; habitus in Fig. 459). – Paratypes: “Paratypus / Gabon Makokou 23.8.87 A. Bilardo / *Laccophilus biai* sp. n. Bil. & Rocchi” (1 ex. MSMN); “Gabon Makoukou 15 km Ouest 23.8. 87 A. Bilardo / *Laccophilus biai* sp. n. Bil. & Rocchi” (2 exx. CSR).

##### Additional material studied

(5 exs.). **Gabon**: “Bissok (Oyem) F. Lara 8.8. 1991 Bilardo leg” (5 exx. MZH).

##### Diagnosis.

*Laccophilus biai* resembles most of *Laccophilus concettae*, *Laccophilus deceptor*, *Laccophilus bilardoi* and *Laccophilus decorosus*. From *Laccophilus deceptor*, *Laccophilus bilardoi* and *Laccophilus decorosus*, *Laccophilus biai* is distinguished by the transverse pale area at base of elytra, which in *Laccophilus biai* is unbroken or almost unbroken, while in the three other the pale basal area is divided into separate, pale spots. *Laccophilus biai* and *Laccophilus concettae* is separated by differences in the shape of penis; *Laccophilus biai* has pointed penis apex (Fig. 312) while in *Laccophilus concettae* penis apex is obtuse (Fig. 310).

##### Description.

Body length 3.9–4.0 mm, width 2.0–2.1 mm. Habitus as in Fig. 459. Elytra extensively dark coloured; at base with transverse paler area. Posterior to transverse pale area with dark irroration.

Head: Pale ferrugineous. Submat, finely microsculptured. Reticulation double; of two kinds but difference between coarse and fine meshes in part vague. Large meshes contain 2–5 fine meshes. At eyes with dense, irregular punctures.

Pronotum: Pale ferrugineous. Frontally, often with indistinct, vague, darkened area. Basally, with medial, somewhat vague dark marking. Submat to rather shiny, finely microsculptured. Reticulation double, of two kinds; large meshes contain 2–5 fine meshes. At margins with fine, somewhat irregular punctures.

Elytra: Extensively with dark irrorations. Basally, with pale ferrugineous, transverse marking. Towards apex, elytra with dark irroration slightly sparser (Fig. 459). Submat, finely microsculptured. Anteriorly, reticulation of two kinds; division of size classes fades away posteriorly. Very fine, irregular punctures form a discal, dorsolateral and lateral row of punctures.

Ventral aspect: Dark ferrugineous to ferrugineous. Prothorax pale ferrugineous. Metacoxal plates with a few, indistinct transverse (almost obliterated) furrows. Rather shiny, although finely reticulate. Abdomen basally with distinct but sparse, curved striae. Impunctate. Prosternal process slender, apically pointed. Apical ventrite with pointed apex and minor knob (Fig. 122).

Legs: Pale ferrugineous to ferrugineous. Pro- and mesotarsi rather long, basally slightly enlarged, with distinct suckers.

Male genitalia: Penis in lateral aspect strongly undulate; posterior to middle penis quite slender and at base provided with a soft flap. Apex of penis in lateral aspect extended to a small, obtuse extension; in dorsal aspect extreme apex bluntly pointed (Figs 311–312).

Female: Pro- and mesotarsus slender. Apical ventrite apically rounded; minor knob absent (Fig. 123).

##### Distribution.

Gabon (Fig. 563).

##### Collecting circumstances.

Original description gives some information of the collecting localities in Gabon (Bilardo and Rocchi 1990). This information, however, relates to the general description of the collecting sites and overlooks specific data on *Laccophilus biai*.

#### Laccophilus deceptor

Taxon classificationAnimaliaColeopteraDytiscidae

Guignot, 1953

Figs 124–125 , 313–315 , 460 , 561

Laccophilus deceptor Guignot 1953d: 1 (original description, faunistics); Guignot 1954: 25 (description, faunistics); Guignot 1959a: 533, 535, 537 (description, faunistics); Nilsson 2001: 242 (catalogue, faunistics); Nilsson 2015: 210 (catalogue, faunistics).

##### Type locality.

Zaire: Parc National Upemba, Mubale.

##### Type material studied

(2 exs.). Holotype: male: “Holotypus / Congo belge P.N.U. Mubale (1.480 m.) 10-13-V-1947 Mis. G.F. de Witte. 352a / Coll. Mus. Congo (ex. coll. I. P. N. C. B.) / *Laccophilus deceptor* Guignot Type male / Guignot det. 1952 *Laccophilus deceptor* Guign. Type male” (MRAC). – Paratype: female: Same data as in holotype, but labelled as “Paratypus” (1 ex. MRAC).

##### Additional material studied

(2 exs.). **Zaire**: “Paratypus / Mbuye Bala (1750 m.) / F. Guignot det., 1953 *Laccophilus deceptor* sp. n. (1 ex. MRAC; habitus in Fig. 460); “PNU Katongo affl. Mubale (1750 m) 12.4. 1948 de Witte, 1522a / Paratype / *Laccophilus deceptor* Guign. det. Guignot 1953” (1 ex. IRSNB). [Comment: not type material although labelled as paratypes; label data do not fit the original description.]

##### Diagnosis.

Among resembling species most similar and probably closest related to *Laccophilus bilardoi* but body of *Laccophilus deceptor* is distinctly larger. Male genitalia resemble also of genitalia in *Laccophilus bilardoi*. *Laccophilus deceptor* can be distinguished by specific differences in shape of penis (see also, diagnosis of *Laccophilus bilardoi* on p. 170).

##### Description.

Body length 4.5 mm, width 2.40–2.5 mm. Dorsal, aspect of body as in Fig. 460. Pale markings quite distinct.

Head: Pale ferrugineous. Slightly mat, finely and quite densely microsculptured. Reticulation double but difference between fine and coarser meshes rather small. Coarser meshes contain 2–6 finer meshes. At eyes with fine punctures.

Pronotum: Pale ferrugineous, frontally and at base medially with a dark ferrugineous to ferrugineous area. Submat, reticulation double. Coarser meshes fine but clearly discernible; contain 2-6 finer meshes. Frontally and laterally with irregular, fine punctures.

Elytra: In frontal part with an irregular, transverse row of distinct, pale ferrugineous spots. Posterior to pale spots elytra, with somewhat irregular irrorations (Fig. 460). Submat, rather densely microsculptured. Reticulation double, posteriorly, difference between kinds of reticulation gradually disappears. Scattered fine punctures form on each elytron three somewhat indistinct, longitudinal areas of punctures. Lateral, pre-apical furrow distinctly pubescent.

Ventral aspect: Pale ferrugineous to ferrugineous; exhibits no distinct colour pattern. Rather shiny to slightly mat, finely and extensively microsculptured. Almost impunctate. Basal ventrites with fine but distinct, somewhat curved striae. Metacoxal plates with a few very shallow transverse furrows. Prosternal process slender, apex pointed. Apical ventrite asymmetric; with lateral, on one side located, small but distinct knob (Fig. 124).

Legs: Pro- and mesotarsus quite long, basally slightly enlarged; with distinct suckers.

Male genitalia: Penis in lateral aspect with external outline strongly undulate but from middle forwards straightened; apex extended forwards to a distinct tip. Penis at base with a soft flap (Figs 313–315).

Female: Externally as male. Apical ventrite almost symmetric (striae with different location), lacks lateral knob (Fig. 125). Pro- and mesotarsus slender, basally not distinctly enlarged.

##### Distribution.

Zaire (Fig. 561).

##### Collecting circumstances.

Not documented, unknown.

#### Laccophilus bilardoi

Taxon classificationAnimaliaColeopteraDytiscidae

Pederzani & Rocchi, 1982

Figs 126–127 , 316 , 461 , 561

Laccophilus bilardoi Pederzani and Rocchi 1982: 71, 77 (original description, faunistics); Bartolozzi et al. 1984: 75 (list of types in MZUF); Nilsson 2001: 241 (catalogue, faunistics); Bilardo and Rocchi 2004: 286 (discussion); Bilardo and Rocchi 2006: 129 (faunistics); Bilardo and Rocchi 2008: 210, 230, 231, 234 (faunistics, biology); Bilardo and Rocchi 2013: 141 (faunistics, biology); Nilsson 2015: 209 (catalogue, faunistics).

##### Type locality.

Congo (Brazzaville): Impfondo.

##### Type material studied

(1 ex.). Holotype: male: “Congo Rep. Pop., Reg. Nord-Est, Impfondo, à la lumiére 7.6. 80 Onore / *Laccophilus bilardoi* sp. n. Holotype / *Laccophilus bilardoi* Pederzani & Rocchi, Rocchi S. det. 1982 / “La Specola” Firenze 6869” (MZUF).

##### Additional material studied

(7 exs.). **Gabon**: “Plateaux Batéké Village Léwou, 30.8. 2008 Bilardo / *Laccophilus bilardoi* Ped. & Roc. det. Rocchi 2009” (3 exs. CSR; habitus in Fig. 461); “Parc Nat. Plateaux Batéké CP 40 17.6. 2012 Bilardo / Bosquet en savane alt. 575 m S02.19,256, E014.10,547 / *Laccophilus bilardoi* Ped. & Roc. det. Rocchi 2013” (4 exs. CSR).

##### Diagnosis.

Externally *Laccophilus bilardoi* resembles much of *Laccophilus deceptor* but body-size distinctly smaller in *Laccophilus bilardoi* (length of *Laccophilus deceptor* is about 4.5 mm). Pale colour patches and irrorations are also distinctly more extensive in *Laccophilus deceptor*. Both species with quite similarly shaped penis. Useful features for identification are differences in details of penis-apex-outline when penis viewed laterally. See also illustrations of *Laccophilus decorosus* (Fig. 317).

##### Description.

Body length 3.6–3.8 mm, width 2.0–2.1 mm. Dorsal colour extensively dark with limited paler markings on pronotum and elytra (Fig. 461).

Head: Pale ferrugineous, posteriorly slightly darker; ferrugineous to pale brownish; change in colour is gradual. Submat, finely microsculptred; reticulation double, although size categories in part difficult to distinguish. Frontally double reticulation disappears. Almost impunctate, except at eyes with fine, scattered punctures.

Pronotum: Dark ferrugineous to dark brown. Frontally, posterior to eyes, pronotum with a small pale brown to yellowish marking (Fig. 461). Rather shiny, finely microsculptured; reticulation double but size-categories difficult to distinguish. Large meshes include 2–5 small meshes. Almost impunctate; at frontal margin with scattered fine punctures.

Elytra: Dark ferrugineous to dark brown, with distinctly delimited pale spots arranged transversely over the elytra at base (Fig. 461). Colour pattern exhibits slight variation but same ground plan discernible. Rather shiny, although finely microsculptured. Reticulation indistinctly double. Large meshes contain 2–5 small meshes. Double reticulation extensively (laterally and posteriorly) indistinct or absent. Discal, dorsolateral and lateral rows of punctures indistinct; consist of sparse and scattered, very fine (hardly discernible) punctures.

Ventral aspect: Pale ferrugineous to ferrugineous, in part slightly darker. Rather shiny, with fine, partially somewhat indistinct microsculpture. Metacoxal plates in frontal half with some fine and indistinct, almost transversely located shallow furrows. Basal ventrites with fine, curved striae. Almost impunctate. Prosternal process slender, apex pointed. Apical ventrite provided with an asymmetric small knob (Fig. 126). Apex of apical ventrite pointed.

Legs: Pale ferruginous, metatarsus a little darker, ferrugineous to dark ferrugineous. Pro-and mesotarsus with suckers.

Male genitalia: Penis in lateral aspect with external outline quite strongly undulate; distinctly curved but from middle of penis to apex, outline almost straight. Extreme apex formed as a broad hook; penis basally provided with a soft flap (Fig. 316).

Female: Apex of apical ventrite rounded; ventrite lacks asymmetric knob (Fig. 127). Body submat to mat, dorsal aspect of body strongly microsculptured.

##### Distribution.

Gabon, Congo (Brazzaville) (Fig. 561).

##### Collecting circumstances.

Almost unknown. Regarding collecting localities, see Bilardo and Rocchi (2008, 2013): The species has been collected in both savannah and forest sites; no detailed information related directly *Laccophilus bilardoi* is given. Label-data of holotype indicate that it was collected at light.

#### Laccophilus decorosus sp. n.

Taxon classificationAnimaliaColeopteraDytiscidae

http://zoobank.org/E87F69A8-FE8C-4B39-8902-CAE23DF78529

Figs 128–129 , 317 , 462 , 463 , 561

##### Type locality.

Uganda: Lake Nabugabo.

##### Type material

(3 exs.). Holotype, male: “Stn. No. B31(HR) / Uganda Lake Nabugabo vii-viii. 1962 / Cambridge Univ. Biol. Survey 1962. B.M. 1963-727” (BMNH; habitus in Fig. 462). – Paratypes: Same data as holotype (1 ex. BMNH, 1 ex. MZH; habitus in Fig. 463).

##### Diagnosis.

Closest relatives to *Laccophilus decorosus* seem to include *Laccophilus concettae*, *Laccophilus biai*, *Laccophilus bilardoi* and *Laccophilus deceptor*. These species are characterized especially by their complicated aedeagus-structure, ground plan of which is still similar in all the species. Differences in shape of male genitalia and elytral colour pattern distinguish *Laccophilus decorosus* from the other species.

##### Description.

Body length 4.2 mm, width 2.2–2.3 mm. Dorsal, colour pattern of body distinct but on elytra it is quite variable; transverse row of pale spots may be reduced to simple, humeral spots (Figs 462–463).

Head: Pale ferrugineous, posteriorly head becomes gradually darker; at pronotum dark ferrugineous to brownish. Slightly mat, finely microsculptured; reticulation distinctly double (size-categories clearly separated). Large meshes contain 2–6 small meshes. Almost impunctate. At eyes with fine, irregular punctures. Puncture-areas extend towards head-middle but leave still a wide, impunctate gap between them.

Pronotum: Dark ferruginous to dark brown, with small, pale ferrugineous spots on pronotal disc posterior to eyes. Rather shiny to slightly mat, finely microsculptured; reticulation clearly double. Large meshes contain 2–6 small meshes. Impunctate, except at margins, where fine, scattered punctures are discernible.

Elytra: Blackish to dark ferrugineous, with well delimited but variable pale ferrugineous spots. Basally with a transverse row of irregularly shaped, pale spots. Spots can be reduced and restricted to a humeral spot. Laterally, in middle and apically with a small, pale area. Extensively on elytra, dark, quite rude irrorations may be discerned (Figs 462–463). Discal row of punctures is formed by slightly irregular, fine punctures. Laterally, to discal puncture-row with scattered, fine punctures, not forming distinct rows. Pre-apical, lateral row of punctures located in a shallow, pubescent furrow posteriorly along edge of elytron. Slightly mat, finely microsculptured, reticulation clearly double. Large meshes may contain between 2–6 small meshes. Laterally and posteriorly size-categories of reticulation becomes diffuse and difficult to distinguish.

Ventral aspect: Dark ferrugineous to ferrugineous, apically blackish ferrugineous. Colour change gradual, no distinct colour pattern formed. Rather shiny, although with very fine microsculpture, except on abdomen which is mainly shiny with microsculpture extensively absent. Ventrites, with sparse, fine and slightly curved striae. Almost impunctate; apical ventrite with a few, scattered, fine punctures. Apical ventrite asymmetric; provided with a comparatively low, but broad, sharp, process on one side (Fig. 128). Metacoxal plates with shallow, rather indistinct, transverse furrows, which in posterior half are strongly reduced and almost absent.

Legs: Pale ferrugineous to ferrugineous. Pro- and mesotarsus slightly enlarged, provided with distinct suckers.

Male genitalia: Penis in lateral aspect with external outline undulate; posterior to middle penis slender. Extreme apex of penis with a quite broad extension and a pre-apical enlargement (Fig. 317).

Female: Pro- and mesotarsus slender. Apical ventrite lacks lateral, process (Fig. 129). Body of female slightly duller because of microsculpture stronger than in male.

##### Etymology.

The species name *decorosus* is a Latin adjective meaning “very fair”. It relates to the nice and decorative appearance of the new species.

##### Distribution.

Uganda (Fig. 561).

##### Collecting circumstances.

Unknown.

#### Laccophilus tschoffeni

Taxon classificationAnimaliaColeopteraDytiscidae

Régimbart, 1892

**Figures 130–138.**
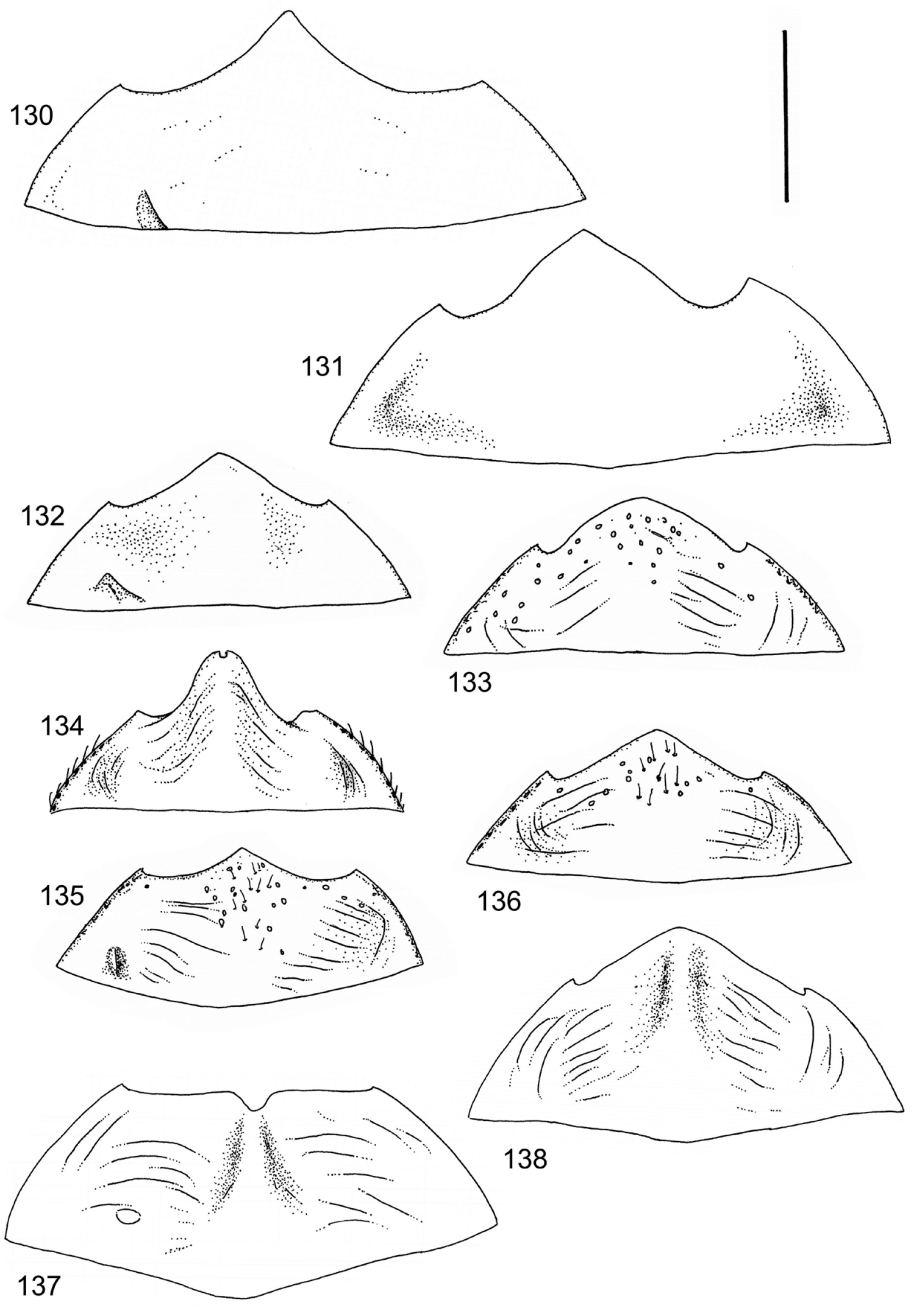
Apical ventrites **130** *Laccophilus tschoffeni* male, and **131** female **132** *Laccophilus persimilis* male, and **133** female **134** *Laccophilus caiaricus* female **135** *Laccophilus poecilus* male, and **136** female **137** *Laccophilus mutatus* male, and **138** female. Scale bar 0.5 mm.

Figs 130–131 , 318–319 , 464 , 562

Laccophilus tschoffeni Régimbart; Severin 1892: 472 (type material in IRSNB; no description); Régimbart 1895: 139 (original description, faunistics); Zimmermann 1920a: 27 (catalogue, faunistics); Guignot 1943: 98 (faunistics); Guignot 1952e: 168 (discussion, correction of earlier misidentification); Guignot 1953d: 1 (discussion); Guignot 1954: 26 (discussion); Guignot 1955b: 1096 (discussion); Guignot 1956b: 219 (discussion, faunistics); Guignot 1959a: 533, 535, 537, 564 (description, discussion, faunistics); Nilsson 2001: 252 (catalogue, faunistics); Nilsson 2015: 218 (catalogue, faunistics).

##### Type locality.

Zaire: Boma.

##### Type material studied

(7 exs.). Lectotype (by present designation): male: “Banana Boma M. Tschoffen 91 Det. Régimb. 91 / 11176 / Régimbart det, 1891 *Laccophilus tschoffeni* Rég.” (IRSNB). – Paralectotypes: Same data as lectotype (3 exs. IRSNB; habitus in Fig. 464); “Boma M. Tschoffen / *Laccophilus tschoffeni* Rég.” (1 ex. IRSNB); “Matadi M. Tschoffen / *Laccophilus tschoffeni* Rég. Typus / Régimbart 1895 *Laccophilus tschoffeni* Rég. / Type” (1 ex. IRSNB); “Severin Banana Africa / Banana Boma M. Tshoffen 91 Dét. Régimb. / *Laccophilus tschoffeni* Type Régimb. / Type” (1 ex. RMNH).

##### Additional material studied

(2 exs.). **Zaire**:“Congo Boma M. Tshoffen” (2 exs. SAMC; possibly type material).

##### Diagnosis.

*Laccophilus tschoffeni* is particularly distinguished by the volumnious male genitalia, and therefore it is not to be confused with any other African *Laccophilus* species; penis in lateral aspect curved, apically provided with membranous, rather narrow flaps.

##### Description.

Body length 4.3–4.5 mm, width 2.40 mm. Habitus and dorsal colour pattern of body as in Fig. 464.

Head: Pale ferrugineous. Submat, rather finely but distinctly microsculptured. Reticulation double; large meshes only slightly more strongly developed than fine meshes. In part, large meshes indistinct. When discernible, large meshes contain 4-8 small meshes. Almost impunctate, except at eyes, with sparse, fine punctures. Punctate area extends towards middle of head.

Pronotum: Pale ferrugineous, basally with vague darker marking. Submat, finely microsculptured. Extensively with double reticulation but difference between mesh-categories in part indistinct. At margins, pronotum with scattered fine punctures.

Elytra: Dark ferrugineous to ferrugineous, with irrorations, except on pale ferrugineous, basal transverse area (Fig. 464). Irrorations are sparser on a somewhat vague, transverse area posterior to middle. Finely reticulated; reticulation extensively double. Punctures fine and scattered; clear rows cannot be distinguished. Pre-apical, lateral furrow punctate and finely pubescent.

Ventral aspect: Ferrugineous to pale ferrugineous. Prosternal process slender, apically pointed. Almost impunctate; irregularly distributed, hardly visible, punctures are discernible. Rather shiny, very finely microsculptured. Basal ventrites finely striated. Apical ventrite with a minute ridge, as in Fig. 130.

Legs: Pale ferrugineous to ferrugineous. Protarsus rather slender, claws slightly curved and somewhat prolonged. Tarsi provided with suckers.

Male genitalia: Penis large, voluminous; in lateral aspect medially bent, curved, apically provided with membranous, rather narrow flaps (Figs 318–319).

Female: Apical ventrite symmetric, lacks ridge (Fig. 131).

##### Distribution.

Zaire (Fig. 562).

##### Collecting circumstances.

Unknown.

#### Laccophilus persimilis

Taxon classificationAnimaliaColeopteraDytiscidae

Régimbart, 1895

Figs 132–133 , 320 , 465 , 563

Laccophilus persimilis Régimbart 1895: 144 (original description, faunistics); Zimmermann 1920a: 24 (catalogue, faunistics); Guignot 1959a: 533, 540 (description, faunistics); Bilardo 1982a: 447 (description, faunistics, given as *Laccophylus*); Franciscolo and Sanfilippo 1990: 146 (description, faunistics); Nilsson et al. 1995: 505 (faunistics); Nilsson 2001: 248 (catalogue, faunistics); Bilardo and Rocchi 2002: 155, 160, 174 (faunistics, list); Nilsson 2015: 216 (catalogue, faunistics).

##### Type locality.

Gabon: Cap Lopez.

##### Type material studied

(4 exs.). Lectotype (by present designation): male: “Cap Lopez / Museum Paris coll. Maurice Régimbart 1908” (MNHN; lectotype is the specimen to right on a label with two specimens, mounted side by side). – Paralectotypes: On same label as lectotype with same data (1 ex. MNHN); “Chutes de Samba, Riv. N’Gounie 92 Mocquerys / *persimilis* Rég. type / Régimbart det. 1895 *Laccophilus persimilis* Rég. / Type” (1 ex. IRSNB); “Museum Paris coll. Maurice Régimbart 1908 / Chutes de Samba, Riv. N’Gounié Gabon (Mocquerys) / *persimilis* Rég.”(1 ex. MNHN).

##### Additional material studied

(42 exs.): **Gambia**: “Bathurst Jan. 1968 Palm / *Laccophilus persimilis* Régb. det. S. Persson det.” (1 ex. NHRS). – **Guinea Bissau**: “Oio, 2 km E Binar, temp. pool 21.7. 1992 Persson leg.” (3 exs. MZLU); “Oio 10 km W Binar, flooded area 29.8. 1992” (3 exs. MZLU) “Cacheu, Bula, temporary pools 25.7. 1992 Persson leg.” (10 exs. MZLU); same but “16.7.” (5 exs. MZLU); same but “5 km W Bula 18.7. 1992” (6 exs. MZLU); “Gabu, 10 km E Gabu, ponds 3.4. 1993 Persson leg.” (8 exs. MZLU). – **Sierra Leone**: “Makeni 12°03'W, 8°53'N, 27.11. 1993 light trap 18-21 / Cederholm, Danielsson & Hall leg.” (1 ex. MZLU; habitus in Fig. 465); “Njala Riv. Tiai 8. 1944 Walton” (1 ex. BMNH). – **Nigeria**: “Lagos Colony Iseri 29-30.3. 1949 B. Malkin / Meander pool, shallow water” (1 ex. BMNH). – **Gabon**: “Lagune Iguelà Gen. 97 Bilardo / *Laccophilus persimilis* Régb. det. Rocchi 1999” (3 exs. CSR).

##### Diagnosis.

*Laccophilus persimilis* is particularly characterized by peculiar elytral colour pattern and uniquely shaped penis apex (broad and triangular). This character-combination distinguishes the species from all other African *Laccophilus* species.

##### Description.

Body length 3.8–4.3 mm, width 2.0–2.3 mm. Dorsal aspect with distinct colour pattern (Fig. 465); slight variation observed in appearance of pale, transverse, subbasal marking of elytra.

Head: Pale ferrugineous, posteriorly at eyes with vague, somewhat darker area. Reticulation double; in part weakly developed. Large meshes (when discernible) contain generally 3–6 small meshes. Almost impunctate; at eyes with a few, rather indistinct, slightly coarser punctures.

Pronotum: Pale ferrugineous, frontally at head (between eyes) and mediobasally with dark ferrugineous, slightly vague marking. Slightly mat, rather distinctly microsculptured. Reticulation double. Large meshes contain generally 2–6 small meshes. Small meshes sometimes, weakly developed and rather indistinct. Almost impunctate.

Elytra: Dark ferrugineous, with distinct pale ferrugineous, subbasal and pre-apical markings (Fig. 465). Subbasal, pale marking sometimes formed by a number of connected, pale spots located transversely over elytra. Submat, with double reticulation. Larger meshes at base and frontally at suture distinct and their shape mainly longitudinal. Laterally and posteriorly large meshes indistinct or absent. A longitudinal mesh may contain 4–10 small meshes. Discal and lateral row of punctures discernible, but very weakly developed and rather indistinct. Lateral, pre-apical furrow fine, finely pubescent.

Ventral aspect: Pale ferrugineous. Abdomen laterally and apically with vague darker (dark ferrugineous to ferrugineous) area. Rather shiny, very finely and indistinctly microsculptured. Basal ventrites with fine, in part indistinct, curved striae. Almost impunctate; scattered fine punctures may be discerned. Prosternal process slender, apex somewhat extended, pointed. Metacoxal plates in anterior half with quite distinct, transversely located, shallow furrows. Posteriorly the furrows are almost absent. Apical ventrite asymmetric; on one side with a sharp knob (Fig. 132).

Legs: Pro- and mesotarsus slightly enlarged; provided with suckers.

Male genitalia: Penis in lateral aspect strongly modified; from base to apex enlarged, broadly triangular with a sharp knob in middle of external outline (Fig. 320).

Female: Apical ventrite lacks lateral knob, almost symmetric (Fig. 133). Pro- and mesotarsus rather slender.

##### Distribution.

Gambia, Guinea Bissau, Sierra Leone, Nigeria, Gabon (Fig. 563). Bilardo (1982) adds Cameroon to range state.

##### Collecting circumstances.

According to label data the species has been collected in flooded area, in temporary ponds and at light.

#### Laccophilus caiaricus

Taxon classificationAnimaliaColeopteraDytiscidae

Guignot, 1956

Figs 134 , 466 , 562

Laccophilus caiaricus Guignot 1956f: 792 (original description, faunistics); Nilsson 2001: 241 (catalogue, faunistics); Nilsson 2015: 210 (catalogue, faunistics).

##### Type locality.

Senegal: Cayar.

##### Type material studied

(2 exx.). Holotype: female: “IFAN 1954 Kayar Senegal / IFAN 1954 No 3254 Abonnene / Type / F. Guignot det., 1954 *Laccophilus caiaricus* sp. n. Type female” (MNHN). – Paratype: Almost same data but labelled: “Paratype” (1 ex. MNHN; habitus in Fig. 466).

##### Diagnosis.

Most probably to be regarded a distinct species although only female is known. It is distinguished by its peculiar colour pattern, different from all other recognized *Laccophilus* species in Africa. From species placed in this species group *Laccophilus caiaricus* is distinguished by being the smallest species with maximum length of body 3.5 mm (minimum length of body in other species included is 3.6 mm). [Comment: location of *Laccophilus caiaricus* in this species group is uncertain.]

##### Description.

Body length 3.5 mm, width 1.9–2.0 mm. Dorsal colour pattern of body rather distinct, slightly variable (Fig. 466).

Head: Pale ferrugineous; posteriorly at eyes with vague, dark ferrugineous areas. At eyes with fine, scattered punctures; punctures extend towards middle of head-disc (puncture-areas still clearly separated medially). Submat, distinctly microsculptured. Reticulation double. Coarse meshes fine, in part hardly discernible. Coarse meshes may contain 2–6 finer meshes.

Pronotum: Pale ferrugineous to ferrugineous. Frontally in middle with distinct darkened areas. Basally in middle with vague darker area. Frontally with fine, irregular, in part indistinct punctures. Submat, finely microsculptured. Reticulation double. Coarse meshes contain 2–6 fine meshes. Fine meshes in part indistinct and hardly discernible.

Elytra: Ferrugineous to dark ferrugineous, with slightly variable, pale ferrugineous markings; in part dark colour forms vague undulations (Fig. 466). Almost impunctate; very fine, sparse punctures may be discerned discally, dorsolaterally and laterally. Submat, very finely microsculptured. Reticulation indistinctly double; coarse meshes weakly developed, in part obliterated.

Ventral aspect: Pale ferrugineous. Almost impunctate. Rather shiny although with very fine microsculpture. Metacoxal plate with about 10 transverse, shallow furrows. Abdomen with fine curved striae. Female apical ventrite (Fig. 134). Prosternal process slender, apex extended and pointed.

Legs: Pro- and mesotarsus slender, somewhat extended.

Male: Unknown.

##### Distribution.

Senegal (Fig. 562).

##### Collecting circumstances.

Unknown.

### Species group 12 (*Laccophilus poecilus* group)

**Diagnosis.** Medium sized species with length 3.6-4.0 mm and width 2.0–2.1 mm.

Body, shape oval and dorsoventrally flattened. Elytra with vague, quite robust, ferrugineous irrorations and pale areas formed as vague patches (Fig. 467). Body microsculpture double; large meshes only slightly more strongly developed than small meshes. In part large meshes almost absent and only fragments of meshes discernible.

Prosternal process slender, moderately extended posteriorly, apex pointed. Apical ventrite with posterior end distinctly excavated and medial part posteriorly extended; male provided with an asymmetrical knob on one side of ventrite (Fig. 135). Metacoxal plates lack stridulatory apparatus. Metacoxal process ends abruptly; lacks posterior extension (Fig. 6).

Paramere rather simple; broad at base and narrows strongly towards apex (Fig. 321). Penis quite robust; towards apex enlarged and ends abruptly; externally at base with a distinct incision (Fig. 321).

**Species composition and distribution.** One species is recognized from Africa, north of Sahara.

#### Laccophilus poecilus

Taxon classificationAnimaliaColeopteraDytiscidae

Klug, 1834

Figs 135–136 , 321–322 , 467 , 529

Laccophilus poecilus Klug 1834: fig. 8 (original description); Schaum 1864: 106 (faunistics); Sharp 1882: 287, 821 (description, faunistics); v. d. Branden 1885: 23 (catalogue, faunistics); Régimbart 1895: 133 (description, faunistics); Zimmermann 1920a: 24 (catalogue, faunistics); Zimmermann 1930: 21, 23 (description, faunistics); Guignot 1959a: 532, 533, 534, 538 (description, biology, faunistics, discussion); Guignot 1961a: 932 (biology); Alfieri 1976: 31 (faunistics, discussion); Nilsson 2001: 249 (catalogue, faunistics); Nilsson 2003: 77 (faunistics, list); Angus 2003: 16 (synonym *Laccophilus ponticus* Sharp); Bennas and Sàinz-Cantero 2006: 60, 62 (faunistics, list); Nilsson 2015: 216 (catalogue, faunistics).
Laccophilus variegatus (Germar & Kaulfuss, 1816), Zimmermann 1920a: 27 (catalogue, faunistics); Guignot 1959a: 533, 538 (praeoccupied, Geoffroy 1785); Angelini 1982: 82 (faunistics); Nilsson 2015: 216 (catalogue, faunistics, synonymy, *Laccophilus poecilus* Klug).
Laccophilus ponticus Sharp, 1882, Nilsson and Holmen 1995: 149 (description, faunistics, biology); Angus 2003:16 (synonymy, lectotype designation of *Laccophilus poecilus* Klug); Nilsson 2015: 216 (catalogue, faunistics, synonym *Laccophilus poecilus* Klug).

##### Comments on synonymy.

Synonymy follows earlier studies. List of references is not complete and includes only studies with an African dimension.

##### Type locality.

Egypt.

##### Type material studied

(1 ex.): Lectotype: female: “9987 / P(not readable) / Aegypten Ehrenberg Nr. 9987/*Laccophilus poecilus* / *Laccophilus poecilus* Klug, Brancucci Klug det. M. Brancucci 92 / Lectotype des., *Laccophilus poecilus* Klug. R.B. Angus det. 2003 (1 ex. ZMHB).

##### Additional material studied

(15 exs.). **Algeria**: “Ouargla / Coll. Régimbart 1908 / *Laccophilus poeclius* Kl.” (1 ex. MNHN). – (non-African): **Greece**: “Corfu, U. & J. Sahlb.”(11 exs. MZH; habitus in Fig. 467). – **Croatia**: “Bokanjac Zadar D. Nowak 9-1899” (1 ex. MZH). – **Croatia-Bosnia-Herzegovina**: “Narenta Gabela U. Sahlb.” (1 ex. MZH).

##### Specimen with unclear labelling.

“Merw Ahnger” (1 ex. MZH).

##### Diagnosis.

*Laccophilus poecilus* is characterized by peculiar elytral colour pattern (resembles elytral colour pattern in species group 11 (*deceptor* group)) in combination with broad, truncate penis apex (lateral aspect). Penis, external curvature, with deep incision anterior to base of penis – only African species, exhibiting this feature.

##### Description.

Body length 3.6–4.0 mm, width 2.0–2.1 mm. Dorsal, colour pattern of body generally distinct, slightly variable but with same ground-plan (Fig. 467).

Head: Pale ferrugineous, posteriorly towards pronotum narrowly darker; ferrugineous to dark ferrugineous. Submat, finely microsculptured. Reticulation double; large meshes only slightly more strongly developed than fine meshes. Large meshes may contain 2–4 smaller meshes. Impunctate, except at eyes, with scattered, irregular punctures.

Pronotum: Pale ferrugineous. Frontally at area between eyes with vague, ferrugineous to dark ferrugineous marking. At base in middle, with a rather distinct, bilobed, blackish to dark ferrugineous spot. Submat, rather finely microsculptured. Reticulation double. Large meshes somewhat more strongly developed than small meshes. Large meshes contain 2–4 small meshes. Almost impunctate, except at margins. At margins except basally in middle with fine, scattered punctures.

Elytra: Dark to blackish ferrugineous with distinct, somewhat variable, pale ferrugineous markings (Fig. 467). Submat to rather shiny, finely microsculptured. Reticulation double. Large meshes only slightly more strongly developed than small meshes. Large meshes, when discernible, contain 2–5 small meshes. Laterally, large meshes in part reduced. Almost impunctate. Discal row of punctures consists of fine, irregular punctures. Outside discal row with scattered, irregularly distributed fine punctures, not forming distinct rows. Pre-apical, lateral row of punctures form a shallow furrow with some hairs.

Ventral aspect: Pale ferrugineous to ferrugineous. No distinct colour pattern but apical half of abdomen generally somewhat darker. Submat, finely microsculptured. Abdomen extensively with microsculpture absent or very fine. Almost impunctate; apical ventrite with sharp knob on one side and some scattered punctures (Fig. 135). Ventrites with fine, slightly curved striae. Metacoxal plates with some 10 almost transversely located furrows, which posteriorly, gradually become indistinct. Prosternal process slender, apex moderately extended and pointed.

Legs: Pro- and mesotarsus somewhat enlarged and extended, provided with distinct suckers.

Male genitalia: Penis in anterior portion enlarged gradually to broad, truncate apex (Fig. 321). In lateral aspect with external outline of penis at base provided with a distinct, deep incision illustrated in Fig. 322.

Female: Pro- and mesotarsus slender. Apical ventrite lacks asymmetric knob on one side (Fig. 136).

**Distribution** in Africa: Egypt, Algeria (Fig. 529). Nilsson (2003) gives also Morocco.

##### Collecting circumstances.

Guignot (1959a) reports the species in standing water of desert areas. Nilsson and Holmen (1995) report that the species in Scandinavia is mainly known from brackish water, where it occurs in sheltered bays on silty bottoms with dense vegetation (*Phragmites* and *Scirpus*). Also known from inland fresh water, e.g. from peaty water bodies.

### Species group 13 (*Laccophilus lineatus* group)

**Diagnosis.** Small to large sized species, length of body 3.0–5.1 mm, width 1.6–2.8 mm.

Body shape oblong to oval; dorsoventrally flattened. All recognized species exhibit distinct colour pattern, the ground-plan of which is dark coloured, longitudinal markings on each elytron. In one species the markings are strongly undulate or totally reduced (Figs 478–480). Many species have markings which partly may be slightly undulate and also connected to neighbour-markings (Figs 482, 486). In some cases dark markings are merged to extensive dark areas and their longitudinal origin may be difficult to discern (Figs 483, 489). A more or less distinct, transverse pale marking at base of elytra is present in some species (Figs 477, 490). Body microsculpture is double, often however so that large meshes are strongly reduced and only fragmentarily discernible.

Prosternal process is slender, posteriorly extended and apically pointed. Apical ventrite modified; posterior part on both side of midline excavated and medial part forms a backwards extending process. Apical ventrite of male provided with asymmetrical knob on one side of ventrite (Figs 141, 152). Metacoxal plates lack stridulatory apparatus. Metacoxal process ends abruptly; lacks posterior extension (Fig. 6).

Paramere rather simple, apically enlarged but not strongly modified (Fig. 340). Penis apex in many species strongly modified; asymmetric and apically often hooked. Sometimes apex of penis constricted posterior to hook and in a few species constriction reduced to a simple incision. Penis sometimes robust, voluminous; almost straight to strongly bent. In determination, check of male genitalia is obligatory.

**Species composition and distribution.** 20 species are recognized in this species group. All of them are distributed in mainland Africa, South of Sahara and Madagascar.

#### Key to species (males)

**Table:** 

1	Tip of penis straight or slightly bent (Figs 327, 350)	**2**
–	Tip of penis strongly bent (up to 90°) (Figs 354, 356)	**19**
2	Penis lacks distinct apex (apically smooth, truncate, pointed, with minor apical extension) (Figs 344, 349)	**3**
–	Penis with distinct asymmetric apex (with hook, truncate lateral enlargement) (Figs 324, 335)	**7**
3	Large species, body length 4.1–4.3 mm; penis, lateral aspect, strongly bent (Fig. 345)	***Laccophilus grammicus*** (p. 208)
–	Smaller species, body length 3.5–4.0 mm; penis, lateral aspect, distinctly straighter	**4**
4	Penis apex broad, clearly twisted (Fig. 349)	**5**
–	Penis apex comparatively slender, slightly curved, not twisted (Fig. 347)	**6**
5	Penis apex with minor but distinct, apical extension (Fig. 350)	***Laccophilus lineatus*** (p. 214)
–	Penis lacks distinct apical extension (Figs 348–349)	***Laccophilus burgeoni*** (p. 212)
6	Penis, lateral aspect, slightly and evenly curved; equally broad (Fig. 347)	***Laccophilus flavoscriptus*** (p. 210)
–	Penis, lateral aspect, external curvature uneven; width unequal (Fig. 353)	***Laccophilus incomptus*** (p. 224)
7	Penis, lateral aspect, two-sided hooked (Fig. 324); elytra with dark, longitudinal markings variable, mixed (Figs 468–469)	***Laccophilus mutatus*** (p. 181)
–	Penis, lateral aspect, one-sided hooked (Fig. 332); colour pattern of elytra different	**8**
8	Elytral longitudinal, dark markings complete, not interrupted; each marking from base to apex strongly undulate (Figs 479–480), or elytral colour pattern reduced, no dark markings (Fig. 478); apex of penis strongly hooked (Fig. 332)	***Laccophilus cyclopis*** (p. 189)
–	Elytral dark markings complete, only in part, slightly undulate (in some species elytral dark markings merged into extensive larger area; apex of penis hooked or shape different	**9**
9	Penis apex distinct, separated by distinct contraction (Fig. 335)	**10**
–	Penis apex contraction merged to narrow incision (Fig. 339)	**16**
10	Base of elytra entirely dark coloured; dark colour interrupted by transverse pale coloured marking which is broken narrowly at suture (Fig. 482)	**11**
–	Base of elytra pale coloured; sometimes provided with dark spots but they do not merge to a complete dark, basal area (Fig. 471)	**14**
11	Dark longitudinal markings of elytra discernible although often partly merged (Fig. 482)	**12**
–	Dark longitudinal markings of elytra merged into uniform, dark area (Fig. 483)	**13**
12	Larger species (body length 3.8–4.3 mm); penis longer and straighter (Fig. 335)	***Laccophilus necopinus*** (p. 199)
–	Small species (body length 3.4–3.8 mm); penis short and curved (Fig. 333)	***Laccophilus adjutor*** (p. 196)
13	Larger species (body length 3.2–3.5 mm); penis delicate, medially distinctly constricted (Figs 337–338)	***Laccophilus conjunctus*** (p. 201)
–	Small species (body length 3.1–3.2 mm); penis more robust, medially not constricted (Fig. 343)	***Laccophilus inconstans*** (Fig. 207)
14	Small species (body length 3.8–4.2 mm); penis posterior to apex on right side with curved outline (Fig. 326)	***Laccophilus quindecimvittatus*** (Fig. 182)
–	Large species (body length 4.5–5.1); penis posterior to apex on right side with straight outline (Fig. 328)	**15**
15	Penis robust; from apex posteriorly, distinctly enlarged (Fig. 328)	***Laccophilus empheres*** (p. 185)
–	Penis more slender; from apex posteriorly moderately enlarged (Fig. 327)	***Laccophilus incrassatus*** (p. 184)
16	Penis apical half almost straight, evenly broad (Fig. 339)	***Laccophilus brownei*** (p. 202)
–	Penis apical half slightly angled, width variable (Fig. 330)	**17**
17	Broad, robust species (length of body 3.9–4.4 mm); penis as in Fig. 326 (Madagascar)	***Laccophilus lateralis*** (p. 187)
–	Elongate, generally smaller species (length of body 3.5–3.9 mm) (Africa, mainland)	**18**
18	Small species (body length 3.5 mm); penis as in Fig. 352	***Laccophilus brancuccii*** (p. 223)
–	Larger species (length of body 3.5–3.9 mm); penis as in Fig. 341–342	***Laccophilus contiro*** (p. 204)
19	Strongly bent tip of penis short (Fig. 354)	***Laccophilus secundus*** (p. 226)
–	Strongly bent tip of penis prolonged (Fig. 356)	***Laccophilus australis*** (p. 230)

#### Laccophilus mutatus

Taxon classificationAnimaliaColeopteraDytiscidae

Omer-Cooper, 1970

Figs 137–138 , 323–325 , 468–469 , 564

Laccophilus mutatus Omer-Cooper 1970: 286, 287 (original description, faunistics); Nilsson and Persson 199a: 79 (status discussion); Nilsson 2001: 247 (catalogue, faunistics); Nilsson 2015: 214 (catalogue, faunistics).

##### Type locality.

Kenya: Athi River.

##### Type material studied

(4 exs.). Holotype: male: “Athi River 19.X. 1957 (CAS, not examined). – Paratypes: “Paratype / *Laccophilus mutatus* O-C. / Kenya Athi River 1530 m X-19-1957 / E.S. Ross & R.E. Leech collectors” (2 exs. AMGS); “Paratype / *Laccophilus mutatus* O-C. / Kenya 17 mi. SW of Nairobi, 1800 m, XI-24-1957 / E.S. Ross & R.E. Leech collectors” (2 exs. AMGS).

##### Additional material studied

(30 exs.). **Kenya**: “Ol Toroto Athi Riv., 5.7. 1970 E.S. Brown leg.” (2 exs. BMNH; habitus in Figs 468–469); “Nairobi 7.10. 1967 / Reichart leg.” (3 exs. USNM); same data, except “16.10. 1967” (2 exs. USNM); same data except “3.11. 1967” (7 exs. USNM, 4 exs. MZH); same data except “Oct. 1968 / Conway leg.” (1 ex. USNM); “Kiserian 26.10.1967 / Reichart leg.” (3 exs. USNM); “Langata Rd. 4.11. 1967 / Reichart leg.”(1 ex. USNM); “10 km N Nyeri Kinganjo 3.11. 1995 Wewalka” (4 exs. CGW, 1 ex. MZH). – **Tanzania**: “Kilimandj. Sjöstedt / Kibonto 1-1200 m / *Laccophilus grammicus* Shp” (1 ex. NHRS).

##### Diagnosis.

*Laccophilus mutatus* externally resembles of *Laccophilus cyclopis*. Peculiar features of penis apex (extreme apex provided with two distinct processes in *Laccophilus mutatus*) distinguish *Laccophilus mutatus* from *Laccophilus cyclopis* as well as does appearance of elytral colour pattern in general. Beware of *Laccophilus cyclopis* specimens with reduced elytral colour pattern.

##### Description.

Body length 4.2–4.7 mm, width 2.4–2.6 mm. Elytra with, in part, reduced dark longitudinal lines; habitus, as in Figs 468–469.

Head: Pale ferrugineous. Slightly mat, finely microsculptured. Reticulation double, coarser meshes contain 2–5 smaller meshes. At eyes with some very fine, irregularly distributed punctures.

Pronotum: Pale ferrugineous, anteriorly in middle with a bilobed, slightly darker spot. Submat, distinctly microsculpturd. Large meshes contain 3–6 fine meshes. Scattered, irregular fine punctures present.

Elytra: Pale ferrugineous, with dark, in part reduced, longitudinal markings (Figs 468–469). Punctation indistinct, posteriorly with sparse and fine punctation. Very finely microsculptured and reticulation almost simple, of one kind.

Ventral aspect: Pale ferrugineous to pale brown, no distinct colour pattern discernible. Rather shiny, although extensively with fine but in part inditinct microsculpture. Metacoxal plates with some shallow, vague and transverse furrows. Abdomen with some inwards curved striae. Almost impunctate. Apex of prosternal process slender and pointed. Apical ventrite asymmetric, with a small, lateral knob (Fig. 137).

Legs: Pro- and mesotarsus rather slender, provided with distinct suckers.

Male genitalia: Penis, apical portion, with two distinct processes (Fig. 324). In lateral aspect penis curved from base to apex; apically, on external outline, provided with a narrow membranous area (Fig. 323).

Female: Apical ventrite apically not incised (Fig. 138). Pro- and mesotarsus slender.

##### Distribution.

Kenya, Tanzania (Fig. 564).

##### Collecting circumstances.

Unknown, not documented.

#### Laccophilus quindecimvittatus

Taxon classificationAnimaliaColeopteraDytiscidae

Régimbart, 1895

**Figures 139–146.**
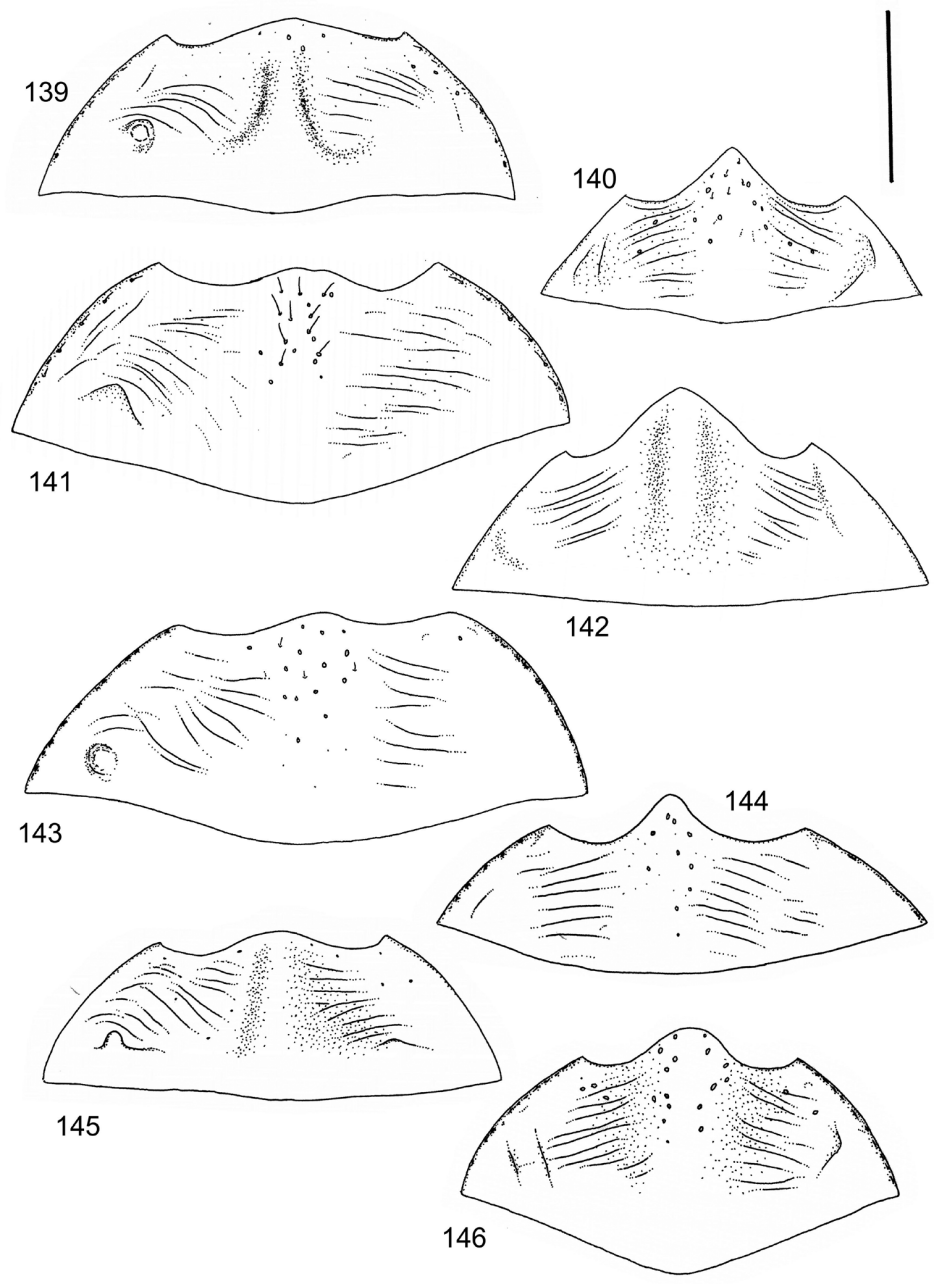
Apical ventrites **139** *Laccophilus quindecimvittatus* male, and **140** female **141** *Laccophilus incrassatus* male, and **142** female **143** *Laccophilus empheres* male, and **144** female **145** *Laccophilus lateralis* male, and **146** female. Scale bar 0.5 mm.

Figs 139–140 , 326 , 470–471 , 564

Laccophilus quindecimvittatus Régimbart 1895: 142 (original description, faunistics); Zimmermann 1920a: 25 (catalogue, faunistics); Omer-Cooper 1931: 758 (description, biology, faunistics); Guignot 1946c: 263 (discussion, description, faunistics); Guignot 1948: 14 (faunistics, discussion); Balfour-Browne 1950: 360 (description, discussion, faunistics); Omer-Cooper 1957: 10 (discussion); Guignot 1959a: 551, 552 (description, faunistics); Nilsson and Persson 1993: 58, 80, 88, 94 (faunistics, discussion, biology); Nilsson 2001: 250 (catalogue, faunistics); Nilsson 2015: 217 (catalogue, faunistics). [Comment: records and information of the species from outside Ethiopia are regarded incorrect.]

##### Type locality.

Ethiopia: Abyssinia.

##### Type material studied

(1 ex.). Holotype: female: “Abyss. Raffray / Type / *15*-*vittatus* Rég. Type unique” (MNHN).

##### Additional material studied

(43 exs.). **Ethiopia**: “Abyssinia Wouramboulchi 9000 ft. 2-7.X. 1926 JOC.” (18 exs. AMGS, 4 exs. MNHN); “Abyssinia Stream W of Zaguala 6000 ft. 27.X. 1926 JOC.” (3 exs. AMGS); “Abyssinia 7900ft Pond Djem Djem forest 10.X. 1926 JOC.” (1 ex. AMGS); “Abyssinia Katterere Riv. Lake Zwai 6000 ft., 5.IX. 1926“ (1 ex. AMGS); “Arsi, Assella Life Stock Farm 28.10. 1988, 2350 m, flooded oat field, Persson leg. / *Laccophilus quindecimvittatus* Régimbart det. Nilsson” (1 ex. TMSA); “Arsi, 10 km S Sagure, Ashebaka Riv., 19.6. 1988 leg. Persson / *Laccophilus quindecimvittatus* Régimbart det. Nilsson” (4 exs. TMSA; habitus in Fig. 470); “Arsi, 10 km S Sagure, two streams, 12.6. 1988 leg. Persson / *Laccophilus quindecimvittatus* Régimbart det. Nilsson” (5 exs. TMSA; habitus in Fig. 471); “Kaffa Jimma 7-8.2. 1974 Silfverberg leg. / *Laccophilus quindecimvittatus* Régb. det. Nilsson” (3 exs. MZH); same data but ”8.2. 1974” (1 ex. MZH); “Kaffa Shebe 11-12.2. 1974 Silfverberg leg.” (1 ex. MZH); “Süd Aethiopien Neumann / Gimivra”(?) (1 ex. ZMHB).

##### Diagnosis.

*Laccophilus quindecimvittatus* belongs to a group of species within this species group, characterized by separate, dark, longitudinal elytral markings (only weekly undulate), which anteriorly can be reduced, forming a transversely located series of dark spots at base of elytra. Additionally, penis of *Laccophilus quindecimvittatus*, has distinct, hooked apex. It seems to be closely related to *Laccophilus incrassatus* and the two species are externally separated by difference in size of body, *Laccophilus incrassatus* being distinctly larger (length 4.6–5.0 mm). Clear differences are also discernible in shape of penis apex, being narrower in *Laccophilus quindecimvittatus*.

##### Description.

Body length 3.8–4.2 mm, width 2.1–2.4 mm. Habitus with dark colour pattern (Figs 470–471).

Head: Pale ferrugineous, close to pronotum narrowly blackish ferrugineous to dark ferrugineous. Submat, finely microsculptured; reticulation double. Large meshes contain 3–5 small meshes. Almost impunctate, except at eyes; with rather fine, somewhat irregular punctures.

Pronotum: Pale ferrugineous. Frontally with broad dark ferrugineous area; basally in middle with quite narrow, somewhat vague, dark ferrugineous marking. Submat, finely microsculptured; reticulation double. Large meshes only slightly more pronounced than small meshes. Large meshes contain 3–5 small meshes. Frontally and laterally with very fine, hardly discernible, scattered punctures.

Elytra: Pale ferrugineous with blackish to dark ferrugineous, longitudinal markings (Figs 470–471). Colour pattern slightly variable; dark longitudinal lines sometimes enlarged so that pale ferrugineous lines narrow. Sometimes dark longitudinal markings strongly reduced frontally so that a pale transverse area is formed (pale transverse area located a little posterior to suture between elytra and pronotum). Submat, finely microsculptured. Reticulation double, but large meshes strongly reduced and almost absent. Minor traces of large meshes may be discerned. Discal, dorsolateral and lateral rows indicated by fine, irregularly located, sparse punctures. Laterally with fine, pre-apical, finely pubescent furrow.

Ventral aspect: Black to blackish ferrugineous. Prothorax pale ferrugineous to ferrugineous. Slightly mat, finely microsculptured. Abdomen with fine, slightly curved striae. Almost impunctate. Prosternal process slender, apex somewhat extended, pointed. Metacoxal plate with about 10 almost transversely located, fine and shallow furrows. Apical ventrite asymmetric; with lateral knob (Fig. 139).

Legs: Pro- and mesotarsus slightly enlarged, extended, provided with suckers.

Male genitalia: Penis in lateral aspect at base curved, anteriorly quite straight; extreme apex angular, rather narrow. External outline provided apically with a rather narrow membranous area (Fig. 326).

Female: Pro- and mesotarsus slender, extended. Apical ventrite symmetric (Fig. 140).

##### Distribution.

Considered as an Ethiopian endemic species (Fig. 564).

##### Collecting circumstances.

According to label-data, collected at high altitudes (6000–9000 ft. = appr. 1850–2700 m). The species occurs in both lotic and lentic water bodies; common in densely vegetated locations; collected from high altitudes (1450–2700 m a.s.l.) (Nilsson and Persson 1993).

#### Laccophilus incrassatus

Taxon classificationAnimaliaColeopteraDytiscidae

Gschwendtner, 1933

Figs 141–142 , 327 , 472–473 , 564

Laccophilus incrassatus Gschwendtner 1933: 85 (original description, faunistics); Guignot 1946c: 263, 264, 265, 312 (description, faunistics, discussion); Legros 1954: 268 (discussion); Guignot 1959a: 544, 549, 550 (description, faunistics); Nilsson 2001: 245 (catalogue, faunistics); Nilsson 2015: 212 (catalogue, faunistics).
Laccophilus virgatus Guignot 1953e: 4 (original description, faunistics); Guignot 1954: 27 (description, faunistics); Guignot 1959a: 544, 546, 550 (description, faunistics); Nilsson 2001: 253 (catalogue, faunistics); Nilsson 2015: 219 (catalogue, faunistics). **New synonym**.

##### Type localities.

*Laccophilus incrassatus*: Zaire: Moero, Kasiki.

*Laccophilus virgatus*: Zaire: Upemba National Park, Riv. Dipwa.

##### Type material studied

(14 exs.). *Laccophilus incrassatus*: Holotype: male: “Holotypus / Musée du Congo Tang. – Moero: Kasiki 20/27-VI-1931 G.F. de Witte / Type Gschw. / R. DÈT. A 2223 / *Laccophilus incrassatus* Gschw. det. Gschwendt.” (MRAC; habitus in Fig. 472). – Paratype: female (considered male in original description): Same data as holotype, but: “Paratype Gschw. / Coll. Gschwendtner / Paratype” (1 ex. OLML).

*Laccophilus virgatus*: Holotype: male: “Holotypus / Congo belge: P.N.U., R. Dipwa (1.900 m) 17-I-1948 Mis. G.F. de Witte, 1239a / Coll. Mus. Congo (ex. coll. I.P.N.C.B.) / *Laccophilus virgatus* sp. n. Type, male symbol / F. Guignot det., 1952 *Laccophilus virgatus Guign*. Type, male symbol” (MRAC). – Paratype: “Congo belge: P.N.U., R. Dipwa (1.900 m) 17-I-1948 Mis. G.F. de Witte, 1242 / Paratype” (5 exs. IRSNB); same but “1293” (1 ex. IRSNB); “Congo Belge P.N.U. Katobwe (Mukana 1810 m) 22-III-1947 Mis. G.F. de Witte: 92a / Paratype” (2 exs. IRSNB); “PNU Mukana 1810 m/12.3. 1947 F. de Witte / Paratype” (1 ex. MNHN); “Congo Belge: PNU Lusinga (Mukana) 28-V-1945 / Paratype / Guignot det., 1953: *Laccophilus virgatus* sp. n.” (1 ex. IRSNB, habitus in Fig. 473); “PNU Lusinga (Galerie) / 22-25.5. 1945 G.F. de Witte / Paratype” (1 ex. MNHN).

##### Additional material studied

(1 ex.). **Zaire**: “PNU Mukana 1810 m, 24.III. 1947 / F. Guignot det., 1953: *Laccophilus virgatus* sp. n.” (1 ex. IRSNB). [Comment: labelled as paratype, but not listed as such in the original description.]

##### Comments on synonymy.

Holotypes of *Laccophilus incrassatus* and *Laccophilus virgatus* have been examined and compared but no characteristics, justifying separation in two species were found. Accordingly, the two species are considered conspecific and *Laccophilus incrassatus*, being older, is the valid name of the species.

##### Diagnosis.

*Laccophilus incrassatus* resembles most of *Laccophilus cyclopis*, *Laccophilus quindecimvittatus* and *Laccophilus empheres*. From *Laccophilus cyclopis*, *Laccophilus incrassatus* is separated by the elytral reticulation, which is clearly double in *Laccophilus cyclopis* while simple or almost simple in *Laccophilus incrassatus* (if larger meshes are discerned they are always rather indistinct and reduced). From *Laccophilus quindecimvittatus*, *Laccophilus incrassatus* is separated by having distinctly larger body (max. 4.2 mm in *Laccophilus quindecimvittatus*). Shape of penis is also characteristic; apex broader in *Laccophilus incrassatus*. From *Laccophilus empheres*, *Laccophilus incrassatus* is distinguished by differences detected in male genitalia; penis distinctly broader in *Laccophilus empheres*.

##### Description.

Body: Length 4.6–5.0 mm, width 2.7–2.8 mm. Pale ferrugineous with distinct blackish ferrugineous to dark ferrugineous colour pattern (Figs 472–473).

Head: Pale ferrugineous. Narrowly at pronotum with a dark ferrugineous, fairly well delimited marking. Submat, rather distinctly microsculptured. Reticulation double; meshes of two kinds and large meshes only slightly coarser than fine meshes. Large meshes discernible in medial part of head; posteriorly and anteriorly with simple reticulation. Large meshes, when discernible, contain two to almost ten small meshes. At eyes with some scattered, fine punctures extending towards centre of head.

Pronotum: Pale ferrugineous, frontally with a quite broad, slightly vague dark ferrugineous marking; mediobasally with a rather narrow, blackish ferrugineous marking. Microsculpture dense and rather distinct; reticulation double. Large meshes only slightly more distinctly developed in comparison with fine meshes. Large meshes contain two to six fine meshes. Almost impunctate. Sparse and irregular, scattered punctures may be discerned.

Elytra: Pale ferrugineous, with distinct dark ferrugineous colour pattern; with longitudinal, blackish to dark ferrugineous markings, which exhibit some variation (Figs 472–473). Submat, rather densely microsculptured; reticulation almost of one kind (large meshes hardly discernible). Discal, dorsolateral and lateral rows of punctures discernible, but fine, irregular and sparse. Lateral, pre-apical furrow finely pubescent.

Ventral aspect: Pale ferrugineous to ferrugineous, metacoxal plates laterally with a narrow blackish area. Metacoxal plates with transveresely located, very shallow furrows. Rather shiny, although, with very fine microsculpture. Abdomen with sparsely located, fine and somewhat curved striae. Almost impunctate. Prosternal process apex extended, slender and pointed. Apical ventrite with a lateral knob (Fig. 141).

Legs: Pro- and mesotarsus quite long and slender, provided with distinct suckers.

Male genitalia: Penis comparatively long, slightly curved and extreme apex forms an angulate, medium broad process. External outline of penis provided with a distinct membranous area (Fig. 327).

Female: Apical ventrite lacks knob (Fig. 142). Pro- and mesotarsus slender.

##### Distribution.

Zaire (Fig. 564). Not recorded from other African countries.

##### Collecting circumstances.

Unknown, not documented.

#### Laccophilus empheres sp. n.

Taxon classificationAnimaliaColeopteraDytiscidae

http://zoobank.org/1D21E352-086B-44C9-9572-B6452B788150

Figs 143–144 , 328–329 , 474 , 475 , 565

##### Type locality.

Kenya: Nairobi.

##### Type material

(10 exs.). Holotype: male: “Nairobi, Kenya 3-XI-67 (STAS) / C.V. Reichart Collector” (USNM; habitus in Fig. 474). – Paratypes: Same data as holotype (1 ex. MZH); “Kenya, Ol Kalou E Nakuru 28.10. 1995 leg. Wewalka (K5)” (6 exx. CGW, 2 exs. MZH; habitus in Fig. 475).

##### Diagnosis.

*Laccophilus empheres* resembles strongly the species mentioned under “Etymology” below. It can be separated by its dark elytral markings, which are always somewhat reduced in humeral region and sometimes in part, also medially. Moreover there are deviating details in the shape of the penis and its apex; penis robust, almost straight and apical, angled process broad.

##### Description.

Body length 4.5–5.1 mm, width 2.5–2.8 mm. Dorsal, colour pattern of body distinct, exhibits minor variation (Figs 474–475).

Head: Pale ferrugineous. Posteriorly at pronotum, head narrowly somewhat darker; ferrugineous to dark ferrugineous. Submat, finely microsculptured; reticulation double. Larger meshes contain 2–5 small meshes. Impunctate, except at eyes; with fine, scattered punctures. Medially punctures extend towards middle of head-disc.

Pronotum: Pale ferrugineous, frontally at area between eyes with a vague, dark ferrugineous marking. At base in middle with a rather narrow, blackish to dark ferrugineous marking. Submat, rather finely microsculptured; reticulation double. Large meshes contain 2–6 small meshes. Impunctate, except at margins; with fine, scattered punctures, which are also lacking medio-basally.

Elytra: Pale ferrugineous, with distinct, blackish to dark ferrugineous, longitudinal stripes, which are often almost complete in central area but basally reduced especially in humeral region (Figs 474–475). Submat, finely microsculptured; reticulation double. Large meshes of elytra are strongly reduced and only fragments of meshes can be discerned. Fine meshes distinct, of same size, and evenly distributed on elytra. Fine, irregular punctures form a vague, discal row of punctures. Similar but more sparsely distributed punctures indicate presence of a vague, dorsolateral and lateral row of punctures. Posteriorly on elytra punctures appear scattered and mixed, and no rows are formed. Pre-apical, lateral furrow of elytra rather shallow, punctate and provided with hairs.

Ventral aspect: Pale ferrugineous to ferrugineous, with no distinct colour pattern. Rather shiny, very finely microsculptured. In part microsculpture somewhat reduced and hardly discernible. Abdomen with fine, slightly curved striae. Almost impunctate; apical ventrite with some irregular punctures and a small knob on one side (Fig. 143). Fine, shallow, transverse furrows on anterior half of metacoxal plates. Metacoxal plates laterally close to epipleura with distinct, longitudinal impression. Prosternal process rather slender, posteriorly somewhat extended, apically pointed.

Legs: Pro- and mesotarsus slightly enlarged, provided with distinct suckers.

Male genitalia: Penis in lateral aspect almost straight, comparatively broad and extreme apex broad and angulate (Figs 328–329).

Female: Apical ventrite lacks asymmetric knob (Fig. 144). Pro- and mesotarsus slender.

##### Etymology.

The Greek word “empheres” is a noun in apposition and refers to something resembling or like. This epithet refers to the fact that the new species, especially externally, resembles strongly of some other *Laccophilus* species located in this species group, as *Laccophilus incrassatus*, *Laccophilus brownei* and *Laccophilus quindecimvittatus*.

##### Distribution.

Kenya (Fig. 560).

##### Collecting circumstances.

Unknown, not documented.

#### Laccophilus lateralis

Taxon classificationAnimaliaColeopteraDytiscidae

Sharp, 1882

Figs 145–146 , 330–331 , 476–477 , 568

Laccophilus lateralis Sharp 1882: 307 (original description, faunistics, discussion); Kolbe 1883: 401 (description, faunistics); v. d. Branden 1885: 21 (catalogue, faunistics); Severin 1892: 472 (discussion); Régimbart 1895: 140 (description, faunistics); Régimbart 1903: 14 (description, faunistics, discussion); Zimmermann 1920a:21 (catalogue, faunistics); Guignot 1942: 15 (description, discussion); Guignot 1946c: 264, 267, 281, 316 (description, faunistics); Guignot 1947: 26 (discussion); Guignot 1948: 13 (discussion); Guignot 1952d: 5, 6 (discussion, description); Omer-Cooper 1957: 10 (discussion, faunistics); Omer-Cooper 1958b: 40: (discussion); Guignot 1959a: 551, 553, 555 (discussion, description, faunistics); Bertrand and Legros 1971: 244 (faunistics, biology); Rocchi 1991: 86 (faunistics, list); Nilsson and Persson 1993: 80 (faunistics, discussion); Nilsson 2001: 245 (catalogue, faunistics); Pederzani and Rocchi 2009: 95 (faunistics, list); Nilsson 2015: 213 (catalogue, faunistics).
Laccophilus lateralis var. polygrammus Régimbart 1903: 14 (original description, faunistics); Zimmermann 1920a:21 (catalogue, faunistics); Omer-Cooper 1931: 756 (description, biology, faunistics); Guignot 1946c: 264 (description, given as Laccophilus lateralis ab. polygrammus); Guignot 1948: 14 (faunistics, given as *Laccophilus lateralis polygrammus*); Balfour-Browne 1950: 360 (faunistics); Omer-Cooper 1957: 10 (discussion, given as Laccophilus lateralis var. polygrammus); Guignot 1959a: 552, 553, 554 (discussion, description, faunistics); Nilsson and Persson 1993: 80 (list, synonymy, discussion); Nilsson 2001: 245 (catalogue, faunistics, list, synonymy); Nilsson 2015: 213 (catalogue, faunistics, list, synonymy). **Confirmed synonym.**

##### Type localities.

*Laccophilus lateralis*: Madagascar.

Laccophilus lateralis var. polygrammus: Madagascar: Centre-Sud.

##### Type material studied

(3 exs.). *Laccophilus lateralis*: Lectotype (by present designation): male: “Madagascar / Sharp Coll. 1905-313 / *rivulosus* Klug / Type 572 *Laccophilus lateralis* sp. n. Madagascar” (BMNH).

Laccophilus lateralis var. polygrammus: Lectotype (by present designation): male: “Madagascar Centre-Sud Alluaud 1901 67 / male symbol / Cotype / cotype of *polygrammus”* (MNHN). – Paralectotype: “Madagascar Centre-Sud Alluaud 1901 66 / Museum Paris coll. Maurice Régimbart 1908” (1 ex. MNHN).

##### Additional material studied

(37 exs.). **Madagascar**: “Madagascar P. Camboué / Museum Paris, coll. Maurice Régimbart 1908” (1 ex. MNHN; could belong to type material?); “Antsianaka / Museum Paris coll. Maurice Régimbart 1908 / var. polygrammus Rég.” (1 ex. MNHN; could belong to type material?); “Madagascar Ambijoroa Tsaremandroso E.M.C. Callan / *Laccophilus lateralis* Shp. Det. J. Omer-Cooper” (1 ex. AMGS); “Foret de Fito, ex. coll. Breuning” (4 exs. MRAC, 1 ex. MZH; habitus in Fig. 476); “Suberbieville ex. coll. Breuning” (1 ex. MRAC); “F. Dauphin 13.9. 2001 Mandena (QMM area) / Pond at right border of Riv. Amendano, 10 m asl / Gerecke & Goldschmidt leg.” (1 ex. BMNH); “Toli Zombitse, Andranomena R. Pool N:-22.64, E: 44.864, 577 m, 15.5. 2006 Bergsten et al. / BMNH(E) <794176> DNA voucher / *Laccophilus lateralis* Bergsten det.” (1 ex. NHRS); “Toli Zombitse, Ambiamena: stagnant marshland N: -22.86, E: 44.617. 533 m, 14.5. 2006 Bergsten et al. / BMNH(E) <794191> DNA voucher / *Laccophilus lateralis* Bergsten det.” (1 ex. NHRS); “Mah/Tol: Melaky/Menabe, Ambohijanahary NP., S18.26842, E045.46352, 906 m.a.o., 19.12. 2009, 22 w black light, field Bergsten et al. / NHRS-JLBK 0000000722” (1 ex. NHRS); “Fian, Andringitra, Zomandao R.: River edge, bottle trap, N: -22.104, E: 46.92, 1420 m, 9.5. 2006 Bergsten et al. / BMNH(E) <794183> DNA voucher / *Laccophilus lateralis* Bergsten det.” (4 exs. NHRS); “Fian, Ambavalavao, Sendrisoa, Hygropetric, 1164 m, 7.5.2006, Bergsten et al./BMNH(E) <830775> DNA voucher / *Laccophilus lateralis* Bergsten det.” (1 ex. NHRS); Fian, Col des Tapias, Rte Tana-Fianarantsoa: Pond, N: -20.772, E: 47.179, 1717 m, 6.5. 2006, Bergsten et al. / BMNH(E) <794188> DNA voucher / *Laccophilus lateralis* Bergsten det.” (1 ex. NHRS); same data but “N: -20.771, E: 47.18, 1686 m” and <794177> DNA voucher” (1 ex. NHRS); “Mahajanga Melaky betw Morafenobe-Ambohijanahary, S18.19091, E045.19986, 290 m.a.o., 19.12. 2009, water net, field Bergsten et al. NHRS-JLKB” (1 ex. NHRS); “Mahajanga Boeny, Ankarafantsika NP, S16.30271, E046.80995, 75 m.a.o., 8.12. 2009, water net, field, Bergsten et al. / NHRS-JLKB 000000480” (1 ex. NHRS); “Ampamoho near Andilamena 1200-1300 masl, 18-20.1. 1995 Dunay & Janak” (1 ex. NMW, 1 ex. MZH; habitus in Fig. 477); “Prov. Fianarantsoa 7 km W Ranomafana, 1000 m 23-28.2. 1990 Steiner W. E.” (4 exs. USNM, 1 ex. MZH); “Pr. Tamatave, Foret de Perinet 17.7. 1970 / *Laccophilus lateralis* Sharp det. Pederzani” (3 exs. NHMB); “Diego” (Diego Suarez ?) (1 ex. NMW); “Madagascar / *Laccophilus tenuivittis* Rég. type unique / in litt” (1 ex. IRSNB; taxon not described); “Madagascar Goud.” (2 exs. ZMHB).

##### Specimen with uncertain determination.

“NP Ankarrafantsika 5-12.91(?) 2002 Andreev, Dolin & Andreva” (1 female ex. NMPC).

##### Comments on synonymy.

Examination of type material reveals that earlier established synonymy is valid.

##### Diagnosis.

*Laccophilus lateralis* is an externally, variable species, which resembles many of the species located in same species group. Shape of penis is, however, characteristic and separates *Laccophilus lateralis* from all species being externally quite similar. Penis with distinct, asymmetric apex, which is not separated from basal part of penis by a distinct contraction. Instead penis apex merged to basal part and simply divided by a vague incision. *Laccophilus lateralis* (Madagascar) is generally slightly larger than resembling species, occurring in mainland of Africa.

##### Description.

Body length 3.9–4.4 mm, width 2.2–2.4 mm. Dorsal, aspect of body with variable colour pattern (Figs 476–477). Elytra and pronotum dark ferrugineous with minute and vague, paler areas. Pronotum often medially with broad pale area. Elytra with variable, dark ferrugineous, longitudinal markings, which often are rather vague.

Head: Pale ferrugineous to ferrugineous, posteriorly at pronotum a little darker. Submat, finely microsculptured. Reticulation double; in part the two kinds of meshes difficult to distinguish. Larger meshes (when discernible) contain 2–5 small meshes. At eyes (and from eyes towards centre of head) with fine, irregularly distributed punctures.

Pronotum: Pale ferrugineous, frontally and basally in middle with dark ferrugineous to blackish, somewhat vague areas. Pronotum sometimes only with vague, slightly paler medial area. Submat, rather finely microsculptured. Reticulation distinctly double, large meshes contain 2–5 small meshes. Laterally and anteriorly with fine to rather fine, irregularly distributed punctures.

Elytra: Extensively blackish to dark ferrugineous, with moderate, ferrugineous markings. Often, however, with longitudinal dark markings. There is a gradual change between a morph with distinct, dark, elytral markings and an almost totally dark coloured morph, with reduced pale areas (Figs 476–477). Submat, distinctly microsculptured. Reticulation almost simple; two kinds of reticulation are solely distinguished frontally at pronotum and at suture. Separation in two mesh-size-classes obscure because feature very weakly developed. Punctures very fine: four indistinct rows of irregular punctures may be discerned: at suture (punctures very indistinct), a discal, a dorsolateral and a lateral row.

Ventral aspect: Blackish ferrugineous to blackish. Basal segments of abdomen paler; pale ferrugineous to ferrugineous. Rather shiny, although very finely microsculptured. Transverse furrows of metacoxal plates very fine, shallow and hardly discernible. Abdomen in basal half distinctly striated. Almost impunctate. Apex of prosternal process slender and pointed. Apical ventrite asymmetric, as in Fig. 145.

Legs: Pro- and mesotarsus slightly enlarged, with fine but distinct suckers.

Male genitalia: Penis long, in lateral aspect slightly sinuate and in apical half with a distinct enlargement; extreme apex moderately sized, separated by narrow incision (Figs 330–331).

Female: Pro- and mesotarsus slender, slightly extended. Apical ventrite lacks lateral knob (Fig. 146).

##### Distribution.

Madagascar (Fig. 568). Guignot (1946c) reports, besides from Madagascar, the species from some countries on mainland of Africa. Régimbart (1903) gives also Ethiopia. Records outside Madagascar are, however, to be considered uncertain.

##### Collecting circumstances.

Almost unknown. Literature information and available label data are very superficial; collected in pools and ponds.

#### Laccophilus cyclopis

Taxon classificationAnimaliaColeopteraDytiscidae

Sharp, 1882

Figs 147–149 , 332 , 478–480 , 564

Laccophilus cyclopis Sharp 1882: 308 (original description, faunistics); v. d. Branden 1885: 20 (catalogue, faunistics); Régimbart 1894: 237: (description, faunistics); Régimbart 1895: 140 (description, faunistics); Zimmermann 1920a:18 (catalogue, faunistics); Zimmermann 1920b: 225 (faunistics); Gschwendtner 1935a: 18 (faunistics); Guignot 1946c: 269 (description, faunistics); Omer-Cooper 1957: 15, 90 (description, discussion, faunistics); Guignot 1959a: 558, 562, 564 (description, faunistics); Omer-Cooper 1962: 295 (faunistics); Omer-Cooper 1965: 76, 83, 85 (description, biol.); Bertrand and Legros 1967: 862 (faunistics); Curtis 1991: 186 (faunistics); Nilsson 2001: 242 (catalogue, faunistics); Pederzani and Reintjes 2002: 38 (faunistics); Nilsson 2015: 210 (catalogue, faunistics).
Laccophilus shephardi Omer-Cooper 1965: 76, 84 (original description, faunistics); Nilsson 2001: 251 (catalogue, faunistics) Nilsson 2015: 217 (catalogue, faunistics). **New synonym.**

##### Type localities.

*Laccophilus cyclopis*: South Africa: Grahamstown.

*Laccophilus shephardi*: South Africa: Mt Currie district, nr. Zwartberg.

##### Type material studied

(18 exs.). *Laccophilus cyclopis*: Lectotype (by present designation): male: “Type / S. Africa / Grahamstown / Type 573 *Laccophilus cyclopis* sp. Afr. mer. / Sharp Coll. 1905-313” (BMNH). – Paralectotypes: Same data, but “Cotype” (1 ex. BMNH); same data but “Cotype Caffrarie” (1 ex. BMNH); “Cotype / S. Africa / *Laccophilus cyclopis* Sharp Co-type” (3 exs. BMNH).

*Laccophilus shephardi*: Lectotype (by present designation): male: “Syntype / *Laccophilus shephardi* male type / E Cape province Mt Currie Dist. nr Zwartberg 14-XI-1957 J. O-C.” (BMNH). – Paralectotypes: Same as lectotype but labelled as “female type” (1 ex. BMNH); “Cotype / E. Cape Prov. Maclear 9.V.1926 No 275 J. Omer-Cooper /det. J. Omer-Cooper *Laccophilus shephardi* O-C.” (5 exs. AMGS); “E. Cape Prov. Mount Currie 13.II. 1957 No. 400 J. Omer-Cooper” (5 exs. AMGS); “S. Africa E. Cape Prov. Barkley East 14.II. 1948 JOC. / det. J. Omer-Cooper *Laccophilus shephardi* O-C.” (1 ex. AMGS).

##### Additional material studied

(655 exs.). **Namibia**: “Windhoek Town Dam 7.7. 1939 JOC.” (1 ex. AMGS); “Windhoek New Dam 7.7. 1939 JOC.” (1 ex. AMGS); “Windhoek River Bed 9 July 1937” (2 exs. AMGS); “Windhoek 5.7. 1939” (1 ex. AMGS); “Windhoek Eros Mt. 1600 m 22.34S-17.06E / 10.9. 1974 shorewashing Endrödy-Younga: 365” (1 ex. TMSA); “Windhoek Distr., Valencia Ranch / 14-24.4. 1972 Strydom” (1 ex. TMSA); “Osona b. Okahandja 19-20.10. 1991 Göllner leg.” (1 ex. ZMHB); “Osona b. Okahandja p. III. –m IV 1988 leg. J. Irish” (1 ex. ZMHB); “Pools in overflow stream from dam, much weed & algae / Okarupa, ca. 17 mi E of Okahandja 4900 ft, 22.5. 1954 / J. Balfour-Browne” (21 exs. BMNH, 2 exs. MZH); “Okahandja 4700 ft. 22.5. 1954 / Water-hole and seepage through sand J. Balfour-Browne” (8 exs. BMNH, 1 ex. MZH); “Okahandja 4400 ft. 21.5. 1954 Small, deep water-hole, thick *Lemna* cover J. Balfour-Browne” (3 exs. BMNH); “Khomashochl, Farm Wissenfels 23°20'S-16°25'E / 14.9. 1974 shore washing E-Y: 371” (5 exs. TMSA, 1 ex. MZH); “Hardap Dam, Mariental Dist. 10-14. 4. 1972 Strydom & Jones” (6 exs. TMSA); “Hardap GR: Water Institute, pool shore waterplant treating, water catcher 1.12. 1997, 24°29.41S/17°51.52E, leg. M. Uhlig” (9 exs. ZMHB, 4 exs. MZH, 2 exs. NMNW); “Otjozondjupa Dist., Toggekry 250 (Omatako) 21°30'42.9"S, 16°43'56.6"E, 1520 m NN, 8.3. 2003, hand light trap Frisch & Vohland leg.” (1 ex. ZMHB); same data, but “1100 m, 5.3. 2003” (1 ex. ZMHB); “Otjozondjupa Dist., Otjiamongombe West 44, 21°35'44.7"S, 16°56'17.4"E, 1498 m, NN 28.2.2003 hand light trap Frisch & Vohland leg.” (1 ex. ZMHB); “Keetmanshoop Dist., Gellap Ost 3, 23 km NW Keetsmanshoop, dwarf shrub savannah (Nama-Karoo) / 4.4. 2001, 34-28˚C, 26°24'17.7"S, 18°00'41.9” lux 18.00-21.00 leg. Uhlig et al.” (1 ex. ZMHB); “Damaral. Groot Barmen 22.05S-16.40E / 12.9. 1975, shorewashing, Endrödy-Younga: 370” (1 ex. TMSA); Namib Tinkas Dam 22.50S-15.30E / 1.11. 1974 shore washing Endrödy-Younga 440” (1 ex. TMSA); “Grootfontein v. Erffa S.G.” (1 ex. NHMB); “DSWA, ?-erseba 8. 1905 Schultze” (1 ex. NHMB); “DSWA Gr. Etemba Casper S.G. / *Laccophilus cyclopis* Sharp det. Brancucci 1982” (2 exs. ZMHB). – **Botswana**: “Metsimaklaba 7-12.3. 1930 / *Laccophilus cyclopis* Sharp det. J. Omer-Cooper” (1 ex. TMSA); “Tiokweng 15-21.3. 1988 Ward / *Laccophilus cyclopis* Sharp det. Rocchi 1991” (1 ex. CSR). – **South Africa**: “Pretoria, van Son / *Laccophilus cyclopis* Shp det. J. Omer-Cooper” (1 ex. TMSA); “Pretoria 8.2., A.J.T. Janse /*Laccophilus cyclopis* Shp det. J. Omer-Cooper” (1 ex. TMSA); “Pretoria 27.9. 1958 L. Vári” (1 ex. TMSA); “Pretoria 1948” (1 ex. TMSA); “Moorddrift 9. 1924 G. v. Dam / *Laccophilus cyclopis* Shp det. J. Omer-Cooper” (13 exs. TMSA); same data but *“Laccophilus cyclopis* Shp det. J. Omer-Cooper” (1 ex. TMSA); “Transvaal gravel pits Ermelo 8.12. 1948 JOC. / *Laccophilus cyclopis* Sharp J. Balfour-Browne det.” (1 ex. AMGS); “Transvaal gravel pits Ermelo 8.12. 1948” (1 ex. AMGS) “S. Afr. Johannesburg Rivonia 19.XI. 1952 Carpenter” (3 exs. AMGS); “Gauteng, Natalspruit Vlei, Johannesburg, N-26.280, E28.150, 5.6. 1971 Reavell” (4 exs. AMGS); “Transvaal nr Standerton 8.IV. 1954/Pool by muddy stream: algae et grass” (1 ex. BMNH; habitus in Fig. 480); “Transvaal Middleburg 29.11. 1948 JOC.” (2 exs. AMGS); “Middelburg Dam 22.2. 47 JOC” (3 exs. AMGS); “Freddy van Zyl Bridge, Oorsloog Spruit 25.2. 1947 JOC.” (1 ex. AMGS); "Trsvl, Jukskei R at Sandspruit N-26.010, E28.050, 9.2. 1956 Allansaon” (2 exs. AMGS); “Trsvl, Jukskei R, at Alexandra, N-26.100, E28.110, 7.2. 1956 Allanson” (2 exs. AMGS); “Trsvl Sandfonteinspruit at Bramley Bridge N-26.110, E28.070, 14.3. 1956 Allanson” (2 exs. AMGS); “Trsvl., Germiston Lake, N-26.230, E28.170, 12.8. 1971 Reavell” (1 ex. AMGS); “Trsvl, stream, Cowle’s Dam, Brakpan N-26.230, E28.310, 11.8. 1971 Reavell” (1 ex. AMGS); “Transvaal Belfast N. 1948 O-C.” (1 ex. AMGS); “Belfast N. 30. 1948 JOC.” (4 exs. AMGS); “Belfast pond 23. N. 1948 OC.” (4 exs. AMGS); “Belfast 29.11. 1948 JOC.” (5 exs. AMGS); “Transvaal Standerton 8.12. 1948 JOC.” (1 ex. AMGS); “Transvaal Sand R. Pietersburg distr. 27.7. 1948” (3 exs. AMGS); “Transvaal Dynamite Factory Dams Wormsley April 1946” (4 exs. AMGS); “Koster 10. 1924 G. v. Dam / J. Omer-Cooper” (1 ex. TMSA); “pond Breyton rd nr. Chrissie Dec. 1948” (1 ex. AMGS); “Trib. R. Koop nr Barberton 1.12. 1948” (1 ex. AMGS); “Bandolierkop 23. N. 1948 OC.” (1 ex. AMGS); “Transvaal Springsl. (?) 20.6. 1950 G.B.H.” (1 ex. AMGS); “Trsvl nr Standerton / pool by muddy stream, algae & grass J. Balfour-Browne leg.” (1 ex. BMNH); “NW Distr. Klerksdorph 20 km W of Botha Ville, Vaal Riv. 31.1. 2001 Snizek leg.” (2 exs. NMW); “Fount. Grove 17.08 05 C. Swiestrai / *Laccophilus lineatus* Aubé C. Swiestra” (1 ex. TMSA); “NW, 70 km SW Mmabatho Setlagole 21.1. 2000 Halada leg.” (1 ex. NMW); “NW, 50 km S Kimberley, Ritchie 12.1. 2000 Halada leg.” (1 ex. NMW); “TRSVL, trib. of Sand river, Peekes / *Laccophilus cyclopis* Shp det. J. Omer-Cooper” (1 ex. TMSA); “Fountains Pta 5.10. 1932 G. van Son / *Laccophilus cyclopis* Sharp det. Gschwendtner / Omer-Cooper” (2 exs. TMSA); “Trsvl, Pond So Ermelo, Hwy N11, 1.12. 1995 Challet” (1 ex. CGC); “Mpumalanga Ezemvelo, Nat. R. 1374 m, 25.42S-29.01E / 27.1.2004 light trap, Highveld E-Y: 3609 TMSA Staff” (1 ex. TMSA); “OFS Vredefort nr Honingsspruit 29.VIII. 1947 JOC.” (1 ex. AMGS); “O.F.St. Sasolburg 11. 1982 D.M. Kroon” (1 ex. TMSA, 1 ex. MZH); “Van Rhyn’s Pass N. 1933 G. van Son” (1 ex, TMS); “OFS, Parys 4 km E, 26.54S-27.35E/Farm Abel 52, 4-7.12. 1992 M. Krüger leg.” (3 exs. TMSA); “OFS, Entembeni Mission nr Kraansfontein, 30.3. 1991 Reavell” (1 ex. AMGS); “Colesburg Dam Outsloe Town 12.2. 1947” (1 ex. AMGS); “Norvals Point Colesburg 23.2. 1947 JOC.” (6 exs. AMGS); same but “24.2. 1947” (1 ex. AMGS); “van Wyk´s Fontein, Colesburg 23.2. 1947 JOC.” (1 ex. AMGS); “Colesburg, Outside Town 22.2. 1947 JOC.” (1 ex. AMGS); “Kw. Natal, Karkloof R., Mgeni Confluence, N-29.4438, E30.301833, 11.11. 2003 Graham & Dickens” (1 ex. AMGS); “Kw. Natal, Mooi R at Mearns Weir, N-29.250, E29.970, light trap, 5.1. 1996 De Moor & Dickens” (2 exs. AMGS); “Kw. Natal, Mooi R, at Retreat Farm, N-29.270, E29.970, light trap, 4.1. 1996 De Moor & Dickens” (2 exs. AMGS); “Kw. Natal, Mooi R, at Dalcrue Farm, N-29.360, E29.890, light trap, 4.1. 1996 De Moor & Dickens” (5 exs. AMGS); “Kw. Natal, Klein Mooi R, at Dartington Vlei, N-29.260, E29.870, light trap, 5.1. 1996 De Moor & Dickens” (2 exs. AMGS); “Kw. Natal, Mcleod’s Farm nr Dargle, 4.2. 1989 Reavell” (2 exs. AMGS); “Kw. Natal, Ngagane R., Steildrift rd, N-27.770, E30.020, 27.8. 1994 Metz” (1 ex. AMGS); “Kw. Natal, Lions R., N-29.470, E30.150, 4.7. 1995 Dickens” (3 exs. AMGS); “Kw. Natal, Yorkshire Wolds Farm, N-29.3044, E29.773055, 10.6. 1998 De Moor & al.” (5 exs. AMGS); “Natal Engelbrechts Drift Pond, Zaaihoek Dam, 272750S, 300510E, alt. 1740 m, Coke, Ngwenya” (1 ex. CCT); “Natal: Bergville Mont-Aux-Sources 4000 ft. 6.IV. 1954 / farm dam, thick weed at edge / *Laccophilus cyclopis* Sharp J. Balfour-Browne det. VIII, 1958” (1 ex. BMNH); “Natal Kokstad 14.4. 1947 JOC.” (2 exs. AMGS); “Nqutu Zululand 5.8. 1949 Newton” (3 exs. SAMC); “Nqutu Zululand 21.9. 1949 Newton” (1 ex. SAMC); “Durban Natal 20.1. 1903” (1 ex. SAMC); “Natal Bergville Mont-aux-Sources, 4000 ft.6.4. 1954 / farm dam, thick weed at edges J. Balfour-Browne / *Laccophilus cyclopis* Shp det. J. Balfour-Browne” (1 ex. BMNH); “Natal Crocodile R. 13.VI. 1956” (1 ex. AMGS); “Natal, reservoir next of Pickle Pot on R617, 1000 m 2.2. 1997 Turner” (1 ex. CCT); “NC., drying ponds in stream, 12 km SE Garies 26.2. 1997 Challet leg.”(2 exs. CNG, 1 ex. MZH); “CP, Creek 9 km W Ft Beaufort 6.3. 1997 Challet” (1 ex. CNC); “Hutchinson, Cape, Marshall” (1 ex. SAMC); “Kimberley Bro. Power 1.6. 1912” (1 ex. SAMC); “MIL 9 A” (= Scott & Millard 1947 Estuary at Milnerton near Cape Town) (3 exs. AMGS); “U.C.T. FR. W. 72” (1 ex. AMGS); “WC. Pr. Arniston July 1946” (4 exs. AMGS); “ERS 26B” (= WCPr. Krom River 33,56,29S, 18,50,56 E) “8.1. 1953 A.D. Harrison” (2 exs. AMGS); “WC, Franschhoek, rd R45, 25.3. 2001, river 3 km SE Franschhoek Ribera & Cieslak leg.” (1 ex. CIR); “WC Du Toits Kloof rd N1, pond and River Wit in resort 24.3. 2001 Ribera & Cieslak leg.” (2 exs. CIR); “WC Rd 310 before Khayelitsha artificial lagoon 21.3. 2001 Ribera & Cieslak leg.” (1 ex. CIR); “Ceres Pond (964 m), S32°57'59.75” E19°22'59.87” Hidalgo-Galiana & Kleynhans leg.” (1 ex. CIR); “W. Cape, Meerlust dam, farm and fish dam surrounded by indigenous and alien plants, 18.74692E, -34.01554N, alt. 20 m, 2006-2007” (1 ex. CTT); “Victoria West 23.6. 1911 Morris” (2 exs. AMGS); “nr Wellington 300ft. 10.VIII. 1954 J. Balfour-Browne / Deep dam pool with grass and *Juncus* edging / *Laccophilus cyclopis* Sharp J. Balfour-Browne det. IV. 1962” (1 ex. BMNH);“Paarl 16.10. 1949 Malkin / river” (1 ex. BMNH); “ECpr. Gt Fish R. Res. 33;6;59S, 26;39;3E (ECR 732) sm. river dam, clay, vegetation 7. XI. 2008 O. Biström leg.” (1 ex. MZH); “ECPr. Glenstone Farm nr Grahamstown ca. 1400 ft a.s.l. (ECR 731) in Suurberg Quartzite Fynbos, pool below spring 16.XI. 2008 O. Biström leg.” (2 exs. MZH); “Willowmore 21.2. 1947 JOC.” (2 exs. AMGS); “Elliot 11.V. 1956 JOC.” (1 ex. AMGS); “Kowie Riv. nr. Grahamstown Uys leg.” (1 ex. MZH); “C. Prov. Albany District Grahamstown April 1951” (1 ex. AMGS); “Cape Prov. Albany Distr. 5 m. Hedam, Grahamstown April 1951” (1 ex. AMGS); “EC. Ft Fordyce NR, pond, 32°40'S, 26°29'E, 1.12. 2009 P. Bulirsch leg.” (2 exs. NMPC); “WC. Witsenberg Valley in ditch 25.5. 2005 Challet” (1 ex. CGC); “E. C. Pr. Cathcart 3.IV. 1955” (1 ex. AMGS); “E. C. Pr. Cradock July 1946” (2 exs. AMGS); “E. C. Pr. Plutos Vlei 20.VII. 1946 JOC.” (1 ex. AMGS); “E. Cape Dam at head of stream, Lang? Barn? Dist. 14.2. 1948 JOC.” (1 ex. AMGS); “E. C. Pr. Glengrey nr Ladyfrere 6.4. 1955” (1 ex. AMGS); “E. C. Pr. Indwe small pond 6.IV. 1955” (1 ex. AMGS); “E C.Pr. Graaf Reinet Nieu Bethseda 28.1.2001 Snizek leg.” (2 exs. NMW, 1 ex. MZH); “E CPr. Bethesda Rd, Graaf Reinet distr. 29.2. 1941 JOC.” (3 exs. AMGS); “ECPr., 34 km SE Idutywa N of Willowvale, shallow pool, S32°12.577, E28°36.192 alt. 447 m, 23.1. 2005 Bergsten” (1 ex. NHRS); “Barkley East dam at head of Lang Kloof 14.2. 1948” (1 ex. AMGS); “Aliwal North 18-25.8. 1954 H. Andreae” (3 exs. SAMC); “C.Pr.Karoo NP, Mountain View River 32°13.6S, 22°31.6'E, 17.11. 1997, *Phragmites* grass + litter sievings, 900 m Uhlig, Ndamane et Ari leg.” (2 exs. ZMHB); “C.Pr., Karoo NP, pond + shore 13.11. 1993, 32°19'S, 22°30'E, Uhlig leg.” (1 ex. ZMHB; habitus in Fig. 479); “C.Pr., Karoo NP, 32°19'S, 22°30'E, 12-14.11. 1993 Deckert leg.” (1 ex. ZMHB; habitus in Fig. 478); “C.Pr., 20 mi SE Swellendam 3.1. 1951 Brinck-Rudebeck exp.” (6 exs. MZLU); “C.Pr., Albertinia 10.1. 1951 Brinck-Rudebeck Exp.” (4 exs. MZLU); “C.Pr., Zuurberg Pass 15 mi N Addo 16.1. 1951 Brinck-Rudebeck Exp.” (1 ex. MZLU); “C.Pr. Brandvlei 6 mi SW Worchester 11.2. 1951 Brinck-Rudebeck Exp.” (1 ex. MZLU); “C. Pr. nr. Wellington 300 ft. 10.8. 1954 / Deep, dam pool with grass and *Juncus* edging J. Balfour-Browne leg. / *Laccophilus cyclopis* Shp det. J. Balfour-Browne” (1 ex. BMNH); “C.Pr. Heidelberg 4.1. 1994 Wewalka leg.” (1 ex. NMW); “ECPr., Sundays R. below Graaf Reinet, N-32.270, E24.550, 20.2. 1967 Allanson & Forbes” (1 ex. AMGS); “ECPr., Sundays R. at Letskraal, N-32.060, E24.830, 14.2. 1968 Allanson & Forbes” (2 exs. AMGS); “ECPr., Ronan Vlei, Kuntwanazana R., N-31.20.16, E28.03.33, 26.3. 1993 De Moor & Barber-James” (2 exs. AMGS); “ECPr., Mncotsho R, N-32.64.48, E27.36.52, 11.8. 2003 De Moor & Barber-James” (1 ex. AMGS); “ECPr., Mncotsho R, Trib. Buffalo R., N-32.54.43, E27.36.48, 18.5. 2004 De Moor & Barber-James” (1 ex. AMGS); “ECPr., Xolo R., small dam, N-32.52.43, E27.37.05, 10.12. 2002, De Moor” (1 ex. AMGS); “Karree Douw Pass, Humansdorp, 17.2. 1947” (1 ex. AMGS); “Ca. 12 mi from Queenstown on road to Lady Frere, ca. 3800 ft., 26.3. 1954 / in small grassy dam / J. Balfour-Browne” (5 exs. BMNH, 1 ex. MZH); “ECPr. ca 5 km N Queenstown, Alan Marsh Prop, small pond, muddish water, S31°48.859, E26°49.715 alt.1208 m 19.1. 2005 Bergsten” (11 exs. NHRS); “ECPr., ca. 5 km N Queenstown, Alan Marsh Prop., muddy pond with grass beds, S31°48.861, E26°50.067, alt. 1232 m, 19.1. 2005 Bergsten” (17 exs. NHRS); same data but “muddy pond with sand and grass bed, S31°48.887, E26°49.529, alt. 1191 m” (5 exs. NHRS); “ECPr., Ca. 2 km N Queenstown, Longhill Game Reserve, pond with muddy water, S31°51.317, E26°51.322, alt. 1175 m, 19.1. 2005 Bergsten” (3 exs. NHRS); “EC., stream NW Burgersdorp 18.5. 2005 Challet” (1 ex. CGC); “EC., road to Venterstad from Burgersdorp 18.5. 2005 Challet” (2 exs. CGC); “EC., muddy pond So of Burgersdorp 18.5. 2005 Challet” (2 exs. CGC); “Nyebo Stream Transkei 5.4. 1947 JOC.” (1 ex. AMGS). “ECPr. Hogsback 32.35S 26.56E, 6.9. van Noort” (1 ex. SAMC); “ECape prov, King Williams Town 4.4. 1947” (1 ex AMGS); “ECPr., close to Dwesa nature Reserve, muddy pond with vegetation edges S32°18.582, E28°49.002, alt. 76 m, 24-25.1. 2005 Bergsten leg.” (1 ex. NHRS); “EC., 78.7 km So Tarkastadr, rain pond, 21.5. 2005 Challet leg.” (1 ex. CGC); “EC., Gt Brak R., Hwy 56 at Schoombe, 17.5. 2005 Challet leg.” (2 exs. CGC); “EC., Pond 20 km No. Adelaide Hwy 344, 29.8. 2004 Challet” (3 exs. CGC); “EC., stream So of Adelaide 17.5. 2005 Challet” (1 ex. CGC); “ECPr. Farm at Bedford, N-32.670, E26.050, 5.12. 1972 Stuart & Greig” (15 exs. AMGS); “ECPr., 3 km N Sterkstroom, Ivan Hansen Prop., small shallow pond, S31°30.337, E26°32.523, alt. 1380 m, 20.1. 2005 Bergsten” (5 exs. NHRS); “ECPr, 2 km N Sterkstroom, Ivan Hansen Prop., small pond with vegetation S31°30.233, E26°32.160, alt 1414 m 20, 28.1. 2005 Bergsten leg.” (5 exs. NHRS); “ECPr. 3 km N Sterkstroom, Ivan Hansen Prop., small, shallow pond, S31°30.337, E26°32.523, alt. 1380 m, 20.1. 2005 Bergsten” (3 exs. NHRS); “ECPr., 2 km N Sterkstroom, Stretton Prop., vegetation rich pond (at sides and in water), S31°29.221, E26°43.984, alt 1533 m, 21.1. 2005 Bergsten” (7 exs. NHRS); “ECPr., N of Sterkstroom, small pond, S31°31.297, E26°32.259, alt.1388 m, 28.1. 2005 Bergsten” (4 exs. NHRS); “ECPr., N of Sterkstroom, Large dam with vegetation at edges, S31°28.571, E26°29.602, alt. 1975 m, 29.1. 2005 Bergsten” (1 ex. NHRS); “EC., pond @HwyR350, No Hellsport Pass 8.12. 1995 Challet” (6 exs. CGC, 1 ex. MZH); “SA NW, CPr., 1 km No Palersheuwel, 28.2. 1997 Challet” (5 exs. CGC, 1 ex. MZH); “EC., rain pond 20 km SO Indutwe 29.8. 2004 Challet” (3 exs. CGC); “ECPr., 7 km E Idutywa, small dirt pool, S32°07.169, E28°22.563, alt. 772 m, 23.1. 2005 Bergsten” (4 exs. NHRS); “ECPr., S31°24.592, E26°54.700, alt. 1745 m, Bradgate Wilson prop., Large Dam, Ca. 15 km W Dordrecht 22.1. 2005 Bergsten” (78 exs. NHRS, 5 exs. MZH); “Cape Col. Victoria West / P.D. Morris coll.” (2 exs. NMW); “Somerset West 28-29.12.1991 Mazzoldi / Marshy area nr Firgrove / *Laccophilus cyclopis* Shap. det. Rocchi” (1 ex. CSR); “WCPr., Somerset West Mall, Azolla pond, 28.3.2005 Reavell” (1 ex. AMGS); “Cape Town” (4 exs. SAMC); “WCape, pond, Cape Good Hope Res. 26.2. 1997 Turner” (2 exs. CCT); “WC, Townsriver rd, N Guydo Pass, Ceres, N-33.10.27, E19.23.28, sandy stream, 3.9. 2003 Turner, Mann & Reavell” (12 exs. CCT); “CPr., pools in riverbed, Williston to Calvinia on R65, Gt Karoo 11.2. 1997 Turner” (5 exs. CCT); “WCp., reservoir on roadside, Bordjilesri, C. G.H. Res., Cape Town 15..2. 1997 Turner” (1 ex. CCT); “WCPr., Zekoevlei Fm, 14 ks S Bremersdorp, Cape L’Algulhas, river, 25.2. 1997 Turner” (1 ex. CCT); “WC, E barrydale on R62, River, slow flowing, silty with sandy rock bottom and emergent veg. N-33.49.05, E20.53.37, 386 m, 4.9. 2003 Turner. Mann & Reavell” (2 exs. CCT); WC. Reservoir, Road from Ouplas to Bredasdorp, N-34.27.02, E20.10.51, 62 m, 10.9. 2003 Turner. Mann & Reavell” (1 ex, CCT); “WC, sandy bottomed stream N Guydo Pass on Toawsrivier rd., N-33.10.27, E19.23.28, 305 m, 3.9. 2003 Turner. Mann & Reavell” (5 exs. CCT); “WC. De Hoop Res., N-34.27.23, E20.26.19, 13 m, 9.9. 2003, Turner. Mann & Reavell” (63 exs. CCT, 10 exs. MZH); “CPr., Stellenbsch, Fauna Aponogeton Vlei 18.9. 1994 Reavell” (1 ex. CCT); “WCPr., Reservoir on R316 ca 20 km S Caledon N-34.15.86, E19.35.54, 122 m, 10.9. 2003 Turner, Mann, Reavell” (17 exs. CCT); “WCPr. Reservoir, Rd N15 E Worcester, N-33.37.05, E19.21.45, 4.9. 2003 Turner, Mann, Reavell” (1 ex. CCT); “WC., River to E Ashton on Mnt. Rd, N-33.49.35, E20.05.31, 263 m, Turner, Mann, Reavell” (3 exs. CCT); “CP., Cape Francis in ditch, 7.12. 1995 Challet” (1 ex. CGC); “C.Pr. Simons Town 12-20.4. 1915 Cameron leg.” (1 ex. BMNH); “C.P. Grootvaderbos 1-6.11. 1940 G. van Son / *Laccophilus cyclopis* Shp det. J. Omer-Cooper” (9 exs. TSMA); “Cape, swampy dam nr Pot R., N-31.990, E28.260, 27.3. 1993 De Moor & al” (2 exs. AMGS); “WCPr., Klein Berg R., N-13.30.03, E18.58.35, 26.4. 1951 Harrison” (2 exs. AMGS); “WCPr., Muizenberg, Pond, N,-33.9505, E, 18.510, 3.1. 1962 M Harrison” (4 exs. AMGS); “Cape, Kroomie R. Ft. Baeufort-Adelaide Rd., N-32.77, E26.43, 5.12.1992 Stuart & Greig” (5 exs. MZH); “Cape, Gorah Farm Dam, N-33,63, E26.610, 30.10. 1972 Stuart & Greig” (1 ex. AMGS); “Cape, Wit R at Slagbloom, N-33.370 E25.670, 20.10. 1972 Stobbs” (1 ex. AMGS); “Cape, Stream, 19 km of Brand R Rd., N-33.890, E21.060, 30.9.1972 Stobbs” (6 exs. AMGS); “Still Bay C.P. 9-12.11. 1940 G. van Son / *Laccophilus cyclopis* Sharp det. J. Omer-Cooper” (1 ex. TMSA); “Capland Willowmore Dr. Brauns” (5 exs. TMSA); “Golden Gate G.G.H.N.P. 16-25.1. 1968 Potgieter & Jones” (1 ex. TMSA). – **Lesotho**: “Teyateyaneng 2.12. 1955 JOC.” (9 exs. AMGS); “Basutoland Maputomy ?, small dam 7.6. 1956 G. Ewer” (1 ex. AMGS); “Sani Pass, stream, riffles and pools, 2864 m 1.2. 1997 Turner” (6 exs. CCT).

##### Specimens with unknown country association.

“Africa / J. Hope Coll. B.M. 1948-217” (4 exs. BMNH).

##### Comments on synonymy.

Type material of *Laccophilus cyclopis* and *Laccophilus shephardi* have been examined and compared. *Laccophilus cyclopis*, being a quite widely distributed species exhibits distinct variation in appearance of dorsal colour pattern of body. Being so, it is evident that *Laccophilus shephardi* represents a colour morph of the species. We have not observed the differences in male genitalia and last abdominal ventrite of male, stressed by Omer-Cooper in the original description of *Laccophilus shephardi*. Accordingly, we consider *Laccophilus cyclopis* and *Laccophilus shephardi* as synonyms. *Laccophilus cyclopis*, which is the older name, is the valid name of this species.

##### Diagnosis.

*Laccophilus cyclopis* is a distinct, although, externally a variable species. It resembles quite much of species exhibiting distinct, dark, longitudinal, elytral markings, e.g. as *Laccophilus incrassatus* and *Laccophilus quindecimvittatus*. Longitudinal markings, however, when present, are strongly undulate in *Laccophilus cyclopis*. In some specimens of *Laccophilus cyclopis* elytral dark markings strongly reduced and are lacking. Double elytral reticulation exhibited by *Laccophilus cyclopis* is also a useful feature at identification because large meshes often strongly reduced in a number of close species, and microsculpture appears in such cases, simple or almost simple.

##### Description.

Body length 3.8–4.8 mm, width 2.2–2.6 mm. Body dorsally with distinct, somewhat variable colour pattern. Dark longitudinal irrorations of elytra rarely strongly reduced (Figs 478–480).

Head: Pale ferrugineous. At pronotum, generally with vague darker area. Rather shiny, although distinctly microsculptured. Reticulation double; finer meshes often reduced, and hardly visible. At eyes, with minute irregular punctures.

Pronotum: Pale ferrugineous. Frontally and posteriorly often extensively darker; dark areas sometimes absent. Rather shiny, although distinctly microsculptured. Reticulation double; fine meshes in part indistinct or totally absent. At margins, with fine, scattered punctures.

Elytra: Pale ferrugineous with dark ferrugineous, almost uniform, longitudinal irrorations (Fig. 475). Irrorations sometimes reduced (Figs 478–480). Submat, with distinct reticulation which is double. In general, large meshes contain 3–8 fine meshes. Sporadic fine punctures form vague, longitudinal, punctured areas (not distinct rows). Lateral punctate area continues towards apex as a densely punctate and pubescent row.

**Figures 147–159.**
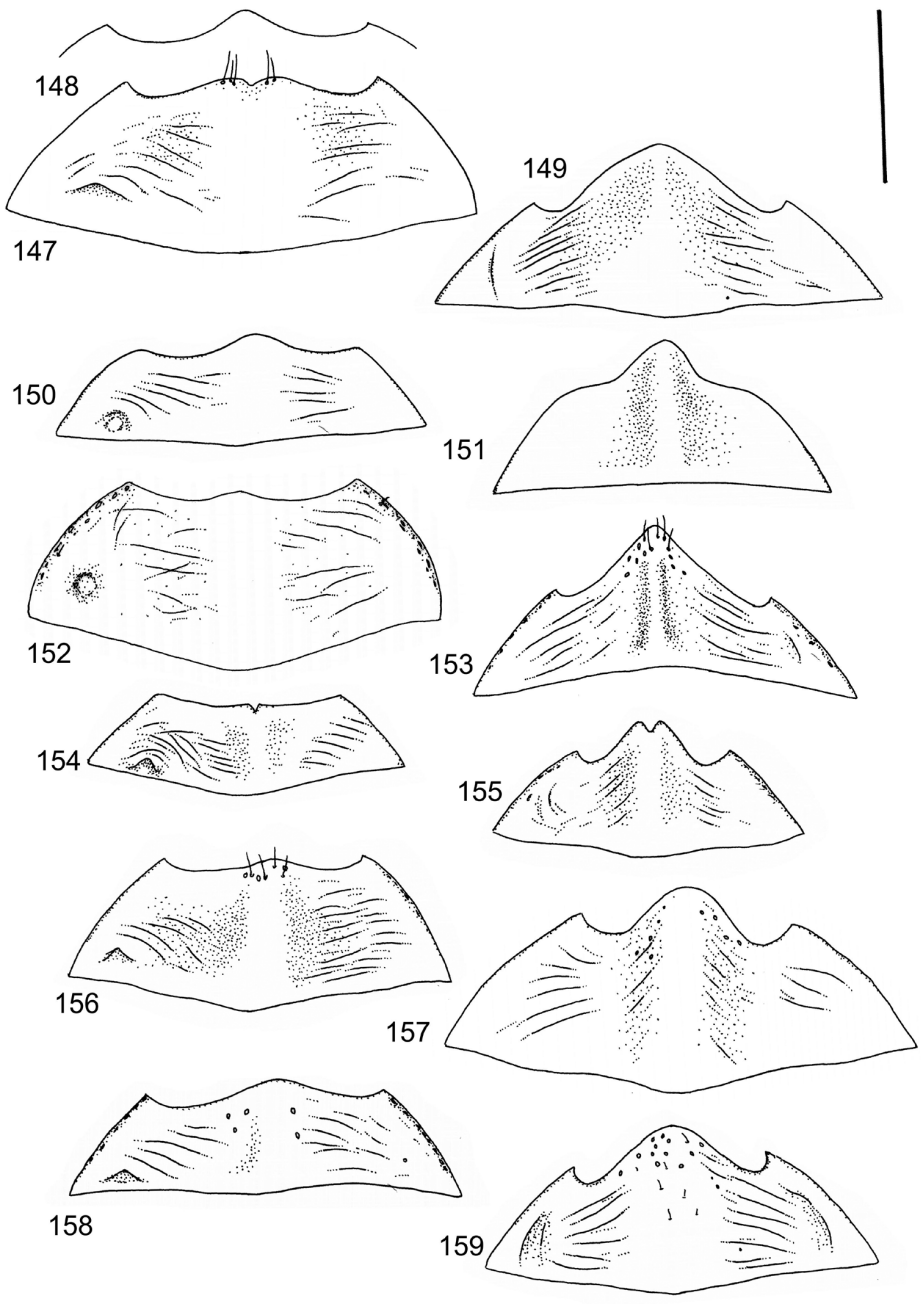
Apical ventrites **147** *Laccophilus cyclopis* male, **148** same, but view from other direction, and **149** female **150** *Laccophilus adjutor* male, and **151** female **152** *Laccophilus necopinus* male, and **153** female **154** *Laccophilus conjunctus* male, and **155** female **156** *Laccophilus brownei* male, and **157** female **158** *Laccophilus contiro* male, and **159** female. Scale bar 0.5 mm.

Ventral aspect: Pale ferrugineous, metathorax and base of metacoxal plates slightly darker; colour variation vague. Submat, finely microsculptured. Metacoxal plates with some transversely located shallow depressions. Base of metacoxal plates somewhat elevated in comparison with base of abdomen. Abdomen densely striated. Apical ventrite (Figs 147–148); apex bifid and on one side provided with a low knob. Prosternal process long, slender and pointed.

Legs: Pro- and mesotarsus long, at base slightly enlarged, narrows gradually towards apex. Segments provided with distinct suckers.

Male genitalia: Penis in lateral aspect broad, from base to apex slightly curved; extreme apex formed as a short, medium broad and apically, sharp extension (Fig. 332).

Female: Apical ventrite symmetric, lacks knob and apex not bifid (Fig. 149). Pro- and mesotarsus slender.

##### Distribution.

Namibia, Botswana, South Africa, Lesotho (Fig. 564). Omer-Cooper (1965) adds Swaziland. Records outside southern Africa are considered doubtful.

##### Collecting circumstances.

According to label data, the species has been sampled in dams, pools, water-holes etc., with various, often thick vegetation of weed, algae, grass, *Juncus* and *Lemna*. Also collected at light. Omer-Cooper (1965) reports the species as occurring “in every type of fresh water habitat”.

#### Laccophilus adjutor

Taxon classificationAnimaliaColeopteraDytiscidae

Guignot, 1950

Figs 150–151 , 333–334 , 481 , 564

Laccophilus adjutor Guignot 1950b: 271 (original description, faunistics); Guignot 1955c: 182 (faunistics); Omer-Cooper 1957: 8, 9, 10, 90 (description, faunistics, discussion); Omer-Cooper 1958a: 59 (faunistics); Omer-Cooper 1958b: 37, 38, 39 (description, faunistics); Guignot 1959a: 550, 552, 556 (discussion, description, faunistics); Guignot 1959d: 161 (discussion); Omer-Cooper 1965: 77, 79 (discussion, earlier records of *Laccophilus adjutor* by Omer-Cooper belong to *Laccophilus necopinus* Guign.); Omer-Cooper 1967: 58 (discussion); Nilsson and Persson 1993: 80, 94 (discussion, faunistics); Nilsson 2001: 240 (catalogue, faunistics); Nilsson 2015: 208 (catalogue, faunistics).

##### Type locality.

Uganda: Ounyoro province, Kadjoura marsh.

##### Type material studied

(2 exs.). Holotype: male: “Ouganda Ounyoro Marais Kadjoura pres Hoima Ch. Alluaud 1909 / Male symbol / Paratype” (MNHN). [Comments: the original description lists only holotype and allotype and no paratype and accordingly type material consists of two specimens both from same locality. One additional specimen is provided with a holotype label but it does not fit with given type locality (see below). The male specimen with a paratype label, however, fits with given type locality and obviously this specimen is the real holotype. This enigmatic situation is considered a case of mislabeling.] Allotype (= paratype): female: Same data as holotype, but “Allotype / female symbol / *Laccophilus adjutor* Guign. Allotype, female symbol” (1 ex. MNHN).

##### Additional material studied

(40 exs.). **Nigeria**: “Stream of Assab 36 mi from Jos 13.4. 1963 JOC” (1 ex. AMGS); “Stream, escarpment, road Jos-Wambe 13.4. 1963 JOC.” (1 ex. AMGS). – **Uganda**: “Ouganda Occidental Province de Toro env. de Fort Portal Ch. Alluaud 1909 / male symbol / Type / *Laccophilus adjutor* Guign. Type male symbol” (1 ex. MNHN; not holotype, see above); “Prov. d’Ounyoro Albert Nyanzas.-e Rivière Mousisi Alluaud 1909 / février / Guignot det. *Laccophilus adjutor* sp. n. / paratype” (2 exs. IRSNB); “Ounyoro Marais Kadjoura près Hoima Alluaud 1909 / paratype” (2 exs. IRSNB); “Paratype / Mus. Paris Ouganda Ounyoro Marais Kadjoura pres Hoima Ch. Alluad 1909 / F. G. det. 57 *Laccophilus adjutor* Guign.” (1 ex. AMGS). [Comment: not type material; later obviously provided with paratype labels.] “Uganda Kibale K 15, 12.9. 1991 Nummelin leg.” (2 exs. MZH); Same but “6.9. 1991” (1 ex. MZH); “W Prov. Kibale Forest, swamp K 14, 8.4. Nummelin leg.” (1 ex. MZH); “Kampala 9.12. 1929 Hopkins” (2 exs. BMNH, 1 ex. MZH); same but “4.9. 1929” (1 ex. BMNH). – **Kenya**: “B.O.A. Kibwezi 26.11. 1907 Scheffler” (1 ex. ZMHB). – **Zaire**: “Parc National Albert, 2.3. 1957 P. Vanschuytbroeck/Secteur Nord r. dr. Moyenne-Lume affl. dr. Semliki 1340 m” (8 exs. MRAC, 2 exs. MZH; habitus in Fig. 481); same but “10.10. 1957/Secteur Nord riv. Lutakira, affl. dr. Semliki 910 m” (1 ex. MRAC); same but “26.8. 1956/Secteur Nord May ya Moto 1320 m” (2 exs. MRAc); same but “27.8. 1957/Secteur Nord rive de Semliki, rte Muramba, 905 m” (3 exs. MRAC); same but “23.8. 1957/Secteur Nord, rive dr. Semliki, rte Muramba, 905 m”(1 ex. MRAC); same but “11.2. 1957/Secteur Nord Katamangu affl. g. Butahu 1300 m.” (1 ex. MZh); same but “27.8. 1957/Secteur Nord, river Ihunga, af. dr. Semliki 1300 m” (1 ex. Mrac); same but “6.12. 1956/Secteur Nord, Lume, affl. dr Semliki route Beni-Katwe 1000 m” (1 ex. MRAC); same but “19.9. 1955, 2690 m (ex. plankton)/Secteur Nord, Lusilube –affl. Semliki-Piste Mwenda-Katuka alt. 1860 m” (1 ex. MRAC). – **Tanzania**: “DOA, 1.9. 1911 W Ruanda 1850 m Sümpf, Meyer” (1 ex. NHMB). – **Angola**: “Luanda Airport-Catete Road 21 km, 23-25.8. 1949 Malkin B. / small deep pond, sand and gravel bottom” (1 ex. BMNH).

##### Diagnosis.

*Laccophilus adjutor* resembles much of especially *Laccophilus necopinus* and *Laccophilus conjunctus* in regard of male genitalia and external appearance. From *Laccophilus necopinus*, the species is separated by having a shorter and more curved penis. From *Laccophilus conjunctus*, *Laccophilus adjutor* is separated by having, in general, slightly larger body and by elytral, dark colour pattern, which is formed as separate longitudinal markings. Elytra of *Laccophilus conjunctus* are predominantly dark with limited pale markings. The male genitalia of *Laccophilus adjutor* and *Laccophilus conjunctus* are quite similar and future studies may show that the two species are conspecific.

##### Description.

Body length 3.4–3.8 mm, width 1.8–2.0 mm. Habitus and dorsal colour pattern (Fig. 481). Exhibits only slight variation in dorsal colour pattern.

Head: Pale ferrugineous to ferrugineous. Finely microsculptured; reticulation indistinctly double. Large meshes only slightly more strongly developed than small meshes. Large meshes, when discernible, contain 3–6 small meshes. Almost impunctate, except at eyes where some irregularly distributed fine punctures are discernible.

Pronotum: Pale ferrugineous, frontally and mediobasally blackish to dark ferrugineous. Reticulation indistinctly double; large meshes generally contain 3–6 minor meshes. Very finely punctate. Punctures irregularly distributed and partly indistinct; densest at foremargin and laterally.

Elytra: Pale ferrugineous with blackish to dark ferrugineous colour pattern; pale subbasal area slightly irregular. Posteriorly, with irregular, longitudinal markings which are sometimes partly confluent (Fig. 481). Finely and densely microsculptured, submat; with double reticulation but large meshes strongly reduced, hardly discernible. Very fine, irregularly distributed punctures discernible. Lateral, pre-apical furrow shallow, finely pubescent.

Ventral aspect: Blackish ferrugineous to dark ferrugineous, except prothorax, pale ferrugineous to ferrugineous. Submat, with very fine microsculpture. Basal ventrites with curved striae. Almost impunctate. Transversely located, shallow furrows on metacoxal plates in anterior half rather distinct; in posterior half indistinct, almost absent. Prosternal process rather slender, posteriorly somewhat extended, apically pointed. Apical ventrite; asymmetric with one lateral knob (Fig. 150).

Legs: Protarsus rather slender and protarsal claws slender, moderately curved and somewhat extended. Pro- and mesotarsus provided with suckers.

Male genitalia: Penis in lateral aspect almost straight; extreme apex with a small, rounded extension; external outline of penis in apical portion provided with a distinct membranous part (Figs 333–334).

Female: Apical ventrite medioapically somewhat extended and keeled (Fig. 151). Pro- and mesotarsus slender.

##### Distribution.

Nigeria, Uganda, Kenya, Zaire, Tanzania, Angola (Fig. 564). Only personally verified records are mapped because of widespread earlier confusion in deciding the species-identity.

##### Collecting circumstances.

Almost unknown.

#### Laccophilus necopinus

Taxon classificationAnimaliaColeopteraDytiscidae

Guignot, 1942

Figs 152–153 , 335–336 , 482 , 569

Laccophilus necopinus Guignot 1942: 15 (original description, faunistics); Guignot 1946c: 264, 266, 313 (description, faunistics); Guignot 1953b: 234 (faunistics); Guignot 1954a: 26 (faunistics); Guignot 1955a: 29 (discussion); Guignot 1959a: 552, 555 (description, faunistics); Omer-Cooper 1965: 77, 79 (discussion, faunistics); Omer-Cooper 1967: 58 (discussion); Nilsson 2001: 247 (catalogue, faunistics); Nilsson 2015: 214 (catalogue, faunistics).

##### Type locality.

Uganda: Musisi River.

##### Type material studied

(3 exs.). Holotype, male: “Prov. d’Ounyoro Albert Nyanza S.-E. Riviere Mousisi Ch. Alluaud 1909 / fevrier / Male symbol / Type / *Laccophilus necopinus* Guign. Type male” (MNHN). – Paratypes: Same data as holotype, but labelled “Paratype” (1 ex. MNHN); same data as holotype, but labelled “Paratype” and provided with female symbol (1 ex. MNHN, habitus in Fig. 482).

##### Additional material studied

(98 exs.). **Nigeria**: “R. Kaduna 4,5 mi. from Jos 13.4. 1963 JOC.” (2 exs. AMGS). – **Cameroon**: “Dschang 7-14.11. 1967” (1 ex. NHMB); “Ngoundere 30.8. 1969” (1 ex. NHMB). – **Zaire**: “PNG, Pali/8, 22.3. 1952 De Saeger” (1 ex. MRAC); same but “II/gd/11, 10.8. 1952” (1 ex. MRAC); same but “Ndelele/14s, 1.8. 1952” (1 ex. MRAC, 1 ex. MZH); same but “K 117/14s, 19.3. 1952“(1 ex. MRAC); “Elisabethville, à la lumière 1. 1956-1. 1957 Seydel” (3 exs. NHMB); same but “1953-1955” (1 ex. NHMB); “Ruhengeri (s. Kiril) 31.8.-3.9. 1934 G.F. de Witte. Parc Nat. Albert” (2 exs. NHMB). – **Uganda**: “Kampala 13.Vii. 1929 G.H.E. Hopkins / *Laccophilus adjutor* Guign. Det. J. Omer-Cooper” (1 ex. AMGS); “Kampala 11.VII. 1929 H. Hargreaves” (1 ex. AMGS); “Monts Ruwenzori (zone Inf.) Roubona 1500-1600 m Alluaud 1909 / Janvier” (2 exs. MNHN). – **Kenya**: “Bassin du Tana Thika Alluaud 1909 / *Laccophilus necopinus* Guign. Parartype” (1 ex MNHN [Comment: labelled as paratype but specimen is not mentioned in the original description]); “15 km N Nyahurutu, small lake 6.2. 1995 leg. Travnicek” (1 ex. NMPC); “L. Naivasha, Fisherman’s Camp 14.2. 1995 leg. Travnicek” (1 ex. NMPC). – **Rwanda**: “Cyangugu Gishoma 14.2. 1983 Mühle” (1 ex. NHMB). – **Tanzania**: “Rungwe X. 1948 JOC.” (3 exs. AMGS); “Kilimandjaro Sjöstedt / Kibonoto 1-1200 m” (1 ex. NHRS); “Usa River 3900 ft. Szunyoghy / light trap 15.11.-31.12. 1965” (1 ex. CGW). – **Malawi**: “R. Mtiti 1.10. 1948 J.OC.” (6 exs. AMGS); “6 mi N of R. Mtiti, stream 2.10. 1948 JOC.” (9 exs. AMGS); “Dedza-Lilongwe rd. Stream 30.IX. 1948” (1 ex. AMGS); “R. Diedma Lilongwe rd. 30.9. 1948” (2 exs. AMGS); “dam in lower Lilongwe rd 29.9. 1945” (2 exs. AMGS); “Dedza, hotel dam, 29.9. 1948” (3 exs. AMGS). – **Zimbabwe**: “Inyanga N. 1948 JOC.” (3 exs. AMGS); “Vamba Nat. Park 31.XII. 1962” (3 exs. AMGS); “Stream at Salisbury 17.IX. 1948” (3 exs. AMGS); “Stream at Salisbury 1948” (5 exs. AMGS); “Marandellas 2 N. 1948 JOC.” (7 exs. AMGS); “Stream Rusapi 13.XI. 1948 / *Laccophilus adjutor* Guign. Det. J. Omer-Cooper” (3 exs. AMGS). – **South Africa**: “Transvaal Ermelo stream 7.Dec. 1948 JOC.” (2 exs. AMGS); “Trsvl 5 mi W Warmbad 24-25.2. 1968 Spangler” (1 ex. USNM); “Trsvl, pond, rd to Stoffleberg 10.12. 1995 Challet” (1 ex. CGC, 1 ex. MZH); “Trsvl, Hwy 555 No. Stoffberg 10.12. 1995 Challet” (1 ex. CGC); “Nelspruit, pond 27.4. 2010, S25°32'13,83”, E30°59'50,35” Hidalgo, Galiana & Kleynhans leg.” (1 ex. CIR); “Kw. Natal, Lions R nr N-29.471, E30.150, 4.70 Dickens“ (1 ex. AMSG); “Kw. Natal, Buffalo R. Cloontarf, -27.630N, E29.98, 23.9. 1974 Metz” (2 exs. AMGS); “Kw. Natal, Ngagne R, Steildrift, N-27.770, E30.02, 24.9. 1974 Metz” (1 ex. AMGS); Kw. Natal, Ngagagne R’ St. 19.3. 1974 Metz” (1 ex. AMGS); “Natal, Dragon Peaks Park, 29°02'S-29°26'E, 1150-1450 m, 9-12.11. 1993 Deckert leg.” (1 ex. ZMHB, 1 ex. MZH); “Nqutu 1953 Newton” (1 ex. BMNH); “Natal, (handwritten, unreadable locality text), 25.9. 1967 Omer-Cooper” (4 exs. AMGS); “E.C.Pr. Dias Cross dune slack pond, 334300S 263730E 27.3. 1994 E. Bruce-Miller” (1 ex. AMGS). – **Swaziland**: “Mbabane 5.12. 1948 JOC. / *Laccophilus necopinus* Guignot det. G. Challet 06” (1 ex. AMGS).

##### Diagnosis.

*Laccophilus necopinus* resembles most of *Laccophilus adjutor* and *Laccophilus conjunctus*. The species is distinguished by apex of penis, which differs clearly from the two other species; extreme apex of *Laccophilus necopinus* is distinctly broader and clearly expanded on one side. See also diagnosis of *Laccophilus adjutor* (p. 198).

##### Description.

Body length: 3.8–4.3 mm, width 2.1–2.4 mm. Elytral colour pattern slightly variable; longitudinal, dark markings sometimes merged into a larger dark area (Fig. 482).

Head: Pale ferrugineous. Posteriorly, with vague, dark ferrugineuos area. Almost impunctate, except at eyes with fine, irregular punctures. Areas with punctures slightly extended towards middle of head-discussion. Rather shiny, finely microsculptured. Reticulation indistinctly double. Large meshes contain 2–5 fine meshes.

Pronotum: Pale ferrugineous. Frontally and posteriorly in middle with dark ferrugineous to dark brown area. Almost impunctate, except laterally and anteriorly, with scattered fine punctures. Submat, finely microsculptured. Reticulation double but size categories in part difficult to separate. Large meshes reduced and only partially discernible.

Elytra: Pale ferrugineous, with dark ferrugineous to dark brown to blackish, longitudinal markings, which sometimes merge into each other. At base, with distinct pale, transverse area (at suture elytra, however, dark coloured). Rarely pale areas strongly reduced; only minor spots present (Fig. 482). Submat, finely and densely microsculptured. Reticulation appears simple, but very fine, scattered fragments of large meshes may be discerned. Almost impunctate; very fine, scattered punctures form a vague, discal, dorsolateral and lateral row of punctures.

Ventral aspect: Dark ferrugineous to ferrugineous, prothorax paler, pale ferrugineous. Almost impunctate. Submat to rather shiny, very finely microsculptured. Abdomen with curved striae. Metacoxal plates with approx. 10 shallow furrows. Prosternal process quite slender, apex extended and pointed. Apical ventrite asymmetric, with lateral, rounded knob (Fig. 152).

Legs: Pro- and mesotarsus slightly enlarged, extended and provided with distinct suckers.

Male genitalia: Penis long, in lateral aspect almost straight; extreme apex produced to a quite broad extension, being somewhat expanded on one side. In apical half externally, with a membranous area (Figs 335–336).

Female: Externally resembles much of male. Pro- and mesotarsus are more slender. Apical ventrite almost symmetric, lateral knob lacking (Fig. 153).

##### Distribution.

Nigeria, Cameroon, Zaire, Kenya, Uganda, Rwanda, Tanzania, Malawi, Zimbabwe, South Africa, Swaziland (Fig. 569).

##### Collecting circumstances.

Rather insufficiently known. Label data give stream and pond as collecting localities.

#### Laccophilus conjunctus

Taxon classificationAnimaliaColeopteraDytiscidae

Guignot, 1950

Figs 154–155 , 337–338 , 483–484 , 567

Laccophilus lineatus ab. conjunctus Guignot 1946c: 264 (description, faunistics).
Laccophilus conjunctus Guignot 1950b: 272 (original description, faunistics, discussion); Guignot 1953b: 236 (discussion); Guignot 1954: 24 (faunistics); Guignot 1959a: 534, 537, 541, 542, 578 (description, discussion, faunistics); Medler 1980: 155 (catalogue, faunistics); Nilsson 2001: 242 (catalogue, faunistics); Nilsson 2015: 210 (catalogue, faunistics).

##### Type locality.

Cameroon: Yaoundé.

##### Type material studied

(4 exs.). Lectotype (by present designation): male: “Cameroun Yaoundé Vadon! / male symbol / Type” (MNHN). – Paralectotypes: Same data as lectotype but “female symbol / Paratype” (1 ex. MNHN; habitus in Fig. 484); same data as preceding paralectotype but labelled “Ebolowa” (1 ex. MNHN); “Nanga-Eboko Cameroun II. 1937 – Andr. / female symbol / Paratype” (1 ex. MNHN; habitus in Fig. 483).

##### Additional material studied

(7 exs.). **Zaire**: “PNG, Morubia/9, 12.3. 1952 De Saeger, 3187” (3 exs. MRAC, 1 ex. MZH); same data but “II/gd/11, 10.4. 1952, 3314” (1 ex. MRAC, 1 ex. MZH); same data but “Pali’’/11, 25.7. 1952, 3831” (1 ex. MRAC).

##### Diagnosis.

Close to *Laccophilus necopinus* and especially to *Laccophilus adjutor*, from which *Laccophilus conjunctus* is separated by smaller body size and by dorsal colour pattern; elytral pale areas strongly reduced in *Laccophilus conjunctus*. Penises of *Laccophilus adjutor* and *Laccophilus conjunctus* are almost similar, but minor differences exhibited in shape and outline of extreme apex; almost obtuse in *Laccophilus conjunctus* vs. rounded in *Laccophilus adjutor*. External outline of membranous area in apical half of penis shows also minor differences, being slightly sinuate in *Laccophilus adjutor*. Further studies will show if the two species are conspecific.

##### Description.

Body length 3.2–3.5 mm, width 1.7–1.9 mm. Dorsal, colour pattern of body slightly variable; pale areas reduced to a few spots and a subbasal area (Figs 483–484).

Head: Ferrugineous, frontally narrowly slightly paler. At eyes with irregular, fine punctures. Submat, densely microsculptured. Reticulation indistinctly double. Coarser meshes in part strongly obliterated; indistinct. Coarse meshes, when discernible, contain 2–6 fine meshes.

Pronotum: At base and anteriorly darkened, blackish to dark ferrugineous; laterally and towards frontal corners pronotum paler; pale ferrugineous. Change of colour sometimes gradual. Pronotum discally impunctate. At margins with very fine, hardly discernible punctures. Submat, finely and densely microsculptured. Reticulation double. Large meshes distinct; contain 2–6 fine meshes.

Elytra: Extensively black to blackish ferrugineous, with somewhat variable pale ferrugineous to ferrugineous markings (Figs 483–484). Discally, dorsolaterally and laterally with scattered very fine punctures (not forming distinct rows). Submat, distinctly microsculptured. Reticulation double but large meshes fine, in part (laterally and posteriorly) hardly discernible or absent.

Ventral aspect: Black to dark ferrugineous, prothorax pale, ferrugineous to pale ferrugineous. Almost impunctate. Rather shiny, extensively with very fine, in part indistinct, microsculpture. Metacoxal plates with about 10 indistinct and shallow furrows, most of which are transversely located. Abdomen in basal half with curved striae. Apical ventrite asymmetric, with one, distinct, lateral knob (Fig. 154). Prosternal process slender, apex extended and pointed.

Legs: Pro- and mesotarsus rather long and slender. Tarsi provided with suckers.

Male genitalia: Penis delicate, in lateral aspect slightly curved; apically penis ends in a small extension (Figs 337–338).

Female: Apical ventrite lacks knob; as in Fig. 155. Pro- and mesotarsus slender.

##### Distribution.

Cameroon, Zaire (Fig. 567). Medler (1980) gives Nigeria.

##### Collecting circumstances.

Unknown.

#### Laccophilus brownei

Taxon classificationAnimaliaColeopteraDytiscidae

Guignot, 1947

Figs 156–157 , 339–340 , 485–486 , 567

Laccophilus brownei Guignot 1947: 26 (original description, faunistics); Guignot 1948: 13 (description, faunistics); Guignot 1953e: 4 (discussion); Guignot 1954a: 28 (discussion); Omer-Cooper 1957: 12 (discussion); Guignot 1959a: 552, 553, 554 (description, faunistics); Nilsson 2001: 241 (catalogue, faunistics); Nilsson 2015: 209 (catalogue, faunistics).
Laccophilus brownei ab. celidotus Guignot 1947: 26 (description, faunistics); Guignot 1948: 14 (description, faunistics); Guignot 1959a: 553, 554 (description, faunistics); Nilsson 2001: 281 (infrasubspecific name, not valid taxon).

##### Type locality.

Zaire: PNA, Lac Magera.

##### Type material studied

(5 exs.). Holotype: male: “Holotypus / Congo belge: P.N.A. Lac Magera 2000 m, 27-VIII-1935 Mission H. Damas: (A) 370 / Coll. Mus. Congo (ex. coll. I.P.N.C.B.) / Type / F. Guignot det. 1945 *Laccophilus brownei* Guign. Type, male” (MRAC). – Paratypes (incl. Allotype): Same sampling data as holotype (1 ex. IRSNB, 1 ex. MRAC) “Paratypus / Congo belge P.N.A. Ngesho 3-VIII-1935 Mission H. Damas: 291 / F. Guignot det. 1948 *Laccophilus brownei* Guign.” (2 exs. MRAC; habitus in Fig. 485).

##### Additional material studied

(15 exs.). **Zaire**: “Congo belge P.N.A. Ngesho 3-VIII-1935 Mission H. Damas: 291 / F. Guignot det. 1948 Laccophilus brownei ab. celidotus sp. n. / Holotype / Paratype” (3 exs. IRSNB, 1 ex. MNHN, 4 exs. MRAC; habitus in Fig. 486); “Kivu Kagogo 2-9.6. 1952 Damas” (1 ex. MRAC). – **Tanzania**: “Tanganjika PWD Camp Tundume Mbeya rd 14.X. 1948 JOC.” (1 ex. AMGS); “Tanganyika creek Chunya-Mbeya road 11.10. 1948” (5 exs. AMGS).

##### Diagnosis.

*Laccophilus brownei* is especially characterized by shape of penis; apical half of penis almost straight and evenly broad to distinct apex. Apical part is merged to basal part so that no contraction visible but apex separated from basal part by narrow incision. Dorsal colour pattern resembles corresponding features in some other species located in this species group (e.g. *Laccophilus incrassatus*, *Laccophilus quindecimvittatus* and *Laccophilus empheres*).

##### Description.

Body length 4.2–4.5 mm, width 2.4–2.6 mm. Habitus (Fig. 485). Longitudinal dark lines medially independent, not confluent. Sometimes dark markings of head and pronotum slightly more enlarged; dark elytral lines medially confluent (celidotus-aberration) (Fig. 486).

Head: Pale ferrugineous, posteriorly at pronotum narrowly darkened. Slightly mat, distinctly reticulated. Reticulation in part double, which is clearly discernible in a medial area where large meshes contain 2–5 smaller meshes. At eyes with irregular, quite dense punctures.

Pronotum: Pale ferrugineous; anteriorly at head with a vague, dark ferrugineous marking. Posteriorly, at scutellar region with a broad but narrow, distinct, dark ferrugineous marking. Slightly mat, densely reticulated. Reticulation extensively double; large meshes contain 2–7 small meshes. At margins with scattered, irregular puntures (hardly visible at scutellar region).

Elytra: Pale ferrugineous, with dark ferrugineous to dark brownish, longitudinal lines. Lines variable; medially independent, not confluent or confluent, especially in centre of elytra (Figs 485–486). Submat, distinctly reticulated. Reticulation simple. Sometimes, indistinct fragments of larger meshes may be discerned. Impunctate, except for three, irregular and vague rows of fine punctures.

Ventral aspect: Metathorax and abdomen pale ferrugineous to ferrugineous; metacoxal plates blackish to dark ferrugineous. Impunctate. Very finely microsculptured. Metacoxal plates with shallow, transverse furrows. Prosternal process sharp, narrow. Apical ventrite asymmetric, with a lateral knob (Fig. 156). Abdomen with slightly curved striae.

Legs: Pale ferrugineous to ferrugineous. Pro- and mesotarsus somewhat enlarged, provided with distinct suckers.

Male genitalia: Penis long, slightly curved at base and almost straight and evenly broad in apical half. Extreme apex broad and moderately extended (Figs 339–340).

Female: Apical ventrite lacks knob (Fig. 157). Pro- and mesotarsus slender.

##### Distribution.

Zaire, Tanzania (Fig. 567).

##### Collecting circumstances.

Not known.

#### Laccophilus contiro

Taxon classificationAnimaliaColeopteraDytiscidae

Guignot, 1952

Figs 158–159 , 341–342 , 487–488 , 570

Laccophilus contiro Guignot 1952d: 5 (original description, faunistics); Guignot 1953b: 234 (faunistics); Omer-Cooper 1957: 10 (discussion, description); Omer-Cooper 1958b: 37, 38, 39, 40 (description, faunistics, biology); Guignot 1959a: 550, 554 (description, discussion, faunistics); Guignot 1959d: 161 (discussion); Guignot 1961b: 238 (discussion); Omer-Cooper 1962: 295 (faunistics); Omer-Cooper 1965: 76, 77, 78 (description, discussion, faunistics); Bilardo and Pederzani 1978: 119 (faunistics, description); Medler 1980: 155 (faunistics, list); Bilardo 1982a: 447 (given as *Laccophylus*, description, faunistics); 1982b: 251 (faunistics); Pederzani and Rocchi 1982: 72 (faunistics); Pederzani 1988: 107 (faunistics, biology); Nilsson and Persson 1993: 80: (faunistics); Bilardo and Rocchi 1999: 232, 234 (faunistics); Nilsson 2001: 242 (catalogue, faunistics); Bilardo and Rocchi 2002: 174 (list, faunistics); Nilsson 2015: 210 (catalogue, faunistics). [Comment: in the original description Guignot (1952d: 6) also distinguishes a separate morph and gives it the name Laccophilus contiro ab. nigrovirgatus. This name is, however, infrasubspecific and has no value in nomenclature.]

##### Type locality.

Ethiopia: Mt Chilalu.

##### Type material studied

(4 exs.). Holotype: male: “Abessinia 7,000 ft. Mt. Chilalu 8.xi. 1926 J. Omer-Cooper / male symbol / Type” (MNHN). – Paratypes: “Abyssinia Mount Chillálo ponds 7,000-8,000 ft. 8-9.xi. 1926 J. Omer-Cooper / female symbol / Allotype / *contiro”* (1 ex. MNHN); “Abyssinia 7000 ft. Mt Chilalu 8.xi. 1926 J. Omer-Cooper / male symbol / Paratype” (1 ex. MNHN; habitus in Fig. 487). – Uganda: “Uganda Kampala K 11? IX. 1929 H. E. Hopkins / female symbol / paratype” (1 ex. MNHN; habitus in Fig. 488).

##### Additional material studied

(162 exs.). **Sudan**: “W. Nile IX. 1929” (2 exs. AMGS); “Gallery Forest Jebel Marra 12°55’N, 24°08’E, 7. 1984 Ruse P. light trap” (8 exs. BMNH, 1 ex. MZH). – **Ethiopia**: “Suc-Suci Lake Zwai 5,500 ft 12.11. 1926 JOC.” (1 ex. AMGS); “7000 ft Mt. Chilalu 8.11. 1926 JOC.” (5 exs. AMGS); “West Marsh Lake Zwai 5,500 ft 2-3.11. 1926 JOC.” (3 exs. AMGS); “March N of Lake Zwai ca 5,500 ft. 4.11. 1926” (1 ex. AMGS); “Kattere River Lake Zwai 6,000 ft 5.11. 1926 JOC.” (1 ex. AMGS); “Water Hole N of Makki River 6,000 ft 28.11. 1926 JOC.” (1 ex. AMGS); “C Abyssinia, Abesata Wajju, Bull Bullo 6.3. 1915 Lovacs leg.” (1 ex. BMNH); “Belleta Forest 13-14.6. 1963 Linnavuori” (1 ex. MZH). – **Sierra Leone**: “Freetown 1945/Walton G.A.” (1 ex. BMNH). – **Nigeria**: “NC St. Malumfashi 26-30.7. 1973 Linnavuori leg.”(2 exs. MZH); “W St. Ife 7-8.7. 1973 Linnavuori leg.” (1 ex. MZH); “Stream crossing Kaduna rd. nr Zaria 4.4. 1963 JOC.” (2 exs. AMGS); “Zaria 1969 Brancucci” (1 ex. NHMB); “Stream & reservoir Jos 10.4. 1963 JOC.” (3 exs. AMGS); “Stream nr Bakori en rte Katsina 5.4.1963 JOC.” (1 ex. AMGS); “Stream nr Zaria 4.4. 1963 JOC” (1 ex. AMGS); “Ponds in dry stream bed Kontagora 5.4. 1963” (2 exs. AMGS); “Kontagora stream 3.4. 1963 JOC.” (6 exs. AMGS). – **Cameroon**: “20 km NW Ban-Gante Forest, savannah at river, at light 15.1. 1978 / Gärdenfors, Hall & Samuelsson” (1 ex. MZLU); “Kamerun int. Satsche 10-14.5. 1909 Riggenbach” (5 exs. ZMHB); “Koza 1.7.1974”(1 ex. NHMB). – **Uganda**: “Prov. d’Ounyoro, Albert Nyanza S-E, Riv. Mousisi, Alluaud 1909 / Type” (1 ex. MNHN; ”type” of ab. nigrovirgatus); “Kampala 13.7. 1929 G.H.E. Hopkins” (1 ex. AMGS); same but “15.9. 1929” (1 ex. AMGS); same but “28.2. 1929” (1 ex. AMGS); same but 28.11. 1929” (1 ex. BMNH); same but “29.11. 1929” (1 ex. BMNH); same but “21.2. 1930” (1 ex. BMNH); same but “5.12. 1929” (1 ex. MZH); same but “21.8. 1929” (1 ex. BMNH); same but 9. 1929/Paratype” (1 ex. MNHN; not type material); same but “Kitante Swamp 26.9. 1969” (1 ex. BMNH); “Madi 5. 1927 G.D.H. Carpenter” (1 ex. AMS). – **Kenya**: “Nyeri 2.12. 1989 Jäch” (1 ex. NMW); “Ngong Forestry Sta., 13.4, 1968 Spangler” (7 exs. USNM, 2 exs. MZH); “Nairobi 3.11. 1967 / Reichert collector” (1 ex. USNM). – **Tanzania**: “?stream Mbeya-Tunduma rd. 18.10. 1948 JOC.” (3 exs. AMGS); “Mpemba stream Mbeya-Tunduma 16.X. 1948” (2 exs. AMGS); “D.O.A. W Ruangwa 1.9. 1911, 1850 m Sumpf H. Meyer S.G.” (2 exs. ZMHB). – **Zambia**: “Mbesuma Ranch (Isoka) 9-10.12. 2004 Werner & Smrz” (2 exs. NHRS); “Watergreen Farm Chongwe Valley 60 km E Lusaka 4.8. 1986 Pederzani / *Laccophilus contiro* Guignot det. Pederzani” (1 ex. CSR); “Kapiri Mpushi env. 13.12. 2002 Kantner” (6 exs. NHMB, 2 exs. MZH). – **Malawi**: “Swamp Dally’s Hotel nr Ft Johnstone 23.8. 1948” (6 exs. AMGS); “Swamp Hawkes Bay 25.9. 1948” (2 exs. AMGS); “Dambo below Livingstonia lake shore 21.10. 1948” (15 exs. AMGS); “Dally’s 18.12. 1946 R.H. Lowe” (2 exs. BMNH); “Ft. Johnston Dally’s swamp nr L. Nyasa 7.6. 1946” (1 ex. BMNH); “Selima env., 4.1. 2002 Kantner” (1 ex. NHMB); “Selima env., 60 km E Lilongwe 5-8.1. 2002 Kantner” (1 ex. NHMB); “Dedza env., 85 km SE Lilongwe 7-13.1. 2002 Kantner” (1 ex. NHMB). – **Namibia**: “East Caprivi Katima Mulilo 17°29'S-24°17'E, 3-8.3. 1992 Uhlig leg., lux” (8 exs. ZMHB, 2 exs. MZH, 1 ex. NMNW); same but “Deckert leg.” (1 ex. ZMHB). – **Zimbabwe**: “Mashunald Salisbury” (1 ex. SAMC). – **South Africa**: “Gauteng, RAU system, Germiston Lake 12.8. 1997 Reavell” (1 ex. AMGS); “Natal Umlazi R. 19.9. 1962” (1 ex. AMGS); “Kw. Natal S, Port Shepstone 20 km W, 2.2. 2000 Halada leg.” (1 ex. NMW); “Kw. Natal, Barringtonia Swamp For., Amotikela Nat. Res., 24.2. 1991 Reavell” (1 ex. AMGS); “Natal, Durban, Stamford Hill Umgeni Trägårdh leg. / *Laccophilus contiro* Guign. det. Omer-Cooper” (1 ex. MZLU); “ECPR. St. Johns 16.2. 1956 JOC. / *Laccophilus contiro* Guign. det. Omer-Cooper” (6 exs. AMGS); “ECPr. 6 km S of port of St Johns, outside Silaka Nature Res., pond, S31°38.862, E29°30.551, alt. 90 m 26-27.1. 2005 Bergsten leg.” (4 exs. NHRS); “EC, East London, Gorncie Park, coastal pond 18.3. 1955” (2 exs. AMGS); “EC., Pond on Hwy 344 at Adelaide 17.5. 2005 Challet” (3 exs. CGC, 1 ex. MZH); “EC, Groot R., Humansdorp 19.2. 1947 JOC.” (11 exs. AMGS).

##### Diagnosis.

Dorsal colour pattern of *Laccophilus contiro* is variable. Extremes are represented by specimens with almost totally confluent dark, longitudinal lines of elytra (dark lines only discernible posterior to middle) or specimens with separate dark, longitudinal lines. Fortunately penis is quite characteristic, the apex being “harpoon-shaped”, and this character distinguishes *Laccophilus contiro* from all other *Laccophilus* species. Morphological variation is still quite extensive and further study will reveal if *Laccophilus contiro* must be split up in different species.

##### Description.

Body length 3.5–3.9 mm, width 1.9–2.1 mm. Elytral colour pattern variable; separate, dark, longitudinal areas almost absent because merged to larger dark areas, or elytral colour pattern consists of distinct longitudinal, dark areas, which sometimes are reduced (Figs 487–488).

Head: Pale ferrugineous to ferrugineous; no distinct colour pattern. Impunctate, except at eyes; with fine, dense and irregular punctures. Area of punctures extended towards middle of head-disc but they are not connected. Submat, finely microsculptured. Reticulation double but difference in delimitation of size classes very small; in part hardly discernible. Large meshes, when discernible, contain 2–5 fine meshes.

Pronotum: Pale ferrugineous to ferrugineous, frontally and basally in middle with a distinct blackish to dark ferrugineous area. Submat, finely microsculptured. Reticulation quite distinctly double; large meshes contain generally 2–5 small meshes. Almost impunctate. At margins, except basally in middle, with fine, sparse and scattered punctures.

Elytra: Colour pattern variable. Dark elytral lines, to a variable degree discernible; sometimes distinct and sometimes almost absent (Figs 487–488). Submat, finely microsculptured. Reticulation indistinctly double. Large meshes almost absent because strongly reduced (delimiting lines of meshes have often vanished); sometimes indistinctly visible as fine fragments in frontal parts of elytra. Almost impunctate; very fine, sparse punctures form a discal, dorsolateral and lateral row of punctures.

Ventral aspect: Dark ferrugineous to ferrugineous, prosternum paler, pale ferrugineous. Almost impunctate. Rather shiny; scattered very fine, in part indistinct microsculpture may be discerned. Metacoxal plates with approximately 10 shallow and transversely placed furrows. Base of abdomen with curved striae. Prosternal process rather slender, apex somewhat extended and pointed. Apical ventrite with a lateral, sharp knob (Fig. 158).

Legs: Pro- and mesotarsus somewhat extended, provided with distinct suckers.

Male genitalia: Penis in lateral aspect quite broad; from base to apex slightly curved. Extreme apex broad, only slightly extended (Figs 341–342).

Female: Pro- and mesotarsus slender, somewhat extended. Apical ventrite lacks knob (Fig. 159).

##### Distribution.

Sudan, Ethiopia, Sierra Leone, Nigeria, Cameroon, Uganda, Kenya, Tanzania, Zambia, Malawi, Namibia, Zimbabwe, South Africa (Fig. 570). Additional country records are Zaire, Rwanda-Burundi (Omer-Cooper 1965), the Ivory Coast (Bilardo and Pederzani 1978), Congo (Brazzaville) (Pederzani and Rocchi 1982) and Gabon (Bilardo and Rocchi 1999).

##### Collecting circumstances.

Label data gives limited information on ecological preferences; collected in pools, streams and swamps and sometimes at light. Literature data are considered rather poor.

#### Laccophilus inconstans sp. n.

Taxon classificationAnimaliaColeopteraDytiscidae

http://zoobank.org/63563001-1560-41E1-B296-DAA1E05118BF

**Figures 160–172.**
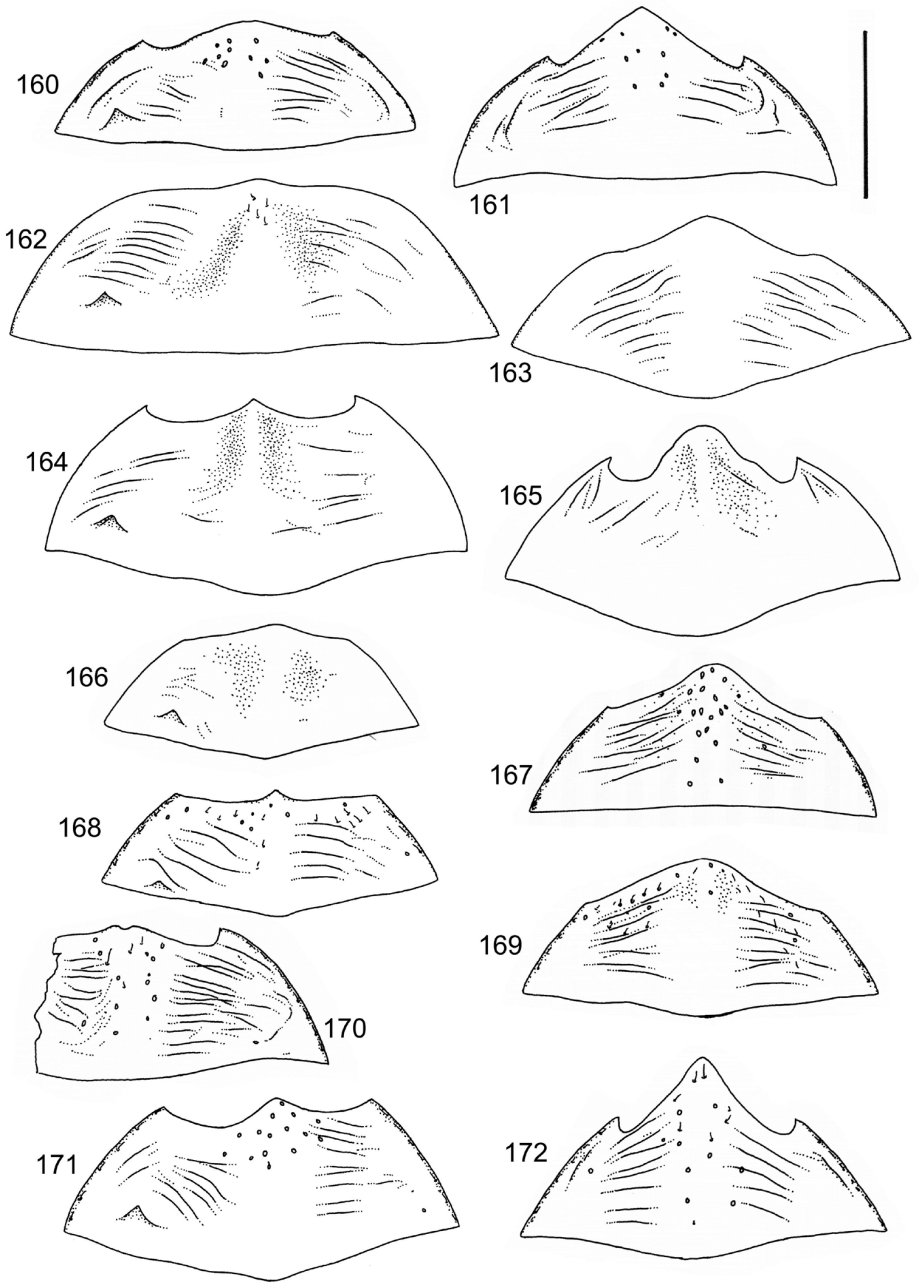
Apical ventrites **160** *Laccophilus inconstans* male, and **161** female **162** *Laccophilus grammicus* male, and **163** female **164** *Laccophilus flavoscriptus* male, and **165** female **166** *Laccophilus burgeoni* male, and **167** female **168** *Laccophilus lineatus* male, and **169** female **170** *Laccophilus brancuccii* male (broken) **171** *Laccophilus incomptus* male, and **172** female. Scale bar 0.5 mm.

Figs 160–161 , 343 , 489–490 , 566

##### Type locality.

The Ivory Coast: Man.

##### Type material

(63 exs.): Holotype: male: “Ivory Coast Man 14–21.10. 1973 R. Linnavuori” (MZH; habitus in Fig. 490). – Paratypes: Same data as holotype (1 ex. MZH); “Coll. Mus. Tervuren Cote d’Ivoire: Bingerville VI. 1962 J. Decelle” (1 ex. MRAC, 1 ex. MZH); same and “à la lampe U.V.” (1 ex. MRAC); “same but “VIII. 1962” (2 exs. MRAC); “Bouakè 12.8. 1973 Bilardo & Pederzani” (1 ex. NHMB); “Toumodi 11.8. 1973 Bilardo & Pederzani” (1 ex. NHMB); “Cote d’Ivoire Duékoué / 12. 1930-IV. 1931 Alluaud & Chappuis” (1 ex. MNHN). – Guinea: “Rep. Guinea Seredoux, lux, 7-8.4. 1975 leg. Zott” (1 ex. ZMHB); same data but “18.4. 1975” (1 ex. ZMHB); same data but “4.4. 1975” (1 ex. ZMHB, 1 ex. MZH). – Ghana: “Eastern Region Boti Falls 30 km NE Koforidua Dr. S. Endrödy-Younga / Nr. 490 shore washing 27.XII. 1971” (1 ex. CGW); “Ashanti Region Kwadaso 259 m, N 6.55-W 1.39 Dr. S. Endrödy-Younga / Nr. 367 – light trap on field 2.VI. 1969” (1 ex. CGW); “Ashanti Region Kumasi, Nhiasu 330 m, N 6.43-W 1.36, Dr. S. Endrödy-Younga / Nr. 229 light trap 24.VI. 1967” (2 exs. CGW, 1 ex. MZH); same data but “Nr. 256 at light 10.VIII. 1967” (1 ex. CGW); same data but “Nr. 274 at light 15.IX. 1967” (1 ex. CGW); same data but “Nr. 280 at light 9.X. 1967” (1 ex. MZH); same data but “Nr. 281 at light 18.X. 1967” (4 exs. CGW); same data but “Nr. 282 at light 20.X. 1967” (1 ex. CGW); “Kumasi 24.6. 1967 S. Endrödy-Younga” (2 exs. CGW, 1 ex. MZH, 1 ex. HNHM); same but “18.5. 1967” (3 exs. CGW, 2 exs. HNHM); same but “20.5. 1967” (3 exs. CGW, 3 exs. HNHM); same but 12.6. 1967 (2 exs. HNHM); same but 16.4. 1967 (2 exs. HNHM). – Nigeria: “Nigeria W. St. Ife 7-8.7. 1973 R. Linnavuori” (3 exs. MZH); “Ile-Ife W. State 13. Aug. 1972 J.T. Medler Coll.” (2 exs. USNM); “Lagos Colony Iseri 29-30.3. 1949 Malkin / meander pool in shallow water” (2 exs. CGW); same data but “26-27.3. 1949 / Stream, deep slimy mud with sand over” (1 ex. CGW). – Cameroon: “Cameroun Buea-Nord-est Malende 17-1-79 A. Bilardo / *Laccophilus* sp. (?) near *contiro* det. S. Rocchi 1985” (1 ex. CSR; habitus in Fig. 489); “Cameroon, 25 km WNW Douala Modeka, Secondary Forest and Plantation, at light 18.1. 1978 Loc. No. 27/Lund Univ. Syst. Dept. Sweden Cameroon Exp. Dec.-Jan. 1977-78 Gärdenfors-Hall- Samuelsson / *Laccophilus secundus* Régimbart Det. A. Nilsson -96” (5 exs. MZLU, 2 exs. MZH).

##### Diagnosis.

*Laccophilus inconstans* is especially characterized by small body, by extensively black coloured elytra with minor and somewhat variable, pale ferrugineous markings and peculiar penis. *Laccophilus inconstans* resembles quite much *Laccophilus conjunctus*, from which it can be distinguished by slightly smaller body and by examination of penis; in lateral aspect penis curved, quite broad and extreme apex extends to an almost square-like process (corresponding apical process in *Laccophilus conjunctus* is distinctly smaller).

##### Description.

Body length 3.1–3.2 mm, width 1.7–1.8 mm. Elytral colour pattern variable; sometimes with quite extensive basal pale marking; sometimes pale markings strongly reduced (Figs 489–490).

Head: Pale ferrugineous, head sometimes a little darker posteriorly. Impunctate, except at eyes with fine, irregular punctures. Slightly mat, finely microsculptured. Reticulation double but size-classes of meshes difficult to separate because difference between them is often minimal. When discernible, large meshes may contain 3–6 small meshes.

Pronotum: Pale ferrugineous. Basally in middle with distinct, blackish to dark ferrugineous spot. Anteriorly, with a somewhat vague, dark ferrugineous to ferrugineous spot. Impunctate, except anteriorly, where very fine, irregular punctures may be discerned. Slightly mat, microsculptured; reticulation double. Large meshes generally discernible; may contain 3–6 fine meshes.

Elytra: Black to dark ferrugineous, with variable pale ferrugineous markings (Figs 489–490). Almost impunctate. Only a discal row of fine and irregular punctures clearly discernible. Pre-apical, lateral row of punctures form a shallow furrow which is finely pubescent. Slightly mat, finely microsculptured. Delimiting lines of large meshes laterally and posteriorly in part reduced, indistinct.

Ventral aspect: Dark ferrugineous to ferrugineous; no distinct colour pattern. Prothorax pale ferrugineous. Almost impunctate. Shiny, indistinctly microsculptured; in part microsculpture lacking. Basal ventrites with fine, slightly curved striae. Metacoxal plates with some ten shallow, in part indistinct furrows, which are mostly transversely located. Apical ventrite asymmetric; on one side with a small knob (Fig. 160). Prosternal process slender, apically extended and pointed.

Legs: Pale ferrugineous. Pro- and mesotarsus slightly enlarged, with suckers.

Male genitalia: Penis slightly curved, quite broad and ends in a distinct, square-like process (Fig. 343).

Female: Pro- and mesotarsus slender. Apical ventrite simple, no lateral knob (Fig. 161).

##### Etymology.

The name *inconstans* is a Latin adjective that here refers to the highly variable appearance of the elytra.

##### Distribution.

Guinea, Ivory Coast, Ghana, Nigeria, Cameroon (Fig. 566).

##### Collecting circumstances.

In Cameroon collected with light in secondary forest and plantation. From Nigeria label data give that the species has been collected in a meander pool in shallow water and in a stream.

#### Laccophilus grammicus

Taxon classificationAnimaliaColeopteraDytiscidae

Sharp, 1882

Figs 162–163 , 344–346 , 491 , 565

Laccophilus grammicus Sharp 1882: 306, 307 (original description, faunistics, discussion); v. d. Branden 1885: 21 (catalogue, faunistics); Régimbart 1895: 141 (description, faunistics); Régimbart 1905: 208 (faunistics); Régimbart 1906: 249 (faunistics); Régimbart 1908a: 5 (faunistics); Zimmermann 1920a: 19 (catalogue, faunistics); Régimbart 1922: 532 (faunistics); Omer-Cooper 1931: 758 (description, discussion, biology, faunistics); Gschwendtner 1938a: (faunistics, discussion); Guignot 1946c: 263, 264, 265, 279, 312 (description, faunistics, discussion); Legros 1954: 268 (discussion); Guignot 1959a: 544, 548, 549, 550, 552 (description, faunistics, discussion); Omer-Cooper 1970: 286, 287 (description, discussion); Nilsson and Persson 1993: 79, 94 (faunistics, discussion, biology); Nilsson 2001: 244 (catalogue, faunistics); Nilsson 2015: 212 (catalogue, faunistics). [Comments: information on *Laccophilus grammicus* outside Ethiopia and Eritrea is considered uncertain.]

##### Type locality.

Ethiopia: Abyssinia.

##### Type material studied

(3 exs.). Lectotype (by present desgination): male: “Type 569 / Type / Abyssinia / Sharp Coll. 1905-313 / *Laccophilus grammicus* sp. n. Abyssinia” (BMNH). – Paralecotytpes: “569 / Co-type / Abyssinia/Sharp Coll. 1905-313 / *Laccophilus grammicus* Shp Co-type”(1 female ex. BMNH); “Co-type / Abyss. Raffray / Abyssinia A. Raffray / *Laccophilus grammicus* Shp Co-type”(1 male ex. BMNH).

##### Additional material studied

(16 exs.). **Ethiopia**: “Stream W of Zaguala 6000 ft. 27.10. 1926 JOC.” (5 exs. AMGS); “Abyss. Raffr. / *Laccophilus grammicus* Shp det. M. Brancucci” (3 exs. NHMB); “Tigray Province ca. 20 km E Axum 2000 m 17.4. 2006 leg. Wewalka” (4 exs. CGW; habitus in Fig. 491). – **Eritrea**: “Adi-Ugri 5. 1901 Andreini / *Laccophilus grammicus* Sharp det. Rocchi 1995” (2 exs. CSR); “Asmara-Decamere 25.5. 1963 Linnavuori” (1 ex. MZH); “Ghinda Levander” (6 exs. MZH).

##### Diagnosis.

*Laccophilus grammicus* resembles a number of species in the same species group and can therefore be difficult to identify solely using external features as appearance of body colour pattern. The species is, however, easily separated by examination of male genitalia; penis in apical half tapers gradually towards apex; in lateral aspect, penis very strongly curved.

##### Description.

Body length 4.1–4.3 mm, width 2.2–2.4 mm. Dorsal, colour pattern of body distinct and quite uniform (Fig. 491).

Head: Pale ferrugineous. Slightly mat, finely microsculptured. Reticulation double: number of fine meshes in one large mesh varies from 2–5. In part, double reticulation weakly developed and rather indistinct: fine and large meshes difficult to distinguish. At eyes and medially from eyes towards middle of head finely and sparsely punctured. Head extensively impunctate.

Pronotum: Pale ferrugineous; mediobasally with narrow blackish ferrugineous marking. Submat, finely microsculptured; reticulation double: large meshes contain 2–6 fine meshes. At margins with irregular, sparse row of fine punctures. At base, row of punctures in part absent.

Elytra: Pale ferrugineous, with distinct blackish to dark ferrugineous, longitudinal markings, which are in part reduced basally, slightly posterior to middle and apically (Fig. 491). Submat, finely microsculptured. Reticulation predominantly simple; large meshes discernible but vague and weakly developed. Three rather indistinct rows of sparse punctures are discernible.

Ventral aspect: Pale ferrugineous to dark ferrugineous. Almost impunctate. Very finely microsculptured; in part fine reticulation indistinct or absent. Metacoxal plates in anterior half with shallow, transversely located, in part rather indistinct furrows. Abdomen with fine, curved striae. Apical ventrite with sharp knob on one side (Fig. 162). Prosternal process slender, pointed.

Legs: Pro- and mesotarsus quite long, slightly enlarged, provided with suckers.

Male genitalia: Penis in apical half tapers rather evenly and gradually towards apex; in lateral aspect, penis very strongly curved (Figs 344–346).

Female: Apical ventrite lacks sharp knob (Fig. 163). Pro- and mesotarsus slender.

##### Distribution.

Ethiopia, Eritrea (Fig. 565). Records outside Ethiopia and Eritrea are considered doubtful.

##### Collecting circumstances.

Collected at high altitudes (Omer-Cooper 1931, Nilsson and Persson 1993).

#### Laccophilus flavoscriptus

Taxon classificationAnimaliaColeopteraDytiscidae

Régimbart, 1895

Figs 164–165 , 344–346 , 492–494 , 567

Laccophilus flavoscriptus Severin 1892: 472 (discussion); Régimbart 1895: 142 (original description, faunistics); Zimmermann 1920a: 18 (catalogue, faunistics); Gschwendtner 1930: 90 (faunistics); Gschwendtner 1931: 180 (faunistics); Gschwendtner 1938a: 5 (faunistics); Omer-Cooper 1956: 23 (discussion, faunistics); Guignot 1959a: 533, 537 (given as *Laccophilus flavopictus*), 540 (description, faunistics); Pederzani and Rocchi 1982: 79 (discussion); Nilsson 2001: 243 (catalogue, faunistics); Bilardo and Rocchi 2002: 174 (list, faunistics); Nilsson 2015: 211 (catalogue, faunistics).
Laccophilus flavosignatus Régimbart 1895: 146 (original description, faunistics); Zimmermann 1920a: 18 (catalogue, faunistics); Omer-Cooper 1931: 759 (description, biology, faunistics); Gschwendtner 1932b: 260 (faunistics); Guignot 1946c: 262, 266 (discussion); Guignot 1950b: 272 (discussion); Guignot 1959a: 540, 578 (description, discussion, faunistics, synonymy, Laccophilus flavoscriptus ab. flavosignatus); Guignot 1961b: 238 (faunistics, discussion); Nilsson 2001: 243 (catalogue, faunistics, list, synonymy); Nilsson 2015: 211 (catalogue, faunistics, list, synonymy). **Confirmed synonym.**

##### Type localities.

*Laccophilus flavoscriptus*: Zaire: Matadi.

*Laccophilus flavosignatus*: Gabon.

##### Type material studied

(29 exs.). *Laccophilus flavoscriptus*: Lectotype (by present designation): male: “Congo belge Matadi / male symbol / Cotype / Museum Paris coll. Maurice Régimbart 1908 / *flavoscriptus* Rég.” (MNHN; habitus in Fig. 493). – Paralectotypes: “Banana Boma M. Tschoffen 91 Dét / Museum Paris coll. Maurice Régimbart 1908” (1 ex. MNHN); “Banana Boma M. Tschoffen 91 Det. Régimb. 91 / 11165 / Ex Type / Régimbart det. 1895 *Laccophilus flavoscriptus* Rég.” (6 exs. IRSNB, 1 ex. BMNH; habitus in Fig. 492); same but labelled with additional “Cotype” (1 ex. IRSNB); “Boma Tschoffen / Ex Type / Régimbart det. 1895 *Laccophilus flavoscriptus* Rég.” (1 ex. IRSNB); “Severin Banana Africa / Banana Boma M. Tschoffen 91 Régimb. / Type” (1 ex. RMNH); “Severin Banana Afr. Occ. / *Laccophilus flavoscriptus* Dét. Régimb. Type / Banana Boma M. Tschoffen 91 Dét. Régimb. / Type” (1 ex. RMNH); “Banana Boma M. Tschoffen 91 / *Laccophilus flavoscriptus* Régb. Det. Régimbart” (1 ex. SAMC); “Matadi Congo / Museum Paris coll. Maurice Régimbart 1908” (4 exs. MNHN); “Matadi M. Tschoffen / Museum Paris coll. Maurice Régimbart 1908 / *flavoscriptus* Rég.” (2 exs. MNHN); same data but “SAM Type Acc. No. 840” (3 exs. SAMC).

*Laccophilus flavosignatus*: Lectotype (by present designation): male: “Gabon Mocquerys /male symbol / Cotype” (MNHN; habitus in Fig. 494). – Paralectotypes: “Gabon / Museum Paris coll. Maurice Régimbart 1908 / *flavosignatus* Rég.” (3 exs. MNHN); “Gabon Mocquerys / Museum Paris coll. Maurice Régimbart 1908 / *flavosignatus* R. Paulian det.”(2 exs. MNHN).

##### Additional material studied

(1 ex.): **Gabon**: “Ogové River leg. A. C. Good” (1 ex. CSR).

**Comment on synonymy**: Study of type material of the two involved taxa and designation of lectotypes for them proved us that that earlier established synonymy was correct.

##### Diagnosis.

*Laccophilus flavoscriptus* externally resembles a number of species as *Laccophilus necopinus*, *Laccophilus conjunctus*, *Laccophilus adjutor* and *Laccophilus lineatus*, on the basis of dorsal colour pattern of body. Elytral colour pattern is variable in this group of resembling species; variation appears from almost entirely dark to pale with rather distinct, dark, longitudinal markings. For identification of *Laccophilus flavoscriptus*, fortunately, the penis is very characteristic; apical half of penis is evenly broad and evenly curved towards abrupt apex of penis.

##### Description.

Body length 3.5–3.8 mm, width 1.9–2.1 mm. Body with variable dorsal colour pattern (see below).

Head: Pale ferrugineous, close to pronotum often slightly darker. Submat, finely, microsculptured; reticulation double but large meshes only slightly more strongly developed than small meshes. Sometimes large meshes strongly reduced and only discernible as fragments of meshes. Almost impunctate; at eyes finer punctures may be discerned.

Pronotum: Pale ferrugineous with mediofrontal and -basal dark ferrugineous to ferrugineous areas, delimitation of which are vague. Sometimes dark markings almost black to blackish ferrugineous. Submat, reticulation double. Meshes of large reticulation fine; in general one mesh contains 3–5 small meshes. Almost impunctate. Very fine, sparse punctures may be discerned frontally and laterally.

Elytra: Pale ferrugineous, with variable dark ferrugineous to blackish colour pattern; elytra sometimes almost entirely dark, sometimes with more or less distinct, longitudinal, pale lines and a basal, pale, transverse area (Figs 492–494). Submat, finely reticulated. Reticulation double; large meshes often somewhat indistinct. Almost impunctate. Very fine scattered punctures form a discal row. Dorsolateral and lateral rows indicated by a few scattered punctures. Lateral, pre-apical furrow fine, moderately pubescent.

Ventral aspect: Blackish to dark ferrugineous, prothorax pale ferrugineous. Rather shiny to slightly mat, very finely microsculptured. Basal ventrites with fine, curved striae. Almost impunctate. Apex of prosternal process somewhat extended, slender, pointed. Metacoxal plates in anterior half with very fine, transversely located, shallow furrows; in posterior half furrows almost absent. Apical ventrite asymmetric; on one side with a subbasal minute tubercle (Fig. 164).

Legs: Protarsus slender, claws moderately curved. Pro- and mesotarsus with suckers.

Male genitalia: Penis in lateral aspect, long, slightly curved and almost evenly broad from base to apex; extreme apex almost unmodified (Figs 344–346).

Female: Apical ventrite (Fig. 165). Pro- and mesotarsus slender.

##### Distribution.

Gabon, Zaire (Fig. 567). Additional country records are Ethiopia (Omer-Cooper 1931) and Cameroon (Guignot 1961b) but we have not verified these determinations. A record from Senegal is considered uncertain because based on a single female specimen (Guignot 1961b).

##### Collecting circumstances.

Almost unknown. In Ethiopia collected at high altitude (ca. 5500 ft. = ca. 1672 m) (Omer-Cooper 1931).

#### Laccophilus burgeoni

Taxon classificationAnimaliaColeopteraDytiscidae

Gschwendtner, 1930

Figs 166–167 , 348–349 , 495–496 , 565

Laccophilus burgeoni Gschwendtner 1930: 89 (original description, faunistics); Guignot 1946c: 282 (description, faunistics); Guignot 1953e: 4 (discussion); Guignot 1954: 25 (discussion); Guignot 1959a: 557, 563 (description, faunistics); Nilsson 2001: 241 (catalogue, faunistics); Pederzani and Reintjes 2002: 38 (faunistics); Nilsson 2015: 210 (catalogue, faunistics).
Laccophilus wittei Guignot 1952b: 3 (original description, faunistics); Guignot 1954: 28 (description, faunistics); Guignot 1955a: 29, 37 (faunistics, biology); Guignot 1959a: 544, 550 (description, faunistics); Medler 1980: 155 (faunistics, list); Nilsson 2001: 253 (catalogue, faunistics); Nilsson 2015: 219 (catalogue, faunistics). **New synonym.**

##### Type localities.

*Laccophilus burgeoni*: Zaire: de Kindu.

*Laccophilus wittei*: Zaire: PNU, Riv. Difiringi.

##### Type material studied

(5 exs.). *Laccophilus burgeoni*: Holotype: female: “Type Gschw. / Musée du Congo K. 300 de Kindu 14.V. 1911 L. Burgeon / Col. Gschwendtner / *Laccophilus burgeoni* Gschw. det. Gschwendtner / Type” (OLML).

*Laccophilus wittei*: Holotype: male: “Holotypus / Congo belge PNU Difirinji affl. g. Lufira (700 m) 27-IV-1949 Mis. G.F. de Witte 2732a / Coll. Mus. Congo (ex. coll. I.P.N.C.B.) / *Laccophilus wittei* Guign. Type, male symbol / Guignot det. 1952 *Laccophilus wittei* Guign. Type, male symbol” (MRAC). – Paratypes, males: “Congo belge: PNU Ganza pr., r. Kamandula (860 m) saline, 1-VI-1949 Mis. G.F, de Witte, 2648a / Paratype / F. Guignot det., 1953 *Laccophilus wittei* sp. n. / R.I.Sc.N.B. I.G. 24.054” (1 ex. IRSNB; habitus in Fig. 495); same data but “Loie affl. g. Lufira (1000 m) 6-III-1949” and “2666a” (1 ex. IRSNB); “Congo belge PNU Kabangey 1050 m VI-1949 de Witte / male symbol / paratype” (1 ex. MNHN). [Comments: Some confusion prevailed regarding status and location of the holotype of *Laccophilus burgeoni*. One specimen, deposited in MRAC is labelled as holotype of the species and the specimen is provided with label-data which is in accordance with original description. In Linz museum (OLML), however, there are two specimens labelled as types of *Laccophilus burgeoni*, both also provided with corresponding label data. Original description states that *Laccophilus burgeoni* is described on the basis of a single female specimen. All, three involved specimens are females. Having read the morphological description of the species, it is evident that the specimen in MRAC cannot be the holotype. On the other hand the description fits well with the two specimens in OLML, but how can there be two specimens marked as type when only one is mentioned in the description? This dilemma is interpreted as a case of later mislabelling in OLML. Original description gives as label data “K. 300 de Kindu (L. Burgeon)”. The label data of one of the two specimens coincides exactly with this while the other specimen is labelled “Kindu L. Burgeon”. Accordingly, the first mentioned specimen is considered to be the holotype.]

##### Additional material studied

(80 exs.). **Nigeria**: “River 3,5 mi. from Jos on Kaduna rd. 13.IV. 1963 JOC.” (2 exs. AMGS); “Trib. R. Gagere en rte Zaria-Katsina 5.10. 1963 JOC.” (1 ex. AMGS); “Dam, Vom?, Jos Plateau 11.IV. 1963 JOC.” (1 ex. AMGS); “Stream at Assob 36 mi. from Jos 13.IV. 1963 JOC.” (1 ex. AMGS); “R. Kaduna 4,5 mi. from Jos 13.IV. 1963 JOC.” (2 exs. AMGS); “Stream, escarpment Jos-Wambe rd. 13.IV. 1963 JOC.” (17 exs. AMGS); “Plateau Prov. Jos 14-17.3. 1949 Malkin leg. / Muddy running stream, gravel bottom” (38 exs. BMNH, 5 exs. MZH; habitus in Fig. 496); “Zaria 1969 Brancucci” (1 ex. NHMB). – **Sudan**: “Equatoria Lalyo-Juba 26-27.2. 1963 Linnavuori” (1 ex. MZH). – **Zaire**: “Type Gschw. / Musée du Congo Kindu L. Burgeon / Coll. Gschwendtner / *Laccophilus burgeoni* Gschw. det. Gschwendtner / Type” (1 ex. OLML; not type material); “Ituri Mahagi 19.V. 1925 / *Laccophilus wittei* Gschw. det. Guignot 1959” (1 ex. NHMB); “Lukonzolwa 9-17.2. 1931 de Witte” (1 ex. NHMB); “Musosa / 10. 1939 Bredo” (1 wx. MNHN); “PNU Mabwe 2.3. 1949 de Witte” (1 ex. MNHN); “Elisabethville 1935 Richard” (1 ex. NHMB). – **Uganda**: “Arua 24.2. 1931 Hancock” (3 exs. BMNH). – **Tanzania**: “NW Usagara 1700-1900 m 15.12. 1912 / *Laccophilus lineatus* Aubé det. Zimmermann / *Laccophilus wittei* Guign. det. Brancucci 1982” (1 ex. ZMHB); “Iringa 1.10. 1964 ex pond James” (1 ex. CGC).

##### Comments on synonymy.

Holotypes of *Laccophilus burgeoni* and *Laccophilus wittei* have been examined and compared. Minor difference is present in appearance of dorsal colour pattern but at least for the time being this is considered a case of ordinary variation within one species. *Laccophilus burgeoni*, being the older name is the valid name of the species.

##### Diagnosis.

*Laccophilus burgeoni* resembles much of *Laccophilus lineatus*. The two species are generally distinguished by differences in appearance of external colour pattern and by study of male genitalia. In *Laccophilus burgeoni* dark markings of pronotum are generally restricted to a narrow basal area and very rarely to a vague anterior marking (in *Laccophilus lineatus* both anterior and posterior dark markings of pronotum are distinct). Apex of penis in *Laccophilus lineatus* is shaped as a distinct knob while corresponding feature in *Laccophilus burgeoni* is absent or at most developed to a minor knob.

##### Description.

Body length 3.6–3.8 mm, width 1.9–2.2 mm. Habitus and dorsal colour pattern; exhibit some variation.

Head: pale ferrugineous. Rather shiny, although finely reticulated. Double reticulation fine but clearly discernible; large meshes contain two to six small meshes. At eyes with fine punctures.

Pronotum: Pale ferrugineous. Medially, at foremargin with vague, slightly darker area (area sometimes hardly discernible). Medially, at base with distinct but narrow, dark ferrugineous marking. Rather shiny, although finely reticulated. Reticulation partly double. Larger meshes contain two to nine small meshes. At margins with fine and irregular punctures, except basally in middle; impunctate.

Elytra: Colour pattern variable. Pale ferrugineous, with blackish to dark ferrugineous markings. Rarely, some specimens have slightly broader longitudinal lines, in part touching each other or than dark markings anteriorly on elytra are totally lacking (Figs 495–496). Submat due to dense microsculpture. Extensively with double reticulation; laterally and posteriorly double reticulation becomes indistinct. Almost impunctate; three indistinct, longitudinal rows of scattered, fine punctures may be discerned. Lateral, pre-apical furrow fine, pubescent.

Ventral aspect: Pale ferrugineous to ferrugineous, sometimes with vague, lateral, somewhat darker areas. Rather shiny, with very fine, in part hardly visible microsculpture. Abdomen with very fine, curved striae. Almost impunctate. Transversely on metacoxal plates located, shallow furrows discernible but weakly developed and in part reduced. Prosternal process slender, apex extended and pointed. Metacoxal process not modified. Apical ventrite with sublateral knob (Fig. 166).

Legs: Pale ferrugineous. Pro- and mesotarsus somewhat enlarged, provided with suckers. Claws of pro- and mesotarsus slightly extended, moderately curved.

Male: Genitalia: Penis quite long and somewhat twisted; extreme apex extended to a minor, hardly discernible knob; smetimes knob reduced and absent (Figs 348–349).

Female: Apical ventrite lacks lateral knob (Fig. 167). Pro- and mesotarsus slender.

##### Distribution.

Nigeria, Zaire, Uganda, Tanzania (Fig. 565). Guignot (1955a) gives Rwanda under the name *Laccophilus wittei*.

##### Collecting circumstances.

Very little information on ecology is available. Guignot (1955a) reports that *Laccophilus burgeoni* (under name *Laccophilus wittei*) is a rheophil species. Label data from Nigeria indicate that the species has been collected in a muddy running stream with gravel bottom.

#### Laccophilus lineatus

Taxon classificationAnimaliaColeopteraDytiscidae

Aubé, 1838

Figs 168–169 , 350–351 , 497–498 , 566

Laccophilus lineatus Aubé 1838: 426 (original description, faunistics); Sharp 1882: 287, 820 (description, faunistics); Kolbe 1883: 426 (description, faunistics); v. d. Branden 1885: 21 (catalogue, faunistics); Régimbart 1894: 237 (description, faunistics); Régimbart 1895: 141, 142 (description, faunistics, discussion, type locality given incorrectly); Alluaud 1897: 212 (faunistics); Régimbart 1906: 249 (faunistics); Régimbart 1908: 5 (faunistics); Peschet 1917: 24, 55 (description, faunistics); Zimmermann 1920a: 21 (catalogue, faunistics); Zimmermann 1920b: 225 (faunistics); Zimmermann 1926a: 23 (faunistics); Gschwendtner 1931: 180 (faunistics, description, discussion); Omer-Cooper 1931: 758 (description, biology, faunistics); Guignot 1942: 15 (description, discussion); Guignot 1946c: 263, 266, 267, 279, 281, 312 (description, faunistics); Guignot 1950b: 270 (discussion); Guignot 1954: 28, 29 (discussion); Vinson 1956: 28 (description, faunistics); Omer-Cooper 1957: 8, 9, 10, 90 (faunistics, discussion, description); Omer-Cooper 1958a: 59 (faunistics); Omer-Cooper 1958b: 37, 38, 39 (description, faunistics, biology); Guignot 1959a: 544, 549, 550, 551 (description, faunistics, discussion); Guignot 1959d: 161 (faunistics, discussion); Guignot 1961a: 929 (faunistics, discussion); Omer-Cooper 1962: 295 (faunistics, discussion); Omer-Cooper 1965: 76, 77 (description, discussion, faunistics); Bertrand and Legros 1967: 861, 867: (faunistics); Curtis 1991: 186 (faunistics); Pederzani 1988: 107 (faunistics, biology data available); Nilsson and Persson 1993: 80 (discussion); Nilsson 2001: 245 (catalogue, faunistics); Bilton 2014: 478 (faunistics, biology); Bilton and Gentili 2014: 400 (faunistics, biology); Nilsson 2015: 213 (catalogue, faunistics).
Laccophilus brevicollis Sharp 1882: 307 (original description, faunistics); Régimbart 1894: 237 (discussion, synonymy); Régimbart 1895: 141 (discussion, list, synonymy); Régimbart 1906: 249 (synonymy); Omer-Cooper 1931: 758 (synonymy); 1957: 9 (list, synonymy); Omer-Cooper 1965: 77, 78 (discussion, list, synonymy) Nilsson 2001: 246 (catalogue, faunistics, list, synonymy); Nilsson 2015: 213 (catalogue, faunistics, list, synonymy). **Confirmed synonym.**

##### Type localities.

*Laccophilus lineatus*: Mauritius: Ile de France. [Comment: according to Régimbart (1895), there is a mistake in original description; should be South Africa: Cape of Good Hope.]

*Laccophilus brevicollis*: South Africa: Cape of Good Hope.

##### Type material studied

(4 exs.). *Laccophilus lineatus*: Holotype: female: *“Laccophilus lineatus* Aubé, *irroratus* var., h. ad Cap. Bosp. n., D. Westerman / Data in NHRS JLKB 000030277 / Ex.-Museo Dejean / *lineatus* Aubé type = *brevicollis* Shp type / Dr. Régimbart vidit 1893 / *Laccophilus brevicollis* / D. Sharp Monogr. / coll. Oberthur” (MNHN).

*Laccophilus brevicollis*: Lectotype (by present designation): female: “Type / S. Africa / Type 570 *Laccophilus brevicollis* sp. n. Grahamstown / Sharp Coll. 1905-313 / *Laccophilus lineatus* Aubé J. Balfour-Browne det.” (BMNH; habitus in Fig. 497). – Paralectotypes, female: “570 / Cotype / S. Africa / Grahamstown C.G.H. / *Laccophilus brevicollis* Sharp co-type / *Laccophilus lineatus* Aubé J. Balfour-Browne det.” (2 exs. BMNH).

##### Additional material studied

(1316 exs.). **Tanzania**: “Tanganyika Nis(?)pasa R. 35 mi. from Mbeya on Tunduma rd. 14.10. 1948 JOC.” (4 exs. AMGS); “Tanganyika, small stream Rungwe 8.10. 1948 JOC.” (1 ex. AMGS); “Tanganyika Mbeya-Tunduma rd. 4.10. 1948” (1 ex. AMGS). – **Angola**: Ongueria Ca. 5300 ft. 12.6. 1954 / Side pools above waterfall” (1 ex. BMNH). – **Malawi**: “Nysld, mountain stream Dedza 29.9. 1948” (1 ex. AMGS); “Dedza dam on lower Lilongwe rd 29.9. 1948” (1 ex. AMGS); “Nysld Zombo Plateau, reservoir 7.11. 1948” (1 ex. AMGS); “Nysld R. Diedma Lilongwe rd.30.9. 1948” (1 ex. AMGS); “Nakwa Distr. 18. Oct. 1948 J.O-C.” (2 exs. AMGS); “Fort Hill, Yambe Stream 5.10. 1948 JOC” (3 exs. AMGS); “Balaka env. 5-6.1. 2002 Bezdek leg.” (1 ex. NMPC); “Selima env. 5-8.1. 2002 60 km E Lilongwe Kantner leg.” (1 ex. NHMB). – **Namibia**: “Namib Mt. Naukluft Riv. 24.16S-16.15E / 10.8. 1989 shore washing, river, Endrödy & Klimaszew leg, E-Y: 2644” (48 exs. TMSA, 5 exs. MZH; habitus in 498); same data but “Naukluft camp / 11.8. 1989 flowering bushes” (1 ex. TMSA); “Namib-Naukluft NP, 24.15.78S, 16.14.08E, Naukluft R., *Phragmites* grass + leaf litter, shore washing + sieving Uhlig” (2 exs. MZH, 1 ex. NMNW); “SWA Bullspoort Strey / *Laccophilus lineatus* Aubé det. J. Omer-Cooper” (1 ex. TMSA). – **Zimbabwe**: “S. Rhodesia stream of Salisbury” (1 ex. AMGS); “Mavhuradonha wilderness area 180 km N Harare 18.12. 1998 Kantner leg.” (1 ex. NMPC). – **Mozambique**: “Niassa Prov. Cmimulimuli Riv. S12°11.520’, E34°42.288’ Watson 10.2. 2008” (3 exs. CGF); “Niassa Prov. S12°26’, E34°42'24.4”, Stream 2 N of Nkwichi Lodge 31.3. 2009 Watson leg.” (1 ex. CGF). – **South Africa**: “Johannesburg Zumpt V. 1949” (1 ex. MNHN); “Johannesburg XI. 1950 leg. Zumpt” (1 ex. MNHN); “Johnsbg. Cookes Stream T. 13.10. 1901” (2 exs. TMSA); “Johannesburg, Bloubank Riv. 15.10. 1982 Bilardo / *Laccophilus lineatus* Aubé det. Bilardo” (2 exs. CSR); “Trsvl n. e. Mcapaan’s Port / Museum Paris coll. Maurice Régimbart 1908” (4 exs. MNHN); “Sikororo 7. 1922 v. Dam / *Laccophilus lineatus* Aubé det. J. Omer-Cooper” (7 exs. TMSA); “Trsvl Louis Trichart / 27.8. 1948 / *Laccophilus lineatus* Aubé det. J. Omer-Cooper” (1 ex. TMSA); “Tvl, Rustenburg 1-2.12. 1957 Rorke” (1 ex. TMSA); “Wylie’s Poort 5.11. 1920 Swierstra / *Laccophilus lineatus* Aubé det. J. Omer-Cooper” (1 ex. TMSA); “Trsvl E, Mariepskop 24.35S-30.50E / 6.5. 1981, from road puddle, Endrödy-Younga leg. E-Y: 1788” (7 exs. TMSA); “Trsvl NE, Macapan’s poort / Coll. Régimbart” (2 exs. NHMB); “Trsvl, stream Hwy R555 No, Stoffberg 10.12. 1995 Challet” (2 exs. CGC); “Trsvl Nylstroom 20.8. 1948” (5 exs. AMGS); “Trsvl Donkerpoort dam 1948 JOC.” (1 ex. AMGS); “Trsvl Nylstroom Donkerpoort dam 24.8. 1948 JOC.” (1 ex. AMGS); “Fount. Grove Pret. distr., 24.4. 1910 Swiestra / *Laccophilus lineatus* Aubé det. J. Omer-Cooper” (1 ex. TMSA); “Zpbg, Valdesia N. 1931 van Son / *Laccophilus lineatus* Aubé det. J. Omer-Cooper” (1 ex. TMSA); “Trsvl Koop R. Nr. Baberton 15. Dec. 1948 JOC.” (3 exs. AMGS); “Trib. of Koop R. nr. Baberton 14. Dec. 1948 JOC.” (1 ex. AMGS); “Trib. of Koop R. Nr. Nelspruit 1.12. 1948 JOC.” (1 ex. AMGS); “Trsvl Tambouti R. Aug. 1948 JOC.” (1 ex. AMGS); “Trsvl Brakfontein Tamboutie R. Waterberg distr. 19.8. 1948 JOC.” (2 exs. AMGS); “Trsvl Louis Trichart 27.8. 1948 JOC. / *Laccophilus lineatus* Aubé J. Balfour-Browne det.” (1 ex. AMGS); “Trsvl Duivels Kloof 24. N. 1948 JOC. / *Laccophilus lineatus* Aube J. Balfour-Browne det.” (2 exs. AMS); “Trsvl South Africa, Sandspruit, before klein Juskei R. N-26.030, E28.060, Venter 19.2 1964 (1 ex. AMGS); “Trsvl Juskei R at Rietfontein, N-26.140, E28.130, stones in current, 12.3. 1956 Allanson” (2 exs. AMSG); “Trsvl, Juskei R below Modderfonteinspruit, N-26.040, E28.110, 19.2. 1964 Allanson & Venter” (3 exs. AMSG); “Trsvl, Juskei R. below Sandspruit confl. N-26.010, E28.050, 13.3. 1956 Allanson” (6 exs. AMSG); “Trsvl, Juskei R. at Rietfontein, N-26.140, E28.130, 12.3. 1956 Allanson” (1 ex. AMGS); “Trsvl, Juskei R. at Buccleugh, N-26.070, E28.110, 8.2. 1956 Allanson” (1 ex. AMGS); “Trsvl, Juskei R. at Alexandra, N-26.100, E28.110, 7.2. 1956 Allanson” (1 ex. AMGS); “Trsvl, Juskei R. at Sandspruit Conf., N-26.010, E28.050, 9.2. 1956 Allanson” (8 exs. AMGS); “Trsvl, Sandfonteinspruit at Witkoppen N-26.070, E28.070, 14.3. 1956 Allanson” (10 exs. AMGS); “Trsvl, Braamfontein Stream at Witkoppen N-26.070, E28.040, 14.3. 1956 Allanson” (5 exs. AMGS); “Trsvl, Kliprivier Dam, N-26.410, E28.110, 30.7. 1971, Reavell” (1 ex. AMGS); “Trsvl, Ravine Stream, Bartlett’s Farm, Rustenburg N-25.880, E27.370 20.5.1971 Reavell” (12 exs. AMGS); “NPr., Rustemburg Res., fast flowing streams, 8.2. 1997 Turner” (1 ex. CCT); “Trsvl, Mountain stream in Magaliesberg Mnts. 11.9. 1972 Reavell” (1 ex. AMGS); “Trsvl, Randburg Stream N-26.070, E27.950, 6.6. 1971 Reavell” (29 exs. AMGS); “Trsvl, Bartlett’s Farm Rust, Dystrophic Dam N-25.880, E27.370, 20.5. 1971 Reavell” (1 ex. AMGS); “Trsvl, Pond So. Ermelo Hwy N11, 1.12. 1995 Challet” (2 exs. CGC); “Trsvl gravel pits Ermelo 8.12. 1948 JOC. / *Laccophilus lineatus* Aube J. Balfour-Browne det.” (1 ex. AMGS); “Transvl Ermelo Dec. 1948 JOC.” (1 ex. AMGS); “Trsvl Poerzyn R. Waterberg distr. 19. Aug. 1948 JOC.” (2 exs. AMGS); “Trsvl Del Kraal 10.8. 1948 JOC.” (1 ex. AMGS); “Trsvl Potgietersrust 23.4. 1933 Taylor” (1 ex. AMGS); “Trsvl E, Berlin; 300 m below, 25.33S-30.43E / 4.2. 1987 UV light collection, Endrödy-Younga leg, E-Y: 2416” (1 ex. TMSA); “Trsvl, Uitsoek Waterfall Area 25.16S-30.33E / 5.2. 1987 UV light collection, Endrödy-Younga leg, E-Y: 2421” (1 ex. TMSA); “Tv, Nelshoogte, gallery forest below St. 25.51S-30.53E / 4.12. 1987 UV light collection, Endrödy-Younga leg, E-Y: 2354” (1 ex. TMSA); “Trsvl 5 mi. W Warmbad 24-25.2. 1968 Spangler” (410 exs. USNM, 10 exs. MZH); “Pta, Fountains 5.11. 1932 van Son / *Laccophilus lineatus* Aubé det. Gschwendtner” (2 exs. TMSA); same data, but “det. J. Omer-Cooper” (8 exs. TMSA); same data, but “Oct. 1931” (1 ex. TMSA); “Fountains 26.8. 1905 / *Laccophilus lineatus* Aubé det. J. Omer-Cooper” (10 exs. TMSA); “Fount. Grove 27.8. 1915 Swierstra / *Laccophilus lineatus* Aubé det. J. Omer-Cooper” (20 exs. TMSA); “Koster 10. 1924 v. Dam / *Laccophilus lineatus* Aubé det. J. Omer-Cooper” (7 exs. TMSA); same data but ”det. Gschwendtner” (1 ex. TMSA); “Woodb. Vill. 4. 1915 Swierstra leg. / *Laccophilus lineatus* Aubé det. J. Omer-Cooper” (3 exs. TMSA); same data. but “det. Gschwendtner” (1 ex. TMSA); “Trsvl Buffel R at Hwy 30, 30.11.1995 Challet” (4 exs. CGC, 1 ex. MZH); “Trsvl Zoutpansberg Distr., Khalavha: L. Funduzi ca. 3000 ft., 24.4. 1954 / along muddy shore in weeds” (1 ex. BMNH); “W Prov., Soutpans 25,24S-27.33E/4.12.1996 on black light Müller leg. E-Y: 3256” (2 exs. TMSA); “N Prov., Geelhoutbosch Farm 24.22 E-27.34E / 14.1. 1999 at light Bellamy leg.” (1 ex. TMSA); “Trsvl Rhenosterpoort N.R. 25.45S-28.55E / 30.12. 1973 leg. Schulze” (2 exs. TMSA); “VAL 150F 12.6.56 (= Chutter, Vaal River below Klip River Confluence) (1 ex. AMGS); “Natal Bizana 13.4. 1947 JOC.” (1 ex. AMGS); “Natal Middld. Karkloof grassveld 29.19S-30.15E / 9.12. 1989, floating debris, dam, Endrödy & Klimaszew, E-Y: 2753” (3 exs. TMSA); “Natal Greyton Mountain stream 7.1. 1948 JOC.” (1 ex. AMGS); “Mpumalanga Leroro Burve’s Luck Potholes, Blyde Riv. Stillwasserzone Skale 29.5. 2001 / *Laccophilus lineatus* Aubé det. Hendrich” (1 ex. NMW); “Greyton Blinkwater Res., first stream from entrance 1100 mist belt grassland 2930 AB 1:50000 ref., 4.II. 1997 C.R. Turner / *Laccophilus lineatus* Aube Turner det. 97” (1 ex. AMGS); “Natal, nr. Drummond 1500 ft. 1.4. 1954 / small stream, stony & weedy pools” (5 exs. BMNH, 1 ex. MZH); “Kw. Natal, Lions R. at Weltevreden Farm, N-29.440, E30.150, 4.7. 1995 Dickens” (6 exs. AMGS); “Kw. Natal, Stream 2 mi E village, N27.179, E32.050, 17.7.1954 Oliff” (8 exs. AMGS); “Kw. Natal, *Typha* ditch, Reavell 20.1. 1989” (1 ex. AMGS); “Kw. Natal, Injambili R., inland S Coast rd. N-30.620, E30.520, 20.1. 1989 Reavell” (2 exs. AMGS); “Kw. Natal, Izotsha R., inland S Coast rd. 5.6. 1972 Reavell” (6 exs. AMGS); “Kw. Natal, Little Amanzimtoti R., N-30.060, E30.820, 15.6. 1984 Pretorius” (2 exs. AMGS); “Kw. Natal, Sinkwazi R., trib. from Flourspar mine N-29.14.19, E31.22.6, 14.7. 1964 Pretorius” (1 ex. AMGS); “Natal Pt Shepstone 1. 1913” (1 ex. SAMC); “Kw. Natal, above confluence with Bushman’s R., Little Bushman’s R., S-29.010, E29.880, 12.3. 1953 Oliff” (3 exs. AMGS); “Kw. Natal, Site 19 weir above Saicor N-30.169, E30.698, 12.10. 1996 Barber-James et al” (3 exs. AMGS); “Kw. Natal, Wartberg rd, Mploweni confluent N-29.464, E30.461, 17.6. 2004 Graham & Dickens” (1 ex. AMGS); “Kw. Natal, Umziki Pan nr. Hluhluwe, in swim pool, 20.4. 1997 Reavell” (1 ex. AMGS); “Kw. Natal, Mooi R. trib., nr Riverside, N-31.06, E28.19, 6.12. 1990 De Moor & Barber-James (3 exs. AMGS); “Kw. Natal, Nahoon R. at Wiutch Kranz, site NO, N-32.50.28, E27.39.21, 22.5. 2002 De Moor & Barber-James (1 ex. AMGS); “Kwazulu Natal” (1 ex. AMGS); “Natal, Malvern Sep. 1897 Marshall” (1 ex. BMNH); “Natal, Res. Pickle Pot on R617, 1000 m, fast stream 2.2. 1997 Turner” (4 exs. CCT); “Blinkwater Reserve, first stream from entrance, Greyton Natal, 1100 m mist belt grassland 4.2. 1997” (3 exs. CCT); “Kokstad 14.4. 1947” (1 ex. AMGS); “Mt Currie Distr., 14.4. 1947 (1 ex. AMGS); “Empangeni, Msintsi Stream, 2.3. 1990 Reavell” (1 ex. AMGS); “Nqutu 1953 Newton” (3 exs. BMNH); “Nqutu Zululd. 9.6. 1949 Newton” (1 ex. SAMC); same data but “14.5. 1949 (2 exs. SAMC); “ERS 36C (= WCPr. Krom River 23.3. 53, 33, 55,56S, 18,51,02 E), A.D. Harrison” (3 exs. AMGS); “Kimberly Bro. Bower 3. 1913” (7 exs. SAMC); “W.C. Mossel Bay, rte Herbertsdale-Langberg 19.1. 2001 Snizek Leg.” (1 ex. NMW, 1 ex. MZH); “C.Pr., Mossel Bay 2.1. 1992 Mazzoldi / Pond on road Oudtshoom m 500 / *Laccophilus lineatus* Aubé det. Mazzoldi 1992” (2 exs. CSR); “Tulbagh Gt Winthoek 3900 ft.” (1 ex. SAMC); “Tulbagh Lightfoot” (1 ex. SAMC); “W. C. Du Toits Mts 15 km E Paarl 33°45'S, 18°58'E, 1.3. 1997 Hess & Heckes leg. / *Laccophilus lineatus* Aubé det. Wewalka 2001” (4 exs. NMW); “W.C. Du Toits Mts 8 km SE Franschhoek (33.55S, 19.08E) Hess & Heckes 28.2. 1997 / Du Toits Riv. (Bergbach) S. Franschhoek –Pass 2,5 km von der Passhöhe o’hlb Strassenbrücke / *Laccophilus lineatus* Aubé det. Wewalka 1998” (2 exs. NMW); same data but ”9 km / Du Toits Riv. und kl. Seitenbach S Franschhoek-Pass (ca 5 km von Passhöhe, Höhe Rastplatz)” (3 exs. NMW); “WC, Franschhoek 25.3. 2001, rd R45, river 3 km SE Franschhoek Ribera & Cleslak leg.” (1 ex. CIR); “Upper Sources Olifants River, Ceres, CP / Dec. 1949” (1 ex. SAMC); “W.C. Hex River Mts, 7 km SW Ceres, (33.23S, 19.19E) 400 m NN. 26.2. 1997 Hess leg. / Breede Riv., Michell’s Pass, Bergbach, Sandtümpel, Rockpools, Nassmoose / *Laccophilus lineatus* Aubé det. Wewalka 1998” (5 exs. NMW); same data but “25.2./“Rockpools, Stillwasserzone, überrieseltes Moos” (4 exs. NMW); “WC, N-34.15.43., E18.23.16., alt 1 m, Olifants stream, Cape Point Res., 12.9. 2003 Turner, Mann & Reavell” (6 exx, CCT); “WC, Ceres, Townsrivier Rd N of Guydo Pass, sandy stream, N-33.10.27, E19.22.32, 3.9. 2003 Turner, Mann & Reavell” (3 exs. CCT); “WC, N-24.12.26, E13.25.17, 167 m, 13.6. 2003 Turner, Mann & Reavell” (1 ex. CCT); “WC, Stream in Vyeboom on R321 to Villierdorp, 22.2. 1997 Turner” (17 exs. CCT, 6 exs. MZH); “WC, Reservoir roadside to Bordjilesri, C.G.H. Res., Cape Town 15.2. 1997 Turner” (12 exs. CCT); WC, Cape Town, C.G.H. Res., pool 17.2. 1997 Turner” (2 exs. CCT); “WC, N-34.29.24, E20.05.37, alt. 51 m, River, De Hoop, Oaplas junct. 10.9. 2003 Turner, Mann & Reavell” (8 exs. CCT); “WC, N-34.27.23, E20.26.19, Grassy margins De Hoop Res, alt. 13 m, 9.9.2003 Turner, Mann & Reavell” (1 ex. CCT); “WC, N-34.18.56, E19.35.54, 122 m Reservoir on R316, ca 20 km S Caledon 10.9. 2003, Turner, Mann & Reavell” (1 ex. CCT); WC, rd 43, Michell’s Pass, riv. Breé in cross with R 46 Ribera & Cleslak leg. (1 ex. CIR); “ECPr., 7 km E Idutywa, small dirt pool, S32°07.169, E28°22.563, alt. 772 m, 23.1. 2005 Bergsten” (1 ex. NHRS); “ECPr., 60 km SE Idutywa, creek beside gravel road, S32°15.781, E28°45.576, alt. 96 m, 23.1. 2005 Bergsten” (3 exs. NHRS); “EC., pond on Hwy 344 at Adelaide 17.5. 2005 Challet” (2 exs. CGC, 1 ex. MZH); “ECPr., Howisons Poort, Palmiet R., N-33.330, E26,480, 2.3. 1964 Chutter” (6 exs. AMGS); “ECPr., TT Hool’s Farm, Slaaikraal Reservoir, New Year R., N-33.320, E26.530, 19.11. 1964 Chutter” (16 exs. AMGS); “ECPr., Mncotsho R., Trib. Buffalo R. N-32.54.17, E27.36.52.6, 11.12. 2003 De Moor & Barber-James” (2 exs. AMGS); “ECPr., Mncotsho R., Trib. Buffalo R. N-32.54.17, E27.36.52.6, 10.12. 2002 De Moor & De Moor” (4 exs. AMGS); “ECPr., Dam on Rwantsa R., N-32°53'20”, E27°37'55”, 30.8. 2000 De Moor & Barber-James” (1 ex. AMGS); “ECPr., Rwantsa R dam on Farm Mistrey, nr Roundhill site, N-32°53'20”, E27°37'55”, 10.11. 2000 De Moor & Barber-James” (1 ex. AMGS); “ECPr., Rwantsa R at Witchkranz, S-32°52'25”, E27°38'34”, 1.9. 2000 De Moor & Barber-James” (2 exs. AMGS); “ECpr., Rwantsa R. at Witchkranz, N-32°52'25”, E27°38'34” 9.11. 2000 DeMoor & Barber-James” (7 exs. AMGS); same but “7.6. 2000” (13 exs. AMGS); same but “16.1. 2001 Barber-James & Kohly” (9 exs. AMGS); “ECPr., Dam on Rwantsa R. of Farm Mistrey, N-32.53.20, E27.37.55 11.12. 2033 De Moor & Barber-James” (2 exs. AMGS); “ECPr., Rwantsa R at Wolsley, N-32°54'03”, E27°41'51”, 18.2. 2002, de Moor& Barber-James” (1 ex. AMGS); “ECPr., Xolo R., N-32°50'15”, E27°37'48”, 8.6. 2000 De Moor & Barber-James” (AMGS); “ECPr., Xolo R, dam at Lilly Stone Farm, N-32°52'10”, E27°38'55”, 16.5. 2001 De Moor & Barber-James” (1 ex. AMGS); “ECPr., Xolo R trib. carrying sewage discharge, N-32°50'11”, E27°37'49”, 9.11. 2000 De Moor & Barber-James” (2 exs. AMGS); same but “8.6. 2000” (2 exs. AMGS); same but “20.2. 2002” (1 ex. AMGS); “ECPr., Xolo R Dam at Lilly Stone Farm N-32°52'00”, E27°37'09”, 4.5. 2000 De Moor & Barber-James” (5 exs. AMGS); “ECPr., Rwantsa R., Farm Sebastopol, N-32°53'00”, 27°40'45, 7.5. 2000 De Moor & Barber-James” (4 exs. AMGS); “EC, Mncotsho R., 11,8, 2003, N-32°54'48”, E27°,36'52”, 11.8. 2003, De Moor & Barber-James” (1 ex. AMGS); “EC, Mncotsho R, trib. of Buffalo R., N-32°54'43”, E27°36'48”8.11. 2000 De Moor & Barber-James” (1 ex. AMGS); “EC. Muddy pool below small seep below pine plantation, N-31°04'00”, E28°09'02”, 27.3. 1993 De Moor & al.” (6 exs. AMGS); “ECPr., Nahoon R. at Witchkranz, N-32°51'10”, E27°39'08”, 19.5. 2004 De Moor & Barber-James” (1 ex. AMGS); same data but “N-32°50'28”, E27°39'21”, 22.5.2002” (1 ex. AMGS); “ECPr., Mncotsho R, Trib. Buffalo R. N-32°54'43”, E27°36'48”, 30.8. 2000, de Moor & Barber-James” (7 exs. AMGS); same but “18.5. 2004” (2 exs. AMGS); “CPr., Upper Gatberg R, at Madun, S-31.270, E28.170, 24.3. 1991 Barber-James & De Moor” (5 exs. AMGS); “Cape, Nqancule R, at Waterval, N-31.370, E28.220, 25.3. 1991 de Moor & Barber-James” (1 ex. AMGS); “Cape, Upper Klein Mooi R. at Fairvalley, N-31.130, E28.090, 12.5. 1990 Barber-James & De Moor” (4 exs. AMGS); “Cape, Wildebees R, at Glenelg, N-31.230, E28.0060, 12.6. 1990 Barber-James & de Moor” (1 ex. AMGS); “CPr., Hwy R102 nr Woodlands 7.12. 1995 Challet” (3 exs. CGC); “ECPr., ca 2 km N Queenstown, Longhill Game Reserve, pond with muddy water, S31°51.317, E26°51.322, alt. 1175 m, 19.1. 2005 Bergsten” (2 exs. NHRS); “EC, Great Brak R., HWY R56 at Schoombee 17.5. 2005 Challet” (2 exs. CGC);“EC, Queenstown 31.54S 26.51E, 27.4.1986 van Noort” (1 ex. SAMC); “Kowie Riv. nr. Grahamstown, Uys leg.” (4 exs. MZH); “Grahamstown distr. 6.4. 1946 Lovemore” (1 ex. AMGS); “Butterworth River 16.4. 1947 JOC.” (1 ex. AMGS); “Mt Ayliffe Distr., 5.4. 1947 JOC.” (1 ex. AMGS); “ECProv. Port St. Johns 15.2. 1956 J. O-C. / *Laccophilus lineatus* Aubé det. J. Omer-Cooper” (3 exs. AMGS); “ECPr. 7 km S of port of St. Johns, outside Silaka Nature Reserve, pond in grassland, S31°38.735, E29°29.299, alt. 265 m, 26.1. 2005 Bergsten leg.” (2 exs. NHRS); “ECPR. 7 km S of Port of St Johns, outside Silaka Nature Reserve, small vegetation rich pond S31°39,218, E29°29.133, alt. 222 m, 26.1.2005” (1 ex, NHRS); “E Cape Hogsback 19.VII. 1946 JOC.“ (1 ex. AMGS); “EC Hogsback 32.35S 26.56E 6.9. 1986 van Noort” (1 ex. SAMC); “Bathurst, Roundhill Reserve / 33.25S-26.53E, 27.2. 1994 Bruce-Miller” (1 ex. TMSA); “EC Pr. East London Gonubie Park, coastal pond 18.3. 1955” (1 ex. AMGS); “E. Cape Prov. Elliot 11.5. 1953 JOC.” (1 ex. AMGS); “ECPr., Ft. Forsdyce NR pond 32°40'S, 26"29'E P. Bulrisch leg. 1.12. 2009” (5 exs. NMPC, 1 ex. MZH); “ECPr., 15 km NW Stutterheim, grazed grassland, small creek, S32°25.654, E27°19.469, alt. 1071 m, 16.1. 2005 Bergsten” (1 ex. NHRS); “ECPr., S31°30,314, E26°32,766, alt. 1403 m, Ivan Hansen Prop., 3 km N Sterkstrom, cement pond, 20,1, 2005 Bergsten” (1 ex. NHRS); “ECPr., Kokstad 14.4.1947 / *Laccophilus lineatus* Aubé det. JOC.” (4 exs. NHMB); “CPr., Stream, 19 km on Brand R. rd S-33.890, E21.060, pool, 20.10. 1972 Stobbs” (3 exs. AMGS); “C.P., Grootvadersbos 1-6.11. 1940 v. Son / *Laccophilus lineatus* Aubé det. J. Omer-Cooper” (2 exs. TMSA); “C. Pr. Swartbg. Meiringspoort cent. 33.25S-22.33E / 1.11.1993, shore washing leg. Endrödy-Younga, E-Y: 2925” (1 ex. TMSA); “King Williams Town, Maden Dam 25.3. 1954 / Boggy pond close to Maden Dam” (11 exs. BMNH, 3 exs. MZH); “CPr., creek E Wilderness at Die Vleie 3.3. 1997 Challet” (2 exs. CGC, 1 ex. MZH); “Little Karroo, Kamanasiberg 33.37S-22.33E / 21.11. 1992 Water and shore, leg. Endrödy-Younga, E-Y: 2931” (38 exs. TMSA, 5 exs. MZH); “Little Karroo, Raubenheimer Dam E, 33.25S-22.19E / 21.10. 1993, shorewashing, leg. Endrödy-Younga, E-Y: 2888” (1 ex. TMSA); “Little Karroo Baviaanskloof N, 33.37S-24.15E / 28.10.1993 shorewashing, Endrödy-Younga leg., E-Y: 2917” (2 exs. TMSA); “C. Pr., 32°19.5'S-22°26.7’ Prov. Karroo NP, Permanent spring at Klipspringer Pass, grass + litter sievings + rivulet bank washing, Uhlig & Ndamane” (1 ex. ZMHB); “WC, stream N Theawaterskloof on R321 to Villiersdorp 22.2. 1997 Turner” (1 ex. CCT); “Pr. 5 mi SW Villiersdorp 11.2. 1951” (7 exs. MZLU); “Cape Reg., stream on road, Tzizikama 3.3. 1997” (4 exs. CGC); “C. Pr. Tzizikama Forest, Storms River Mouth 14.1. 1951 (3 exs. MZLU); “EC, Tzizikama N.P., stream, N-34.01.39, E25.53.28, 7.9. 2003 Turner, Mann & Reavell” (1 ex. CCT); “EC, Tzizikama NP, 2 m, N-34.01.39, E23.53.28, 7.9. 2003 Turner, Mann & Reavell” (1 ex. CCT); “EC. Tzizikama National Park, stream, 7.9. 2003 Turner, Mann & Reavell” (6 exs. CCT); “EC. N-34.01.45, E23.54.01, Tzizikama National Park, pools in forest stream, 7.9. 2003 Turner, Mann & Reavell” (4 exs. CCT); “WCN, N-34.00.49, E23.52.13, alt. Tzizikama National Park, alt. 194 m, stream and pools 8.9. 2003 Turner, Mann and Reavell” (11 exs. CCT); “EC, Humansdorp Dutchie R. 19.2. 1947 JOC.” (2 exs. AMGS); same data but “Barker’s Farm Feb. 1947” (7 exs. AMGS); same data but “Witte Els Bosch 14.2. 1947” (2 exs. AMGS); “Colesburg Van Wyks Fontein 23.2. 1947 JOC.” (2 exs. AMGS); “Riv. Homdini, Goukamma Bridge 15.3. 1954 / Edge of river, among thick weed” (1 ex. BMNH); “CPr, Pond No. Knysna, on HWY R340, 8.3. 1997 Challet” (1 ex. CGC); “Knysna Main Forest 1725 ft. 17.3. 1954 / Small pool in glade” (1 ex. BMNH); “C. Pr. Knysna Distr., Knysna-Avontoor Rd. 900 ft. 17.3. 1954” (2 exs. BMNH); “WC, Streams nr track to Brackenville Falls, E Knysna 23.2. 1997 Turner” (8 exs. CCT); “WC, Phantom Pass off N2 nr Knysna, ditches, 24.2. 1997 Turner” (11 exs. CCT); “CPr., Kromrivier, Cedarberg Mnts 12.2. 1997 Turner” (9 exs. CCT); N Cape, Kromrivier Fm, Cedarberg Mnts 12.2. 1997 Turner” (10 exs. CCT); “WC., Cederberg Wilderness Area, Sanddrift Camp 29.1.2005, camp, 32°29.27S, 19°16.13'E, 831 m Hotovy & Mateju” (5 exs. NMPC, 1 ex. MZH); “WC., Lake Wilderness, 22.2. 1997 Turner” (3 exs. CCT); “NC. Kromrivier Fm, Cedarberg Mnts, Turner” (27 exs. CCT, 6 exs. MZH); “C. Pr. Simons Town 12-20.4 1915, Cameroon” (4 exs. BMNH, 1 ex. MZH); “Cape, Langebg. Ruitersbos For. St. 33.54S-22.02E / 4.11. 1993 water surface Endrödy-Younga leg. E-Y: 2941” (3 exs. TMSA); “Clanwilliam Lightfoot” (1 ex. SAMC); “WCPr., stream at Parkhuis E of Clanwilliam 3.3. 1997 Challet” (1 ex. CGC); “Strandfontein June 1938” (1 ex. SAMC); “Stellebosch” (2 exs. SAMC)“Stellenbosch, Jonkershoek, Eerste River, 301 m, 3.3.10 S, 33°69'4.42 E 18°57'12.89 Hidalgo-Galiana & Terblacnche” (1 ex. CIR); “NW.Prov. Rustenburg Nat. Res. (= Kgaswane Mountain Res.) 25°42.92'S, 27°11.67E, 18.1. 2005, 1508 m, Hotovy & Mateju leg.” (3 exs. NMPC); “WC. Pr., Kirstenbosch cultural garden dam, Cape Peninsula 3.9. 2007, 33.99817S, 18.42905E Pryke leg. / *Laccophilus lineatus* Aubé det. Turner” (1 ex. CCT); “WC. Pringle bay Rd. 21.3. 2001, pond in Harold Porter bot. gard Ribera & Cieslak” (1 ex. CIR); “WC, Du Toits Kloof 24.3. 2001rd N 1 pond and river Wit in resort, Ribera & Cieslak” (1 ex. CIR); “WC. St. 15 Vergelegen dam 3, Pond near river, connected to river, 18.89125N, -34.07397S, alt. 85 m / *Laccophilus lineatus* Aubé det. Turner” (1 ex. CCT); “C.P. 14 mi NE Wellington 4.3. 1968 Spangler” (110 exs. USNM, 2 exs. MZH); “White River Wellington 1500 ft.” (5 exs. SAMC); “C. Town” (23 exs. SAMC); “Cape Town 1892” (3 exs. SAMC); “Cape of Good Hope nr Junction M64 & M65 26.2. 1997 Challet” (4 exs. CGC); “Cape of Good Hope Nature Reserve 7-10.3.1968 Spangler leg.” (130 exs. USNM); “Cape Good Hope Jan. 1817(?)” (1 ex, ZMUC); “Cap b. sp. De Vylder” (5 exs. NHRS); “Caffraria / J. Wahlb.” (1 ex. NHRS); “Misc 53D 20.10.53 A.D. Harrison” (= Palmiet R., Elgin Forest Reserve causeway) (1 ex. AMGS); “K.R.L. 122B” (= Harrison, Klein River Lagoon, Hermanus) (1 ex. AMGS); “GBG 470 G 7.12.51” (= Harrison, weedy backwater on forest stream, Great Berg River) (1 ex. AMGS); “Mt-aux-Sources ca. 4000 ft, 5.4. 1954, in weedy stream” (2 exs. BMNH); “Transkei Port St. Jones, Silaka 31.33S-29.30E / 30.11. 1987 water collection, leg. Endrödy-Younga, EY: 2543” (1 ex. TMSA). – **Swaziland**: “Little Usutu R. nr. Bremersdorp 5.12. 1948” (1 ex. AMGS); “Swzld 9 mi. from Mbabane 6.12. 1948” (6 exs. AMGS). – **Lesotho**: “Basutoland stream at Bushmans caves nr. ? Tyalaganeng 4.12. 1948” (4 exs. AMGS).

##### Specimen with uncertain locality.

“Stream forest nr. Mngesha ? 24.2. 1926” (1 ex. AMGS).

##### Comments on synonymy.

Holotype of *Laccophilus lineatus* and lectotype of *Laccophilus brevicollis* have been compared and found to be conspecific; earlier synonymy is herewith confirmed.

##### Diagnosis.

*Laccophilus lineatus* resembles most of all of *Laccophilus burgeoni*. The two species are distinguished by study of the male genitalia; penis apically with a small but distinct knob/process in *Laccophilus lineatus* while corresponding knob is reduced to a minor extension or it is absent in *Laccophilus burgeoni*.

##### Description.

Body length 3.7–4.0 mm, width 2.0–2.2 mm. Dorsal colour pattern of body fairly uniform, exhibits only some modest variation from its ground plan.

Head: Pale ferrugineous. Slightly mat, finely microsculptured. Reticulation double. Large meshes only slightly more strongly developed than small meshes. Large meshes contain generally 2–5 small meshes. Small meshes sometimes weakly developed and hardly discernible. Impunctate, except at eyes; with some scattered, irregularly distributed, small punctures.

Pronotum: Pale ferrugineous. Medio-frontally and –basally with a distinct, blackish to dark ferrugineous marking. Slightly mat, microsculptured. Reticulation double; small meshes sometimes indistinct, hardly discernible. Large meshes may contain 2–5 small meshes. Laterally and frontally, with fine, often rather indistinct, scattered punctures.

Elytra: Pale ferrugineous, with distinct blackish- to dark ferrugineous, longitudinal markings. Sometimes dark longitudinal lines in part confluent, forming a larger dark area (Figs 497–498). Rather shiny, finely microsculptured. Reticulation double; laterally and posteriorly reticulation become indistinct. Large meshes contain generally 2–5 small meshes. Almost impunctate. Scattered, irregularly distributed, sparse punctures form a discal row. Dorsolateral and lateral rows of punctures sparser and more irregular than discal row. Laterally with a pre-apical, finely pubescent furrow.

Ventral aspect: Pale ferrugineous to ferrugineous, prothorax slightly paler; pale ferrugineous. Rather shiny, although very finely microsculptured; in part reticulation indistinct. Abdomen with fine, curved striae. Prosternal process fairly slender, apex extended, pointed. Metacoxa in anterior half with more or less transverse, shallow furrows, a part of which are mixed and reduced. Apical ventrite asymmetric, with one, small, lateral knob (Fig. 168).

Legs: Pro- and mesotarsus slightly enlarged, extended, provided with suckers.

Male genitalia: Penis in lateral aspec slightly twisted; extreme apex extended to a small but distinct process (Figs 350–351).

Female: Externally as male but pro- and mesotarsus slender. Apical ventrite lacks asymmetric, lateral knob (Fig. 169).

##### Distribution.

Tanzania, Angola, Malawi, Zimbabwe, Mozambique, Namibia, South Africa, Swaziland, Lesotho (Fig. 566). Records from Mauritius are to be considered incorrect. Due to widespread, common problems in identification of *Laccophilus lineatus*, only verified records are included in the present map.

##### Collecting circumstances.

Detailed information is lacking. Label data indicate that the species has been collected in pools and puddles at streams, often with weed. The species has also been sampled from gravel pits, dams and a boggy pond. Some records are from mountain areas up to ca. 7500 ft. a. s. l. The species has also been sampled at light collection. Some additional information on ecology may be yielded from Omer-Cooper (1958b).

#### Laccophilus brancuccii sp. n.

Taxon classificationAnimaliaColeopteraDytiscidae

http://zoobank.org/0F88BD88-DA0A-4396-8621-84F4244DEF21

Figs 170 , 352 , 499 , 567

##### Type locality.

Central African Republic: Bozo.

##### Type material

(1 ex.). Holotype, male: “Bozo (lum.) XI. 1981 / R. Centr. Afr. N. Degallier” (NHMB; habitus in Fig. 499).

##### Diagnosis.

*Laccophilus brancuccii* resembles most of dark specimens of *Laccophilus contiro* but it is separated externally from this species by smaller body. Moreover, distinct differences in shape of penis apex separate the two species; penis of *Laccophilus contiro* is almost straight and almost evenly broad while penis of *Laccophilus brancuccii* is slightly curved and medially slightly expanded.

##### Description.

Body length 3.5 mm, width 2.0 mm. Dorsal, colour pattern of body distinct (Fig. 499).

Head: Pale ferrugineous; at pronotum head slightly darker but change to darker colour is gradual. Submat, finely microsculptured. Reticulation double. Larger meshes only slightly more strongly developed than small meshes. Large meshes contain 3–5 small meshes. Impunctate, except at eyes, with fine, irregular punctures, the area of which extends for a short distance towards middle of head-disc.

Pronotum: Pale ferrugineous, at base and frontally in middle with a fairly broad blackish to dark ferrugineous marking. Submat, finely and densely microsculptured. Large meshes somewhat more strongly developed than fine meshes; large meshes contain 2–5 fine meshes. Impunctate, except frontally and laterally; with very fine to fine, irregular punctures.

Elytra: Blackish ferrugineous with rather limited pale ferrugineous colour pattern; humeral region with 2–3 small, pale spots, Posterior to middle with an irregular, transverse, pale marking, interrupted narrowly at suture. Apically, with some irregular, rather small, pale spots (Fig. 499). Submat, finely and densely microsculptured; reticulation double but large meshes strongly reduced and only rudiments discernible, in part large meshes absent. Impunctate, except for a discal row, the punctures of which are fine and irregularly distributed. Outside discal row, a dorsolateral and a lateral row are indicated by a few, fine punctures. Preapical–lateral row of punctures finely pubescent.

Ventral aspect: Blackish to dark ferrugineous, prothorax paler. Rather shiny although in part very finely microsculptured. Basal ventrites with fine, curved striae. Impunctate, except for some fine punctures apically on abdomen. Apical ventrite broken (one lateral part is lost), as in Fig. 170. Metacoxal plates with very fine, shallow, in part indistinct, transverse furrows. Apex of prosternal process broken.

Legs: Pale ferrugineous to ferrugineous. Pro- and mesotarsus somewhat enlarged, with suckers.

Male genitalia: Penis in lateral aspect quite broad, from base to middle almost straight, medially bent and slightly enlarged. Extreme apex broad, weakly pronounced (Fig. 352).

Female: Unknown.

##### Etymology.

The name is a noun in its genitive form based on the name of the late Dr. Michel Brancucci, Basel, Switzerland, who kindly provided a large material of African *Laccophilus* for this revision, including the holotype of the new species. During the years Brancucci worked intensively with the taxonomy of the genus *Laccophilus* and the tribe Laccophilini.

##### Distribution.

Central African Republic, only known from the type locality (Fig. 567).

##### Collecting circumstances.

Almost unknown, collected with light.

#### Laccophilus incomptus sp. n.

Taxon classificationAnimaliaColeopteraDytiscidae

http://zoobank.org/72CB9B6C-66FA-4B4D-A217-0ECF944D1BE8

Figs 171–172 , 353 , 500 , 567

##### Type locality.

Cameroon: Subd. Bétaré-Oya, Bindiba.

##### Type material

(2 exs.). Holotype: male: “Fr. Cameroons, Bindiba, subd. Bétaré-Oya 19-22.vii. 1949 B. Malkin / Running water, muddy over gravel / Brit. Mus. 1956-234” (BMNH; habitus in Fig. 500). – Paratype: female: Same label data as holotype (1 ex. BMNH).

##### Additional material studied

(non-type). **Cameroon**: “Foumbot Fev. 67 / Cameroun B. de Miré”. [Comment: only male genitalia left on a label while glued specimen is lost. The male genitalia are identical with those of the holotype.]

##### Diagnosis.

*Laccophilus incomptus* resembles a number of species placed in species group 13 (*lineatus* group) and characterized by generally distinct, longitudinal markings on elytra. The new species is possibly closest related to *Laccophilus flavoscriptus*, which has an evenly broad and curved penis, extreme apex being almost unmodified. Penis of *Laccophilus incomptus* in lateral aspect is plain, exhibiting hardly any modifications; basally quite broad and almost straight, medially bended and towards apex penis slightly tapers to a broad, truncate end.

##### Description.

Body length 3.6–3.9 mm, width 2.0–2.2 mm. Body dorsally with distinct, somewhat variable colour pattern. Base of elytra with complete dark area or corresponding dark area divided into a few, variable spots (Fig. 500).

Head: Pale ferrugineous. Posteriorly at pronotum with a vague, slightly darker area. Rather shiny, although finely microsculptured. Reticulation double. Large meshes of microsculpture slightly more strongly developed than small meshes. In lateral areas of head size categories of microsculpture gradually disappears. Large meshes, when discernible, contain 2–5 small meshes. Impunctate, except at eyes, with fine, irregular punctures. Area of punctures extends towards centre of head-disc.

Pronotum: Pale ferrugineous. Anteriorly on area between eyes with a vague, dark ferrugineous to ferrugineous area. At base in middle, with a narrow and distinct dark brownish area. Rather shiny, although finely microsculptured. Reticulation double, large meshes contain 2–5 small meshes. Impunctate, except at margins; fine, scattered punctures discernible except at base in middle.

Elytra: Pale ferrugineous, with distinct, somewhat variable, dark ferrugineous markings (Fig. 500). Slightly mat, with fine and dense microsculpture. Reticulation double but large meshes extensively almost rudimentary and in part difficult to discern; large meshes only slightly more strongly developed than small meshes. Almost impunctate. Sparse, irregular punctures form a discal row. Dorsolateral and lateral rows indicated by some scattered fine punctures. Pre-apical, lateral row of punctures forms a shallow furrow provided with fine hairs.

Ventral aspect: Ferrugineous except prothorax, pale ferrugineous. Rather shiny, finely microsculptured. Ventrites with fine, slightly curved striae. Metacoxal plates with about 10 almost transversely located, shallow furrows; in part furrows reduced and indistinct. Plates laterally close to epipleura with a distinct, longitudinal impression. Prosternal process slender, posteriorly slightly extended, apically pointed. Impunctate, except apical ventrite; with some fine, scattered punctures and an asymmetric, fine knob on one side (Fig. 171).

Legs: Pale ferrugineous to ferrugineous. Pro- and mesotarsus slightly enlarged, with distinct suckers.

Male genitalia: Penis in lateral aspect is plain, exhibiting hardly any modifications; basally quite broad and almost straight, medially bended and towards apex penis slightly tapers to a broad, truncate end (Fig. 353).

Female: Apical ventrite lacks asymmetric knob (Fig. 172). Pro- and mesotarsus slender.

##### Etymology.

The species name *incomptus* is a Latin adjective meaning “unadorned”. It here refers to the simple shape of aedeagus, which seems to be a characteristic feature of the new species.

##### Distribution.

Cameroon (Fig. 567).

##### Collecting circumstances.

According to collecting label associated to the specimens, the new species *Laccophilus incomptus* was sampled in running water, the bottom obviously gravel and covered with mud.

#### Laccophilus secundus

Taxon classificationAnimaliaColeopteraDytiscidae

Régimbart, 1895

Figs 173–175 , 354–355 , 508–509 , 573

Laccophilus secundus Régimbart 1895: 146 (original description, faunistics); Zimmermann 1920a: 25 (catalogue, faunistics); Peschet 1925: 31 (faunistics); Gschwendtner 1930: 90 (faunistics); Gschwendtner 1931: 181 (faunistics); Omer-Cooper 1931: 759 (discussion); Gschwendtner 1935a: 15 (faunistics); Gschwendtner 1938b: 337 (faunistics); Guignot 1943: 99 (faunistics); Guignot 1959a: 577, 578, 581 (description, faunistics); Omer-Cooper 1965: 76, 80 (description, discussion, faunistics); Legros 1972: 467 (faunistics); Bilardo and Pederzani 1978: 119 (discussion, faunistics, description); Pederzani and Rocchi 1982: 72 (faunistics); Bilardo and Rocchi 1987: 104 (faunistics, biology); Bilardo and Rocchi 1990: 160, 162, 177 (faunistics, biology); Nilsson 2001: 250 (catalogue, faunistics); Bilardo and Rocchi 2002: 174 (list, faunistics); Bilardo and Rocchi 2008: 211, 236 (faunistics, biology); Bilardo and Rocchi 2013: 141 (faunistics, biology); Nilsson 2015: 217 (catalogue, faunistics).
Laccophilus torquatus Guignot 1956c: 318, 320 (original description, faunistics); Guignot 1956b: 219 (faunistics, discussion); Guignot 1956e: 52 (discussion, female ab. description); Omer-Cooper 1958b: 37, 38, 39, 41 (description, faunistics, biology); Guignot 1959d: 160 (discussion, faunistics); Bruneau de Miré and Legros 1963: 873, 888 (faunistics); Omer-Cooper 1965: 76, 80 (description, discussion, faunistics); Bertrand and Legros 1967: 862 (faunistics); Bilardo and Pederzani 1978: 119 (discussion); Nilsson and Persson 1993: 81, 94 (faunistics); Nilsson 2001: 252 (catalogue, faunistics); Nilsson 2015: 218 (catalogue, faunistics). **New synonym.**

##### Type localities.

*Laccophilus secundus*: Zaire: Boma.

*Laccophilus torquatus*: Zaire: Kivu, Kavimvira (Uvira).

##### Type material studied

(21 exs.). *Laccophilus secundus*: Lectotype (by present designation): male: “Congo belge Boma / male symbol / Cotype” (MNHN). – Paralectotypes: “Gabon Mocquerys / female symbol / Cotype” (1 ex. MNHN); same data as preceding, but additionally labelled “Museum Paris Coll. Maurice Régimbart / *secundus* Rég.” (4 exs. MNHN).

*Laccophilus torquatus*: Holotype: male: “Holotypus / I.R.S.A.C. –Mus. Congo Kivu: Kavimvira (Uvira) (à la lumière) IX/X-1954 G. Marlier / Type male / F. Guignot det., 1955 *Laccophilus torquatus* sp. n. Type male” (MRAC). – Paratypes: Same sampling data as in holotype but labelled as “Paratype / R. DET. 6777” (7 exs. MRAC, 1 ex. IRSNB); same sampling data but “XII-1954” (1 ex. IRSNB, 1 ex. AMGS; status as paratype uncertain); “Soudan Egyptien Roseires (Ht Nil Bleu) Ch. Alluaud 1906 / Paratype” (1 ex. MNHN); “Ethiop. Merid. Bourié, Bord de la Riv. Omo 600 m / Mission de l’Omo / Paratype” (1 ex. MNHN); “Senegal IFAN – 1948 Tianaga / Paratype” (1 ex. MNHN); “Afrique Orient. Angl. Kisoumou Baie Kavirondo Ch. Alluaud 1909 / Paratype” (1 ex. MNHN).

##### Additional material studied

(233 exs.). **Sudan**: “Minkammon 31,31E, 6,2N, 16-17.1. 1954 JJOC.” (2 exs. AMGS); “1 mi. from Tali Post 5,53N, 30,47E 14.1. 1954 JJOC.” (1 ex. AMGS); “Tombe 17.1. 1954 JJOC.” (3 exs. AMGS); “R. Lau at Payii 7 mi. west of Yirol 17.I. 1954 JOC.” (1 ex. AMGS); “L. Nyibor 25.I. 1954 JJOC.” (1 ex. AMGS); “L. Nyibor II. 1954 JOC.” (2 exs. AMGS); “Tombe 17.1. 1954 JJOC.” (5 exs. AMGS); “L. Baya 6.II. 1954 JOC.” (1 ex. AMGS); “Khor Gwaar 31,34E, 5,7N, 17.1. 1954 JJOC.” (1 ex. AMGS); “Upper Nile Malakal 5-20.1. 1963 Linnavuori“ (3 exs. MZH). – **Ethiopia**: “Bahar Dar, at light 4.4.1967 P. Stys leg.” (1 ex. NMPC). – **Benin**: “Dep. Mono, Lakossa, Doukonta (village) 2.2.2006 leg. Goergen, Komarek & Hounguè / 06°40'21,3"N, 01°41'33,5"E, ca. 40 m asl, very slowly running stream” (1 ex. NMW, 1 ex. MZH); “Dep. Atlantique, Glotomè (village) 1.2. 2006 leg. Goergen, Komarek & Hounguè / 06°41'06,8"N, 02°02'36,8"E, 17 m asl, slowly running stream” (1 ex. NMW). – **Nigeria**: “Ondo Prov. Akure 30.1.1949 Malkin / muddy pool, gravelly bottom” (1 ex. BMNH). – **Cameroon**: “Matute, Tiko Plantation 24.4.-6.5. 1949 Malkin / at light” (1 ex. BMNH); “Dimako 12-13.6. 1973 Linnavuori” (2 exs. MZH). – **Gabon**: “Ogové Riv., Good leg.” (1 ex. CSR); “Makokou 1-14.5. 1975 Mateu / *Laccophilus secundus* Régb. det. Hájek” (4 exs. NHMB); “Makokou 1-30.IV. 1971 Mateu light / *Laccophilus secundus* Rég. det. Brancucci” (5 exs. NHMB). – **Central African Republic**: “Bozo 21.5. 1981 / Degallier” (1 ex. NHMB); Bozo 12. 1981 / Degallier” (1 ex. NHMB). – **Congo**: “Rep. pop., Plateau Koukouya, Lekana 9.4. 80 Onore” (1 ex CSR); same data but “4. 1980 (stagno soleggiata in savana)” (3 exs. NHMB); same data but “m. 850” (1 ex. NHMB); “Voka prés de Boko I / 1980 Onore / *Laccophilus secundus* Régb. det. Pederzani” (1 ex. NHMB). – **Zaire**: Same sampling data as paratypes above, except “6. 1955” (2 exs. IRSNB); “PNG II/fd/13, 5.5. 1952 De Saeger 3421” (1 ex. MRAC); “PNG PpK.51/g/9, 2.4. 1952 De Saeger 3272” (10 exs. MRAC, 3 exs. MZH); “PNG II/fd/12, 10.3. 1952 De Saeger 3180”(3 exs. MRAC); “PNG II/fd/12, 6.3. 1952 De Saeger 3886” (1 ex. NHMB); “PNG II/gd/8, 10.4. 1952 De Saeger 3316” (1 ex. MRAC); “PNG II/fd/14s, 3.4. 1952 De Saeger 3278” (2 exs. MRAC); “PNG II/fd/14, 28.1. 1952 De Saeger 3061” (1 ex. MRAC); “Tshuapa Bamanya 1968 P. Hulstaert” (1 ex. MRAC); “Kivu Kavimvira (Uvira), à la lumière 10. 1955 Marlier” (1 ex. MRAC); same data but “9-10. 1954” (1 ex. MNHN); same data but “12. 1954” (3 exs. MRAC, 1 ex. MZH, 2 exs. NHMB; habitus in Fig. 508); same data but “6. 1955” (4 exs. MRAC); same data but “I. 1955” (6 exs. MRAC, 2 exs. MZH); same data but “1. 1956” (1 ex. NHMB); “Tshuapa-Mbandaka ca. 0°03'N, 18°28'E, a.l., 1964 A.B. Stam” (26 exs. RMNH, 2 exs. MZH); same data but “3-4.4. 1963” (7 exs. RMNH); same data but “17-18.5. 1963” (9 exs. RMNH); same data but “24-25.5. 1963” (1 ex. RMNH); same data but “zonder datum” (3 exs. RMNH, 1 ex. MZH); same data but “18-19.3. 1962” (2 exs. RMNH); same data but “2-3.3. 1963” (4 exs. RMNH); same data but “8-22.10. 1962” (10 exs. RMNH); “Balenge nr Mbandaka ca. 0°03'N, 18°28'E, 14.5. 1963 A.B. Stam” (2 exs. RMNH); “Coquilhatville 3-4.4. 1963 Stam / at light” (1 ex. RMNH); same data but “27-28.4. 1963” (1 ex. RMNH); same data but “13.5. 1963” (1 ex. RMNH); same data but “26-27.6. 1963” (1 ex. RMNH); same data but “16-17.7. 1963” (1 ex. RMNH, 1 ex. MZH); same data but “10-19.4. 1963” (1 ex. MZH); same data but “20-21.1. 1962” (2 exs. RMNH); same data but “10-11.6. 1962” (2 exs. RMNH, 3 exs. MZH); same data but “11-12.6. 1962” (2 exs. RMNH); same data but “Hygiene Publique 20.5.-3.6. 1963” (2 exs. RMNH); same data but “4-5.7. 1963” (1 ex. RMNH); same data but “17-18.6. 1963” (1 ex. RMNH); “Dima 23.9.1908 A. Koller / *Laccophilus secundus* Régb. det Gschwendtner” (1 ex. OLML); “Bukama 7. 1937 Lt. Marée / *Laccophilus secundus* Régb. det Gschwendtner” (1 ex. OLML); “Elisabethville 2. 1940 H.J. Brédo / *Laccophilus secundus* Régb. det Gschwendtner” (1 ex. OLML); “Elisabethville, á la lum., 1953-1955 Seydel” (1 ex. NHMB). – **Uganda**: “Jinja L. Victoria, Malaise trap 17.9. 2003 Prikryl I. leg.” (1 ex. NMPC). – **Kenya**: “Naivasha Lake 22-27.10. 1995 Wewalka / *Laccophilus torquatus* Guign. det. Rocchi 2003” (1 ex. RMNH); same data but no determination label (11 exs. CGW, 2 exs. MZH); “Naivasha Crater Lake 26.10. 1995 Wewalka” (1 ex. CGW). – **Tanzania**: “E Usambara Mts, Amani Pond 1000 m, 20.7. 1980 Stoltze & Scharff” (1 ex. ZMUC, 1 ex. MZH); same data but “Dodwe Stream 900 m, 10.7. 1980” (1 ex. ZMUC); “L. Malawi Matena 1.7. 1979 Stoltze leg.” (1 ex. ZMUC); “Tang. Terr. Ukerewe I., Father Conrad” (1 ex. BMNH). – **Zambia**: “Luapula Prov., Lake Bangwulu, Chilubi 11.2. 1982 J. Selander” (3 exs. MZH). – **Zimbabwe**: “Victoria Falls, Zambezi NP camp, 17°53'S, 25°49'E, 11-12.12. 1993 lux, Uhlig” (1 ex. ZMHB). – **Namibia**: “E Capriwi, 30 km SE Katima Mulilo 17°31'S, 24°25'E, Zambesi Altwasserarm, lux 6.3. 1992 Uhlig” (8 exs. ZMHB, 2 exs. MZH; habitus in Fig. 509); “E Capriwi, Mundumu NP, Buffalo Trails Camp, lux ca. 18°10'S, 23°26'E, 13.3. 1992 Uhlig” (2 exs. ZMHB, 1 ex. MZH); “E Capriwi, Katima Mulilo 17°29'S, 24°17'E, Gesiebe, Geschwemme Tümpelufer 7.3. 1992 Uhlig” (1 ex. ZMHB, 1 ex. NMNW); “E Capriwi, Mundumu NP Nakatwa 18°10'S, 23°26'E,8-13-3. 1992, lux Göllner” (5 exs. ZMHB); “Kavango Popa Falls 18°07'S, 21°35'E, lux 26.2-3.3. 1992 Uhlig” (1 ex. ZMHB); same data but “Göllner leg.” (1 ex. ZMHB); “Kavango, Mahongo Game Res. 18°14'S, 21°43'E, piknik site lux, 1.3. 1994 Uhlig” (2 exs. ZMHB, 1 ex. MZH). – **Botswana**: “Sitatunga Camp, SE Maun 24°04'33"S, 23°21'16"E, 7.3. 1993 lux, Uhlig” (1 ex. ZMHB); “Tsotsorogo Pan 17.6.-9.7. 1930 V.-L. Kal. Exp. / *Laccophilus secundus* Régb., det. Gschwendtner” (1 ex. TMSA). – **South Africa**: “Kwa Zulu Natal St. Lucia park, 28°12'S, 32°25'E, Koch” (1 ex. ZMHB); “Natal, Waterton Timber Co. 3. 1985, N-28.20.5, E32.14, at light Atkinson” (1 ex. NHMB).

##### Comments on synonymy.

Lectotype of *Laccophilus secundus* and holotype of *Laccophilus torquatus* have been examined and compared. Male genitalia of both taxa are identical but elytral colour pattern exhibits a clear difference. The appearance of elytra is, however, variable and there is a series of intermediates between two extremes. Accordingly, it seems clear the two species are conspecific. *Laccophilus secundus* is the valid name of the species, being the older one of the two available names.

##### Diagnosis.

The peculiar shape of penis in combination with small-medium sized body and blackish elytra with pale markings separates *Laccophilus secundus* from all other African *Laccophilus* species, except of *Laccophilus australis* sp. n. Penis of *Laccophilus secundus* is delicate, almost straight and extreme apex strongly bent forming a minor lateral extension. Penis of *Laccophilus australis* is slightly larger and apical extension, distinctly longer.

##### Description.

Body length 3.0–3.4 mm, width 1.6–1.9 mm. Dorsal, colour pattern of body variable between extremes (Figs 508–509).

Head: Pale ferrugineous. Submat, finely microsculptured. Reticulation double. Large meshes in part reduced and difficult to discern; when discernible they may contain 3–7 small meshes. Almost impunctate except at eyes, with dense and irregularly distributed fine punctures.

Pronotum: Pale ferrugineous to ferrugineous; basally in middle often with a distinct, blackish area. Rather shiny, although densely and finely microsculptured. Reticulation double but large meshes in part reduced and only slightly more strongly developed than fine meshes. When discernible, large meshes may contain 3–9 small meshes. Frontally and laterally, with fine irregular punctures.

Elytra: Blackish to blackish ferrugineous with variable, pale ferrugineous markings. At base with a single, humeral, pale spot, which sometimes is replaced by two or three small, pale spots, which form a subbasal, transverse area. Posterior to elytra-middle and apically with slightly vague, variable, pale markings, which sometimes can be rather indistinct. (Figs 508–509). Submat, finely and densely microsculptured. Reticulation double but large meshes reduced, weakly developed and in large areas difficult to discern. Discal row of punctures consists of irregularly located fine punctures. Lateral row and especially dorsolateral row of punctures indistinct, only indicated by a few, fine punctures.

**Figures 173–185.**
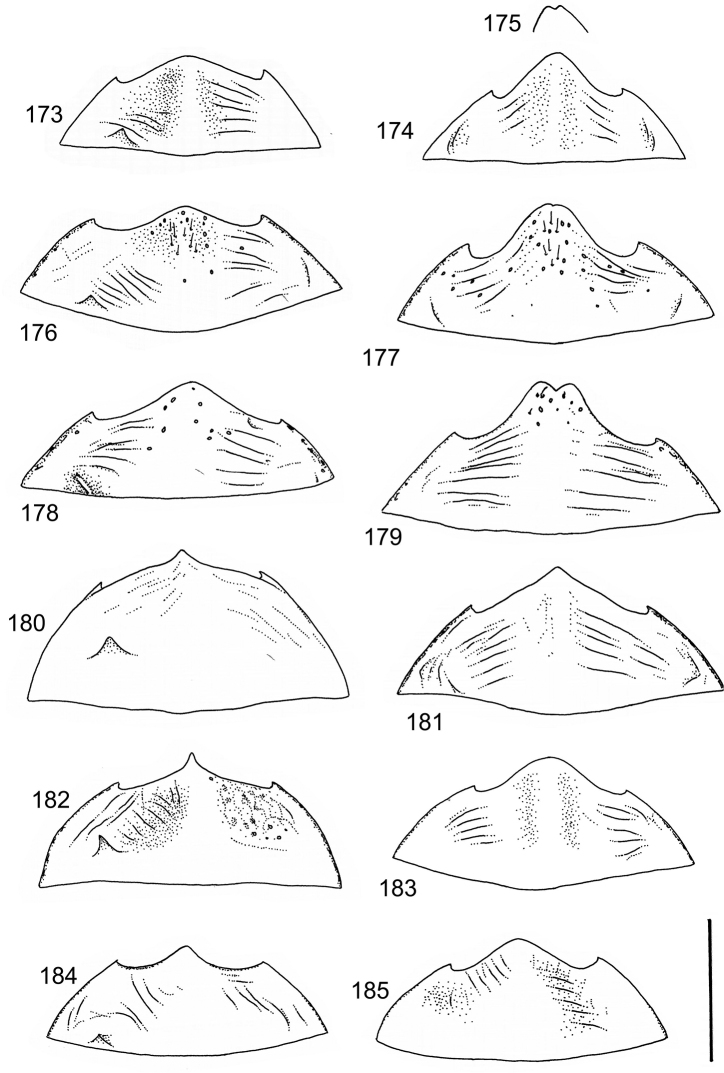
Apical ventrites **173** *Laccophilus secundus* male **174** female **175** same, but tip from other direction **176** *Laccophilus australis* male, and **177** female **178** *Laccophilus desintegratus* male, and **179** female **180** *Laccophilus luctuosus* male, and **181** female **182** *Laccophilus inornatus* male, and **183** female **184** *Laccophilus canthydroides* male, and **185** female. Scale bar 0.5 mm.

Ventral aspect: Blackish to dark ferrugineous, prothorax and apex of abdomen paler, ferruginous. Rather shiny, although finely microsculptured. Microsculpture in part reduced and missing. Almost impunctate. Prosternal process rather slender, apex slightly extended and pointed. Basal ventrites with fine, curved striae. Metacoxal plates with about 10 shallow and transversely located furrows. Apical ventrite assymmetric; on one side with a minute knob (Fig. 173).

Legs: Pro- and mesotarsus slightly enlarged, basally with suckers.

Male genitalia: Penis in lateral aspect almost straight, extreme apex strongly bent and formed as a small, short and truncate extension (Figs 354–355).

Female: Externally resembles male but apical ventrite lacks knob, almost symmetric (Figs 174–175). Pro- and mesotarsus slender.

##### Distribution.

Sudan, Ethiopia, Benin, Nigeria, Cameroon, Central African Republic, Gabon, Congo, Zaire, Uganda, Kenya, Tanzania, Zambia, Zimbabwe, Namibia, Botswana, South Africa (Fig. 573). Non-verified, country records are Angola (Peschet 1925), Ivory Coast (Guignot 1943) and Senegal (Legros 1972). Furthermore, additional, country records under the name *Laccophilus torquatus* are Guinea (Guignot 1956b), Malawi (Omer-Cooper 1958b), Chad (Bruneau de Miré and Legros 1963) and Swaziland (Bertrand and Legros 1967).

##### Collecting circumstances.

Label data indicate that the species has been collected in a slowly running stream. Moreover, the species is reported from a muddy pool with gravel bottom. Often recorded at light collection. Some information on ecology is available in Omer-Cooper (1958b), who briefly describes some sites where the species has been collected. Similar kind of information is available in Bilardo and Rocchi (1987, 1990, and 2013). Additionally, listed as a savannah-species in Bilardo and Rocchi (2008).

#### Laccophilus australis sp. n.

Taxon classificationAnimaliaColeopteraDytiscidae

http://zoobank.org/5B72FFFB-3890-4B21-971B-FAF5EA06946B

Figs 176–177 , 356 , 506–507 , 567

##### Type locality.

South Africa: Zululand, Mission Rock, St. Lucia.

##### Type material

(20 exs.). Holotype: male: “S. Afr., Zululand, St. Lucia, Mission Rock / 18.12. 1975; E-Y: 983 at black light leg. Endrödy-Younga” (TMSA; habitus in Fig. 506). – Paratypes: Same as holotype (5 exs. TMSA, 2 exs. MZH; habitus in Fig. 507); “S. Afr. E. Transvaal Hazyview 25.04S-31.07E / 3.4. 1990 E-y: 2778, UV light trap leg. Endrödy-Younga” (1 ex. TMSA); “S. Afr.: Kruger Nat Pk, Skukuza research ca 25.00S-31.35E / 19.2. 1995 E-Y: 3102, UV light & trap leg. Endrödy-Younga” (1 ex. TMSA); KwaZulu-Natal, Richards Bay, Umhlatuze Floodplain, *Papyrus* swamp, 7.6. 1985 Reavell” (4 exs. AMGS); “Natal Zululand, Mtuba-Tuba 23.9. 1947 JOC” (3 exs. AMGS). – Tanzania: “D.O. Africa Myambo 19.III. 14 leg. Methner” (1 ex. ZMHB); “Daressalaam II. 12” (1 ex. ZMHB). – Malawi: “Nyasaland Dally’s 18.12. 1946 R.H. Love B.M. 1948-309” (1 ex. BMNH).

##### Diagnosis.

*Laccophilus australis* is characterized by colour pattern of body-dorsal-aspect, by its double reticulation on head and elytra, large meshes of which have almost disappeared by reduction and by peculiar shaped penis apex; apex truncate with distinct lateral extension. Externally it resembles of *Laccophilus secundus* and in part also of *Laccophilus luctuosus* (a species placed in an own species group 15). Penis apex extension longer in *Laccophilus australis*, in comparsion with *Laccophilus secundus*. See also diagnosis of *Laccophilus luctuosus* (p. 236).

##### Description.

Body length 3.4–3.6 mm, width 1.9–2.0 mm. Dorsal, colour pattern of body exhibits some variation.

Head: Pale ferrugineous. Submat, finely and quite densely microsculptured. Reticulation double, but large meshes extensively, strongly reduced and hardly discernible. Almost impunctate; at eyes comparatively extensively with fine, irregular punctures. Areas of punctures extended towards middle of head-disc, forming a puncture row, however, medially very sparse.

Pronotum: Pale ferrugineous, basally in middle with a vague blackish to dark ferrugineous marking. At margins except basally in middle with fine, sparse and irregular punctures; otherwise pronotum impunctate. Submat, finely microsculptured; reticulation double. Large meshes only slightly more strongly developed than small meshes. Large meshes contain 2–5 small meshes.

Elytra: Blackish to blackish ferrugineous, with rather distinct but somewhat variable pale ferrugineous markings. Colour pattern consists of a slightly uneven, transverse, pale ferrugineous marking located close to elytral base. Post-medially with variable, longitudinal pale spots (Figs 506–507). Submat, finely and densely microsculptured; reticulation double but large meshes extensively very indistinct, in part absent. Discal row of punctures consists of fine, irregular punctures, discernible from base to apex. Besides discal row towards outer edge, punctures appear fine to very fine, sporadic, irregular and quite sparse; no distinct rows of punctures formed. Pre-apical, lateral, shallow furrow provided with some punctures and hairs.

Ventral aspect: Blackish to blackish ferrugineous, posteriorly on abdomen slightly paler; dark ferrugineous. Prothorax pale ferrugineous. Rather shiny to submat; finely microsculptured but reticulation partially reduced, absent. Ventrites with fine, slightly curved striae. Apical ventrite asymmetric, provided with a small, sharp knob on one side (Fig. 176). Almost impunctate; apical ventrite with some irregular, fine punctures. Metacoxal plates with some 8-9, transverse, shallow and in part reduced furrows. Prosternal process rather slender, posteriorly slightly extended, apically pointed.

Legs: Pro- and mesotarsus slightly extended, enlarged and provided with suckers.

Male genitalia: Penis larger than in *Laccophilus secundus*. In lateral aspect penis straight and extreme apex with a distinct lateral extension (Fig. 356).

Female: Apical ventrite symmetric, lacks lateral knob (Fig. 177). Pro-and mesotarsus slender.

##### Etymology.

The species name *australis* is a Latin adjective meaning “southern”. It refers to the location from where the new species was first detected, i.e. South Africa. Later on the new species was also recorded from more northern sites in Malawi and Tanzania.

##### Distribution.

Tanzania, Malawi, South Africa (Fig. 567).

##### Collecting circumstances.

Flight-capable; sampled at UV and black light collection. In Kwazulu Natal sampled in a *Papyrus* swamp.

### Species group 14 (*Laccophilus desintegratus* group)

**Diagnosis.** Small to medium sized species, length of body 3.4–3.8 mm, width 1.9–2.1 mm.

Shape of body oval; body dorsoventrally flattened. Elytra with extensive dark ferrugineous to blackish marking. Basally often with an irregular, transverse pale marking which is broken narrowly at suture. Posterior to middle with somewhat vague, longitudinal pale ferrugineous markings. Apically with vague pale area. Rarely pale markings on elytra reduced to some small, pale spots. (Figs 501–502). Microsculpture of body simple, of one kind. No fragments of large meshes detected.

Prosternal process rather slender, apex somewhat extended, apically pointed. Apical ventrite of male provided with asymmetrical knob on one side of ventrite (Fig. 178). Apical ventrite posteriorly excavate at both side, medial part posteriorly extended. Metacoxal plates lack stridulatory apparatus. Metacoxal process ends abruptly; lacks posterior extension (Fig. 6).

Paramere rather simple; apically somewhat enlarged (Fig. 358). Penis peculiar and different from all other African species; apex truncate and distinctly expanded on one side (Fig. 357).

**Species composition and distribution.** One species is recognized in this species group. The species is distributed in central parts of Africa, south of Sahara.

#### Laccophilus desintegratus

Taxon classificationAnimaliaColeopteraDytiscidae

Régimbart, 1895

Figs 178–179 , 357–358 , 501–502 , 571

Laccophilus desintegratus Régimbart 1895: 143 (original description, faunistics); Zimmermann 1920a:18 (catalogue, faunistics); Zimmermann 1926: 23 (faunistics); Guignot 1946c: 284, 285, 312 (description, faunistics); Guignot 1959a: 585, 587 (faunistics, description); Bilardo and Pederzani 1978: 119 (faunistics, description); Pederzani and Rocchi 1982: 72 (faunistics); Bilardo 1982a: 447 (given as *Laccophylus*, description, faunistics); Pederzani and Rocchi 1982: 78 (discussion); Bilardo and Rocchi 1990: 177 (faunistics, biology); Nilsson 2001: 242 (catalogue, faunistics); Bilardo and Rocchi 2002: 156, 161, 162, 174 (faunistics); Bilardo and Rocchi 2006: 130 (faunistics); Bilardo and Rocchi 2008: 211, 236 (faunistics, biology); Bilardo and Rocchi 2011: 194, 228 (faunistics, biology); Bilardo and Rocchi 2013: 141 (faunistics); Nilsson 2015: 211 (catalogue, faunistics).
Laccophilus gutticollis Régimbart 1895: 148 (original description, faunistics); Régimbart 1904: 66 (discussion); Zimmermann 1920a:19 (catalogue, faunistics); Guignot 1946c: 284 (given as Laccophilus desintegratus ab. gutticolis), 285 (description, discussion); Guignot 1959a: 578, 587 (description, discussion, faunistics); Bilardo and Pederzani 1978: 119 (faunistics, description); Pederzani and Rocchi 1982: 79 (given as Laccophilus desintegratus var. gutticolis, discussion); Bilardo and Rocchi 1990: 160, 177 (faunistics, biology); Bilardo and Rocchi 1999: 232 (given as Laccophilus desintegratus gutticolis; list, faunistics), 234 (given as Laccophilus desintegratus “variazione” gutticolis, faunistics); Nilsson 2001: 242 (catalogue, faunistics, list, synonymy); Bilardo and Rocchi 2011: 228 (given as Laccophilus desintegratus sub gutticolis; list, faunistics); Nilsson 2015: 211 (catalogue, faunistics, list, synonymy). **Confirmed synonym.**
Laccophilus sanguinosus Régimbart 1895: 148 (original description, faunistics); Zimmermann 1920a: 25 (catalogue, faunistics); Guignot 1946c: 284 (description, faunistics); Guignot 1959a: 585, 586 (description, faunistics); Nilsson 2001: 250 (catalogue, faunistics); Bilardo and Rocchi 2002: 174 (list, faunistics); Nilsson 2015: 217 (catalogue, faunistics). **New synonym.**

##### Type localities.

*Laccophilus desintegratus*: Zaire: Loango interior, Ht Quilou.

*Laccophilus gutticollis*: Gabon: Montagnes de Cristal.

*Laccophilus sanguinosus*: Gabon: Montagnes de Cristal.

##### Type material studied

(13 exs.). *Laccophilus desintegratus*: Lectotype (by present designation): male: “Loango int. Ht Quilou / male symbol / Cotype / *Laccophilus desintegratus* Rég. cotype” (MNHN). – Paralectotypes: “Loango int. Ht Quilou / female symbol / Cotype / *Laccophilus desintegratus* Rég. cotype” (1 ex. MNHN); “Loango interior Haut Quilou Mocquerys 1893 / Museum Paris coll. Maurice Régimbart 1908 / *desintegratus* Rég.” (3 exs. MNHN); “Loango int, Ht Quilou / Museum Paris coll. Maurice Régimbart 1908” (1 ex. MNHN).

*Laccophilus gutticollis*: Lectotype (by present designation): male: “Mts de Cristal Mocquerys / male symbol / Cotype / *Laccophilus gutticollis* Rég. Cotype” (MNHN). – Paralectotypes: “Mts de Cristal Mocquerys / male symbol / Cotype / *Laccophilus gutticollis* Rég. Cotype” (1 ex. MNHN); “Mts de Cristal Mocquerys / Museum Paris coll. Maurice Régimbart 1908 / *gutticollis* Rég.” (3 exs. MNHN); “Mts de Cristal Mocquerys / Museum Paris coll. Maurice Régimbart 1908” (1 ex. MNHN).

*Laccophilus sanguinosus*: Holotype: female: “Mts de Cristal Benito Gabon / Museum Paris coll. Maurice Régimbart 1908 / *sanguinosus* Rég.” (MNHN).

##### Additional material examined

(12 exs.). **Central African Republic**: “Zomea pres de Mbaiki 29-31.12. 1980 Onore” (2 exs. NHMB). – **Gabon**: “Bissok 9.8. 1991 Bilardo leg.” (1 ex. NHMW); “Ovan chutes Mingouli 13.8. 1992 Bilardo leg. / *Laccophilus desintegratus* Rég. det. Rocchi 1994” (1 ex. CSR); “Makokou-Riv. Ivindo, Chutes Kongou 16.8. 2002 Bilardo /*Laccophilus desintegratus* Régb. det. Rocchi 2003” (1 ex. CSR); “Makokou F. Ivindo 26.8. 1987 Bilardo / *Laccophilus desintegratus gutticollis* Rég. det. Bilardo 1988” (1 ex. MSNM); “Makokou 7.8. 1983 Bilardo” (1 ex. MSNM); “Res. Lopé-Okanda, milieu de savane 1.2. 1986 Bilardo / *Laccophilus desintegratus* Rég. det. Bilardo” (2 exs. MSNM); “Cap Esterias 31.7. 1973 Bilardo & Pederzani” (2 exs. NHMB; habitus in Fig. 502). – **Congo**: “Dimonika (Mayombe) 27.6. 1980 Onore” (1 ex. NHMB; habitus in Fig. 501).

##### Comments on synonymy.

Lectotypes and holotype of the three involved species have been examined and compared. Despite clear differences in dorsal colour pattern of body, the three taxa undoubtedly are conspecific. Ground plan of colour pattern is similar in the morphs. Shape of penis is identical. All three were originally introduced in the same publication so the rule of age-preference is irrelevant. We chose *Laccophilus desintegratus* as the valid name for the species.

##### Diagnosis.

Colour pattern of body variable in *Laccophilus desintegratus*. The species is especially characterized by its peculiar penis in combination with uniform, simple microsculpture on dorsal aspect of body. Penis in lateral aspect slightly sinuate; towards apex, penis distinctly expands into an extensive enlargement; apex broadly truncate.

##### Description.

Body length 3.4–3.8 mm, width 1.9–2.1 mm. Dorsal, colour pattern of body variable.

Head: Pale ferrugineous to ferrugineous, posteriorly, sometimes somewhat darker. Submat, finely and evenly microsculptured; reticulation simple, uniform and consists of small meshes. Impunctate, except at eyes; with fine, slightly irregular punctures; areas of punctures extend towards centre of head.

Pronotum: Pale ferrugineous, frontally and basally in middle with vague darker areas. In dark morph, pronotum almost totally dark, blackish ferrugineous with limited, small, pre-lateral, pale markings. Submat, finely and evenly microsculptured; reticulation simple, uniform; consists of small meshes. Impunctate, except anteriorly and laterally; with fine and somewhat irregular, sparse punctures. Medio-basally punctures very fine, hardly discernible.

Elytra: Dark ferrugineous, with distinctly delimited pale ferrugineous, slightly variable markings. Elytra at base often with transverse, pale area. In dark morph, basal, pale markings missing. With pale, longitudinal stripes posterior to middle. (Figs 501–502). Submat, finely and evenly microsculptured; reticulation simple, uniform, consists only of small meshes. Impunctate, except discal row; consists of fine, somewhat irregular punctures. Dorsolateral and lateral row indicated by a few fine, scattered punctures. Lateral pre-apical furrow fine, moderately pubescent.

Ventral aspect: Pale ferrugineous to ferrugineous, without distinct colour pattern. Rather shiny, although very finely microsculptured. Ventrites with fine, slightly curved striae. Almost impunctate. Prosternal process rather slender, apex somewhat extended, pointed. Metacoxal plates with some fine, transversely located, shallow furrows; furrows located to anterior half of plate. Apical ventrite on one side with a sharp knob (Fig. 178).

Legs: Pro- and mesotsarsus slightly enlarged, with fine suckers.

Male genitalia: Penis in lateral aspect slightly sinuate; towards apex it grows distinctly into an extensive enlargement; apex broadly truncate (Figs 357–358).

Female: Pro- and mesotarsus slender, not distinctly enlarged. Apical ventrite simple (Fig. 179).

##### Distribution.

Central African Republic, Gabon, Congo, Zaire (Fig. 571). Bilardo (1982a) gives Cameroon.

##### Collecting circumstances.

Often associated with forests (Bilardo and Rocchi 2008). Some additional information may be present in Bilardo and Rocchi (1990) where general information is given for collecting sites.

### Species group 15 (*Laccophilus luctuosus* group)

**Diagnosis**. Rather small to medium sized species; length of body 3.2–3.6 mm, width 1.7–2.0 mm.

Body shape oval; body dorsoventrally flattened. Recognized species with dark (dark ferrugineous to blackish) elytra, provided with distinct pale ferrugineous markings; a basal, transverse marking and posteriorly on elytra with variable, pale spots or longitudinally placed stripes (Figs 503–505). Pronotum, except for small, dark markings, and head almost entirely pale ferrugineous. Body with dorsal microsculpture double; reticulation divided into two size-classes out of which larger meshes in part reduced, fragmentary, and only in part discernible.

Prosternal process slender, posteriorly extended, apically pointed. Apical ventrite modified; posterior edge on both side of midline excavated and medial part forms a backwards extending process. Apical ventrite of male provided with asymmetrical knob on one side of ventrite (Fig. 180). Metacoxal plates lack stridulatory apparatus. Metacoxal process ends abruptly; lacks posterior extension (Fig. 6). No stridulation apparatus on metacoxal plates.

Paramere apically distinctly enlarged. Penis robust, simply slightly curved but not undulate, apically provided with strong process (Figs 359–360).

**Species composition and distribution.** One species occurring in Madagascar, is recognized in this species group.

#### Laccophilus luctuosus

Taxon classificationAnimaliaColeopteraDytiscidae

Sharp, 1882

Figs 180–181 , 359–360 , 503–505 , 572

Laccophilus luctuosus Sharp 1882: 307 (original description, faunistics); v. d. Branden 1885: 21 (catalogue, faunistics); Régimbart 1895: 145 (description, faunistics); Régimbart 1903: 14 (faunistics); Zimmermann 1919: 123 (faunistics); Zimmermann 1920a: 21 (catalogue, faunistics); Guignot 1959a: 533, 537, 538 (description, discussion, faunistics); Bertrand and Legros 1971: 244 (faunistics, biology); Rocchi 1991: 79, 86 (faunistics); Nilsson 2001: 246 (catalogue, faunistics); Nilsson 2015: 213 (catalogue, faunistics).

##### Type locality.

Madagascar.

##### Type material studied

(1 ex.): Lectotype (by present designation): male: “Type / Madagascar / Sharp Coll. 1905–313 / Type 614 *Laccophilus luctuosus”* (BMNH).

##### Additional material studied

(88). **Madagascar**: “Ampamoho nr Andilamena, 1200-1300 m a.s.l., 18-20.1. 1995 Dunay & Janák leg.” (4 exs. NMW, 1 ex. MZH); “5 km S Ampamoho nr Andilamena, 950-1000 m Dunay & Janak leg.” (1 ex. NMW); same data but “foret humide 18-20.1. 1995 lux” (2 exs. ZMHB); “Ambatombe, near Andilamena 900 m a.s.l., 17.1. 1995 Dunay & Janák leg.” (17 exs. NMW, 4 exs. MZH; habitus in 503-504); “E Sakahara, Manindray 30.1. 1995 700-800 m Dunay & Janak leg.” (1 ex. NMW); “Manindray, W Sakahara 700-800 m asl, 30.1. 1995 Dunay & Janak leg.” (2 exs. NMW); “Tanandava, lumièrè 1963-1964 Schmitz” (2 exs. MRAC, 1 ex. MZH); “Betsiboka Bas, Loc. Ambohimanatrika, Kamoro Riv., 47°10'06"E/16°28'55"S, alt. 40 m, 1.4. 1993” (3 exs. NHRS); “Betsiboka Bas. Loc. Ambalambongo, afl. de Betsiboka Riv. 47°00'30"E/16°48'00"S, 30.3. 1993”(2 exs. NHRS); “Betsiboka Bas. Loc. Fiadanana Ikopa Riv. 46°56'58"E/18°10'03"S, Alt. 975 m 18.4. 1991” (1 ex. NMW; habitus in Fig. 505); “Bas. Cotier Fort Dauphin / Manampanihy Bas 4.2. 1996 leg. Gibon” (1 ex. NHRS); “Antserarana distr., Sambirana Riv., Marovato vill. 5-12.12. 2001 Horak leg.” (6 exs. NHRS); same data but “J. Rolcik leg.” (6 exs. NMPC, 2 exs. MZH); “Toli, NW Ft Dauphin, rice paddies, N: -24.824; E46.866: 34.44 m 19.5. 2006 Bergsten leg. / BMNH(E) <794169> DNA voucher /*Laccophilus luctuosus* Shp. det. J. Bergsten” (1 ex. NHRS); “Ambilobe; Anjiabe Ambony Antsabe. ¾ moon, dry, many water beetles, near camp alt. 49 m, N -13.6518, E 48.7267, 21.11. 2004 Balke & al leg / BMNH(E) <794162; 794163; 794164> DNA voucher / *Laccophilus luctuosus* Shp. det. J. Bergsten” (3 exs. NHRS); “Toa, Maroantsetra: Maroantsetra, light trap N: -15.424, E: 49.738. 12 m, 20.12. 2006 Isambert et al. leg. / BMNH(E) <831014/831015> DNA voucher MSL 402:D11/MSL 402:D12 / *Laccophilus luctuosus* Shp. det. J. Bergsten” (2 exs. NHRS); “Toli, Morondava, les Baobabs Amoureux / BMNH(E) <794165> DNA voucher / *Laccophilus luctuosus* Shp. det. J. Bergsten” (1 ex. NHRS); “Toliara, Menabe, Menabe RS, S19.92773, E045.52253, 102 m.a.o. 10.12. 2009, Water net. field, Bergsten et al. NHRS-JLKB 000000488” (1 ex. NHRS); “Mahajanga Boeny Ankarafantsika NP, N-16.30341, E046.81073, 74 m.a.o. 29.11. 2009, 22 W black light, field Bergsten et al” (2 exs. NHRS); same data, add “NHRS-JLKB 000000490” (1 ex. NHRS); same data, add “NHRS-JLKB 000000485” (1 ex. NHRS); same data but “S16.31418, E046.81731, 30.11. 2009, handpicking, field / NHRS.JLKB 000000489” (1 ex. NMHR); same data but “S16.30270, E046.80996, 75 m.a.o., 30.11. 2009, 22 W black light, field / NHRS-JLKB 000000481” (1 ex. NHRS); same data “S16.30271, E046.80995, 75 m.a.o., 29.11. 2009 water net, field / NHRS-JLKB 000000487” (1 ex. NHRS); “Mahajanga Melaky, Tsingy de Bemaraha NP, S18.75724, E044.71239, 72 m.a.o. 17.12. 2009, waternet, fields, Bergsten et al./000000478” (1 ex, NHRS); same data, but “S18.75797, E044.71289, 81 m.a.o. 17.12. 2009, 22W black-light, field / NHRS-JLKB 000000482” (1 ex. NHRS); “Mahajanga Melaky btw. Morafenobe-Ambohijanahary S18.19091, E045.19986, 290 m.a.o. 19.12. 2009 Water net, field, Bergsten et al” (6 exs. NHRS); same data, add “NHRS-JLKB 000000483” (1 ex. NHRS); “Majunga Prov., 25 km SW Ambalanjankomby 3-11.11. 1962 Cashatt” (1 ex. USNM); “Madagascar Ambanja, R. Sambirano 29.11. 1952 E.S. Brown / Slow flowing river, sandy bottom, little vegetation (with *Chara*) / Brit. Mus. 1953-146 / *Laccophilus luctuosus* Shp. J. Balfour-Browne det. III. 1953” (1 ex. BMNH); “Sambirano, Ambodidimaka, Ambanja, 25 km SE,15-16.12. 2002, Ivo Jenia” (1 ex. NHMB); “Mad. E, Sambava 0-20 m Janak & Moravec lgt / 3.3. 1996 Riv. Anovona env. lux, rizières, bord de la foret” (2 exs. NMPC); “Mad. B, d’Antongil / Régimbart 1908” (1 ex. NHMB); “Maroansetra 10. 1936” (1 ex. MNHN); “Antakotako 2. 1936” (1 ex. MNHN).

##### Diagnosis.

A characteristic species which is confined to Madagascar. Male is easily identified by the combination of distinct, somewhat variable elytral colour pattern in combination with peculiar appearance of penis; robust, curved and not twisted; apex with obtuse extension.

##### Description.

Body length 3.2–3.6 mm, width 1.7–2.0 mm. Dorsal colour pattern rather distinct, somewhat variable (Figs 503–505).

Head: Pale ferrugineous, posteriorly at pronotum narrowly dark ferrugineous. Slightly mat, finely microsculptured. Reticulation in part double but very indistinctly so; in part of head surface reticulation appears as simple. Punctation very fine and sparse, partially absent.

Pronotum: Pale ferrugineous, mediobasally with blackish marking, and anteriorly with dark ferrugineous marking. Rather shiny. Fine microsculpture in part absent. Reticulation double; large meshes contain 3–6 fine meshes. Punctation fine and irregular.

Elytra: Dark ferrugineous, at base with transverse, pale ferrugineous marking (broken by dark suture). Rarely, pale area enlarged. Additionally, posterior to middle and apically with some vague, slightly variable, paler markings (Figs 503–505). Finely microsculptured and in part submat. Reticulation double, but in part kinds of reticulation difficult to distinguish. When discernible, large meshes may contain 2-6 fine meshes. Punctures irregularly distributed, fine to very fine and in part absent.

Ventral aspect: Dark ferrugineous to ferrugineous; prothorax and abdomen in part paler: ferrugineous to pale ferrugineous. Rather shiny although with very fine, in part absent microsculpture and some curved striae. Almost impunctate. Metacoxal plates with a few, indistinct, transversely located furrows. Prosternal process slender and pointed. Apical ventrite asymmetric; on one side with a sharp knob (Fig. 180).

Legs: Pro- and mesotarsus slightly enlarged, provided with fine suckers.

Male genitalia: Penis quite robust and not twisted; in lateral aspect from base to apex curved; apically with an obtuse hook/extension (Figs 359–360).

Female: Pro- and mesotarsus slender. Apical ventrite lacks knob (Fig. 181).

##### Distribution.

Madagascar (Fig. 572). Endemic species of Madagascar.

##### Collecting circumstances.

Almost unknown. Collected at light.

### Species group 16 (*Laccophilus leonensis* group)

**Diagnosis.** Small species; length of body 2.8–3.7 mm, width 1.5–2.0 mm.

Body shape oblong to oval; body dorsoventrally flattened. Elytra with variable colour pattern; from one-coloured dark to one-coloured pale, often provided with pale markings especially at base of elytra (Figs 511, 518, 520, 523). Body microsculpture double, of two different kinds; larger meshes sometimes in part reduced.

Prosternal process slender, extended and apically pointed. Male apical ventrite modified; posterior end of ventrite on both side of midline excavated and medial part extended posteriorly. Ventrite provided with an asymmetric knob on one side (Fig. 194). Fine, in slight curvature placed, dense striae form a delicate stridulation apparatus on metacoxal plates; feature exhibited by both sexes. Metacoxal process not extended posteriorly (Fig. 6).

Paramere apically strongly enlarged (Fig. 366). Penis ground-plan similar in all species; relatively robust and slightly curved. Apex truncate and often sharply edged (Figs 364, 371).

**Species composition and distribution.** 11 species are recognized in this species group. Distribution covers whole Africa south of Sahara and Madagascar. Determination is regarded critical and difficult to conduct – both external appearance and male genitalia should always be considered.

#### Key to species (males)

**Table:** 

1	Dark coloured species; head and pronotum as dark as elytra or slightly paler; black to blackish ferrugineous; elytral pale markings restricted to minor pale spots (Figs 517–518)	**2**
–	Uni- or two-coloured species; head and pronotum mainly pale ferrugineous to ferrugineous, elytra either dark or of same colour as pronotum and head; elytra unicoloured or with variable pale colour pattern (Figs 511, 522)	**3**
2	Large species (body length 3.1–3.5 mm); penis apex almost smooth, lacks distinct processes (Fig. 367)	***Laccophilus melas*** (p. 248)
–	Small species (body length 2.8–3.0); penis provided with apical processes (Fig. 366)	***Laccophilus villiersi*** (p. 247)
3	Elytra unicoloured or with vague pale, humeral spot and in one species often with a second vague spot basally, close to suture (Figs 516, 513)	**4**
–	Elytra with pale spots or pale marking transversely located at base of elytra (rarely spots reduced) (Figs 519, 523)	**8**
4	Distinctly bicoloured species (head and pronotum pale; elytra dark); elytra lack pale markings (Figs 515–516)	**5**
–	One-coloured or almost one-coloured species (head, pronotum and elytra equally pale or elytra slightly darker than head and pronotum); elytra often with vague pale spots in humeral region (Fig. 514)	**6**
5	Apex of penis distinctly wrinkled (Fig. 364)	***Laccophilus eboris*** (p. 244)
–	Apex of penis almost smooth (Fig. 365)	***Laccophilus leonensis*** (p. 246)
6	Inner anterior corner of penis apex blunt (Fig. 363)	***Laccophilus minimus*** (p. 243)
–	Inner anterior corner of penis apex sharp (Fig. 361)	**7**
7	Penis apex truncate; external process of penis apex moderately extended (Fig. 362); elytral base and humeral region often with a vague pale spot (Fig. 513)	***Laccophilus canthydroides*** (p. 242)
–	Penis apex less truncate; external corner of penis apex distinctly extended (Fig. 361); elytra generally one-coloured pale ferrugineous to ferrugineous (Figs 510–511)	***Laccophilus inornatus*** (p. 238)
8	Elytra with basal, narrow, transverse pale marking; narrowly broken at suture (Fig. 523); elytral ground-colour blackish brown to blackish ferrugineous; penis apex moderately modified (outline almost without extensions) (Figs 371–372)	***Laccophilus flavopictus*** (p. 254)
–	Elytra basally with row of pale, often vague, patches (Figs 519, 522); elytral ground-colour dark ferrugineous to ferrugineous; penis apex distinctly modified (outline with angled extension) (Fig. 370)	**9**
9	Inner anterior corner of penis apex distinct and sharp; apex wrinkled (Fig. 370)	***Laccophilus garambanus*** (p. 253)
–	Inner anterior corner of penis moderate; apex almost smooth (Fig. 369)	**10**
10	Main colour of elytra dark ferrugineous; anterior outline of penis apex almost straight (Fig. 368) (Mainland Africa)	***Laccophilus livingstoni*** (p. 250)
–	Main colour of elytra paler, ferrugineous; anterior outline of penis apex curved (apex appears expanded) (Fig. 369) (Madagascar)	***Laccophilus insularum*** (p. 251)

#### Laccophilus inornatus

Taxon classificationAnimaliaColeopteraDytiscidae

Zimmermann, 1926

Figs 182–183 , 361 , 510–511 , 574

Laccophilus inornatus Zimmermann 1926: 24 (original description, faunistics); Gschwendtner 1935b: 373 (faunistics); Guignot 1943: 99 (faunistics); Guignot 1946c: 284, 312, 315 (discussion); Guignot 1953b: 237 (discussion); Omer-Cooper 1953: 24 (discussion); Guignot 1955b: 1096 (faunistics); Guignot 1956b: 220, 221 (description, discussion); Guignot 1959a: 579, 581 (description, faunistics); Guignot 1959d: 163 (faunistics, discussion); Guignot 1961b: 238 (faunistics, discussion); Bruneau de Miré and Legros 1963: 874, 888 (faunistics); Omer-Cooper 1967: 60 (discussion, *Laccophilus livingstoni* O-C. = *Laccophilus inornatus* Zimm.); Bilardo and Pederzani 1978: 119 (faunistics, description); Medler 1980: 155 (faunistics, list); Forge 1981: 500, 501 (description, faunistics); Bilardo 1982a: 447 (description, faunistics, given as *Laccophylus*); Bilardo and Rocchi 1990: 162, 177 (faunistics, biology); Franciscolo and Sanfilippo 1990: 146 (faunistics, description); Nilsson and Persson 1993: 81, 94 (faunistics, biology); Nilsson et al. 1995: 505 (faunistics); Rocchi 2000: 24 (faunistics); Nilsson 2001: 245 (catalogue, faunistics); Bilardo and Rocchi 2002: 164 (discussion, faunistics); Reintjes 2004: 67 (faunistics); van Vondel 2005: 131 (faunistics, biology); Nilsson 2015: 212 (catalogue, faunistics). [Comment: Many of the listed references are to be considered uncertain due to extensive species-confusion.]

##### Type locality.

Tanzania: Usagara.

##### Type material studied

(3 exs.). Lectotype (by present designation): male: “Usagara / Typus / *Laccophilus inornatus* sp. n. Type det. A. Zimmermann / *inornatus* Zimm. sp. n.” (ZMHB). – Paralectotypes: “Daressal. / Type / Samml. A. Zimmermann / Paratypus” (1 ex. ZMSC); “D.O. Afrika / Type / Samml. A. Zimmermann / Paratypus” (1 ex. ZMSC; habitus in Fig. 510).

##### Additional material studied

(283 exs.). **Gambia**: “Abuko Nat. Res., at light at the bamboo pool 18.30–20.30 18.11. 1977 UTM 28PCK2181 / Cederholm & al leg. / *Laccophilus inornatus* Zimm. Brancucci det.” (1 ex. MZLU); “Central R. Div. Lower Fulladu, Sapo Agric. St. 27.8. 1997, MV light, Woodcock” (1 ex. NHMB); “Bathurst jan. 1968 Palm /*Laccophilus inornatus* Zimm. Persson det.” (5 exs. MZLU); “Tanji R, 3 km SW Brufut, at light 19-21.00, 28.2. 1977 Cederholm & al.” (1 ex. NHMB). – **Guinea**: “Sérédou, lux 4.4. 1975 Zott” (26 exs. ZMHB, 5 exs. MZH; habitus in Fig. 511); same data but ”4.5. 1975” (16 exs. ZMHB); same data but “5.4.1975” (1 ex. MZH); same data but “7-8.4. 1975” (13 exs. ZMHB, 1 ex. MZH). – **Liberia**: “Suakoko 10.3. 1953 light trap Blickenstaff” (1 ex. USNM); “Suakoko 14.3. 1952 / 6-9 pm light trap Blickenstaff” (1 ex. USNM). – **Ivory Coast**: “Comoé Nat. P., N8,5°, W3,5° Reintjes leg. / 11.1. 1999” (3 exs. NHMW); same but “15.4. 1999” (1 ex. NHMW); “Bingerville 8. 1963 J. Decelle” (1 ex. MRAC, 1 ex. MZH); same but “12. 1961” (1 ex. MRAC); same but “9. 1963” (1 ex. MRAC); “Danané 26.7. 1966 Thys & Verheyen” (1 ex. MRAC); “Divo 28.11. 1963 J. Decelle” (3 exs. MRAC, 1 ex. MZH); same but “4.12. 1962” (1 ex. MRAC); “Toumodi / 12. 1930-IV. 1931 Alluaud & Chappuis / *Laccophilus inornatus* Zimm. det. Gschwendtner” (2 exs. MNHN, 1 ex. OLML); “Touba, à la lumière 4. 2002 Moretto / *Laccophilus inornatus* Zimm. det. Rocchi 2002” (3 exs. CSR). – **Ghana**: “Ashanti Reg. Kwadaso, agric. st. 6°42'N, 1°39'W / light trap 26.2. 1969 Endrödy-Younga” (4 exs. TMSA, 1 ex. MZH); “Northern Region Damongo Game Res., 9°04'N, 1°48’ / 12.11. 1970” (1 ex. TMSA); “N.R., Damongo Mole game res. 220 m, 9°04'N, 1°48’ S. Endrödy-Younga / on light 12.8. 1971” (1 ex. OLML); “Ghana Ashanti Kumasi, 330 m N 6.43 – W 1.36 / 15.9. 1967 at light, Endrödy-Younga” (1 ex. TMSA); “Kumasi 12.5. 67 Endrödy-Younga / *Laccophilus inornatus* Zimm. det. Wewalka 76” (18 exs. MHNG); same data but ”16.6. 67” (10 exs. MHNG); same data but ”18.5. 67” (13 exs. MHNG). – **Benin**: “Dep. du Zou, commune de Zogbodomè 29.1.2006 Goergen / Lokoli Forest 07°03'N/02°15'E, 17 m a.s.l. light trap” (7 exs. MZH); “Dep. du Zou, commune de Zogbodomè, Lokoli (forest) Hlanzoun riv. 6.2.2006, Goergen, Komarek & Hounguè / 07°30'N, 02°15'E muddy stream” (1 ex. MZH); “Dep. Atlantique, Allada, Glotomè (village) 31.1. 2006 Goergen, Komarek & Hounguè / 06°41'06"N, 02°02'36"E, 17 m a.s.l., slowly running stream” (1 ex. MZH); “Dep. Mono, Comé, Sè (village) 2.2.2006, Goergen, Komarek & Hounguè / 06°30'14,5"N, 01°49'49,2"E, ca. 20 m a.s.l. pond with muddy ground” (1 ex. MZH); “Dep. Littoral, Toho near Pahou (village) 2.2. 2006, Goergen, Komarek & Hounguè / 06°23'11,3"N, 02°12'30,2"E lake with rich riparian vegetation” (2 exs. MZH); “Dep. Littoral Cotonou City, pond, 8.2. 2006, Goergen, Komarek & Hounguè” (1 ex. MZH); “Dep. Atlantique Toffo, near Toffo (village) 1.2. 2006, Goergen, Komarek & Hounguè / 06°48'51,8"N, 02°07'38,6"E, 25 m a.s.l. muddy stream” (1 ex. MZH). – **Nigeria**: “NW St. Badeggi rice field 8-9.8. 1973 Linnavuori” (54 exs. MZH); “NW St. Yelwa 23.7. 1973 Linnavuori” (1 ex. MZH); “W St. Ife 7-8.7. 1973 Linnavuori” (1 ex. MZH); “EC St. Norcap nr Abakaliki 29.6. 1973 Linnavuori” (2 exs. MZH); same, but “ad lucem” (1 ex. MZH); “Ibadan ca. Jan. – Juni 1954 Stenholt Clausen” (9 exs. ZMUC, 1 ex. MZH); “Ibadan, light trap: campus, Dec. 2003 Goergen” (1 ex. NHMW); “Ibadan at light 26.9. 1956 / G.H. Caswell” (1 ex. BMNH); same data but “27.11. 1955” (1 ex. BMNH); “Kontagora stream 3.IV. 1963” (4 exs. AMGS); “River crossing rd to Erugo about 79 miles from Makurdi 24.4. 1963 JOC.” (4 exs. AMGS); “Marsh, Katsina-Daurra rd. 6.IV. 1963” (1 ex. AMGS); “Stream 64 mi. From Bida on Jebba rd. 12.IV. 1963 JOC.” (1 ex. AMGS); “Stream crossing Kaduna rd. Nr. Zaria 4.IV. 1963 JOC.” (2 exs. AMGS); “Zaria, à la lumière 1969 Roberts H.”(4 exs. MRAC, 2 exs. MZH); “River, Kaduna rd. 13,5 mi. From Jos 13.IV. 1963” (3 exs. AMGS); “R. Kaduna 4,5 mi. from Jos 13. IV. 1963 JOC.” (2 exs. AMGS); “Pools in dry stream bed, Kontagora 5.IV. 1963” (1 ex. AMGS); “Stream, reservoir Yom, Jos Plateau 11.IV. 1963 JOC.” (1 ex. AMGS). – **Sudan**: “R. Malia 30,57E 40,39N 29.1. 1954 JJOC.“ (1 ex. AMGS); “Minkammon 31,31E 6,2N 16-17.1. 1954 JJOC.” (1 ex. AMGS); “Equatoria Nimule 11-13.3. 1963 Linnavuori” (1 ex. MZH). – **Cameroon**: “Yaounde, Bor to Kosti by boat 3-14.3. 1978 Perkins” (3 exs. USNM, 1 ex. MZH); “Minkama 15.4. 1970” (3 exs. NHMB). – **Central African Republic**: “Bozo 21.5. 1981 / Degallier” (10 exs. NHMB); same but “11. 1981” (2 exs. NHMB); same but “12. 1981” (2 exs. MZH). – **Kenya**: “Shimba Hills 10.12. 1989 Jäch leg.” (1 ex. NHMW). – **Tanzania**: “Dar es Salaam Tendangiri (?) Exp. / *Laccophilus inornatus* Rég. det. M. Brancucci 81” (1 ex. ZMHB); “Daressal.” (1 ex. ZMHB). – **South Africa**: “E Transvaal Hazyview 25.04S-31.07E / 3.4. 1990 E-Y: 2778 UV light trap leg. Endrödy-Younga” (2 exs. TMSA); “Natal, Waterton Timber Co. 3. 1985 N-28.20.5, E32.14, at light Atkinson” (1 ex. NHMB); “ECPr., 6 km S of Port of St Johns, outside Silaka Nature Reserve, pond, S31°38.862, E29°30.551, alt.90 m, 26.1. 2005 Bergsten” (1 ex. NHRS).

##### Specimen with unclear labelling.

**Egypt**: “Kairo / Trägårdh / *Laccophilus restrictus* var.?” (1 ex. NHRS).

##### Comments on synonymy.

Examination of type material reveals that *Laccophilus inornatus* is not synonymous with *Laccophilus livingstoni* O-C., as stated by Omer-Cooper (1967).

##### Diagnosis.

Externally *Laccophilus inornatus* resembles much of *Laccophilus canthydroides*. The two species can be separated by examination of anatomical details exhibited by penis apex; external corner of penis apex distinctly extended in *Laccophilus inornatus* while less so in *Laccophilus canthydroides*. In general, *Laccophilus canthydroides* has small, pale spots on elytral base while *Laccophilus inornatus* lacks spots on elytral base or has in humeral region, a vague, pale spot.

##### Description.

Body Length 2.9–3.4 mm, width 1.5–1.9 mm. Dorsal colour pattern of body absent or vague; sometimes elytra with slightly paler base (Figs 505–506). From Ghana I have seen some totally black specimens – possible artefacts?

Head: Pale ferrugineous; posteriorly often, close to eyes slightly darker. Finely microsculptured. Reticulation almost simple; difficult to separate two kinds of meshes. Reticulation in part slightly irregular; shape of meshes variable. At eyes and in a narrow medial area between eyes with fine, irregular punctures.

Pronotum: Pale ferrugineous, frontally and medially at base often with vague, a little darker markings. Finely microsculptured; in part double reticulation discernible. A part of large meshes do not contain small meshes. Difference between large and small meshes indistinct or almost absent. Punctation rather indistinct. Fine, irregular punctures discernible at least at base and laterally.

Elytra: Ferrugineous to dark ferrugineous. Sometimes elytra slightly paler in anterior half than posteriorly but change of colour is gradual and vague (Figs 510–511). Rather shiny, although finely microsculptured. Reticulation double, but posteriorly in particular, size-classes indistinct and difficult to discern. Anteriorly two kinds of meshes in general present; larger meshes which contain two to six fine meshes. Number of fine meshes sometimes difficult to count. Fine, rather indistinct and scattered puncture discernible. An irregular row of punctures laterally discernible (in posterior half of elytron distinct).

Ventral aspect: Pale ferrugineous, abdomen, particularly posteriorly somewhat darker, ferrugineous to dark ferrugineous. Rather shiny, finely microsculptured except on abdomen where microsculpture is indistinct. Abdomen, basally with sparse, curved striae. Metacoxal plates with a number of mostly transversely located shallow furrows. Almost impunctate. Apex of prosternal process slender and pointed. Apical ventrite with a small knob on one side (Fig. 182).

Legs: Pro- and mesotarsus rather slender, provided with fine suckers.

Male genitalia: External apex of penis extended and sharp (Fig. 361).

Female: Apical ventrite lacks knob (Fig. 183). Pro- and mesotarsus slender.

##### Distribution.

Gambia, Guinea, Liberia, Ivory Coast, Ghana, Benin, Nigeria, Sudan, Cameroon, Central African Republic, Kenya, Tanzania, South Africa (Fig. 574). Only verified records are mapped. One specimen labelled Kairo (Egypt) is considered uncertain.

##### Collecting circumstances.

Label data contains some information of the ecology of *Laccophilus inornatus*. Accordingly, it has been collected both in running waters (“muddy stream, slow running stream”) and standing waters (“muddy pond, pools in dry stream bed, in lake with rich riparian vegetation”). Often captured at light collection.

#### Laccophilus canthydroides

Taxon classificationAnimaliaColeopteraDytiscidae

Omer-Cooper, 1957

Figs 184–185 , 362 , 512 , 513 , 575

Laccophilus canthydroides Omer-Cooper 1957: 13, 90 (original description, faunistics); Omer-Cooper 1965: 76, 81 (description, faunistics); Nilsson 2001: 241 (catalogue, faunistics); Nilsson 2015: 210 (catalogue, faunistics).

##### Type locality.

South Africa: Transvaal, Barberton, (trib. of Koop River).

##### Type material studied

(2 exs.). Holotype: female: “Type / Transvaal Trib. of Koop R. nr Barberton / Brit. Mus. 1957-660 / *Laccophilus* ? sp. n. J. Balfour-Browwne det. / *Laccophilus canthydroides”* (BMNH; habitus in Fig. 512). – Paratype: female: “Paratype / Transvaal, Trib. of Koop R. nr. Barberton 1.12. 1948 / *Laccophilus canthydroides* / *Laccophilus* ? sp. n. J. Balfour-Browne det.” (1 ex. AMGS; according to the original description to be deposited in TMSA).

##### Additional material studied

(56 exs.). **Ethiopia**: “Bahar Dar 8.10. 1968 Harde” (2 exs. NHMB). – **Cameroon**: “Emana Obala 16.5. 1970” (1 ex. NHMB). – **Zaire**: “Parc National Garamba 10.3. 1952 De Saeger” (3 exs. MRAC, 2 exs. NHMB, 1 ex. MZH); “Elisabethville, à la lumière 1957-1958 Seydel” (1 ex. AMGS, 10 exs. MRAC, 2 exs. MZH; habitus in Fig. 513); same but “1. 1956-1. 1957” (1 ex. MRAC); same but “11. 1951-2. 1952” (1 ex. MNHN); same but “11. 1950-6. 1951” (1 ex. MNHN); same but “1953-1955” (1 ex. AMGS, 2 exs. NHMB); “Elisabethville 2. 1940 H.J. Brédo” (2 exs. OLML); “Elisabethville, lumière 11. 1951-2. 1952 Seydel” (2 exs. IRSNB); “Kivu, Kavimvira (Uvira), à la lumière 1. 1956 Marlier” (1 ex. NHMB). – **Kenya**: “Meru distr., Matiri (Mitunguu) mt. 800, 18.10. 1982 Mourglia” (3 exs. NHMB); same but “8.11. 1983” (1 ex. NHMB). – **Tanzania**: “Tang. Terr. Ukerewe 7. 1933” (3 exs. OLML); “Mufundi Mafinga m. 1900 21.11.-4.12. 1989 leg. R. Mouglia” (1 ex. CSR); “NP Udzungwa, Ifakara, at street from entrance, S08.02.55, E36.43.59, 251 m, trunk under bark 18.7. 2004 Sprecher” (1 ex. NHMB); “Zanzibar Mangapwani rd 13.9. 1955 JOC.”(6 exs. AMGS). – **Zambia**: “Mbesuma Ranch (Isoka) 9-10.12. 2004 Werner & Smrz” (5 exs. NHRS); “Luapula Pr., Lake Bangweulu, Chilubi 11.2. 1982 Selander” (1 ex. MZH). – **Malawi**: “Nkhata Bay env. rainforest 1.1. 2002 Kantner” (1 ex. NHMB). – **South Africa**: “Wellington, Witte River 1500 ft.” (1 ex. SAMC).

##### Diagnosis.

*Laccophilus canthydroides* seems to be closest related to *Laccophilus inornatus* – for separation of the two species, vide diagnosis of *Laccophilus inornatus* (p. 241).

##### Description.

Body length 3.2–3.6 mm, width 1.8–1.9 mm. Head and pronotum extensively pale while elytra often somewhat darker. Often with two, pale spots on base of elytra. Sometimes pale spots absent or reduced to one humeral spot (Figs 512–513).

Head: Pale ferrugineous, posteriorly slightly darker but darker area anteriorly with vague delimitation. Submat, finely microsculptured; reticulation double but large meshes only slightly stronger than fine meshes. By large, 3–7 fine meshes included in one large mesh. At eyes with irregular cluster of fine, rather indistinct punctures.

Pronotum: Pale ferrugineous, basally in middle often with rather narrow darker area. Rather shiny, although finely microsculptured; reticulation double but large meshes only slightly stronger than fine meshes. By large, 3–7 fine meshes included in one large mesh. Laterally and frontally on pronotum with scattered, fine punctures.

Elytra: Dark ferrugineous to ferrugineous, at humeral region and posteriorly often with vague, small, pale spots (Figs 512–513). Rather shiny, although finely microsculptured; reticulation double but in part (laterally and posteriorly) division of meshes in two size-classes indistinct. In general large meshes (when discernible), contain 2–5 fine meshes. Impunctate, except for discal, dorsolateral and lateral rows of fine, somewhat irregular punctures.

Ventral aspect: Pale ferrugineous, except ventrites often somewhat darker; ferrugineous. Rather shiny, although with fine reticulation (densely located fine lines). Metacoxal plates with some irregular and indistinct, laterally projecting striae. Basal, abdominal ventrites laterally with a few, distinct striae. Almost impunctate. Apex of prosternal process slender and pointed. Apical ventrite on one side with a minute knob (Fig. 184).

Legs: Pale ferrugineous. Pro- and mesotarsus slightly enlarged, provided with suckers.

Male genitalia: External, extreme, apex of penis not distinctly extended, nor sharp (Fig. 362).

Female: Pro- and mesotarsus slender. Apical ventrite (Fig. 185).

##### Distribution.

Ethiopia, Cameroon, Zaire, Kenya, Tanzania, Zambia, Malawi, South Africa (Fig. 575).

##### Collecting circumstances.

Largely unknown. Collected in a light trap.

#### Laccophilus minimus sp. n.

Taxon classificationAnimaliaColeopteraDytiscidae

http://zoobank.org/27CD0E8C-69F5-42AB-B024-26E219BCA5B4

**Figures 186–199.**
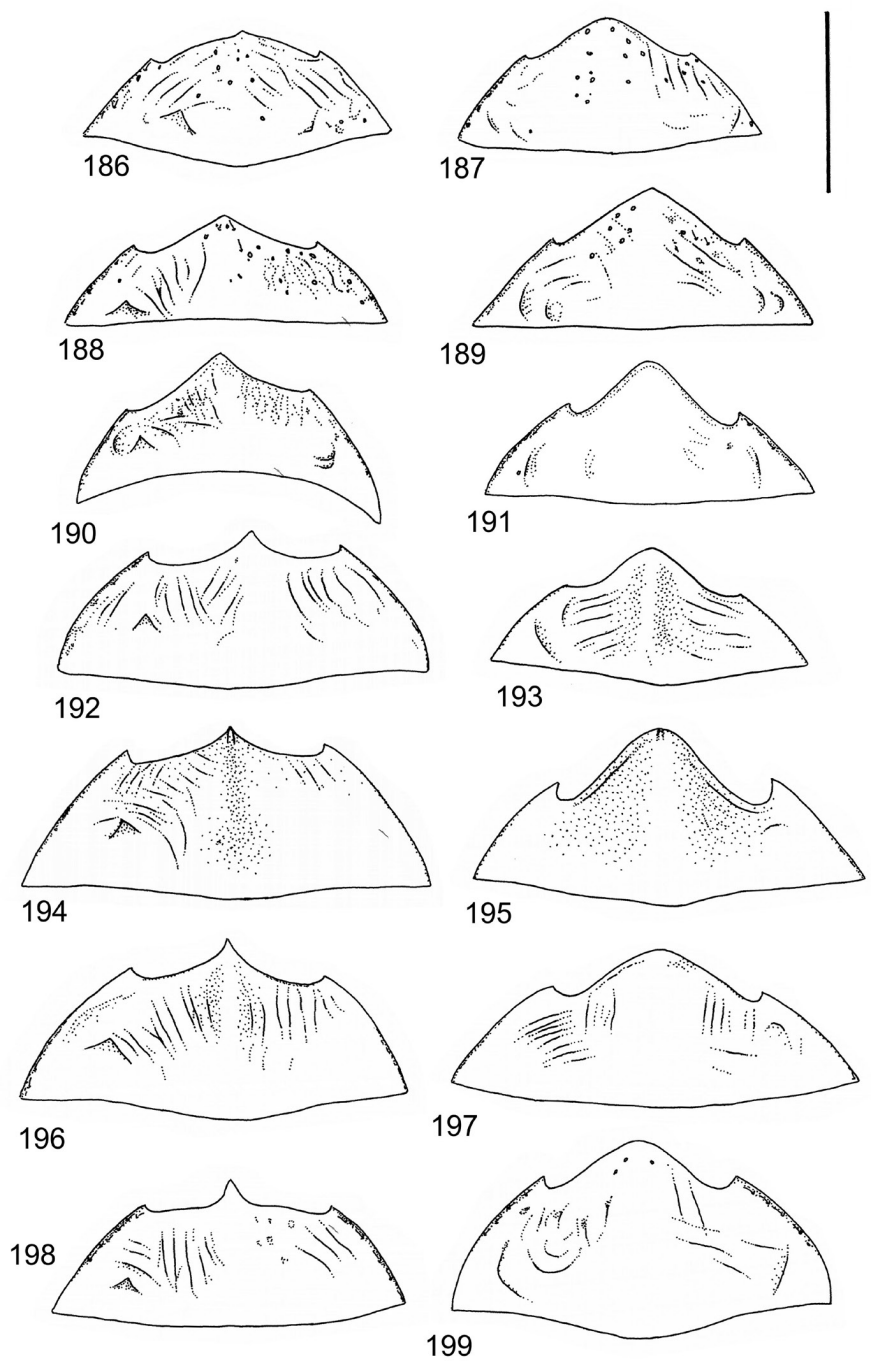
Apical ventrites **186** *Laccophilus minimus* male, **187** female **188** *Laccophilus eboris* male, and **189** female **190** *Laccophilus leonensis* male, and **191** female **192** *Laccophilus villiersi* male, and **193** female **194** *Laccophilus melas* male, and **195** female **196** *Laccophilus livingstoni* male, and **197** female **198** *Laccophilus insularum* male, and **199** female. Scale bar 0.5 mm.

Figs 186–187 , 363 , 514 , 576

##### Type locality.

Namibia: East Caprivi, Mudumu NP, Buffalo Trails Camp.

##### Type material

(5 exs.). Holotype: male: “Namibia-Exp. ZMB 1992, East Caprivi: Mudumu NP: Buffalo Trails Camp, lux ca. 18°10'S/23°26'E, 12.3. 1992 leg. M. Uhlig” (ZMHB). – Paratypes: Same as holotype (1 ex. ZMHB); “Namibia-Exp. ZMB 1992, Kavango: Popa Falls 18°07'S, 21°35'E, lux, 26.2.-3.3. 1993 leg. M. Uhlig” (2 exx. ZMHB, 1 ex. MZH; habitus in Fig. 514).

##### Diagnosis.

*Laccophilus minimus* is probably most closely related to *Laccophilus canthydroides* and *Laccophilus inornatus*. The three species can be separated by comparison of the body size and differences in the shape of male genitalia. *Laccophilus canthydroides* is larger than *Laccophilus minimus*. Difference in the shape of penis apex is a useful feature for the separation of *Laccophilus minimus* from *Laccophilus inornatus*; interior frontal process of penis apex is blunt in *Laccophilus minimus*, while corresponding feature in *Laccophilus canthydroides* and *Laccophilus inornatus* is angled and sharp.

##### Description.

Body length 2.8–3.1 mm, width 1.5–1.6 mm. Elytra uniformly ferrugineous; generally with two pale spots on base of each elytron (Fig. 514).

Head: Pale ferrugineous. Impunctate, except at eyes; with very fine, irregular punctures. Areas with punctures extend towards middle of head-disc, leaving still a broad impunctate area in between. Rather shiny, although very finely microsculptured. Reticulation double but large meshes weakly developed and difficult to discern.

Pronotum: Pale ferrugineous; no distinct colour pattern. Rather shiny, finely to very finely microsculptured; reticulation double. Size categories of meshes, especially in middle, difficult to separate, because of extensive reduction of meshes (especially small meshes rudimentary). Laterally, microsculpture stronger. Frontally and laterally with fine, irregular punctures, which is absent basally in middle and on disc of pronotum.

Elytra: Ferrugineous. Each elytron laterally with vague paler area and basally, generally, with two minute, pale spots (Fig. 514). Rather shiny, although finely to very finely microsculptured. Reticulation double. Size-categories of meshes in part indistinct because of extensive reduction of meshes. Medially small meshes indistinct, in part absent. Laterally and posteriorly meshes stronger and clearly discernible. Very fine, somewhat irregularly distributed punctures form a discal row (narrow area of punctures) of punctures. Dorsolateral row simply indicated by a few fine punctures. Laterally with scattered fine punctures but no clear row (delimited area) of punctures formed. Pre-apical, lateral row of punctures shaped as a shallow pubescent furrow.

Ventral aspect: Pale ferrugineous to ferrugineous, without distinct colour pattern. Slightly mat, finely microsculptured. Sternal microsculpture in part indistinct. Ventrites with fine, slightly curved striae. Apical ventrite with an asymmetric, sharp knob on one side (Fig. 186). Stridulatory file ridges broad; strongest at midline – laterally ridges become gradually weaker and less pronounced. Metacoxal plates in anterior half with some fine, shallow, transverse furrows. Impunctate except apical ventrite, provided with some scattered, irregular punctures. Prosternal process slender, posteriorly somewhat extended, apically pointed.

Legs: Pale ferrugineous to ferrugineous. Pro- and mesotarsus slightly enlarged; with distinct suckers.

Male genitalia: Internal part of penis apex is large and rounded; not angled and sharp (Fig. 363).

Female: Apical ventrite lacks asymmetric knob (Fig. 187). Pro- and mesotarsus slender.

##### Etymology.

The name *minimus* is a Latin comparative adjective meaning “the smallest”. It here refers to the small body-size of the new species.

##### Distribution.

Namibia (Fig. 576).

##### Collecting circumstances.

Almost unknown. Collected at light.

#### Laccophilus eboris sp. n.

Taxon classificationAnimaliaColeopteraDytiscidae

http://zoobank.org/CDF427FA-4113-48FD-B2F3-4635F87116A9

Figs 188–189 , 364 , 515 , 577

##### Type locality.

Ivory Coast: Bingerville.

##### Type material

(26 exs.). Holotype: male: “Coll. Mus. Tervuren, Cote d’Ivoire: Bingerville VI. 1962 J, Decelle (MRAC; habitus in Fig. 515). – Paratypes: Same data as holotype (5 exs. MRAC, 2 exs. MZH); same data as holotype but “1961”and “à la lampe U.V.” (3 exs. MRAC, 1 ex. MZH); same data as preceding but lacks text on UV lamp (1 ex. MRAC); same as preceding but “XII. 1961” (1 ex. MRAC); same data as preceding but “VIII. 1962” (6 exs. MRAC, 1 ex. CGW); same data as preceding but “XI. 1962” (1 ex. MRAC); same data as preceding but “IX. 1963” (1 ex. MZH); same data as preceding but “XI. 1963” (1 ex. MZH); same data as preceding but “1-18.III. 1963” (2 exs. CGW).

##### Diagnosis.

*Laccophilus eboris* is also a species close to *Laccophilus inornatus*, *Laccophilus canthydroides* and *Laccophilus minimus*. *Laccophilus eboris* belongs to the smaller species among them and it has unicoloured, dark elytra without any signs of paler spots. Additionally the apical grove at internal edge of penis distinguishes *Laccophilus eboris* from the other species, which lack a similar feature on penis apex.

##### Description.

Body length 3.2–3.3 mm, width 1.6–1.7 mm. Dorsal, colour pattern quite uniform with minor variation (Fig. 515).

Head: Pale ferrugineous; posteriorly sometimes slightly darker. Dark area not distinctly delimited but vague. Slightly mat, finely microsculptured. Reticulation double. Large meshes very fine and weakly developed; in part rudimentary. Large meshes, when discernible, may contain 2–5 small meshes. Impunctate, except at eyes; with fine irregular punctures. Areas of punctures extended inwards but leave a distinct gap without punctures in middle of head-disc.

Pronotum: Pale ferrugineous, basally with a distinct, blackish to dark brown area. Frontally at area between eyes, generally, with a vague darker area, which may be rather indistinct. Slightly mat, finely microsculptured. Reticulation double. Large meshes weakly developed, in part rudimentary. When discernible, large meshes may contain 2–5 small meshes. Almost impunctate. Along margins except at base in the middle, with fine to very fine, scattered, irregular punctures.

Elytra: Blackish ferrugineous, posteriorly slightly paler but with no distinct colour pattern (Fig. 515). Slightly mat, finely microsculptured. Reticulation double. Large meshes weakly developed, in part rudimentary. When discernible, large meshes may contain 2–5 small meshes. Fine, irregularly distributed, sparse punctures form a discal row. Outside discal row fine punctures appear scattered and irregular. No distinct rows of punctures formed. Pre-apical, lateral furrow provided with hairs.

Ventral aspect: Ferrugineous, abdomen slightly darker; ferrugineous to dark ferrugineous. Prothorax distinctly paler; pale ferrugineous. Rather shiny, although finely microsculptured. Ventrites with fine, slightly curved striae. Almost impunctate; apically on abdomen with fine, irregular punctures. Apical ventrite quite strongly modified, asymmetric with a sharp, lateral knob and rude grooves on one side and fine surface-structures on the other side (Fig. 188). Stridulatory apparatus on metacoxal plates quite broad and curved; consists of rather narrow ridges out of which about 25 are clearly discernible. Anterior half of metacoxal plate with about 10 almost transverse, shallow furrows. Prosternal process slender, posteriorly slightly extended; apex pointed.

Legs: Pro- and mesotarsus slightly extended and enlarged, provided with suckers.

Male genitalia: Outline of frontal edge of penis almost straight; apex with a small but clearly discernible cavity (in part seen in illustration) (Fig. 364).

Female: Pro- and mesotarsus slender. Apical ventrite as in Fig. 189.

##### Etymology.

The Latin noun ebur means ivory and the species name *eboris* is the genitive form of it, simply associating the species with the Ivory Coast.

##### Distribution.

Ivory Coast (Fig. 577). *Laccophilus eboris* is only known from the type locality.

##### Collecting circumstances.

Almost unknown. A number of labels give light collection with a UV lamp.

#### Laccophilus leonensis

Taxon classificationAnimaliaColeopteraDytiscidae

Régimbart, 1895

Figs 190–191 , 365 , 516 , 577

Laccophilus leonensis Régimbart 1895: 147 (original description, faunistics); Zimmermann 1920a: 21 (catalogue, faunistics); Peschet 1925: 31 (faunistics, description, discussion); Gschwendtner 1943: 417 (discussion); Guignot 1953b: 234, 236, 237 (female description, faunistics, discussion); Omer-Cooper 1953: 24 (discussion); Guignot 1956b: 220, 221 (discussion, description); Guignot 1958a: 7 (discussion); Guignot 1959a: 579, 580, 581 (description, faunistics); Guignot 1959d: 163 (faunistics, discussion); Guignot 1961b: 238 (discussion); Bertrand and Legros 1975: 681 (faunistics, list); Bilardo and Pederzani 1978: 119 (faunistics, description); Medler 1980: 155 (faunistics, list); Pederzani and Rocchi 1982: 72 (faunistics); Rocchi 1991: 86 (faunistics, list); Nilsson et al. 1995: 506 (faunistics); Nilsson 2001: 245 (catalogue, faunistics); Bilardo and Rocchi 2002: 156, 161, 162, 164, 174 (discussion, faunistics); Bilardo and Rocchi 2008: 211, 236 (biology); Bilardo and Rocchi 2013: 141 (faunistics, biology); Nilsson 2015: 213 (catalogue, faunistics).

##### Note on taxonomy.

References after 1920 are in part referring also to other *Laccophilus* species but *Laccophilus leonensis*.

##### Type locality.

Sierra Leone: Rhobomp.

##### Type material studied

(1 ex.). Holotype: female: “Sierra Leone Rhobomp Moquerys / Museum Paris coll. Maurice Régimbart 1908 / *Laccophilus leonensis* Reg.” (MNHN).

##### Additional material studied

(13 exs.). Sierra Leone: “Makeni 12°03'W 8°53'N 27.11.1993 light trap 19-21 / Cederholm, Danielsson & Hall leg. / *Laccophilus leonensis* Régb. det. Persson” (8 exs. MZLU; habitus in Fig. 516); same data but “28.11. 1993” (5 exs. MZLU).

##### Diagnosis.

From other species in this species group *Laccophilus leonensis* is distinguished by one-coloured, quite dark elytra and mainly pale pronotum (colour contrast strong; distinctly bicoloured) in combination with minor details in penis apex; apex of penis smooth and not wrinkled.

##### Description.

Body length 3.0–3.3 mm, width 1.6–1.8 mm. Elytra lack distinct colour pattern; almost uniformly dark brown to dark ferrugineous, lacking pale spots (Fig. 516).

Head: Pale ferrugineous to ferrugineous, rarely head becomes posteriorly gradually, slightly darker. Submat, finely microsculptured. Reticulation indistinctly double; large meshes only slightly, more strongly developed in comparison with small meshes. Large meshes contain 3–6 small meshes. Impunctate, except at eyes, with fine, irregular punctures.

Pronotum: Pale ferrugineous to ferrugineous, mediobasally often with dark ferrugineous area. Rather shiny, although microsculptured. Reticulation double. Large meshes, especially on disc weakly developed; only slightly coarser in comparison with fine meshes. Large meshes, in general, contain 3–6 fine meshes. Impunctate, except at margins, with very fine, scattered punctures. Mediobasally impunctate.

Elytra: Dark ferrugineous to dark brown to dark ferrugineous (Fig. 516). Rather shiny, although distinctly and finely microsculptured. Reticulation double; large meshes contain 2–5 small meshes. Small meshes in part indistinct and hardly discernible. Fine and scattered punctures form a discal row of punctures. Dorsolateral and lateral rows of punctures absent or indicated by a few scattered punctures.

Ventral aspect: Ferrugineous, abdomen posteriorly slightly darker, ferrugineous to dark ferrugineous. Almost impunctate. Rather shiny, although finely microsculptured. Abdomen basally with some fine and curved striae. Metacoxal plates besides stridulatory apparatus, anteriorly with 7–8 shallow, transversely located furrows which in part are confluent. Apical ventrite asymmetric; with one distinct, lateral knob (Fig. 190).

Legs: Pro- and mesotarsus slightly enlarged; with suckers.

Male genitalia: Apex of penis characteristic; smooth and not wrinkled (Fig. 365).

Female: Almost as male but, apical ventrite symmetric (Fig. 191). Pro- and mesotarsus slender.

##### Distribution.

Sierra Leone (Fig. 577). Unchecked literature records from countries outside Sierra Leone are considered uncertain.

##### Collecting circumstances.

Almost unknown. Sampled at light collection.

#### Laccophilus villiersi

Taxon classificationAnimaliaColeopteraDytiscidae

Bertrand & Legros, 1975

Figs 192–193 , 366 , 517 , 576

Laccophilus villiersi Bertrand and Legros 1975: 671, 681 (original description, faunistics); Nilsson 2001: 253 (catalogue, faunistics); Nilsson 2015: 219 (catalogue, faunistics).

##### Type locality.

Congo Brazzaville: Odzala.

##### Type material studied

(5 exs.). Holotype: male: “Data in NHRS JLKB 000030290 / Odzala Congo Octobre / Museum Paris Mission A. Descarpentries et A. Villiers 1963-1964 / Type / R. Mouchamps det. 65 *Laccophilus villiersi* nsp” (MNHN). – Paratypes: Same data but “JLKB 000030291 / Allotype” (1 ex. MNHN); same data, but “JLKB 000030292-000030294 / Paratype” (3 exs. MNHN; habitus in Fig. 517).

##### Additional material studied

(4 exs.). **Central African Republic**: “La Maboke 6-9.6. 1973 Linnavuori leg.” (1 ex. MZH). – **Gabon**: “Lagune Iguéla Ntchongorovié (Savane) 22-24.8. 1998 Bilardo” (1 ex. CSR); “Libreville 3.8. 1973 Bilardo & Pederzani” (2 exs. NHMB).

##### Diagnosis.

Besides *Laccophilus melas*, *Laccophilus villiersi* is characterized by mainly dark body colour in this species group. *Laccophilus villiersi* is smaller than *Laccophilus melas*. In both species diagnostic, important features are exhibited by apex of the penis; provided with processes in *Laccophilus villiersi* while almost smooth in *Laccophilus melas*.

##### Description.

Body length 2.8–3.0 mm, width 1.6 mm. Habitus as in Fig. 517. Elytra dark brownish to dark ferrugineous, apically slightly paler. Elytron with a small but distinct, pale ferrugineous, humeral spot. Dorsal colour pattern exhibits only minor variation.

Head: Ferrugineous to pale ferrugineous. Slightly mat, with fine reticulation which is double. Larger meshes weakly developed; when discernible they contain 2–6 fine meshes. Impunctate, except in small area at eyes where punctation is fine and irregularly distributed.

Pronotum: Ferrugineous to pale ferrugineous; lacks distinct colour pattern. Slightly mat although finely microsculptured. Reticulation double; large meshes discally in part weakly developed, when discernible they contain 2–5 fine meshes. Impunctate, except at anterior margin where fine, irregular punctures discernible.

Elytra: Blackish ferrugineous, with pale, small, humeral spots (Fig. 517). Slightly mat, finely microsculptured. Large meshes (especially on disc) in part reduced and hardly discernible. When discernible large meshes contain 2–5 small meshes. Very fine, irregular, row of punctures indicate a discal row. Elytra laterally with fine, pre-apical, row of punctures, which is finely pubescent. Other rows indistinct.

Ventral aspect: Ferrugineous to pale ferrugineous; abdomen slightly darker but no distinct colour pattern formed. Slightly mat, finely microsculptured. Almost impunctate. Metacoxal plates with a few, shallow, transverse furrows. Abdomen with a few, fine, somewhat curved striae. Prosternal process slender, posteriorly somewhat extended, apically pointed. Apical ventrite with an asymmetrically located knob (Fig. 192).

Legs: Pro- and mesotarsus slightly enlarged, provided with fine suckers.

Male genitalia: Penis small; anterior edge somewhat rounded and internal edge of apex with a small extension (Fig. 366).

Female: Externally almost as male. Pro- and mesotarsus narrow. Apical ventrite shape almost symmetric (Fig. 193).

##### Distribution.

Central African Republic, Gabon, Congo Brazzaville (Fig. 576).

##### Collecting circumstances.

Not known.

#### Laccophilus melas

Taxon classificationAnimaliaColeopteraDytiscidae

Guignot, 1958

(Figs 194–195 , 367 , 518 , 577)

Laccophilus melas Guignot 1958: 8 (original description, faunistics); Nilsson 2001: 246 (catalogue faunistics); Nilsson 2015: 216 (catalogue faunistics).

##### Type locality.

Zaire: Garamba National Park.

##### Type material studied

(14 exs.). Holotype: male: “Holotypus / Congo Belge, P.N.G. Miss. H. De Saeger II / gd/11 4-X-1951 Réc. 2511, H. de Saeger / Coll. Mus. Congo (ex. coll. I.P.N.C.B.) / Guignot det., 1957 *Laccophilus melas* sp. n. Holotypus” (MRAC; habitus in Fig. 518). – Paratypes: Same data as holotype but “Paratype” (1 ex. NHMB); “Congo Belge, P.N.G. Miss. H. De Saeger II/gd/11 24-VI-1952 H. De Saeger. 3693 / Paratype / F. Guignot det., 1958 *Laccophilus melas* sp. n. Paratype” (1 ex. IRSNB); same data but “Pali ´´/8 24-VII-1952, 3816” (2 exs. IRSNB); same data but “8-VIII-1952, 3924” (3 exs. IRSNB); same data but “Ndelele/11, 21-II-1952, 3143” (4 exs. IRSNB); same data but “II/fc/11, 25-IV -1952, 3702”(1 ex. IRSNB); same data but “II/me/9, 28.2. 1952, 3156” (1 ex. NHMB).

##### Additional material studied

(4 exs.). **Cameroon**: “Nanga Eboko / 7-10. 59 Lenczy” (1 ex. CGW). – **Zaire**: “PNG II/gd/11, 19.8. 1952 De Saeger 3956” (1 ex. MRAC, 1 ex. MZH); “PNG PpK.51/g/9, 2, 4, 1952 De Saeger 3272” (1 ex. MRAC).

##### Diagnosis.

*Laccophilus melas* is particularly characterized by black to blackish ferrugineous colour of body. Pale colour reduced to two minor spots on pronotum posterior to eyes and to very fine, small, pale spots on humeral region and laterally posterior to middle on elytra. Penis exhibits also minor but diagnostically useful characters; penis quite large, evenly curved and apex outline rounded; basally with an enlargement (vide also diagnosis of *Laccophilus villiersi* on p. 248).

##### Description.

Body length 3.1–3.5 mm, width 1.7–1.9 mm. Dorsal, colour pattern reduced to a few pale spots (Fig. 518).

Head: Blackish to dark ferrugineous. Submat, finely reticulated. Reticulation double; large meshes extensively rather indistinct; only slightly more strongly developed than fine meshes. Large meshes generally contain 3–6 fine meshes. Punctation almost absent; frontally at eyes fine, irregular punctures discernible.

Pronotum: Blackish to dark ferrugineous. At foremargin slightly paler; posterior to eyes close to foremargin, with minute yellowish spot. Submat, finely reticulated; reticulation double. Minor differences between mesh-size classes. Large meshes generally contain 3–6 small meshes. At margins fine punctures may be discerned.

Elytra: Blackish to dark ferrugineous. Laterally, slightly posterior to middle with a minute yellowish spot (Fig. 518). Submat, rather shiny, finely reticulated. Double reticulation anteriorly clearly visible; posteriorly fragmentary and indistinct. Scattered, very fine punctures may be discerned. Lateral, pre-apical furrow fine, pubescent.

Ventral aspect: Blackish ferrugineous to dark ferrugineous. Submat, finely reticulated. Almost impunctate. Metacoxal plates in provided with a broad stridulatory file, delimitation of which is vague. Prosternal process slender, apex somewhat extended backwards, pointed. Transversely located, shallow furrows discernible on anterior half of metacoxal plates. Apical ventrite on one side with a distinct knob (Fig. 194). Basal ventrites with fine, sparse striae.

Legs: Protarsus slender, claws equally long and slightly curved. Pro- and mesotarsus with suckers.

Male genitalia: Penis comparatively large, evenly curved and apex outline rounded; lacks processes or extensions. At base, ventrally, with a distinct enlargement (Fig. 367).

Female: Apical ventrite almost symmetric, lacks lateral knob (Fig. 195). Pro- and mesotasus slender.

##### Distribution.

Cameroon, Zaire (Fig. 577).

##### Collecting circumstances.

Unknown.

#### Laccophilus livingstoni

Taxon classificationAnimaliaColeopteraDytiscidae

Omer-Cooper, 1958

Figs 196–197 , 368 , 519 , 576

Laccophilus livingstoni Omer-Cooper 1958b: 37, 41, 45 (original description, faunistics, biology); Omer-Cooper 1967: 60 (discussion, *Laccophilus livingstoni*, junior synonym *Laccophilus inornatus* Zimmermann); Medler 1980: 155 (faunistics, list); Nilsson 2001: 245 (catalogue, faunistics); Nilsson 2012: 212 (catalogue, faunistics, list, synonymy, *Laccophilus inornatus*). **Restored species.**

##### Type locality.

Malawi: Monkey Bay, Dambo.

##### Type material studied

(6 exs.). Holotype: male: “Paratype / Nyasaland, swamp, Monkey Bay 28.1. 1948 / *Laccophilus livingstoni* O-C. (AMGS; by mistake labelled as paratype).” – Paratypes: “Holotype / Nyasaland Livingstonia, stream by homestead 22.10. 1948 J.O.C. / *Laccophilus livingstoni* O-C. / Brit. Mus. 1957-660 / *Laccophilus livingstoni* J. O.C., M.E Bacchus det. 1978, Holotype/Brit. Mus. 1978-308” (1 ex. BMNH); same, but labelled as “Allotype” (1 ex. BMNH). [Comment: The specimens are pinned together. Label information fits with paratypes, but does not fit with what is given for the holotype in the original description.]; “Paratype / Nyasaland stream 20 mi. From Dedza on lower Lilongwe rd. 30.9. 1948 / *Laccophilus livingstoni* sp. n. Det. J. Omer-Cooper” (2 exs. AMGS); (Tanzania)“Zanzibar Pemba 6. September 1955 J.R. Fowler” (1 ex. AMGS).

##### Additional material studied

(13 exs.): **Tanzania**: “Lake Malawi Matema 1.7. 1979 M. Stoltze leg. / *Laccophilus bergeri* Guignot Holmen det.” (2 exx. ZMUC); “Zanzibar, Manganpwani Rd. 13.9. 1955 JOC.” (2 exs. AMGS). – **Malawi**: “Mulanje Mnts env. 22-28.12. 2001 Kantner” (3 exs. NHMB, 1 ex. MZH; habitus in Fig. 519); “Selima env. 5-6.1. 2002 60 km E of Lilongwe Kantner” (3 exs. NHMB, 1 ex. MZH). – **Mozambique**: “Prov. Manica 20 km NW Chimoio 21-23.12. 2003 Kudrna jr. lgt.” (1 ex. CFP).

##### Comments on synonymy.

Examination of holotype reveals that *Laccophilus livingstoni* is a good species and not a junior synonym of *Laccophilus inornatus* Zimmermann.

##### Diagnosis.

*Laccophilus livingstoni* is closest related to *Laccophilus insularum*. There are, however, some differences in shape of apex of penis; anterior outline of it being almost straight in *Laccophilus livingstoni* and clearly curved in *Laccophilus insularum*. *Laccophilus livingstoni* also resembles quite much of *Laccophilus garambanus* regarding colour pattern of body. *Laccophilus garambanus* is, however, smaller and have more extensive pale colour on posterior half of elytra.

##### Description.

Body length 3.3–3.6 mm, width 1.8–2.0 mm. Dorsal colour pattern quite uniform, exhibits only some variation (Fig. 519).

Head: Pale ferrugineous; posteriorly at pronotum narrowly, slightly darker. Discally, with two, small, dark spots which sometimes are vague. Punctation slightly irregularly distributed, at eyes and from there towards central part, fine to very fine. Submat, finely microsculptured. Reticulation double, but in part this is rather indistinct. Large meshes contain 2–6 finer meshes.

Pronotum: Pale ferrugineous to dark ferrugineous, with vague darker areas. At margins with fine to very fine, irregularly distributed punctures. Reticulation double; large meshes in part lack small meshes, and in part, contain 2 to 5 small meshes.

Elytra: Blackish ferrugineous to dark ferrugineous. Anteriorly with an irregular, subbasal series of pale spots which often are in part confluent. Posteriorly, with vague, pale and irregular stripes (Fig. 519). Rather shiny, although densely microsculptured. Reticulation frontally double; towards apex division into two kinds of reticulation becomes indistinct. Three, (a discal, a dorsolateral and a lateral one) sparse and irregular rows of punctures (or narrow punctured areas) discernible.

Ventral aspect: Pale ferrugineous to ferrugineous; ventrites laterally somewhat darker. Rather shiny, although very finely reticulated. Ventrites with sparse but distinct striae pointing inwards and backwards. Almost impunctate. Ventrite posterior to apex of abdomen with a few rather irregular punctures; on one side provided with a sharp knob (Fig. 196). Metacoxal plates with about 10 almost transversely located, shallow furrows. Prosternal process narrow and pointed.

Legs: Pro- and mesotarsus slightly enlarged, provided with suckers.

Male genitalia: Penis angled (not evenly curved); external outline apically extended and sharp (Fig. 368).

Female: Externally as male but pro- and mesotarsus fairly long and slender. Apical ventrite lacks sharp knob (Fig. 197).

##### Distribution.

Malawi, Tanzania, Mozambique (Fig. 576). Record from Nigeria is incorrect, and refers to earlier accepted synonymy.

##### Collecting circumstances.

Insufficiently known. The species has been collected from both running and standing waters.

#### Laccophilus insularum sp. n.

Taxon classificationAnimaliaColeopteraDytiscidae

http://zoobank.org/5126E56D-3F25-40E1-99BE-B851F1D84AA6

Figs 198–199 , 369 , 520–521 , 578

##### Type locality.

Madagascar: Ankarafantsika NP, Mahajanga, Boeny (S16.30271, E046.80995).

##### Type material

(35 exs.): Holotype: male: “Madagascar Mahajanga: Boeny Ankarafantsika NP, S16.30271, E046.80995, 75 m.a.o. 8.12. 2009 Water net, field# MAD09–39 Bergsten et al.” (NHRS). – Paratypes: “Madagascar (95)25.ix. 2001 Ranohira (Fianarantsoa), right affl. of Riv. Ihazofotsy nr Isalo Ranch, stagnant areas/750 m asl, 21.0°C 0.005 mS/cm/Gerecke & Goldschmidt collectors BMNH(E) 2004-46” (BMNH; habitus in Fig. 520); “W-Madagascar (19) Manindaray, W Sakahara 700-800 m asl., 30.01. 1995 G. Dunay & J. Janák coll.” (1 ex. NMW); “S-Madagascar (14) Ambatoveve, Road Betioky-Beneloka 150 m a.s.l., 26.01. 1995 G. Dunay & J. Janák coll.” (1 ex. NMW, 1 ex. MZH); “S-Madagascar (15) Ambialialika, Road Betioky-Beneloka 50 m a.s.l., 27.01. 1995 G. Dunay & J. Janák coll.” (1 ex. NMW; habitus in Fig. 521); “BMNH(E) <745072> DNA voucher/MAD: FIAN: Isalo Source of Piscine Naturelle: Waterhole P41K: N: -22.553: E:45.368, 859 m, 12.V. 2006: Leg. Bergsten et al” (1 ex. MZH); same data but “<794195> DNA voucher” (1 ex. MZH); same data but “<745073> DNA voucher” (1 ex. NHRS); same data but “<745074> DNA voucher” (1 ex. NHRS); “DNA Voucher BMNH(E) <831021>MSL 402:E6/MAD. Isaky-Ivondro Ampasy (E2); rice paddies: P66: N-24.93: E:46.863: 64 m, 08.IV. 2007 Leg. Isambert et al.” (1 ex. NHRS); same data but “BMNH(E) <831023>MSL 402:E8” (1 ex. NHRS); Same data but “BMNH(E) <831022>MSL 402:E7” (1 ex. NHRS); “BMNH(E) <742297> DNA voucher/MAD: FIAN: Isalo: River R41D, N:-22.486: E:45.383: 723 m 11.V. 2006 Leg. Bergsten et al” (1 ex. NHRS); “RM: Betsiboka Bas. (PO123) Loc. Ambalambongo aff. de Betsiboka Riv., 47°00'30"E/16°48'00"S, 30.3. 1993 leg. ORSTOM Antananarivo” (1 ex. NHRS/NMW); “RM: Betsiboka Bas.(PO124) Loc. Ambohimanatrika Kamoro Riv. 47°10'06"E/16°28'55"S, 1.4. 1993 leg. ORSTOM Antananarivo” (1 ex. NHRS/NMW); “Mahajanga: Melaky btw. Morafenobe-Ambohijanahary S18.19091 E045.19986, 290 m.a.o., 19.12. 2009 Water net, field#MAD09-74 Bergsten et al / NHRS-JLKB 0000000479” (1 ex. NHRS); same data but not vouchers (6 exs. NHRS); same data but “S18.19880, E045.15651, 313 m.a.o., MAD09-73 / NHRS-JLKB 0000000484” (1 ex. NHRS); same data as holotype (7 exs. NHRS); “Mahajanga: Boeny Ankarafantsika NP, S16.30271, E046.80995, 75 m.a.o. 8.12. 2009 Water net, field# MAD09-39 Bergsten et al / NHRS-JLKB 0000000491” (1 ex. NHRS); same data but “29.11. 2009, MAD09-10 / NHRS-J LKB 0000000486 (1 ex. NHRS); Est, Sambava c. 20 m Janac & Moravec leg. / 3.3. 1996 Riv. Anovona env., lux, rizieres / bord de la foret” (1 ex. NMPC).

##### Diagnosis.

*Laccophilus insularum* is very closely related to *Laccophilus livingstoni*, occurring in mainland of Africa. Clear differences in appearance of penis apex show that they are not conspecific, but represent good species (vide diagnosis of *Laccophilus livingstoni* on p. 250).

Description (Only diagnostic differences from description of *Laccophilus livingstoni* considered.): Body length 3.0–3.6 mm, width 1.7–2.0 mm. Dorsal, colour pattern uniform; pale spots on elytra may sometimes be in part reduced or expanded (Figs 520–521).

Head: Uniformly pale; posteriorly not darker.

Pronotum: Pale ferrugineous, basally with vague, darker area.

Elytra: Basal, pale markings variable in size; sometimes somewhat extended (Figs 520–521).

Ventral aspect: Apical ventrite (Fig. 198). Stridulatory apparatus distinct; file of ridges quite broad.

Male genitalia: Penis apex distinct; outline rounded and apex expanded both at external and internal edge (Fig. 369).

Female: Apical ventrite (Fig. 199).

##### Etymology.

The name *insularum* is a Latin noun in genitive plural form meaning “belonging to the island”. It refers to the fact that the new species is only known from Madagascar.

##### Distribution.

Madagascar (Fig. 578).

##### Collecting circumstances.

Insufficiently known. At least once collected in a river, in stagnant sites of the water body.

#### Laccophilus garambanus

Taxon classificationAnimaliaColeopteraDytiscidae

Guignot, 1958

**Figures 200–208.**
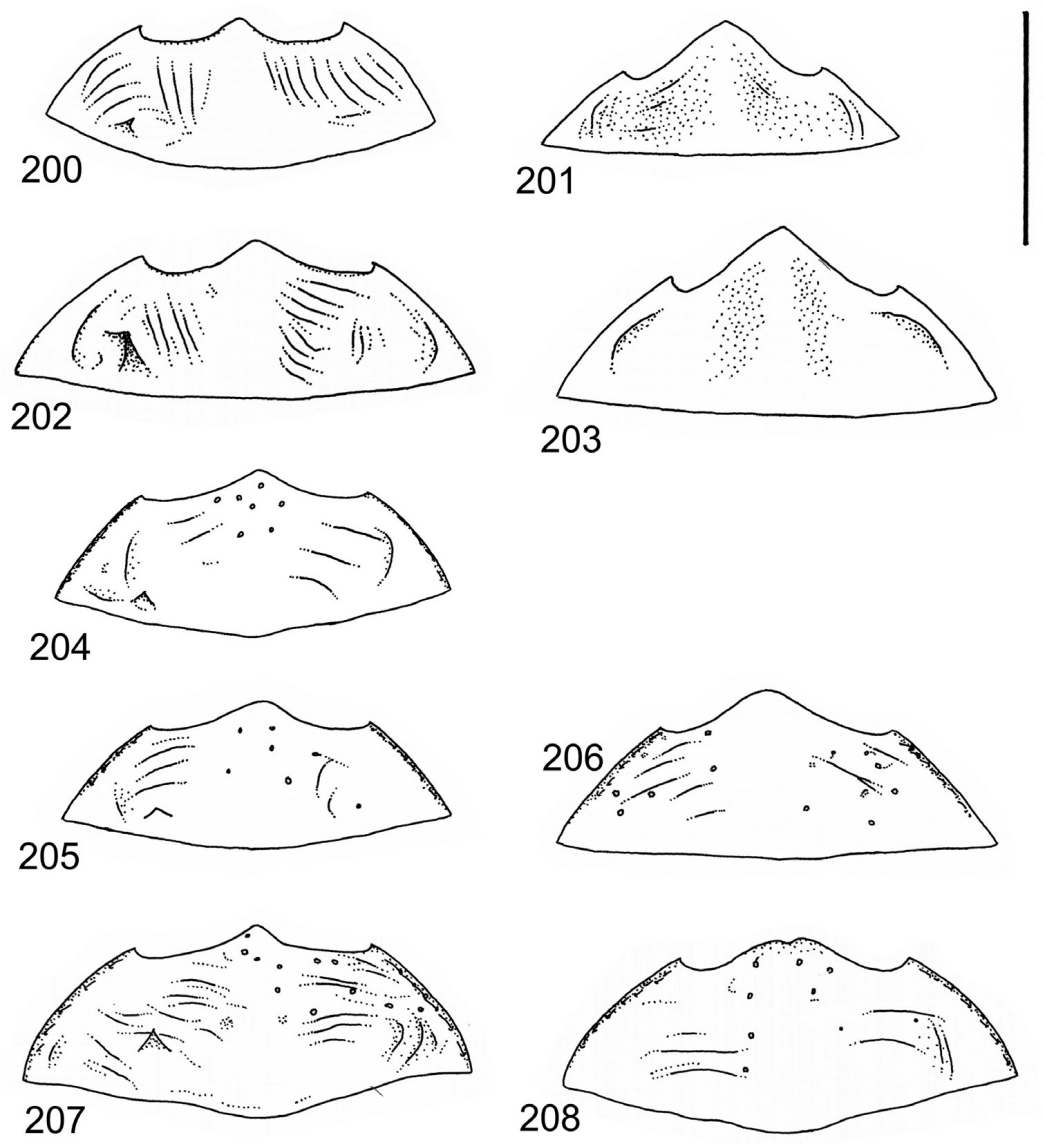
Apical ventrites **200** *Laccophilus garambanus* male, **201** female **202** *Laccophilus flavopictus* male, and **203** female **204** *Laccophilus laeticulus* male **205** *Laccophilus occidentalis* male, and **206** female **207** *Laccophilus transversovittatus* male, and **208** female. Scale bar 0.5 mm.

**Figures 209–218.**
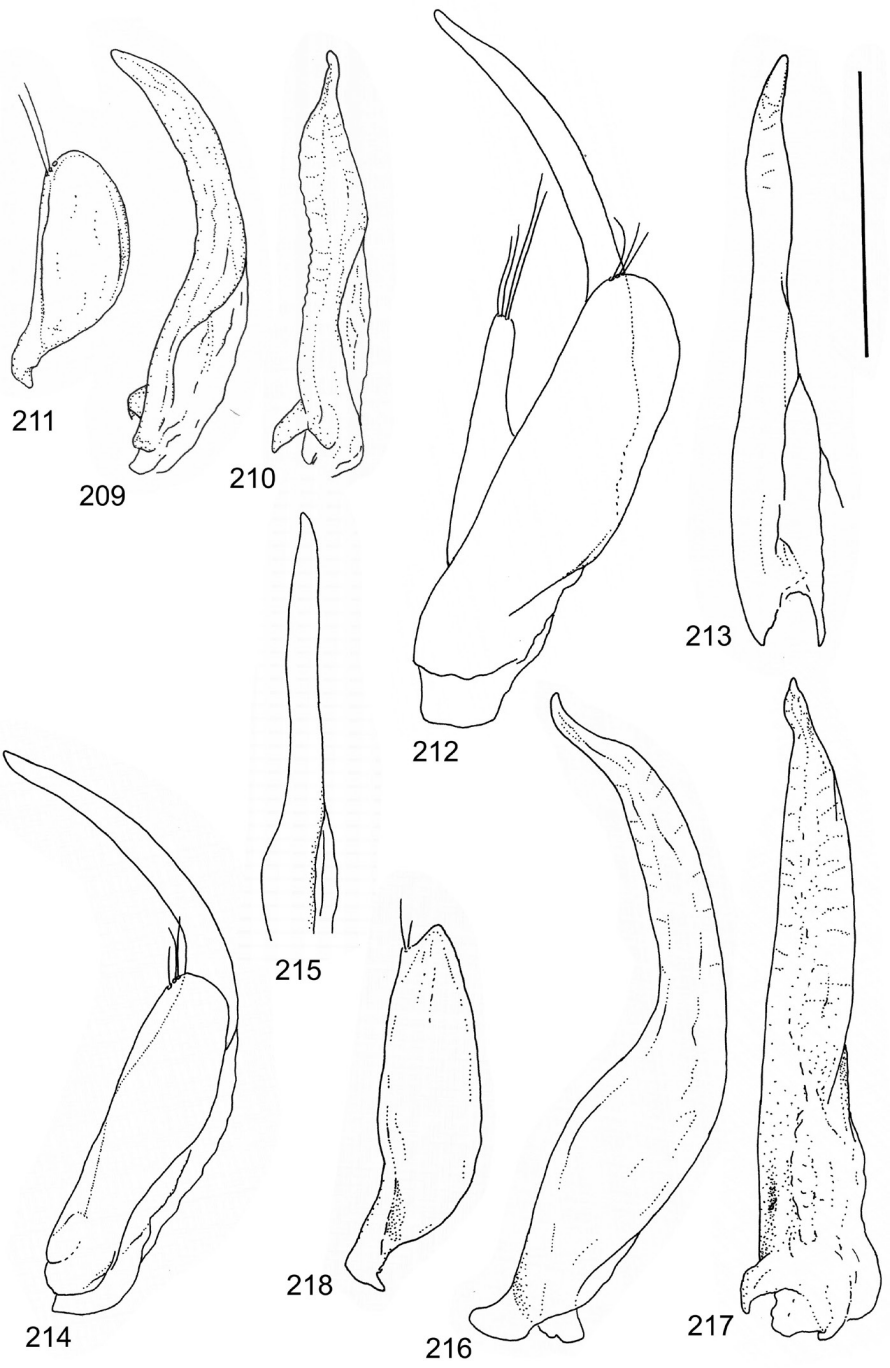
Male genitalia **209** *Laccophilus tavetensis*, penis, lateral aspect **210** penis, dorsal aspect, and **211** paramere **212** *Laccophilus grossus*, penis and paramere, lateral aspect, and **213** penis, dorsal aspect **214** *Laccophilus rocchii*, penis and paramere, lateral aspect, and **215** penis, dorsal aspect **216** *Laccophilus morondavensis*, penis, lateral aspect **217** penis, dorsal aspect, and **218** paramere. Scale bar 0.5 mm.

**Figures 219–229.**
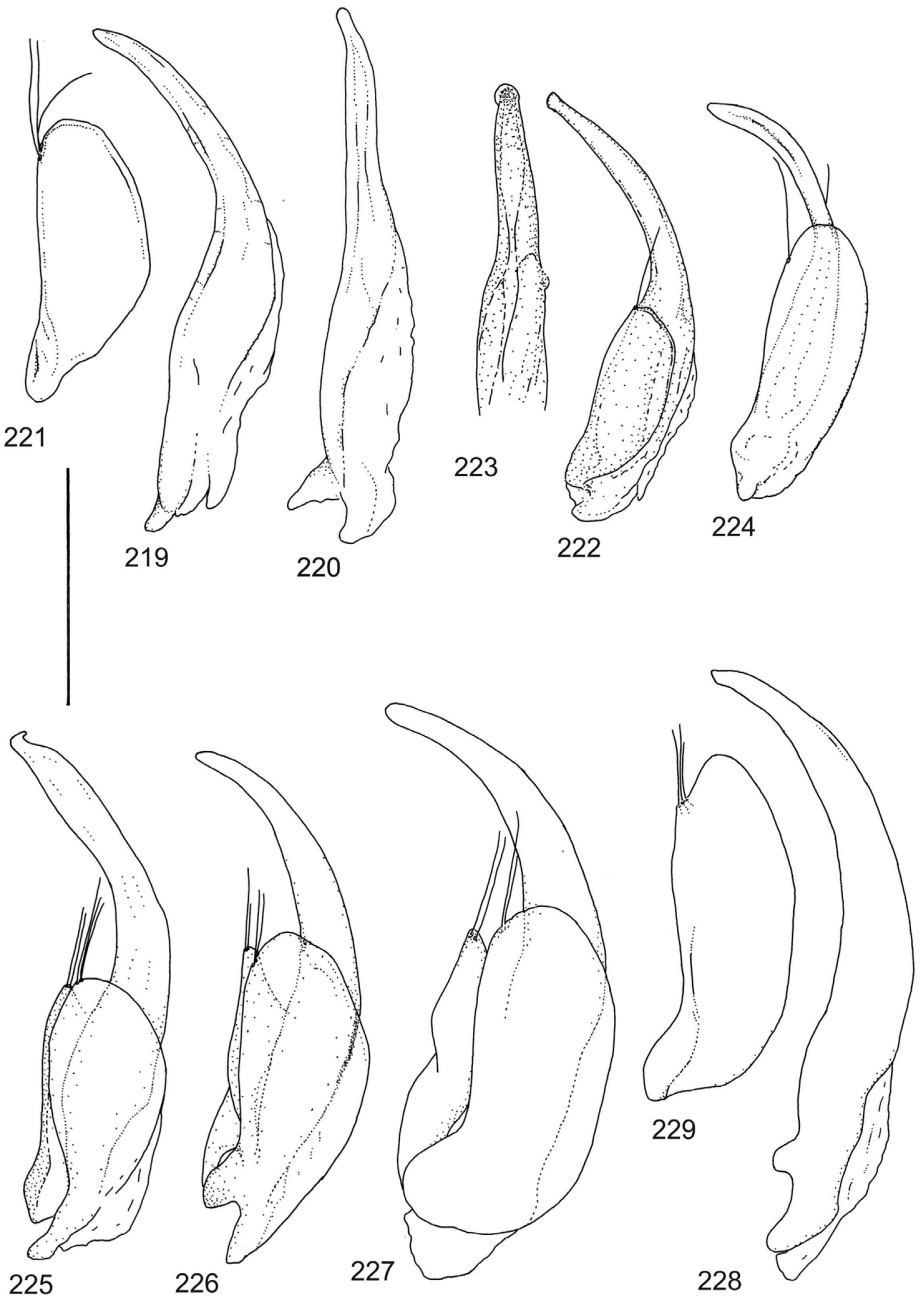
Male genitalia **219** *Laccophilus productus*, penis, lateral aspect **220** penis, dorsal aspect, and **221** paramere **222** *Laccophilus ferrugo*, penis and paramere, lateral aspect, and **223** penis, dorsal aspect **224** *Laccophilus ruficollis*, penis and paramere, lateral aspect **225** *Laccophilus hyalinus*, penis and paramere, lateral aspect **226** *Laccophilus minutus*, penis and paramere, lateral aspect **227** *Laccophilus mateui*, penis and paramere, lateral aspect **228** *Laccophilus sordidus*, penis, lateral aspect, and **229** paramere, lateral aspect. Scale bar 0.5 mm.

**Figures 230–239.**
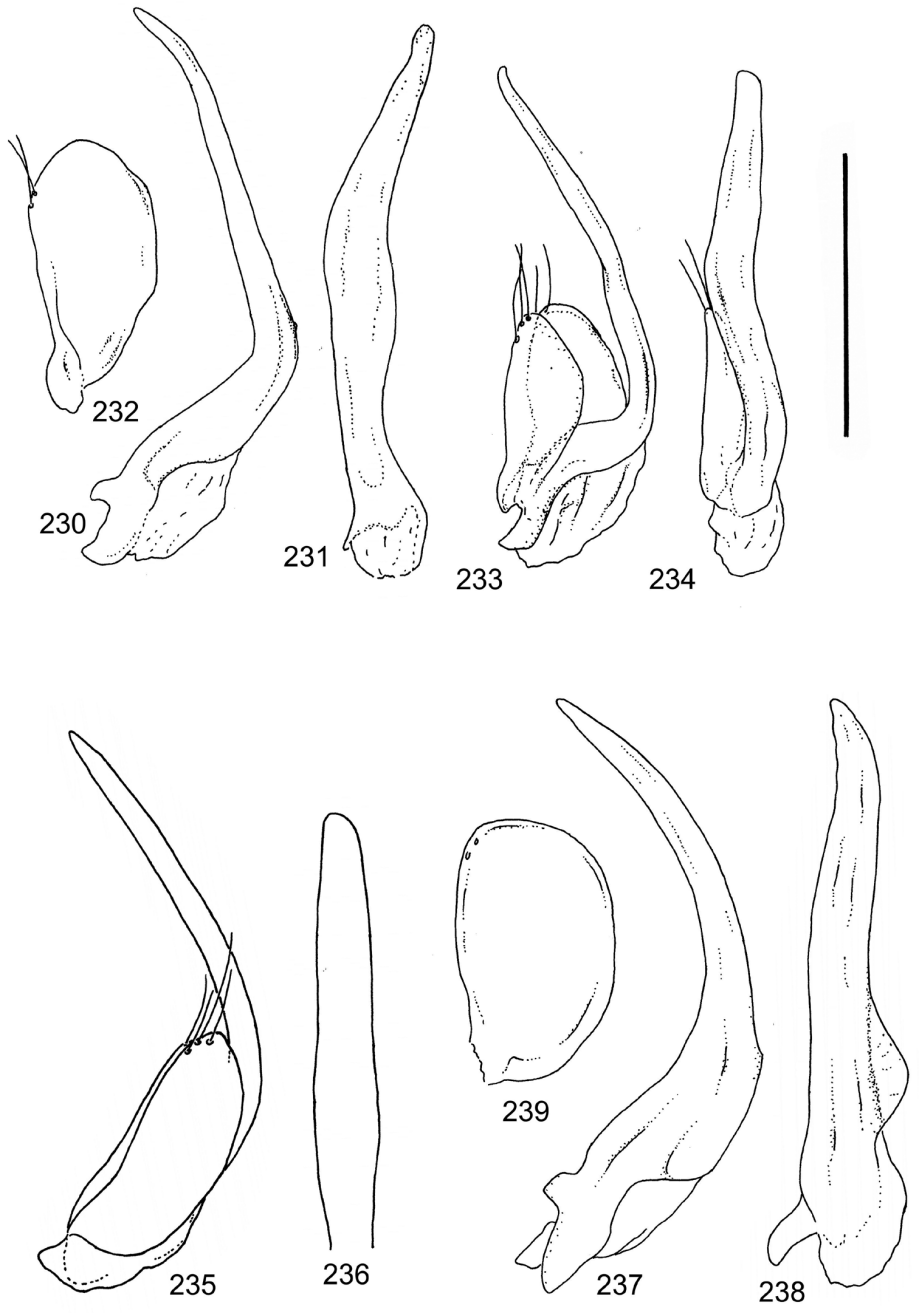
Male genitalia **230** *Laccophilus comes*, penis, lateral aspect **231** penis, dorsal aspect, and **232** paramere **233** *Laccophilus alluaudi*, penis and paramere, lateral aspect, and **234** penis and paramere, dorsal aspect **235** *Laccophilus furthi*, penis and paramere, lateral aspect, and **236** penis, dorsal aspect **237** *Laccophilus tigrinus*, penis, lateral aspect **238** penis, dorsal aspect, and **239** paramere. Scale bar 0.5 mm.

**Figures 240–248.**
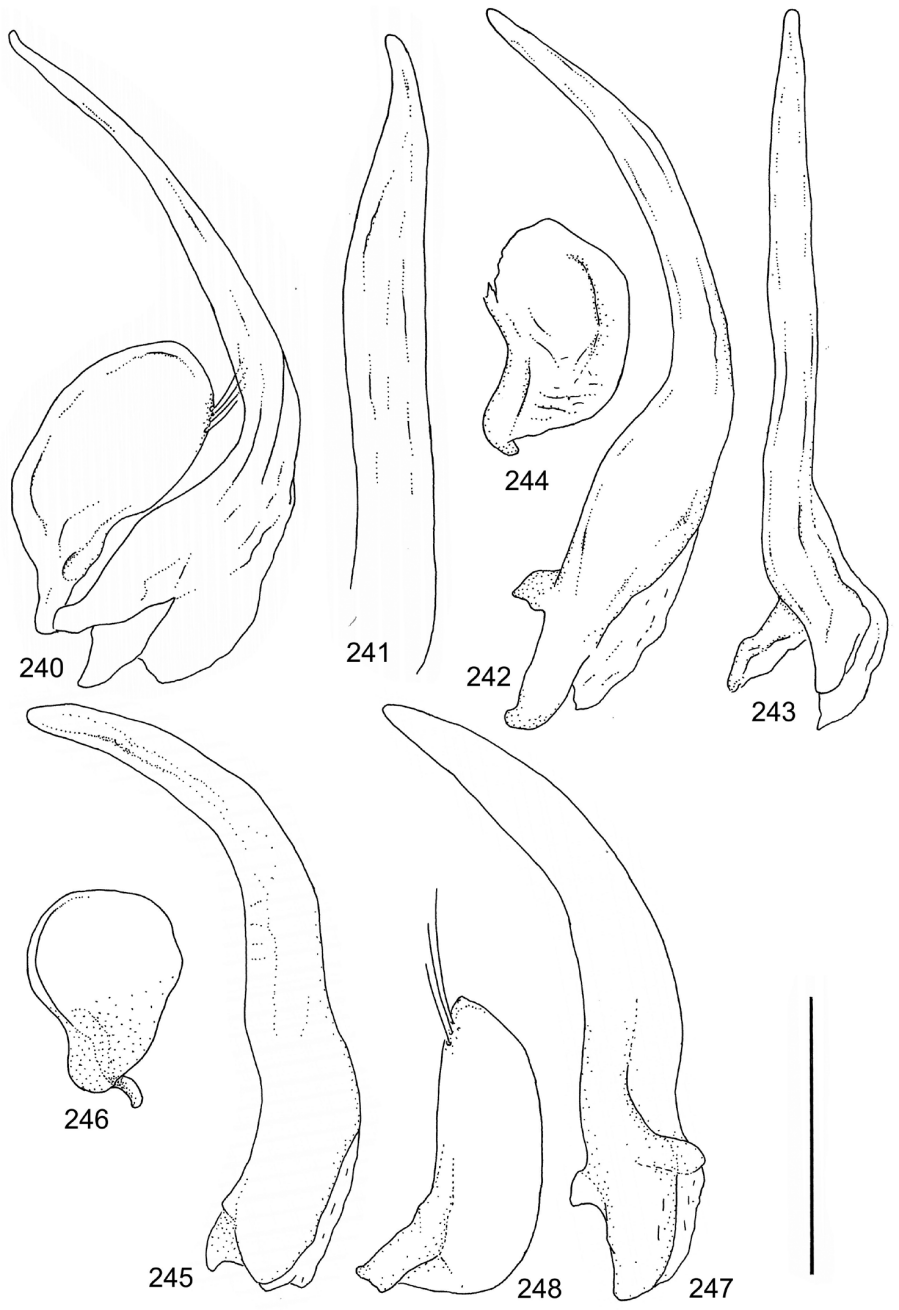
Male genitalia **240** *Laccophilus pseustes*, penis and paramere, lateral aspect, **241** penis, dorsal aspec **242** *Laccophilus seyrigi*, penis, lateral aspect **243** penis, dorsal aspect and **244** paramere **245** *Laccophilus isamberti*, penis, lateral aspect, and **246** paramere **247** *Laccophilus pictipennis*, penis, lateral aspect, and **248** paramere. Scale bar 0.5 mm.

**Figures 249–256.**
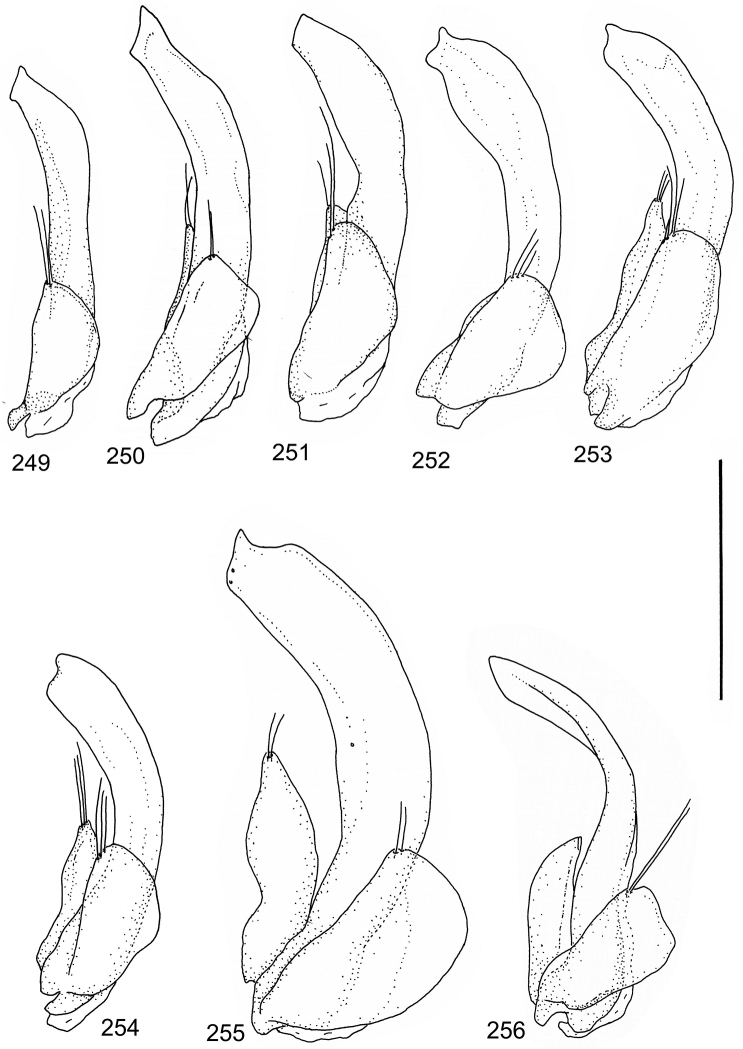
Male genitalia **249** *Laccophilus continentalis*, penis and paramere, lateral aspect **250** *Laccophilus posticus*, penis and paramere, lateral aspect **251** *Laccophilus inobservatus*, penis and paramere, lateral aspect **252** *Laccophilus simplicistriatus*, penis and paramere, lateral aspect **253** *Laccophilus taeniolatus*, penis and paramere, lateral aspect **254** *Laccophilus propinquus*, penis and paramere, lateral aspect **255** *Laccophilus complicatus*, penis and paramere, lateral aspect **256** *Laccophilus irroratus*, penis and paramere, lateral aspect. Scale bar 0.5 mm.

**Figures 257–260.**
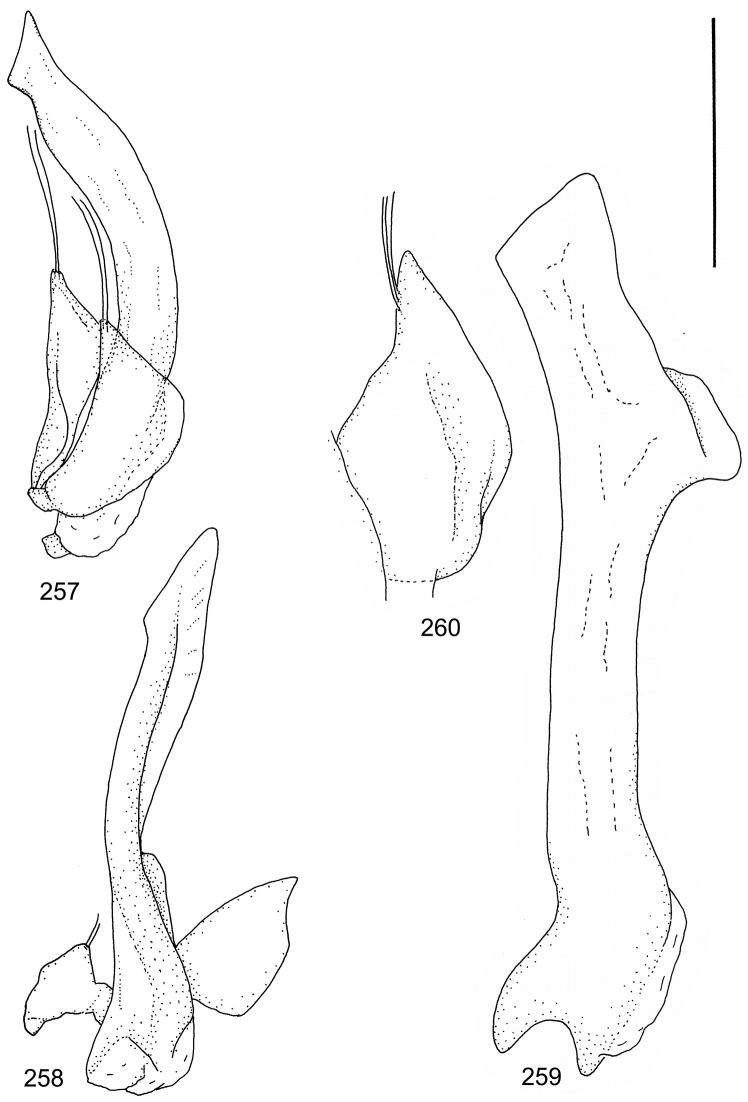
Male genitalia **257** *Laccophilus rivulosus*, penis and paramere, lateral aspect **258** *Laccophilus immundus*, penis and paramere, lateral aspect **259** *Laccophilus pellucidus*, penis, lateral aspect, and **260** paramere. Scale bar 0.5 mm.

**Figures 261–268.**
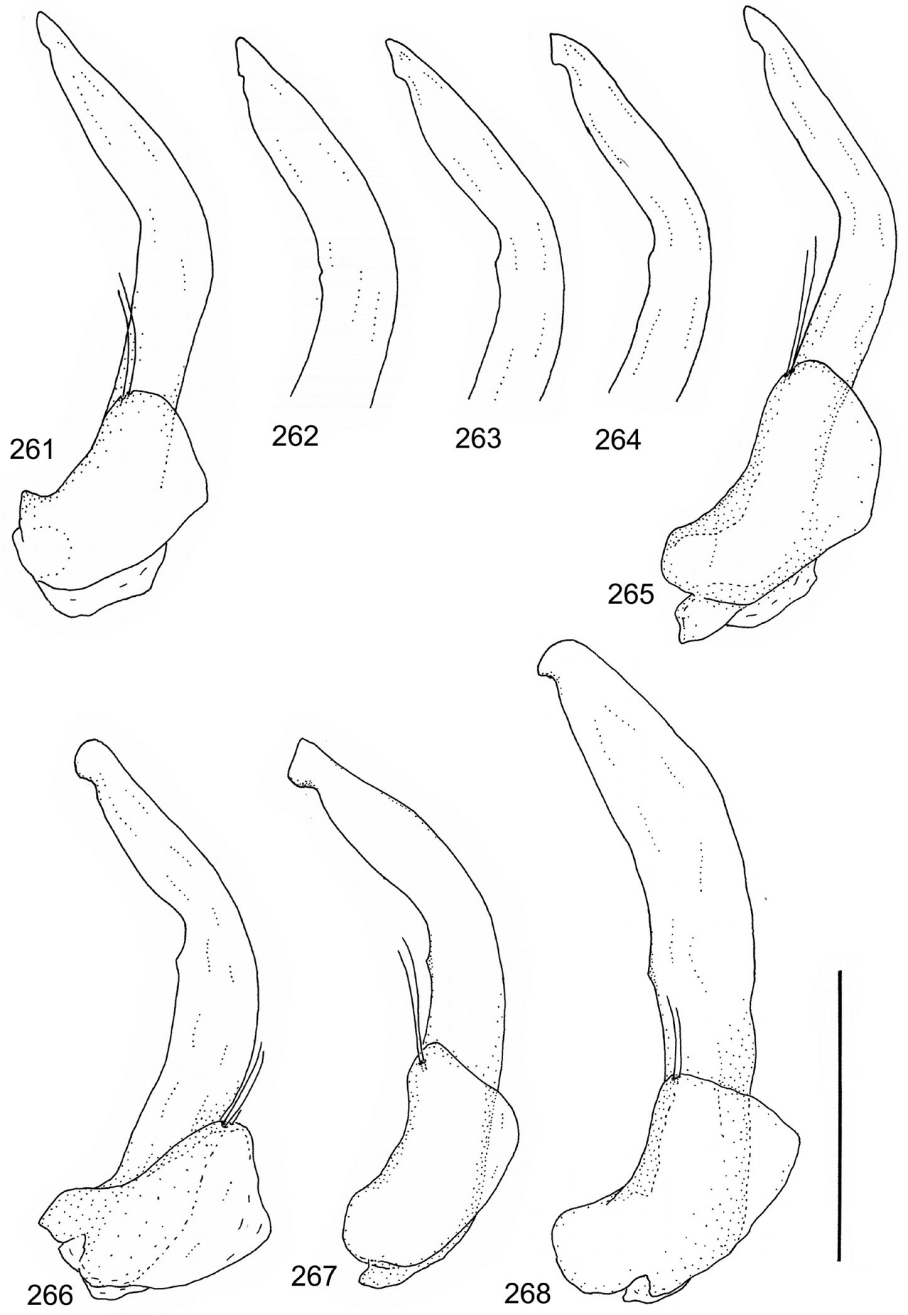
Male genitalia **261** *Laccophilus adspersus*, penis and paramere, lateral aspect, **262** (*Laccophilus livens*, type material), penis apex, lateral aspect **263** (*Laccophilus adspersus nigeriensis*, type material), penis apex, lateral aspect, and **264** (*Laccophilus adspersus sudanensis*, type material), penis apex, lateral aspect **265** *Laccophilus olsoufieffi*, penis and paramere, lateral aspect **266** *Laccophilus modestus*, penis and paramere, lateral aspect **267** *Laccophilus cryptos*, penis and paramere, lateral aspect **268** *Laccophilus nodieri*, penis and paramere, lateral aspect. Scale bar 0.5 mm.

**Figures 269–279.**
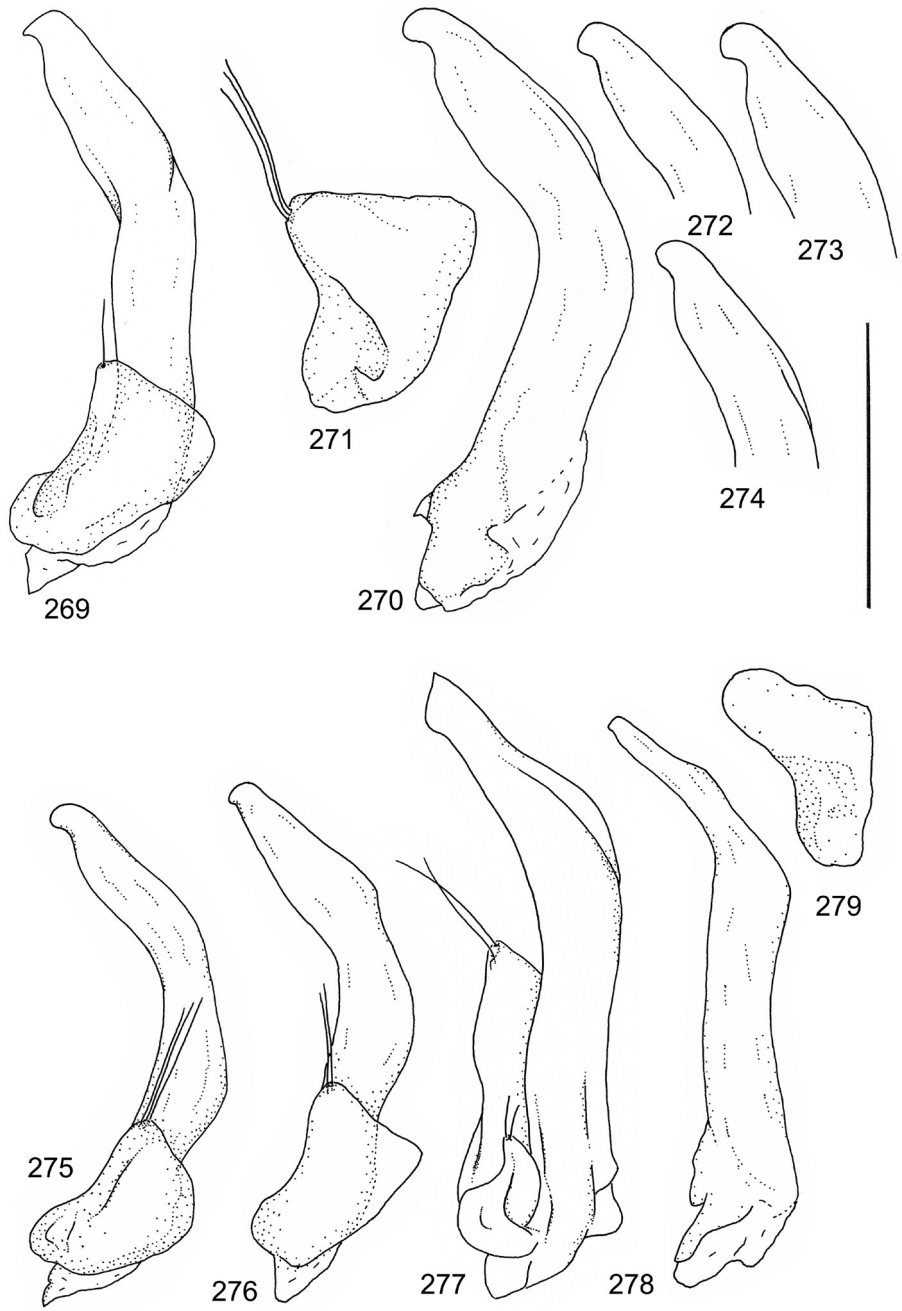
Male genitalia **269** *Laccophilus flaveolus*, penis and paramere, lateral aspect **270** *Laccophilus remex* (species complex), penis, lateral aspect and **271** paramere, **272** (*Laccophilus concisus*, type material), penis apex, lateral aspect **273** (*Laccophilus turneri*, type material), penis apex, lateral aspect, and **274** (*Laccophilus praeteritus*, type material), penis apex, lateral aspect **275** *Laccophilus turbatus*, penis and paramere, lateral aspect **276** *Laccophilus pallescens*, penis and paramere, lateral aspect **277** *Laccophilus trilineola*, penis and paramere, lateral aspect **278** *Laccophilus mediocris*, penis, lateral aspect, and **279** paramere. Scale bar 0.5 mm.

**Figures 280–288.**
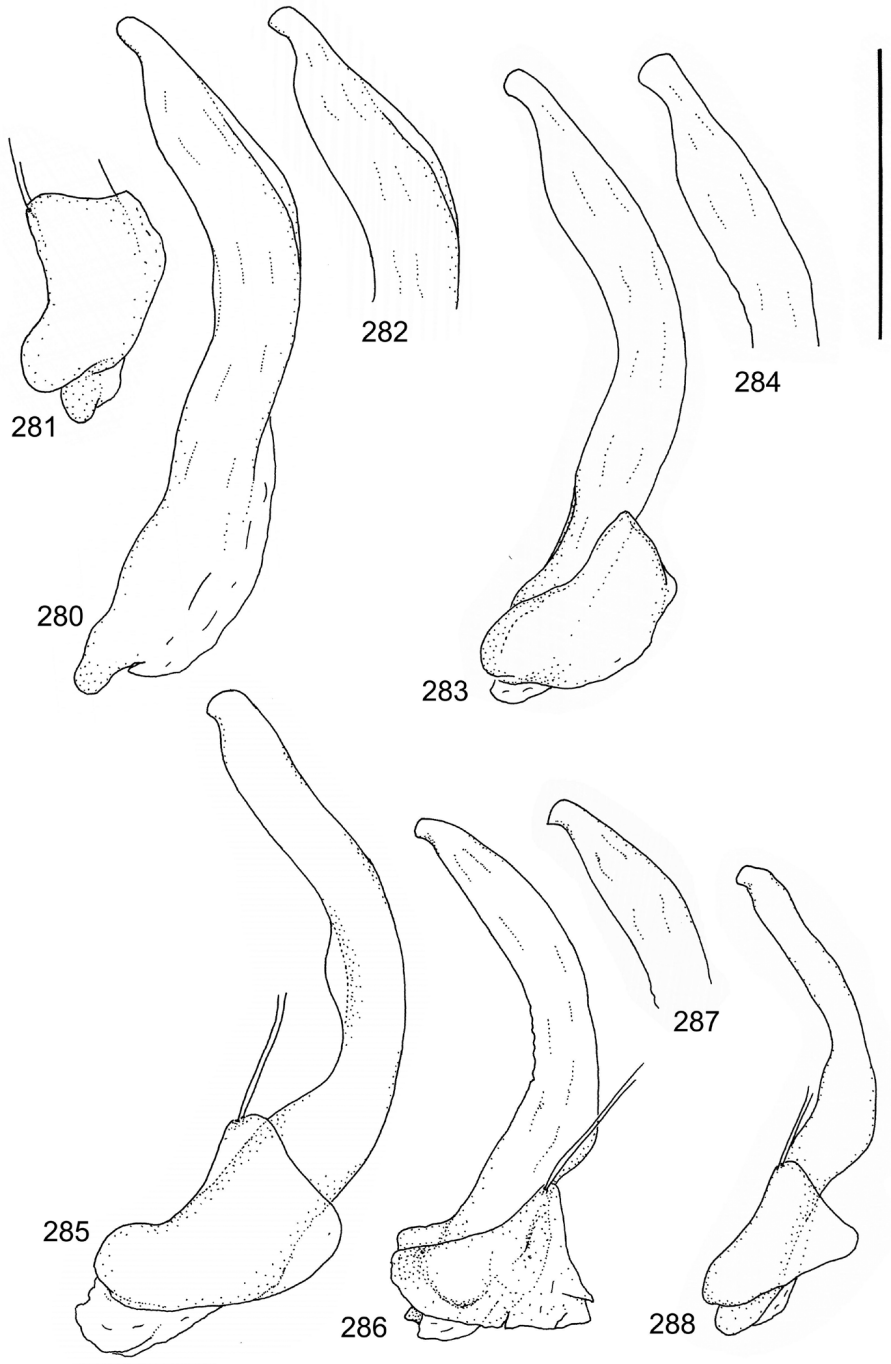
Male genitalia **280** *Laccophilus epinephes*, penis, lateral aspect **281** paramere and **282** (*Laccophilus castaneus*, type material) penis apex, lateral aspect **283** *Laccophilus saegeri*, penis and paramere, lateral aspect, and **284** (*Laccophilus comoensis*, type material) penis apex, lateral aspect **285** *Laccophilus enigmaticus*, penis and paramere, lateral aspect **286** *Laccophilus restrictus*, penis and paramere, lateral aspect, and **287** (variation), penis apex, lateral aspect **288** *Laccophilus bellus*, penis and paramere, lateral aspect. Scale bar 0.5 mm.

**Figures 289–300.**
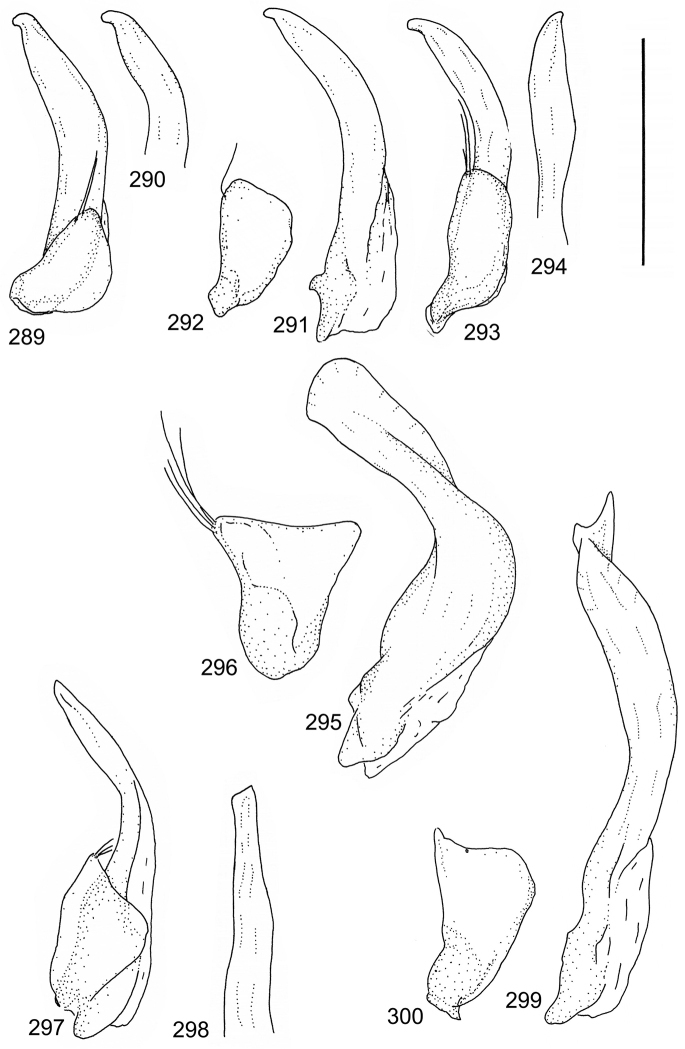
Male genitalia **289** *Laccophilus septicola*, penis and paramere, lateral aspect, and **290** (*Laccophilus alberticus*, type material) penis apex, lateral aspect **291** *Laccophilus pullatus*, penis apex, lateral aspect, and **292** paramere **293** *Laccophilus luteosignatus*, penis and paramere, lateral aspect, and **294** penis, dorsal aspect **295** *Laccophilus benoiti*, penis, lateral aspect, and **296** paramere **297** *Laccophilus addendus*, penis and paramere, lateral aspect, and **298** penis, dorsal aspect **299** *Laccophilus vermiculosus*, penis, lateral aspect, and **300** paramere. Scale bar 0.5 mm.

**Figures 301–308.**
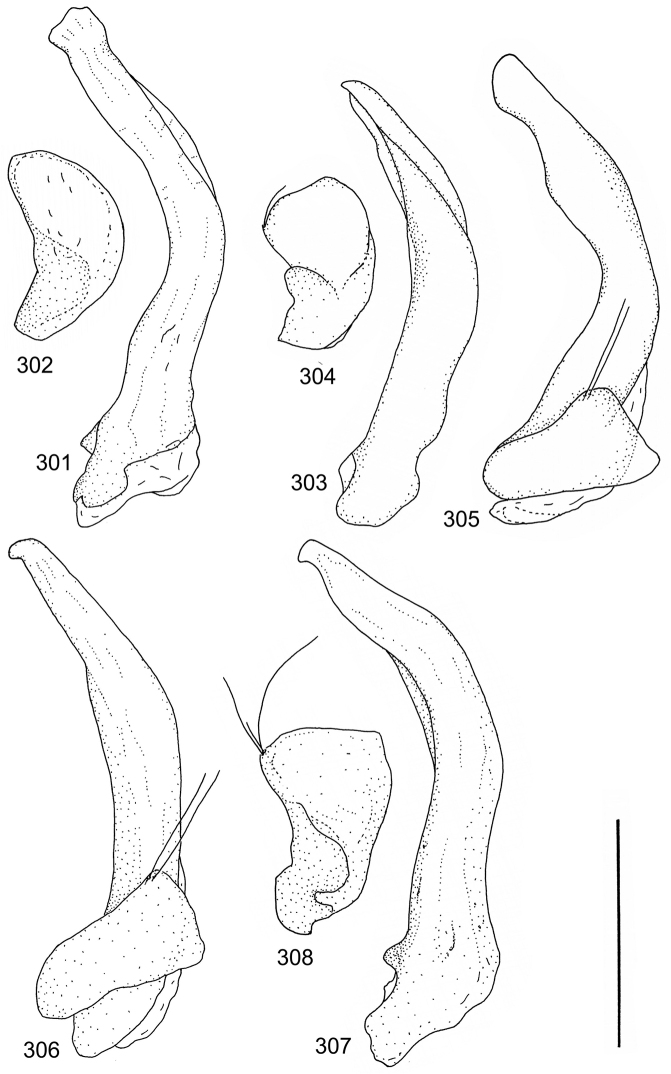
Male genitalia **301** *Laccophilus guignoti*, penis, lateral aspect, and **302** paramere **303** *Laccophilus guentheri*, penis, lateral aspect, and **304** paramere **305** *Laccophilus guineensis*, penis and paramere, lateral aspect **306** *Laccophilus bizonatus*, penis and paramere, lateral aspect **307** *Laccophilus pulcher*, penis, lateral aspect, and **308** paramere. Scale bar 0.5 mm.

**Figures 309–316.**
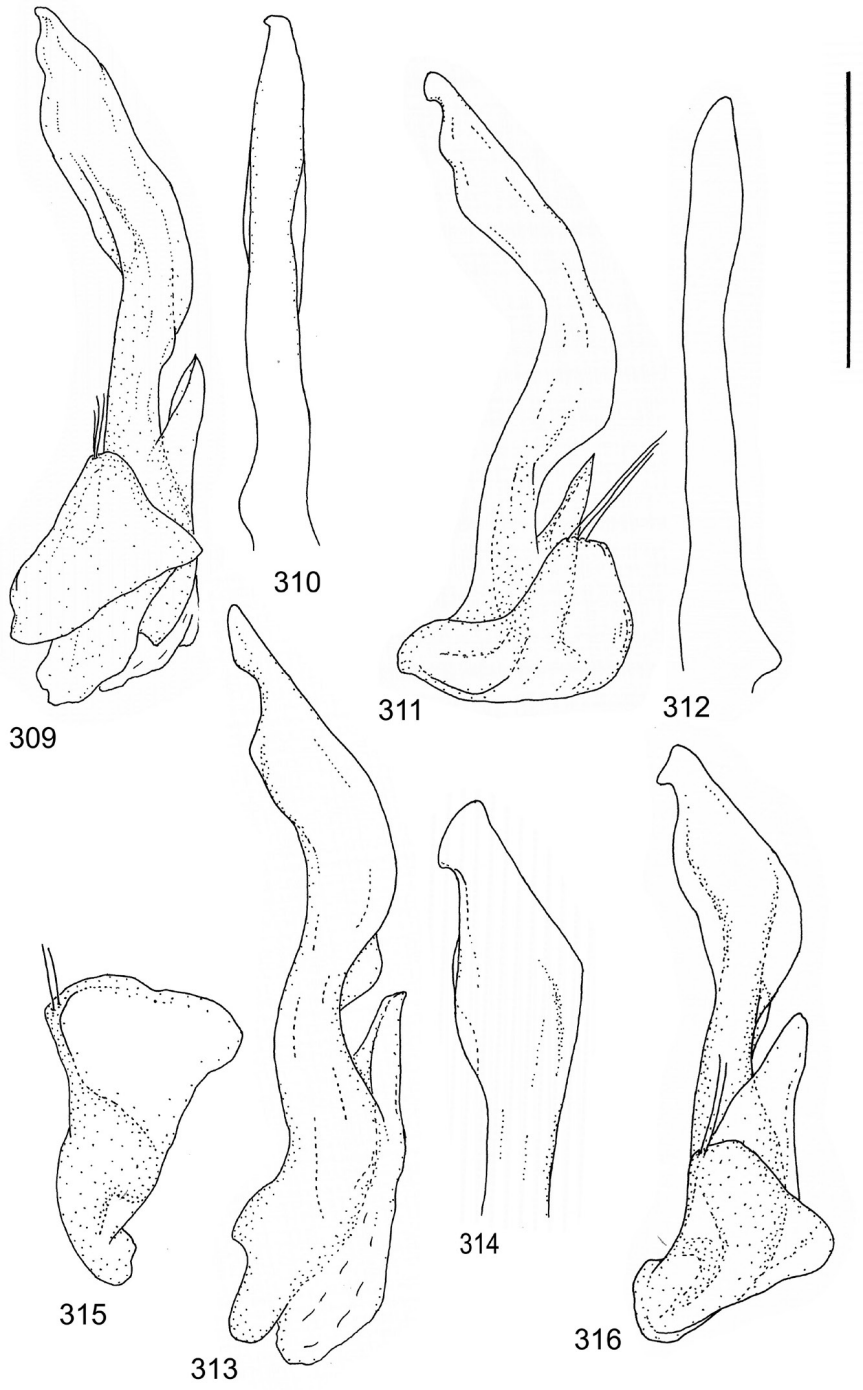
Male genitalia **309** *Laccophilus concettae*, penis and paramere, lateral aspect, and **310** penis, dorsal aspect **311** *Laccophilus biai*, penis and paramere, lateral aspect, and **312** penis, dorsal aspect **313** *Laccophilus deceptor*, penis, lateral aspect, **314** penis apex, view from other angle, and **315** paramere **316** *Laccophilus bilardoi*, penis and paramere, lateral aspect. Scale bar 0.5 mm.

**Figures 317–322.**
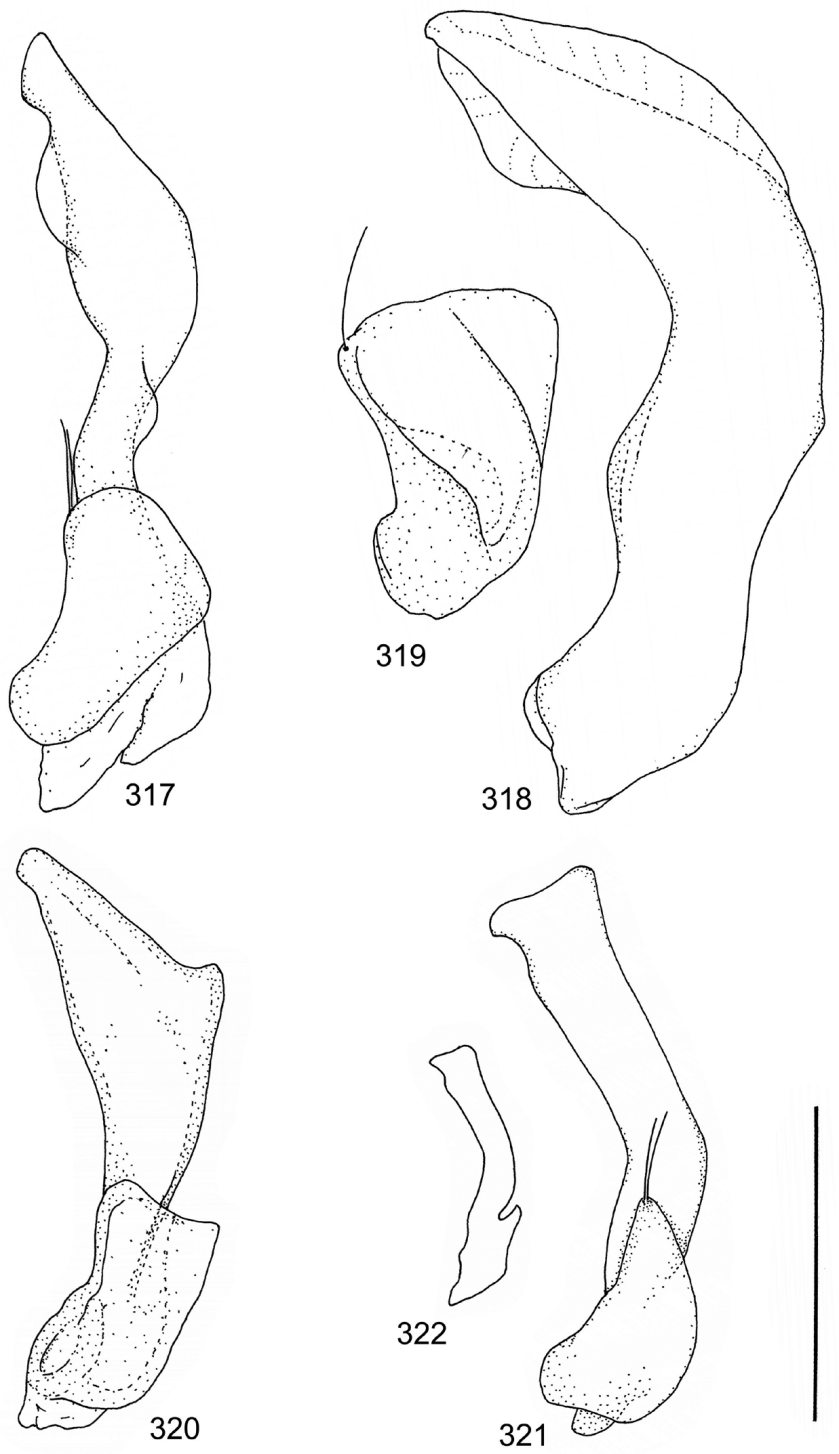
Male genitalia **317** *Laccophilus decorosus*, penis and paramere, lateral aspect **318** *Laccophilus tschoffeni*, penis, lateral aspect, and **319** paramere **320** *Laccophilus persimilis*, penis and paramere, lateral aspect **321** *Laccophilus poecilus*, penis and paramere, lateral aspect, and **322** supporting illustration of penis, lateral aspect. Scale bar 0.5 mm (excl. Fig. 322).

**Figures 323–331.**
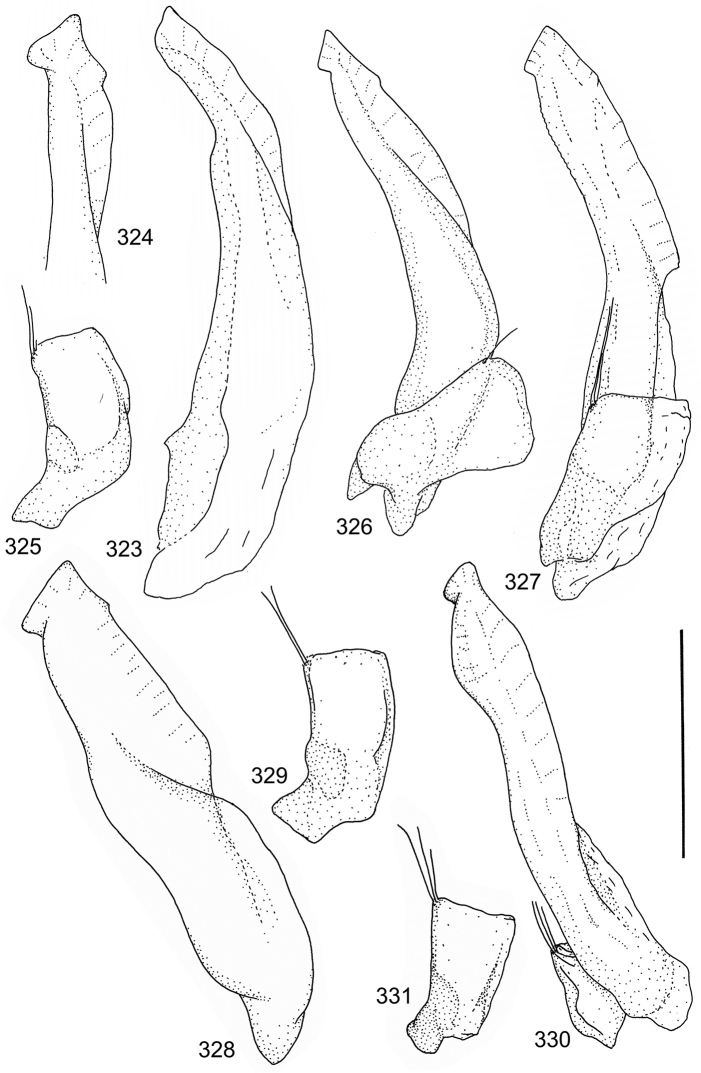
Male genitalia **323** *Laccophilus mutatus*, penis, lateral aspect, **324** penis apex, dorsal aspect, and **325** paramere **326** *Laccophilus quindecimvittatus*, penis and paramere, lateral aspect **327** *Laccophilus incrassatus*, penis and paramere, lateral aspect **328** *Laccophilus empheres*, penis, lateral aspect, and **329** paramere **330** *Laccophilus lateralis*, penis, lateral aspect, and **331** paramere. Scale bar 0.5 mm.

**Figures 332–342.**
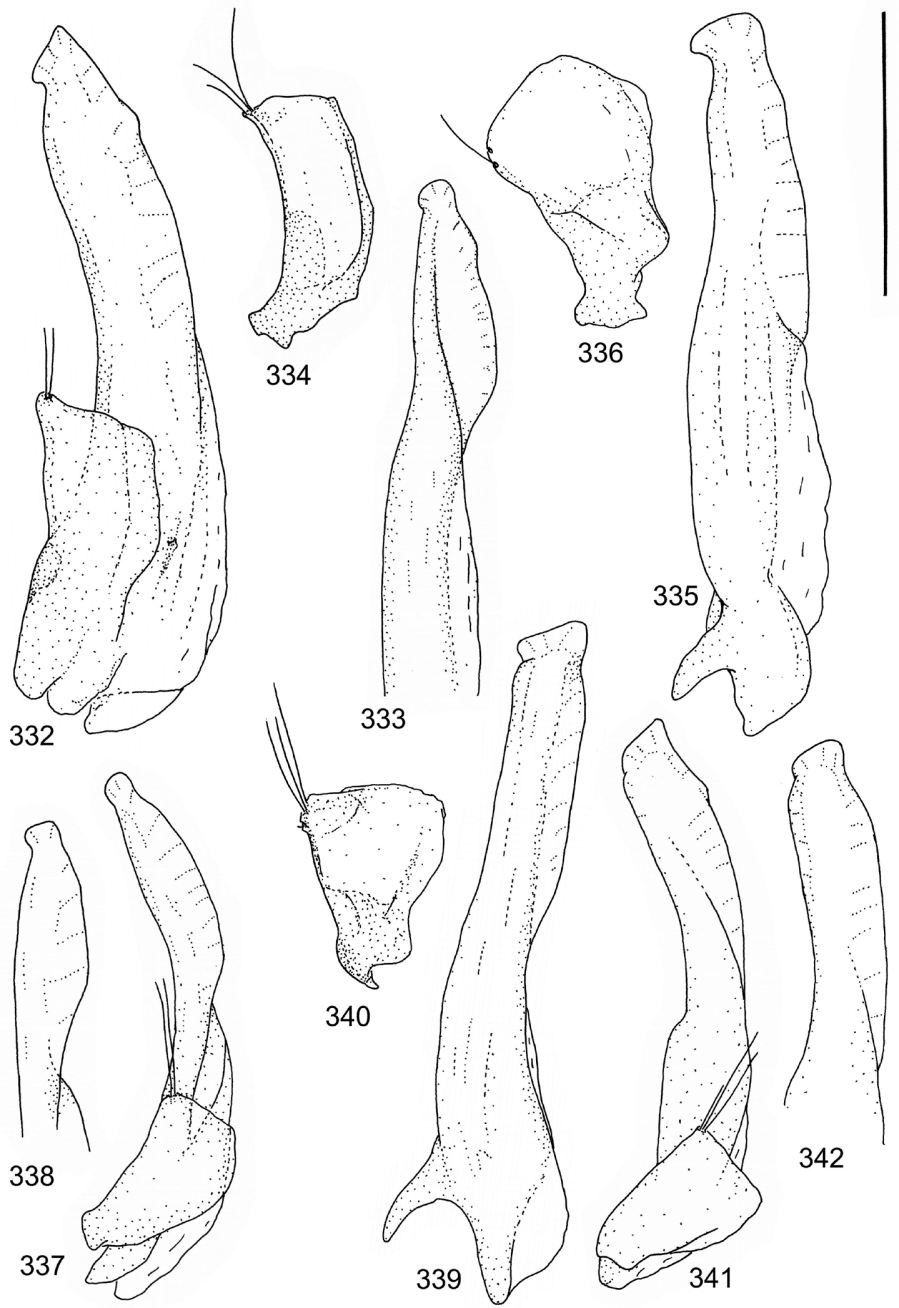
Male genitalia **332** *Laccophilus cyclopis*, penis and paramere, lateral aspect **333** *Laccophilus adjutor*, penis, lateral aspect, and **334** paramere **335** *Laccophilus necopinus*, penis, lateral aspect, and **336** paramere **337** *Laccophilus conjunctus*, penis and paramere, lateral aspect, and **338** penis apex, dorsal aspect **339** *Laccophilus brownei*, penis, lateral aspect, and **340** paramere **341** *Laccophilus contiro*, penis and paramere, lateral aspect, and **342** penis apex, dorsal aspect. Scale bar 0.5 mm.

**Figures 343–351.**
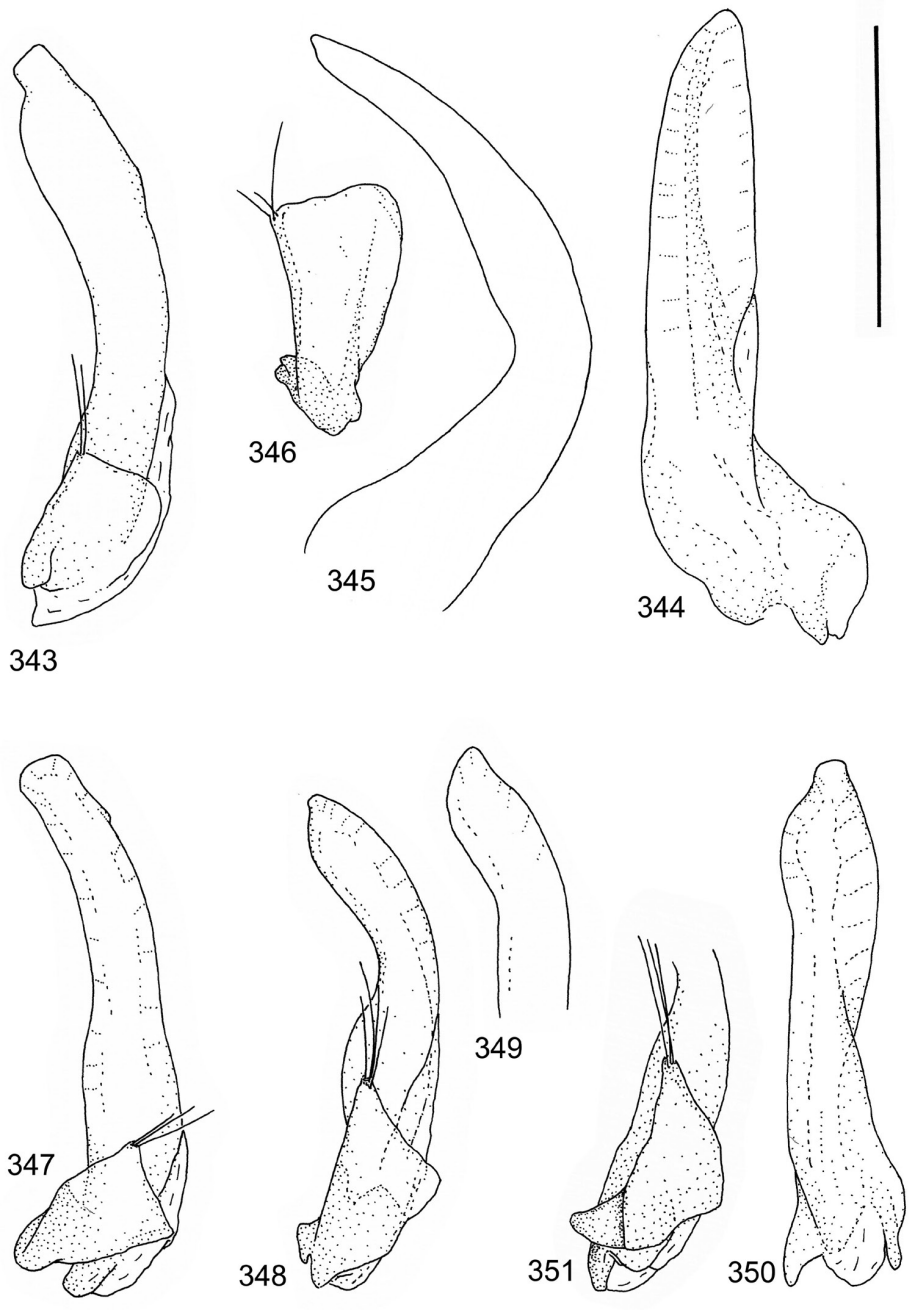
Male genitalia **343** *Laccophilus inconstans*, penis and paramere, lateral aspect **344** *Laccophilus grammicus*, penis, dorsal aspect, **345** penis, lateral aspect, and **346** paramere **347** *Laccophilus flavoscriptus*, penis and paramere, lateral aspect **348** *Laccophilus burgeoni*, penis and paramere, lateral aspect, and **349** penis apex, dorsal aspect **350** *Laccophilus lineatus*, penis, dorsal aspect, and **351** paramere and penis base, lateral aspect. Scale bar 0.5 mm.

**Figures 352–360.**
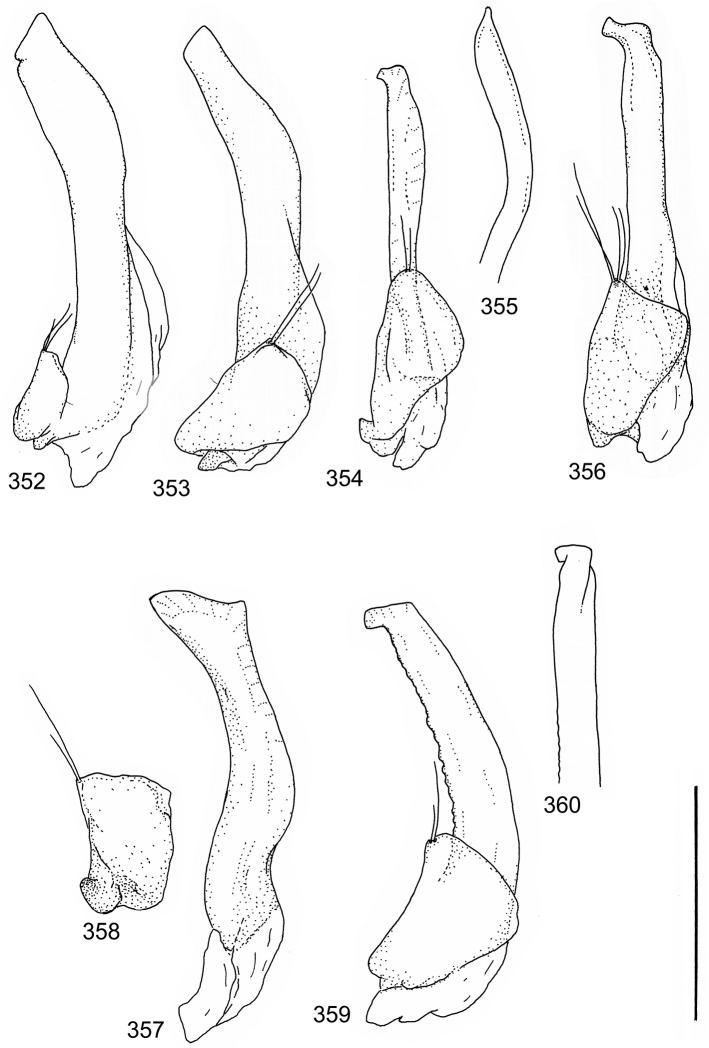
Male genitalia **352** *Laccophilus brancuccii*, penis and paramere, lateral aspect **353** *Laccophilus incomptus*, penis and paramere, lateral aspect **354** *Laccophilus secundus*, penis and paramere, lateral aspect, and **355** penis apex, view from other angle **356** *Laccophilus australis*, penis and paramere, lateral aspect **357** *Laccophilus desintegratus*, penis, lateral aspect, and **358** paramere **359** *Laccophilus luctuosus*, penis and paramere, lateral aspect, and **360** penis apex, dorsal aspect. Scale bar 0.5 mm.

**Figures 361–372.**
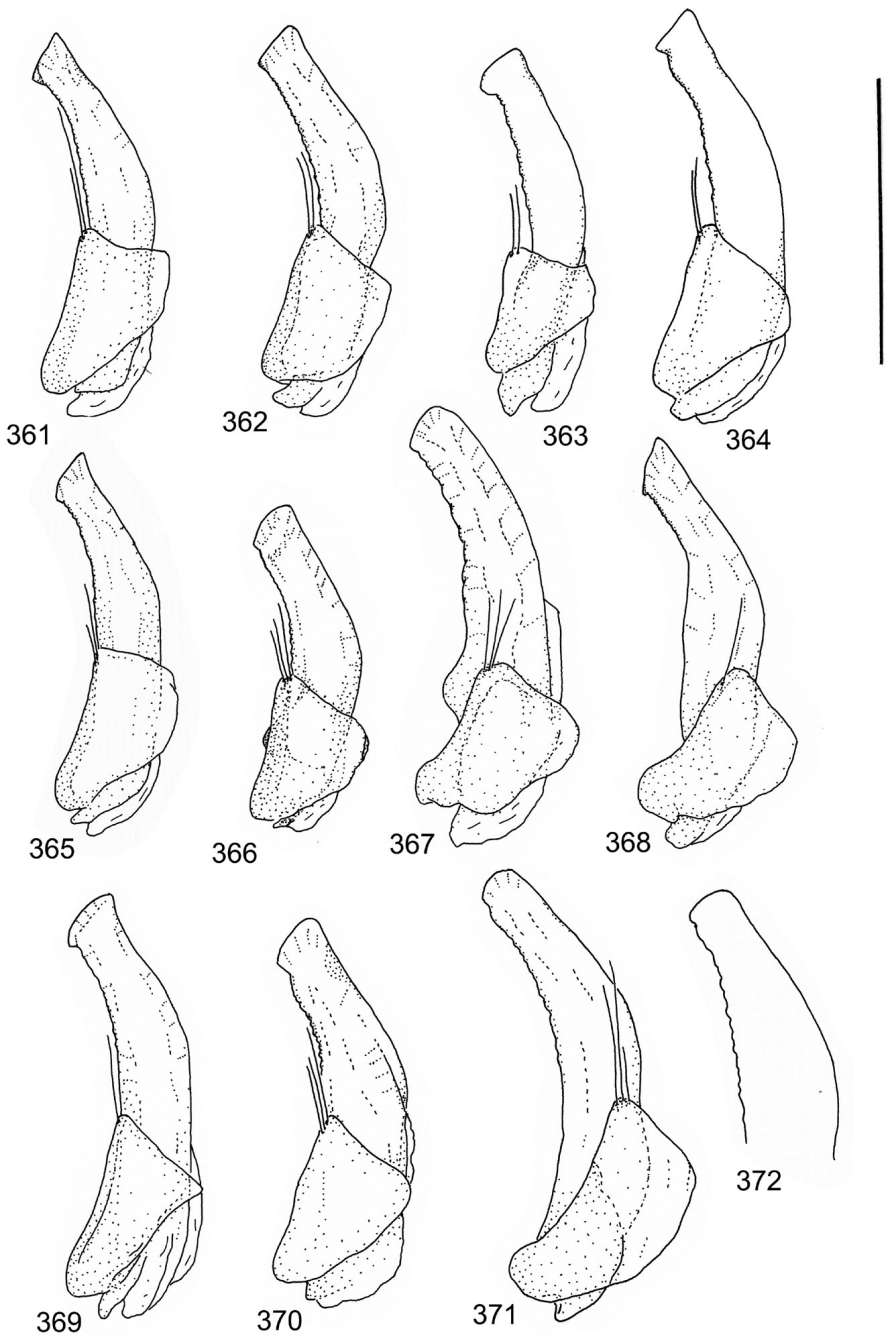
Male genitalia **361** *Laccophilus inornatus*, penis and paramere, lateral aspect **362** *Laccophilus canthydroides*, penis and paramere, lateral aspect **363** *Laccophilus minimus*, penis and paramere, lateral aspect **364** *Laccophilus eboris*, penis and paramere, lateral aspect **365** *Laccophilus leonensis*, penis and paramere, lateral aspect **366** *Laccophilus villiersi*, penis and paramere, lateral aspect **367** *Laccophilus melas*, penis and paramere, lateral aspect **368** *Laccophilus livingstoni*, penis and paramere, lateral aspect **369** *Laccophilus insularum*, penis and paramere, lateral aspect **370** *Laccophilus garambanus*, penis and paramere, lateral aspect **371** *Laccophilus flavopictus*, penis and paramere, lateral aspect, and **372** (*Laccophilus segmentatus* type material), penis apex, lateral aspect. Scale bar 0.5 mm.

Figs 200–201 , 370 , 522 , 576

Laccophilus garambanus Guignot 1958: 7 (original description, faunistics); Nilsson 2001: 244 (catalogue, faunistics); Nilsson 2015: 212 (catalogue, faunistics).

##### Type locality.

Zaire: Parc National Garamba.

##### Type material studied

(7 exs.). Holotype: male: “Holotypus / Congo Belge, P.N.G. Miss. H. De Saeger II/gd/14s, 4-VIII-1951 Réc. H. De Saeger, 2209 / Coll. Mus. Congo (ex. coll. I.P.N.C.B.) / Guignot det., 1957 *Laccophilus garambanus* sp. n.” (MRAC; immature specimen). – Paratype: male and female: “Congo Belge, P.N.G. Miss. H. De Saeger II/gc/10, 28-VIII-52 H. De Saeger. 3987 / Paratype / F. Guignot det., 1959 *Laccophilus garambanus* sp. n.” (1 ex. IRSNB; habitus in Fig. 522); same data, but “II/gd/11, 24-VI-1952, 3963” (1 ex. IRSNB); same data, but “II/fd/14, 23-VIII-52, 3966 / F. Guignot det., 1958 *Laccophilus inornatus garambanus* Guignot, paratype” (1 ex. IRSNB); same data, but “II/fd/12, 10-III-1952, 3180” (3 exs. IRSNB); same data, but “II/gd/10, 14-IX-1952, 4099” (1 ex. IRSNB).

##### Additional material studied

(11 exs.). **Zaire**: “PNG II/fd/12, 10.3. 1952, 3180” (1 ex. MRAC); “P.N.G. II/gd/10, 14-IX-52, 4099” (3 exs. IRSNB); “Tshuapa, Mbandaka ca. 0.03N - 18.28E, 1964 Stam” (2 exs. RMNH); “Tshuapa, Mbandaka ca. 0.03N - 18.28E, 3-4.4. 1964 Stam” (1 ex. RMNH); “Thsuapa-Mbandaka ca. 0.03N - 18.28E, a.l. 1964 Stam (1 ex. RMNH); “Quqilhatville 3-4.4. 1963 Stam 3-4.4. 1963 / at light (2 exs. RMNH); “Quqilhatville 20-21.1. 1962 Stam 3-4.4. 1962 / at light (1 ex. MZH).

##### Specimen with uncertain determination.

**Cameroon**: “Ngoundéré 27.7. 1969” (1 female ex. NHMB).

##### Diagnosis.

*Laccophilus garambanus* is characterized by distinct, although somewhat variable, elytral colour pattern. It is distinguished from other species in this species group by the appearance of male genitalia (penis is quite robust, short, apical external outline almost straight), besides characteristic, elytral colour pattern.

##### Description.

Body length 2.9–3.2 mm, width 1.6–1.8 mm. Elytral colour pattern variable (Fig. 522); subbasal, pale markings sometimes confluent and form a transverse pale marking. Preapical pale spots also variable in size; often confluent.

Head: Pale ferrugineous, partly often slightly darker but lacks clearly delimited, dark area. Submat, finely microsculptured; reticulation simple and dense. At eyes, finely and irregularly punctate.

Pronotum: Pale ferrugineous, medially with large area slightly darker; ferrugineous (delimitation of area generally vague). Submat to rather shiny, finely microsculptured; reticulation fine and dense; indistinct traces of double reticulation discernible. When discernible large meshes contain 3–6 small meshes. Almost impunctate.

Elytra: Dark ferrugineous to blackish ferrugineous, with variable, ferrugineous to pale ferrugineous markings (Fig. 522). One female (uncertain determination) has slightly paler elytra. Submat, rather shiny, finely microsculptured; reticulation fine, rather distinct. Double reticulation indistinct and fragmentary. Longitudinal areas with fine, rather indistinct and irregular punctures. Lateral, pre-apical furrow shallow, finely pubescent.

Ventral aspect: Pale ferrugineous to ferrugineous, abdomen slightly darker in comparison with rest of ventral aspect. Submat to rather shiny, extensively with very fine microsculpture. Almost impunctate. Stridulatory apparatus fine but clearly discernible; consisting of about 25 narrow but quite long ridges. Apex of prosternal process slender, somewhat extended and pointed. Apical ventrite as in Fig. 200. Transversely located, shallow furrows on metacoxal plates fine (discernible in anterior half). Basal ventrites with fine, distinctly reduced, curved striae.

Legs: Pro- and mesotarsus slightly enlarged, with fine suckers.

Male genitalia: Penis quite robust, short, apical external outline almost straight; extreme external apex broadly rounded (Fig. 370).

Female: Apical ventrite simple (Fig. 201). Pro- and mesotarsus slender.

##### Distribution.

Zaire (Fig. 576). Uncertain record from Cameroon.

##### Collecting circumstances.

Almost unknown. Sampled at light collection.

#### Laccophilus flavopictus

Taxon classificationAnimaliaColeopteraDytiscidae

Régimbart, 1889

Figs 202–203 , 371–372. 523, 577

Laccophilus flavopictus Régimbart 1889: 53 (original description, faunistics); Régimbart 1895: 146 (description, faunistics); Zimmermann 1920a:18 (catalogue, faunistics); Guignot 1946c: 262, 312 (discussion); Guignot 1948: 14 (faunistics); Guignot 1956c: 321 (discussion); Legros 1958: 211 (faunistics); Guignot 1959a: 537, 543 (description, faunistics, discussion); Nilsson and Persson 1993: 79, 94 (faunistics, biology); Nilsson 2001: 243 (catalogue, faunistics); Nilsson 2015: 211 (catalogue, faunistics).
Laccophilus bergeri Guignot 1953b: 234, 236 (original description, faunistics); Guignot 1959a: 534, 537, 542 (description, faunistics); Omer-Cooper 1965: 76 (description, faunistics); Omer-Cooper 1967: 58 (discussion, synonymy *Laccophilus segmentatus* and *Laccophilus bergeri*); Nilsson 2001: 240 (catalogue, faunistics); Nilsson 2015: 209 (catalogue, faunistics). **New synonym.**
Laccophilus segmentatus Omer-Cooper 1957: 12, 90 (original description, faunistics); Omer-Cooper 1958a: 59 (faunistics); Omer-Cooper 1958b: 37, 38, 39, 40, 42 (description, faunistics, biology, discussion); Omer-Cooper 1965: 80 (list synonym *Laccophilus bergeri* Guignot); Omer-Cooper 1967: 58 (discussion, list, synonymy); Nilsson 2001: 240 (catalogue, faunistics, list, synonymy); Nilsson 2015: 209 (catalogue, faunistics, list, synonymy, *Laccophilus bergeri*). **New synonym.**

##### Type localities.

*Laccophilus flavopictus*: Angola: Humpata.

*Laccophilus bergeri*: Zaire (DRC): Elisabethville.

*Laccophilus segmentatus*: South Africa: Transvaal, Ermelo.

##### Type material studied

(6 exs.). *Laccophilus flavopictus*: Holotype (monotypy): male: “P.Y. v d Kellen Humpata Afr. trop / *Laccophilus flavopictus* sp. n. type / type / *flavopictus* Régimbart sp. n.” (RMNH).

*Laccophilus bergeri*: Holotype: male: “Holotypus / Coll. Mus. Congo Elisabethville lumière XI-50/VI-51 Ch. Seydel / Type / R. Det. J. 6182 / Guignot det. 1953 *Laccophilus bergeri* Guign. Type, male” (MRAC; habitus in Fig. 523). – Paratypes: Same data as holotype, but: “Paratypus female / R. Det. L. 6182” (1 ex. MRAC); same as holotype, but: “Allotypus female / (A la lumière) X/XI. 1950/R. Det. K” (1 ex. MRAC); same as holotype, but: “Paratypus male / (A la lumière) X/XI. 1950” (1 ex. MRAC).

*Laccophilus segmentatus*: Holotype: male: “Type, male-symbol / Type / Transvaal, sluggish stream nr Ermelo 7. 12. 1948 J. O-C / Brit. Mus. 1957-660 / *Laccophilus* sp. n. J. Balfour-Browne det. / *Laccophilus segmentatus* O-C.” (BMNH).

##### Additional material studied

(25 exs.). **Zaire**: “Elisabethville (à la lumière) 1957-1958 Seydel” (1 ex. MRAC). – **Zambia**: “Mwinilunga District, Ikelenge, nr Kalene, Zambezi Rapids / Pinhey 3.5. 1963 M.V. light trap” (4 exs. BMNH, 1 ex. MZH); “NC, Mkushi env. E 16-18.12. 2004 Snizek leg.” (1 ex. NHPC); “Northern Province 15 km E Luwingu 3-4.12. 2007 1400 m Kudrna Jr. leg. / *Laccophilus bergeri* Guign. det. Pederzani” (2 exs. CFP); “100 km SW Serenje 7.12. 2002 Kantner” (1 ex. NHMB). – **Malawi**: “Dedza Sept. 1948 O-C. / small stream” (1 ex. AMGS); “Dedza Sept. 1948 O-C.”(4 exs. AMGS); “Dedza dam, lower Lilongwe rd 29.IX. 1948” (1 ex. AMGS); “Dedza Hotel, dam 29.IX. 1948” (1 ex. AMGS). – **Zimbabwe**: “Inyanga N. 1948 JOC. / *Laccophilus segmentatus* O-C. = *Laccophilus bergeri* Guign. Det. J. Omer-Cooper” (1 ex. AMGS); “Stream Rusapi 13.XI. (?) 1948 / *Laccophilus segmentatus* O-C. det. J. Omer-Cooper” (1 ex. AMGS); “Marandellas 2.11. 1948 J. Omer-Cooper” (3 exs. MNHN). – **Swaziland**: “Mbabane 5.XII. 1948 J.O.C. / *Laccophilus segmentatus* O-C. = *Laccophilus bergeri* Guign. Det. J. Omer-Cooper” (1 ex. AMGS). – **South Africa**: “Natal Durban Umgeni Tragardh” (1 ex. AMGS); *“Laccophilus* sp. n. J. Balfour-Browne / Paratype / Vlei nr Wasserman’s Beacon 6.12. 1948 / *Laccophilus segmentatus* O-C.” (1 ex. AMGS).

##### Comments on synonymy.

Male holotypes of the three involved taxa, *Laccophilus flavopictus*, *Laccophilus bergeri*, and *Laccophilus segmentatus*, have been examined and found to be conspecific. Because *Laccophilus flavopictus* is the oldest available name, it is also the valid name of the species.

##### Diagnosis.

*Laccophilus flavopictus* is characterized by the following combination of features: Elytra almost black to blackish ferrugineous; basally provided with a quite narrow but distinct, transverse, pale ferrugineous marking and by non-hooked penis apex. Externally it resembles of some species not located in this species group, eg. *Laccophilus luctuosus* and *Laccophilus secundus*. Presence of a stridulation apparatus and shape of male genitalia, however, shows that *Laccophilus flavopictus* belongs to another species group than these two species.

##### Description.

Body length 3.4–3.7 mm, width 1.9–2.0 mm. Colour pattern generally distinct and exhibits only minor variation; sometimes in part, dark areas with somewhat vague delimitation (Fig. 523).

Head: Pale ferrugineous to ferrugineous; posteriorly at pronotum darker; dark ferrugineous. Slightly mat, finely microsculptured; reticulation double (in part indistinct). Large meshes when discernible, contain 2–5 small meshes. At eyes and in a narrow, transverse area between eyes with very fine, somewhat irregular punctures; medially punctures indistinct and hardly visible.

Pronotum: Pale ferrugineous, frontally and basally with a medial, dark marking (Fig. 518). Submat to rather shiny, fienly microsculptured; reticulation in part double (partially indistinct). Large meshes generally contain 2–5 small meshes. At margins with fine, in part indistinct and sparse punctures.

Elytra: Blackish ferrugineous to dark ferrugineous, with distinct pale ferrugineous markings (Fig. 523). Submat to rather shiny, finely microsculptured. Reticulation in part double but double reticulation somewhat indistinct; large meshes, when discernible, contain 2–5 small meshes. Punctation very fine and form three rather irregular, longitudinal areas.

Ventral aspect: Pale ferrugineous, abdominal ventrites at least in part darker, ferrugineous to dark ferrugineous. Stridulatory apparatus consists of about 30 fine ridges. Submat, finely microsculptured. Abdomen provided with fine, slightly sparse, curved striae. Apical ventrite provided with a sharp knob on one side (other side lacks knob) (Fig. 202). Prosternal process narrow, posteriorly slightly extended and apically pointed.

Legs: Pro- and mesotarsus slender, somewhat extended and provided with suckers.

Male genitalia: Penis comparatively long, slightly angled; apical outline rounded to almost straight (Figs 371–372).

Female: Externally as male but apical ventrite almost symmetric, lacking sharp knob (Fig. 203), Pro- and mesotarsus slender.

##### Distribution.

Zaire, Angola, Zambia, Malawi, Zimbabwe, South Africa, Swaziland (Fig. 577). Only verified records are used in the map.

##### Collecting circumstances.

Omer-Cooper (1958b) reports the species from different kinds of water bodies, as ponds, pools, streams, bogs and an artificial dam. Often in association with some vegetation as water lilies, rushes, weeds etc. Collected at light.

### Species group 17 (*Laccophilus laeticulus* group)

**Diagnosis.** Small to medium sized species; length of body 2.8–3.4 mm, width 1.6–1.9 mm.

Body shape oval; body dorsoventrally flattened. All recognized species with dark ferrugineous elytra, provided with distinct pale ferrugineous, basal, transverse marking. Posteriorly on elytra with variable, pale spots or irregular, pale stripes (Fig. 525). Pronotum, except for small, basal, dark marking, and head almost entirely pale ferrugineous. Body with dorsal microsculpture double; reticulation divided into two size-classes; meshes of microsculpture in part vague.

Prosternal process slender, posteriorly extended, apically pointed. Apical ventrite modified; posterior part on both side of midline excavated and medial part forms a backwards extending process. Apical ventrite of male provided with asymmetrical knob on one side of ventrite (Fig. 207). Metacoxal process ends abruptly; lacks posterior extension (Fig. 6). Fine stridulation apparatus on metacoxal plates but it is is weakly developed, and function in sound production is unclear.

Paramere with slight modification; apically distinctly enlarged (Fig. 376). Ground plan of penis similar in all species; apical half narrows gradually either to a slender or a broad apex. A small dent is discernible on apex of inner curvature of penis (Fig. 377).

**Species composition and distribution.** Three species are recognized in this species group. Distribution covers Africa south of Sahara and Madagascar.

#### Key to species (males)

**Table:** 

1	Penis narrows gradually towards slender apex (Fig. 373)	***Laccophilus laeticulus*** (p. 257)
–	Penis narrows abruptly at broad apex (Fig. 375)	**2**
2	Penis, lateral aspect, quite straight; apical dent indistinct (Fig. 375)	***Laccophilus occidentalis*** (p. 259)
–	Penis, lateral aspect, curved; apical dent clearly discernible (Fig. 377)	***Laccophilus transversovittatus*** (p. 260)

#### Laccophilus laeticulus

Taxon classificationAnimaliaColeopteraDytiscidae

Régimbart, 1895

**Figures 373–377.**
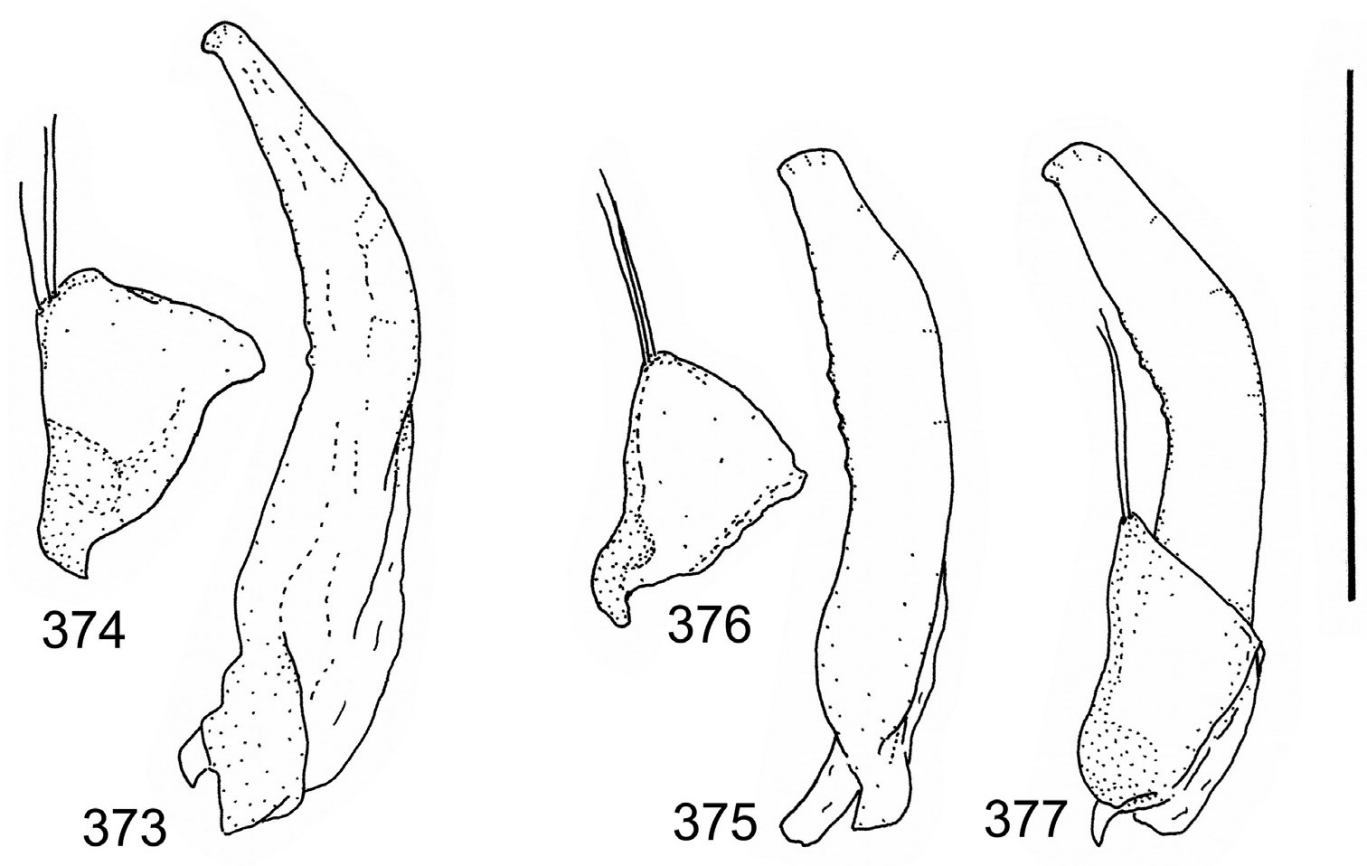
Male genitalia **373** *Laccophilus laeticulus*, penis, lateral aspect, and **374** paramere **375** *Laccophilus occidentalis*, penis, lateral aspect, and **376** paramere **377** *Laccophilus transversovittatus*, penis and paramere, lateral aspect. Scale bar 0.5 mm.

**Figures 378–389.**
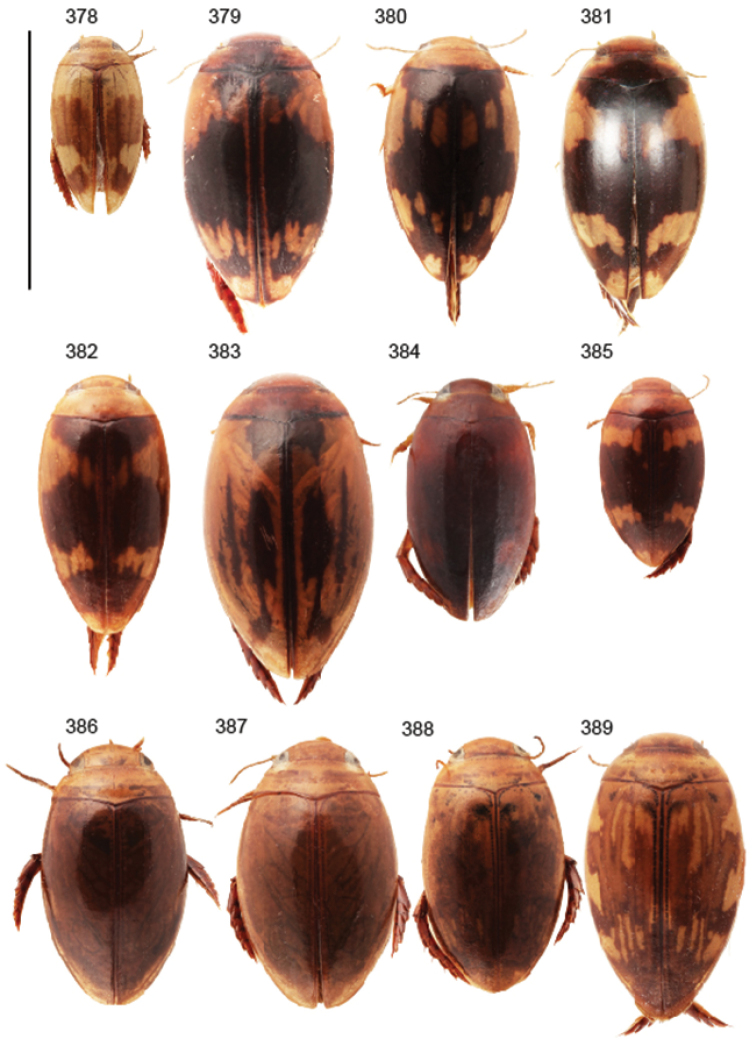
Dorsal habitus **378** *Laccophilus tavetensis* **379** *Laccophilus grossus* **380** *Laccophilus rocchii* **381** *Laccophilus morondavensis* **382** *Laccophilus productus* **383** *Laccophilus mirabilis* **384** *Laccophilus ferrugo* **385** *Laccophilus ruficollis* **386** *Laccophilus hyalinus* **387** *Laccophilus demoflysi* **388** *Laccophilus minutus* **389** *Laccophilus mateui*. Scale bar 5.0 mm.

**Figures 390–401.**
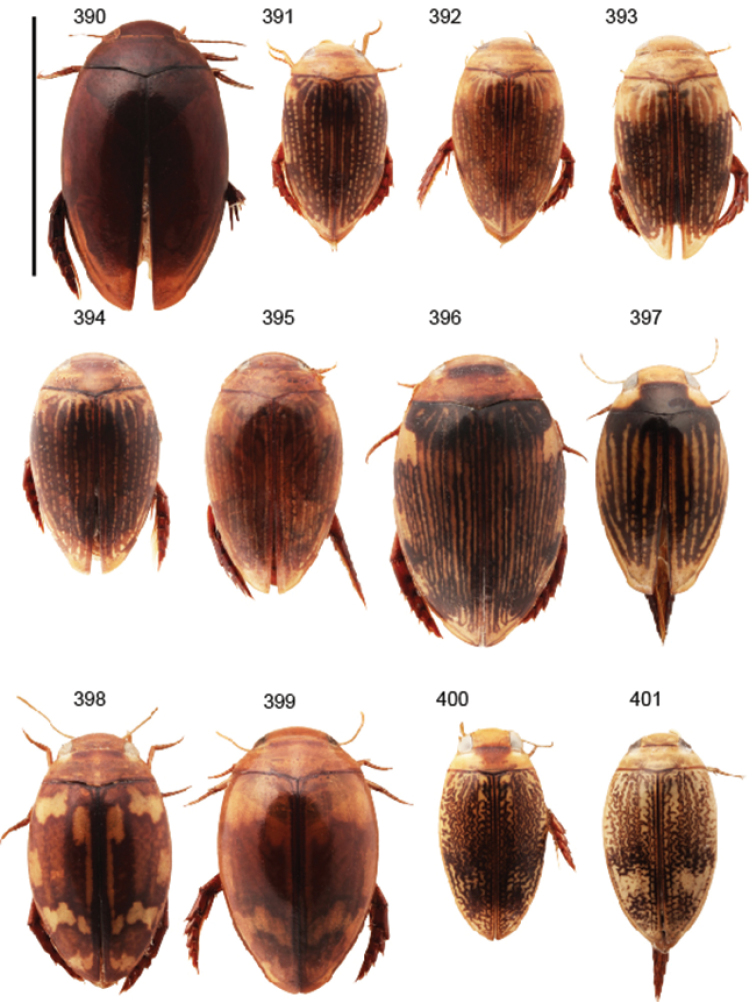
Dorsal habitus **390** *Laccophilus sordidus* **391** *Laccophilus comes* **392** *Laccophilus alluaudi* **393** *Laccophilus furthi* **394** *Laccophilus tigrinus* **395** *Laccophilus pseustes* **396** *Laccophilus seyrigi* **397** *Laccophilus isamberti* **398** *Laccophilus pictipennis* **399** *Laccophilus pictipennis* **400** *Laccophilus continentalis* **401** *Laccophilus continentalis*. Scale bar 5.0 mm.

**Figures 402–413.**
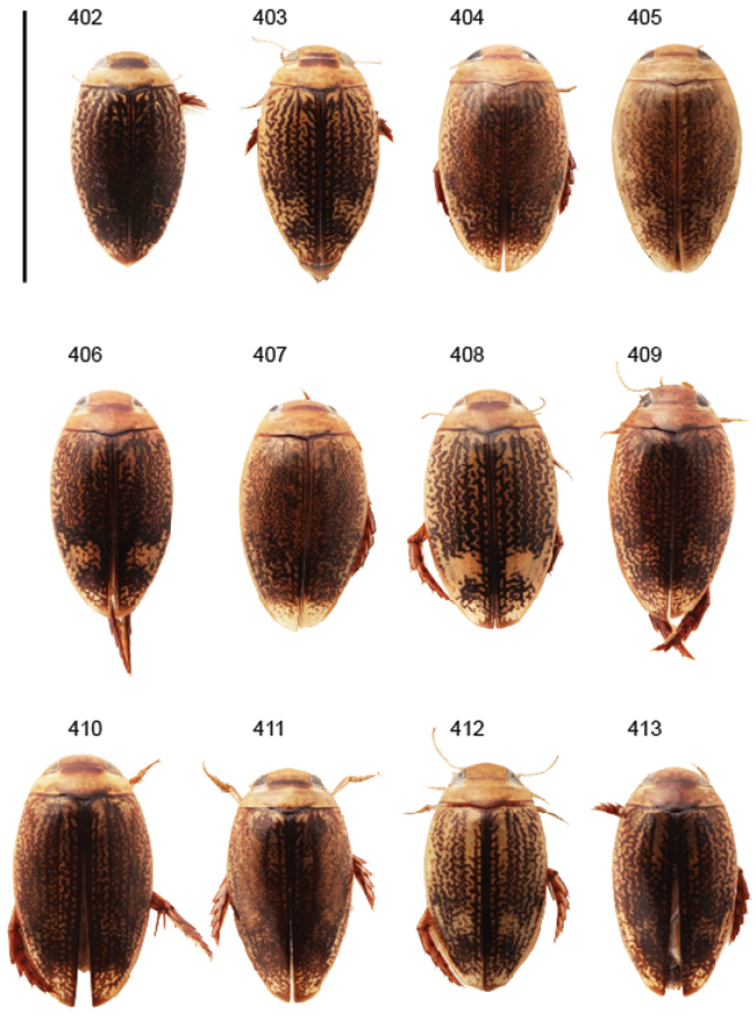
Dorsal habitus **402** *Laccophilus posticus* **403** *Laccophilus posticus* **404** *Laccophilus posticus* **405** *Laccophilus posticus* **406** *Laccophilus inobservatus* **407** *Laccophilus inobservatus* **408** *Laccophilus inobservatus* **409** *Laccophilus simplicistriatus* **410** *Laccophilus taeniolatus* **411** *Laccophilus taeniolatus* **412** *Laccophilus taeniolatus* **413** *Laccophilus propinquus*. Scale bar 5.0 mm.

**Figures 414–425.**
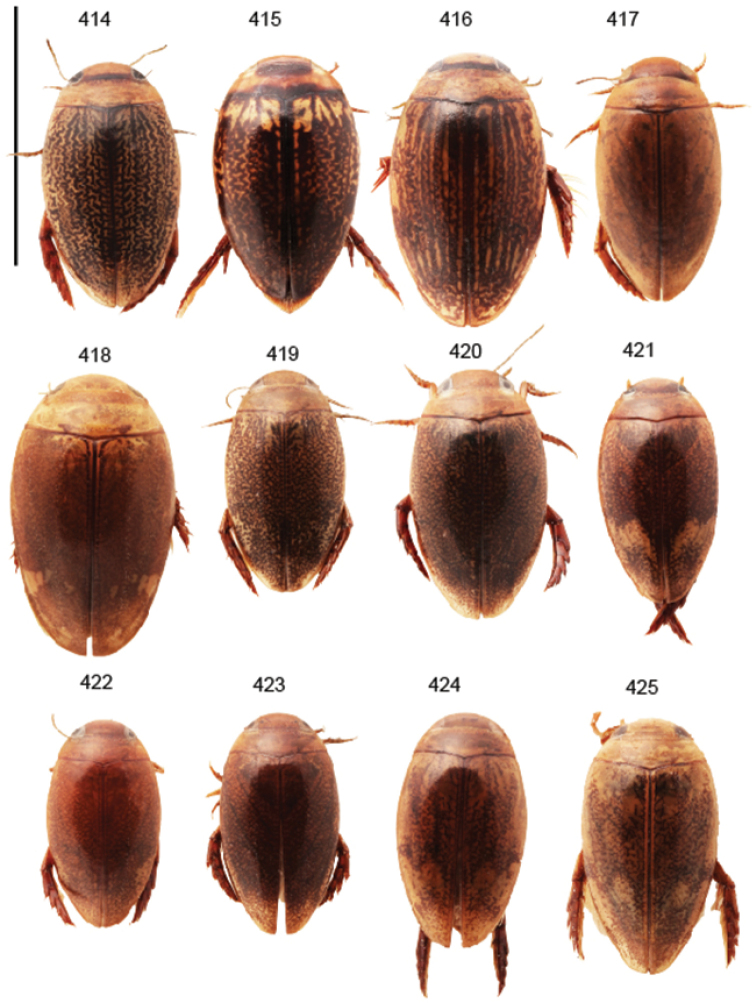
Dorsal habitus **414** *Laccophilus complicatus* **415** *Laccophilus irroratus* **416** *Laccophilus rivulosus* **417** *Laccophilus immundus* **418** *Laccophilus pellucidus* **419** *Laccophilus adspersus* **420** *Laccophilus olsoufieffi* **421** *Laccophilus olsoufieffi* **422** *Laccophilus modestus* **423** *Laccophilus cryptos* **424** *Laccophilus nodieri* **425** *Laccophilus flaveolus*. Scale bar 5.0 mm.

**Figures 426–437.**
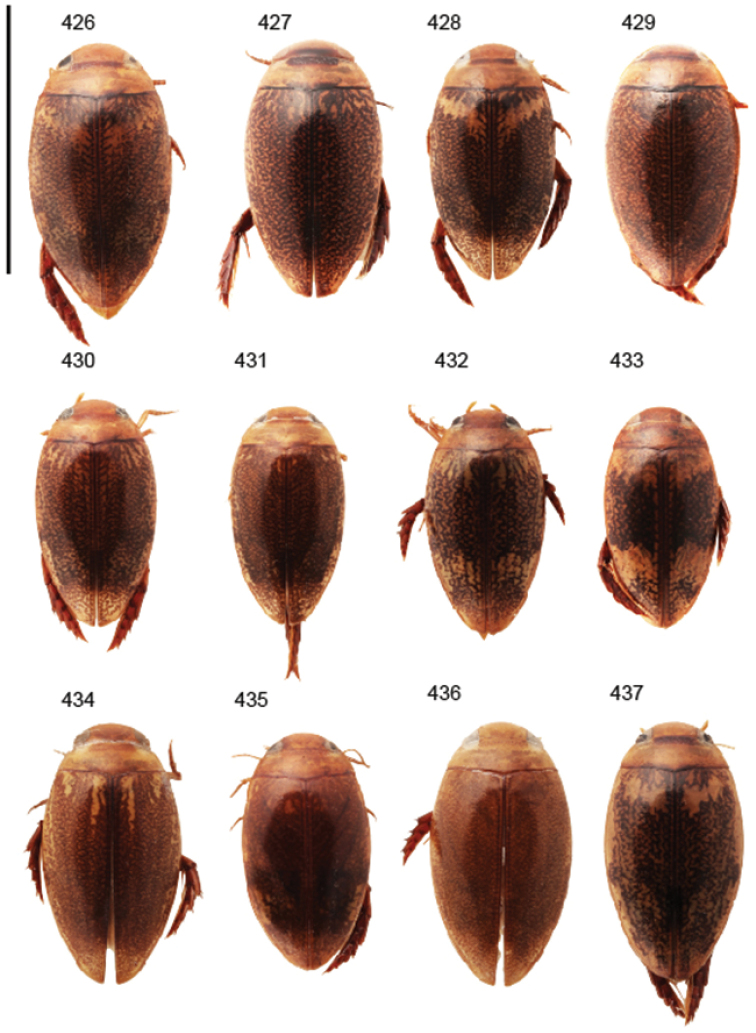
Dorsal habitus **426** *Laccophilus remex* **427** *Laccophilus remex* **428** *Laccophilus remex* **429** *Laccophilus remex* **430** *Laccophilus turbatus* **431** *Laccophilus turbatus* **432** *Laccophilus pallescens* **433** *Laccophilus pallescens* **434** *Laccophilus trilineola* **435** *Laccophilus mediocris* **436** *Laccophilus epinephes* **437** *Laccophilus saegeri*. Scale bar 5.0 mm.

**Figures 438–450.**
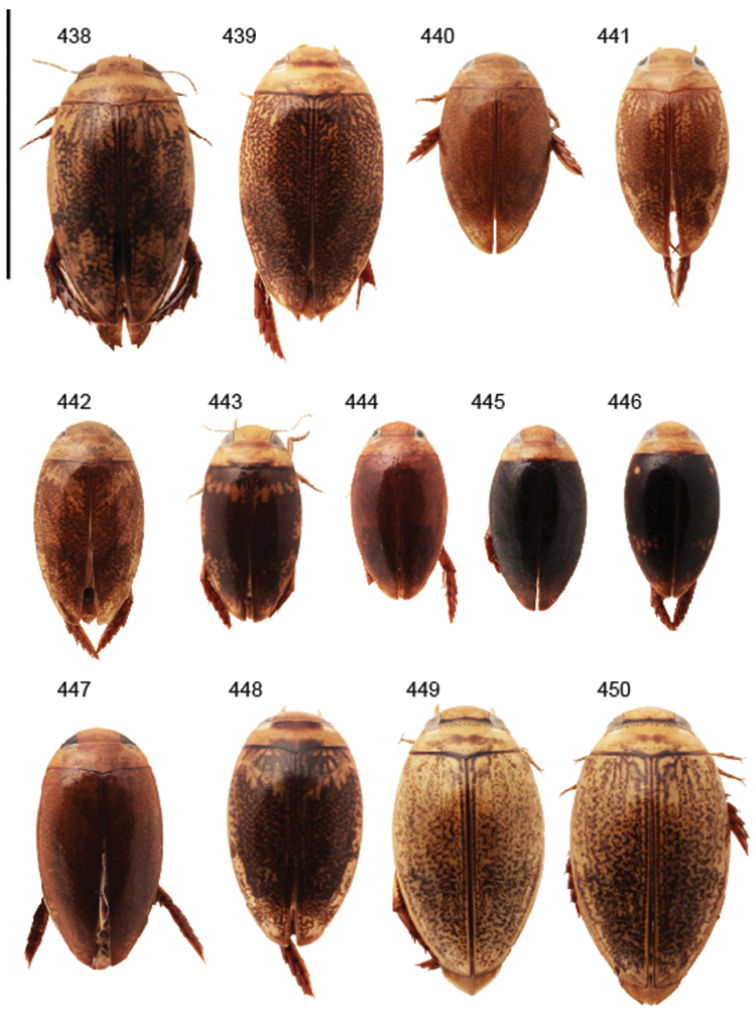
Dorsal habitus **438** *Laccophilus saegeri* **439** *Laccophilus enigmaticus* **440** *Laccophilus restrictus* **441** *Laccophilus restrictus* **442** *Laccophilus amicus* **443** *Laccophilus bellus* **444** *Laccophilus septicola* **445** *Laccophilus pullatus* **446** *Laccophilus luteosignatus* **447** *Laccophilus benoiti* **448** *Laccophilus addendus* **449** *Laccophilus vermiculosus* **450** *Laccophilus vermiculosus*. Scale bar 5.0 mm.

**Figures 451–462.**
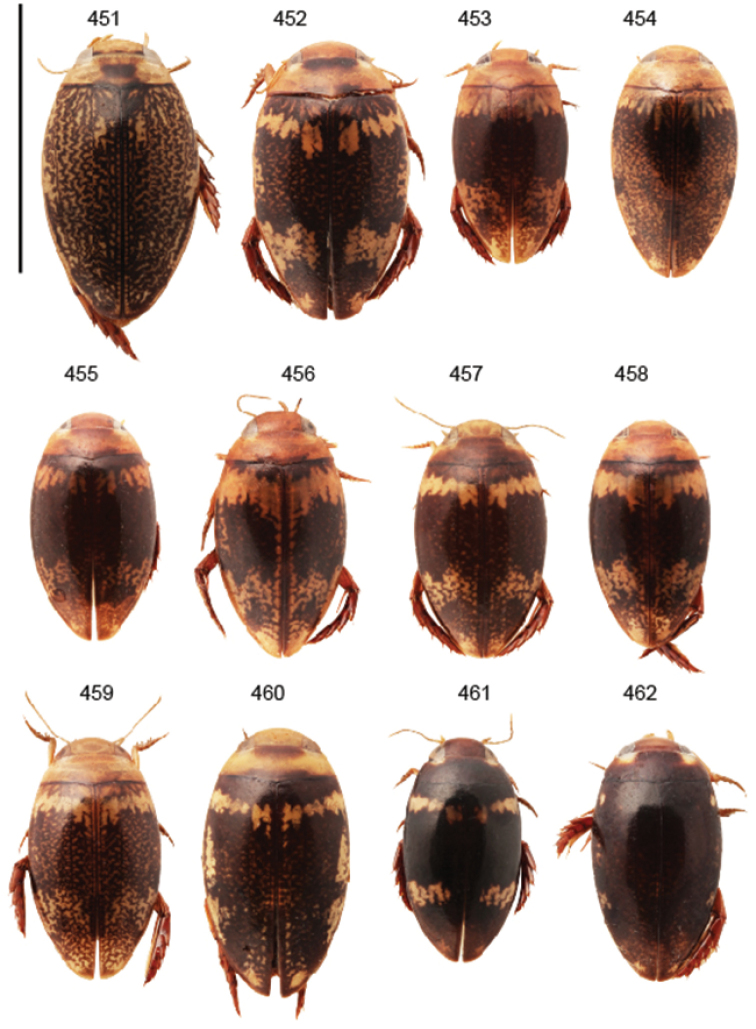
Dorsal habitus **451** *Laccophilus vermiculosus* **452** *Laccophilus guignoti* **453** *Laccophilus guentheri* **454** *Laccophilus guineensis* **455** *Laccophilus bizonatus* **456** *Laccophilus bizonatus* **457** *Laccophilus pulcher* **458** *Laccophilus concettae* **459** *Laccophilus biai* **460** *Laccophilus deceptor* **461** *Laccophilus bilardoi* **462** *Laccophilus decorosus*. Scale bar 5.0 mm.

**Figures 463–474.**
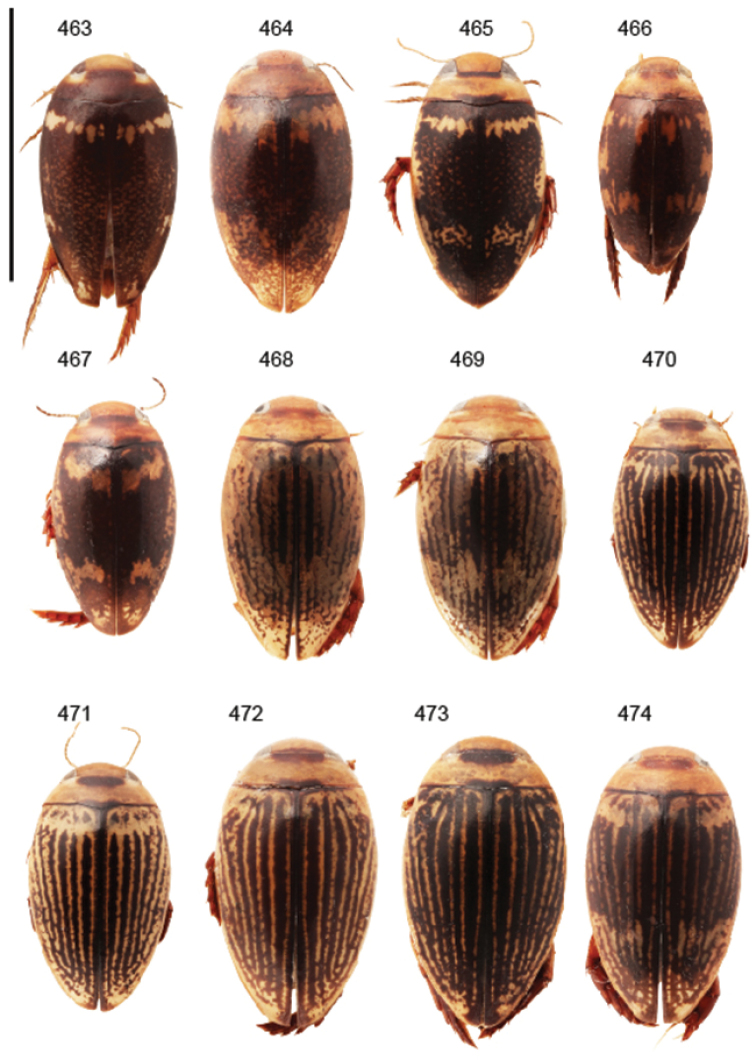
Dorsal habitus **463** *Laccophilus decorosus* **464** *Laccophilus tschoffeni* **465** *Laccophilus persimilis* **466** *Laccophilus caiaricus* **467** *Laccophilus poecilus* **468** *Laccophilus mutatus* **469** *Laccophilus mutatus* **470** *Laccophilus quindecimvittatus* **471** *Laccophilus quindecimvittatus* **472** *Laccophilus incrassatus* **473** *Laccophilus incrassatus* **474** *Laccophilus empheres*. Scale bar 5.0 mm.

**Figures 475–486.**
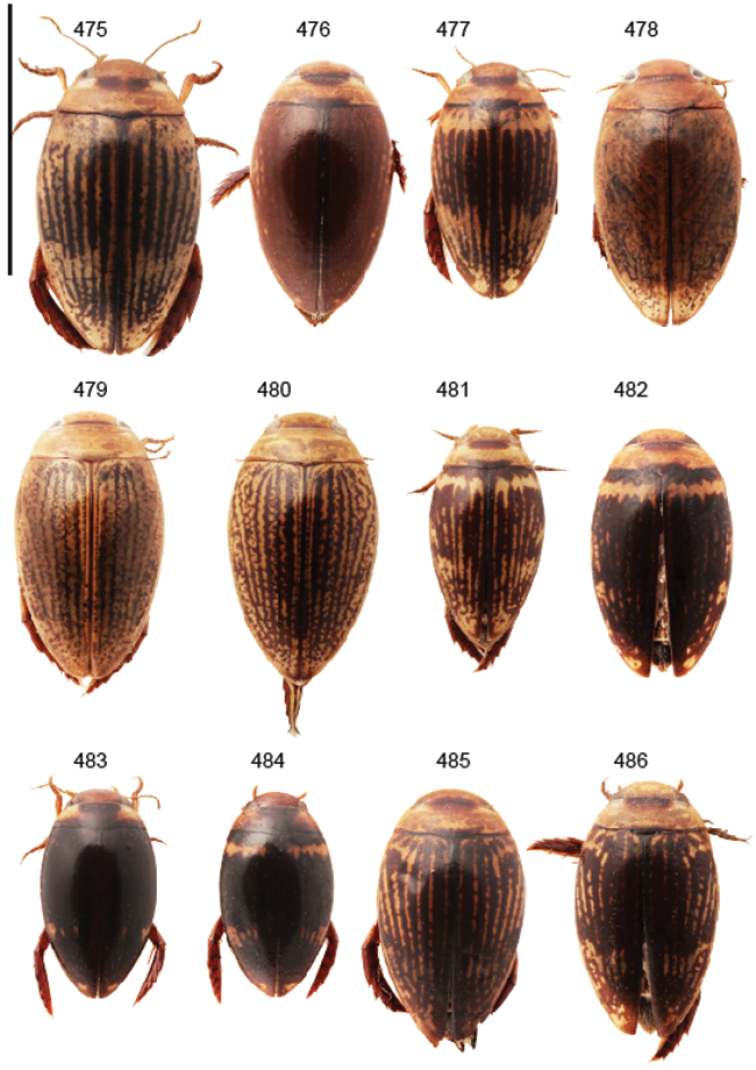
Dorsal habitus **475** *Laccophilus empheres* **476** *Laccophilus lateralis* **477** *Laccophilus lateralis* **478** *Laccophilus cyclopis* **479** *Laccophilus cyclopis* **480** *Laccophilus cyclopis* **481** *Laccophilus adjutor* **482** *Laccophilus necopinus* **483** *Laccophilus conjunctus* **484** *Laccophilus conjunctus* **485** *Laccophilus brownei* **486** *Laccophilus brownei*. Scale bar 5.0 mm.

**Figures 487–498.**
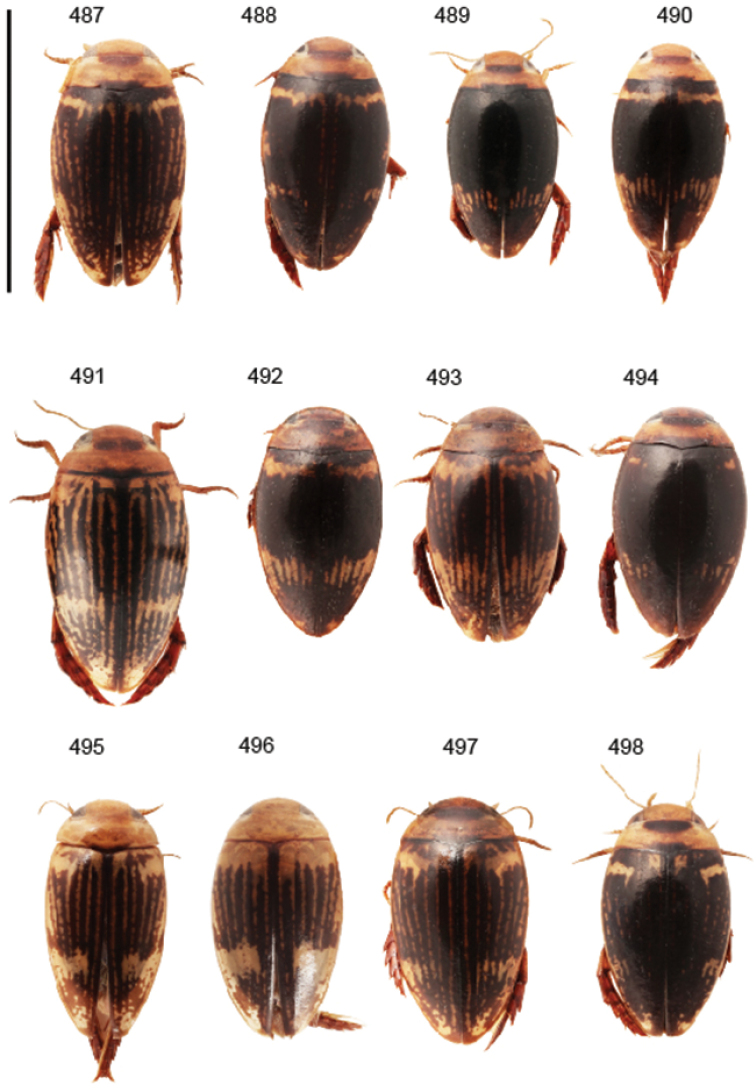
Dorsal habitus **487** *Laccophilus contiro* **488** *Laccophilus contiro* **489** *Laccophilus inconstans* **490** *Laccophilus inconstans* **491** *Laccophilus grammicus* **492** *Laccophilus flavoscriptus* **493** *Laccophilus flavoscriptus* **494** *Laccophilus flavoscriptus* **495** *Laccophilus burgeoni* **496** *Laccophilus burgeoni* **497** *Laccophilus lineatus* **498** *Laccophilus lineatus*. Scale bar 5.0 mm.

**Figures 499–512.**
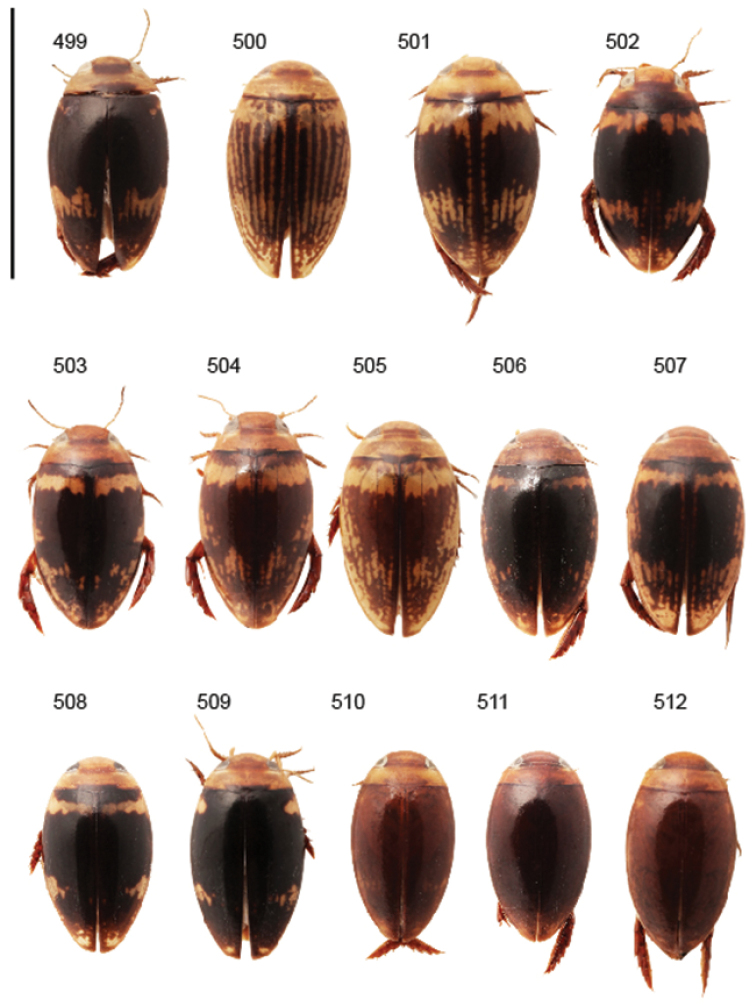
Dorsal habitus **499** *Laccophilus brancuccii* **500** *Laccophilus incomptus* **501** *Laccophilus desintegratus* **502** *Laccophilus desintegratus* **503** *Laccophilus luctuosus* **504** *Laccophilus luctuosus* **505** *Laccophilus luctuosus* **506** *Laccophilus australis* **507** *Laccophilus australis* **508** *Laccophilus secundus* **509** *Laccophilus secundus* **510** *Laccophilus inornatus* **511** *Laccophilus inornatus* **512** *Laccophilus canthydroides*. Scale bar 5.0 mm.

**Figures 513–526.**
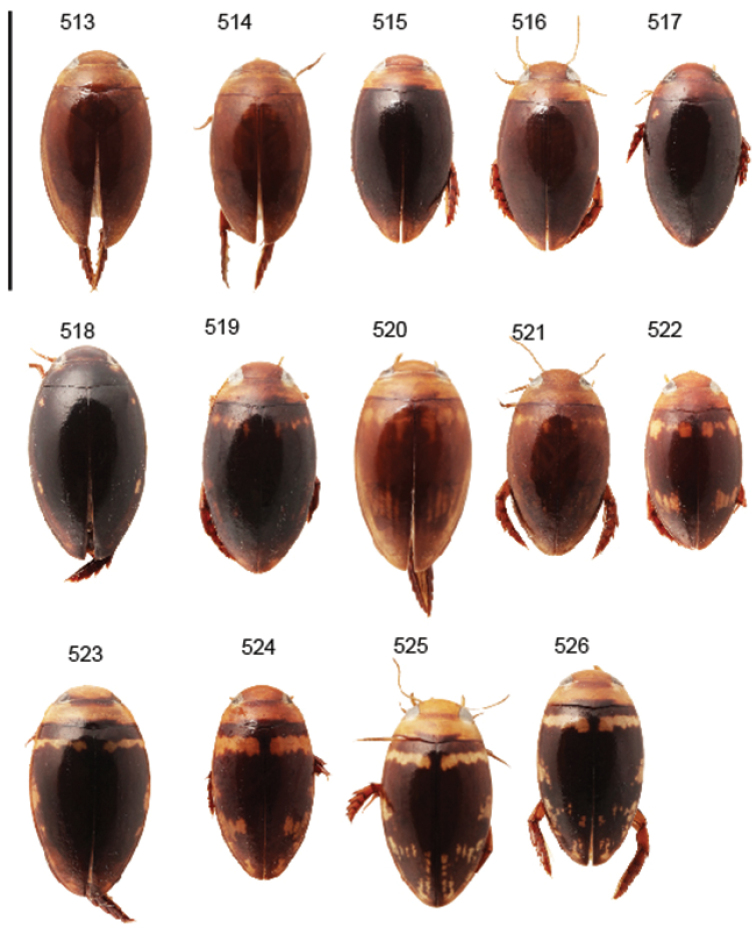
Dorsal habitus **513** *Laccophilus canthydroides* **514** *Laccophilus minimus* **515** *Laccophilus eboris* **516** *Laccophilus leonensis* **517** *Laccophilus villiersi* **518** *Laccophilus melas* **519** *Laccophilus livingstoni* **520** *Laccophilus insularum* **521** *Laccophilus insularum* **522** *Laccophilus garambanus* **523** *Laccophilus flavopictus* **524** *Laccophilus laeticulus* **525** *Laccophilus occidentalis* **526** *Laccophilus transversovittatus*. Scale bar 5.0 mm.

Figs 204 , 373–374 , 524 , 579

Laccophilus laeticulus Régimbart 1895: 145 (original description, faunistics); Zimmermann 1920a:19 (catalogue faunistics); Guignot 1943: 98 (faunistics); Guignot 1955b: 1096 (faunistics); Omer-Cooper 1958b: 37, 38, 39, 41 (description, faunistics, biology); Guignot 1959a: 533, 538 (description, faunistics); Medler 1980: 155 (faunistics, catalogue); Franciscolo and Sanfilippo 1990: 145, 146 (description, faunistics); Nilsson and Persson 1993: 79, 94 (faunistics, biology); Nilsson et al. 1995: 505 (faunistics); Rocchi 2000: 23 (faunistics); Nilsson 2001: 245 (catalogue, faunistics); Reintjes 2004: 67 (faunistics); van Vondel 2005: 131 (faunistics, biology); Nilsson 2015: 213 (catalogue, faunistics).

##### Notes on taxonomy.

Records, published later than Régimbart (1895) and Zimmermann (1920a) are considered uncertain because they may refer also to other species than *Laccophilus laeticulus*.

##### Type locality.

Guinea.

##### Type material studied

(1 ex.): Holotype: male: “Guinée / Museum Paris coll. Maurice Régimbart 1908 / *laeticulus* Rég.” (MNHN; habitus in Fig. 524).

##### Additional material studied

(1 ex.). **Sierra Leone**: “Sierra Leone Freetown A. Mocquerys 1889 / Museum Paris coll. Maurice Régimbart 1908” (1 ex. MNHN).

##### Diagnosis.

*Laccophilus laeticulus* is especially characterized by distinct elytral colour pattern and peculiar, narrow apex of penis. It is closely related to *Laccophilus occidentalis* sp. n., also occurring on mainland of the African continent. Male genitalia must be examined for correct determination. Apex of penis narrows in *Laccophilus laeticulus* while clearly broader in *Laccophilus occidentalis*. Subbasal pale marking on elytra in *Laccophilus laeticulus* is generally broader than corresponding marking in *Laccophilus occidentalis*. See also diagnosis of *Laccophilus transversovittatus* occurring in Madagascar (p. 260).

##### Description.

Body length 3.1–3.2 mm, width 1.7 mm. Dorsal, colour pattern uniform (Fig. 524).

Head: Pale ferrugineous. Submat, finely and somewhat indistinctly microsculptured. Reticulation double. Difference between size-categories of meshes minor and separation accordingly difficult. Large meshes, when discernible, contain about 3–5 small meshes. Head impunctate, except at eyes; with fine, scattered punctures. A few separate punctures may be discerned in centre of head between eyes.

Pronotum: Pale ferrugineous, frontally and at base in middle with a somewhat vague dark ferrugineous to ferrugineous area. Submat, finely and somewhat indistinctly microsculptured. Reticulation double. Difference between size-categories of meshes of microsculpture minor and separation accordingly difficult. Large meshes, when discernible, contain about 3-5 small meshes. Frontally and laterally with very fine, rather sparse punctures, which in part may be difficult to discern.

Elytra: Dark ferrugineous, with distinct, pale ferrugineous markings (Fig. 524). Submat, finely microsculptured. Reticulation double. Difference between size-categories of meshes minor and separation accordingly difficult. Large meshes, when discernible, contain about 3-5 small meshes. Laterally and posteriorly double reticulation becomes indistinct and finally disappears. Almost impunctate. In posterior half lateral punctures form a narrow and shallow, pre-apical furrow.

Ventral aspect: Pale ferrugineous to ferrugineous, posteriorly gradually darker but exhibits no distinct colour pattern. Slightly mat, finely microsculptured. In part reticulation reduced and obliterated. Almost impunctate. Basal ventrites with fine, curved striae. Prosternal process rather slender, apex extended and pointed. Metacoxal plates with some indistinct, shallow furrows. Fine stridulatory apparatus file consists of broad, fine ridges. Apical ventrite asymmetric, with lateral, triangular knob (Fig. 204).

Legs: Pro- and mesotarsus slightly enlarged, extended. With distinct suckers.

Male genitalia: Penis curved; narrows distinctly towards apex, provided with a small and blunt hook (Figs 373–374).

Female: Unknown.

##### Distribution.

Guinea, Sierra Leone (Fig. 579).

##### Collecting circumstances.

Unknown.

#### Laccophilus occidentalis sp. n.

Taxon classificationAnimaliaColeopteraDytiscidae

http://zoobank.org/ED6393AD-9EC3-4B75-97A9-FFAB600A5417

Figs 205–206 , 375–376 , 525 , 579

##### Type locality.

Sierra Leone: Makeni.

##### Type material

(96 exs.). Holotype: male: “Sierra Leone: Makeni 12°03'W, 8°53'N, 27.XI. 1993 loc. 9 light trap 18-21 / Lund University Sierra Leone Expedition 1993 leg. L. Cederholm-R. Danielsson-R. Hall / *Laccophilus laeticulus* Régimbart Det. AN Nilsson -94” (MZLU). – Paratypes: Same data as holotype (7 exs. MZLU, 6 exs. MZH; habitus in Fig. 525). – Gambia: “Gambia Bathurst jan. 68. Palm / *Laccophilus laeticulus* Rég. det. Sven Persson” (1 ex. MZLU). – Senegal: “Niokolo Koba NP, 13°01.13'N, 13°18.48'W 15.7. 2004 leg. Marek Halada” (2 exs. NMPC). – Mali: “Soudan francais Bamako” (1 ex. MNHN). – Guinea: “République de Guinea PNHN8 10°28'40"N, 10°26'42"W, Faranah F. Niger, Somorya17-21.1. 1996 leg. M. Mei / *Laccophilus laeticulus* Rég. det. S. Rocchi 96” (2 exs. CSR); “Seredou 4.4. 1975 lux, Zott” (5 exs. ZMHB, 1 ex. MZH); same data but “5.4. 1975” (1 ex. ZMHB); same data but “4.5. 1975” (4 exs. ZMHB, 1 ex. MZH); same data but “7-8.4. 1975” (9 exs. ZMHB, 3 exs. MZH); “Kindia Villiers 1954” (1 ex. MNHN). – Ivory Coast: “Cote d’Ivoire Comoé NP, N8,5° W3,5° leg. N. Reintjes, det. F. Pederzani / 11.1. 1999 KB8A Kongo River / KR in Koleopt. Rdsch. 74: 45-74 / *Laccophilus laeticulus* Régimbart, 1895” (2 exs. NMW). – Ghana: “Ghana: Northern Reg. Damongo Game res. 9.04N-1.48W / 11.11. 1970: no. 440 leg. Endrödy-Younga” (5 exs. TMSA, 2 exs. MZH); “Ghana Kumasi 12.6. 67 S. Endrödy-Younga” (2 exs. MHNG, 1 ex. MZH); “Volta Region, R. Volta at Kpong 28.11. 1993 Andersen, light trap” (1 ex. MZH). – Benin: “Benin, Pénésseoulou, pond: forest area Oct. 2003 Leg. G. Georgen / 37 / *Laccophilus laeticulus* Rég. det. Wewalka 2005” (1 ex. NMW); “Parakou at light 29.9.1988 leg. C. v. Houdt / *Laccophilus laeticulus* Rég. det. S. Rocchi 2000” (1 ex. CSR). – Nigeria: “Nigeria NW St. Badeggi rice fields 8-9.8. 1973 R. Linnavuori leg.” (2 exs. MZH); “Ibadan, Nigeria ca, Jan.-Juni 1954 H. Stenholt-Clausen / *Laccophilus laeticulus* Rég. J. Balfour-Browne det. VII. 1961” (2 exs. ZMUC, 1 ex. MZH); “Ibadan at light 27.XI. 1955” (2 exs. BMNH, 1 ex. MZH); “Coll. Mus. Tervuren Nigeria Zaria, à la lumière 1969 Dr. H. Roberts” (7 exs. MRAC, 2 exs. MZH); “Stream, Enugo-Makurdi rd. 26.4. 1963 J.O-C.” (1 ex. AMGS); “R. Kaduna 4.5 mi from Jos 13.4. 1963 JOC” (16 exs. AMGS). – Central African Republic: “Bozo 7. 1981 Degallier” (1 ex. NHMB); same data but “21.5. 1981”(1 ex. NHMB). – Zaire: “Congo Belge PNG Miss. H. De Saeger/II/fd/12, 10.3. 1952, 3180” (1 ex. MNHN).

##### Diagnosis.

Very close to *Laccophilus laeticulus* and *Laccophilus transversovittatus*. Correct determination requires study of male genitalia. Diagnostic features for separation of the three species is given under diagnosis of *Laccophilus laeticulus* (p. 258) and *Laccophilus transversovittatus* (p. 260).

##### Description.

Body length 2.8–3.3 mm, width 1.6–1.9 mm. Dorsal, colour pattern distinct, quite uniform (Fig. 525).

Head and pronotum: As in *Laccophilus laeticulus*.

Elytra: As in *Laccophilus laeticulus* but pale basal marking generally somewhat narrower in *Laccophilus occidentalis*. Pale markings posterior to middle often reduced to small, separate spots (Fig. 525).

Ventral aspect: Apical ventrite (Fig. 205)

Legs: As in *Laccophilus laeticulus*.

Male genitalia: Penis in apical half broad, narrows only moderately towards apex (Figs 375–376).

Female: Pro- and mesotarsus slender. Apical ventrite lacks sharp knob (Fig. 206). Female has similar appearance of stridulatory apparatus as in male.

##### Etymology.

The name *occidentalis* is a Latin adjective meaning “western”. It here refers to the wide range of the new species in western part of Africa, south of Sahara.

##### Distribution.

Gambia, Senegal, Mali, Guinea, Sierra Leone, Ivory Coast, Ghana, Benin, Nigeria, Central African Republic, Zaire (Fig. 579).

##### Collecting circumstances.

Almost unknown. Sometimes collected at light.

#### Laccophilus transversovittatus sp. n.

Taxon classificationAnimaliaColeopteraDytiscidae

http://zoobank.org/A4BEEBD3-45DD-420C-A536-B4595E4D5D95

Figs 207–208 , 377 , 526 , 580

##### Type locality.

Madagascar: Isalo, Menamaty River.

##### Type material

(32 EXS.). Holotype: male: “MAD: FIAN: Isalo Menamaty R, degraded river with lots of vegetation, used by women to wash clothes in P41AM01, 11.5. 2006 N-22°33.001 E45°24.074, 757 m leg. Bergsten et al / BMNH(E) 741824” (NHRS). – Paratypes: Same as holotype (5 exs. NHRS, 2 exs. NMW, 2 exs. MZH); “MAD, FIAN. Isalo, Running water, P41B, N -22.546, E 45.397, 773 m, 11.5. 2006 Bergsten / BMNH(E) DNA voucher” (6 exs. NHRS); “MAD, FIAN. Isalo, Menamaty R, degraded river, N -22.55, E -45,401: 757 m, 11.5. 2006 Bergsten et al. leg / BMNH(E) voucher” (5 exs. NHRS); “Madagascar: Mahajunga Melaky btw. Morafenobe-Ambohijanahary S18.19091, E045.19986, 290 m.a.o. 19.12. 2009 water net, field# MAD09-74 leg. J. Bergsten, N. Jönsson, T. Ranarilalatiana, H.J. Randriamihaja” (5 exs. NHRS; habitus in Fig. 526); same data, add “NHRS-JLKB 0000000720” (1 ex. NHRS); “Madagascar: Mahajunga Melaky: Tsingy de Bemaraha NP. S19.13260, E044.80891, 62 m.a.o., 14.12. 2009, water net, field# MAD09-54, leg. J. Bergsten, N. Jönsson, T. Ranarilalatiana, H.J. Randriamihaja” (1 ex. NHRS); “Madagascar (102) 6.10. 2001 Ankaratra (Antananarivo) Reserve Manjakatompo / Helocrene at left border of affluent to Lac Froid, 1700 m asl. 19.5-25.0°C, 0.010-0.100 mS/cm / Gerecke et Goldschmidt collectors BMNH(E)2004-46” (1 ex. BMNH); “Sambirano, Ankaramy env., J. Moravec leg. 4.11. 2000” (1 ex. NMPC); “Antsabe Lat. -13.648 Lon 48.721, 21.11. 2004 Balke Lees & Monaghan / DNA voucher BMNH <672773>” (1 ex. NHRS); “MAD FIAN, Isalo Antsabe source of Piscine Naturelle, Waterhole N-22.553, E 45.368, 859 m, 12.5. 2006 Bergsten et al.” (1 ex. NHRS).

##### Diagnosis.

*Laccophilus transversovittatus* is closely related to *Laccophilus laeticulus* and *Laccophilus occidentalis*. Diagnostic characters for all three species are found in the shape of penis apex. Apex in *Laccophilus laeticulus* is distinctly more slender than in the two other species. Penis of *Laccophilus occidentalis* is straighter than in *Laccophilus transversovittatus* and apical dent is almost absent while distinct in *Laccophilus transversovittatus*. *Laccophilus transversovittatus* resembles externally also of *Laccophilus luctuosus*, which is also a species solely recorded thus far in Madagascar. The male genitalia of *Laccophilus luctuosus* is, however, clearly different in comparison with *Laccophilus transversovittatus*.

##### Description.

Body length 3.2–3.5 mm, width 1.7–1.9 mm. Body appears somewhat flattened. Colour pattern exhibits only minor variation; basal, pale, transverse marking on elytra moderately broad but always distinct and only broken narrowly at suture (Fig. 526).

Head: Pale ferrugineous; posteriorly head becomes slightly darker. Change of colour-intensity is vague. Slightly mat, finely to very finely microsculptured. Reticulation double; small meshes in part reduced and almost absent. Large meshes contain, when discernible, 3–5 small meshes. Almost impunctate. At eyes with some irregular punctures. Area of punctures continues towards middle of head as a sparse, irregular row of punctures.

Pronotum: Pale ferrugineous. Basally with a broad but quite narrow, distinct, dark ferrugineous marking. Anteriorly with a somewhat vague, dark ferrugineous to ferrugineous spot. Rather shiny, although finely to very finely microsculptured. Reticulation double. Small meshes in part reduced and almost absent. Large meshes contain, when discernible, 3–5 small meshes. Almost impunctate; laterally and anteriorly with fine but irregular punctures.

Elytra: Dark ferrugineous, with a distinct, subbasal, pale ferrugineous marking. In addition, laterally with a narrow pale area and posterior to middle and apically with some irregular, small pale spots (Fig. 526). Rather shiny, although finely microsculptured. Reticulation double. Anteriorly small meshes in part reduced and almost absent while large meshes clearly discernible. Posteriorly small meshes dominate and large meshes become rather indistinct. Almost impunctate. Fine, irregular, punctures form a discal row of punctures. Dorsolateral row of punctures simply indicated by a few, fine and irregular punctures. Lateral row consists of sparse punctures. Lateral, preapical row of punctures located in a shallow and pubescent furrow.

Ventral aspect: Pale ferrugineous to ferrugineous; abdomen becomes gradually darker towards apex; dark ferrugineous. Rather shiny, although finely microsculptured. Reticulation of abdomen strongly reduced and extensively absent. Abdomen with fine, curved striae. Almost impunctate. Apical ventrite provided with a sharp knob on one side (Fig. 207). Metacoxal plates with very fine, shallow furrows which in posterior half are reduced and largely lacking. Stridulatory apparatus somewhat vague; consists of a number of fine striae, arranged close to each other. Prosternal process slender, apex slightly extended and pointed.

Legs: Pro- and mesotarsus slightly enlarged; provided with fine suckers.

Male genitalia: Penis in lateral aspect quite broad, distinctly angled with a small but distinct apical dent (Fig. 377).

Female: Pro- and mesotarsus slender. Apical ventrite lacks knob (Fig. 208).

##### Etymology.

The name *transversovittatus* is a Latin adjective based on a verb meaning “provided with transverse stripes”. It here refers to the transversely located, subbasal, pale area on the elytra.

##### Distribution.

Madagascar (Fig. 575).

##### Collecting circumstances.

Collected in a degraded river with lots of vegetation, used by women to wash clothes in.

## Figures

**Figure 527.**
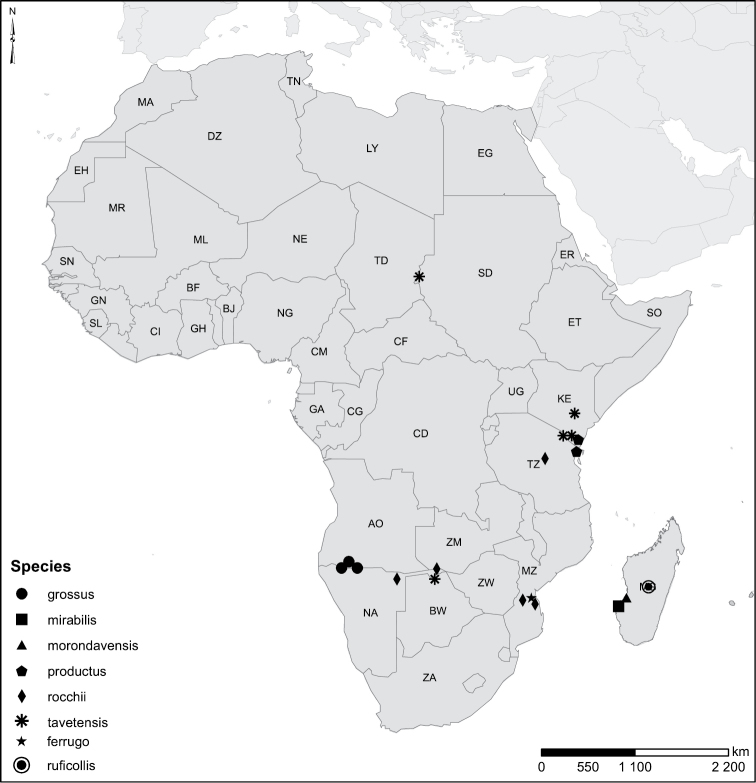
Known distribution based on examined specimens of *Laccophilus grossus*, *Laccophilus mirabilis*, *Laccophilus morondavensis*, *Laccophilus productus*, *Laccophilus rocchii*, *Laccophilus tavetensis*, *Laccophilus ferrugo* and *Laccophilus ruficollis*.

**Figure 528.**
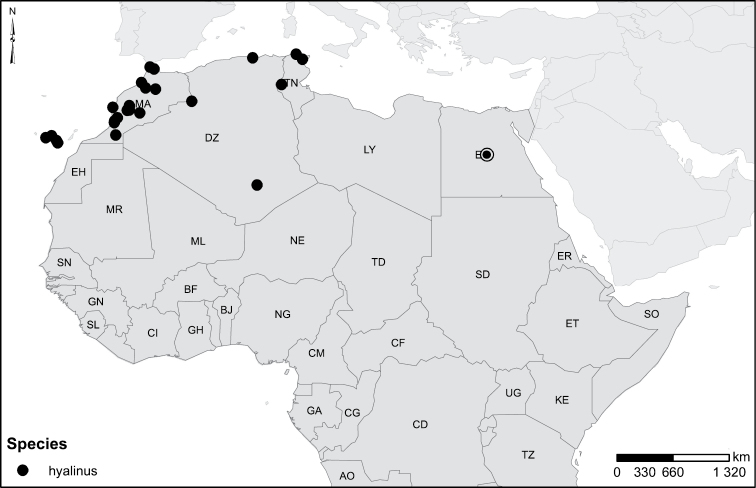
Known distribution based on examined specimens of *Laccophilus hyalinus*.

**Figure 529.**
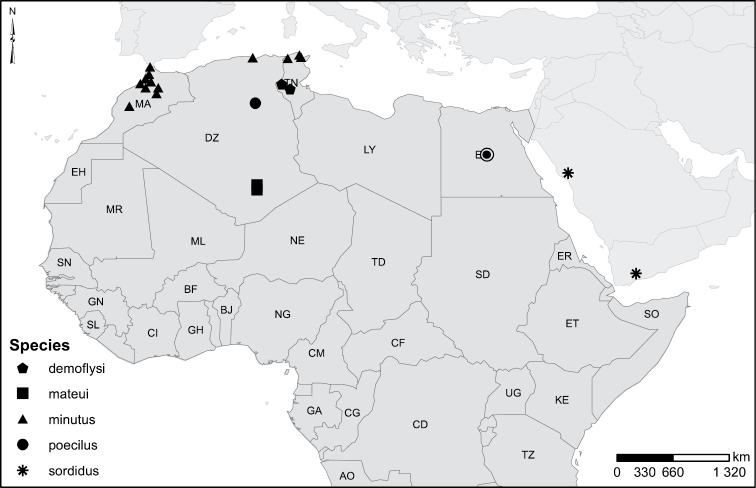
Known distribution based on examined specimens of *Laccophilus demoflysi*, *Laccophilus mateui*, *Laccophilus minutus*, *Laccophilus poecilus* and *Laccophilus sordidus*.

**Figure 530.**
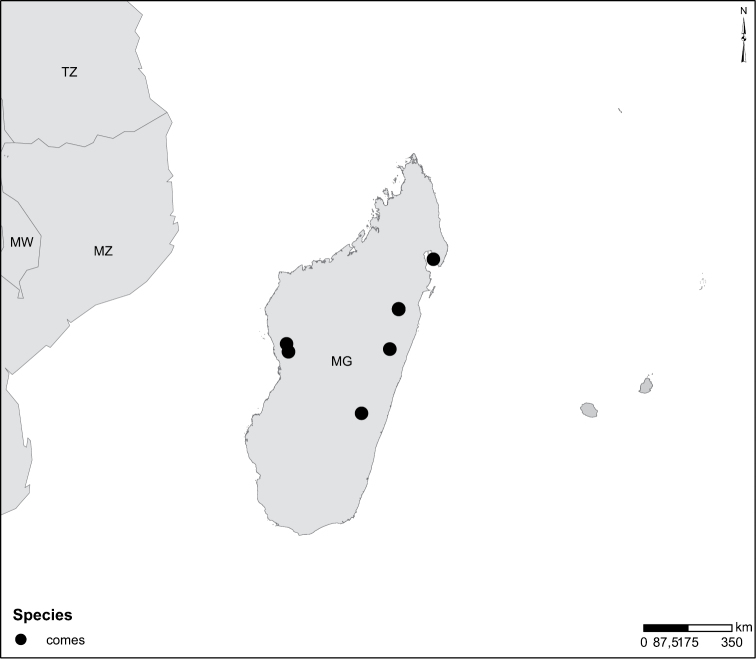
Known distribution based on examined specimens of *Laccophilus comes*.

**Figure 531.**
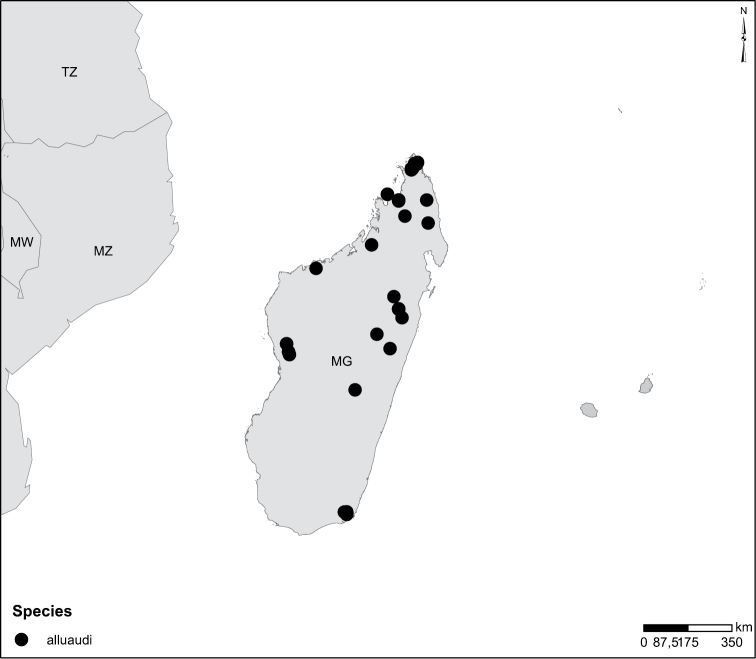
Known distribution based on examined specimens of *Laccophilus alluaudi*.

**Figure 532.**
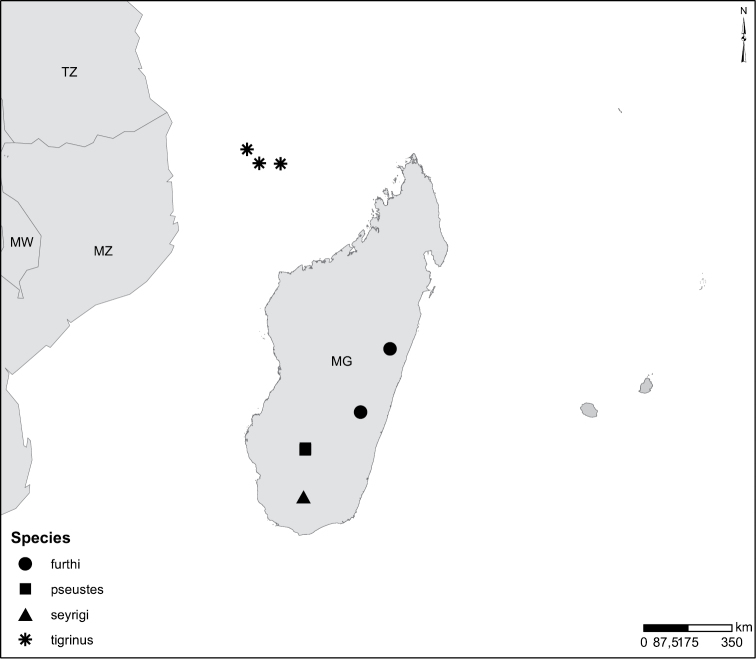
Known distribution based on examined specimens of *Laccophilus furthi*, *Laccophilus pseustes*, *Laccophilus seyrigi* and *Laccophilus tigrinus*.

**Figure 533.**
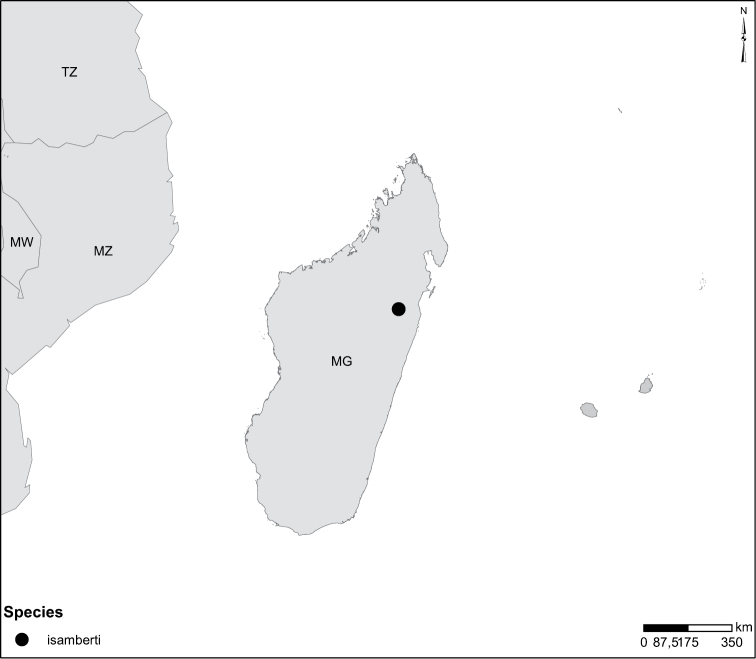
Known distribution based on examined specimens of *Laccophilus isamberti*.

**Figure 534.**
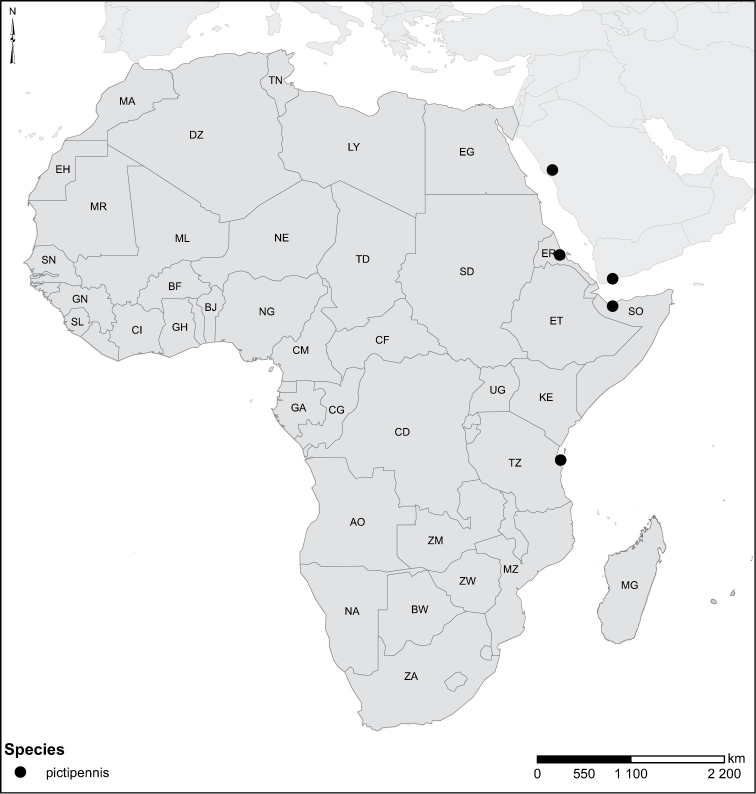
Known distribution based on examined specimens of *Laccophilus pictipennis*.

**Figure 535.**
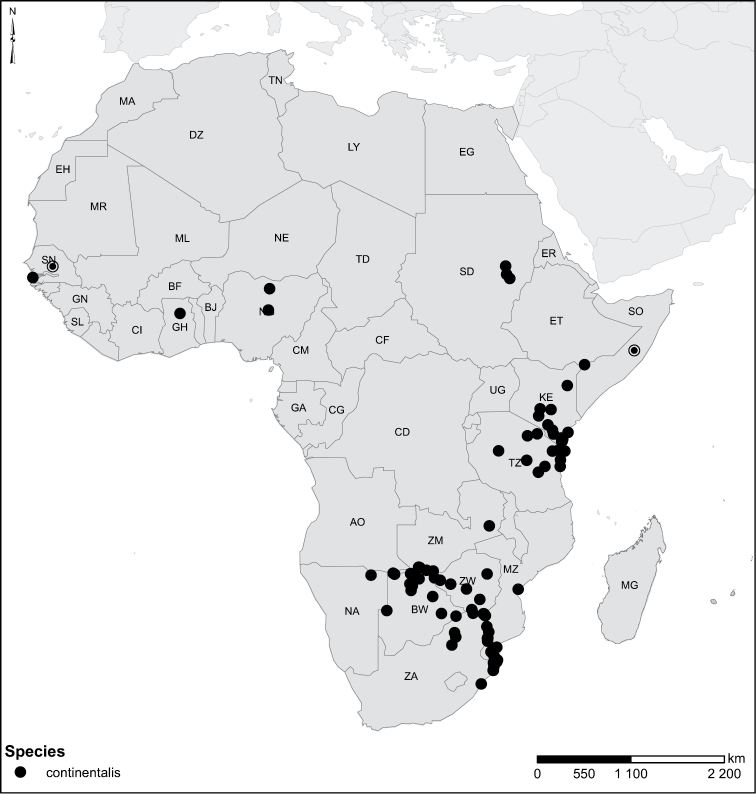
Known distribution based on examined specimens of *Laccophilus continentalis*.

**Figure 536.**
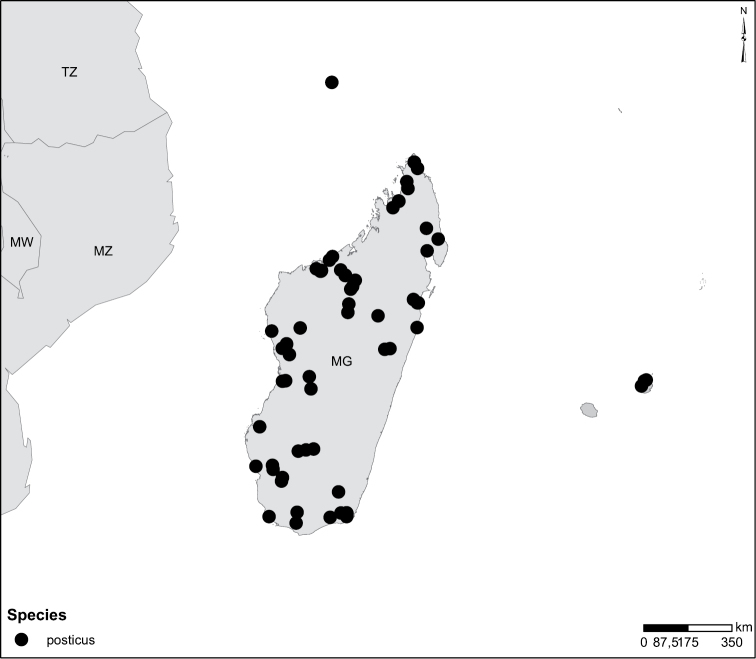
Known distribution based on examined specimens of *Laccophilus posticus*.

**Figure 537.**
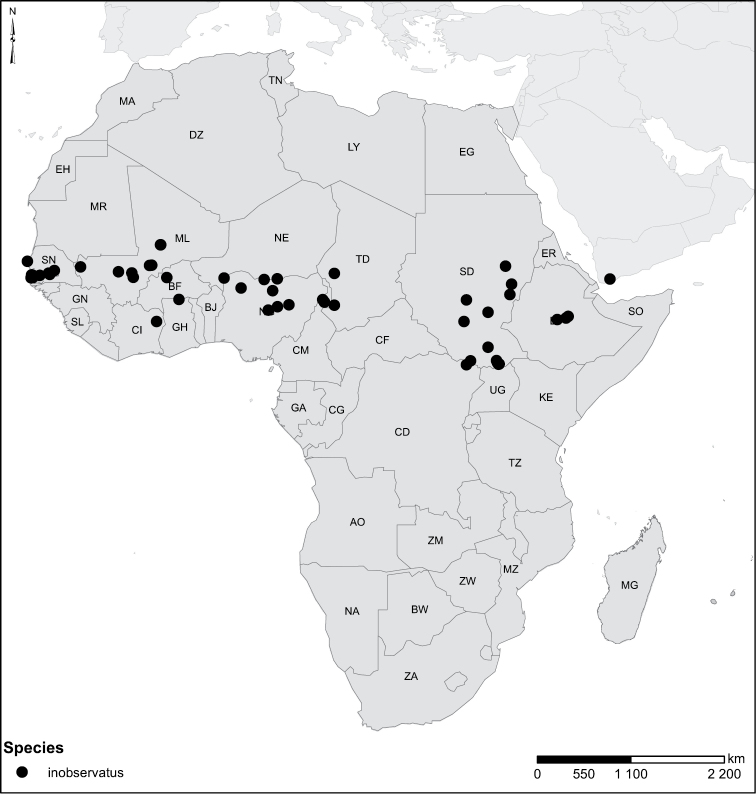
Known distribution based on examined specimens of *Laccophilus inobservatus*.

**Figure 538.**
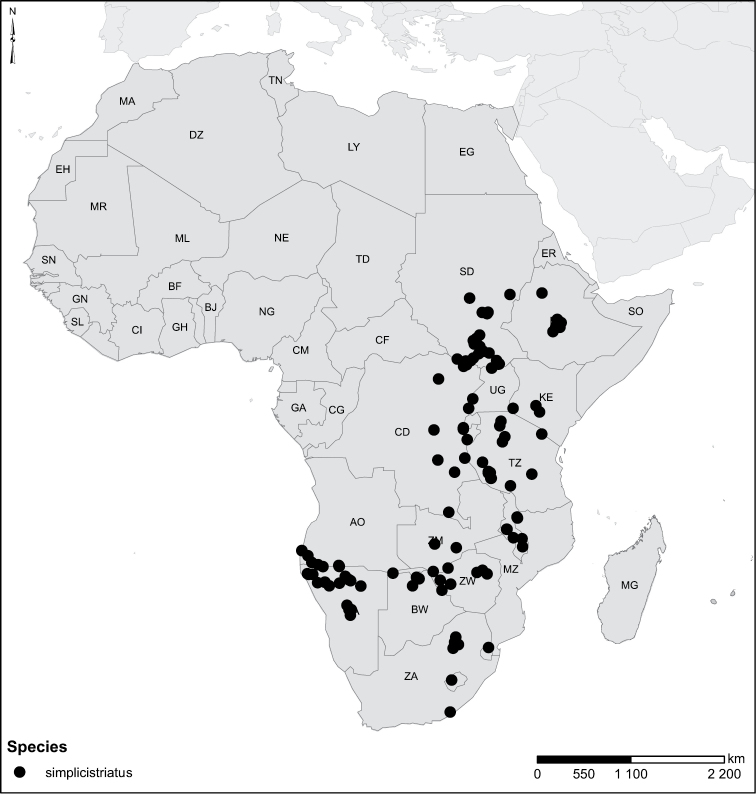
Known distribution based on examined specimens of *Laccophilus simplicistriatus*.

**Figure 539.**
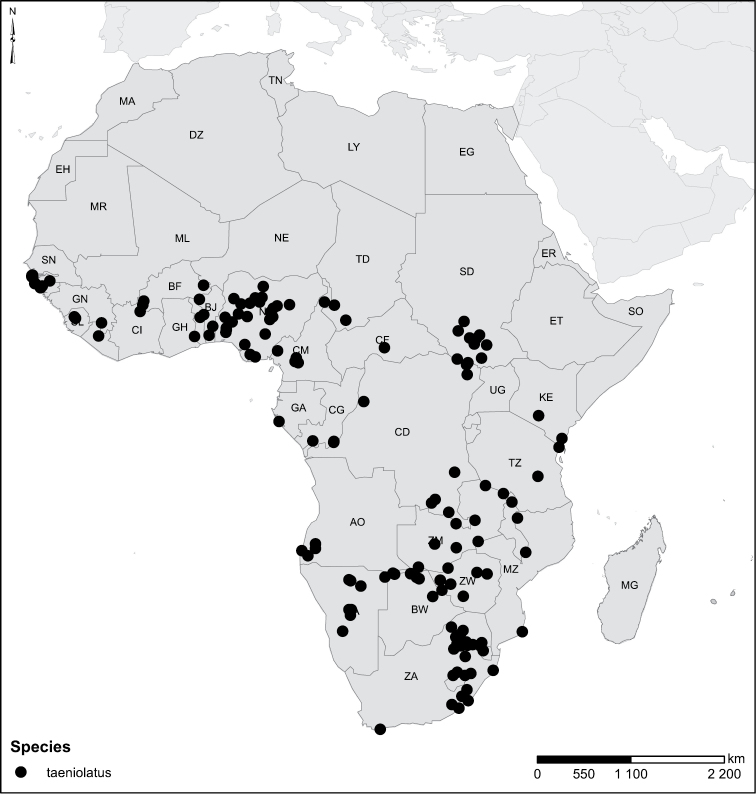
Known distribution based on examined specimens of *Laccophilus taeniolatus*.

**Figure 540.**
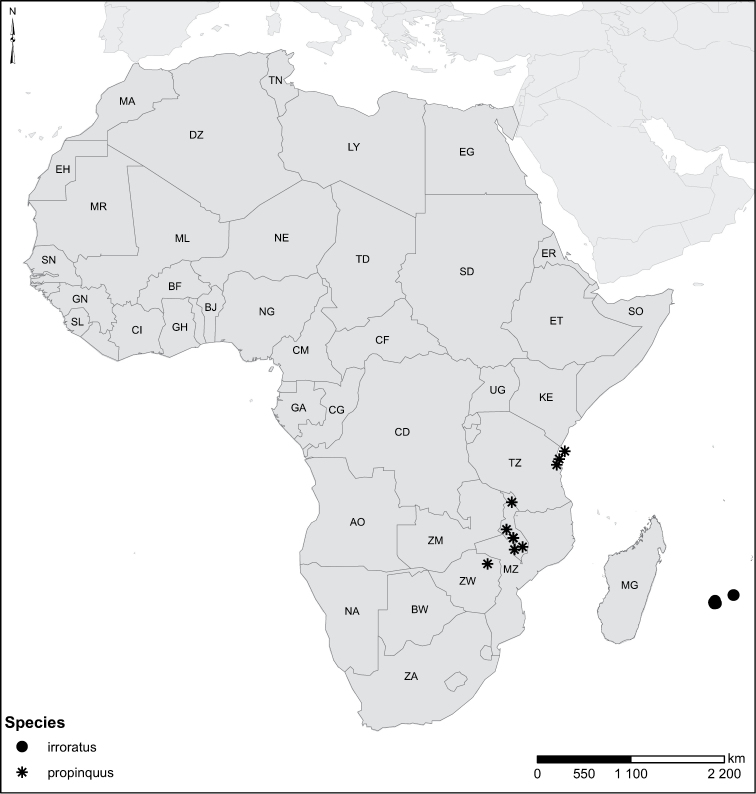
Known distribution based on examined specimens of *Laccophilus irroratus* and *Laccophilus propinquus*.

**Figure 541.**
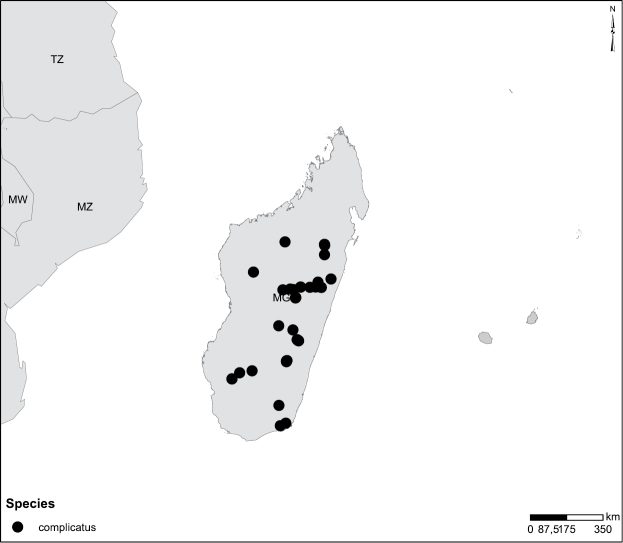
Known distribution based on examined specimens of *Laccophilus complicatus*.

**Figure 542.**
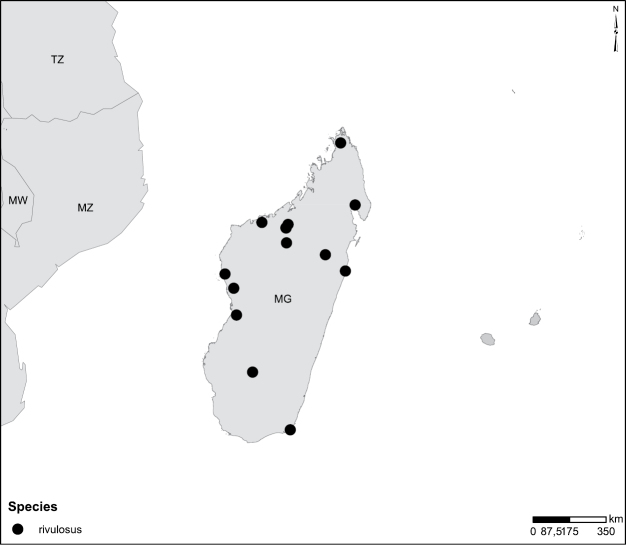
Known distribution based on examined specimens of *Laccophilus rivulosus*.

**Figure 543.**
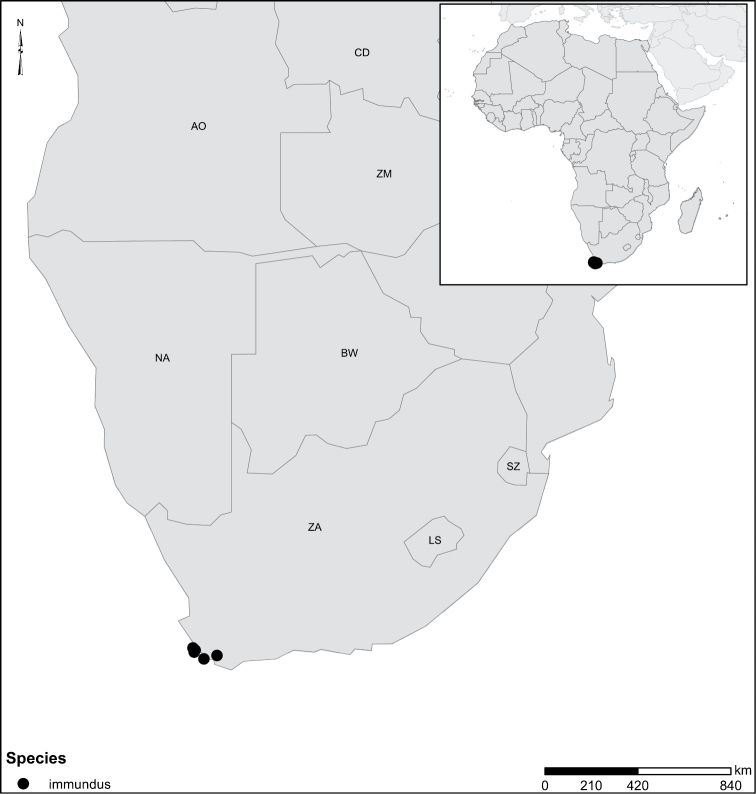
Known distribution based on examined specimens of *Laccophilus immundus*.

**Figure 544.**
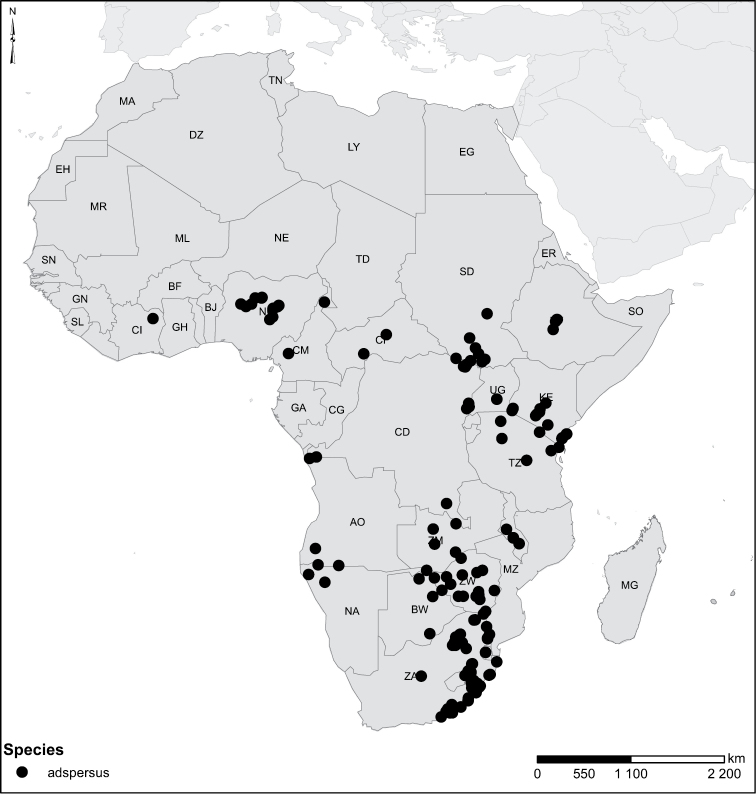
Known distribution based on examined specimens of *Laccophilus adspersus*.

**Figure 545.**
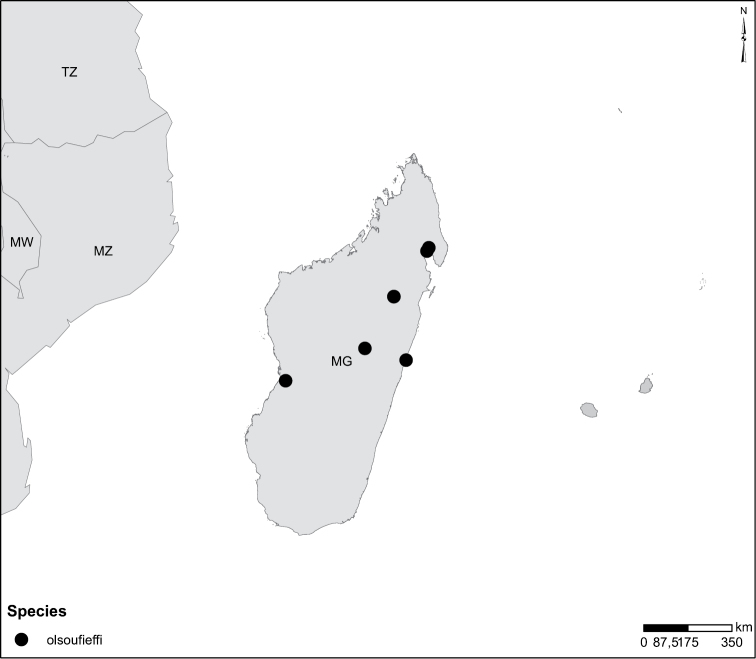
Known distribution based on examined specimens of *Laccophilus olsoufieffi*.

**Figure 546.**
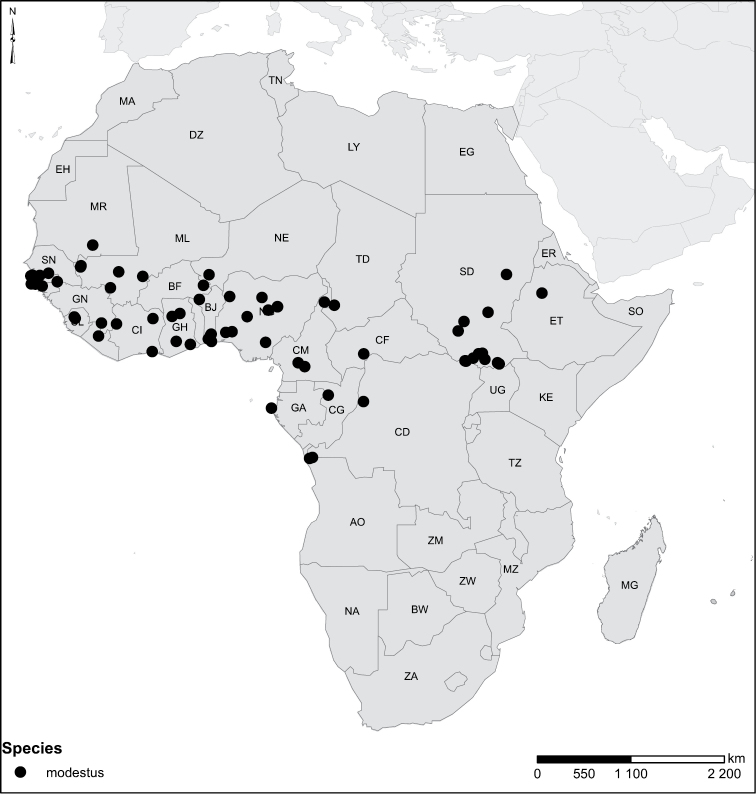
Known distribution based on examined specimens of *Laccophilus modestus*.

**Figure 547.**
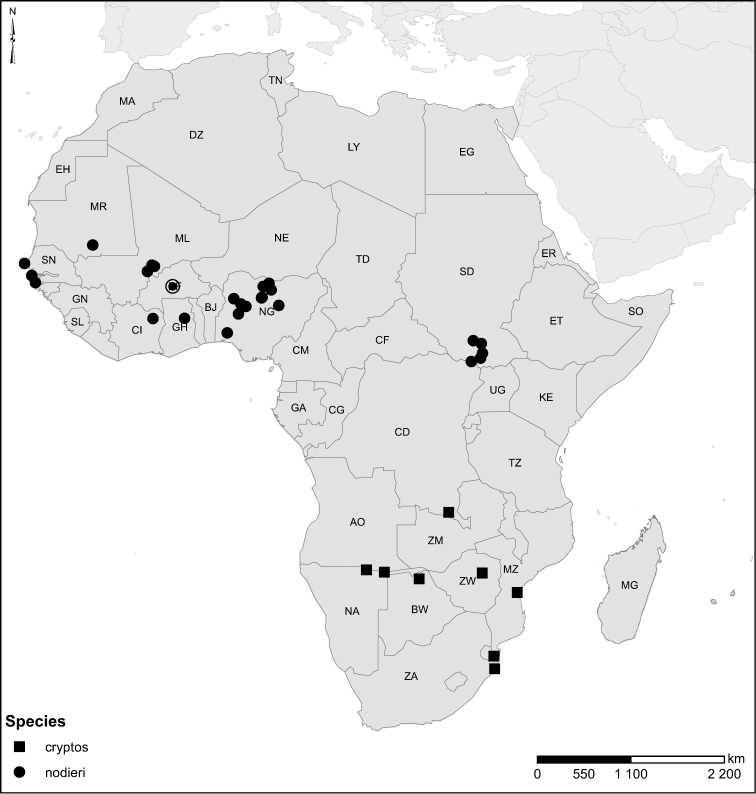
Known distribution based on examined specimens of *Laccophilus cryptos* and *Laccophilus nodieri*.

**Figure 548.**
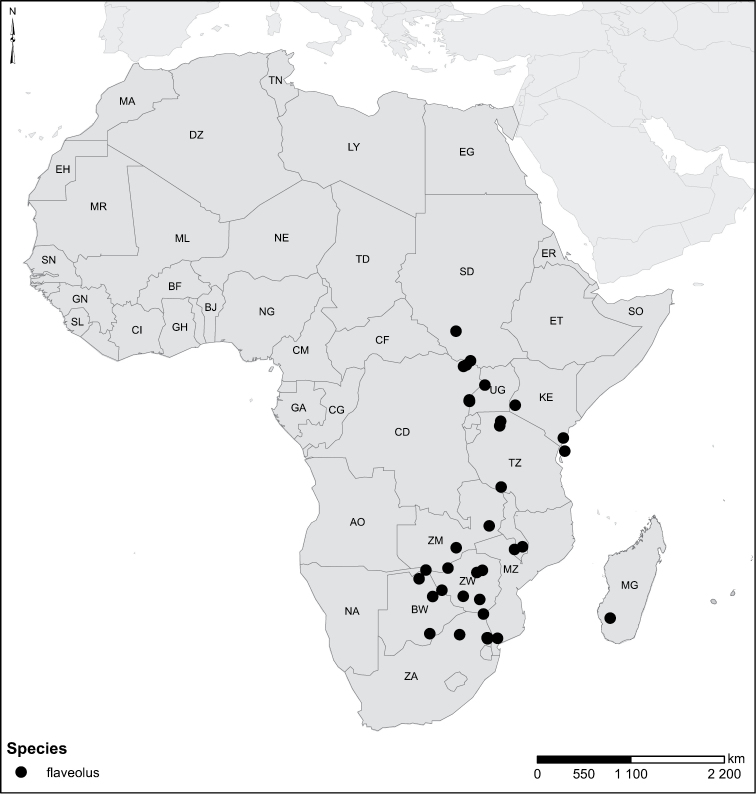
Known distribution based on examined specimens of *Laccophilus flaveolus*.

**Figure 549.**
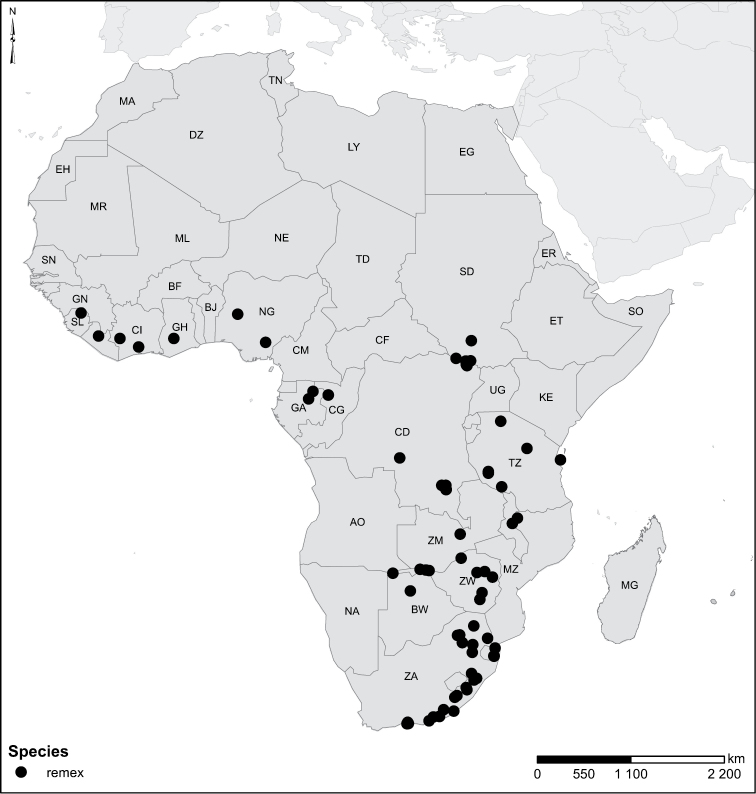
Known distribution based on examined specimens of *Laccophilus remex*.

**Figure 550.**
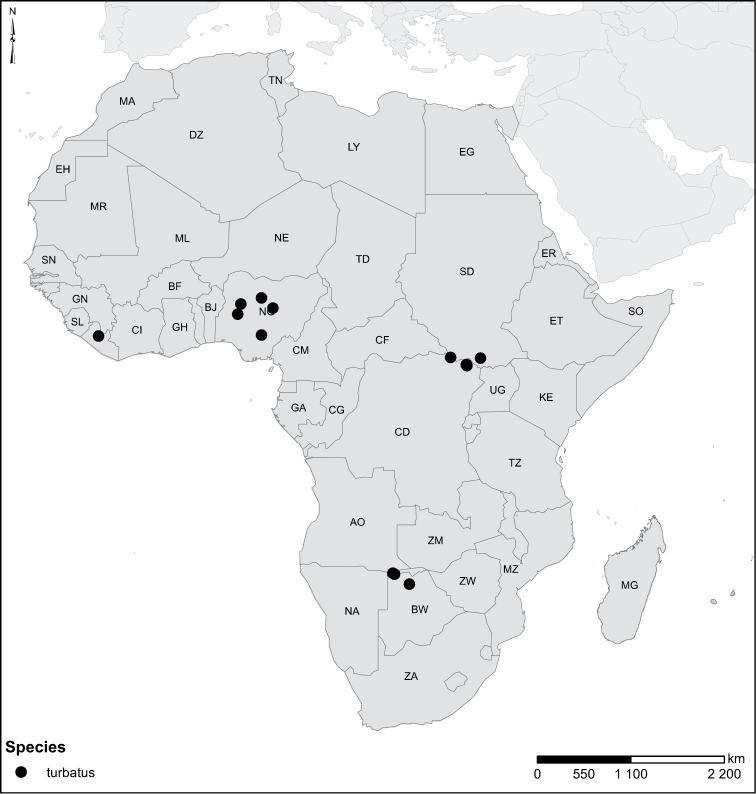
Known distribution based on examined specimens of *Laccophilus turbatus*.

**Figure 551.**
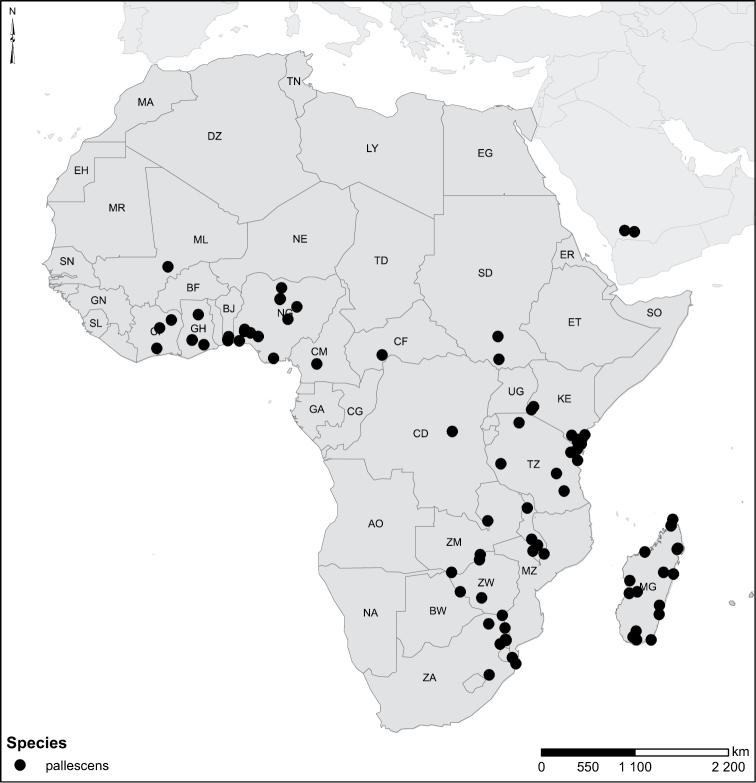
Known distribution based on examined specimens of *Laccophilus pallescens*.

**Figure 552.**
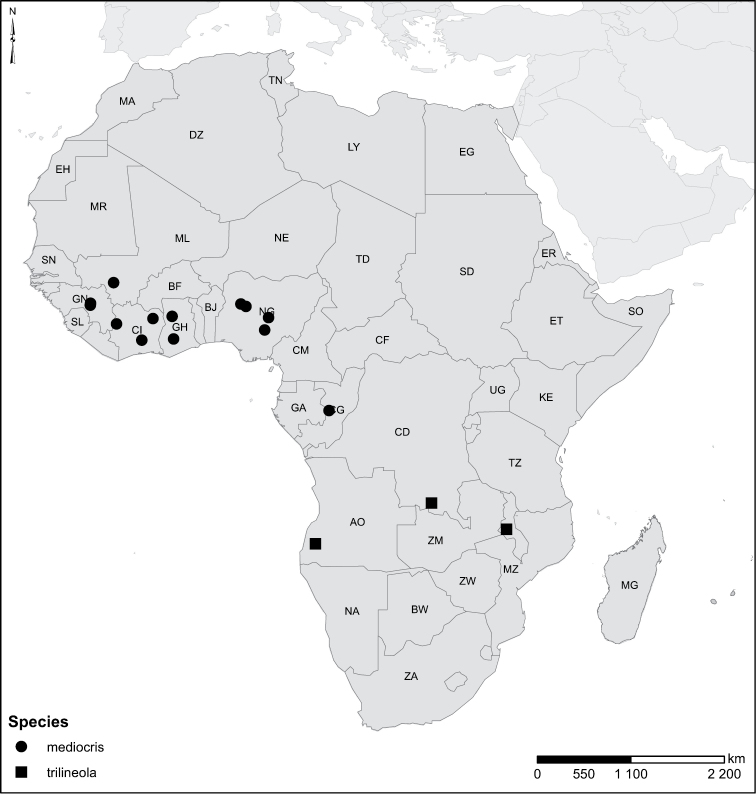
Known distribution based on examined specimens of *Laccophilus mediocris* and *Laccophilus trilineola*.

**Figure 553.**
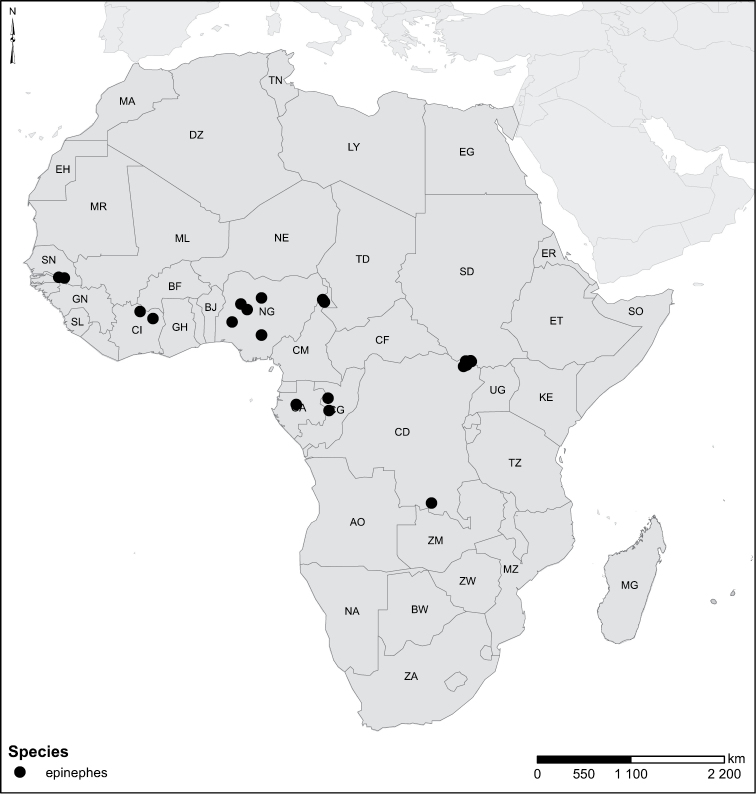
Known distribution based on examined specimens of *Laccophilus epinephes*.

**Figure 554.**
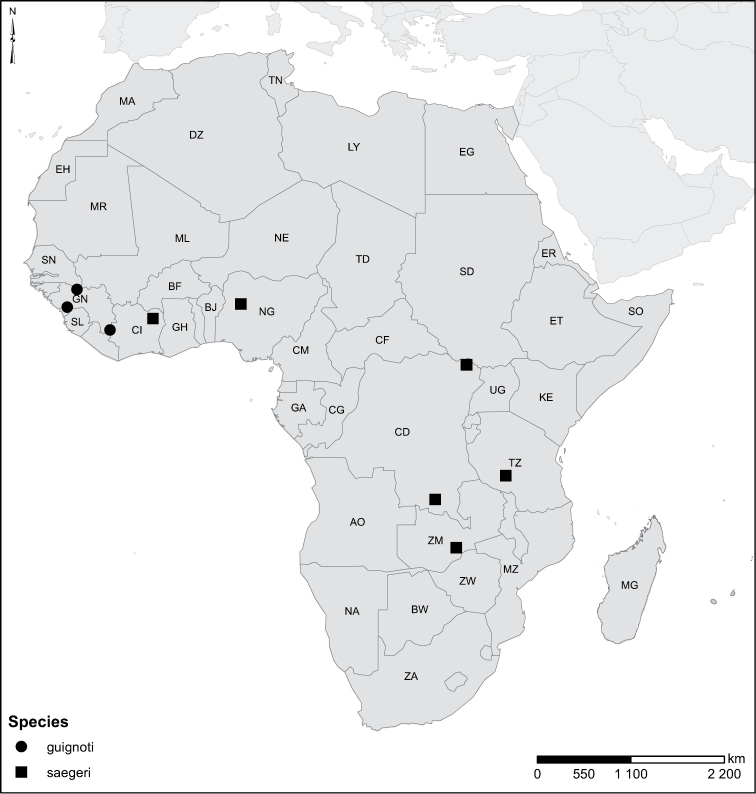
Known distribution based on examined specimens of *Laccophilus guignoti* and *Laccophilus saegeri*.

**Figure 555.**
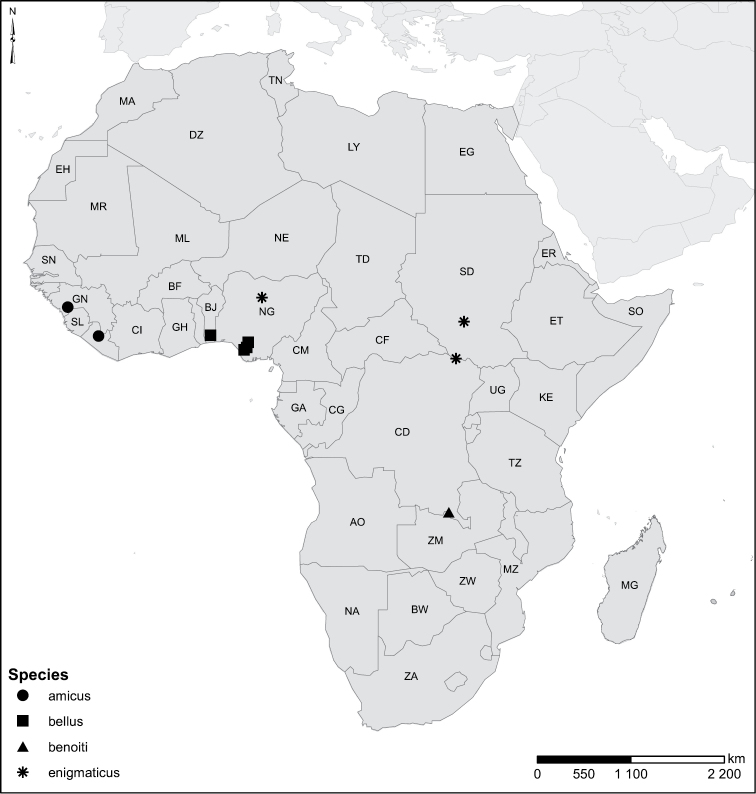
Known distribution based on examined specimens of *Laccophilus amicus*, *Laccophilus bellus*, *Laccophilus benoiti* and *Laccophilus enigmaticus*.

**Figure 556.**
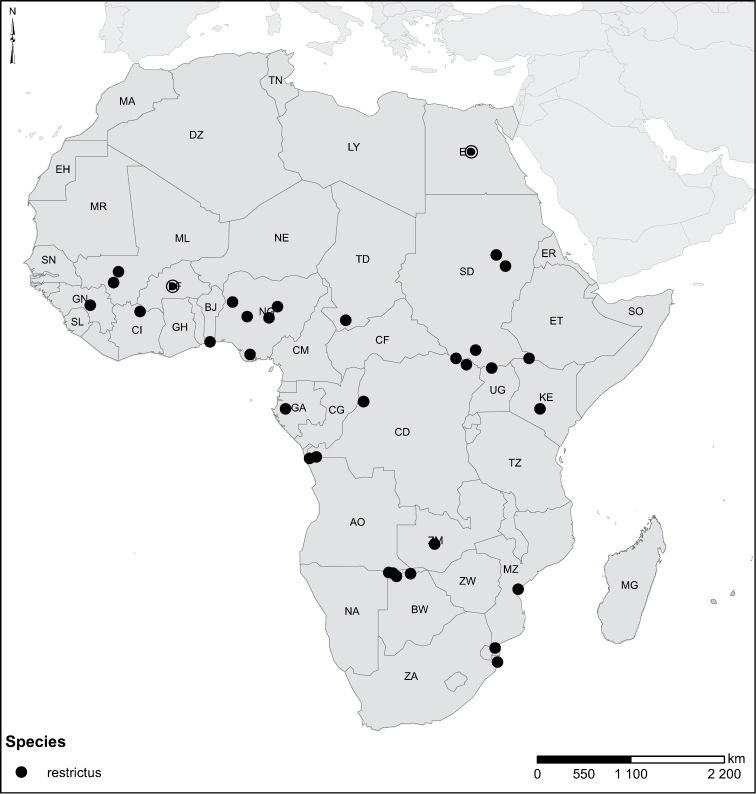
Known distribution based on examined specimens of *Laccophilus restrictus*.

**Figure 557.**
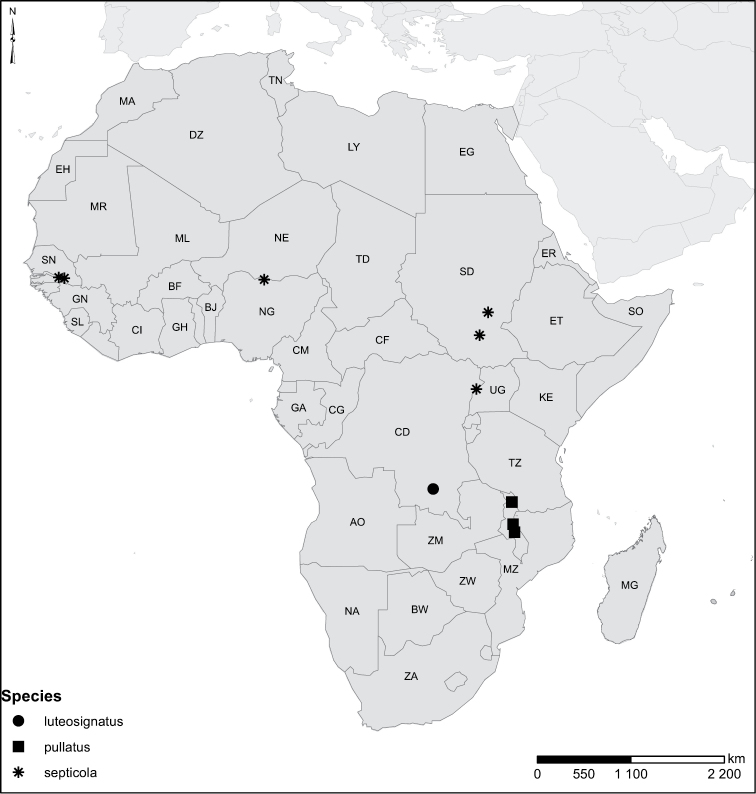
Known distribution based on examined specimens of *Laccophilus luteosignatus*, *Laccophilus pullatus* and *Laccophilus septicola*.

**Figure 558.**
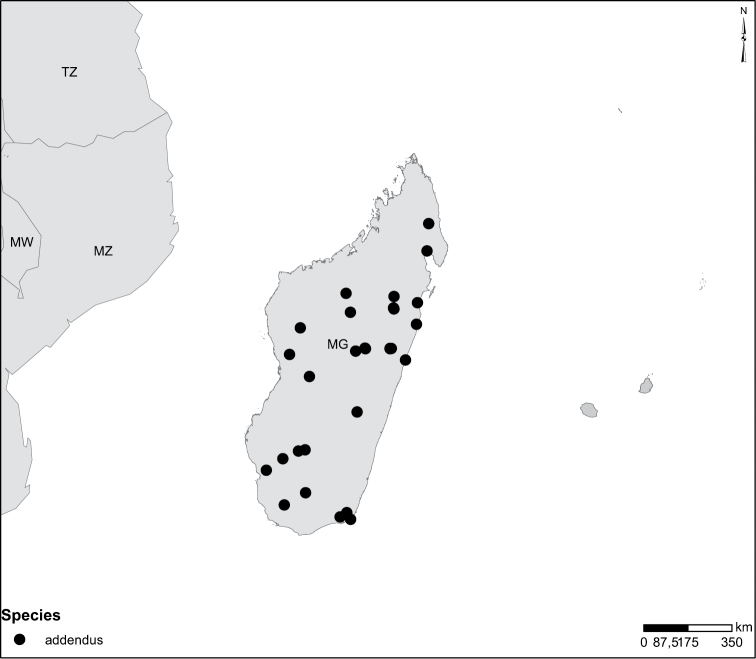
Known distribution based on examined specimens of *Laccophilus addendus*.

**Figure 559.**
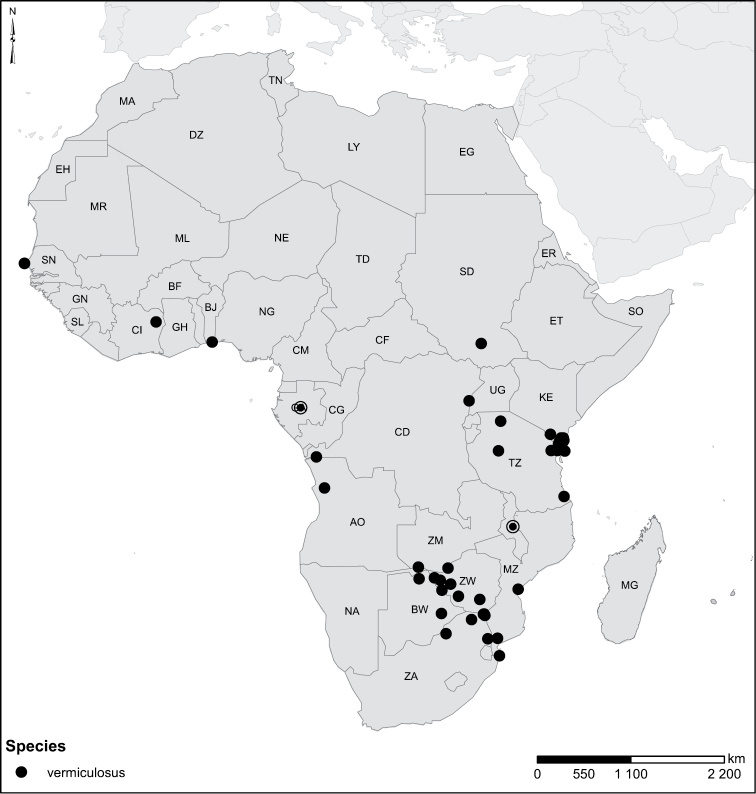
Known distribution based on examined specimens of *Laccophilus vermiculosus*.

**Figure 560.**
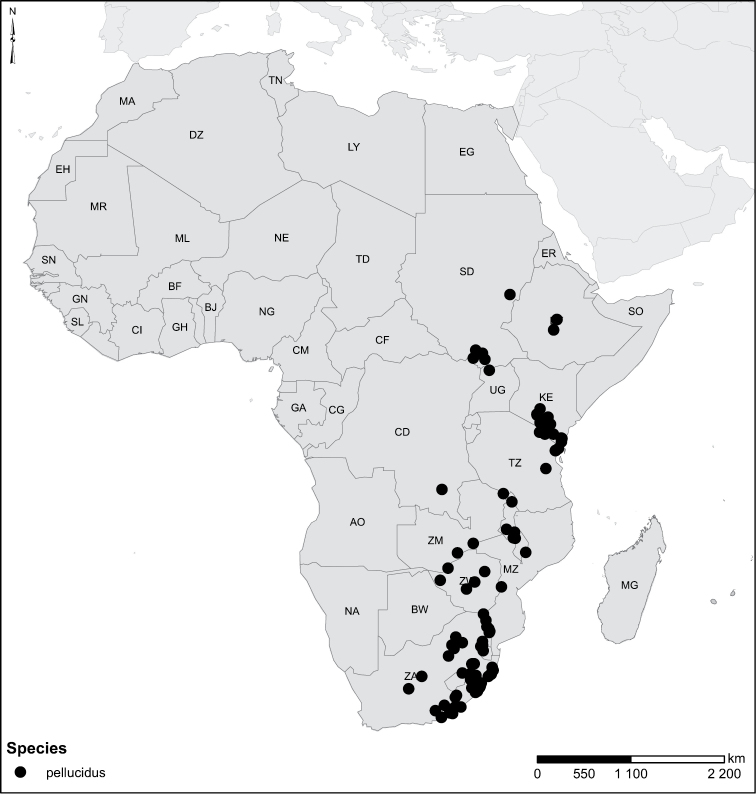
Known distribution based on examined specimens of *Laccophilus pellucidus*.

**Figure 561.**
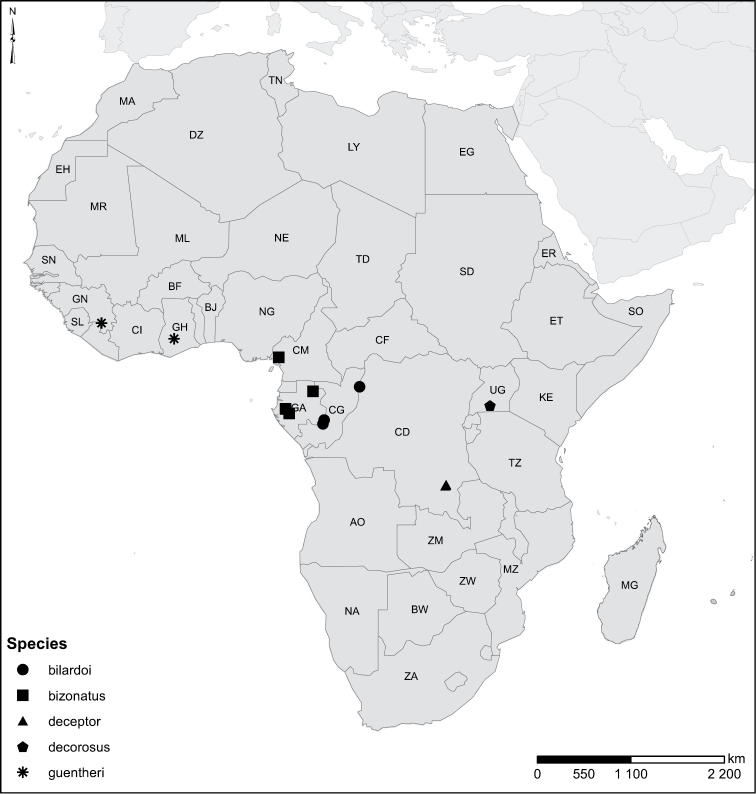
Known distribution based on examined specimens of *Laccophilus bilardoi*, *Laccophilus bizonatus*, *Laccophilus deceptor*, *Laccophilus decorosus* and *Laccophilus guentheri*.

**Figure 562.**
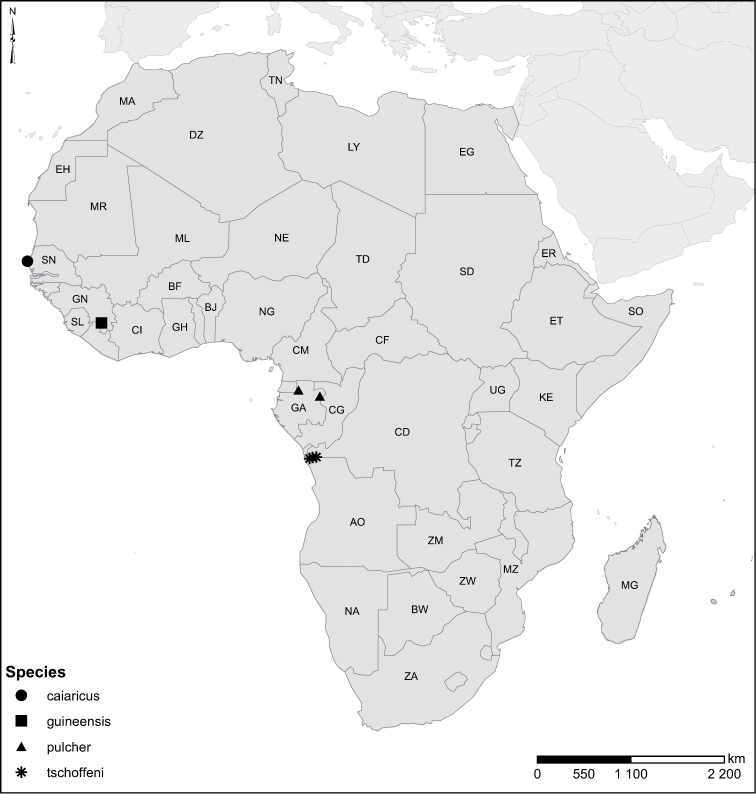
Known distribution based on examined specimens of *Laccophilus caiaricus*, *Laccophilus guineensis*, *Laccophilus pulcher* and *Laccophilus tschoffeni*.

**Figure 563.**
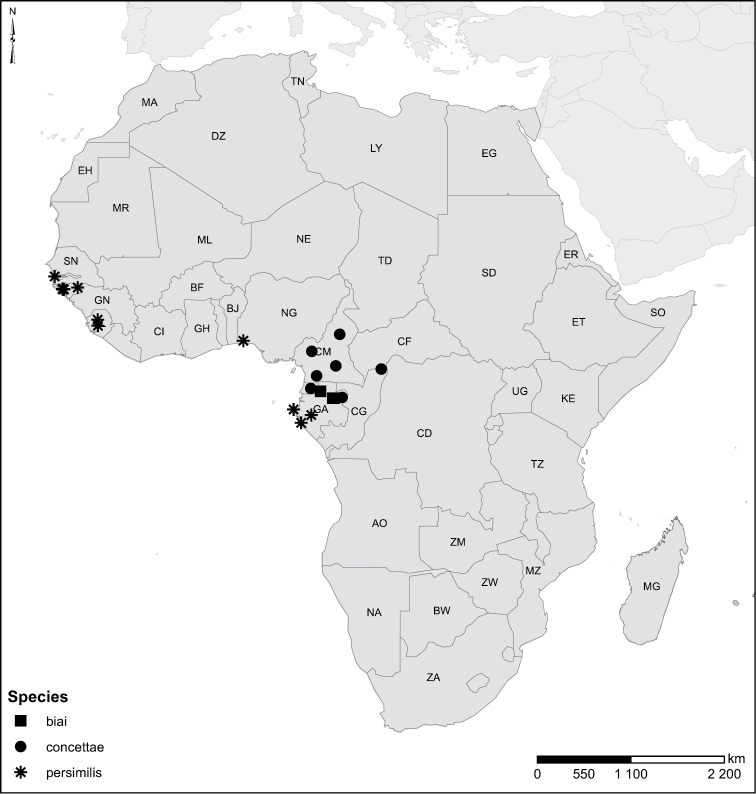
Known distribution based on examined specimens of *Laccophilus biai*, *Laccophilus concettae* and *Laccophilus persimilis*.

**Figure 564.**
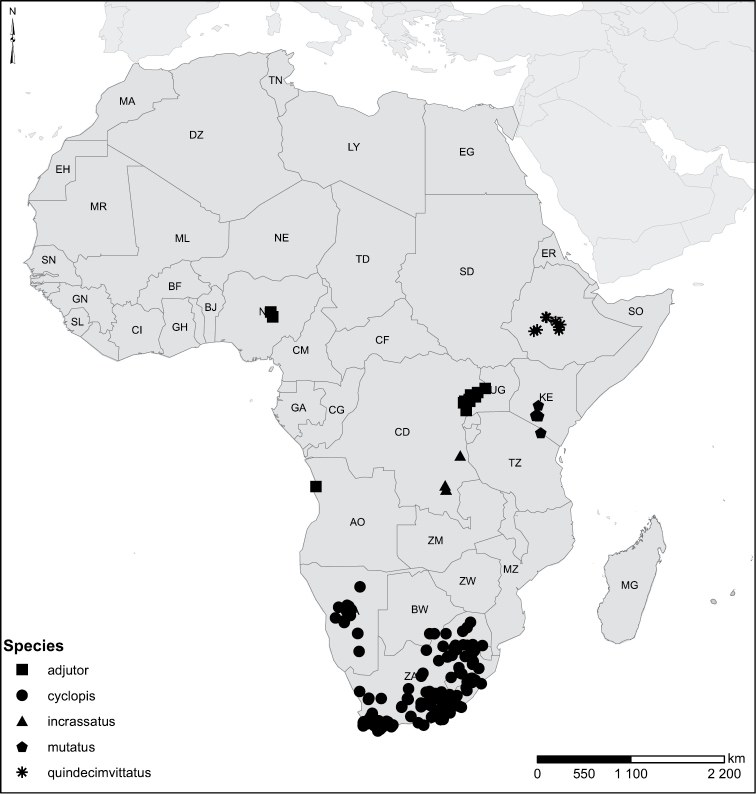
Known distribution based on examined specimens of *Laccophilus adjutor*, *Laccophilus cyclopis*, *Laccophilus incrassatus*, *Laccophilus mutatus* and *Laccophilus quindecimvittatus*.

**Figure 565.**
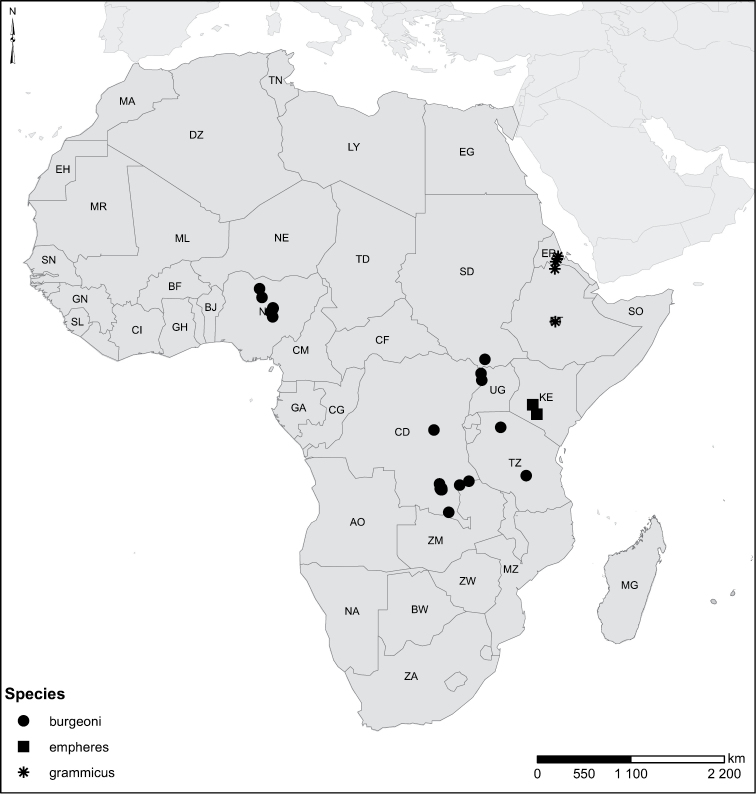
Known distribution based on examined specimens of *Laccophilus burgeoni*, *Laccophilus empheres* and *Laccophilus grammicus*.

**Figure 566.**
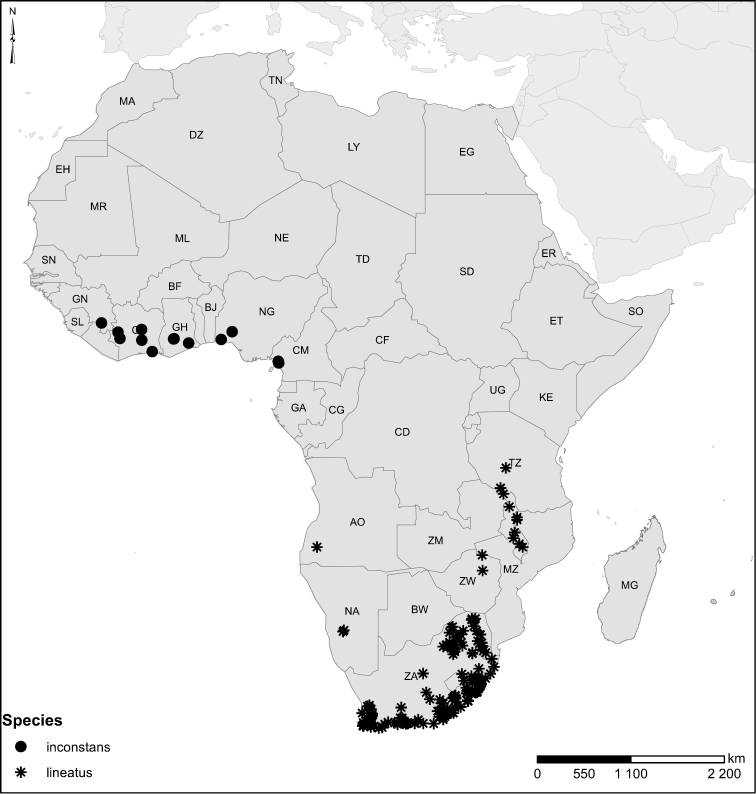
Known distribution based on examined specimens of *Laccophilus inconstans* and *Laccophilus lineatus*.

**Figure 567.**
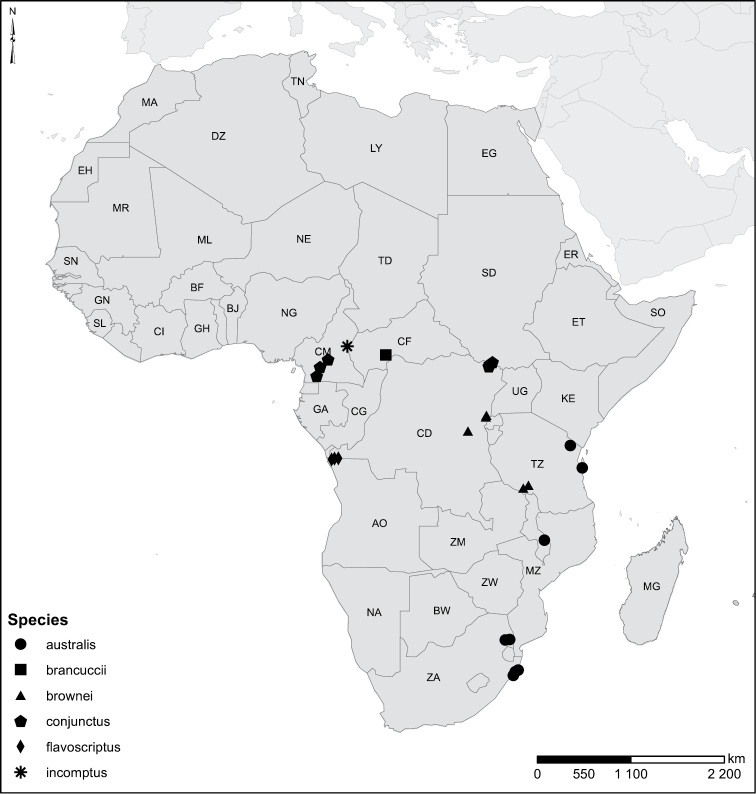
Known distribution based on examined specimens of *Laccophilus australis*, *Laccophilus brancuccii*, *Laccophilus brownei*, *Laccophilus conjunctus*, *Laccophilus flavoscriptus* and *Laccophilus incomptus*.

**Figure 568.**
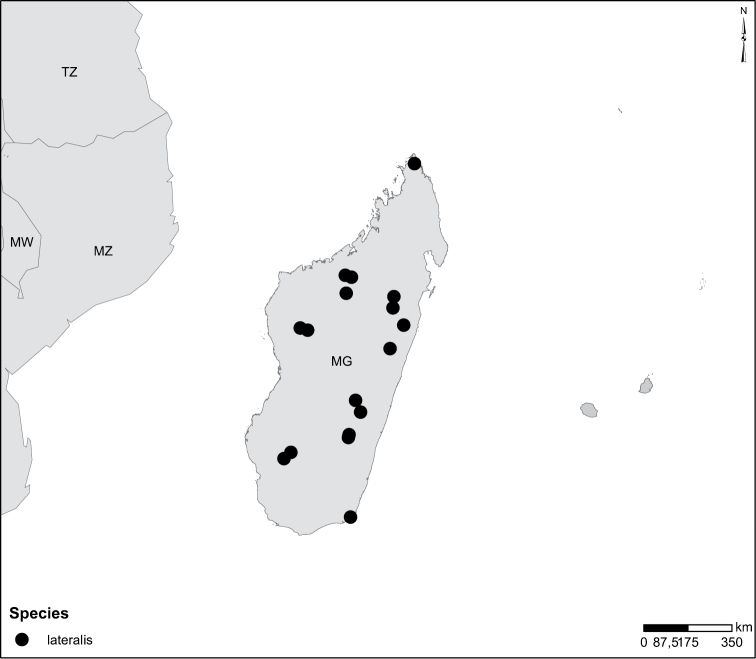
Known distribution based on examined specimens of *Laccophilus lateralis*.

**Figure 569.**
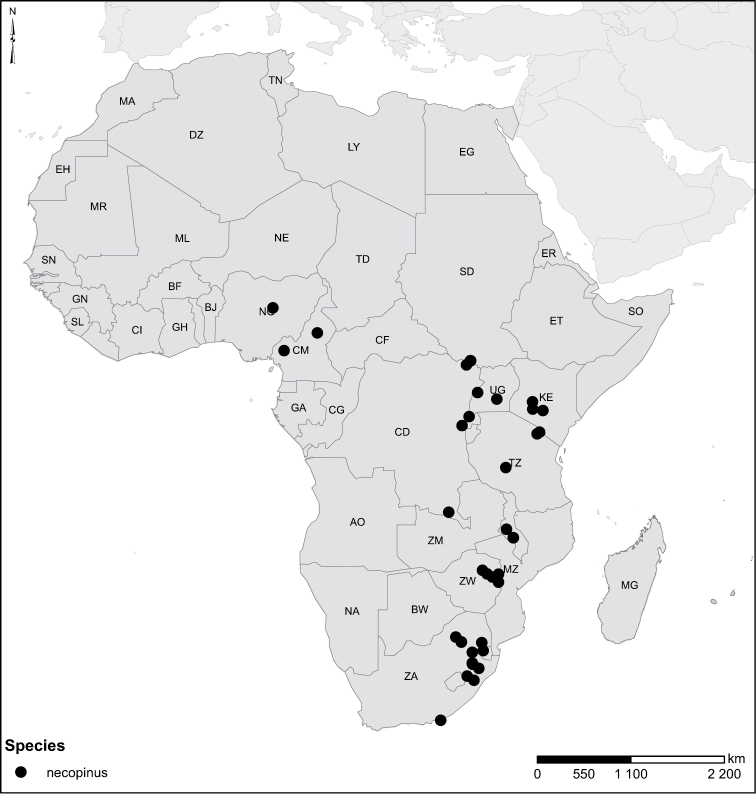
Known distribution based on examined specimens of *Laccophilus necopinus*.

**Figure 570.**
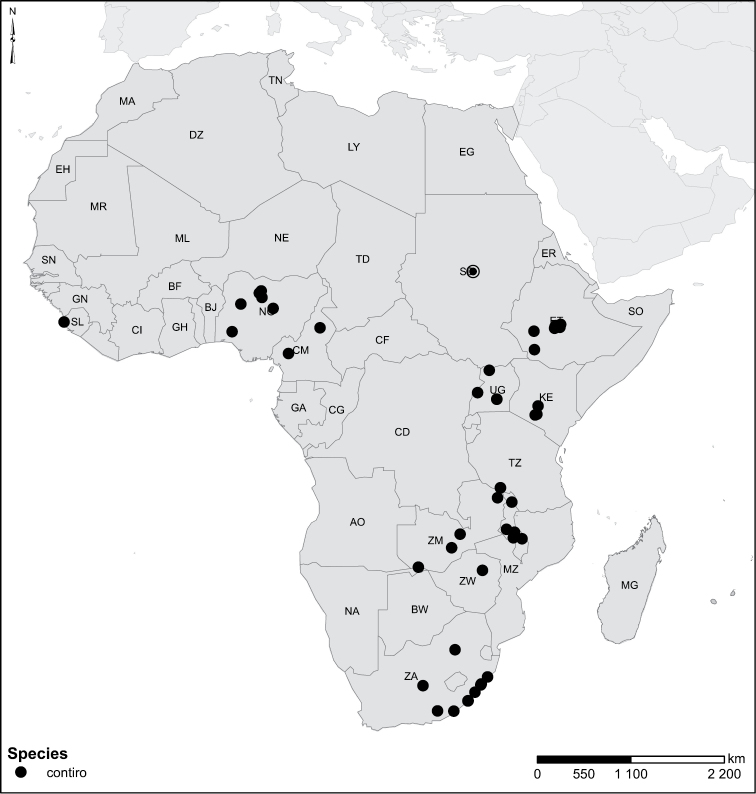
Known distribution based on examined specimens of *Laccophilus contiro*.

**Figure 571.**
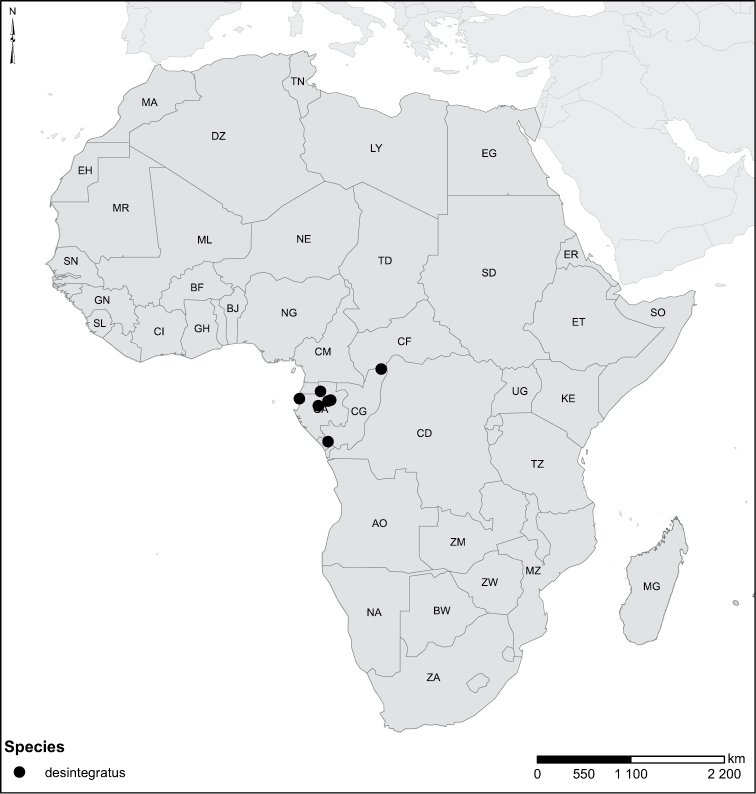
Known distribution based on examined specimens of and *Laccophilus desintegratus*.

**Figure 572.**
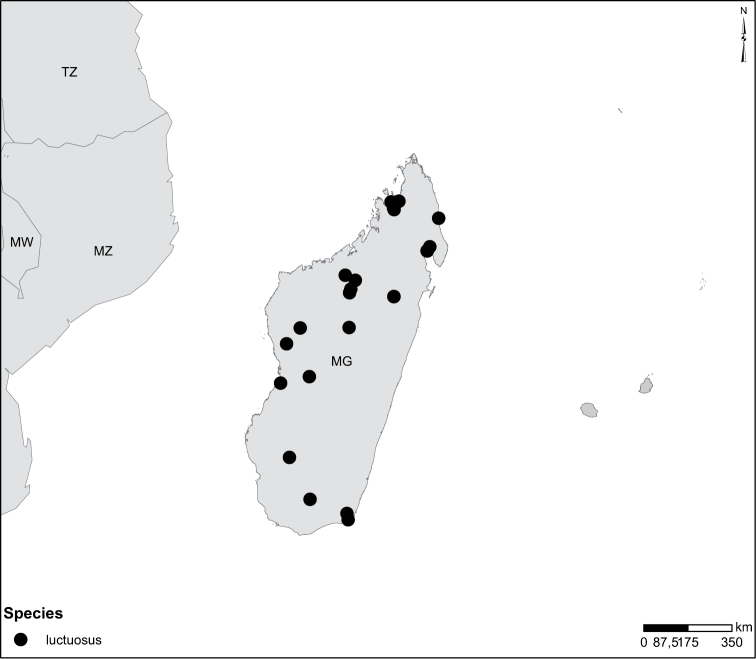
Known distribution based on examined specimens of *Laccophilus luctuosus*.

**Figure 573.**
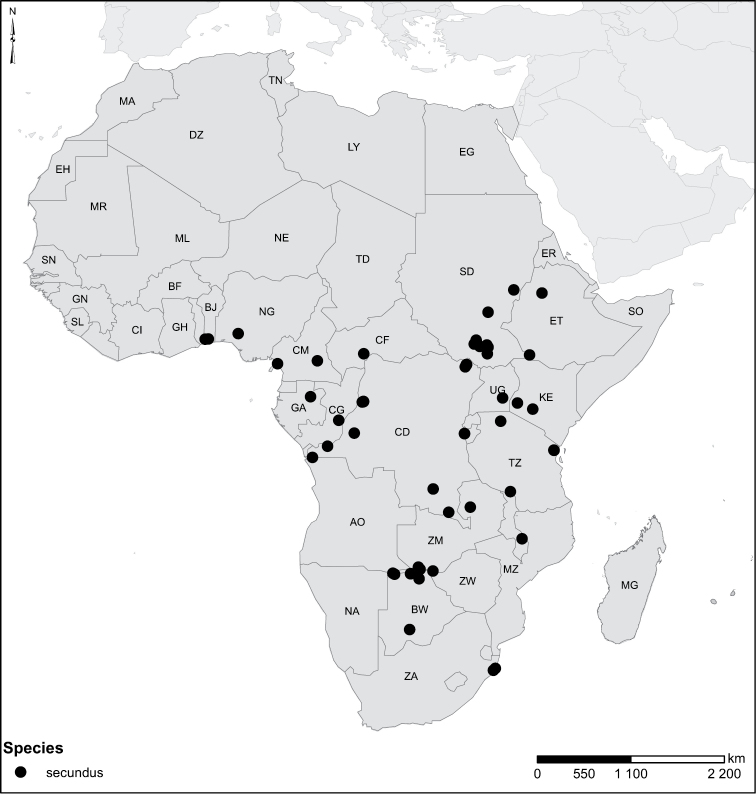
Known distribution based on examined specimens of *Laccophilus secundus*.

**Figure 574.**
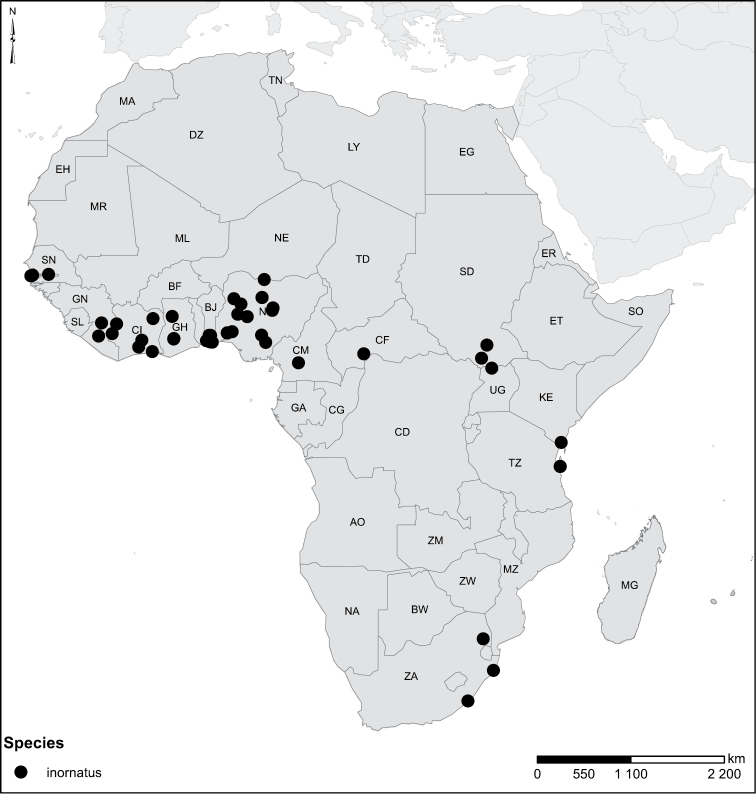
Known distribution based on examined specimens of *Laccophilus inornatus*.

**Figure 575.**
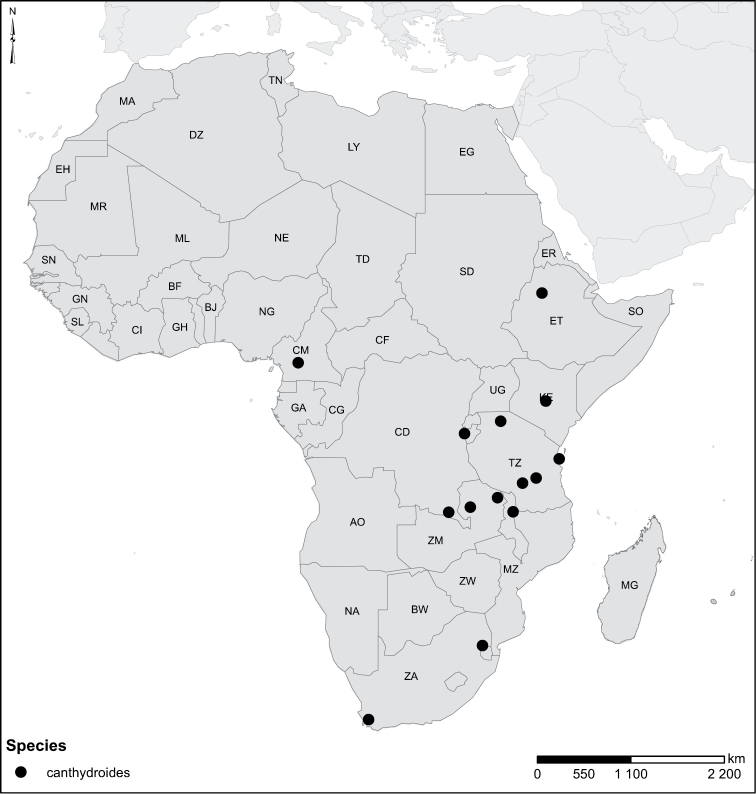
Known distribution based on examined specimens of *Laccophilus canthydroides*.

**Figure 576.**
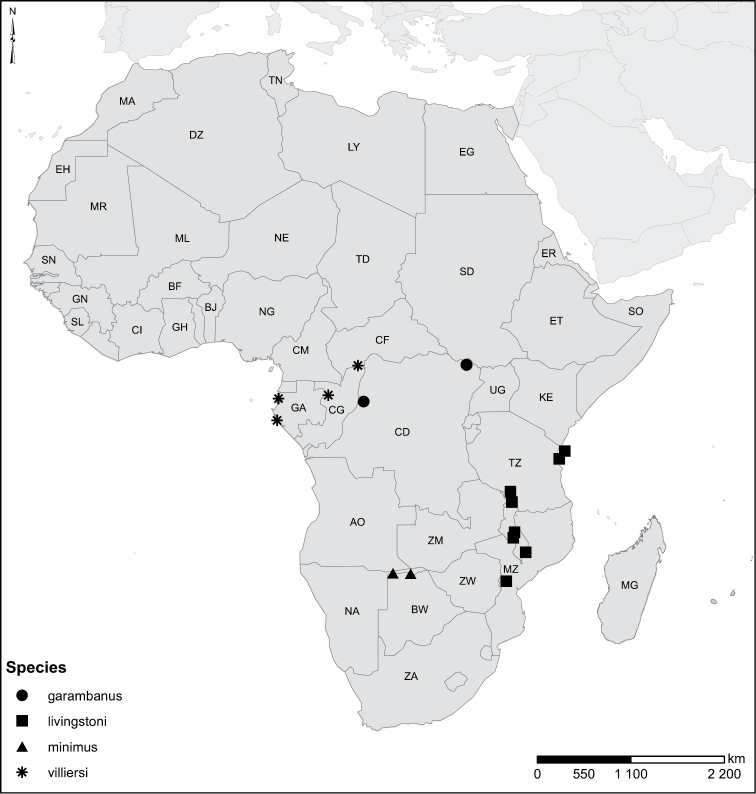
Known distribution based on examined specimens of *Laccophilus garambanus*, *Laccophilus livingstoni*, *Laccophilus minimus* and *Laccophilus villiersi*.

**Figure 577.**
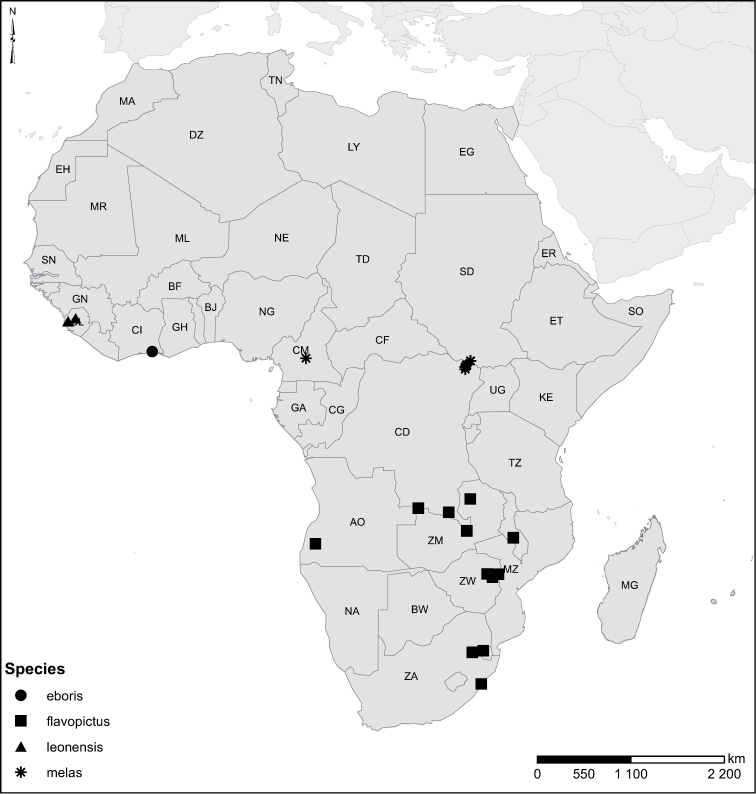
Known distribution based on examined specimens of *Laccophilus eboris*, *Laccophilus flavopictus*, *Laccophilus leonensis* and *Laccophilus melas*.

**Figure 578.**
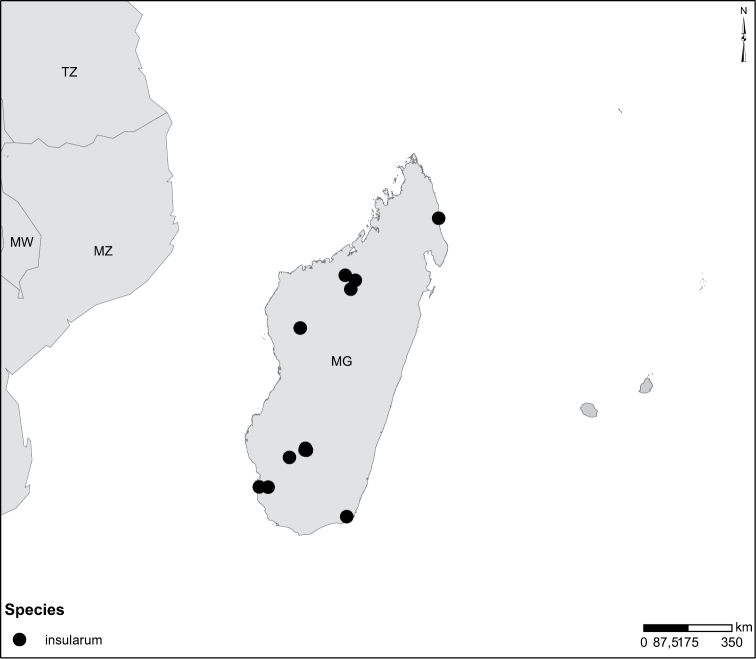
Known distribution based on examined specimens of *Laccophilus insularum*.

**Figure 579.**
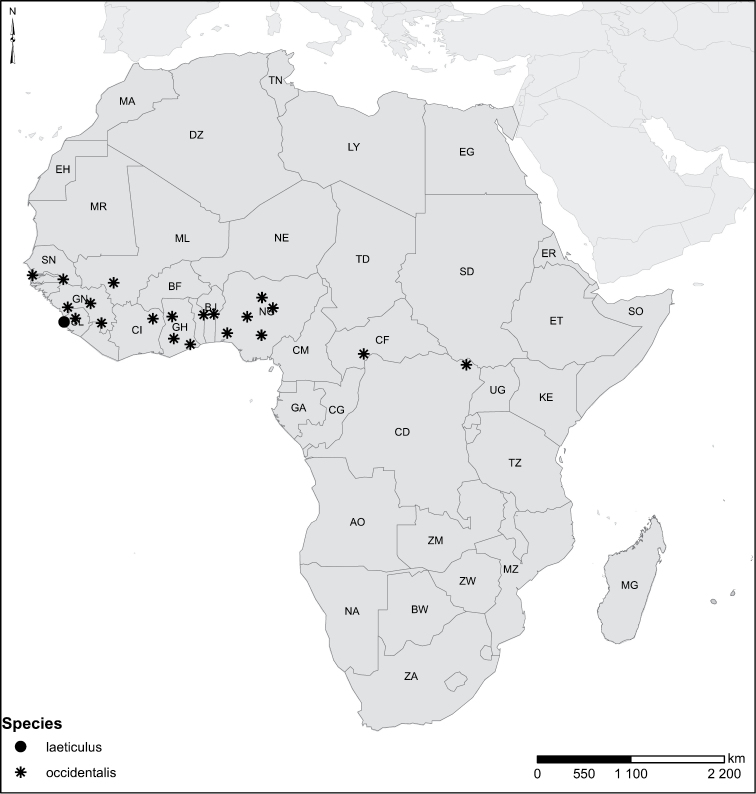
Known distribution based on examined specimens of *Laccophilus laeticulus* and *Laccophilus occidentalis*.

**Figure 580.**
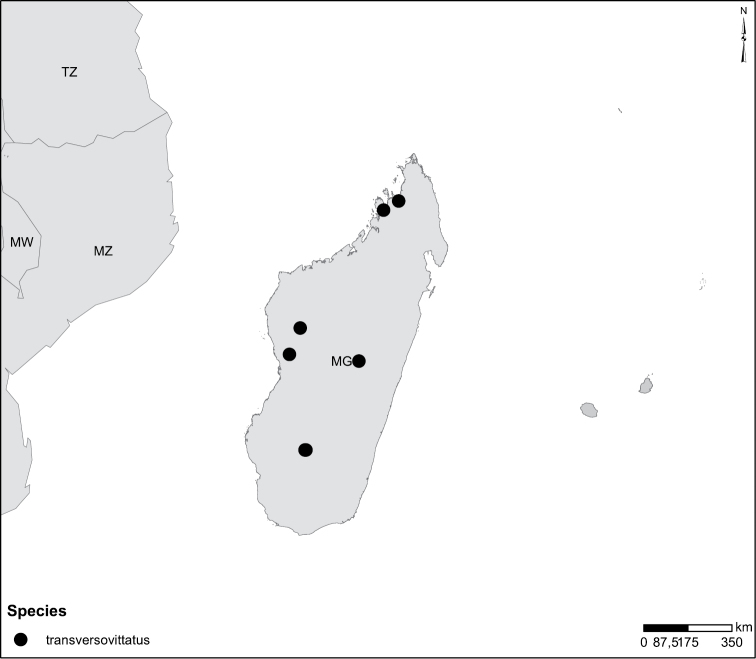
Known distribution based on examined specimens of *Laccophilus transversovittatus*.

In a few cases we have mapped country records lacking more detailed locality information. Such records are placed in the center of relevant country and are provided with a circle around the symbol (Figs 527, 528, 529, 535, 547, 556. 559 and 570).

## Index to species names of African *Laccophilus*

synonyms marked with *

*addendus* Sharp, 1882 151

*adjutor* Guignot, 1950 196

*adspersus* Boheman, 1848 97

**alberticus* Guignot, 1959 145

*alluaudi* Régimbart, 1900 39

*amicus* Guignot, 1955 143

**ampliatus* Régimbart, 1895 90

*australis* sp. n. 230

*bellus* sp. n. 144

*benoiti* Guignot, 1953 150

**bergeri* Guignot, 1953 254

*biai* Bilardo & Rocchi, 1990 167

*bilardoi* Pederzani & Rocchi, 1982 170

*bizonatus* Régimbart, 1895 163

*brancuccii* sp. n. 223

**brevicollis* Sharp, 1882 215

*brownei* Guignot, 1947 202

*burgeoni* Gschwendtner, 1930 212

*caiaricus* Guignot, 1956 175

*canthydroides* Omer-Cooper, 1957 242

**castaneus* Guignot, 1956 134

*comes* Guignot, 1955 37

**comoensis* Pederzani & Reintjes, 2002 136

*complicatus* Sharp, 1882 80

*concettae* Pederzani, 1983 165

**concisus* Guignot, 1953 119

**congener* Omer-Cooper, 1957 72

*conjunctus* Guignot, 1950 201

*continentalis* Gschwendtner, 1935 53

*contiro* Guignot, 1952 204

*cryptos* sp. n. 111

*cyclopis* Sharp, 1882 189

*deceptor* Guignot, 1953 168

*decorosus* sp. n. 171

*demoflysi* Normand, 1938 31

*desintegratus* Régimbart, 1895 232

*eboris* sp. n. 244

*empheres* sp. n. 185

*enigmaticus* sp. n. 138

*epinephes* Guignot, 1955 134

**espanyoli* Hernando, 1990 107

**evanescens* Régimbart, 1895 140

*ferrugo* sp. n. 25

*flaveolus* Régimbart, 1906 115

*flavopictus* Régimbart, 1889 254

*flavoscriptus* Régimbart, 1895 210

**flavosignatus* Régimbart, 1895 210

*furthi* sp. n. 42

*garambanus* Guignot, 1958 253

**geminatus* Régimbart, 1895 151

*grammicus* Sharp, 1882 208

*grossus* sp. n. 19

*guentheri* sp. n. 160

*guignoti* Legros, 1954 157

*guineensis* sp. n. 161

**gutticollis* Régimbart, 1895 232

*hyalinus* (De Geer, 1774) 28

*immundus* Sharp, 1882 87

*incomptus* sp. n. 224

*inconstans* sp. n. 207

*incrassatus* Gschwendtner, 1933 184

**inflatus* Wollaston, 1864 29

*inobservatus* sp. n. 63

*inornatus* Zimmermann, 1926 238

*insularum* sp. n. 251

*irroratus* Aubé, 1838 83

*isamberti* sp. n. 47

*laeticulus* Régimbart, 1895 257

*lateralis* Sharp, 1882 187

*leonensis* Régimbart, 1895 246

*lineatus* Aubé, 1838 214

**livens* Régimbart, 1895 98

*livingstoni* Omer-Cooper, 1958 250

*luctuosus* Sharp, 1882 235

*luteosignatus* Gschwendtner, 1943 148

*mateui* Omer-Cooper, 1970 33

*mediocris* Guignot, 1952 132

**meii* Rocchi, 2000 132

*melas* Guignot, 1958 248

*minimus* sp. n. 243

*minutus* (Linnaeus, 1758) 31

*mirabilis* Guignot, 1956 24

**mocquerysi* Régimbart, 1895 154

*modestus* Régimbart, 1895 107

**monas* Guignot, 1953 66

*morondavensis* Guignot, 1957 22

*mutatus* Omer-Cooper, 1970 181

*necopinus* Guignot, 1942 199

**nigeriensis* Omer-Cooper, 1970 98

*nodieri* Régimbart, 1895 113

*occidentalis* sp. n. 259

*olsoufieffi* Guignot, 1937 105

*pallescens* Régimbart, 1903 125

**pampinatus* Guignot, 1941 115

*pellucidus* Sharp, 1882 90

**perplexus* Omer-Cooper, 1970 53

*persimilis* Régimbart, 1895 174

*pictipennis* Sharp, 1882 50

**pilitarsis* Régimbart, 1906 90

*poecilus* Klug, 1834 177

**polygrammus* Régimbart, 1903 187

**ponticus* Sharp, 1882 177

*posticus* Aubé, 1838 58

**praeteritus* Omer-Cooper, 1957 119

*productus* Régimbart, 1906 23

*propinquus* Omer-Cooper, 1958 79

*pseustes* Guignot, 1955 44

*pulcher* Bilardo & Rocchi, 2004 164

*pullatus* Omer-Cooper, 1958 147

*quindecimvittatus* Régimbart, 1895 182

*remex* Guignot, 1952 118

*restrictus* Sharp, 1882 140

*rivulosus* Klug, 1833 85

*rocchii* sp. n. 20

*ruficollis* Zimmermann, 1919 27

*saegeri* Guignot, 1958 136

**sanguinosus* Régimbart, 1895 232

*secundus* Régimbart, 1895 226

**segmentatus* Omer-Cooper, 1957 254

*septicola* Guignot, 1956 145

*seyrigi* Guignot, 1937 46

**shephardi* Omer-Cooper, 1965 190

*simplicistriatus* Gschwendtner, 1932 66

**simulator* Omer-Cooper, 1958 130

*sordidus* Sharp, 1882 35

**spadix* Omer-Cooper, 1953 87

**sudanensis* Omer-Cooper, 1970 99

*taeniolatus* Régimbart, 1889 72

*tavetensis* Guignot, 1941 18

**testaceus* Aubé, 1837 29

*tigrinus* Guignot, 1959 43

**torquatus* Guignot, 1956 226

**tostus* Régimbart, 1895 107

*transversovittatus* sp. n. 260

*trilineola* Régimbart, 1889 130

*tschoffeni* Régimbart, 1895 172

*turbatus* Guignot, 1958 123

**turneri* Omer-Cooper, 1956 119

**variegatus* Germar & Kaulfuss, 1812 177

*vermiculosus* Gerstaecker, 1867 154

*villiersi* Bertrand & Legros, 1975 247

**virgatus* Guignot, 1953 184

**vitshumbii* Guignot, 1959 98

**wehnckei* Sharp, 1882 50

**wittei* Guignot, 1952 212

## Supplementary materials

Supplementary material 1 Georeferenced locality data for studied African *Laccophilus* species Data type: Occurrence data with coordinates. Explanation note: Tabulation of locality data with coordinates including all species records mapped in our revision of African *Laccophilus* (Coleoptera: Dytiscidae). Coordinates given as decimal latitude and longitude. This dataset is made available under the Open Database License (http://opendatacommons.org/licenses/odbl/1.0/). The Open Database License (ODbL) is a license agreement intended to allow users to freely share, modify, and use this Dataset while maintaining this same freedom for others, provided that the original source and author(s) are credited.Olof Biström, Anders N. Nilsson, Johannes Bergsten
